# Species identification of silks by protein mass spectrometry reveals evidence of wild silk use in antiquity

**DOI:** 10.1038/s41598-022-08167-3

**Published:** 2022-03-17

**Authors:** Boyoung Lee, Elisabete Pires, A. Mark Pollard, James S. O. McCullagh

**Affiliations:** 1 Department of Chemistry, University of Oxford, Mansfield Road, Oxford, OX1 3TA UK; 2 Research Laboratory for Archaeology and the History of Art, University of Oxford, 1 South Parks Road, Oxford, OX1 3TG UK; 3 Smithsonian Institution, Museum Conservation Institute, Suitland, MD 20746 USA

**Keywords:** Analytical biochemistry, Mass spectrometry, Proteomic analysis, Biomaterials, Techniques and instrumentation

## Abstract

Silk has been a luxurious commodity throughout modern human history and sericulture has played an important role in ancient global trade as well as technological and cultural developments. Archaeological findings suggest that prior to domestication of the mulberry silkworm (*Bombyx* *mori*) silks were obtained from a range of silk-producing moth species with regional specificity. However, investigating the origins of sericulture is difficult as classification of silks by species-type has proved technically challenging. We therefore investigated a range of methods for solubilising modern and archaeological silks and developed a mass spectrometry-based proteomics method that was able to successfully differentiate modern *Bombyx,* *Antheraea,* and *Samia*-produced silks down to the species level. We subsequently analysed archaeological silk materials excavated from the ancient city of Palmyra. Solubilisation behaviour and proteomic analysis provided evidence that the Palmyra silks were constructed from wild silk derived from *Antheraea* *mylitta*, the Indian Tasar silkworm. We believe this is the first species-level biochemical evidence that supports archaeological theories about the production and trade of Indian wild silks in antiquity.

## Introduction

**Figure 1:**
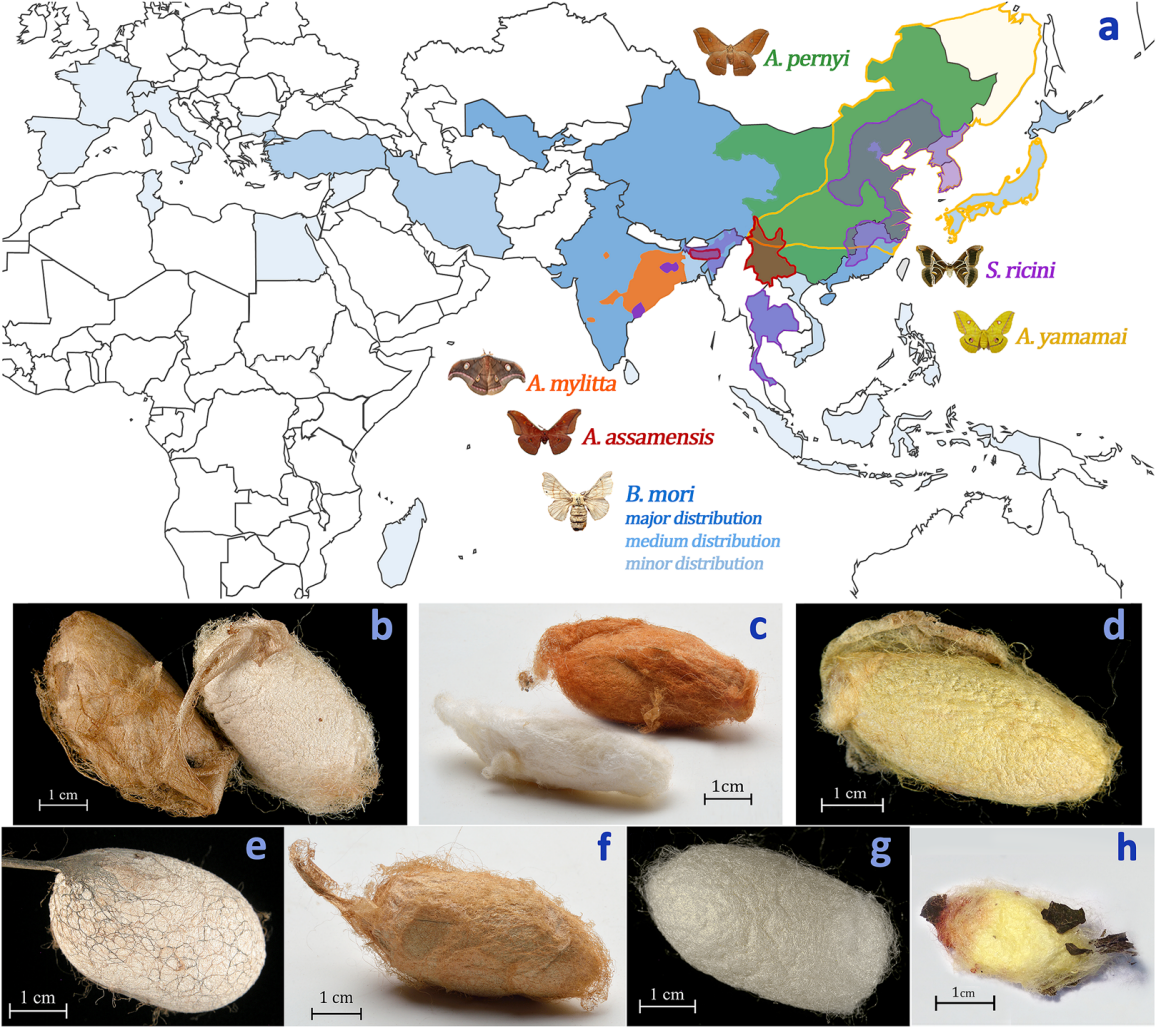
Distribution of silk moths by their indigenous habitats **(a)** and their cocoons **(b–h)**. **(a)** Areas marked in colors correspond to habitats of silk moths: green—*A.* *pernyi* **(b)** is indigenous to southern China but was introduced to sub-tropical and tropical Asia for wild silk production; purple—*S. ricini* (**c**) is indigenous to Korea and eastern China, though it naturally spread to parts of India and was introduced to Thailand in the 1970s for commercial silk production; yellow—*A. yamamai* **(d)** is endemic to east Asia but was also introduced unsuccessfully to southeastern Europe for cultivation (not included here); orange—*A.* *mylitta* **(e)** is endemic to the northeastern region of India; red—*A.* *assamensis* **(f)** is indigenous to the Assam region of India and naturally immigrated to southern China; blue—*B. mori* (**g**) was domesticated from its wild precursor *B. mandarina* (**h**) in southern China and is now widespread from Asia to Europe. This figure was created by the first author using photographs of samples and opensource images edited with Adobe Photoshop CS6.

Silk is a fibrous protein commonly produced by the domesticated mulberry silkworm, *Bombyx* *mori* and the first evidence of its use to make silk textiles dates back to the Chinese Neolithic Period around 6000 years ago^1^. Historical evidence suggests that in parallel, or prior to this, a range of silkworm species may have been used for silk harvesting and textile production^2–4^. The non-*Bombyx* wild silks are likely to have been derived directly from wild or semi-cultivated silkworms^5^. To this day, various species of wild silk are used for textile production, such as those produced by *Antheraea* and *Samia* silkworms. These species are adapted to living in different climatic conditions (from tropical to temperate), inhabiting most of Asia with regional dependency (Fig. 1). Thus, species identification of silk materials is likely to provide insights into their provenance and trade. A wealth of alleged silk materials have been found at archaeological sites located on or near the ancient Silk Road, where it is known that silks were an important commodity linking Asia with the Middle East and Europe for almost 2000 years^1^. Species identification of these alleged silks will provide a better understanding of the production of silk in antiquity and prehistory, informing on textile use, technology development, and the origin and development of the silk trade.

Fibre analysis for species identification has traditionally relied on morphological observations using light microscopy, scanning electron microscopy (SEM) and chemical characterisation using Fourier-transform infrared spectroscopy (FTIR)^6^. However, species identification from archaeological fibres has proven challenging using these methods (Supplementary Fig. S1). Well-established test standards for fibre identification by microscopic methods place the greatest importance on the cross-sectional shape of the natural fibres^7,8^, but it is difficult to capture subtle morphological differences between silk species, particularly in deteriorated samples that are commonly found among historical and archaeological textiles. The misidentification of silks from prehistory and antiquity using these methods can be found in several standard texts on the European Iron Age in the context of interpretating trade links^9^. To partially address the challenge of sample deterioration, proteomic approaches have been recently applied, including amino acid analysis^10,11^, liquid-chromatography tandem mass spectrometry (LC–MS/MS)^12–14^, and enzyme-linked immunosorbent assay (ELISA)^13,15^. While these techniques provide some evidence that domesticated *B.* *mori* silk proteins can be identified from highly degraded archaeological samples, differences in individual amino acid abundance were not reliable for distinguishing silk species since degradation-driven effects on amino acid abundances are more likely to play a dominant role compared to gene-encoded species differences^16^. ELISA, which relies on the detection of a known, specific, and intact amino acid peptide sequence using a specific antibody, is a promising technique, but reported studies have used purified polyclonal antibody sera, which has the potential for cross-reactivity and false-positive results^15,17^. In a study applying ELISA to the differentiation of *A.pernyi* from *B.* *mori*, an antibody specific to the wild silk C-terminal peptide (CSHSHSYEASRISVH) was used^13^. However, it is not known if this peptide will always be well-preserved in degraded archaeological samples, as the degradation behaviour of silk fibroin is poorly understood. The identification of proteins by tandem mass spectrometry (MS/MS) enables the determination of amino acid sequences, which can be matched to a specific protein at the species-level via genetic and proteomic databases^18^. It is also possible to observe altered amino acid sequences in the same protein across different species that result from genetic drift and speciation^19,20^. For silk, the various silk proteins of the *Bombycidae* and *Saturniidae* families have been sequenced and made available in public databases including UniProt and the National Center for Biotechnology Information (NCBI). However, the primary barrier to routine, effective proteomic analysis of silks is currently their lack of solubility. The combination of hydrogen bonding, high hydrophobicity, and crystalline regions of wild silk fibroins makes them particularly resistant to solubilisation in most aqueous or organic solvents^21–24^, which renders them not-amenable to many conventional proteomics sample preparation protocols that involve solubilisation and denaturation of proteins prior to enzymatic digestion using a protease.

**Figure 2:**
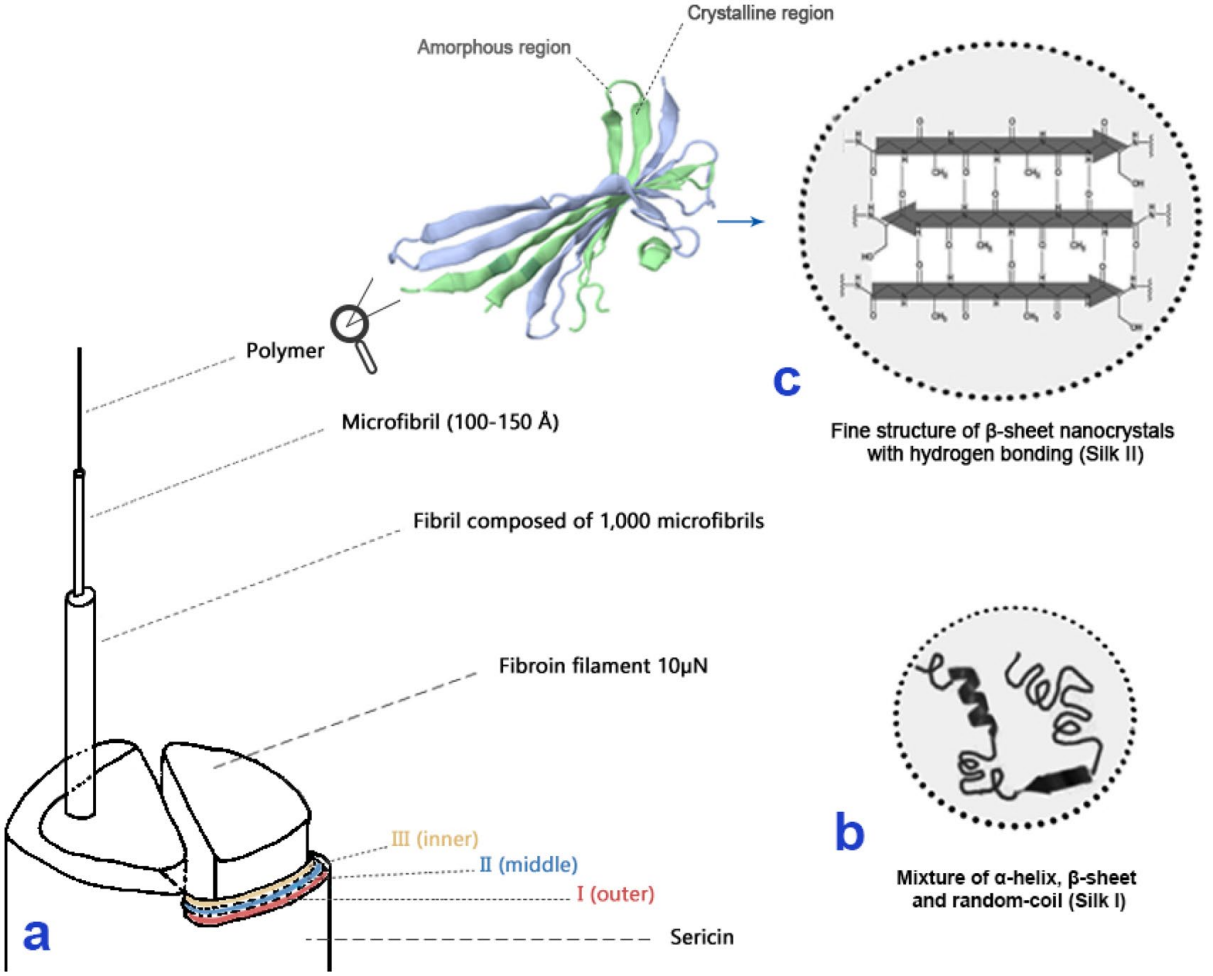
Structural hierarchy of a silk fibre and schematic crystal structure of silk fibroin (based on *B.* *mori*)**.** **(a)** Structural hierarchy of a moth silk fibre from macro to micro scale. A moth silk fibre is composed of two fibroin filaments covered in sericin. The 3D model of a fibroin polymer is generated by Proteopedia (proteopedia.org)**.** **(b)** A representation of the secondary structure of liquid Silk I (present in the silk glands of the silk moth before extrusion)**.** **(c)** A representation of the secondary structure of secreted, solid Silk II. This figure was created by the first author using original drawing and opensource images edited with Adobe Photoshop CS6.

Silk is composed of the two proteins sericin and fibroin, which account for about 25 wt% and 75 wt%, respectively^25^. Sericin is a gummy protein that envelopes two fibroin filaments to form a bundle (Fig. 2). The protein composition of sericin is 76% hydrophilic chains, and it is often stripped from the fibroin bundle by hot water and mild chemical treatments in preparation for silk production (the reeling process, known as degumming)^26^. Fibroin is a fibrous protein, such as collagen and keratin, possessing a hydrophobic protein structure organized into a natural block co-polymer^25,27^. It is rich in short glycine and alanine residues, which allows for close packing of *β*-sheets and an interlocking arrangement of amino acid R-groups^28^. Once the silk fibre is spun and secreted, fibroins behave similarly to thermoset polymers and do not tend to re-solubilise^24^. However, domesticated *B.* *mori* silks generally appear more amenable to solubilisation than wild silks; the latter do not solubilise under the same conditions^21,24,29^. It has been reported that *B.* *mori* silk is soluble in a ternary solution of calcium chloride^30^ and saturated solutions of chaotropic salts such as lithium bromide^31^, lithium thiocyanate^32^, and calcium nitrate^24,33^. The relative ease of solubilising *B.* *mori* silk has enabled further study of its fibroin and exploration of its biocompatibility and mechanical performance^34,35^. Structural studies of wild cocoon silks are not as well established, however, due mainly to their lack of solubilisation. It is known that they have considerably different protein structures and mechanical properties^36–38^. For example, *Bombyx* fibroin possesses two polypeptide chains, the light chain (LC, 30 kDa) and the fibrohexamerin (P25, 25 kDa)^39^, in addition to the heavy chain (HC, 390 kDa), while *Antheraea* and *Samia* fibroins are only comprised of HC coupled by disulfide bonds^40^. The most distinctive feature of *Bombyx* silk fibroin is the repetitive glycine-alanine hexapeptide GAGAG(X) of the HC^41^, where X is S, A, Y, T, V, or G (Supplementary Table S1). This arrangement makes up more than 80% of the entire sequence but is not found in *Antheraea* and *Samia* silk fibroin sequences^31^. The most distinctive characteristics of *Antheraea* and *Samia* silk fibroins are polyalanine sequences of four or more alanine residues (A_n_, n ≤ 4), such as (X)A_12_(X), where X is often G, S, or R. These motifs are not present in *Bombyx* fibroins but are abundant in *Antheraea* and *Samia* fibroins.

Proteomic analysis presents clear opportunities for the accurate speciation of silk fibroins, but current limitations in silk sample solubilisation and processing prevent the successful application of most techniques. To overcome this, we developed and optimised a workflow for the solubilisation and identification of various silk fibroins using nano-flow liquid chromatography tandem mass spectrometry (nanoLC-MS/MS). Our first objective was to explore the solubilisation of domesticated and wild silks. We examined the solubilisation behaviour of seven silk species commonly used in textile production historically and contemporaneously: *Bombyx* *mori* (domesticated mulberry silk, or Chinese silk)*,* *B.* *mandarina* (wild precursor of *B.* *mori*), *Antheraea* *pernyi* (Chinese tasar)*,* *Antheraea* *mylitta* (Indian tasar)*,* *Antheraea* *yamamai* (Japanese tasar, or Tensan)*,* *Antheraea* *Assamensis* (Indian Muga), and *Samia* *ricini* (Eri) (Fig. 1). Our second objective was to understand features of known fibroin reference sequences and their optimal protease digestion profiles. We aligned reference sequences to identify motifs unique to each silk species, after which we performed in silico protease digestion experiments with trypsin, chymotrypsin, and chymotrypisin-trypsin to optimize nanoLC-MS/MS protein coverage conditions. Our third objective was to develop an analytical protocol for peptide sequence analysis of digested silk solutions using nanoLC-MS/MS, allowing us to identify the composition and differentiating characteristics of modern silk samples. Finally, we applied our newly developed protocol to species identification of archaeological silks. We analysed five different samples of fibres from three alleged wild silk textiles discovered in the ancient site of Palmyra (Schimidt-Colinet/Stauffer Katalog Nr. 305, 312, and 313)^42^.

Palmyra is an ancient oasis city in the present-day Homs province of Syria, northeast of Damascus. It was established sometime around the third millennium BCE and became an important trading post during Roman imperial times, connecting the Far East, Central Asia, and the Roman Empire on the Silk Road^42–44^. Between 1930 and 1990, expeditions by French, Syrian, Polish, and German archaeologists recovered valuable artifacts from the tower tombs of Palmyra^42,45–47^. So far, more than 2000 textile fragments, of over 500 different types, have been found in the 9 different tower tombs, comprising one of the largest groups of antique textiles with a proven origin^42^. Based on information gathered from inscriptions at the tomb towers, these textiles can be dated to between the first century BCE and second century CE^43,48^. During this time period, silks were not produced in Palmyra and are therefore assumed to have been imported^48^. Stylistic analyses and microscopic examinations of some silks from the Palmyra textiles have speciated them to *Bombyx* *mori*, which was a silk known to be imported from China and woven locally in Syria^42^. Possible “tussah silks” have also been described^42,45–47^, but similar microscopic analyses have not provided sufficient information for species identification^42^. Moreover, tussah silk (also known as ‘tasar silk’) does not refer to a specific silk species and can mean a variety of silk moths of *Antheraea* species adapted to climates ranging from tropical to temperate regions in southwest China and India^49^. The textiles of Palmyra that are “possible tussah silks” therefore remain to be definitively identified as silks and, if proven to be, speciated to a specific silk-moth species. A better understanding of the origins of these textiles will therefore provide unique insights into their production and the broader economy and trade of silks in antiquity.

## Results

### Solubilisation of silk fibroins

**Figure 3:**
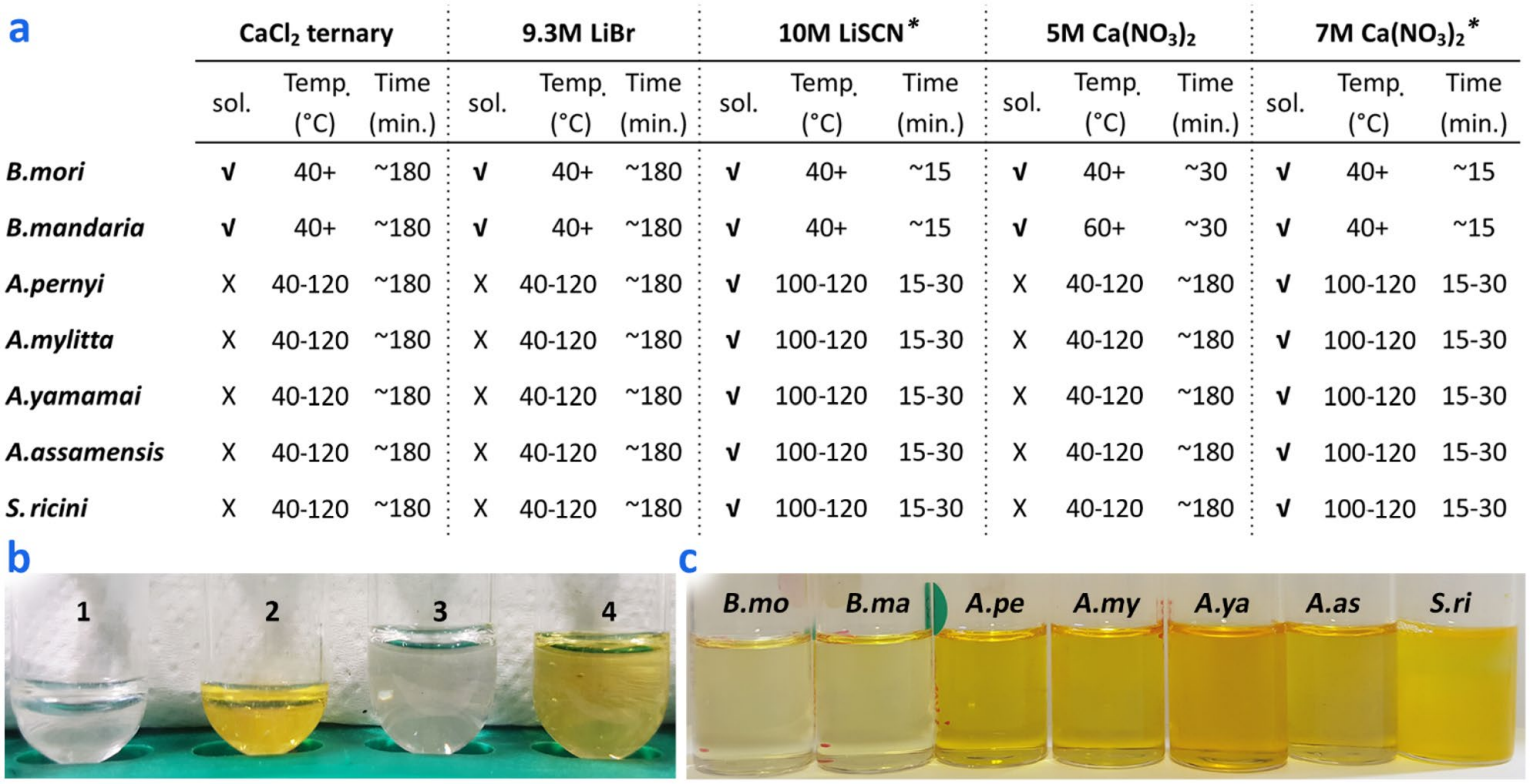
Comparison of the solubilisation capabilities of 5 different experimental salt solutions using modern silk samples derived from 7 different silk moth species. **(a)** Summary of the conditions required for solubilisation (sol.) (and whether achieved or not), including temperature (temp.) and time in minutes (min.) as determined but the disappearance into solution of the solid silk sample. *Wild silk samples showed no evidence of solubilisation at 40°, 60°, or 80 °C for ~ 180 min. **(b)** Xanthoproteic reaction of silk fibroin (*S.* *ricini*) solution by formic acid: 0.5 mg fibers in each tube with (1) 500 μl of 7 M Ca(NO_3_)_2_, (2) 500 μl 7 M Ca(NO_3_)_2_ + 0.1% FA on the weight of fabric, (3) 1 ml 7 M Ca(NO_3_)_2_, and (4) 1 ml 7 M Ca(NO_3_)_2_ + 0.1% FA. Formic acid was added after solubilisation occurred in the salt solution at 130 °C for 10 min. **(c)** 7 M Ca(NO_3_)_2_ fibroin solutions of *B.* *mori,* *B.* *mandarina,* *A.* *pernyi,* *A.* *mylitta,* *A.* *yamamai,* *A.* *assamensis*, and *S.* *ricini* after xanthoproteic reaction. Color intensity results from the presence of aromatic residues. This figure in this research was created by the first author using photographs of experimental processes edited with Adobe Photoshop CS6.

We investigated several solvent systems to identify an effective method for solubilising silk fibroins from a range of different silk genera and species, specifically *B.* *mori*, *B.* *mandarina,* *A.* *pernyi,* *A.* *mylitta,* *A.* *yamamai*, *A.* *assamensis,* and *S.* *ricini* (Fig. 1). The following five solubilising solutions were selected for further evaluation based on a literature review: (1) a ternary solution of H_2_O:ethanol:CaCl_2_ (molar ratio 8:2:1)^12,30,50^, (2) a 9.3 M LiBr solution^23,31^, (3) a 10 M LiSCN solution^31,32^, (4) a 5 M Ca(NO_3_)_2_ solution^21^, and (5) a 7 M Ca(NO_3_)_2_ solution^21,22,24,33^. Each silk sample was added to each experimental solubilising solution and heated separately to the following temperatures: 40°, 60°, 80°, 100°, and 120 °C for up to 180 min (Fig. 3a). Solubilisation was visually assessed throughout the process and then confirmed by a xanthoproteic assay. The different salt solutions resulted in different levels of protein solubility for the different species of silk fibroin analysed, and the heating temperature required for protein dissolution also varied by silk type, with higher temperatures required for *Antheraea* and *Samia* silks compared to *Bombyx* silks. Solubilisation of non-*Bombyx* fibroins was observed only in aqueous 10 M LiSCN and 7 M Ca(NO_3_)_2_ solutions when they reached approximately 105 °C, while *Bombyx* silks showed signs of solubilisation in all five solutions beginning at 40 °C. 10 M LiSCN and 7 M Ca(NO_3_)_2_ solutions solubilised all seven silk fibroins beginning at approximately 105 °C and became increasingly effective at 120 °C, reaching the most rapid solubilisation when heated to just below the boiling points of the solutions (151° and 136 °C, respectively). The xanthoproteic assay led to a colour change that indicated the presence of solubilised protein (see Fig. 3b,c). The protein solutions were also analysed by SDS-PAGE which indicated the presence of high molecular weight proteinaceous material in solution corresponding approximately to the *B.* *mori* (390 kDa) and *Antheraea* and *Samia* (~ 240 kDa) fibroins. Supplementary Fig. S2 shows the SDS-PAGE gels bands for the samples analysed. The gels also show evidence of significant protein degradation products, indicated by smearing in the lanes. This smearing was commensurate with four similar studies previously conducted on *B.* *mori,* *A.* *pernyi*, and *A.* *yamamai* silks^21,24,51^.

### Analysis of reference sequences and protease digestion profiles

Amino acid reference sequences for silk fibroins were sourced from UniProt: *B.* *mori* (P05790) and *B.* *mandarina* (Q99059) were obtained from Swiss-Prot, and *A.* *pernyi* (O75786)*,* *A.* *mylitta*(Q8ISB3)*,* *A.* *yamamai* (E1CGA3), *A.* *assamensis* (A0A0K0KR73)*,* and *S.* *ricini* (A0A0D5ZYI3) were obtained from TrEMBL. We hypothesized that the presence of any species-specific sequences of amino acids in the primary structure of each fibroin, could be used as a biomarker to differentiate silks derived from different silk moth species^18^. In order to test this hypothesis we directly aligned the amino acid sequences of fibroins from each species using Jalview (2.10.3b1) and the MUSCLE algorithm (v.3.8.31). Differences in primary structure (the sequence order of amino acids in the protein) became apparent between different genera as well as some unique sequences between different species within the same genera (Supplementary Figs. S3, S4). For example, among the many similar polyalanine blocks of *Antheraea* and *Samia* fibroins, the sequence GA_12_ is present in *A.* *mylitta,* *A.* *assamensis*, and *S.* *ricini* but not in *A.* *pernyi*, while the sequence A_15_S is present in *A.* *mylitta* *and* *A.* *yamamai* but not in *A.* *pernyi* or *A.* *assamensis*. The results of this alignment also revealed homologies of 97.77% between *Bombyx* species, between 68.94% and 77.33% among the *Antheraea* species, 41.49% between *Bombyx* and *Antheraea* species, and 60.21% between *Antheraea* and *Samia* species (Supplementary Table S2). Regardless of varying degrees of sequence homology, unique sequence motifs or even a single amino acid substitution in an area of otherwise strong homology should result in peptide sequences when digested which would be potentially capable of speciating silk fibroins based on differences in mass.

To predict an optimal protease digestion approach for nanoLC-MS/MS we ran each fibroin sequence through PeptideMass (https://web.expasy.org/peptidemass/) using trypsin, chymotrypsin, and chymotrypsin-trypsin digestion within the peptide mass range of 500–3000 Da, the optimal mass range for chromatographic separation and ionization (see Table 1, “Coverage-in silico”). Trypsin is the most commonly used protease in proteomics^28^, however, chymotrypsin has been used in previous studies when analysing the HC of *B.* *mori* fibroin due to the low numbers of the lysine (K) and arginine (R) residues that are required for trypsin cleavage (Supplementary Table S3)^12–14^. For example, in our in silico experiments, tryptic digestion resulted in the least effective sequence coverage for most of the silk fibroins. Coverage for *B.* *mori* fibroin was as low as 2%, with residues between positions 105 to 5212 being cleaved into only three very large polypeptides of 325,340 Da, 43,640 Da, and 3904 Da, all of which fall outside of standard mass detection ranges used in proteomics. In the *B.* *mori* HC, there are 12 residues of lysine, 9 of which are found within the first 104 residues, and 14 residues of arginine, 10 of which are found between residues 5206 and 5263. The light chain (LC) has a greater number of more evenly distributed lysine and arginine residues and can be analyzed effectively using trypsin (see Table 1). For *B.* *mandarina* HC fibroin, the sequence of which is currently incomplete but 98.3% identical to the first 178 residues of *B.* *mori* HC, trypsin achieved 59% sequence coverage. Similarly, trypsin resulted in 71% sequence coverage in *A.* *mylitta*, which is currently an incomplete reference sequence of only 507 residues of an estimated 2500. Chymotryptic digestion achieved 46–47% sequence coverage in *Bombyx* fibroins, 68–71% in *Antheraea* fibroins, and 96% in *S.* *ricini* fibroin. The sequential chymotrypsin-trypsin digestion achieved 48% sequence coverage in *B.* *mori* HC, 100% in B. mandarina HC, 92–100% in *Antheraea* fibroins, and 100% in *S.* *ricini* fibroin, suggesting that chymotrypsin-trypsin digestion would provide a better peptide coverage for most fibroins compared to trypsin alone.

**Table 1:** Summary of peptide analysis metrics for protein identification in each of the modern silk samples solubilised and digested with different proteases.

Sample (accession) Origin/length (signal, full)		Trypsin	FA-Trypsin	Chymotrypsin	Chymotrypsin–Trypsin
***B.*** ***mori*** **HC** **(P05790)**	Coverage (%) (in silico) **experimental**	(2) **5**	**9**	(46) **28**	(48) **19**
Domesticated silk from China	Protein confidence (−10lgP)	214.22	262.43	179.05	207.14
1–21, 22–5263	# Peptides	80	109	249	210
	# Unique	58	84	249	210
*B.* *mori* *LC* *(P12828)*	*Coverage* *(%)* *(*in silico*)* ***experimental***	*(72)* ***93***	***63***	*(83)* ***27***	*(67)* ***41***
*1–353*	*Protein* *confidence* *(−10lgP)*	*243.64*	*214.83*	*73.16*	*115.61*
	*#* *Peptides*	*192*	*88*	*16*	*28*
	*#* *Unique*	*192*	*88*	*16*	*28*
*B.* *mori* *P25* *(P04148)*	*Coverage* *(%)* *(*in silico*)* ***experimental***	*(68)* ***76***	***38***	*(92)* ***ND***	*(76)* ***6***
*1–16,* *17–220*	*Protein* *confidence* *(−10lgP)*	*144.99*	*120.26*	*ND*	*31.75*
	*#* *Peptides*	*36*	*14*	*ND*	*2*
	*#* *Unique*	*36*	*14*	*ND*	*2*
***B.*** ***mandarina*** **HC** (Q99050*)	Coverage (%) (in silico) **experimental**	(59) **0**	**2**	(47) **3**	(100) **3**
Wild silk from China	Protein confidence (−10lgP)	108.76	279.94	214.77	161.16
1–21, 22–78	# Peptides	1	19	20	8
	# Unique^†^	1	15	20	8
*B.* *mandarina* *LC* *(Q9BLL9)*	*Coverage* *(%)* *(*in silico*)* ***experimental***	*(43)* ***38***	***64***	*(69)* ***80***	*(72)* ***19***
*1–16,17–262*	*−10lgP*	*266.03*	*247.14*	*228.25*	*120.80*
	*#* *Peptides*	*12*	*26*	*45*	*4*
	*#* *Unique*	*12*	*26*	*45*	*4*
*B.* *mandarina* *P25* *(Q9BLL8)*	*Coverage* *(%)* *(*in silico*)* ***experimental***	*(83)* ***24***	***10***	*(92)* ***4***	*(75)* ***9***
*1–17,* *18–220*	*−10lgP*	*185.85*	*118.00*	*58.47*	*81.76*
	*#* *Peptides*	*6*	*2*	*1*	*2*
	*#* *Unique*	*6*	*2*	*1*	*2*
***A.*** ***pernyi*** **(O76786)**	Coverage (%) (in silico) **experimental**	(23) **13**	**21**	(71) **45**	(100) **23**
Temperate Tasar from China	−10lgP	286.14	240.07	248.59	279.64
1–18, 19–2639	# Peptides	73	83	210	129
	# Unique	15	24	102	12
***A.*** ***mylitta*** (Q8ISB3*)	Coverage (%) (in silico) **experimental**	(71) **39**	**49**	(68) **71**	(92) **56**
Tropical Tasar from India	−10lgP	278.14	176.66	220.77	371.53
1–18,19–507	# Peptides	29	45	147	115
	# Unique	9	18	76	53
***A.*** ***yamamai*** **(E1CGA3)**	Coverage (%) (in silico) **experimental**	(21) **12**	**19**	(70) **30**	(100) **18**
Japanese Oak silk from Japan	−10lgP	242.9	223.41	237.48	313.66
1–18, 19–2856	# Peptides	47	96	168	203
	# Unique	12	29	48	79
***A.*** ***assamensis*** **(A0A0K0KR73)**	Coverage (%) (in silico) **experimental**	(10) **8**	**16**	(68) **47**	(100) **51**
Muga silk from India	−10lgP	265.14	257.42	376.74	410.64
1–18,19–2809	# Peptides	66	88	157	247
	# Unique	50	60	141	201
***S.*** ***ricini*** **(A0A0D5ZYI3)**	Coverage (%) (in silico) **experimental**	(10) **9**	**21**	(96) **41**	(100) **30**
Eri silk from Thailand	−10lgP	354.91	353.58	383.70	591.87
1–18, 19–2880	# Peptides	93	150	252	278
	# Unique	91	147	249	274

Experimental coverage values are in bold.

Accession codes are in the format of UniProtKB. The false discovery rate (FDR) for peptide probability is ≤ 1%.

*N/A* not available, *ND* not detected.

The light chain (LC) and P25 proteins of *Bombyx* fibroins are displayed in italics font.

*Reference sequences for *B.* *mandarina* HC and *A.* *mylitta* HC are incomplete.

^†^All *B.* *mandarina* HC peptides only matched to the *B.* *mori* reference sequence.

### Identifying silk fibroins using nanoLC-MS/MS

**Figure 4:**
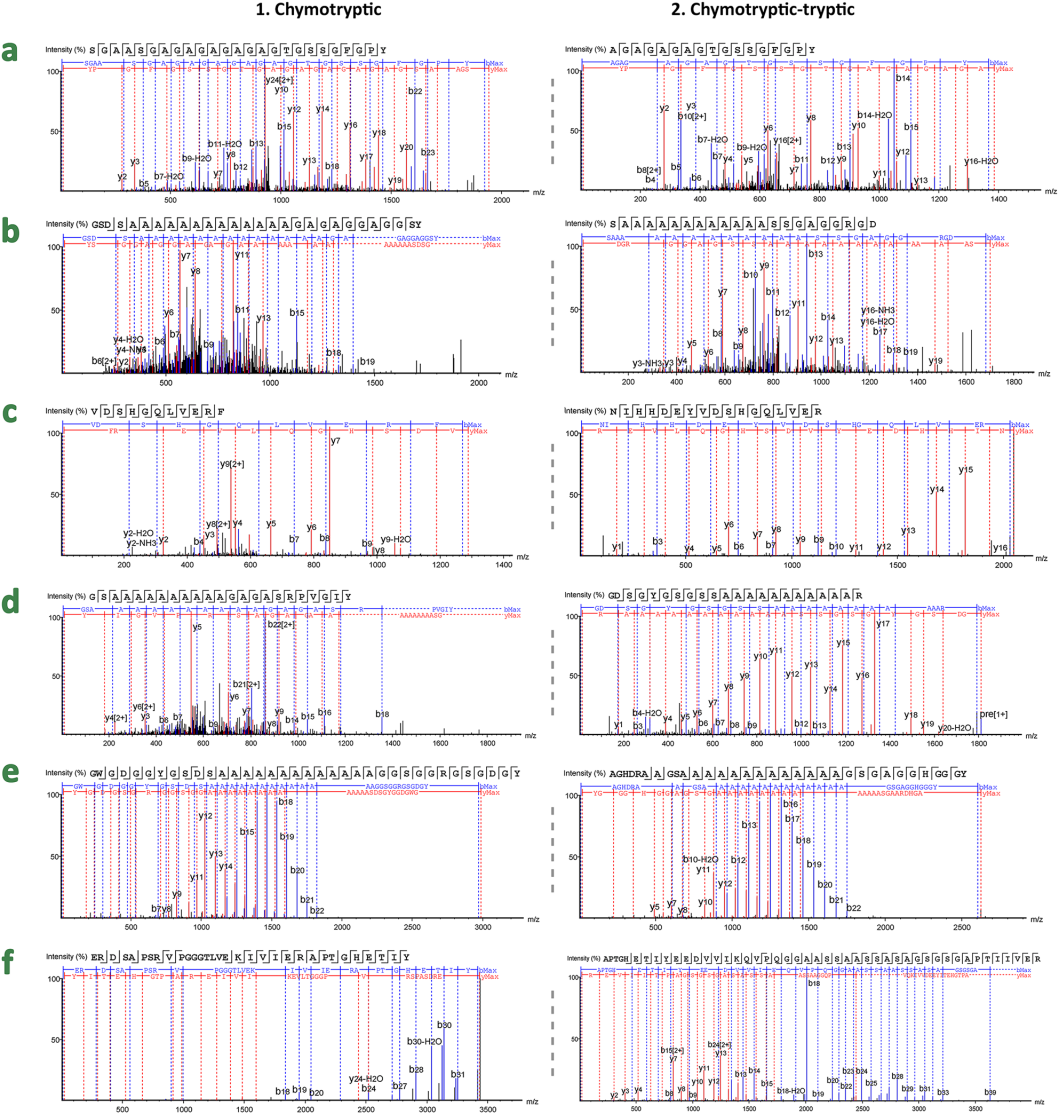
MS/MS spectra from the chymotryptic (column 1) and chymotryptic-tryptic (column 2) unique peptides with the highest probability scores from each sample. Sequences marked in red indicate species-specific sequences, and those marked in blue indicate genera-specific sequences. **(a)** *B.* *mori*: (1) SGAASGAGAGAGAGAGTGSSGFGPY (*m/z* = 899.90, z = 2, −10lgP = 37.21); (2) AGAGAGAGTGSSGFGPY (*m/z* = 692.81, z = 2, −10lgP = 42.48); **(b)** *A.* *pernyi*: (1) GSDSA_13_GAGAGGAGGSY (*m/z* = 698.65, z = 3, −10lgP = 49.23); (2) SA_12_SSGAGGRGD (*m/z* = 851.91, z = 2, −10lgP = 43.94); **(c)** *A.* *mylitta*: (1) VDSHGQLVERF (*m/z* = 643.83, z = 2, −10lgP = 44.27); (2) NIHHDEYVDSHGQLVER (*m/z* = 683.32, z = 3, −10lgP = 88.20); **(d)** *A.* *yamamai*: (1) GSA_10_GAGASRPVGIY (*m/z* = 634.67, z = 3, −10lgP = 44.15); (2) GDSGYGSGSSA_11_R (*m/z* = 905.92, z = 2, −10lgP = 73.57); **(e)** *A.* *assamensis*: (1) GWGDGGYGSDSA_13_GGSGGRGSGDGY (*m/z* = 996.77, z = 3, −10lgP = 80.20); (2) AGHDRAAGSA_13_GSGAGGHGGGY (*m/z* = 874.75, z = 3, −10lgP = 88.81); **(f)** *S.* *ricini*: (1) ERDSAPSRVPGGGTLVEKIVIERAPTGHETIY (*m/z* = 859.46, z = 2, −10lgP = 72.93); (2) APTGHETIYEEDVVIKQVPQGGAASSAASSASAGSGSGAPTIIVER (*m/z* = 1114.06, z = 4, −10lgP = 200). This figure was created by the first author using experimental data from PEAKS 7.5 edited with Adobe Photoshop CS6.

To determine whether the *in-silico* predictions could be practically applied to the differentiation of silk fibroins by species, we started by solubilising fibroins using 7 M Ca(NO_3_)_2_ and performing a range of different proteolytic digestions using (1) trypsin, (2) formic acid followed by trypsin, (3) chymotrypsin, and (4) chymotrypsin followed by trypsin. 7 M Ca(NO_3_)_2_ was selected (over the 10 M LiSCN) as the 7 M Ca(NO_3_)_2_ system did not affect cashmere wool, linen, or cotton fibers in boiling tests (125 °C, 30 min)^49^. Formic acid treatment before trypsin digestion was used to induce aspartic acid (D) cleavages and to prevent protein reaggregation during sample preparation as formic acid has been reported to effectively prevent solubilised silk fibroin molecules from forming micelles, which would assemble into micro-sized globules due to hydrophobic interactions and shielded negative charges^52,53^. All seven species of silk fibroin were digested under each condition, yielding a total of 28 different experimental samples for analysis by nanoLC-MS/MS which was performed next. Analysis of the results was performed using PEAKS Studio 8.5 (Bioinformatics Solutions Inc., Canada) and a summary of subsequent protein identifications is presented in Table 1 (see Supplementary Figs. S5–28 for protein coverage maps). *B.* *mori*, *A.* *pernyi,* *A.* *mylitta,* *A.* *yamamai,* *A.* *assamensis*, and *S.* *ricini* silk samples were successfully identified to species level regardless of the type of protease used. Unique species-specific peptides^18^ were detected in each sample (Table 1, “#Unique”), with some samples resulting in up to 100% of the detected peptides being unique to a particular species of silk fibroin in the context of these seven species (see Supplementary Tables S4–9). Characteristic sequence features for each species were observed, including *B.* *mori*-specific hexapeptides GAGAGA and GAGAGT (Fig. 4a), *Antheraea* genus-specific peptide SGAGG (Fig. 4c) and polyalanine blocks (Fig. 4b,d,e), and *S.* *ricini*-specific peptide GGGYGGDGG (Fig. 4f). Nonspecific cleavages after alanine, glycine, serine, and aspartic acid residues were occasionally observed in all samples other than the trypsin-only digests (Supplementary Fig. S29), though their occurrence was minimal and did not impede unequivocal protein identification to the species level. The differentiation of *B.* *mandarina* from *B.* *mori* was more challenging due to the incomplete 178 residue reference sequence of *B.* *mandarina* HC and the high sequence homology between known *B.* *mori* and theoretical *B.* *mandarina* sequences (99.6% for LC, 99.1% for P25, and 98.3% for HC).

Overall, the highest protein coverages were achieved using chymotrypsin digestion and not chymotrypsin-trypsin digestion as predicted by *in-silico* experiments. The largest numbers of unique peptides were identified with chymotrypsin digestion of *Bombyx* fibroins (HC only), *A.* *pernyi*, and *A.* *mylitta*, but with chymotrypsin-trypsin digestion for *A.* *yamamai*, *A.* *assamensis*, and *S.* *ricini*. The quality of the peptide-spectrum match, represented as a protein probability score (−10lgP), was higher in chymotrypsin-trypsin digests compared to chymotrypsin-only digests for all species except *B.* *mandarina* HC. Protein coverages observed during experimentation were lower than those predicted by in silico analysis, except in the chymotrypsin digest of *A.* *mylitta* and the trypsin digest of *B.* *mori* HC. The difference seen in *A.* *mylitta* was 3%, which was insignificant given the 507 residues reference sequence. The difference in *B.* *mori* HC, however, was significant since the trypsin-only digest (5% coverage) and FA-trypsin digest (9% coverage) where much higher than the *in-silico* trypsin digest (2% coverage) for the 5263 residues reference sequence. This higher experimental coverage appears to be largely due to the degradation of fibroin to larger polypeptides during solubilisation. While in silico analysis predicted no peptides within the 500–3000 Da range from the residues 105–5212, experimental trypsin digestion yielded detectable peptides from residues 105–132, 661–689, 1639–1650, and 5120–5150. For the FA-trypsin digestion, peptides from residues 105–118, 421–448, 492–522, 652–672, 1071–1096, 1249–1263, 1307–1327, 1491–1517, 2531–2582, 3365–3386, 3883–3907, 4455–4482, and 4687–4705 were observed, with most peptides showing the expected formylation. In all fibroin samples, formic acid-trypsin digestion showed largely improved digestion compared to trypsin-only, though samples also showed various post-translational modifications including formylation, acetylation, carbamylation, and dehydration (Supplementary Table 10).

### Species identification of Palmyra samples

**Figure 5:**
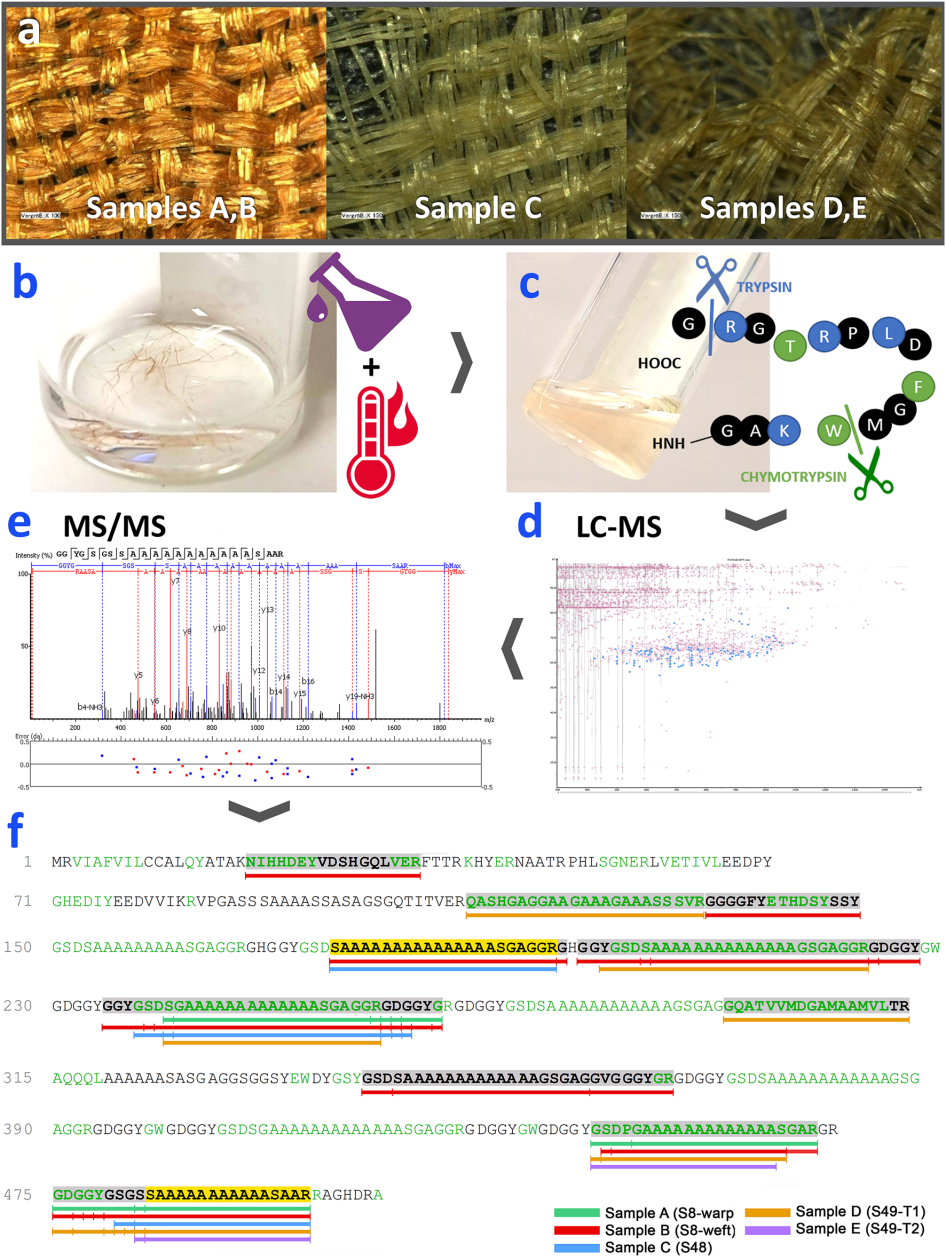
Workflow for sample preparation, sample analysis, data processing and species interpretation of Palmyra silks samples. **(a)** *Sample* *for* *analysis–*images of the Palmyra textiles: sample S8(left), Schimidt-Colinet/Stauffer Katalog Nr. 305 (magnification × 100); sample S48 (center), Schimidt-Colinet/Stauffer Katalog Nr. 312 (magnification × 150); sample S49(right), Schimidt-Colinet/Stauffer Katalog Nr. 313 (magnification × 150). **(b)** Fibers from S8 (weft) placed in a 7 M Ca(NO_3_)_2_ solution at 80˚C. **(c)** Solubilisation of fibers from S8 (weft) solubilised at 125 ˚C and digested with chymotrypsin-trypsin. **(d)** 2-D LC–MS *m/z*-retention time plot for peptides analysed (highest scoring peptide GGYGSGSSA11SAAR (−10lgP = 70.75), which is a peptide unique to Q8ISB3). Sample B had the highest −10lgP value and the largest number of peptides unique to Q8ISB3 among the samples. **(e)** MS/MS spectrum for (D). **(f)** The incomplete sequence map of *A.* *mylitta* fibroin (Q8ISB3, 507 residues) with the marked bars indicating unique peptides of Q8ISB3; the alternating black and green residues indicate theoretical peptides cleaved by chymotrypsin-trypsin digestion; the bold residues indicate the detected peptides; and the vertical bars mark different cleavage sites observed in detected peptides. This figure in this research was created by the first author using photographs of samples and experimental processes, and original graphics edited with Adobe Photoshop CS6.

Five fibre samples were collected from three alleged silk textiles found at the archaeological site of ancient Palmyra (Fig. 5a). Textile S8 was made of very shiny, flat fibres, and only the weft had slight torsion in the Z direction based on previous microscopic analysis. Textiles S48 and S49 had similar looking flat fibres for both warp and weft. It was speculated that all three textiles were made of silk that was not derived from *B.* *mori*, but confirmatory investigations could not be conducted until now due to the lack of a suitable analytical technique^42^. To determine if the textile samples were made of silk and, if so, to identify the fibroin species used, we analysed the samples using our optimized 7 M Ca(NO_3_)_2_ solubilisation and nanoLC-MS/MS protocol described above. Solubilisation was not observed from 40 to 80 °C (Fig. 3a) but did take place between 100 and 125 °C (Fig. 5b,c), as previously observed in experiments with modern wild silk species standards. Each solubilised fibre sample was digested using chymotrypsin-trypsin since our previous experiments demonstrated that chymotrypsin-trypsin produced the highest protein confidence score and largest number of unique peptides in most wild silks (Fig. 5d,e). A summary of the resulting −10lgP values, coverages, and unique peptides identified by a combined analysis of direct database matching with post transitional modification searches (PEAKS PTM) and homology matching of de novo tags (PEAKS SPIDER) is provided in Table 2. The extended searches increased the number of unique peptides detected in the fresh samples (Supplementary Tables 5–10). Due to the incomplete reference sequence of *A.* *mylitta* fibroin, which is over 75% shorter than the fibroin sequences of other *Antheraea* species, we performed a PEAKS SPIDER search to map de novo tags to areas of high homology in known reference sequences.

**Table 2:** Summary of the de novo homology matching performed on the results from the proteomic analysis of the Palmyra samples. Accession codes are in the format of UniProtKB.

Sample	Highest scoring peptide	Accession	−10lgP	Cov (%)	#Peptide (unique)	Unique peptides (position, −10lgP)
**A** S8 warp	SA_12_GSGAGGRGD (−10lgP = 47.33) *Antheraea* *specific*	Q8ISB3|ANTMY	163.41	30	31 (12)	SGA_12_SGAGGR (243–263, 46.77) SSA_11_SAAR (484–500, 44.12)
		E1CGA3|ANTYA	160.30	7	35(7)	
		O76786|ANTPE	151.28	7	31(3)	
		A0A0K0KR73|ANTASA	121.62	8	18(7)	
**B** S8 weft	GGYGSGSSA_11_SAAR (−10lgP = 53.66) *A.* *mylitta* *specific*	Q8ISB3|ANTMY	238.35	48	48 (29)	GGYGSGSSA_11_SAAR (478–500, 70.75) SA_14_GSGAGGRGDGGY (206–232, 41.95) GA_12_SGAGGR (244–263, 40.40) GGGGFYETHDSYSSY (134–148, 37.28) GSDSA_13_GSGAGGVGGGYGR (346–375, 31.11) SA_15_SGAGGR (176–197, 27.45) NIHHDEYVDSHGQLVER (20–36, 21.13)
		E1CGA3|ANTYA	197.23	11	44 (14)	
		O76786|ANTPE	195.64	10	36 (6)	
		A0A0K0KR73|ANTASA	156.27	8	16 (8)	
**C** S48	GAGSA_10_GAGASR (−10lgP = 49.64) *A.* *pernyi* *specific*	O76786|ANTPE	131.54	8	12 (5)	
		1CGA3|ANTYA	123.78	8	14(6)	
		Q8ISB3|ANTMY	115.17	22	12(7)	SA_15_SGAGGR (176–197, 39.04) SGA_12_SGAGGRGD (243–265, 37.66) SGSSA_11_SAAR (482–500, 36.05)
**D** S49 T1	GYGSGSSA_10_SAAR (−10lgP = 55.54) *A.* *pernyi* *specific*	Q8ISB3|ANTMY	179.20	36	17 (13)	GSGSSA_11_SAAR (481–500, 64.48) QASHGAGGAAGAAAGAAASSSVR (111–133, 44.75) SGA_12_SGAGGR (243–263, 25.96) GQATVVMDGAMAAMVLTR (297–314, 20.53)
		E1CGA3|ANTYA	172.99	5	16(4)	
		O76786|ANTPE	170.24	6	18(5)	
**E** S49 T2	SA_11_SAAR (−10lgP = 32.85) *A.* *mylitta* *specific*	E1CGA3|ANTYA	116.35	7	25(8)	
		A0A0K0KR73|ANTASA	105.68	5	19(2)	
		Q8ISB3|ANTMY	103.05	24	15(3)	SA_11_SAAR (485–500, 32.35) GSDPGA_13_ (452–469, 24.41)

The −10lgP score indicates the statistical significance of the peptide-spectrum match. Peptides were filtered by −10lgP values ≥ 20, and the values ≥ 70 is usually considered significant for proteins.

The unique peptides of *Antheraea* silk fibroins, SGAGG and A_n≥8_, that were previously identified in the modern silk samples were found in all Palmyra samples (Table 2 and Supplementary Tables S11–15). The combined database and homology search assigned samples A, B, and D to originate from *A.* *mylitta* fibroins (Fig. 5f). Results from sample B yielded the highest protein probability score (−10lgP = 238.35), with 48% sequence coverage and 29 unique peptides exclusive to *A.* *mylitta* fibroin. Samples C and E matched to other *Antheraea* fibroins by slightly higher probability scores, but the presence of GGYGSGSSA_11_SAAR (478–500) and SA_15_SGAGGR (176–197) in sample C, and SA_11_SAAR (485–500) in sample E suggested that the fibroins belonged to *A.* *mylitta* (Table 2). The unique motifs A_15_S and SSA_11_S present in these peptides are exclusive to *A.* *mylitta* (Q8ISB3), so their detection in unidentified samples is important for distinguishing *A.* *mylitta* from other species. A_15_S was detected in samples B and C, and SSA_11_S was detected in all five samples. The motif GA_12_ (244–257), which is specific to *A.* *mylitta*, *A.* *assamensis*, and *S.* *ricini* but not *A.* *pernyi*, was detected in samples A, B, C, and D. Sample C matched to *A.* *pernyi* fibroin (O76786) followed by *A.* *yamamai* (E1CGA3) and *A.* *mylitta* (Q8ISB3), while sample E matched to *A.* *yamamai* fibroin (E1CGA3) followed by *A.* *assamensis* (A0A0K0KR73) and *A.* *mylitta* (Q8ISB3). Interestingly, the highest −10lgP value peptides in samples C and E were not exclusive to one species: GAGSA_10_GAGASR from sample C is common to *A.* *pernyi* and *A.* *yamamai* fibroins, while SA_13_GSGA from sample E is common to *A.* *pernyi*, *A.* *mylitta*, *A.* *yamamai*, and *A.* *assamensis*. Sample C also contained the peptides A_15_SGAGGR (176–197, −10lgP = 39.04), SGA_12_SGAGGRGD (243–265, −10lgP = 37.66), and SGSSA_11_SAAR (482–500, −10lgP = 36.05). Sample E contained the peptide SA_11_SAAR (485–500, −10lgP = 32.35). All of these are unique to *A.* *mylitta*.

## Discussion

Our successful solubilisation of silk fibroins was dependent on heating temperature, salt type and salt concentration and the optimal combination of conditions was show to be different for the different silk-producing species. When heated over 100˚C, both 10 M LiSCN and 7 M Ca(NO_3_)_2_ solutions were able to fully solubilise all seven species of domesticated and wild silk fibroins. We selected 7 M Ca(NO_3_)_2_ for the solubilisation of silk fibroin from archaeological samples. The different solubilising behaviour of *Bombyx*, *Antheraea*, and *Samia* silk fibroins may be due to varying biochemical properties that result from amino acid sequence variation and post-translational modification of residues in the fibroins themselves. Biophysical variation may be linked to the fibroin secretion process, where a structural transition from liquid Silk I to solid Silk II typically occurs. This transition in *Antheraea* and *Samia* silkworms is known to produce a strongly exothermic phase transition from an α*-*helical conformation to a β-form, whereas the same transition process in *B.* *mori* is only weakly exothermic^54^. This suggests that the dissolution of *Antheraea* and *Samia* silks may be more dependent on heating temperature than *Bombyx* silks; a prediction which is commensurate with our findings.

We performed in silico analyses to guide protease selection for nanoLC-MS/MS experiments. In silico experiments predicted that chymotrypsin-trypsin digestion would yield the highest protein coverage across all fibroins, but experimental nanoLC-MS/MS results showed that chymotrypsin alone provided higher coverage. It is hypothesised this was due to the solubilisation process initially denaturing and degrading some of the fibroins, which would result in some smaller peptides after digestion than predicted, as was indicated by the SDS-PAGE results (Supplementary Fig. S2). FA-trypsin digestion increased the number of peptides detected for each fibroin and increased sequence coverage compared to trypsin alone, but it was less effective than chymotrypsin and resulted in a higher occurrence of post transitional modifications such as formylation. Chymotrypsin remained a better choice for digesting the highly hydrophobic fibroins, particularly since the lysine and arginine residues necessary for trypsin cleavage are uncommon in silk fibroins (see Supplementary Table S3, GRAVY value)^55^. Our results suggest that pre-treatment with weak organic acids before chymotrypsin digestion may be worth investigating further. Despite evidence that partial degradation of fibroins occurred as a result of the solubilisation process, identification of unique peptides by nanoLC-MS/MS led to successful differentiation of silk species and identification. Our method was able to differentiate six of the seven silk species analysed. *B.* *mandarina* and *B.* *mori* were difficult to differentiate due to the limited protein sequence information on publicly available databases. Further work is needed to make a complete sequence for *B.* *mandarina* available in public databases as well as extend the range of silk moth species. The use of PEAKS SPIDER for the analysis of modern silk sample data led to a small number of additional unique peptides being identified. PEAKS SPIDER was then applied to the analysis of the archaeological samples from Palmyra, but no additional peptides were identified, presumably due to their heavily degraded state.

**Figure 6:**
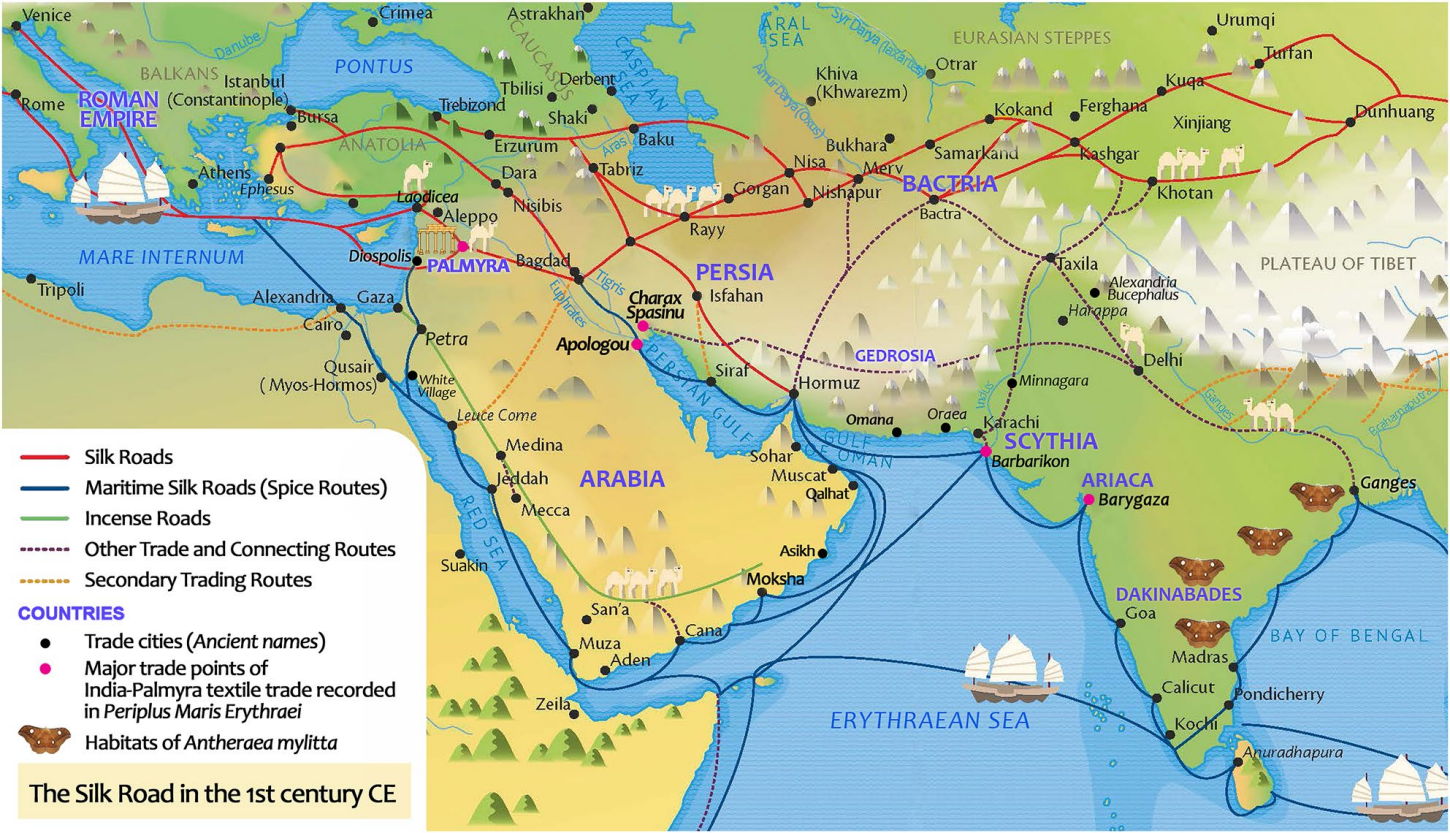
A representation of the Silk Road in the first century CE (adapted from the Silk Road Interactive Map, http://unescosilkroad8.dev2.agiledrop.com/silkroad-interactive-map) and the suggested textile trade routes from India to Palmyra. In *Periplus* *Maris* *Erythraei*, silk and cotton were exported to Palmyra from the ports of Barygaza and Barbarikon^43,60,61^.

Five textile fragments recovered from the archaeological site of Palmyra were analysed using our fibroin solubilisation method followed by nanoLC-MS/MS analysis. The results provide direct evidence that these textiles were produced from wild silks derived from *A.* *mylitta*. This species of silk moth, known as ‘tropical tasar’ or ‘Indian oak silkmoth,’ is native to India and is a member of the *Saturniidae* family. Tasar, also referred to as tassar, tusser, tussar, or tussah, is derived from the Sanskrit word *trasara* (त्रसर), meaning shuttle. Tasar silk in India is mentioned as early as 1590 BCE in the *Ramayana*, where Rama sends tasar silk to Sita as a nuptial gift^56^. Historical records suggest that Chinese silk was imported from India during the Mauryan period (322–183 BCE) or early Han era (202 BCE–202CE), but the species involved have not been explicitly identified to-date^57^. Records refer to ‘Chinese silk’ and ‘indigenous silk’ using separate terms, implying a need to distinguish them and potentially recognizing two separate sericulture practices. In *Arthashastra* of Kautilya (*c.* 5–4th BCE), *patrorna*, *kauseya*, and *cinapatta* silks are listed in the king’s treasury. *Patrorna* is known to be a type of wild silk, spun from cocoons collected from various trees; *kauseya* is the silk made from locally cultivated silkworms mentioned as *koseyya* in the *Ashtadhyayi* of Panini (*c.* 6–4th centuries BCE)^4^; and *cinapatta* is Chinese silk. In the Hindu epic *Mahabharat* (*c*. fifth century BCE), the silkworms brought from China were called “*Patta-keetas*”^58^, and the *Amarakosha* (*c.* fourth century CE) mentioned silk called “*Cheenam* *Sokam*”, meaning the “cloth of China”^57^. However, direct archaeological evidence for silks in South Asian prehistory is limited to a single find of alleged wild silk from Nevasa in central India dating to *c.* 1500–1050 BCE^59^. Archaeologists have speculated that Indian merchandise was imported into Palmyra based on the trade routes recorded in *Periplus* *Maris* *Erythraei* written around the first century CE. The text describes a trade route connecting the Indian port of Barygaza or Barbarikon of Cythia to Apologou and neighbouring Charax Spasinu by the Persian Gulf sea lane (Fig. 6)^43,60,61^. Both Barygaza and Barbarikon exported cotton, silk cloth, and raw silk to Apologou^62^, though these perishable goods did not leave much evidence in the archaeological record^61^. Additionally, no items have ever been identified as Indian silk. By identifying and provenancing the wild silks discovered at ancient Palmyra as *A.* *mylitta* of Indian origin, we provide the first biochemical evidence to support long-standing archaeological speculation surrounding the production of Indian wild silks in antiquity and international trade between ancient Palmyra and the Indian subcontinent.

In summary, the research presented involved the development, testing and validation of a novel method for silk fibroin solubilisation and species identification by nanoLC-MS/MS. This method overcomes limitations of some conventional silk identification techniques by providing direct molecular evidence of fibroin composition including previously unattainable species-level information from wild silk fibroins. The method was successfully applied to the analysis of seven modern silk species and the subsequent analysis of archaeological silk samples from ancient Palmyra. The latter provides the first direct evidence of silk production and export using wild silk moths from India. While the solubilisation and processing of silk fibroin samples, followed by nanoLC-MS/MS analysis, was effective for species identification, the greatest limitation was the incomplete state of publicly available silk protein reference sequences, particularly for *B.* *mandarina* and *A.* *mylitta*. Expanding protein databases with respect to silk moth fibroins would extend the scope and application of our method and improve successful outcomes for the analysis of significantly degraded or contaminated archaeological silk samples. Nevertheless, the ability to solubilise previously challenging wild silk fibroins provides new opportunities for archaeological applications as demonstrated in this study. It also provides a tool for the study of engineered liquid silks that have the potential for new practical applications in materials science.

## Methods

### Preparation of silk fibroins

Silk fibres were freshly prepared from the cocoons. Silk cocoons were sourced from various places: *B.* *mori,* *B.* *mandarina*, and *A.* *pernyi* cocoons was collected from China. *A.* *mylitta,* *A.* *assamensis* cocoons were collected from India, *A.* *yamamai* cocoon was collected from Japan, and *S.* *ricini* was collected from Thailand (Table 1). *B.* *mori* cocoon produced in 2012 was collected by Dr. Fritz Vollarath (Oxford Silk Group) and the rest of the cocoons were collected by the late Dr. Irene Good (Oxford RLAHA) and given to BL in November 2014. This section describes the process to remove sericin (degumming) from the silk moth cocoon. For *Bombyx* silks, cut cocoon pieces were treated for one hour in a 95 °C water bath containing a degumming solution prepared with 0.5% Marseille soap (Marius Fabre) and 0.3% sodium bicarbonate in Milli-Q water. The volume of degumming solution used was 50 × the weight of the fibres. For *Antheraea* and *Samia* silks, cut cocoon pieces were treated for 30 min in a 95 °C water bath containing a degumming solution prepared with 0.1% sodium carbonate and 0.1% sodium hydrosulphite. The volume of degumming solution used was 40 × the weight of the fibres. The treated *Antheraea* and *Samia* fibres were then further degummed for one hour in a 55–60 °C water bath under constant agitation containing a solution of 0.1% Alcalase^®^ 2.4 (*Bacillus* *licheniformis*), 0.5% sodium bicarbonate, and 0.1% Marseille soap in Milli-Q water. The volume of solution used was 50 × the weight of the pre-treated fibres. The treated fibres were then rinsed with a 0.2% Marseille soap solution. As a final step, all degummed fibres were rinsed thoroughly with a 0.2% sodium dithionite solution to remove excess soap and alkali and then rinsed five times with lukewarm Milli-Q water. The collected fibres were dried and conditioned for 48 h according to ISO 139:2005 standard prior to testing.

### Solubilisation of fibroins

*Modern* *silk* *fibres* were weighted to 0.5 mg per sample and treated in one volume (1 mg/ml) of 7 M Ca(NO_3_)_2_ solution or 10 M LiSCN. Samples were treated at five different temperatures (40, 60, 80, 100, and 120ºC) and their change over time (from 10 min to 6 h) were observed. At above 100 ºC, small volume of MilliQ water was added to maintain the liquid volume as the water in the solution evaporates, and the treatment was stopped when no more solid mass was observed.

*For* *archaeological* *samples*, fibres were first treated in 10 volumes of methanol at 60–65 ºC for 10 min with gentle shaking to extract dyes and organic contaminants. Methanol was removed (this portion can be stored for dye analysis) and the samples were gradually heated in 7 M Ca(NO_3_)_2_ solution from 80 to 125–130 ºC. The samples did not show any sign of solubilisation at 80 ºC in the first 5 min, thus the temperature was raised to 125–130 ºC and treated for 10 min. Resulted fibroin solutions were diluted with MilliQ water to 1 ml and centrifuged for 10 min at 13,000 rpm to precipitate any impurities.

### Xanthoproteic assay

This a chemical assay used to test for the presence of protein in solubilised form. It involves the nitration of the phenyl group (–C_6_H_5_) in aromatic amino acids, tryptophan (W) and tyrosine (T), which forms yellow nitro-substitution products. The reaction was induced by adding formic acid (0.1% by volume) to the 7 M Ca(NO_3_)^2^ fibroin solution. The presence of protein in solution is indicated by the solution turning yellow or orange.

#### SDS-PAGE

25 µl of desalted and concentrated fibroin solution was mixed with a same volume of sample buffer (Laemmli × 2/Sigma S3401-1VL) and heated for 5 min at 95 °C for denaturation. Samples were briefly centrifuged, and the supernatants were loaded to each well of a precast gel (Biorad Criterion XT Tris–Acetate Gels 3–8%, 45 µl well). 15 µl of Protein standard marker (1:1 mixture of Biorad Precision Plus Protein™ Dual Colour Standards, 10–250 kDa) and Sigma HiMark™ Pre-Stained Protein Standard, 31–460 kDa) was loaded at the very end of the gel plate. The gel plate was run for 65 min at 150 V in the running buffer (1 M Tris, 1 M Tricine, 1% SDS, pH 8.3). Once removed from the cast, gels were washed with milliQ water and stained with Coomassie dye for an hour. The staining solution was removed, and the gels were detained overnight. Staining process for each type of stains were followed by the product manual (Thermo Coomassie R-250, Sigma ProteoSilver™ Silver Stain Kit).

### Desalting and digestion

Fibroin solutions were mixed with one volume of 100 mM tris(2-carboxyethyl) phosphine (TCEP) in 8 M urea buffer and reduced for an hour at room temperature, then alkylated in 200 mM Iodoacetamide (IAA) for 45 min in dark. The fibroin solution was spun in ultra-centrifugal filter unit (AMICON, NMWL 3K) for 30 min. When 7 M calcium nitrate solution was used as solvent, the fibroin solution was further diluted with MilliQ water to 5–10 volumes to facilitate the buffer exchange process. The reduced volume was filled up with 6 M urea buffer and spun for 30 min; this process was repeated until the total retentate volume was reduced to the initial fibroin solution volume. Then urea was removed by two washes with 50 mM ammonium carbonate (ABC), and further concentrated if necessary. The concentrated fibroin solution was digested using trypsin or chymotrypsin (1:50) in 50 mM ABC for 18–20 h at 37 ºC. Then, acetonitrile (ACN) was added to the filter unit up to 80% of volume with enzyme (1:100) and further digested for 4 more hours. For FA (5%) treatment before trypsin digestion, a portion of fibroin solution was taken to a separate tube, mixed with formic acid to 5% of volume, and set for (4) hour before trypsin digestion. In case of chymotrypsin-trypsin digestion, the sample was first digested with chymotrypsin, then trypsin was added with ACN and further digested for 4–6 h. Digestion process was stopped by adding formic acid to 5% of volume. The resulted peptides were vacuum dried, eluted with 0.1% formic acid (FA) for the peptide cleaning using 100 µl size ZipTip (C18, Thermo)using Buffer A (98% Milli-Q water, 2% ACN, 0.1% FA) and Buffer B (80% ACN, 20% Milli-Q water, 0.1% FA). Extracted peptides were vacuum dried and resuspended in elution buffer (0.05% TFA, 2% ACN in H_2_O) for LC–MS analysis.

### In silico digestion

In-silico digestion was performed using PeptideMass (https://web.expasy.org/peptidemass/) and trypsin (higher specificity), chymotrypsin (C-term to F/Y/W, not before P), and chymotrypsin-trypsin (C-term to K/R/F/Y/W, not before P) environments, with up to 2 missed cleavages being allowed. The signal peptides of each protein sequence were automatically removed by the algorithm. For each peptide display, monoisotopic masses of the occurring amino acid residues were used, with peptide masses being shown as [M + H] + . All cysteine residues were reduced with iodoacetamide, and methionine residues were not oxidized. For the sequence coverage calculation, the number of peptides within a mass range of 500–3000 Da were counted.

### NanoLC-MS/MS data acquisition

The peptides were analysed on a nanoAcquity-UPLC system (Waters) connected to an Orbitrap Elite mass spectrometer (Thermo Fischer Scientific) possessing an EASY-Spray nano-electrospray ion source (Thermo Fischer Scientific). The peptides were trapped on an in-house packed guard column (75 μm i.d. × 20 mm, Acclaim PepMap C18, 3 μm, 100 Å) using solvent A (0.1% Formic Acid in water) at a pressure of 140 bar. The peptides were separated on an EASY-spray Acclaim PepMap^®^ analytical column (75 μm i.d. × 50 mm, RSLC C18, 3 μm, 100 Å) using a linear gradient (length: 100 min, 3% to 60% solvent B (0.1% formic acid in acetonitrile), flow rate: 300 nL/min). The separated peptides were electro-sprayed directly into the mass spectrometer operating in a data-dependent mode using a CID based method. Full scan MS spectra (scan range 350–1500 *m/z*, resolution 120,000, AGC target 1e6, maximum injection time 250 ms) and subsequent CID MS/MS spectra (AGC target 5e4, maximum injection time 100 ms) of 10 most intense peaks were acquired in the Ion Trap. CID fragmentation was performed at 35% of normalized collision energy and the signal intensity threshold was kept at 500 counts. The CID method used performs beam-type CID fragmentation of the peptides.

Due to the moving of the first author, the following seven samples were processed with different instruments at Smithsonian Museum Conservation Institute: chymotrypsin-trypsin digestion of *A.* *pernyi*, *A.* *mylitta*, and *A.* *yamamai*; both chymotrypsin and chymotrypsin-trypsin digestion of *A.* *assamensis* and *S.* *ricini*. The peptides were analysed by nanoLC-MS/MS: The peptides were first loaded onto an in-house packed Thermo BioBasic C_18_ precolumn (30 mm × 75 µm i.d.) after which they were separated on an in-house packed analytical column (210 mm × 75 µm i.d.) made of the same stationary phase, using a Thermo Scientific Dionex UltiMate 3000 nanoLC system with the following gradient: 2% B 0–8 min, 55% B 98 min, 90% B 100–103 min, 2% B 104–120 min, where buffer A is 0.1% FA in H_2_O and buffer B is 0.1% FA in acetonitrile (ACN). The nanoLC system was directly coupled to a Thermo Scientific LTQ Velos Dual Pressure Linear Ion Trap mass spectrometer which analysed the peptides in positive mode using the following parameters: MS1 60,000 resolution, 100 ms acquisition time, 1 × 106 automatic gain control (AGC), MS2 15,000 resolution, 250 ms acquisition time, 5 × 105 AGC, top 8, 30 normalized collision energy (NCE) higher-energy collisional dissociation (HCD).

### Data processing

Protein sequences of each sample silks were downloaded from UniProtKB and consolidated to a FASTA database for de novo analysis. Among the 11 protein sequences of seven different species of silks, only the Bombyx silks sequences are manually annotated through experiments and reviewed (Swiss-Prot), and the others are automatically annotated from their genomic DNA or mRNA and not reviewed (TrEMBL). Thus, sometimes a silk fibroin has multiple sequences that differ from each other. In such cases, the most recent sequence was selected, and the partial sequences were omitted if it is a part of a more extended sequence. De novo analyses and database search (UniProt TrEMBL/NCBInr) were performed with PEAKS Studio 8.5 (Bioinformatics Solutions Inc., Canada). Trypsin/chymotrypsin/chymotrypsin-trypsin with a maximum number of 2 missed cleavages and both unspecific ends were selected as the protease. Carbamidomethylation (cysteine) was set as fixed modification; Oxidation (methionine) and Deamination (asparagine, glutamine) was set as variable modifications. Precursor mass tolerance was set as 15 ppm, fragment mass tolerances for CID were set to 0.5 Da, respectively. All presented peptide is filtered to have False Discovery Rate at ≤ 1% or peptide probability score −10lgP ≥ 20. Individual mass spectra were interrogated manually to search for the presence of unique peptides and CID product ions were subsequently used to confirm the amino acid sequence of each of the most important differentiating peptides. For the seven samples reprocessed at Smithsonian MCI, precursor mass tolerance was set to 10 ppm, and fragment mass tolerances for HCD was set to 0.02 Da following its usual setting; all other analysis parameters were applied as same as described above. Biological samples commonly contain proteins with slightly different sequences than those in protein databases, e.g., due to polymorphisms, database errors, cross-species database searching, protein degradation etc. Ignoring those mutated peptides can potentially lead to an error in protein confirmation or simply low coverage of proteins. The SPIDER algorithm analyses every confident de novo tag (ALC > 15%) whose spectrum is not identified by PEAKS DB with high confidence (−10lgP < 30) to construct new peptide sequences by altering amino acids of database peptides. When a significant similarity is found, the algorithm tries to use both de novo sequencing errors and homology peptide mutations to explain the differences. More specifically, it reconstructs a “real” sequence to minimize the sum of de novo errors between the real sequence and the de novo sequence, as well as homology peptide mutations between the real sequence and the database sequence. Ultimately, the better sequence constructed by SPIDER or found by PEAKS DB will be assigned as the identified peptide.

### Statistical analysis

In PEAKS software, −10lgP score for protein indicates the statistical significance of the peptide-spectrum match. The P-value is converted to −10*log10(P-value) and is denoted by −10lgP as a probability score. Thus, a more significant match will have a higher −10lgP value. −10lgP values of ≥ 70 for proteins and ≥ 20 for peptides is the general threshold for significance (p < 0.05) when assessing the quality of the peptide-spectrum match in PEAKS Studio 8.5 (Bioinformatics Solutions Inc., Canada). Each sample was directly compared against the database for exact matches. The database search also runs ‘PTM search’ and displays protein post-translational modifications and mutations regarded as confident if the two fragment ions at both sides of the modified residue have relative intensity values higher than the A score > 20 (ambiguity score as −10 × log10 P). Samples were also processed using the SPIDER algorithm, which facilitates detection of these peptide mutations and performs a cross-species homology search.

## Supplementary Information

Supplementary Information.

## Data Availability

All data are available in the main text or the supplementary materials. The mass spectrometry proteomics data have been deposited to the ProteomeXchange Consortium via the PRIDE (70) partner repository with the dataset identifier PXD024610 (DOI: 10.6019/PXD024610) for modern silk samples and PXD024606 (DOI:10.6019/PXD024606) for Palmyra samples.
