# Supplementary material for: Species identification of silks by protein mass spectrometry reveals evidence of wild silk use in antiquity
Source: Sci Rep. 2022 Mar 17;12:4579. doi: 10.1038/s41598-022-08167-3 (PMC8931077; doi:10.1038/s41598-022-08167-3)
Supplement: Supplementary file 1 — Supplementary Information. [file 41598_2022_8167_MOESM1_ESM.docx]

Supplementary Materials for

**Species identification of silks by protein mass spectrometry reveals evidence of wild silk use in antiquity**

Boyoung Lee, Elisabete Pires, A. Mark Pollard, and James S.O. McCullagh

*Corresponding authors: [bo0lee@outlook.com](mailto:bo0lee@outlook.com) and [James.mccullagh@chem.ox.ac.uk](mailto:James.mccullagh@chem.ox.ac.uk)

**This PDF file includes:**

Figs. S1 to S29

Tables S1 to S15

References

Table of Contents

[Fig. S1. A general flowchart for a conventional fibre analysis 5](#_Toc92376765)

[Fig. S2. Comparison of SDS-PAGE analysis of different silk fibroin solution 6](#_Toc92376766)

[Fig. S3. Unique sequence features of *B. mori* fibroin heavy chain (P05790) 7](#_Toc92376767)

[Fig. S4. Unique sequence features (in white letters) of *A. pernyi* (highlighted in purple); *A. mylitta* (highlighted in green); *A. yamamai* (highlighted in yellow); *A. assamensis* (highlighted in red) and *S. ricini* (highlighted in blue). The light grey blocks with light purple highlight (in black letters) indicate residues matches the consensus sequence at that position by the BLOSUM62 scoring matrix. Some nonconserved features of *S. ricini* was not highlighted as *S. ricini* was the only available *Samia* species. 8](#_Toc92376768)

[Fig. S5. Sequence coverage obtained from nanoLC-MS/MS analysis of the tryptic digestion of the *B. mori* sample, matched to the P05790 sequence, shows 5% sequence coverage. The alternating black and green blocks indicate theoretical tryptic peptides. Residues in bold indicate the detected peptides; blue bars indicate the peptides coverage, and the vertical bars mark different cleavage sites. Residues not covered (1700-5000) have been cropped. 9](#_Toc92376769)

[Fig. S6. Sequence coverage obtained from nanoLC-MS/MS analysis of the FA-tryptic digestion of the *B. mori* sample matched to the P05790 sequence shows 9% sequence coverage. The alternating black and green blocks indicate theoretical tryptic peptides. Residues in bold indicate the detected peptides; blue bars indicate the peptides coverage, and the vertical bars mark different cleavage sites. 10](#_Toc92376770)

[Fig. S7. Sequence coverage obtained from nanoLC-MS/MS analysis of the chymotryptic digestion of the *B. mori* sample matched to the P05790 sequence shows 28% sequence coverage. The alternating black and green blocks indicate theoretical tryptic peptides. Residues in bold indicate the detected peptides; blue bars indicate the peptides coverage, and the vertical bars mark different cleavage sites. 11](#_Toc92376771)

[Fig. S8. Sequence coverage obtained from nanoLC-MS/MS analysis of the tryptic-chymotryptic digestion of the *B. mori* sample matched to the P05790 sequence shows 19% sequence coverage. The alternating black and green blocks indicate theoretical tryptic peptides. Residues in bold indicate the detected peptides; blue bars indicate the peptides coverage, and the vertical bars mark different cleavage sites. 12](#_Toc92376772)

[Fig. S9. Sequence coverage obtained from nanoLC-MS/MS analysis of the tryptic digestion of the *A. pernyi* sample matched to the O76786 sequence shows 13% sequence coverage. The alternating black and green blocks indicate theoretical tryptic peptides. Residues in bold indicate the detected peptides; blue bars indicate the peptides coverage, and the vertical bars mark different cleavage sites. 13](#_Toc92376773)

[Fig. S10. Sequence coverage obtained from nanoLC-MS/MS analysis of the FA-tryptic digestion of the *A. pernyi* sample matched to the O76786 sequence shows 21% sequence coverage. The alternating black and green blocks indicate theoretical tryptic peptides. Residues in bold indicate the detected peptides; blue bars indicate the peptides coverage, and the vertical bars mark different cleavage sites. 14](#_Toc92376774)

[Fig. S11. Sequence coverage obtained from nanoLC-MS/MS analysis of the chymotryptic digestion of the *A. pernyi* sample matched to the O76786 sequence shows 45% sequence coverage. The alternating black and green blocks indicate theoretical tryptic peptides. Residues in bold indicate the detected peptides; blue bars indicate the peptides coverage, and the vertical bars mark different cleavage sites. 15](#_Toc92376775)

[Fig. S12. Sequence coverage obtained from nanoLC-MS/MS analysis of the tryptic-chymotryptic digestion of the *A. pernyi* sample matched to the O76786 sequence shows 23% sequence coverage. The alternating black and green blocks indicate theoretical tryptic peptides. Residues in bold indicate the detected peptides; blue bars indicate the peptides coverage, and the vertical bars mark different cleavage sites. 16](#_Toc92376776)

[Fig. S13. Sequence coverage obtained from nanoLC-MS/MS analysis of the tryptic digestion of the *A. mylitta* sample matched to the Q8ISB3 shows 39% sequence coverage. The alternating black and green blocks indicate theoretical tryptic peptides. Residues in bold indicate the detected peptides; blue bars indicate the peptides coverage, and the vertical bars mark different cleavage sites. 17](#_Toc92376777)

[Fig. S14. Sequence coverage obtained from nanoLC-MS/MS analysis of the FA-tryptic digestion of an *A. mylitta* sample matched to the Q8ISB3 sequence shows 49% sequence coverage. The alternating black and green blocks indicate theoretical tryptic peptides; residues in bold indicate the detected peptides; blue bars indicate the peptides coverage, and the vertical bars mark different cleavage sites. 17](#_Toc92376778)

[Fig. S15. Sequence coverage obtained from nanoLC-MS/MS analysis of the chymotryptic digestion of the *A. mylitta* sample matched to the Q8ISB3 sequence shows 71% sequence coverage. The alternating black and green blocks indicate theoretical tryptic peptides. Residues in bold indicate the detected peptides; blue bars indicate the peptides coverage, and the vertical bars mark different cleavage sites. 18](#_Toc92376779)

[Fig. S16. Sequence coverage obtained from nanoLC-MS/MS analysis of the tryptic-chymotryptic digestion of the *A. mylitta* sample matched to the Q8ISB3 sequence shows 56% sequence coverage. The alternating black and green blocks indicate theoretical tryptic peptides. Residues in bold indicate the detected peptides; blue bars indicate the peptides coverage, and the vertical bars mark different cleavage sites. 19](#_Toc92376780)

[Fig. S17. Sequence coverage obtained from nanoLC-MS/MS analysis of the tryptic digestion of the *A. yamamai* sample matched to the E1CGA3 sequence shows 12% coverage. The alternating black and green blocks indicate theoretical tryptic peptides. Residues in bold indicate the detected peptides; blue bars indicate the peptides coverage, and the vertical bars mark different cleavage sites. 20](#_Toc92376781)

[Fig. S18. Sequence coverage obtained from nanoLC-MS/MS analysis of the FA-tryptic digestion of the *A. yamamai* sample matched to the E1CGA3 sequence shows 19% sequence coverage. The alternating black and green blocks indicate theoretical tryptic peptides. Residues in bold indicate the detected peptides; blue bars indicate the peptides coverage, and the vertical bars mark different cleavage sites. 21](#_Toc92376782)

[Fig. S19. Sequence coverage obtained from nanoLC-MS/MS analysis of the chymotryptic digestion of the *A. yamamai* sample matched to the E1CGA3 sequence shows 30% sequence coverage. The alternating black and green blocks indicate theoretical tryptic peptides. Residues in bold indicate the detected peptides; blue bars indicate the peptides coverage, and the vertical bars mark different cleavage sites. 22](#_Toc92376783)

[Fig. S20. Sequence coverage obtained from nanoLC-MS/MS analysis of the tryptic-chymotryptic digestion of the *A. yamamai* sample matched to the E1CGA3 sequence shows 18% sequence coverage. The alternating black and green blocks indicate theoretical tryptic peptides. Residues in bold indicate the detected peptides; blue bars indicate the peptides coverage, and the vertical bars mark different cleavage sites. 23](#_Toc92376784)

[Fig. S21. Sequence coverage obtained from nanoLC-MS/MS analysis of the tryptic digestion of the *A. assamensis* sample matched to the A0A0K0KR73 sequence shows 8% sequence coverage. The alternating black and green blocks indicate theoretical tryptic peptides. Residues in bold indicate the detected peptides; blue bars indicate the peptides coverage, and the vertical bars mark different cleavage sites. 24](#_Toc92376785)

[Fig. S22. Sequence coverage obtained from nanoLC-MS/MS analysis of the FA-tryptic digestion of the *A. assamensis* sample matched to the A0A0K0KR73 sequence shows 16% sequence coverage. The alternating black and green blocks indicate theoretical tryptic peptides. Residues in bold indicate the detected peptides; blue bars indicate the peptides coverage, and the vertical bars mark different cleavage sites. 25](#_Toc92376786)

[Fig. S23. Sequence coverage obtained from nanoLC-MS/MS analysis of the chymotryptic digestion of the *A. assamensis* sample matched to the A0A0K0KR73 sequence shows 47% sequence coverage. The alternating black and green blocks indicate theoretical tryptic peptides. Residues in bold indicate the detected peptides; blue bars indicate the peptides coverage, and the vertical bars mark different cleavage sites. 26](#_Toc92376787)

[Fig. S24. Sequence coverage obtained from nanoLC-MS/MS analysis of the tryptic-chymotryptic digestion of the *A. assamensis* sample matched to the A0A0K0KR73 sequence shows 51% sequence coverage. The alternating black and green blocks indicate theoretical tryptic peptides. Residues in bold indicate the detected peptides; blue bars indicate the peptides coverage, and the vertical bars mark different cleavage sites. 27](#_Toc92376788)

[Fig. S25. Sequence coverage obtained from nanoLC-MS/MS analysis of the tryptic digestion of the *S. ricini* sample matched to the A0A0D5ZYI3 sequence shows 9% sequence coverage. The alternating black and green blocks indicate theoretical tryptic peptides. Residues in bold indicate the detected peptides; blue bars indicate the peptides coverage, and the vertical bars mark different cleavage sites. 28](#_Toc92376789)

[Fig. S26. Sequence coverage obtained from nanoLC-MS/MS analysis of the FA-tryptic digestion of the *S. ricini* sample matched to the A0A0D5ZYI3 sequence shows 21% sequence coverage. The alternating black and green blocks indicate theoretical tryptic peptides. Residues in bold indicate the detected peptides; blue bars indicate the peptides coverage, and the vertical bars mark different cleavage sites. 29](#_Toc92376790)

[Fig. S27. Sequence coverage obtained from nanoLC-MS/MS analysis of the chymotryptic digestion of the *S. ricini* sample matched to the A0A0D5ZYI3 sequence shows 41% sequence coverage. The alternating black and green blocks indicate theoretical tryptic peptides. Residues in bold indicate the detected peptides; blue bars indicate the peptides coverage, and the vertical bars mark different cleavage sites. 30](#_Toc92376791)

[Fig. S28. Sequence coverage obtained from nanoLC-MS/MS analysis of the tryptic-chymotryptic digestion of the *S. ricini* sample matched to the A0A0D5ZYI3 sequence shows 30% sequence coverage. The alternating black and green blocks indicate theoretical tryptic peptides. Residues in bold indicate the detected peptides; blue bars indicate the peptides coverage, and the vertical bars mark different cleavage sites. 31](#_Toc92376792)

[Fig. S29. Non-enzymatic cleavages found in experimental samples 32](#_Toc92376793)

[Table S1. The characteristic hexapeptide GAGAG(X) and subsequences unique to *B. mori* heavy chain protein (UniProt accession: P05790) 33](#_Toc92376794)

[Table S2. Homology (%) between the silk fibroin sequences through Pairwise alignment 33](#_Toc92376795)

[Table S3. Amino acid compositions of *B. mori, A. pernyi, A. mylitta, A. yamamai, A. assamensis*, and *S. ricini* silk fibroins (exclude signal peptides) 1](#_Toc92376796)

[Table S4. List of unique peptides detected from *B. mori* digested with trypsin, FA-trypsin, chymotrypsin, and chymotrypsin-trypsin at False Discovery Rate ≤1% 2](#_Toc92376797)

[Table S5. List of unique peptides detected from *A. pernyi* digested with trypsin, FA-trypsin, chymotrypsin, and chymotrypsin-trypsin at False Discovery Rate ≤1%. 17](#_Toc92376798)

[Table S6. List of unique peptides detected from *A. mylitta* digested with trypsin, FA-trypsin, chymotrypsin, and chymotrypsin-trypsin at False Discovery Rate ≤1%. 22](#_Toc92376799)

[Table S7. List of unique peptides detected from *A. yamamai* digested with trypsin, FA-trypsin, chymotrypsin, and chymotrypsin-trypsin at False Discovery Rate ≤1% 29](#_Toc92376800)

[Table S8. List of unique peptides detected from *A. assamensis* digested with trypsin, FA-trypsin, chymotrypsin, and chymotrypsin-trypsin at False Discovery Rate ≤1% 35](#_Toc92376801)

[Table S9. List of unique peptides detected from *S. ricini* digested with trypsin, FA-trypsin, chymotrypsin, and chymotrypsin-trypsin at False Discovery Rate ≤1% 56](#_Toc92376802)

[Table S10. Comparison of tryptic peptides of *B. mori* fibroin yielded by *in-silico* trypsin digestion, experimental trypsin digestion, and experimental formic acid-trypsin digestion 81](#_Toc92376803)

[Table S11. List of peptides detected from sample A (S8 warp) at peptide confidence score -10lgP ≥ 20 89](#_Toc92376804)

[Table S12. List of peptides detected from sample B (S8 weft) at peptide confidence score -10lgP ≥ 20 92](#_Toc92376805)

[Table S13. List of peptides detected from sample C (S48) at peptide confidence score -10lgP ≥ 20 96](#_Toc92376806)

[Table S14. List of peptides detected from sample D (S49-T1) at peptide confidence score -10lgP ≥ 20 97](#_Toc92376807)

[Table S15. List of peptides detected from sample E (S49-T2) at peptide confidence score -10lgP ≥ 20 99](#_Toc92376808)

[References 101](#_Toc92376809)


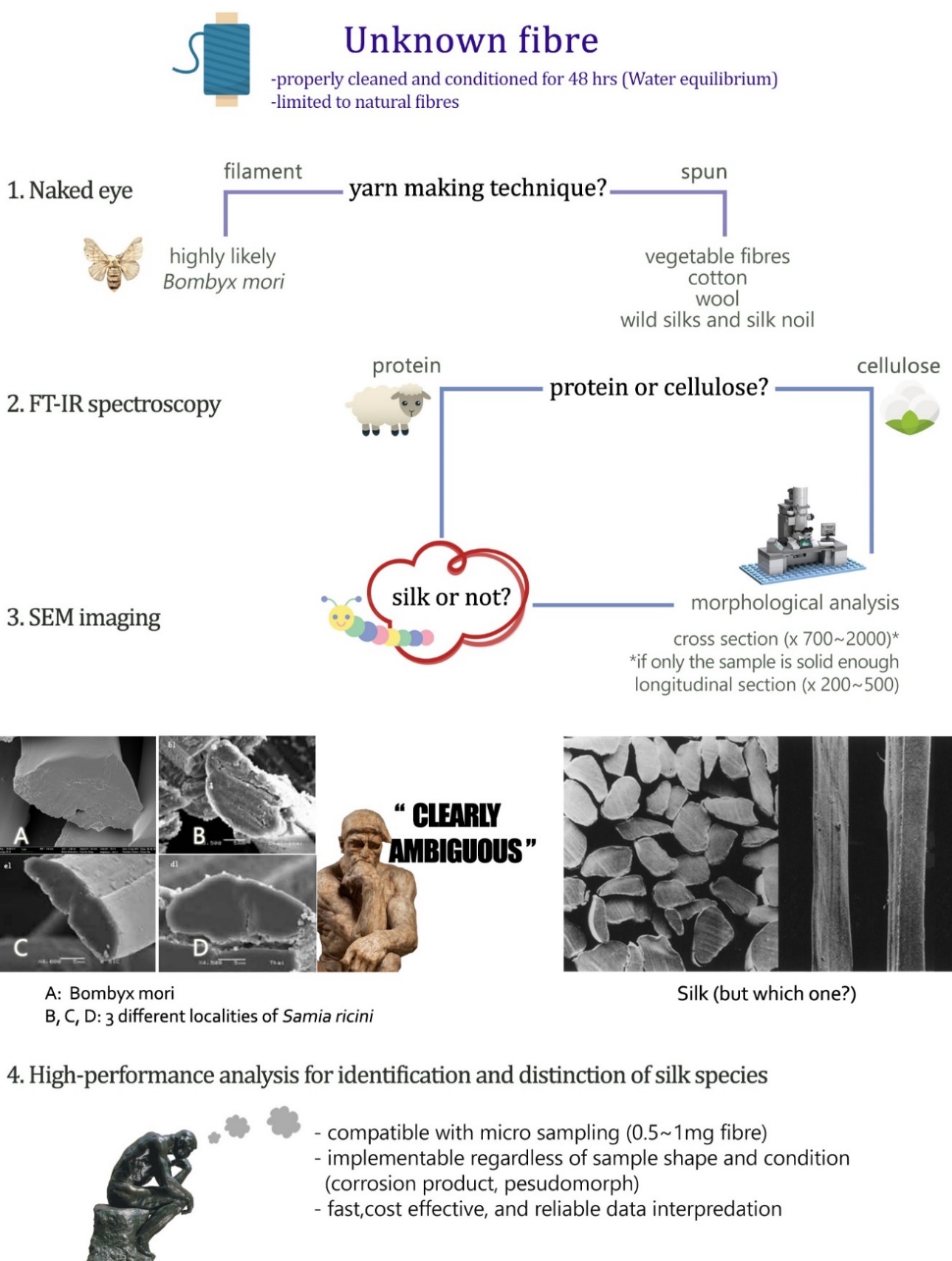


Fig. S1. A general flowchart for a conventional fibre analysis

First, the investigation of thread making techniques must take place. In most cases, natural fibres occur in short lengths about 5 to 15 centimetres so spun into yarns, while *B. mori* silkworm can produce a continuous filament of 300-1500m.(*1*) ATR (attenuated total reflection)-FTIR analysis can tell whether the fibre is proteinaceous or cellulosic by the presence of N-H stretching and amide bands. However, it only gives information on the compounds and cannot be applied to fragile samples since the sample needs to be tightly pressed against the ATR crystal. Once the compound characteristic of the sample is known, optical microscopy such as SEM can be useful. The morphology of silk fibres shows many variations depending upon the silkworm species and among individual cocoons of the same species. The cross-section of cultivated silk fibres is often irregular, ranging from the triangular shape to circular shape.(*2*) Wild varieties exhibit an elongated wedge shape that is large in diameter compared to *B. mori*, however the irregularity of shape occurs even within the same filament, depending upon the level of the cocoon layer.(*3*)


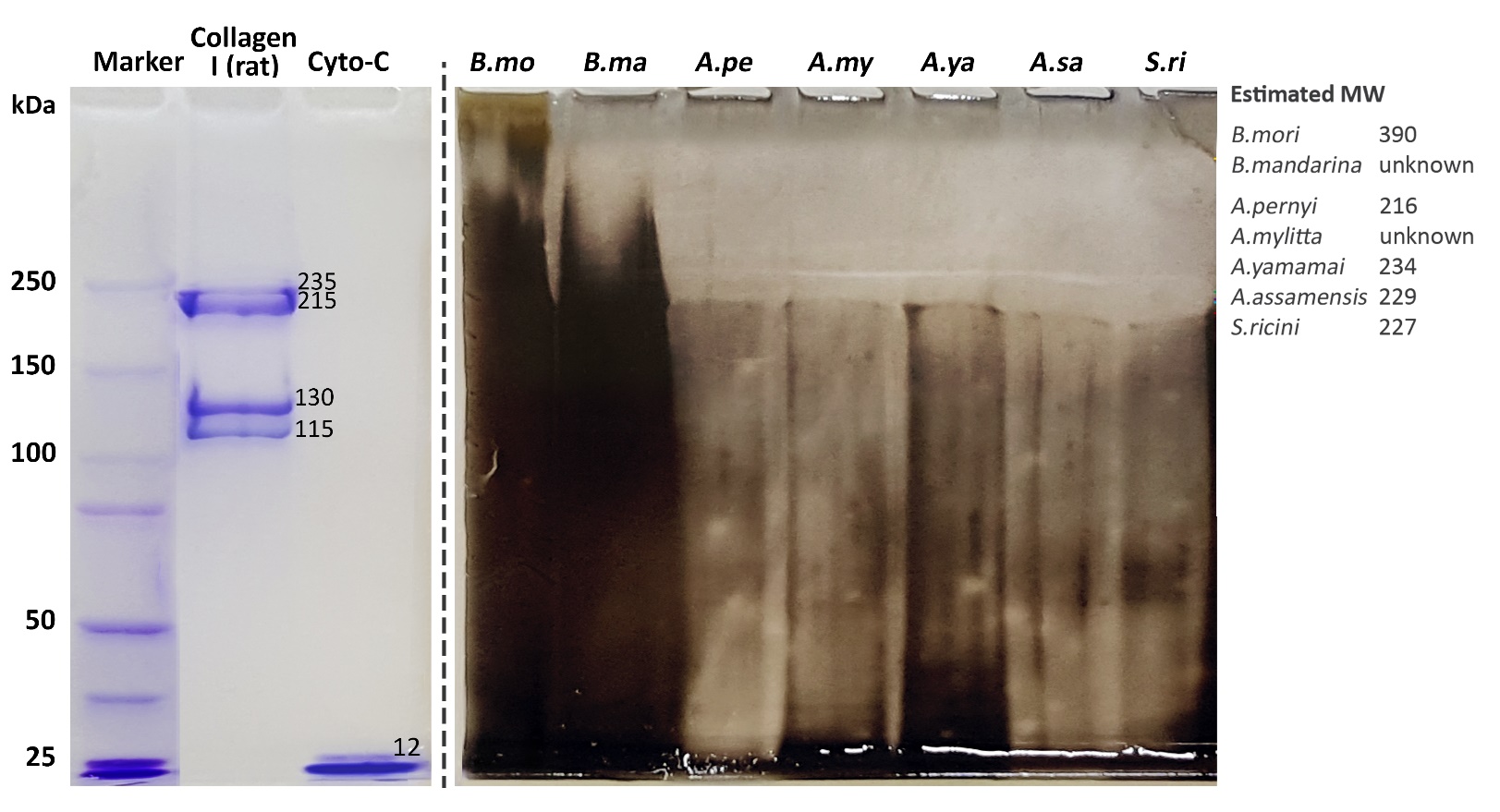


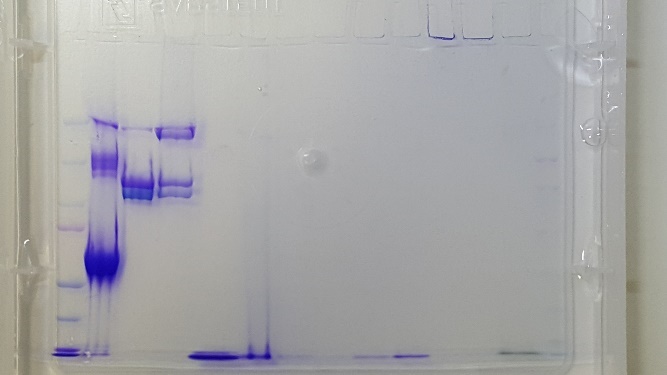

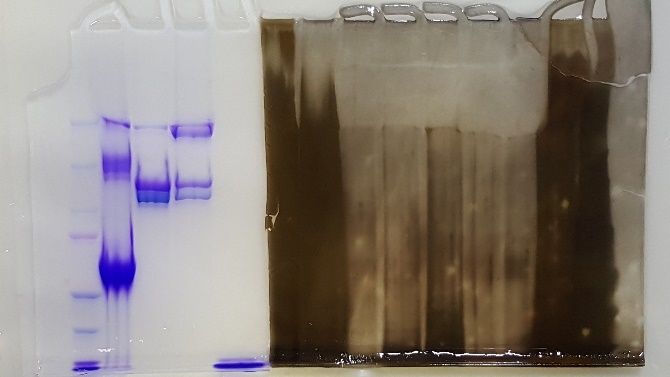


Fig. S2. Comparison of SDS-PAGE analysis of different silk fibroin solution

The presented is an SDS-PAGE result of standard proteins and fibroin solutions in a 3-8% Tris-Acetate gel (40-400kDa), partially silver stained over Coomassie blue. The fibroins did not react well to Coomassie R stain, likely due to the lack of basic amino acid residues.(*4*) The unstained part of the gel was cut, and silver stained. Intact heavy chain (HC) protein from *Bombyx* *mori* fibroin is expected at 390kDa and fibroins from *Antheraea* and *Samia* silks are expected at approximately 220kDa. The right end of the gel contained another lane of marker proteins is cropped for better visibility. See the below two images for the original photographs of the gel after Coomassie and partial silver staining.


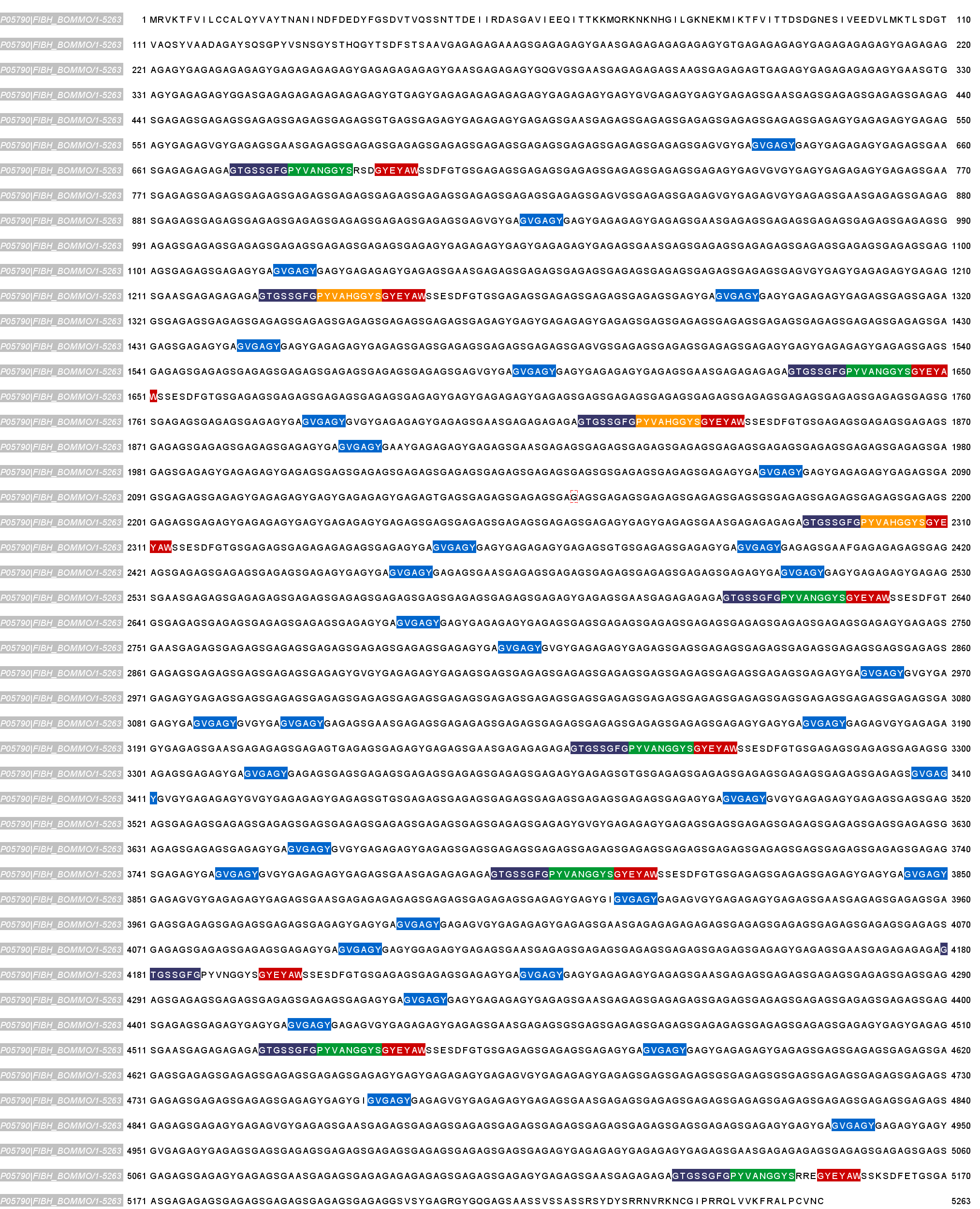


Fig. S3. Unique sequence features of *B. mori* fibroin heavy chain (P05790)

Unique residues other than the GAGAG(X) sequences are marked here in different color block: GVGAGY GTGSSGFG GYEYAW PYVANGGYS PYVAHGGYS.


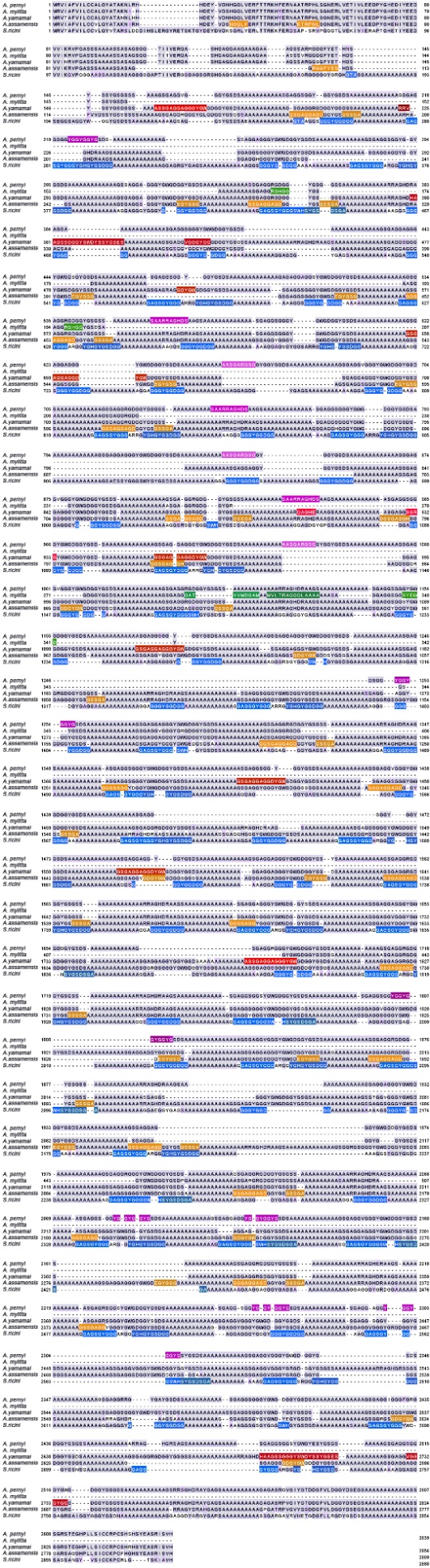


Fig. S4. Unique sequence features (in white letters) of *A. pernyi* (highlighted in purple); *A. mylitta* (highlighted in green); *A. yamamai* (highlighted in yellow); *A. assamensis* (highlighted in red) and *S. ricini* (highlighted in blue). The light grey blocks with light purple highlight (in black letters) indicate residues matches the consensus sequence at that position by the BLOSUM62 scoring matrix. Some nonconserved features of *S. ricini* was not highlighted as *S. ricini* was the only available *Samia* species.


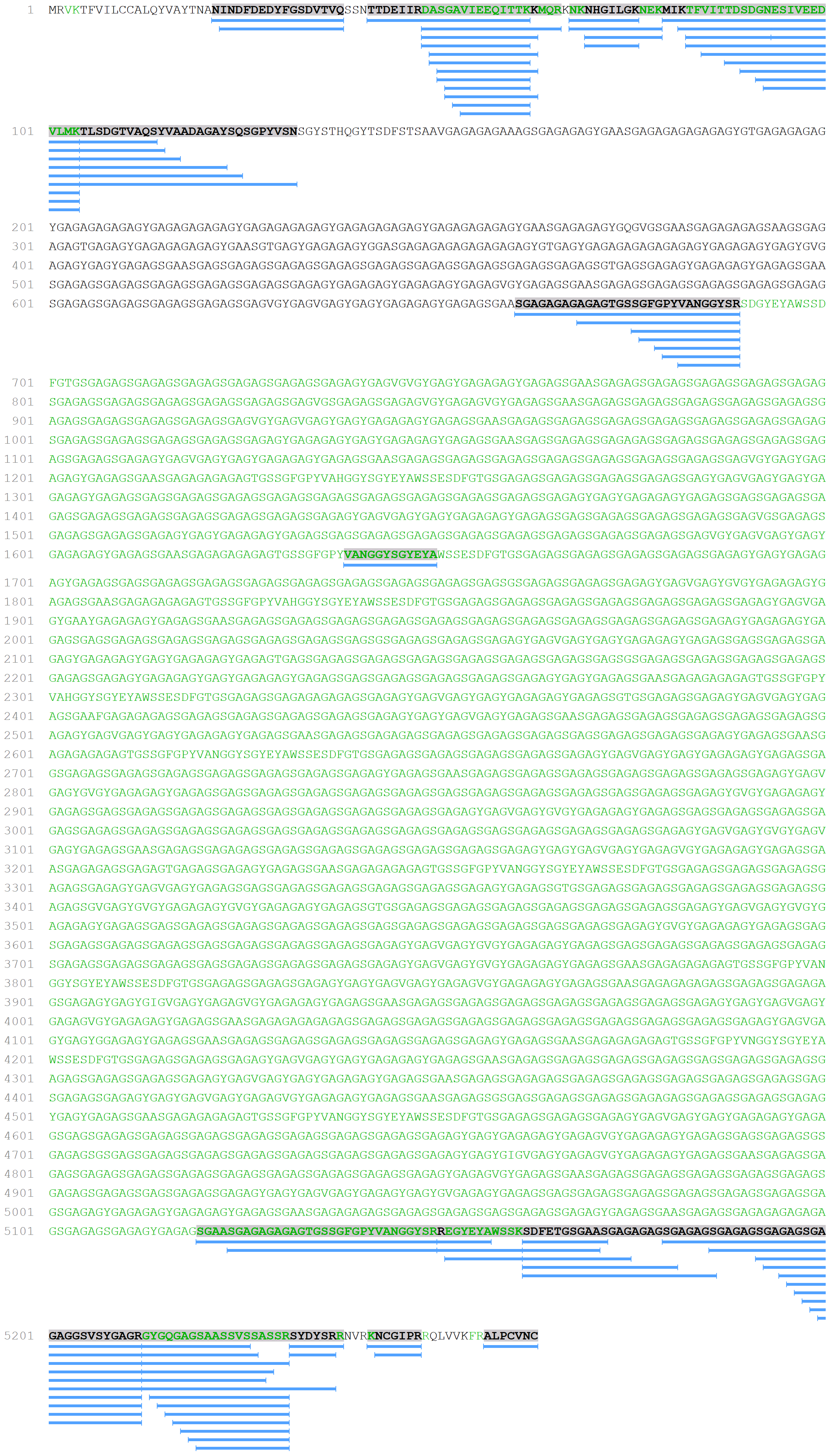


….


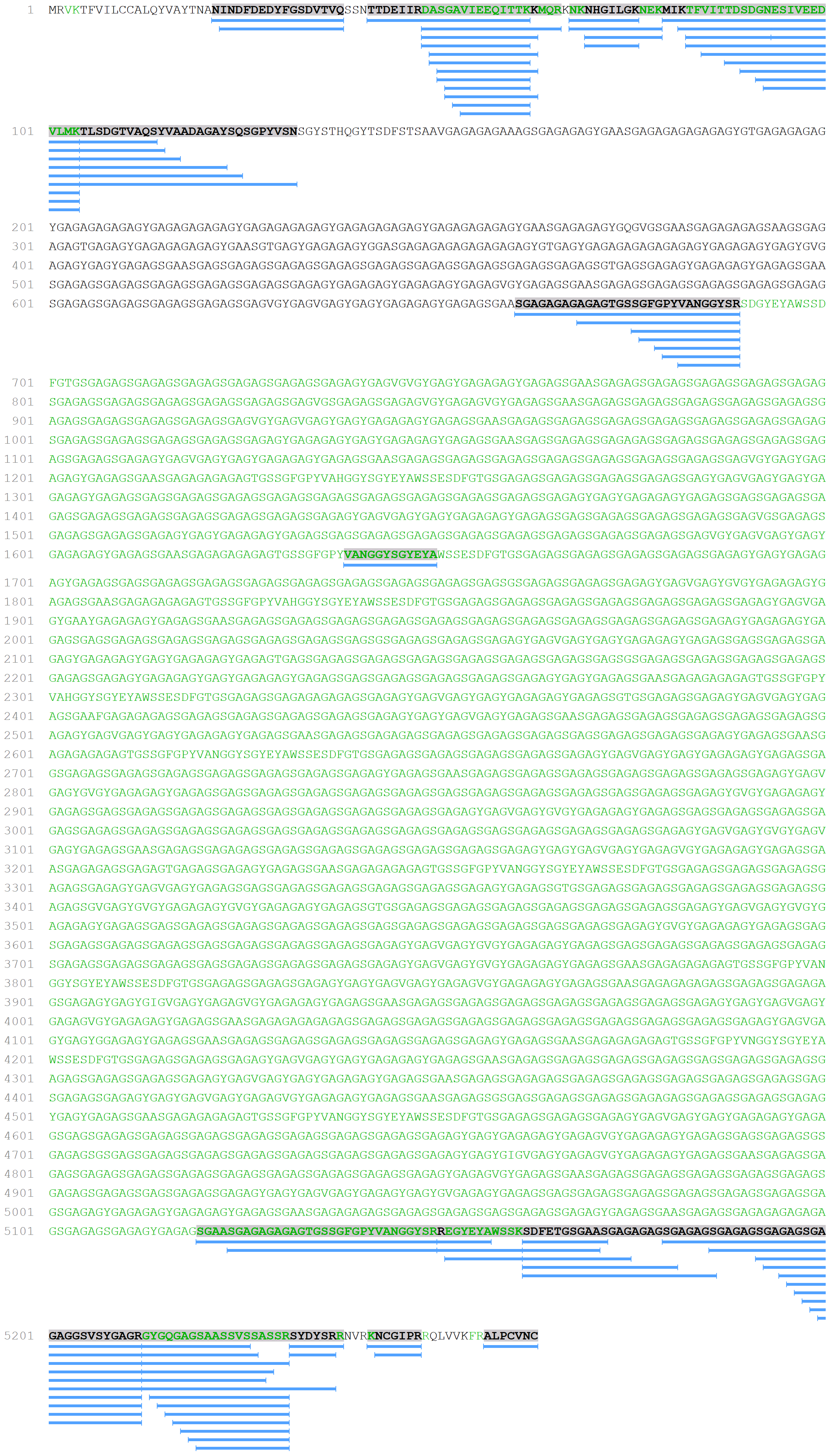


Fig. S5. Sequence coverage obtained from nanoLC-MS/MS analysis of the tryptic digestion of the *B. mori* sample, matched to the P05790 sequence, shows 5% sequence coverage. The alternating black and green blocks indicate theoretical tryptic peptides. Residues in bold indicate the detected peptides; blue bars indicate the peptides coverage, and the vertical bars mark different cleavage sites. Residues not covered (1700-5000) have been cropped.


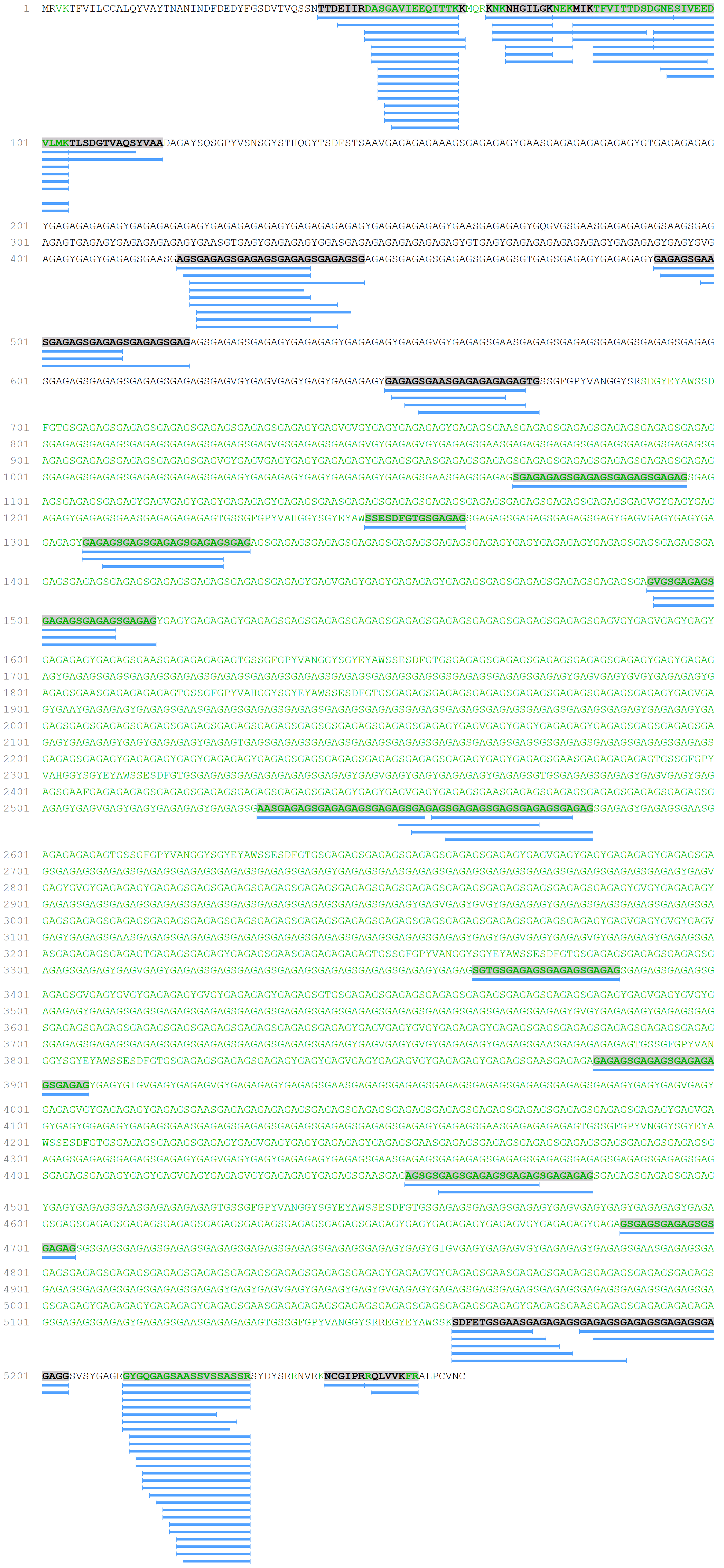


Fig. S6. Sequence coverage obtained from nanoLC-MS/MS analysis of the FA-tryptic digestion of the *B. mori* sample matched to the P05790 sequence shows 9% sequence coverage. The alternating black and green blocks indicate theoretical tryptic peptides. Residues in bold indicate the detected peptides; blue bars indicate the peptides coverage, and the vertical bars mark different cleavage sites.


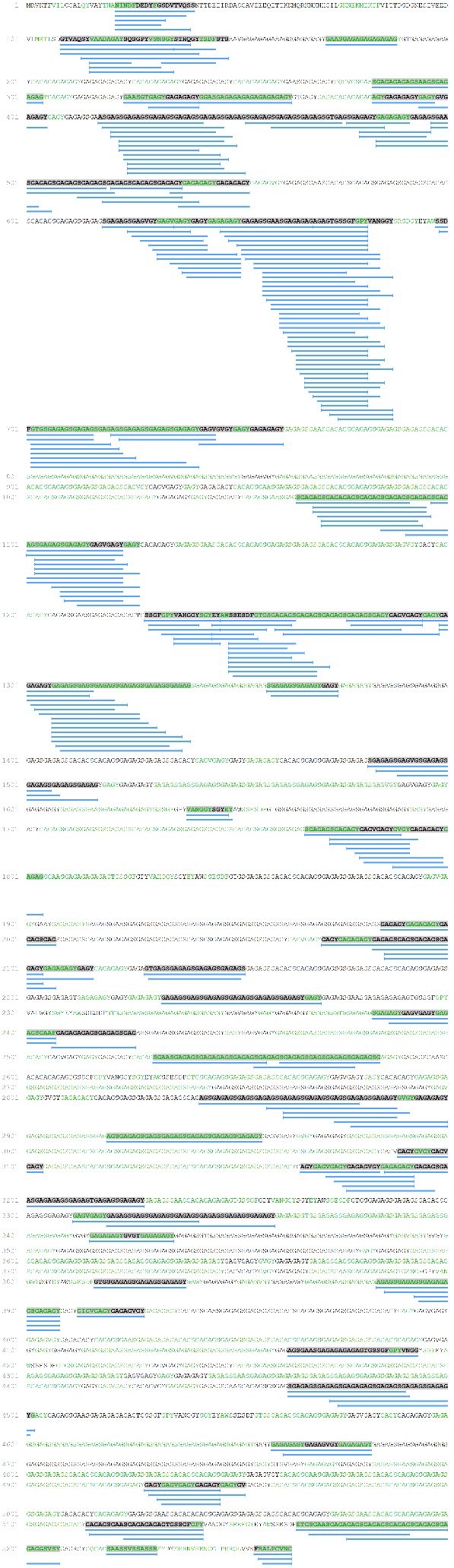


Fig. S7. Sequence coverage obtained from nanoLC-MS/MS analysis of the chymotryptic digestion of the *B. mori* sample matched to the P05790 sequence shows 28% sequence coverage. The alternating black and green blocks indicate theoretical tryptic peptides. Residues in bold indicate the detected peptides; blue bars indicate the peptides coverage, and the vertical bars mark different cleavage sites.


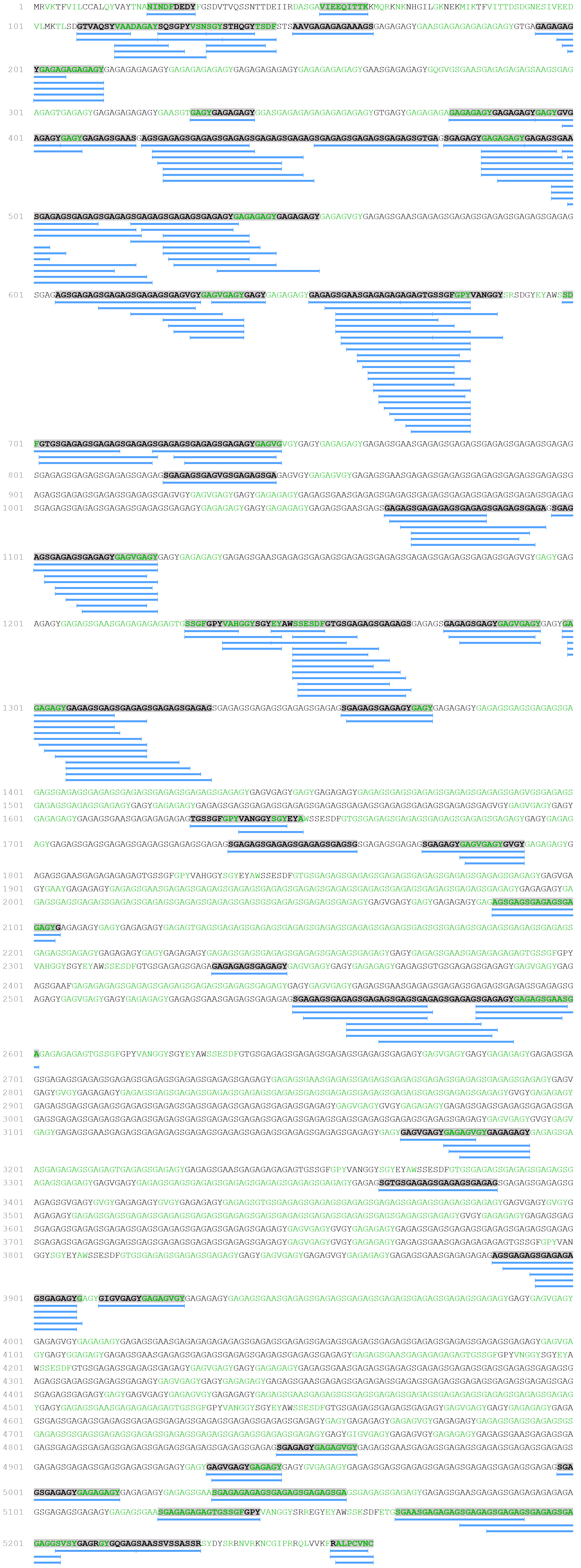


Fig. S8. Sequence coverage obtained from nanoLC-MS/MS analysis of the tryptic-chymotryptic digestion of the *B. mori* sample matched to the P05790 sequence shows 19% sequence coverage. The alternating black and green blocks indicate theoretical tryptic peptides. Residues in bold indicate the detected peptides; blue bars indicate the peptides coverage, and the vertical bars mark different cleavage sites.


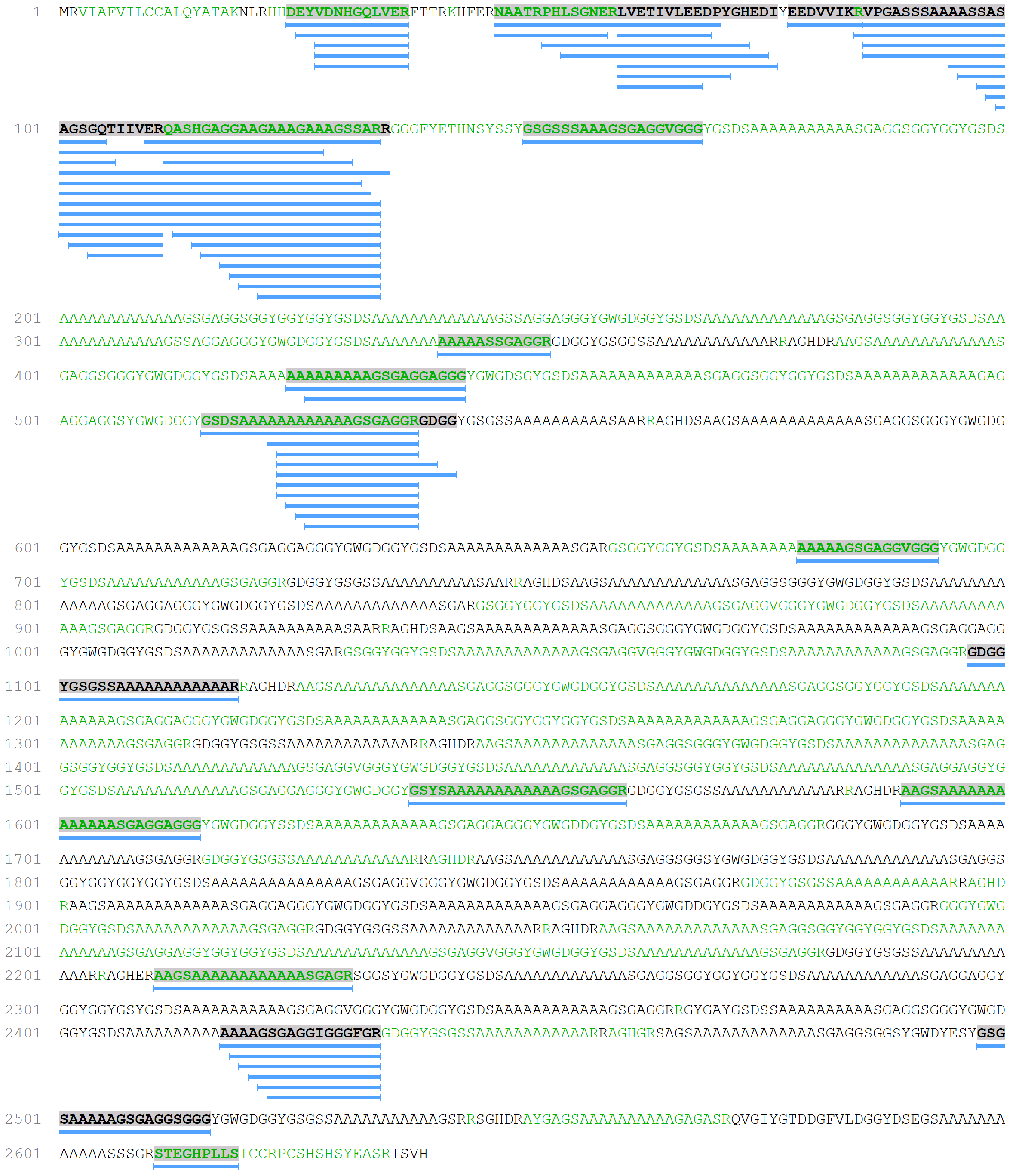


Fig. S9. Sequence coverage obtained from nanoLC-MS/MS analysis of the tryptic digestion of the *A. pernyi* sample matched to the O76786 sequence shows 13% sequence coverage. The alternating black and green blocks indicate theoretical tryptic peptides. Residues in bold indicate the detected peptides; blue bars indicate the peptides coverage, and the vertical bars mark different cleavage sites.


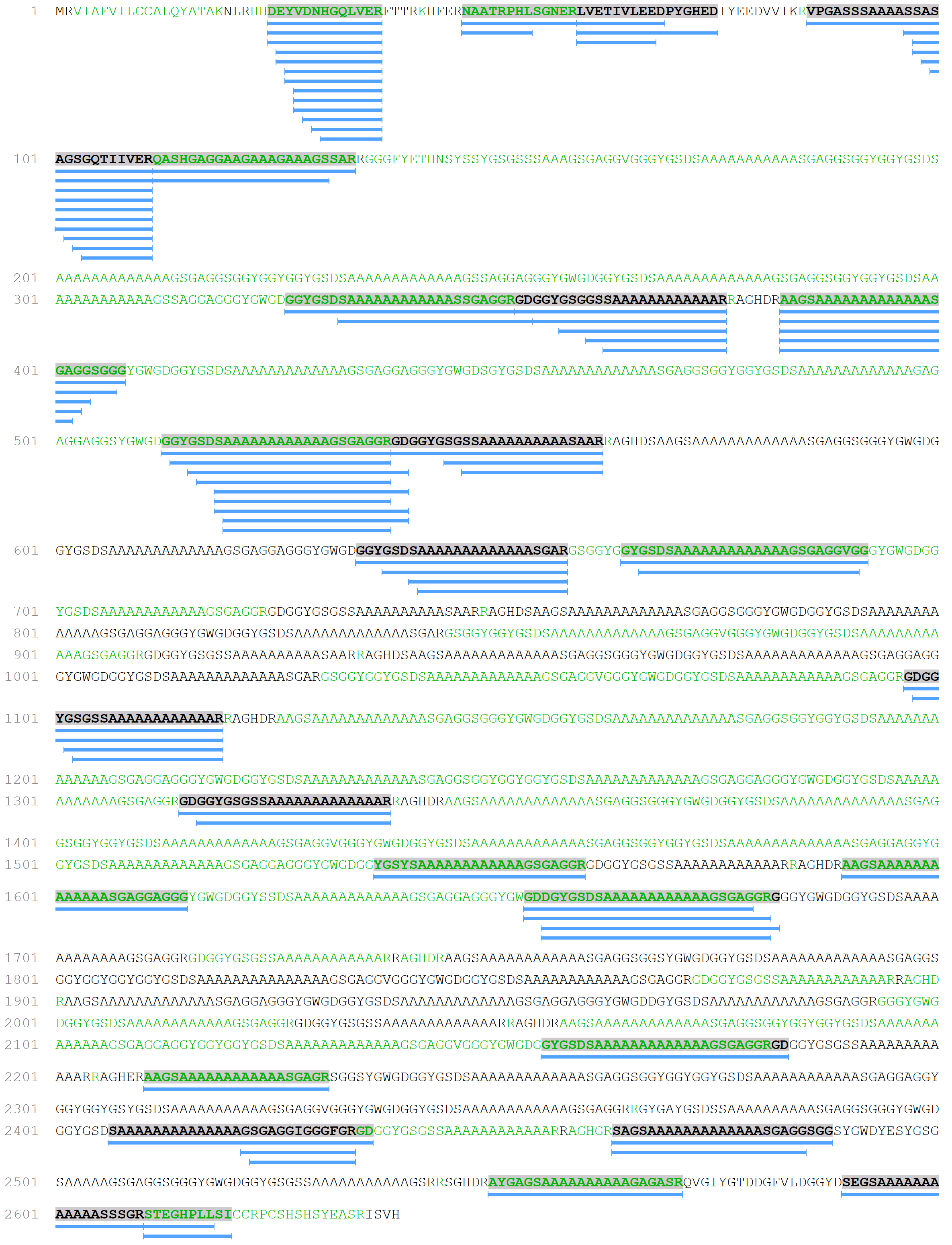


Fig. S10. Sequence coverage obtained from nanoLC-MS/MS analysis of the FA-tryptic digestion of the *A. pernyi* sample matched to the O76786 sequence shows 21% sequence coverage. The alternating black and green blocks indicate theoretical tryptic peptides. Residues in bold indicate the detected peptides; blue bars indicate the peptides coverage, and the vertical bars mark different cleavage sites.


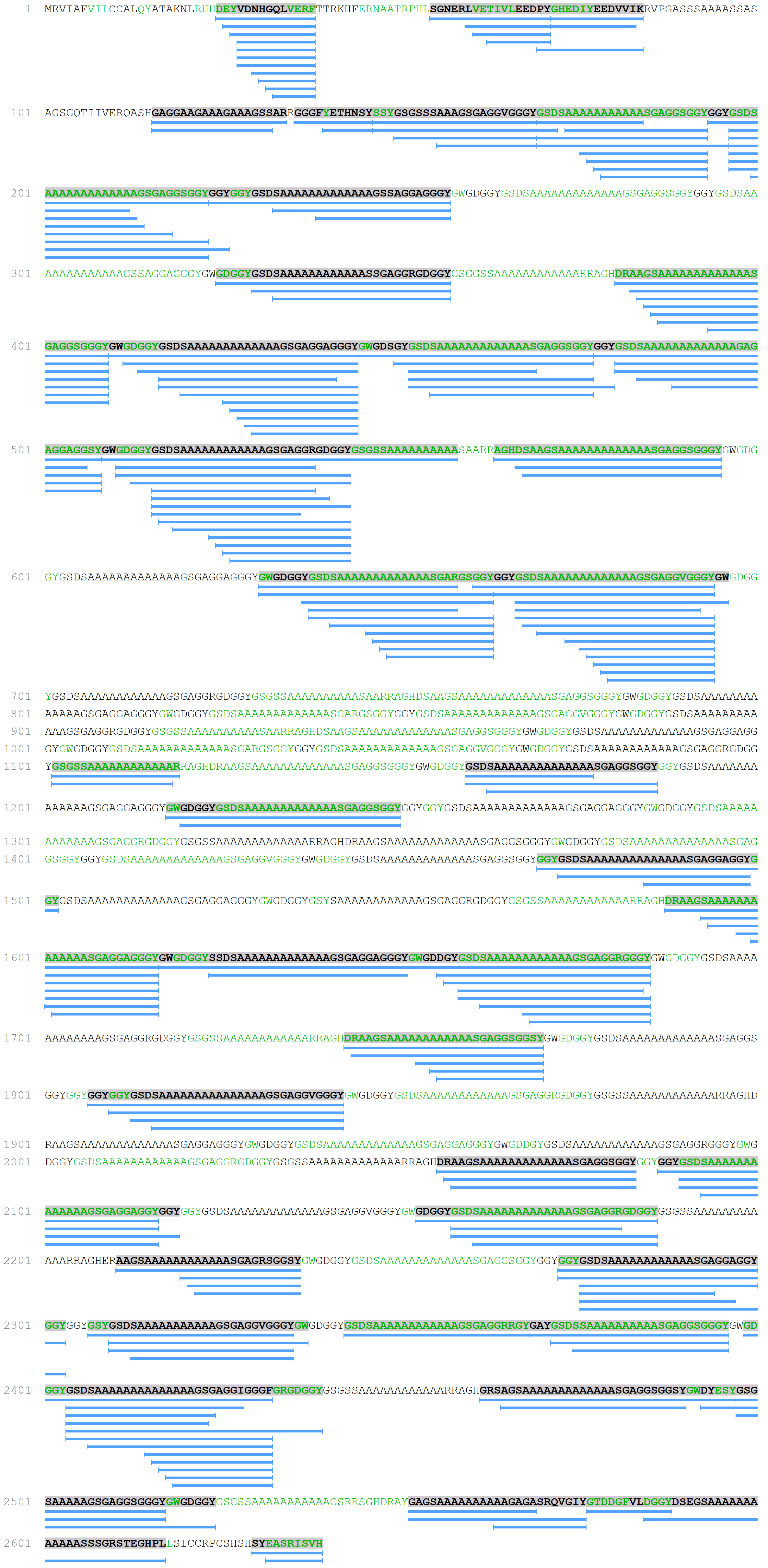


Fig. S11. Sequence coverage obtained from nanoLC-MS/MS analysis of the chymotryptic digestion of the *A. pernyi* sample matched to the O76786 sequence shows 45% sequence coverage. The alternating black and green blocks indicate theoretical tryptic peptides. Residues in bold indicate the detected peptides; blue bars indicate the peptides coverage, and the vertical bars mark different cleavage sites.


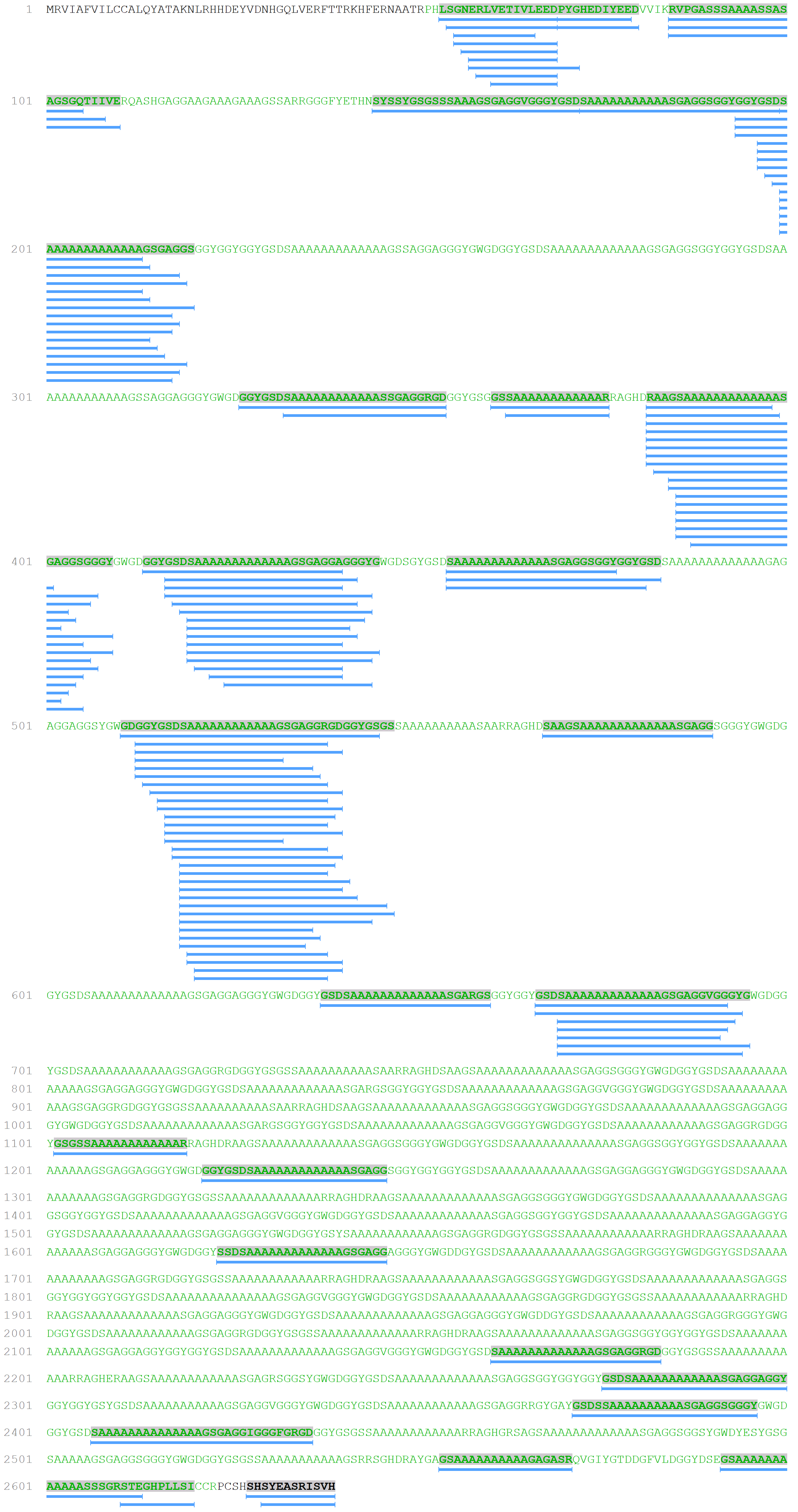


Fig. S12. Sequence coverage obtained from nanoLC-MS/MS analysis of the tryptic-chymotryptic digestion of the *A. pernyi* sample matched to the O76786 sequence shows 23% sequence coverage. The alternating black and green blocks indicate theoretical tryptic peptides. Residues in bold indicate the detected peptides; blue bars indicate the peptides coverage, and the vertical bars mark different cleavage sites.


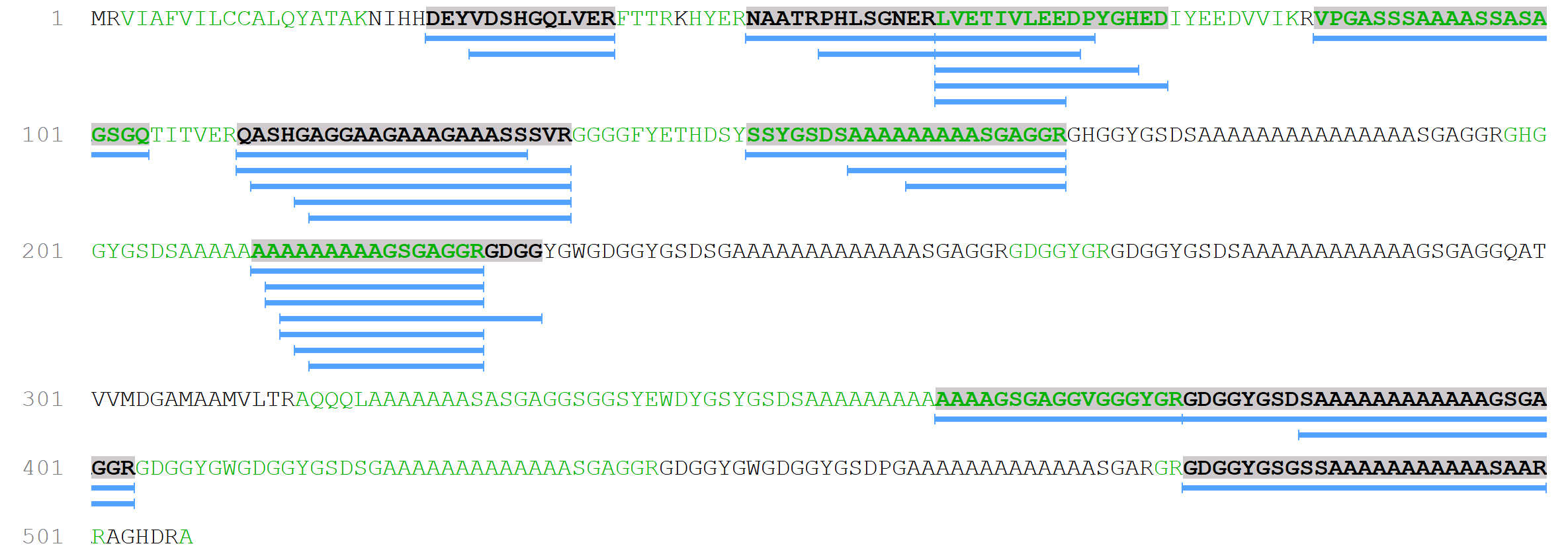


Fig. S13. Sequence coverage obtained from nanoLC-MS/MS analysis of the tryptic digestion of the *A. mylitta* sample matched to the Q8ISB3 shows 39% sequence coverage. The alternating black and green blocks indicate theoretical tryptic peptides. Residues in bold indicate the detected peptides; blue bars indicate the peptides coverage, and the vertical bars mark different cleavage sites.


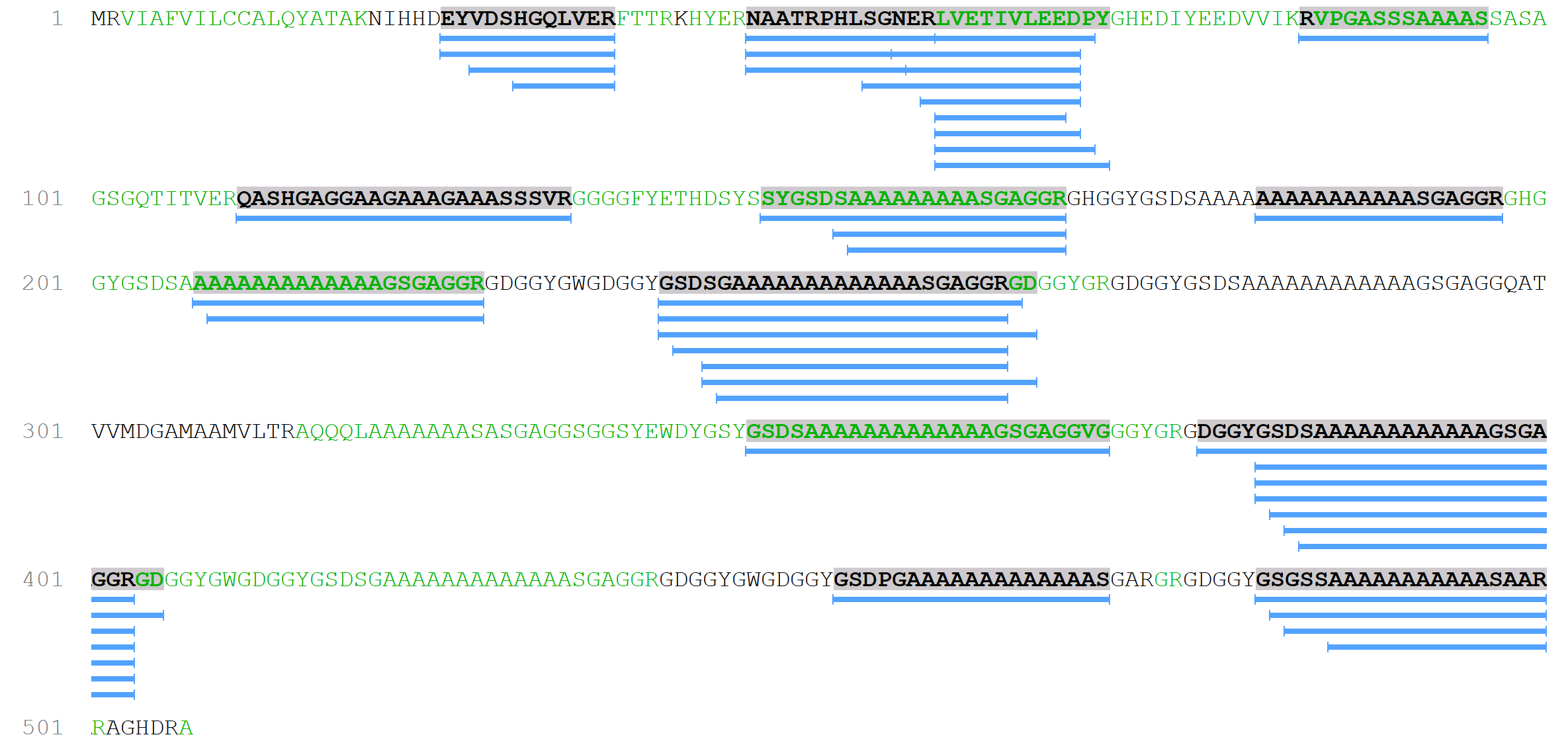


Fig. S14. Sequence coverage obtained from nanoLC-MS/MS analysis of the FA-tryptic digestion of an *A. mylitta* sample matched to the Q8ISB3 sequence shows 49% sequence coverage. The alternating black and green blocks indicate theoretical tryptic peptides; residues in bold indicate the detected peptides; blue bars indicate the peptides coverage, and the vertical bars mark different cleavage sites.


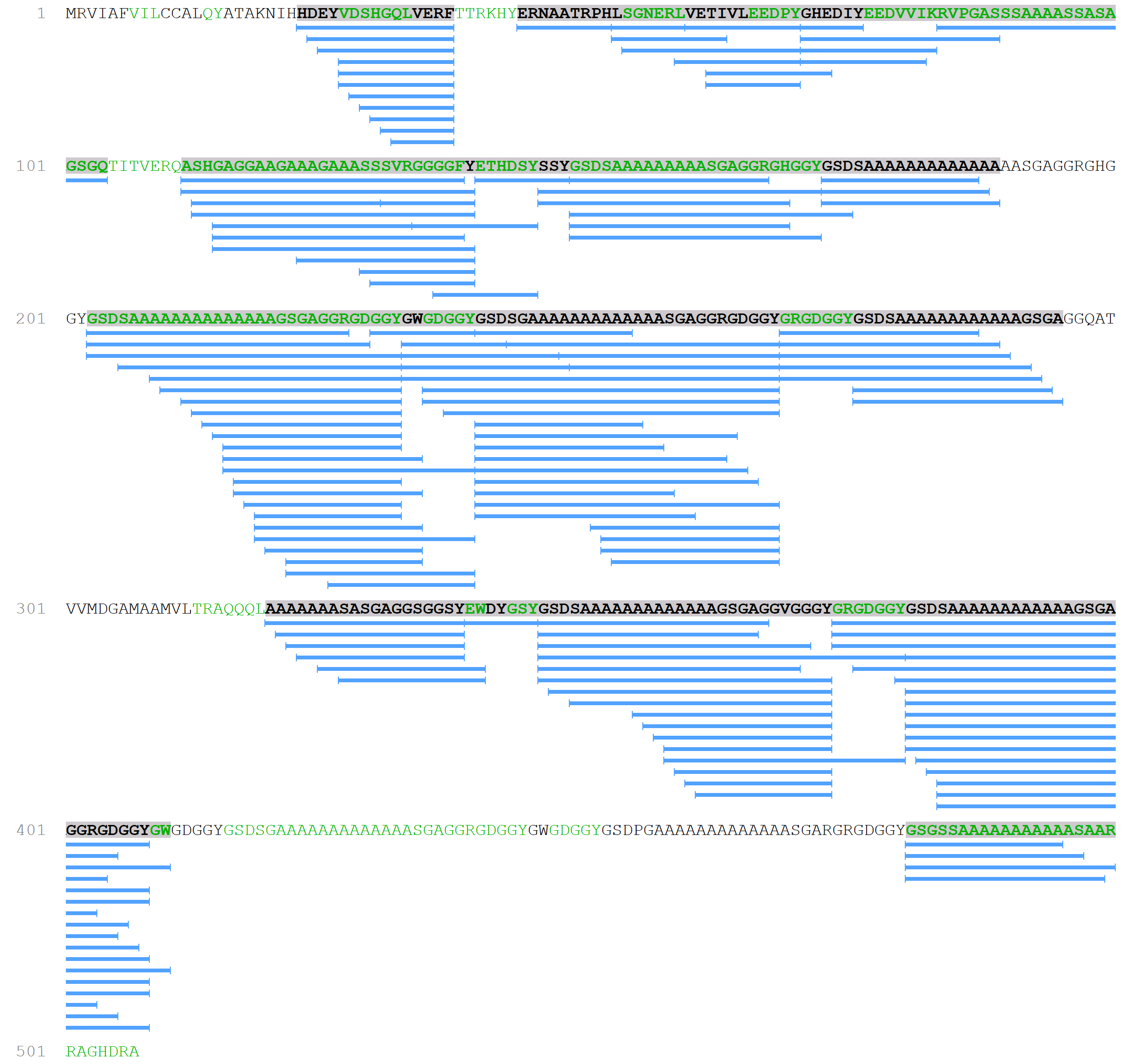


Fig. S15. Sequence coverage obtained from nanoLC-MS/MS analysis of the chymotryptic digestion of the *A. mylitta* sample matched to the Q8ISB3 sequence shows 71% sequence coverage. The alternating black and green blocks indicate theoretical tryptic peptides. Residues in bold indicate the detected peptides; blue bars indicate the peptides coverage, and the vertical bars mark different cleavage sites.


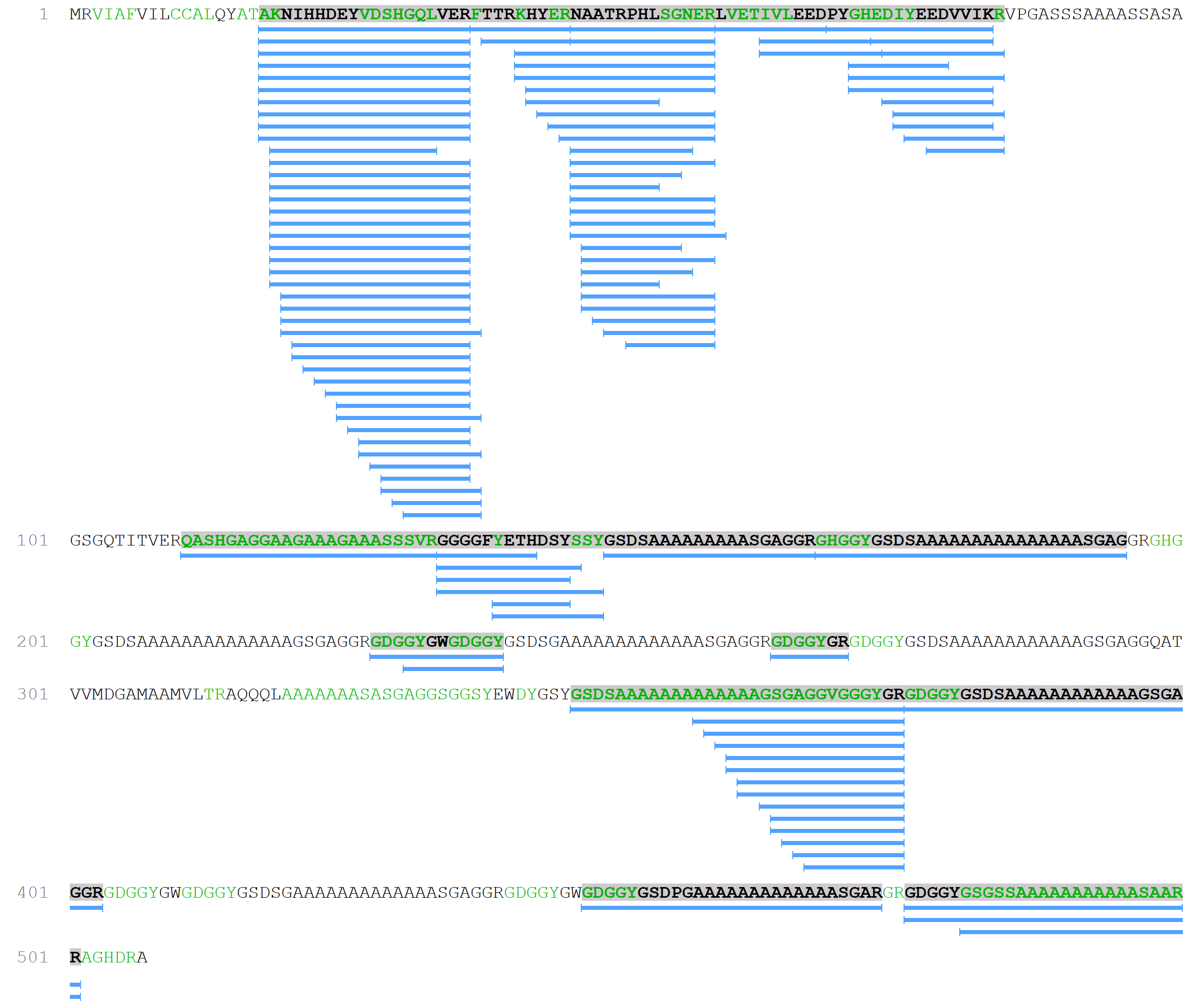


Fig. S16. Sequence coverage obtained from nanoLC-MS/MS analysis of the tryptic-chymotryptic digestion of the *A. mylitta* sample matched to the Q8ISB3 sequence shows 56% sequence coverage. The alternating black and green blocks indicate theoretical tryptic peptides. Residues in bold indicate the detected peptides; blue bars indicate the peptides coverage, and the vertical bars mark different cleavage sites.


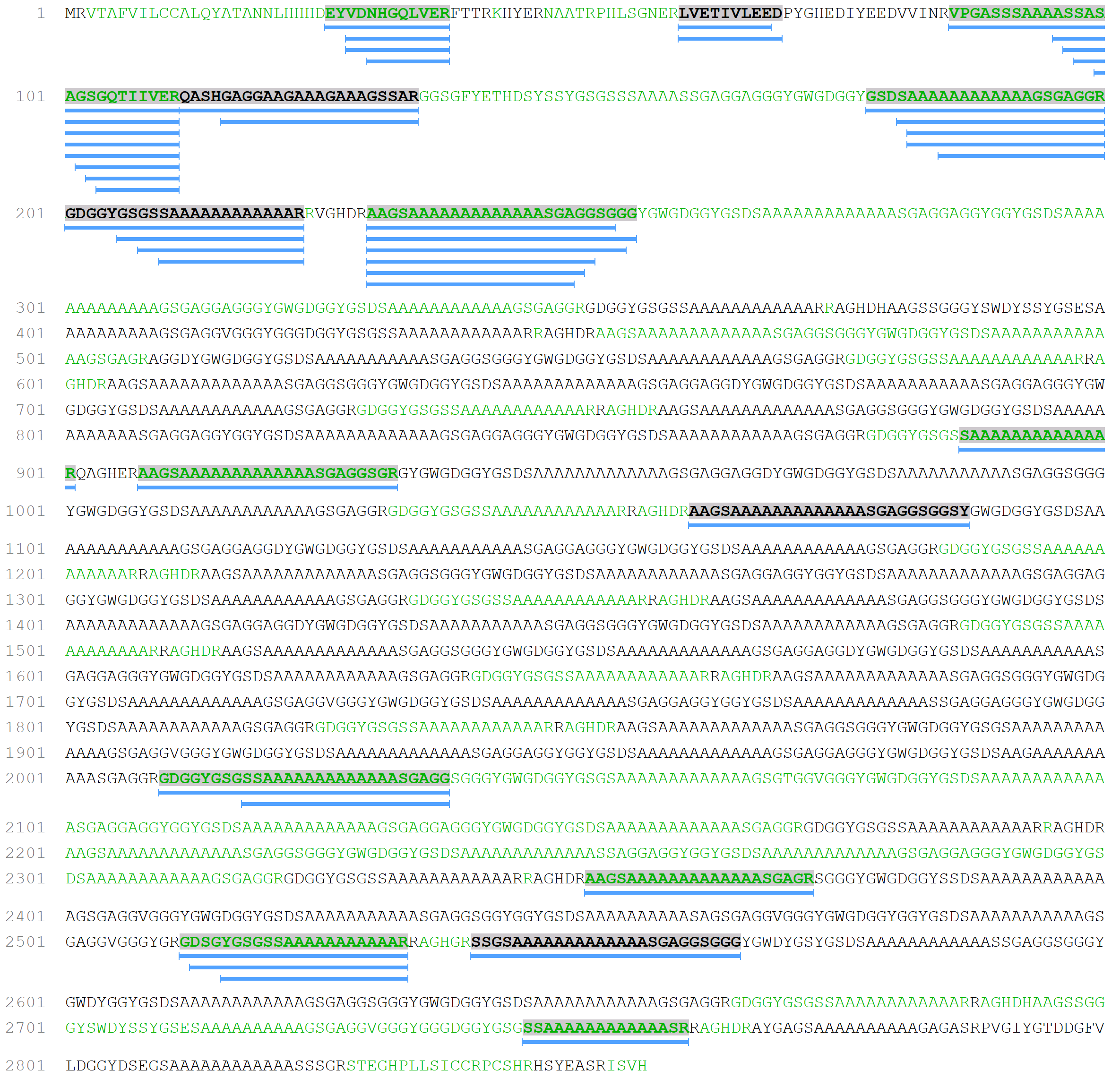


Fig. S17. Sequence coverage obtained from nanoLC-MS/MS analysis of the tryptic digestion of the *A. yamamai* sample matched to the E1CGA3 sequence shows 12% coverage. The alternating black and green blocks indicate theoretical tryptic peptides. Residues in bold indicate the detected peptides; blue bars indicate the peptides coverage, and the vertical bars mark different cleavage sites.


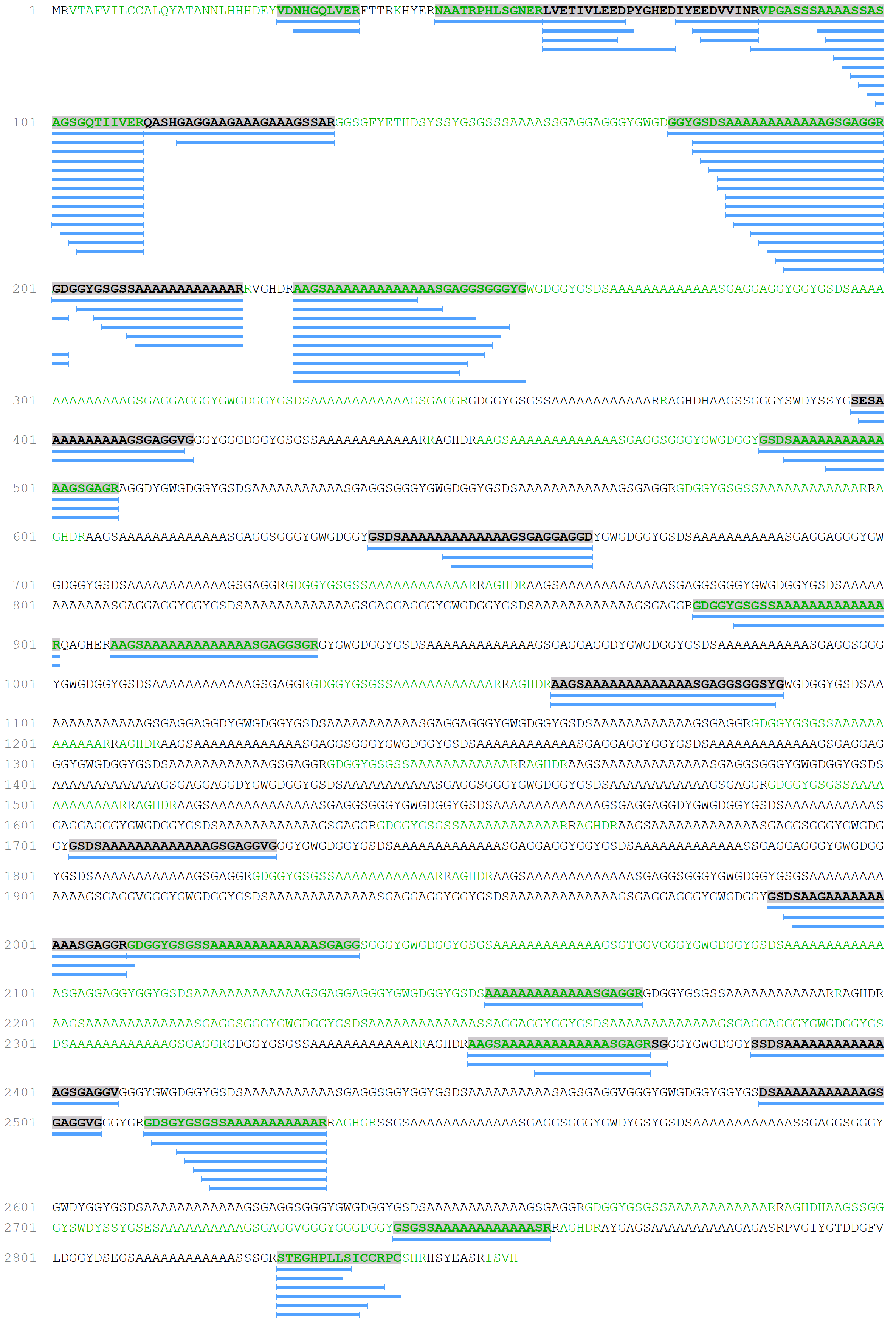


Fig. S18. Sequence coverage obtained from nanoLC-MS/MS analysis of the FA-tryptic digestion of the *A. yamamai* sample matched to the E1CGA3 sequence shows 19% sequence coverage. The alternating black and green blocks indicate theoretical tryptic peptides. Residues in bold indicate the detected peptides; blue bars indicate the peptides coverage, and the vertical bars mark different cleavage sites.


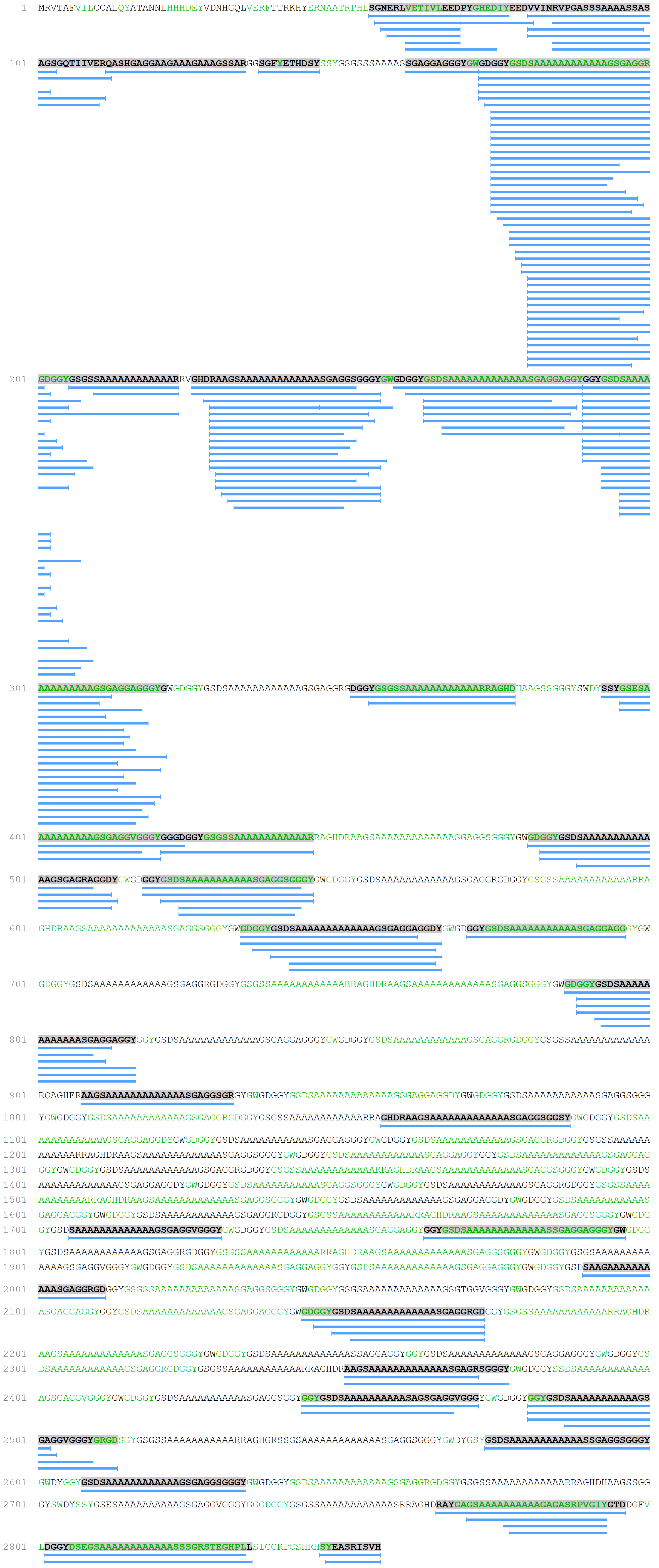


Fig. S19. Sequence coverage obtained from nanoLC-MS/MS analysis of the chymotryptic digestion of the *A. yamamai* sample matched to the E1CGA3 sequence shows 30% sequence coverage. The alternating black and green blocks indicate theoretical tryptic peptides. Residues in bold indicate the detected peptides; blue bars indicate the peptides coverage, and the vertical bars mark different cleavage sites.


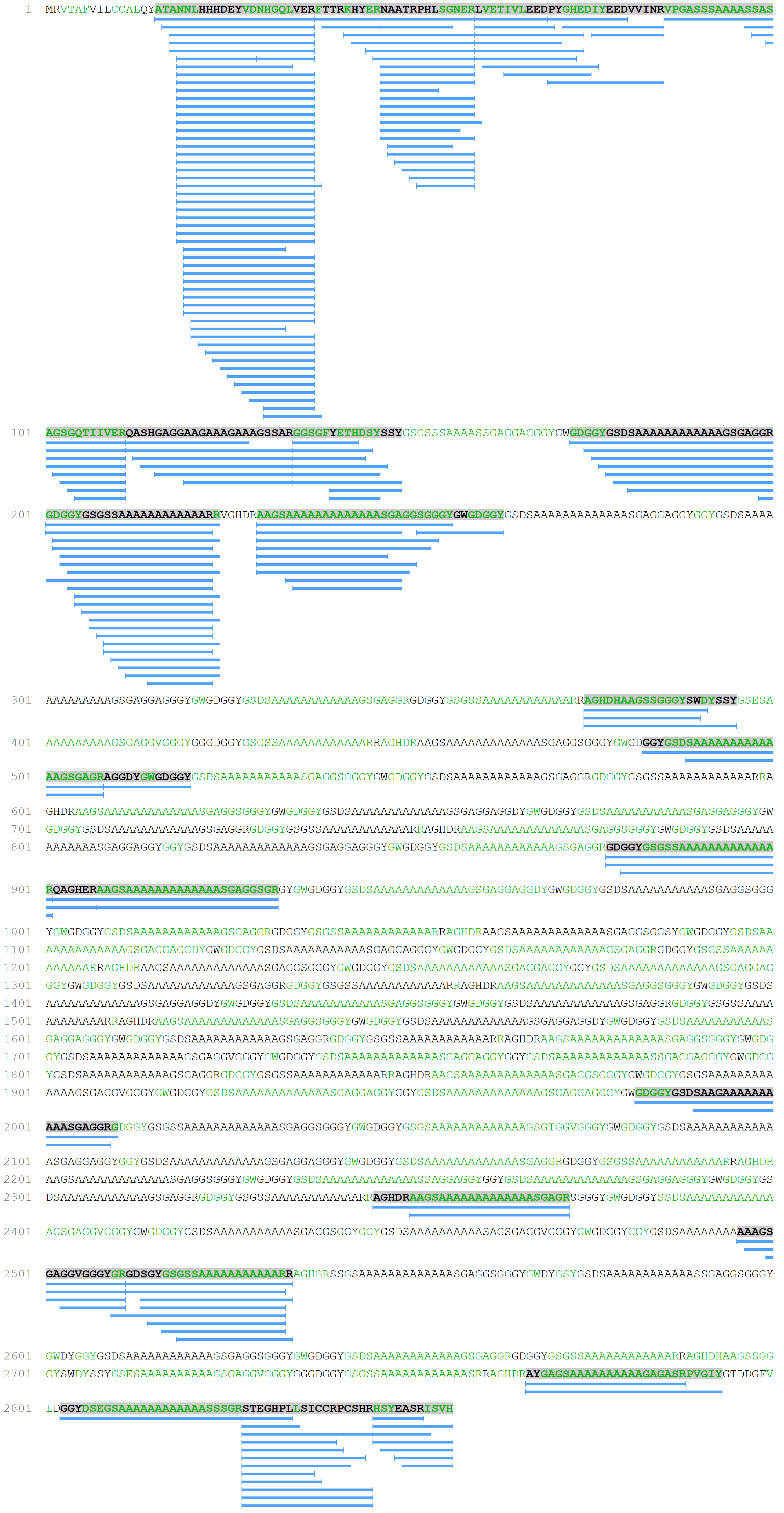


Fig. S20. Sequence coverage obtained from nanoLC-MS/MS analysis of the tryptic-chymotryptic digestion of the *A. yamamai* sample matched to the E1CGA3 sequence shows 18% sequence coverage. The alternating black and green blocks indicate theoretical tryptic peptides. Residues in bold indicate the detected peptides; blue bars indicate the peptides coverage, and the vertical bars mark different cleavage sites.


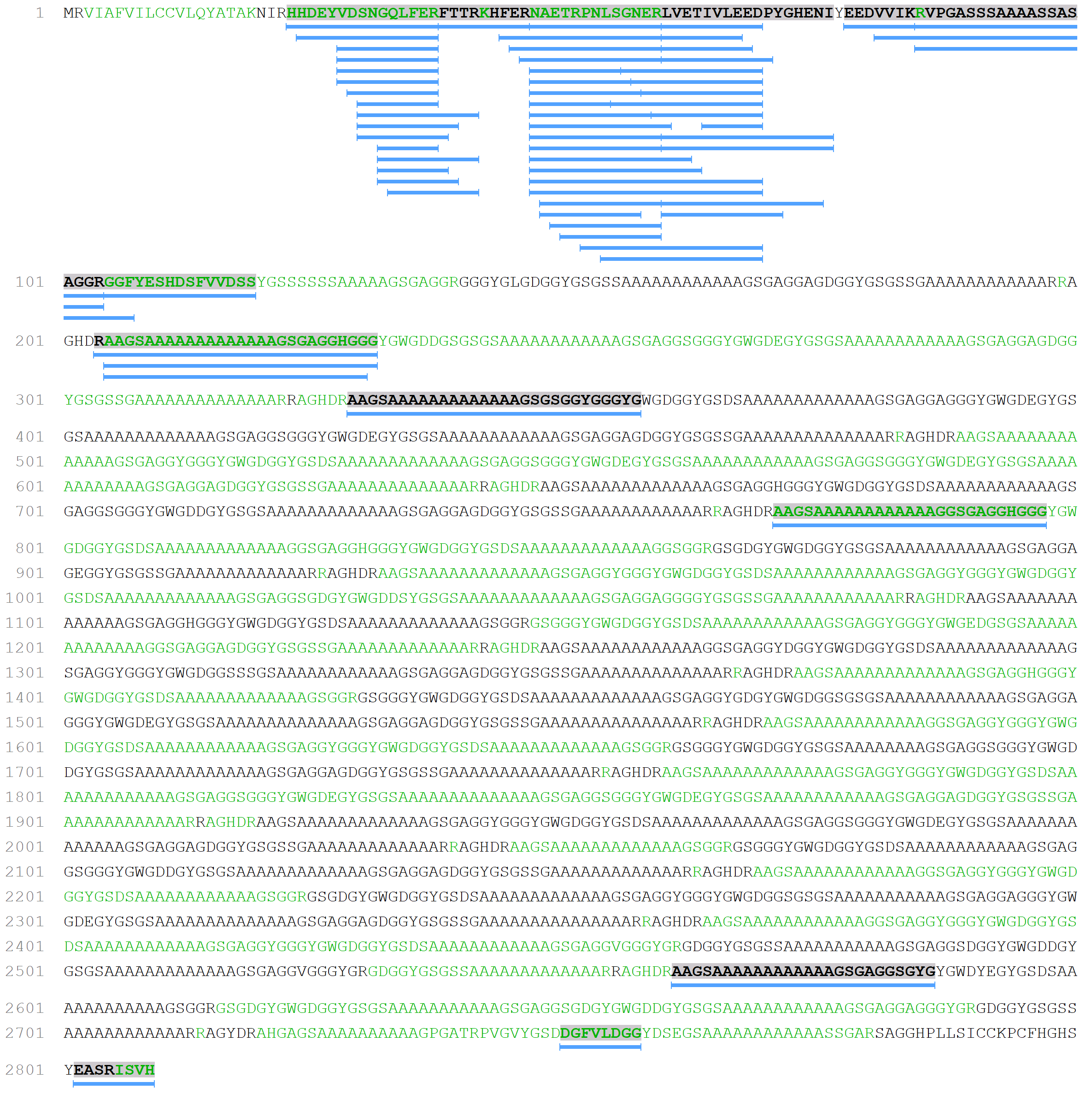


Fig. S21. Sequence coverage obtained from nanoLC-MS/MS analysis of the tryptic digestion of the *A. assamensis* sample matched to the A0A0K0KR73 sequence shows 8% sequence coverage. The alternating black and green blocks indicate theoretical tryptic peptides. Residues in bold indicate the detected peptides; blue bars indicate the peptides coverage, and the vertical bars mark different cleavage sites.


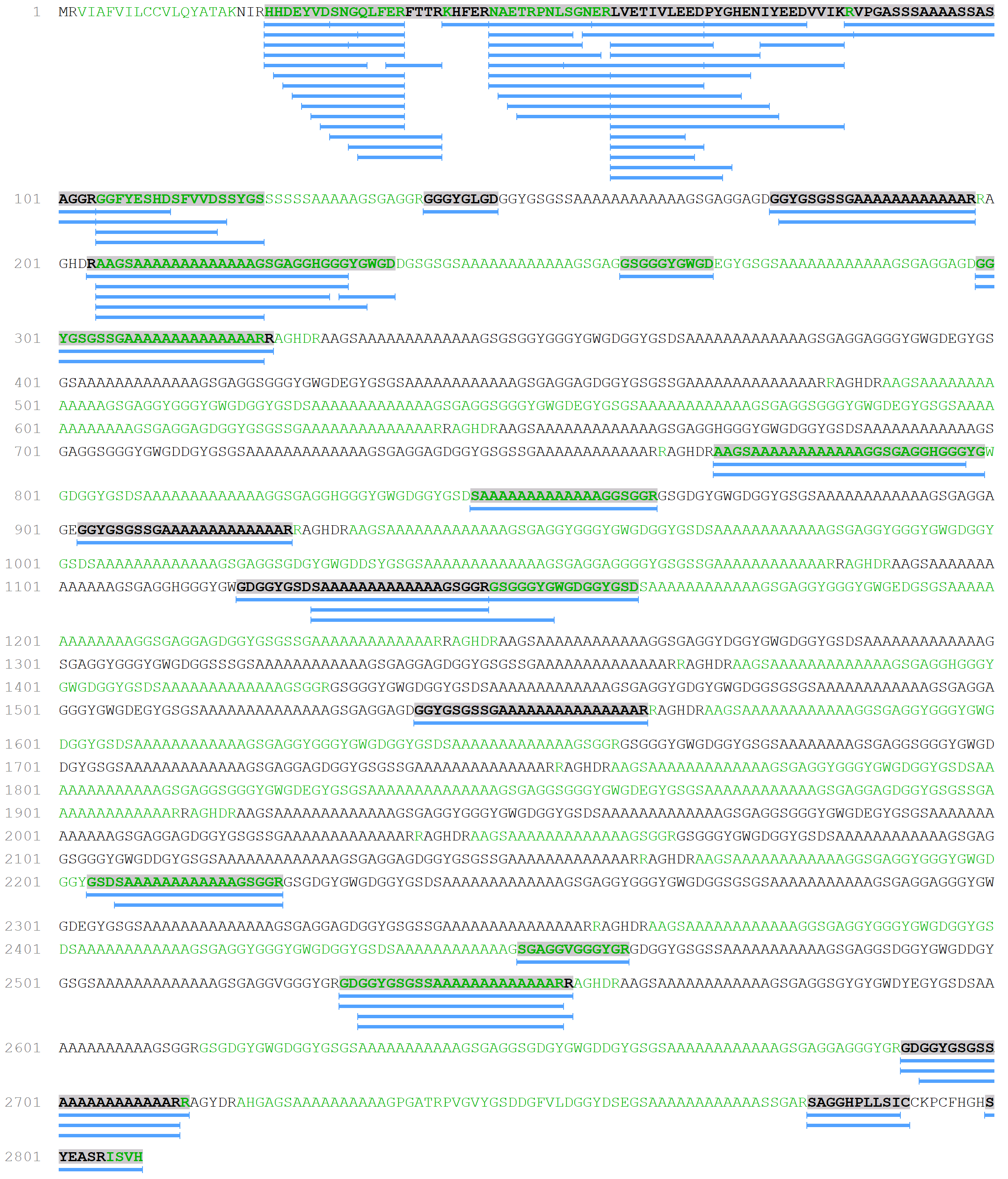


Fig. S22. Sequence coverage obtained from nanoLC-MS/MS analysis of the FA-tryptic digestion of the *A. assamensis* sample matched to the A0A0K0KR73 sequence shows 16% sequence coverage. The alternating black and green blocks indicate theoretical tryptic peptides. Residues in bold indicate the detected peptides; blue bars indicate the peptides coverage, and the vertical bars mark different cleavage sites.


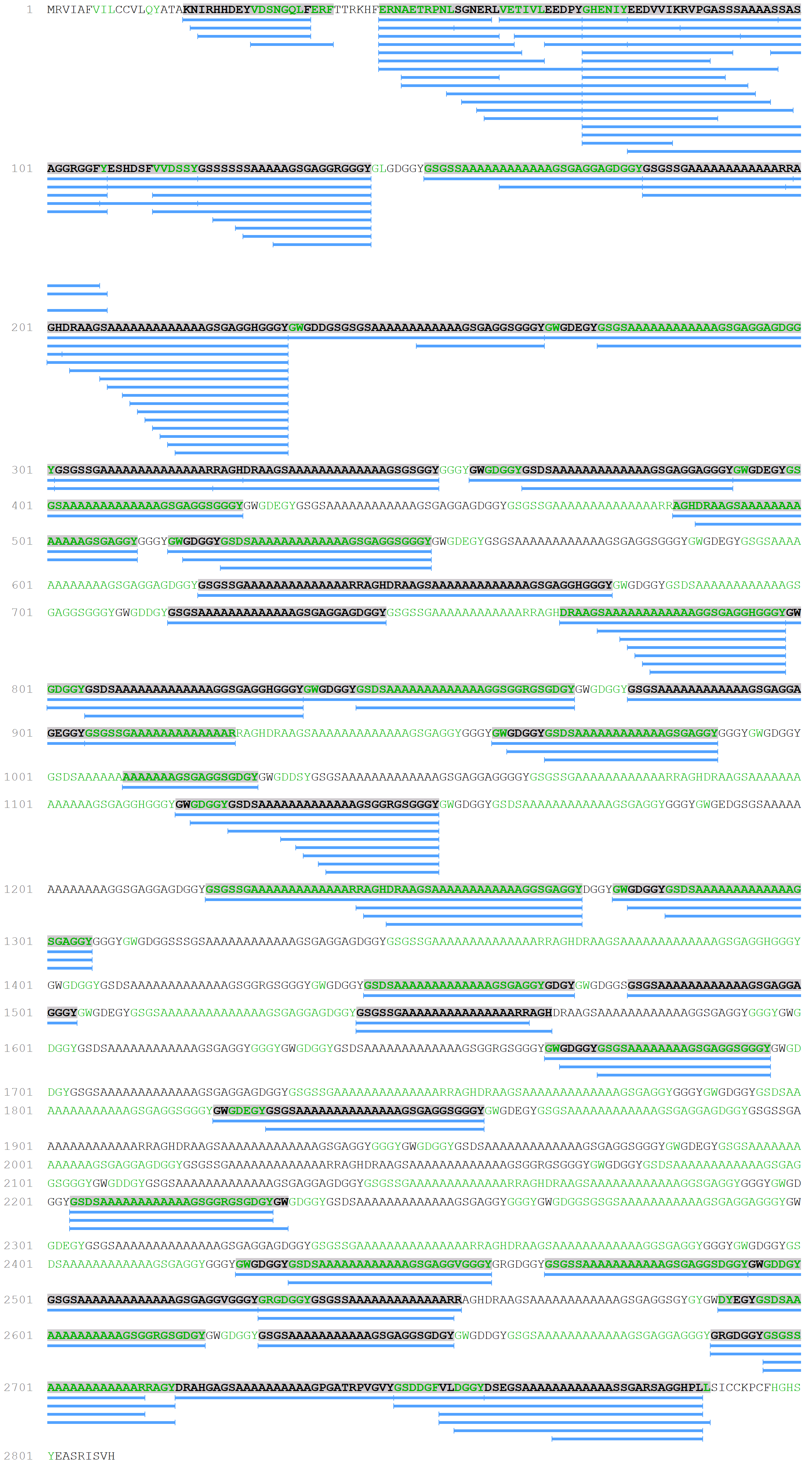


Fig. S23. Sequence coverage obtained from nanoLC-MS/MS analysis of the chymotryptic digestion of the *A. assamensis* sample matched to the A0A0K0KR73 sequence shows 47% sequence coverage. The alternating black and green blocks indicate theoretical tryptic peptides. Residues in bold indicate the detected peptides; blue bars indicate the peptides coverage, and the vertical bars mark different cleavage sites.


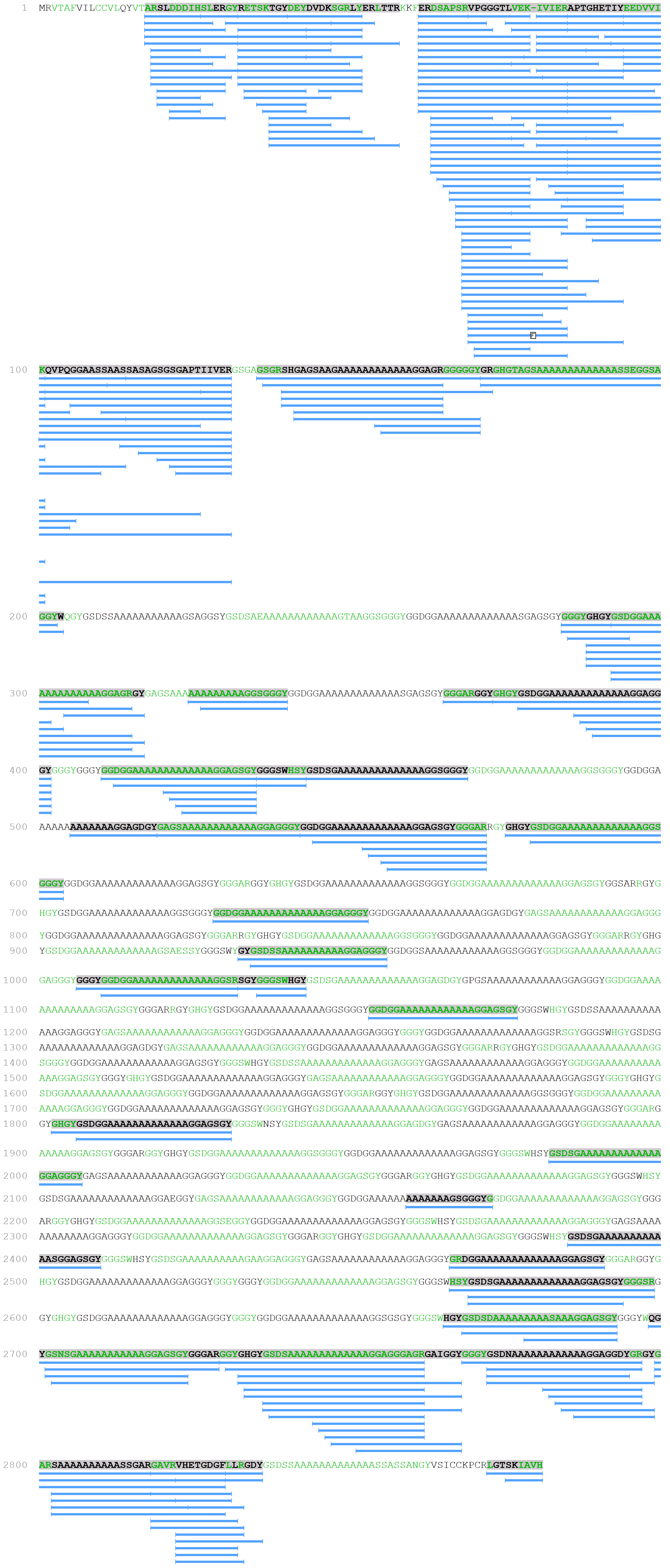


Fig. S24. Sequence coverage obtained from nanoLC-MS/MS analysis of the tryptic-chymotryptic digestion of the *A. assamensis* sample matched to the A0A0K0KR73 sequence shows 51% sequence coverage. The alternating black and green blocks indicate theoretical tryptic peptides. Residues in bold indicate the detected peptides; blue bars indicate the peptides coverage, and the vertical bars mark different cleavage sites.


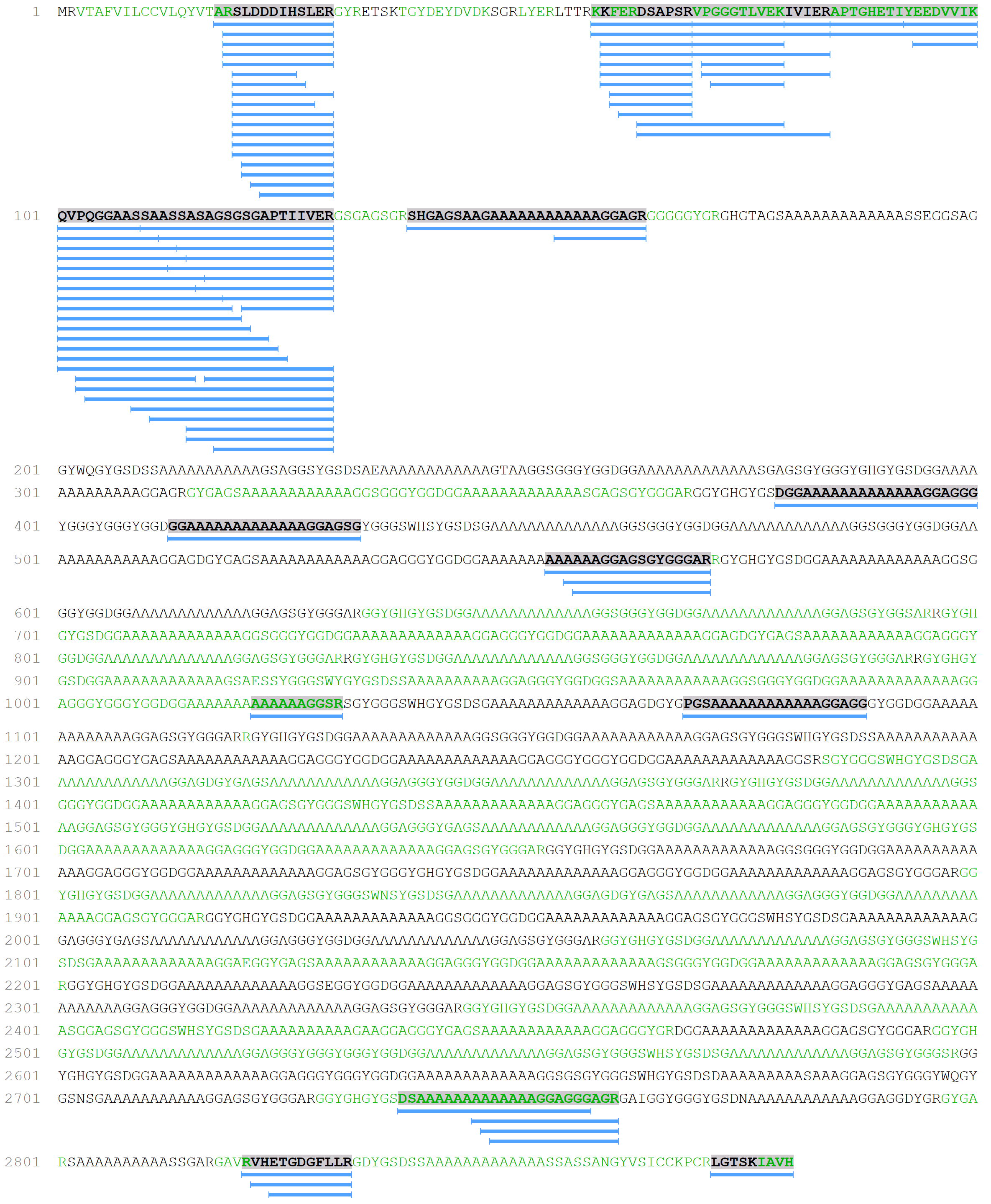


Fig. S25. Sequence coverage obtained from nanoLC-MS/MS analysis of the tryptic digestion of the *S. ricini* sample matched to the A0A0D5ZYI3 sequence shows 9% sequence coverage. The alternating black and green blocks indicate theoretical tryptic peptides. Residues in bold indicate the detected peptides; blue bars indicate the peptides coverage, and the vertical bars mark different cleavage sites.


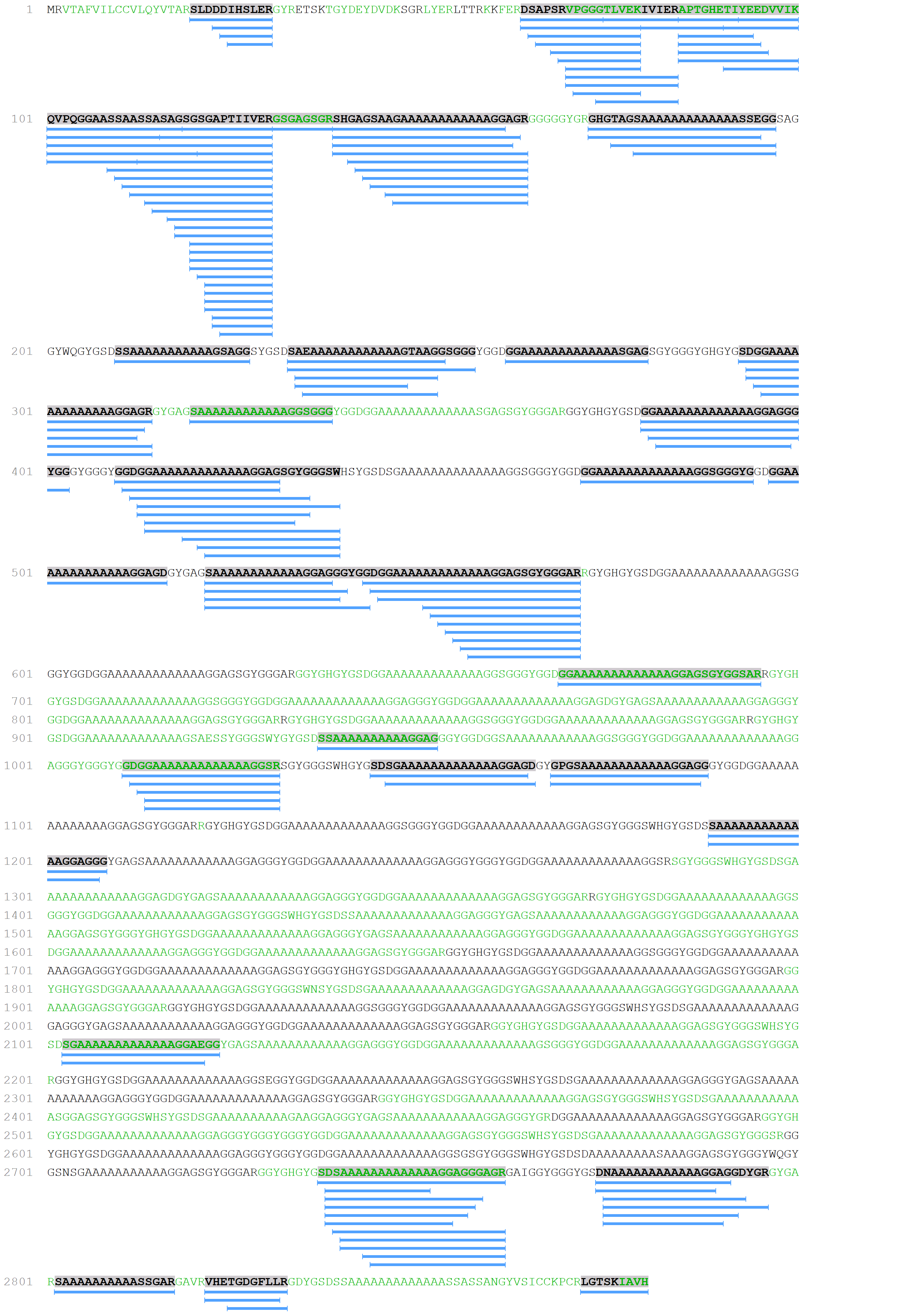


Fig. S26. Sequence coverage obtained from nanoLC-MS/MS analysis of the FA-tryptic digestion of the *S. ricini* sample matched to the A0A0D5ZYI3 sequence shows 21% sequence coverage. The alternating black and green blocks indicate theoretical tryptic peptides. Residues in bold indicate the detected peptides; blue bars indicate the peptides coverage, and the vertical bars mark different cleavage sites.


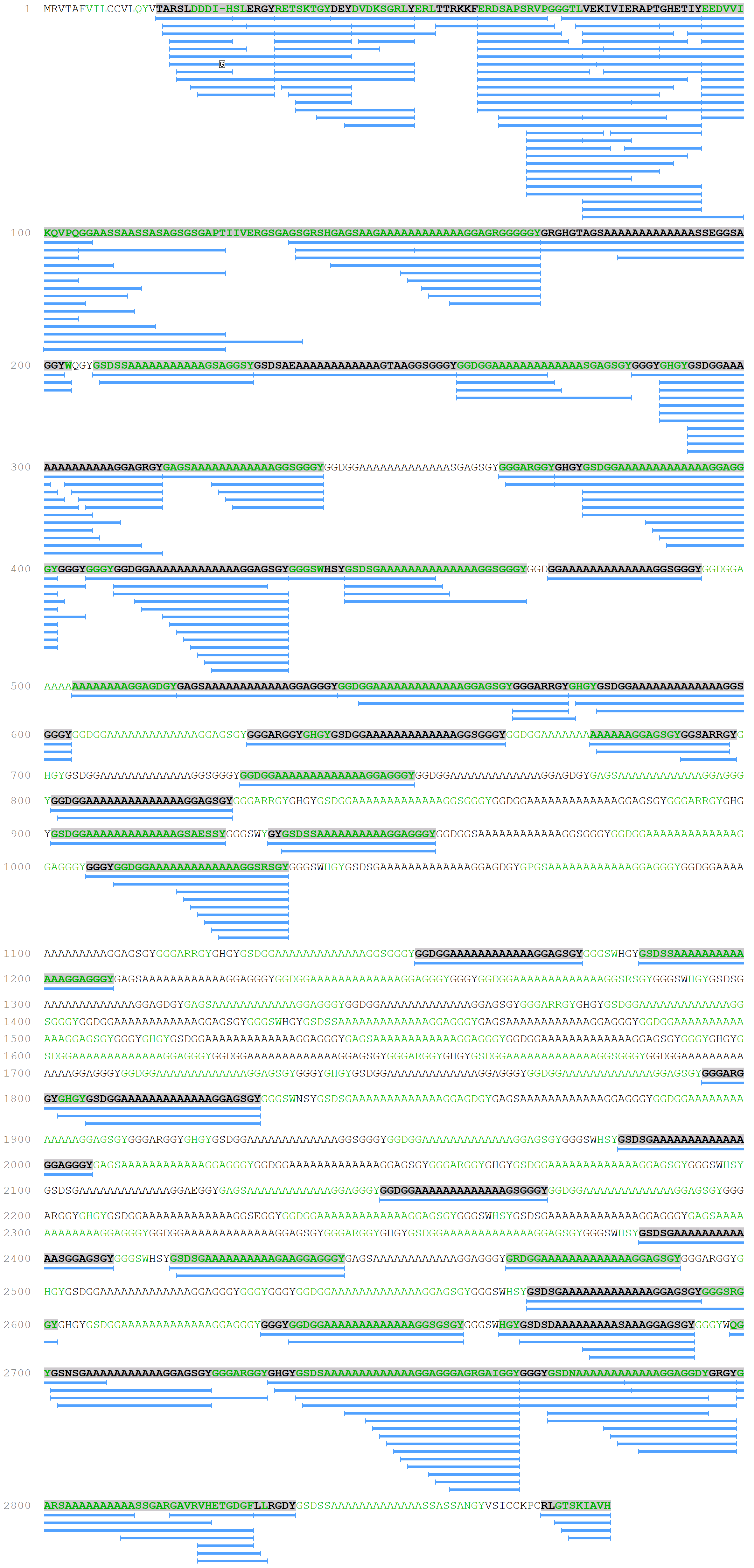


Fig. S27. Sequence coverage obtained from nanoLC-MS/MS analysis of the chymotryptic digestion of the *S. ricini* sample matched to the A0A0D5ZYI3 sequence shows 41% sequence coverage. The alternating black and green blocks indicate theoretical tryptic peptides. Residues in bold indicate the detected peptides; blue bars indicate the peptides coverage, and the vertical bars mark different cleavage sites.


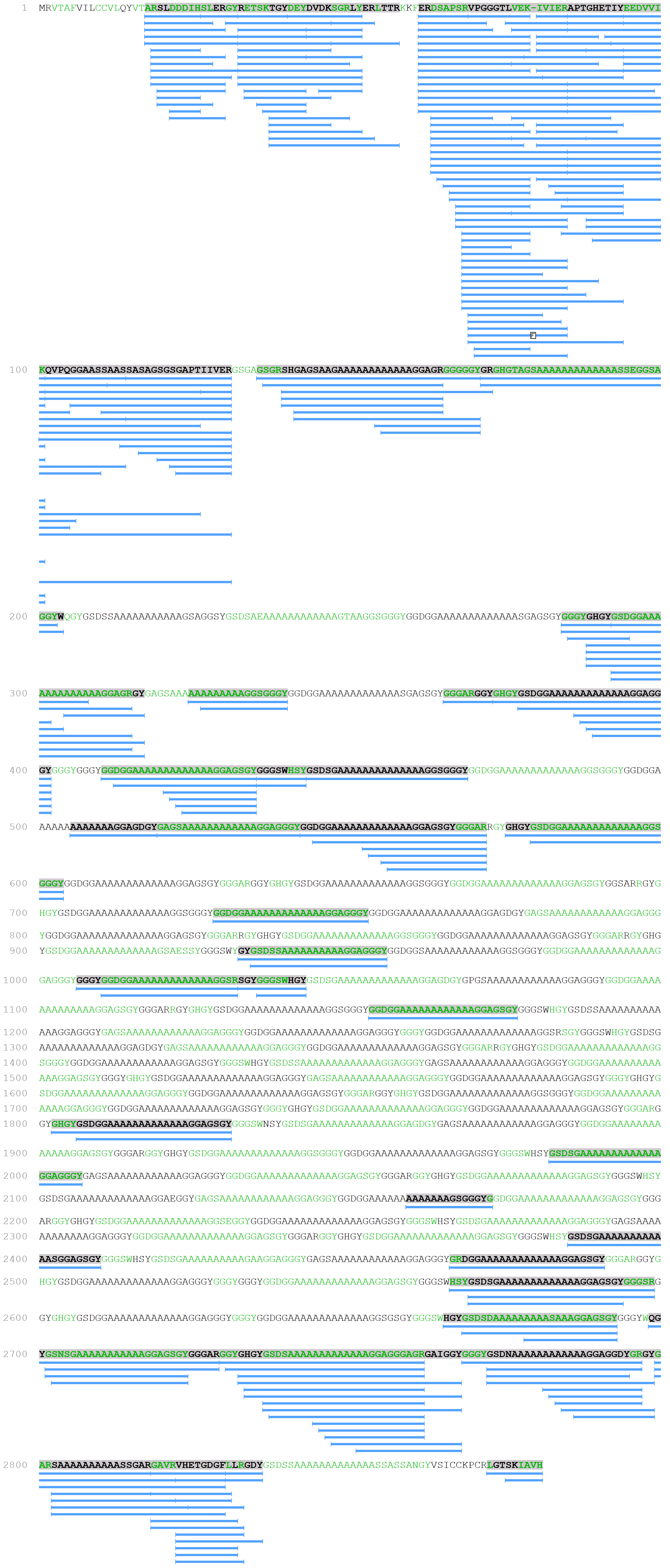


Fig. S28. Sequence coverage obtained from nanoLC-MS/MS analysis of the tryptic-chymotryptic digestion of the *S. ricini* sample matched to the A0A0D5ZYI3 sequence shows 30% sequence coverage. The alternating black and green blocks indicate theoretical tryptic peptides. Residues in bold indicate the detected peptides; blue bars indicate the peptides coverage, and the vertical bars mark different cleavage sites.


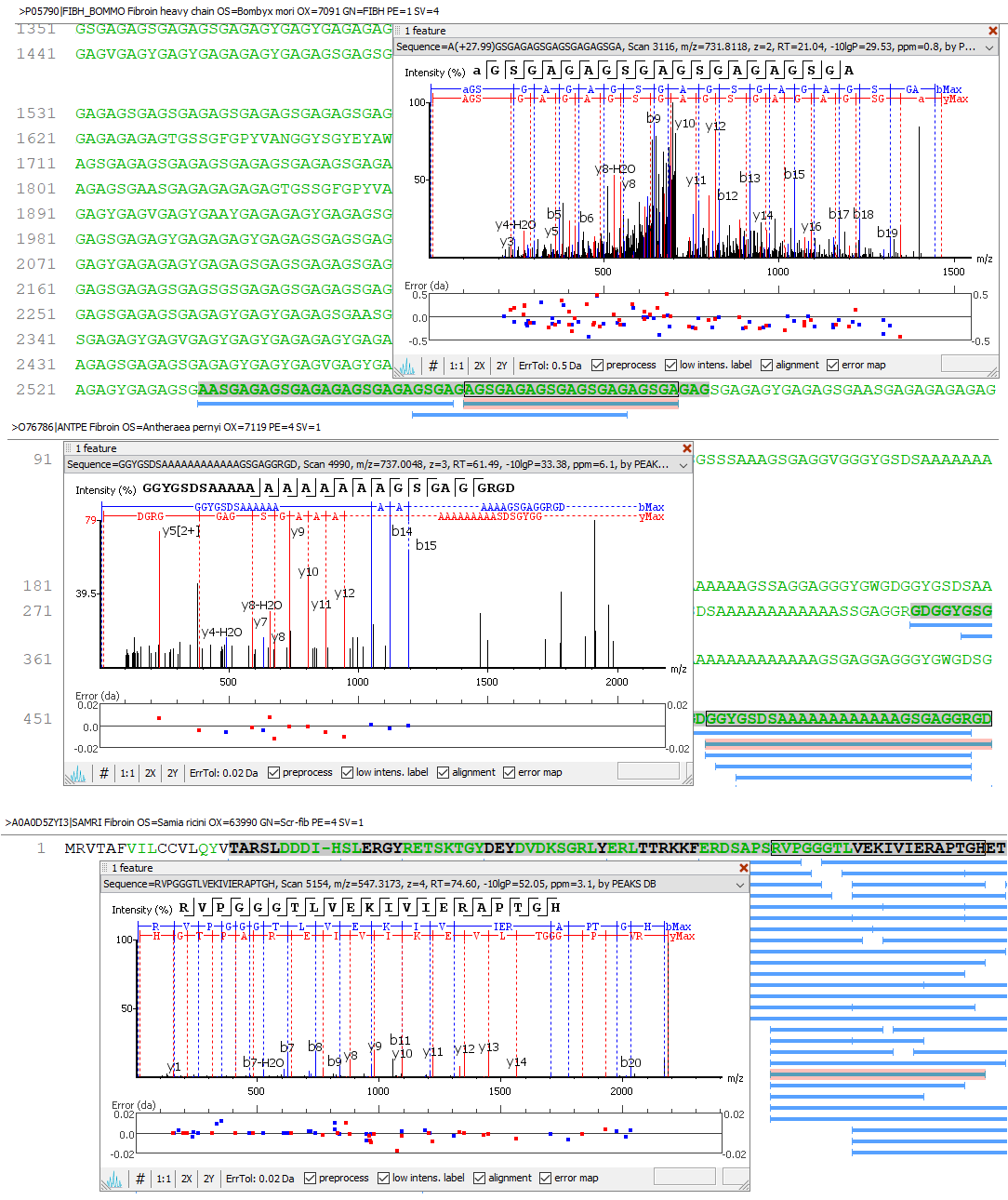


Fig. S29. Non-enzymatic cleavages found in experimental samples

*B. mori* (Trypsin-FA), (G)AGSGAGAGSGAGSGAGAGSGA(G), -10lgP=29.53, 2559-2579; *A. pernyi* (Chymotrypsin-trypsin), (D)GGYGSDSA_12_GSGAGGRGD(G), -10lgP=33.38, 513-540; *S. ricini* (Chymotrypsin), (S)RVPGGGTLVEKIVIERAPTGH(E), -10lgP=52.05 (69-89).

Table S1. The characteristic hexapeptide GAGAG(X) and subsequences unique to *B. mori* heavy chain protein (UniProt accession: P05790)

| GAGAG-**S** | (432) | GAGAG**S**-**G** | (431) | GAGAG**SG**-**A** | (423) |
| --- | --- | --- | --- | --- | --- |
|  |  |  |  | GAGAG**SG**-**T** | (4) |
|  |  |  |  | GAGAG**SG**-**S** | (3) |
|  |  |  |  | GAGAG**SG**-**V** | (1) |
|  |  | GAGAG**S**-**A** | (1) |  |  |
| GAGAG-**A** | (151) | GAGAG**A**-**G** | (150) | GAGAGAG-**Y** | (63) |
|  |  |  |  | GAGAGAG-**A** | (60) |
|  |  |  |  | GAGAGAG-**S** | (15) |
|  |  |  |  | GAGAGAG-**T** | (12) |
|  |  | GAGAG**A**-**A** | (1) |  |  |
| GAGAG-**Y** | (120) | GAGAG**Y**-**G** | (120) | GAGAG**YG**-**A** | (113) |
|  |  |  |  | GAGAG**YG**-**V** | (3) |
|  |  |  |  | GAGAG**YG**-**T** | (2) |
|  |  |  |  | GAGAG**YG**-**G** | (1) |
|  |  |  |  | GAGAG**YG**-**Q** | (1) |
| GAGAG-**T** | (14) | GAGAG**T**-**G** | (14) | GAGAG**TG**-**S** | (11) |
|  |  |  |  | GAGAG**TG**-**A** | (3) |
| GAGAG-**V** | (11) | GAGAG**V**-**G** | (11) | GAGAG**VG**-**YGA** | (11) |
| GAGAG-**G** | (1) |  | | | |

Table S2. Homology (%) between the silk fibroin sequences through Pairwise alignment

* incomplete sequences

|  | ***B. mandarina (HC)**** | ***A. pernyi*** | ***A. mylitta **** | ***A. yamamai*** | ***A. assamensis*** | ***S. ricini*** |
| --- | --- | --- | --- | --- | --- | --- |
| ***B. mori* (HC)** | 97.77% | 40.91% | 38.96% | 41.66% | 42.89% | 42.54% |
| ***B. mandarina* (HC)*** |  | 33.15% | 32.02% | 34.07% | 32.96% | 36.46% |
| ***A. pernyi*** |  |  | 75% | 77.79% | 73.43% | 60.77% |
| ***A. mylitta**** |  |  |  | 68.94% | 72.67% | 57.44% |
| ***A. yamamai*** |  |  |  |  | 77.33% | 61.85% |
| ***A. assamensis*** |  |  |  |  |  | 60.77% |

Table S3. Amino acid compositions of *B. mori, A. pernyi, A. mylitta, A. yamamai, A. assamensis*, and *S. ricini* silk fibroins (exclude signal peptides)

Ala (A), Gly (G), Ser (S) (marked in red) are the most abundant amino acids in fibroins. The GRAVY (Grand average of hydropathicity) values show the hydrophobicity of each protein (*9*).

|  | ***B. mori* (HC)**  #22-5263 MW 389203.41 Theo. pl: 4.32 | | ***A. pernyi***  **#**19-2639 MW 214091.01 Theo. pl: 4.52 | | ***A. mylitta***  #19-507 MW 43433.37 Theo. pl: 4.98 | | ***A. yamamai***  #19-2856 MW 232619.64 Theo. pl: 4.48 | | ***A. assamensis***  #19-2809 MW 227498.35 Theo. pl: 4.50 | | ***S. ricini***  #19-2880 MW 225349.42 Theo. pl: 4.82 | |
| --- | --- | --- | --- | --- | --- | --- | --- | --- | --- | --- | --- | --- |
| Amino acid | #residue | Mol (%) | #residue | Mol (%) | #residue | Mol (%) | #residue | Mol (%) | #residue | Mol (%) | #residue | Mol (%) |
| **Ala (A)** | 1590 | **30.3** | 1133 | **43.2** | 171 | **35.0** | 1221 | **43.0** | 1190 | **42.6** | 1305 | **45.6** |
| **Arg (R)** | 13 | 0.3 | 70 | 2.7 | 22 | 4.5 | 80 | 2.8 | 73 | 2.6 | 47 | 1.6 |
| **Asn (N)** | 19 | 0.4 | 5 | 0.2 | 3 | 0.6 | 6 | 0.2 | 6 | 0.2 | 4 | 0.1 |
| **Asp (D)** | 25 | 0.5 | 118 | 4.5 | 28 | 5.7 | 141 | 5.0 | 127 | 4.6 | 93 | 3.2 |
| **Cys (C)** | 3 | 0.1 | 3 | 0.1 | - | - | 3 | 0.1 | 3 | 0.1 | 3 | 0.1 |
| **Gln (Q)** | 9 | 0.2 | 4 | 0.2 | 7 | 1.4 | 4 | 0.1 | 1 | 0.0 | 4 | 0.1 |
| **Glu (E)** | 30 | 0.6 | 17 | 0.6 | 13 | 2.7 | 18 | 0.6 | 27 | 1.0 | 17 | 0.6 |
| **Gly (G)** | 2415 | **45.9** | 720 | **27.5** | 111 | **22.7** | 778 | **27.4** | 811 | **29.1** | 914 | **31.9** |
| **His (H)** | 5 | 0.1 | 25 | 1.0 | 11 | 2.2 | 32 | 1.1 | 32 | 1.1 | 39 | 1.4 |
| **Lle (I)** | 12 | 0.2 | 9 | 0.3 | 5 | 1.0 | 8 | 0.3 | 6 | 0.2 | 10 | 0.3 |
| **Leu (L)** | 5 | 0.1 | 8 | 0.3 | 6 | 1.2 | 8 | 0.3 | 8 | 0.3 | 8 | 0.3 |
| **Lys (K)** | 11 | 0.2 | 3 | 0.1 | 3 | 0.6 | 1 | 0.0 | 4 | 0.1 | 8 | 0.3 |
| **Met (M)** | 3 | 0.1 | - | - | 3 | 0.6 | - |  | - | - | - | - |
| **Phe (F)** | 28 | 0.6 | 5 | 0.2 | 2 | 0.4 | 3 | 0.1 | 7 | 0.3 | 2 | 0.1 |
| **Pro (P)** | 14 | 0.3 | 5 | 0.2 | 4 | 0.8 | 6 | 0.2 | 7 | 0.3 | 7 | 0.2 |
| **Ser (S)** | 635 | 12.1 | 297 | **11.3** | 51 | **10.4** | 315 | **11.1** | 287 | **10.3** | 194 | **6.8** |
| **Thr (T)** | 45 | 0.9 | 8 | 0.3 | 9 | 1.8 | 9 | 0.3 | 5 | 0.2 | 12 | 0.4 |
| **Trp (W)** | 11 | 0.2 | 34 | 1.3 | 4 | 0.8 | 44 | 1.6 | 46 | 1.6 | 16 | 0.6 |
| **Tyr (Y)** | 275 | 5.3 | 138 | 5.3 | 23 | 4.7 | 141 | 5.0 | 137 | 4.9 | 167 | 5.8 |
| **Val (V)** | 94 | 1.8 | 19 | 0.7 | 13 | 2.7 | 20 | 0.7 | 14 | 0.5 | 12 | 0.4 |
| Instability index | | -20.90 |  | 19.69 |  | 51.35 |  | 20.15 |  | 16.51 |  | 16.01 |
| GRAVY |  | 0.213 |  | 0.210 |  | -0.075 |  | 0.178 |  | 0.175 |  | 0.327 |
|  |  |  |  |  |  |  |  |  |  |  |  |  |

Table S4. List of unique peptides detected from *B. mori* HC digested with trypsin, FA-trypsin, chymotrypsin, and chymotrypsin-trypsin at False Discovery Rate ≤1%

PEAKS DB match is the exact match to the protein sequence in given in the database, while PEAKS PTM is an integrated search of PEAKS database and de novo sequencing results including any post transitional modification; SPIDER is an algorithm to detect peptide mutations and perform cross-species homology search.

| **TRYPSIN** |  |  |  |  |  |  |  |  |  |  |  |
| --- | --- | --- | --- | --- | --- | --- | --- | --- | --- | --- | --- |
| **Peptide** | **-10lgP** | **Mass** | **Length** | **ppm** | **m/z** | **z** | **RT** | **Start** | **End** | **PTM** | **Found By** |
| R.GYGQ(+.98)GAGSAASSVSSASSR.S | 47.44 | 1686.745 | 19 | 0.1 | 844.3796 | 2 | 24.2 | 5213 | 5231 | Deamidation (NQ) | PEAKS DB |
| R.GYGQGAGSAASSVSSASSR.S | 46.38 | 1685.761 | 19 | 0.2 | 843.8877 | 2 | 23.9 | 5213 | 5231 |  | PEAKS DB |
| R.DASGAVIEEQITTK.K | 45.85 | 1460.736 | 14 | 0.6 | 731.3756 | 2 | 35.6 | 49 | 62 |  | PEAKS DB |
| D.ASGAVIEEQITTK.K | 45.36 | 1345.709 | 13 | -0.2 | 673.8616 | 2 | 28.5 | 50 | 62 |  | PEAKS DB |
| R.DASGAVIEEQITTKK.M | 45.16 | 1588.831 | 15 | 0.9 | 795.4234 | 2 | 30.8 | 49 | 63 |  | PEAKS DB |
| Y.GQGAGSAASSVSSASSR.S | 44.66 | 1465.676 | 17 | 1.6 | 733.8463 | 2 | 19.7 | 5215 | 5231 |  | PEAKS DB |
| A.SGAGAGAGAGAGTGSSGFGPYVAN(+.98)GGYSR.S | 44.34 | 2461.089 | 29 | -0.3 | 821.3701 | 3 | 34.8 | 661 | 689 | Deamidation (NQ) | PEAKS DB |
| R.REGYEYAWSSK.S | 42.04 | 1374.62 | 11 | -1.2 | 688.3167 | 2 | 25.7 | 5151 | 5161 |  | PEAKS DB |
| Q.GAGSAASSVSSASSR.S | 40.93 | 1280.596 | 15 | 0.7 | 641.3055 | 2 | 19 | 5217 | 5231 |  | PEAKS DB |
| G.SGAGAGSGAGAGGSVSYGAGR.G | 40.7 | 1652.75 | 21 | 1.2 | 827.3834 | 2 | 22.1 | 5192 | 5212 |  | PEAKS DB |
| R.ALPC(+57.02)VNC(+57.02) | 40.16 | 832.3572 | 7 | 0.1 | 417.1859 | 2 | 29.8 | 5257 | 5263 | Carbamidomethylation | PEAKS DB |
| R.EGYEYAWSSK.S | 39.73 | 1218.519 | 10 | 0.6 | 610.2673 | 2 | 30.9 | 5152 | 5161 |  | PEAKS DB |
| K.SDFETGSGAASGAG.A | 39.66 | 1212.489 | 14 | 0.3 | 607.2521 | 2 | 25.8 | 5162 | 5175 |  | PEAKS DB |
| A.SGAVIEEQITTK.K | 38.57 | 1274.672 | 12 | -0.1 | 638.3431 | 2 | 27.7 | 51 | 62 |  | PEAKS DB |
| A.SGAGAGAGAGTGSSGFGPYVAN(+.98)GGYSR.R | 38.42 | 2333.031 | 27 | -0.8 | 778.6836 | 3 | 34.6 | 5124 | 5150 | Deamidation (NQ) | PEAKS DB |
| R.GYGQGAGSAASSVSSA.S | 38.11 | 1355.595 | 16 | 0 | 678.8049 | 2 | 25.8 | 5213 | 5228 |  | PEAKS DB |
| K.SDFETGSGAASGAGAGAGSG.A | 37.94 | 1612.66 | 20 | 0.4 | 807.3376 | 2 | 27.2 | 5162 | 5181 |  | PEAKS DB |
| R.GYGQGAGSAASSVSSAS.S | 37.65 | 1442.627 | 17 | -0.1 | 722.3209 | 2 | 25.6 | 5213 | 5229 |  | PEAKS DB |
| S.SGFGPYVAN(+.98)GGYSR.S | 37.44 | 1431.642 | 14 | -0.3 | 716.828 | 2 | 34.3 | 676 | 689 | Deamidation (NQ) | PEAKS DB |
| G.SAASSVSSASSR.S | 37.4 | 1095.516 | 12 | 0.2 | 548.7652 | 2 | 17.6 | 5220 | 5231 |  | PEAKS DB |
| S.GAGAGGSVSYGAGR.G | 36.97 | 1165.548 | 14 | -0.6 | 583.7807 | 2 | 20.5 | 5199 | 5212 |  | PEAKS DB |
| G.SGAGAGGSVSYGAGR.G | 36.62 | 1252.58 | 15 | 0.1 | 627.2971 | 2 | 20.7 | 5198 | 5212 |  | PEAKS DB |
| F.GPYVAN(+.98)GGYSR.S | 36.24 | 1140.52 | 11 | 0.3 | 571.2674 | 2 | 24.1 | 679 | 689 | Deamidation (NQ) | PEAKS DB |
| R.GYGQGAGSAASSVS.S | 36.11 | 1197.526 | 14 | 0.4 | 599.7706 | 2 | 24 | 5213 | 5226 |  | PEAKS DB |
| A.GSGAGAGGSVSYGAGR.G | 35.55 | 1309.601 | 16 | 0.4 | 655.808 | 2 | 20.8 | 5197 | 5212 |  | PEAKS DB |
| G.AGSGAGAGGSVSYGAGR.G | 35.22 | 1380.638 | 17 | 0.2 | 691.3265 | 2 | 21.1 | 5196 | 5212 |  | PEAKS DB |
| G.SGAGAGSGAGAGSGAGAGSGAGAGGSVSYGAGR.G | 35.07 | 2453.092 | 33 | 0.6 | 818.705 | 3 | 24.2 | 5180 | 5212 |  | PEAKS DB |
| N.TTDEIIRDASGAVIEEQITTK.K | 34.88 | 2289.17 | 21 | 0.5 | 764.0643 | 3 | 41.4 | 42 | 62 |  | PEAKS DB |
| G.AGAGGSVSYGAGR.G | 34.76 | 1108.526 | 13 | -0.8 | 555.2699 | 2 | 20.2 | 5200 | 5212 |  | PEAKS DB |
| S.GAVIEEQITTK.K | 34.55 | 1187.64 | 11 | 0 | 594.8271 | 2 | 27.3 | 52 | 62 |  | PEAKS DB |
| R.KNC(+57.02)GIPR.R | 33.69 | 843.4385 | 7 | 0.1 | 422.7266 | 2 | 9.49 | 5242 | 5248 | Carbamidomethylation | PEAKS DB |
| A.GAGSGAGAGGSVSYGAGR.G | 33.52 | 1437.66 | 18 | 0.1 | 719.8372 | 2 | 21.3 | 5195 | 5212 |  | PEAKS DB |
| R.EGYEYAWSSK(+15.99).S | 33.38 | 1234.514 | 10 | 1.4 | 618.2652 | 2 | 23.6 | 5152 | 5161 | Oxidation or Hydroxylation | PEAKS DB |
| A.GSAASSVSSASSR.S | 32.65 | 1152.537 | 13 | 0.2 | 577.2759 | 2 | 17.7 | 5219 | 5231 |  | PEAKS DB |
| R.REGYEYAWSSK(+15.99).S | 32.54 | 1390.615 | 11 | -0.3 | 464.5456 | 3 | 23.8 | 5151 | 5161 | Oxidation or Hydroxylation | PEAKS DB |
| R.SYDYSR.R | 32.28 | 789.3293 | 6 | 0.5 | 395.6721 | 2 | 19.8 | 5232 | 5237 |  | PEAKS DB |
| K.N(+.98)C(+57.02)GIPR.R | 32.25 | 716.3276 | 6 | 0.3 | 359.1712 | 2 | 21.1 | 5243 | 5248 | Deamidation (NQ); Carbamidomethylation | PEAKS DB |
| K.NC(+57.02)GIPR.R | 32.24 | 715.3435 | 6 | 0.5 | 358.6792 | 2 | 18.6 | 5243 | 5248 | Carbamidomethylation | PEAKS DB |
| G.SGAGAGSGAGAGSGAGAGGSVSYGAGR.G | 31.72 | 2052.921 | 27 | 1.4 | 685.3152 | 3 | 23.3 | 5186 | 5212 |  | PEAKS DB |
| K.SDFETGSGAAS.G | 31.61 | 1027.409 | 11 | 0.5 | 514.7122 | 2 | 24.5 | 5162 | 5172 |  | PEAKS DB |
| S.GAGAGSGAGAGGSVSYGAGR.G | 31.33 | 1565.718 | 20 | 0.9 | 783.8671 | 2 | 22 | 5193 | 5212 |  | PEAKS DB |
| G.QGAGSAASSVSSASSR.S | 31.02 | 1408.654 | 16 | 2.1 | 705.3358 | 2 | 19.6 | 5216 | 5231 |  | PEAKS DB |
| G.AVIEEQITTK.K | 30.77 | 1130.618 | 10 | -1 | 566.3159 | 2 | 26.5 | 53 | 62 |  | PEAKS DB |
| G.AGAGTGSSGFGPYVAN(+.98)GGYSR.S | 30.39 | 1932.86 | 21 | -1.8 | 967.4355 | 2 | 34.3 | 669 | 689 | Deamidation (NQ) | PEAKS DB |
| G.PYVAN(+.98)GGYSR.S | 29.95 | 1083.499 | 10 | 0.3 | 542.7567 | 2 | 23.6 | 680 | 689 | Deamidation (NQ) | PEAKS DB |
| A.SGAGAGAGAGTGSSGFGPYVANGGYSR.R | 29.31 | 2332.047 | 27 | 1.6 | 778.3575 | 3 | 33.5 | 5124 | 5150 |  | PEAKS DB |
| A.VIEEQITTK.K | 28.9 | 1059.581 | 9 | -0.1 | 530.7978 | 2 | 24 | 54 | 62 |  | PEAKS DB |
| Y.VAN(+.98)GGYSR.S | 28.23 | 823.3824 | 8 | 0.2 | 412.6985 | 2 | 17.5 | 682 | 689 | Deamidation (NQ) | PEAKS DB |
| K.SDFETGSGAASGAGAGAGSGAGAGS.G | 27.87 | 1955.809 | 25 | 1.6 | 978.9135 | 2 | 28.5 | 5162 | 5186 |  | PEAKS DB |
| K.SDFETGSGAA.S | 27.52 | 940.3774 | 10 | -0.5 | 471.1957 | 2 | 25.1 | 5162 | 5171 |  | PEAKS DB |
| D.ASGAVIEEQITTKK.M | 27.5 | 1473.804 | 14 | -0.7 | 737.9087 | 2 | 24.7 | 50 | 63 |  | PEAKS DB |
| R.REGYEY(+15.99)AWSSK.S | 27.2 | 1390.615 | 11 | -0.9 | 696.3143 | 2 | 20.2 | 5151 | 5161 | Oxidation or Hydroxylation | PEAKS DB |
| R.KN(+.98)C(+57.02)GIPR.R | 26.93 | 844.4225 | 7 | 1.6 | 423.2192 | 2 | 17 | 5242 | 5248 | Deamidation (NQ); Carbamidomethylation | PEAKS DB |
| G.YGQGAGSAASSVSSASSR.S | 26.83 | 1628.739 | 18 | 1.4 | 815.3779 | 2 | 22.2 | 5214 | 5231 |  | PEAKS DB |
| Y.VAN(+.98)GGYSGYEYA.W | 26.29 | 1250.509 | 12 | -0.1 | 626.2618 | 2 | 34.6 | 1639 | 1650 | Deamidation (NQ) | PEAKS DB |
| R.GY(+15.99)GQGAGSAASSVSSASSR.S | 25.5 | 1701.755 | 19 | 0.4 | 851.8853 | 2 | 21.2 | 5213 | 5231 | Oxidation or Hydroxylation | PEAKS DB |
| G.AGSAASSVSSASSR.S | 25.23 | 1223.574 | 14 | -0.2 | 612.7943 | 2 | 18.1 | 5218 | 5231 |  | PEAKS DB |
| R.DASGAVIEEQITTKKMQR(+15.99).K | 24.72 | 2020.026 | 18 | -2 | 674.3479 | 3 | 25.5 | 49 | 66 | Oxidation or Hydroxylation | PEAKS DB |
| R.SYDYSRR.N | 24.53 | 945.4304 | 7 | 0.2 | 316.1508 | 3 | 17.2 | 5232 | 5238 |  | PEAKS DB |
| R.DASGAVIEEQITTKKM(+15.99)QR.K | 24.19 | 2020.026 | 18 | 0.1 | 674.3493 | 3 | 25.6 | 49 | 66 | Oxidation (M) | PEAKS DB |
| G.PYVANGGYSR.S | 23.34 | 1082.515 | 10 | 0.2 | 542.2646 | 2 | 22.1 | 680 | 689 |  | PEAKS DB |
| Y.VANGGYSR.S | 23.33 | 822.3984 | 8 | -0.6 | 412.2062 | 2 | 14 | 682 | 689 |  | PEAKS DB |
| R.GYGQGAGSAASSVSSASSRSYDYSR.R | 22.85 | 2457.079 | 25 | 0.1 | 820.0338 | 3 | 26.1 | 5213 | 5237 |  | PEAKS DB |
| G.SGAASGAGAGAGAGTGSSGFGPYVAN(+.98)GGYSR.R | 20.89 | 2619.158 | 31 | 1.1 | 874.061 | 3 | 34.9 | 5120 | 5150 | Deamidation (NQ) | PEAKS DB |
| S.GFGPYVAN(+.98)GGYSR.S | 20.67 | 1344.61 | 13 | -3.6 | 673.3098 | 2 | 33.9 | 677 | 689 | Deamidation (NQ) | PEAKS DB |
| R.DASGAVIEEQITTKK(+15.99)MQR.K | 20.59 | 2020.026 | 18 | 0.1 | 506.0138 | 4 | 25.7 | 49 | 66 | Oxidation or Hydroxylation | PEAKS DB |
| S.GAVIEEQITTKK.M | 19.95 | 1315.735 | 12 | 0.9 | 658.8752 | 2 | 29 | 52 | 63 |  | PEAKS DB |
| R.REGYEYA.W | 19.89 | 886.3821 | 7 | 0 | 444.1983 | 2 | 21.1 | 5151 | 5157 |  | PEAKS DB |
| A.SGAVIEEQITTKK.M | 19.16 | 1402.767 | 13 | 0 | 702.3906 | 2 | 29.1 | 51 | 63 |  | PEAKS DB |
| R.GYGQGAGSAASSVSS.A | 18.61 | 1284.558 | 15 | 6.5 | 643.2906 | 2 | 24.2 | 5213 | 5227 |  | PEAKS DB |
| R.REGY(+15.99)EY(+15.99)AWSSK.S | 18.17 | 1406.61 | 11 | 0.7 | 704.3129 | 2 | 21.7 | 5151 | 5161 | Oxidation or Hydroxylation | PEAKS DB |
| R.EGY(+15.99)EY(+15.99)AWSSK.S | 17.58 | 1250.509 | 10 | -0.1 | 626.2618 | 2 | 28.6 | 5152 | 5161 | Oxidation or Hydroxylation | PEAKS DB |
| R.REGYEY(+15.99)AWSSK(+15.99).S | 17.23 | 1406.61 | 11 | 0.3 | 469.8775 | 3 | 24.3 | 5151 | 5161 | Oxidation or Hydroxylation | PEAKS DB |
| **FA-TRYPSIN** |  |  |  |  |  |  |  |  |  |  |  |
| **Peptide** | **-10lgP** | **Mass** | **Length** | **ppm** | **m/z** | **z** | **RT** | **Start** | **End** | **PTM** | **Found By** |
| R.DASGAVIEEQITTK(+27.99)K.M | 55.91 | 1616.826 | 15 | 0.3 | 809.4203 | 2 | 32.2 | 49 | 63 | Formylation | PEAKS PTM |
| R.DASGAVIEEQITTK.K | 53.96 | 1460.736 | 14 | 0.4 | 731.3755 | 2 | 30 | 49 | 62 |  | PEAKS DB |
| R.GY(+44.99)GQGAGSAASSVSSASSR.S | 53.14 | 1730.746 | 19 | 0.6 | 866.3806 | 2 | 22 | 5213 | 5231 | Oxidation to nitro | PEAKS PTM |
| R.DASGAVIE(+14.02)EQITTK.K | 46.4 | 1474.752 | 14 | -0.5 | 738.3826 | 2 | 35.3 | 49 | 62 | Methylation(others) | PEAKS PTM |
| A.S(+27.99)GAVIEEQITTK.K | 45.54 | 1302.667 | 12 | 0.4 | 652.3408 | 2 | 34.3 | 51 | 62 | Formylation | PEAKS PTM |
| G.Q(-17.03)GAGSAASSVSSASSR.S | 45.49 | 1391.628 | 16 | -0.5 | 696.8208 | 2 | 21.7 | 5216 | 5231 | Pyro-glu from Q | PEAKS PTM |
| Y.GQ(+.98)GAGSAASSVSSASSR.S | 45.45 | 1466.66 | 17 | 0.3 | 734.3373 | 2 | 21.2 | 5215 | 5231 | Deamidation (NQ) | PEAKS DB |
| Y.G(+43.01)QGAGSAASSVSSASSR.S | 45.33 | 1508.682 | 17 | -0.3 | 755.3478 | 2 | 21 | 5215 | 5231 | Carbamylation | PEAKS PTM |
| R.DASGAVIEE(+14.02)QITTK.K | 45.17 | 1474.752 | 14 | 0.3 | 738.3832 | 2 | 36.4 | 49 | 62 | Methylation(others) | PEAKS PTM |
| D.EIIRD(-18.01)ASGAVIEEQITTK.K | 44.96 | 1954.037 | 18 | 0.5 | 978.0264 | 2 | 39.4 | 45 | 62 | Dehydration | PEAKS PTM |
| Y.GQGAGSAASSVSSASSR.S | 44.69 | 1465.676 | 17 | 0.6 | 733.8455 | 2 | 17 | 5215 | 5231 |  | PEAKS DB |
| R.GYGQGAGSAASSVSSASSR.S | 44.56 | 1685.761 | 19 | 1.1 | 843.8885 | 2 | 20.6 | 5213 | 5231 |  | PEAKS DB |
| Q.G(+27.99)AGSAASSVSSASSR.S | 42.48 | 1308.591 | 15 | -3.6 | 655.3002 | 2 | 21.1 | 5217 | 5231 | Formylation | PEAKS PTM |
| A.G(+27.99)SAASSVSSASSR.S | 41.88 | 1180.532 | 13 | 0.4 | 591.2735 | 2 | 19.4 | 5219 | 5231 | Formylation | PEAKS PTM |
| Q.GAGSAASSVSSASSR.S | 41.28 | 1280.596 | 15 | 0.7 | 641.3055 | 2 | 16.3 | 5217 | 5231 |  | PEAKS DB |
| S.A(+27.99)ASSVSSASSR.S | 41.08 | 1036.479 | 11 | -0.4 | 519.2463 | 2 | 18.4 | 5221 | 5231 | Formylation | PEAKS PTM |
| A.SE(sub G)AVIEEQITTK.K | 40.49 | 1346.693 | 12 | 0.9 | 674.3543 | 2 | 36.5 | 51 | 62 |  | SPIDER |
| G.A(+58.01)GSAASSVSSASSR.S | 40.47 | 1281.58 | 14 | -0.1 | 641.797 | 2 | 21 | 5218 | 5231 | Carboxymethyl (KW, X@N-term) | PEAKS PTM |
| D.EIIR(+.98)DASGAVIEEQITTK.K | 40.31 | 1973.032 | 18 | -0.1 | 987.523 | 2 | 40.4 | 45 | 62 | Deamidation (R) | PEAKS PTM |
| D.E(+27.99)IIRDASGAVIEEQITTK.K | 40.17 | 2000.043 | 18 | -0.1 | 1001.028 | 2 | 40.3 | 45 | 62 | Formylation | PEAKS PTM |
| R.GYGQGM(sub A)GSAASSVSSASSR.S | 40.08 | 1745.764 | 19 | -3.3 | 873.8863 | 2 | 22.6 | 5213 | 5231 |  | SPIDER |
| S.G(+27.99)AVIEEQITTK.K | 40.01 | 1215.635 | 11 | 1 | 608.8252 | 2 | 35 | 52 | 62 | Formylation | PEAKS PTM |
| R.DASGAVIEEQIT(-18.01)TK.K | 39.13 | 1442.725 | 14 | 0.4 | 722.3702 | 2 | 31.7 | 49 | 62 | Dehydration | PEAKS PTM |
| D.E(-18.01)IIRDASGAVIEEQITTK.K | 38.74 | 1954.037 | 18 | 0.5 | 978.0263 | 2 | 40 | 45 | 62 | Pyro-glu from E | PEAKS PTM |
| Q.G(+43.01)AGSAASSVSSASSR.S | 38.57 | 1323.601 | 15 | 0.6 | 662.8084 | 2 | 21.2 | 5217 | 5231 | Carbamylation | PEAKS PTM |
| R.RQLVVK(+27.99)FR.A | 38.55 | 1072.651 | 8 | 0.4 | 537.3328 | 2 | 27.5 | 5249 | 5256 | Formylation | PEAKS PTM |
| G.S(+27.99)AASSVSSASSR.S | 38.31 | 1123.511 | 12 | -0.9 | 562.762 | 2 | 18.8 | 5220 | 5231 | Formylation | PEAKS PTM |
| R.GYGQ(+.98)GM(sub A)GSAASSVSSASSR.S | 37.29 | 1746.748 | 19 | -1.2 | 874.3801 | 2 | 22 | 5213 | 5231 | Deamidation (NQ) | SPIDER |
| R.C(sub G)Y(+15.99)GQGAGSAASSVSSASSR.S | 37.16 | 1747.743 | 19 | -2.7 | 874.8765 | 2 | 22.2 | 5213 | 5231 | Oxidation or Hydroxylation | SPIDER |
| A.D(sub S)GP(sub A)VIEEQITTK.K | 36.76 | 1328.682 | 12 | 0.7 | 665.3489 | 2 | 37.2 | 51 | 62 |  | SPIDER |
| K.SDFETGSGAASGAGAG(+15.99).A | 36.53 | 1356.543 | 16 | 0.8 | 679.2793 | 2 | 22.3 | 5162 | 5177 | Oxidation or Hydroxylation | PEAKS DB |
| D.A(+27.99)SGAVIEEQITTKK.M | 36.5 | 1501.799 | 14 | 0.2 | 751.9068 | 2 | 29.7 | 50 | 63 | Formylation | PEAKS PTM |
| A.S(+43.01)GAVIEEQITTK.K | 35.71 | 1317.678 | 12 | 0.1 | 659.8461 | 2 | 33.5 | 51 | 62 | Carbamylation | PEAKS PTM |
| G.A(+27.99)VIEEQITTK.K | 35.56 | 1158.613 | 10 | 0.5 | 580.3141 | 2 | 34.8 | 53 | 62 | Formylation | PEAKS PTM |
| R.DASGAVIEEQITT(-18.01)K.K | 35.3 | 1442.725 | 14 | 0.2 | 722.3701 | 2 | 33.4 | 49 | 62 | Dehydration | PEAKS PTM |
| K.SDFETGSGAASGAG.A | 35.21 | 1212.489 | 14 | 1.4 | 607.2528 | 2 | 22.2 | 5162 | 5175 |  | PEAKS DB |
| R.Q(-17.03)LVVKFR.A | 34.58 | 871.528 | 7 | -0.7 | 436.771 | 2 | 32.6 | 5250 | 5256 | Pyro-glu from Q | PEAKS PTM |
| R.GY(+44.99)GQGAGSAASSVSSAS.S | 34.55 | 1487.612 | 17 | 0.6 | 744.8139 | 2 | 26.3 | 5213 | 5229 | Oxidation to nitro | PEAKS PTM |
| G.A(+43.01)GSAASSVSSASSR.S | 34.12 | 1266.58 | 14 | -0.6 | 634.2969 | 2 | 21.6 | 5218 | 5231 | Carbamylation | PEAKS PTM |
| R.RQ(+.98)LVVK(+27.99)FR.A | 33.74 | 1073.635 | 8 | 0.2 | 537.8246 | 2 | 32.2 | 5249 | 5256 | Deamidation (NQ); Formylation | PEAKS PTM |
| R.DASGAVIEEQ(+.98)ITTK.K | 33.57 | 1461.72 | 14 | 3 | 731.8694 | 2 | 35.6 | 49 | 62 | Deamidation (NQ) | PEAKS PTM |
| R.GY(+44.99)GQGAGSAASSVSSA.S | 33.55 | 1400.58 | 16 | 0.8 | 701.298 | 2 | 26.7 | 5213 | 5228 | Oxidation to nitro | PEAKS PTM |
| G.AGSAASSVSSASSR.S | 33.52 | 1223.574 | 14 | 0.1 | 612.7944 | 2 | 15.7 | 5218 | 5231 |  | PEAKS DB |
| R.GY(+15.99)GQGAGSAASSVSSASSR.S | 33.52 | 1701.755 | 19 | -0.2 | 851.8848 | 2 | 18.5 | 5213 | 5231 | Oxidation or Hydroxylation | PEAKS DB |
| S.GE(sub A)VIEEQITTK.K | 33.15 | 1245.645 | 11 | 0.5 | 623.8302 | 2 | 34.3 | 52 | 62 |  | SPIDER |
| G.Y(+15.99)GN(sub Q)GAGSAASSVSSASSR.S | 32.95 | 1630.718 | 18 | 7.1 | 816.3722 | 2 | 17.7 | 5214 | 5231 | Oxidation or Hydroxylation | SPIDER |
| G.S(+27.99)GAGAGSGAGSGAGAGSGAGAG.S | 32.87 | 1518.629 | 22 | 0.7 | 760.3225 | 2 | 20.6 | 2561 | 2582 | Formylation | PEAKS PTM |
| S.A(+43.01)ASSVSSASSR.S | 32.87 | 1051.489 | 11 | 0 | 526.752 | 2 | 19.1 | 5221 | 5231 | Carbamylation | PEAKS PTM |
| A.S(+117.00)GAVIEEQITTK.K | 32.59 | 1391.67 | 12 | 6.3 | 696.8465 | 2 | 42.1 | 51 | 62 | Phospho-propargylamine | PEAKS PTM |
| A.SGAVIEEQITTK.K | 32.57 | 1274.672 | 12 | -0.9 | 638.3426 | 2 | 25.5 | 51 | 62 |  | PEAKS DB |
| A.E(sub G)SAASSVSSASSR.S | 32.48 | 1224.558 | 13 | -0.4 | 613.2861 | 2 | 20.3 | 5219 | 5231 |  | SPIDER |
| G.QGAGS(+114.04)AASSVSSASSR.S | 32.38 | 1522.697 | 16 | 2.8 | 762.358 | 2 | 17.2 | 5216 | 5231 | Ubiquitin | PEAKS PTM |
| R.Q(+.98)LVVK(+27.99)FR.A | 32.03 | 917.5334 | 7 | 0.9 | 459.7744 | 2 | 33 | 5250 | 5256 | Deamidation (NQ); Formylation | PEAKS PTM |
| K.NA(sub C)GIPR.R | 31.92 | 626.35 | 6 | 0 | 314.1823 | 2 | 15.4 | 5243 | 5248 |  | SPIDER |
| S.G(+58.01)AVIEEQITTK.K | 31.9 | 1245.645 | 11 | 1.1 | 623.8306 | 2 | 33.7 | 52 | 62 | Carboxymethyl (KW, X@N-term) | PEAKS PTM |
| G.QP(sub G)AGSAASSVSSASSR.S | 31.62 | 1448.686 | 16 | 0.7 | 725.3505 | 2 | 19 | 5216 | 5231 |  | SPIDER |
| R.G(+42.01)Y(-18.01)GQGAGSAASSVSSASSR.S | 31.56 | 1709.761 | 19 | -9.3 | 855.8796 | 2 | 18.4 | 5213 | 5231 | Acetylation (N-term); Dehydration | PEAKS PTM |
| R.RQLVVK(+43.01)FR.A | 31.4 | 1087.661 | 8 | 0.5 | 544.8383 | 2 | 31.8 | 5249 | 5256 | Carbamylation | PEAKS PTM |
| R.QLVVK(+43.01)FR.A | 31.34 | 931.5603 | 7 | -0.6 | 466.7871 | 2 | 29.4 | 5250 | 5256 | Carbamylation | PEAKS PTM |
| R.DASGAVIEEQITTKK.M | 31.24 | 1588.831 | 15 | 1 | 795.4235 | 2 | 25.7 | 49 | 63 |  | PEAKS DB |
| K.SDFETGSGAASGAGAGAGSGAGAGSG.A | 31.12 | 2012.831 | 26 | 0 | 1007.423 | 2 | 24.4 | 5162 | 5187 |  | PEAKS DB |
| G.Q(+.98)GE(sub A)GSAASSVSSASSR.S | 31.09 | 1467.644 | 16 | -0.6 | 734.8287 | 2 | 22.1 | 5216 | 5231 | Deamidation (NQ) | SPIDER |
| R.QLVVK(+27.99)FR.A | 31.08 | 916.5494 | 7 | 0.1 | 459.282 | 2 | 29.6 | 5250 | 5256 | Formylation | PEAKS PTM |
| R.GY(+44.99)GQGAGSAASSVS.S | 31 | 1242.511 | 14 | 0.2 | 622.263 | 2 | 25 | 5213 | 5226 | Oxidation to nitro | PEAKS PTM |
| R.D(+27.99)ASGAVIEEQITTK.K | 30.73 | 1488.731 | 14 | -0.5 | 745.3723 | 2 | 36 | 49 | 62 | Formylation | PEAKS PTM |
| K.SDFETGSGAASG.A | 30.27 | 1084.431 | 12 | 0.1 | 543.2228 | 2 | 21.2 | 5162 | 5173 |  | PEAKS DB |
| Y.GQGQ(sub A)GSAASSVSSASSR.S | 30.2 | 1522.697 | 17 | -4.6 | 762.3524 | 2 | 22.8 | 5215 | 5231 |  | SPIDER |
| S.G(+43.01)AVIEEQITTK.K | 30.15 | 1230.646 | 11 | -0.1 | 616.33 | 2 | 34.3 | 52 | 62 | Carbamylation | PEAKS PTM |
| G.A(+58.01)VIEEQITTK.K | 30.06 | 1188.624 | 10 | 0.4 | 595.3194 | 2 | 34.3 | 53 | 62 | Carboxymethyl (KW, X@N-term) | PEAKS PTM |
| R.GYGQ(+.98)GD(sub A)GSAASSVSSASSR.S | 29.95 | 1730.734 | 19 | 6.5 | 866.38 | 2 | 21.5 | 5213 | 5231 | Deamidation (NQ) | SPIDER |
| K.SDFETGSGAASGAGAGAG(+15.99).S | 29.81 | 1484.601 | 18 | 1.1 | 743.3088 | 2 | 23 | 5162 | 5179 | Oxidation or Hydroxylation | PEAKS DB |
| S.E(sub G)AVIEEQITTK.K | 29.58 | 1259.661 | 11 | -0.2 | 630.8376 | 2 | 37.5 | 52 | 62 |  | SPIDER |
| G.A(+27.99)GSGAGAGSGAGSGAGAGSGA.G | 29.53 | 1461.608 | 21 | 0.8 | 731.8118 | 2 | 21 | 2559 | 2579 | Formylation | PEAKS PTM |
| K.SDFETGSGAASGAG(-.98).A | 29.51 | 1211.505 | 14 | 0 | 606.7599 | 2 | 21.1 | 5162 | 5175 | Amidation | PEAKS PTM |
| A.A(+27.99)SSVSSASSR.S | 29.35 | 965.4414 | 10 | 0.5 | 483.7282 | 2 | 17.3 | 5222 | 5231 | Formylation | PEAKS PTM |
| N.T(+27.99)TDEIIRDASGAVIEEQITTK.K | 29.31 | 2317.165 | 21 | 1.1 | 1159.591 | 2 | 40.4 | 42 | 62 | Formylation | PEAKS PTM |
| G.QGAGSAASSVSSASSR.S | 29.02 | 1408.654 | 16 | 0.9 | 705.335 | 2 | 16.8 | 5216 | 5231 |  | PEAKS DB |
| S.G(+43.01)AGAGSGAGAGSGAGAG.S | 29 | 1174.496 | 17 | 0.3 | 588.2556 | 2 | 19.3 | 424 | 440 | Carbamylation | PEAKS PTM |
| G.YGH(sub Q)GAGSAASSVSSASSR.S | 28.95 | 1637.739 | 18 | -9 | 819.8696 | 2 | 18.2 | 5214 | 5231 |  | SPIDER |
| G.YGM(sub Q)GAGSAASSVSSASSR.S | 28.85 | 1631.721 | 18 | -5.3 | 816.8634 | 2 | 18.7 | 5214 | 5231 |  | SPIDER |
| R.DASGAVIE(+21.98)EQITTK.K | 28.53 | 1482.718 | 14 | -1.3 | 742.3652 | 2 | 30.3 | 49 | 62 | Sodium adduct | PEAKS PTM |
| G.A(+43.01)ASGAGAGSGAGAGSGAGAGSGAG.A | 28.4 | 1675.715 | 24 | 1.2 | 838.8656 | 2 | 16.9 | 499 | 522 | Carbamylation | PEAKS PTM |
| S.T(sub A)E(sub A)SSVSSASSR.S | 28.3 | 1096.5 | 11 | -0.2 | 549.257 | 2 | 18.7 | 5221 | 5231 |  | SPIDER |
| R.GY(-18.01)GQGAGSAASSVSSASSR.S | 28.19 | 1667.75 | 19 | -10 | 834.8738 | 2 | 18.3 | 5213 | 5231 | Dehydration | PEAKS PTM |
| A.T(sub S)GE(sub A)VIEEQITTK.K | 28.17 | 1346.693 | 12 | 1 | 674.3544 | 2 | 35.5 | 51 | 62 |  | SPIDER |
| S.GAGAGSGAGAGSGAGAGSGAGAG(-.98).S | 28.12 | 1530.677 | 23 | -0.8 | 766.3452 | 2 | 16.3 | 424 | 446 | Amidation | PEAKS PTM |
| S.GAGAGSGAGAGSGAGAGSGAGAG.S | 28.07 | 1531.661 | 23 | 0.1 | 766.8379 | 2 | 16.8 | 424 | 446 |  | PEAKS DB |
| R.DASGAVIEEQ(+.98)ITTKK.M | 27.39 | 1589.815 | 15 | 1 | 795.9155 | 2 | 28.6 | 49 | 63 | Deamidation (NQ) | PEAKS PTM |
| G.V(+43.01)GSGAGAGSGAGAGSGAGAGSGAGAG.Y | 27.28 | 1817.789 | 26 | 0.1 | 909.9017 | 2 | 18.1 | 1492 | 1517 | Carbamylation | PEAKS PTM |
| D.Q(sub A)SGAVIEEQITTK.K | 27.27 | 1402.73 | 13 | -6.4 | 702.368 | 2 | 38 | 50 | 62 |  | SPIDER |
| R.DASGAVIEEQITTKK(+43.01).M | 27.2 | 1631.837 | 15 | 0.2 | 816.9257 | 2 | 31.5 | 49 | 63 | Carbamylation | PEAKS PTM |
| G.A(+42.01)GSGSGAGSGAGAGSGAGAG.S | 27.19 | 1404.587 | 20 | 0.1 | 703.3006 | 2 | 20.6 | 4455 | 4474 | Acetylation (N-term) | PEAKS PTM |
| S.GAGAGSGAGAGSGAGAGSGAGAG(+21.98).S | 27.09 | 1553.643 | 23 | 0.1 | 777.8288 | 2 | 16.8 | 424 | 446 | Sodium adduct | PEAKS PTM |
| R.RQ(+.98)LVVKFR.A | 27.07 | 1045.64 | 8 | 0.1 | 523.8271 | 2 | 31.8 | 5249 | 5256 | Deamidation (NQ) | PEAKS DB |
| G.A(+43.01)ASGAGAGSGAGAGAGSGAGAGSGA.G | 27.03 | 1746.752 | 25 | 0.8 | 874.3838 | 2 | 17.7 | 2533 | 2557 | Carbamylation | PEAKS PTM |
| Y.G(+43.01)AGAGSGAASGAGAGAGAGAG.T | 26.98 | 1444.629 | 21 | 0.7 | 723.3223 | 2 | 22.7 | 652 | 672 | Carbamylation | PEAKS PTM |
| R.QLVVK(+43.99)FR.A | 26.95 | 932.5443 | 7 | 0.8 | 467.2798 | 2 | 25.2 | 5250 | 5256 | Carboxylation (DKW) | PEAKS PTM |
| G.SE(sub A)ASSVSSASSR.S | 26.85 | 1153.521 | 12 | -1 | 577.7673 | 2 | 19 | 5220 | 5231 |  | SPIDER |
| G.SGAGAGSGAGAGSGAGAGSGAGAGSG.A | 26.84 | 1762.747 | 26 | 1.3 | 882.3817 | 2 | 17 | 423 | 448 |  | PEAKS DB |
| A.GAGSGAGAGSGAGAGSGAGAGG.S | 26.71 | 1460.624 | 22 | -0.1 | 731.3192 | 2 | 16.4 | 5183 | 5204 |  | PEAKS DB |
| S.G(+43.01)AGAGSGAGAGSGAGAGSGAG.A | 26.5 | 1446.608 | 21 | 0.5 | 724.3118 | 2 | 20.4 | 424 | 444 | Carbamylation | PEAKS PTM |
| A.GAGAGSGAGAGSGAGAGAGSGAGAG.Y | 26.5 | 1659.72 | 25 | 0.9 | 830.8679 | 2 | 17.4 | 3883 | 3907 |  | PEAKS DB |
| Y.GAGAGSGAASGAGAGAGAGAG.T | 26.48 | 1401.623 | 21 | -0.3 | 701.8187 | 2 | 17.5 | 652 | 672 |  | PEAKS DB |
| G.A(+27.99)GSGAGSGAGAGSGAGAG.S | 26.34 | 1246.517 | 18 | 0.2 | 624.2661 | 2 | 20.1 | 1310 | 1327 | Formylation | PEAKS PTM |
| S.GAGAGSGAGAGSGAGAGSGAGAGG.S | 26.31 | 1588.683 | 24 | 0.1 | 795.3486 | 2 | 16.9 | 5181 | 5204 |  | PEAKS DB |
| G.A(+27.99)GSGAGAGSGAGAGSGAGAG.S | 26.04 | 1374.576 | 20 | -0.1 | 688.2952 | 2 | 20.7 | 421 | 440 | Formylation | PEAKS PTM |
| S.GAGAGSGAGAGSGAGSGAGAGSGAGAG.S | 25.81 | 1803.773 | 27 | -0.4 | 902.8934 | 2 | 17.4 | 2556 | 2582 |  | PEAKS DB |
| Y.G(+27.99)AGAGSGAGSGAGAGSGAGAG.S | 25.78 | 1431.597 | 21 | -0.4 | 716.8057 | 2 | 20.7 | 1307 | 1327 | Formylation | PEAKS PTM |
| Y.GAGAGSGAASGAGAGSGAGAG.S | 25.71 | 1417.618 | 21 | 0.5 | 709.8167 | 2 | 17.1 | 492 | 512 |  | PEAKS DB |
| S.GAGAGSGAGAGSGAGSGAGAGSGAGAG(+21.98).S | 25.7 | 1825.755 | 27 | 1.4 | 913.886 | 2 | 17.4 | 2556 | 2582 | Sodium adduct | PEAKS PTM |
| G.S(+27.99)GAGAGSGAGAGSGAGA.G | 25.69 | 1189.496 | 17 | 0.6 | 595.7556 | 2 | 20.2 | 423 | 439 | Formylation | PEAKS PTM |
| G.V(+43.01)GSGAGAGSGAGAGSGAGAG.S | 25.66 | 1417.618 | 20 | 0.2 | 709.8165 | 2 | 16.6 | 1492 | 1511 | Carbamylation | PEAKS PTM |
| G.SGAGAGAGSGAGAGSGAGAGSGAGAG.S | 25.61 | 1746.752 | 26 | 0.3 | 874.3834 | 2 | 17.2 | 1071 | 1096 |  | PEAKS DB |
| R.RQLVVKFR(+.98).A | 25.51 | 1045.64 | 8 | -1.2 | 523.8265 | 2 | 31.1 | 5249 | 5256 | Deamidation (R) | PEAKS PTM |
| R.D(+21.97)ASGAVIEEQITTK.K | 25.38 | 1482.705 | 14 | -6.9 | 742.3547 | 2 | 44.6 | 49 | 62 | Replacement of 2 protons by magnesium | PEAKS PTM |
| G.AGAGSGAASGAGAGAGA.G | 25.33 | 1159.522 | 17 | -0.1 | 580.7681 | 2 | 16.8 | 653 | 669 |  | PEAKS DB |
| A.G(+27.99)SGAGAGSGAGAGSGAGSGAG.A | 25.25 | 1447.592 | 21 | 0.2 | 724.8035 | 2 | 19.8 | 2554 | 2574 | Formylation | PEAKS PTM |
| S.GAGAGSGAGAGSGAGSGAGAGSGAGAG(-.98).S | 25.25 | 1802.789 | 27 | 1.3 | 902.403 | 2 | 16.9 | 2556 | 2582 | Amidation | PEAKS PTM |
| Y.GAGAGSGAGSGAGAGSGAGAG.S | 25.24 | 1403.603 | 21 | 0.1 | 702.8086 | 2 | 16.3 | 1307 | 1327 |  | PEAKS DB |
| W.SSESDFGTGSGAGAG.S | 25.09 | 1285.506 | 15 | -0.3 | 643.76 | 2 | 22.4 | 1249 | 1263 |  | PEAKS DB |
| R.DASGAVIEEQITTK(+27.99).K | 24.96 | 1488.731 | 14 | -1 | 745.3719 | 2 | 31.8 | 49 | 62 | Formylation | PEAKS PTM |
| S.G(+127.06)AVIEEQITTK.K | 24.95 | 1314.703 | 11 | -0.4 | 658.3586 | 2 | 28.5 | 52 | 62 | N-Succinimidyl-2-morpholine acetate | PEAKS PTM |
| S.GAGSGAGAGSGAGAGSGAGAGAG(+15.99).S | 24.94 | 1547.656 | 23 | 0.1 | 774.8354 | 2 | 16.4 | 4460 | 4482 | Oxidation or Hydroxylation | PEAKS PTM |
| A.E(sub S)GT(sub A)VIEEQITTK.K | 24.88 | 1346.693 | 12 | -0.6 | 674.3533 | 2 | 34.3 | 51 | 62 |  | SPIDER |
| G.AGSGAASGAGAGAGAGAG.T | 24.86 | 1216.543 | 18 | 0.3 | 609.2791 | 2 | 16.5 | 655 | 672 |  | PEAKS DB |
| Y.GAGAGSGAGSGAGAGSGAGAGSGAG(-.98).A | 24.85 | 1674.731 | 25 | 0.9 | 838.3733 | 2 | 16.5 | 1307 | 1331 | Amidation | PEAKS PTM |
| A.G(+42.01)SGAGSGAGAGSGSGAGAG.S | 24.74 | 1333.549 | 19 | -1.1 | 667.7812 | 2 | 19.7 | 4687 | 4705 | Acetylation (N-term) | PEAKS PTM |
| A.G(+27.99)SGAGAGSGAGAGSGAGAG.S | 24.55 | 1303.539 | 19 | -0.1 | 652.7766 | 2 | 19.7 | 422 | 440 | Formylation | PEAKS PTM |
| S.E(sub A)T(sub A)SSVSSASSR.S | 24.54 | 1096.5 | 11 | 0.1 | 549.2571 | 2 | 17.3 | 5221 | 5231 |  | SPIDER |
| R.Q(+.98)LVVK(+43.01)FR.A | 24.52 | 932.5443 | 7 | 0 | 467.2794 | 2 | 33.8 | 5250 | 5256 | Deamidation (NQ); Carbamylation | PEAKS PTM |
| G.S(+42.01)GAGAGSGAGAGSGAGAG.S | 24.48 | 1260.533 | 18 | 0.1 | 631.2738 | 2 | 20.9 | 423 | 440 | Acetylation (N-term) | PEAKS PTM |
| G.SGT(-18.01)GSGAGAGSGAGAGSGAGAG.S | 24.46 | 1502.635 | 22 | 1.9 | 752.3259 | 2 | 21.7 | 3365 | 3386 | Dehydration | PEAKS PTM |
| G.S(+27.99)GAASGAGAGAGAGAGTG.S | 24.28 | 1274.549 | 18 | 0.1 | 638.2817 | 2 | 22.1 | 657 | 674 | Formylation | PEAKS PTM |
| G.S(+27.99)GAGAGSGAGAGSGAGAGSGAG.A | 24.21 | 1518.629 | 22 | -0.5 | 760.3216 | 2 | 21.1 | 423 | 444 | Formylation | PEAKS PTM |
| Y.GAGAGSGAGSGAGAGSGAGAG(+21.98).S | 24.11 | 1425.585 | 21 | 0.1 | 713.7996 | 2 | 16.3 | 1307 | 1327 | Sodium adduct | PEAKS PTM |
| G.AGAGSGAASGAGAGSGAGAG.S | 24.07 | 1360.597 | 20 | 0.4 | 681.3059 | 2 | 16.7 | 493 | 512 |  | PEAKS DB |
| A.G(+43.01)VGSGAGAGSGAGAGSGAGAG.S | 24.06 | 1474.64 | 21 | 0.6 | 738.3275 | 2 | 16.6 | 1491 | 1511 | Carbamylation | PEAKS PTM |
| **CHYMOTRYPSIN** |  |  |  |  |  |  |  |  |  |  |  |
| **Peptide** | **-10lgP** | **Mass** | **Length** | **ppm** | **m/z** | **z** | **RT** | **Start** | **End** | **PTM** | **Found By** |
| G.SGAASGAGAGAGAGAGTGSSGFGPY.V | 38.36 | 1941.845 | 25 | 0 | 971.9299 | 2 | 48.8 | 657 | 681 |  | PEAKS DB |
| G.AASGAGAGAGAGAGTGSSGFGPY.V | 37.21 | 1797.792 | 23 | 0.5 | 899.9036 | 2 | 48.7 | 659 | 681 |  | PEAKS DB |
| G.AGSGAASGAGAGAGAGAGTGSSGFGPY.V | 35.24 | 2069.904 | 27 | -0.5 | 1035.959 | 2 | 49 | 655 | 681 |  | PEAKS DB |
| G.AGAGAGAGTGSSGFGPY.V | 33.49 | 1383.606 | 17 | -0.5 | 692.8096 | 2 | 47.8 | 665 | 681 |  | PEAKS DB |
| G.SGAGAGYGAGVGAGYGVGY.G | 31.74 | 1589.711 | 19 | 0.3 | 795.863 | 2 | 52.3 | 1773 | 1791 |  | PEAKS DB |
| Y.EYAWSSESDFGTGSGAGAG.S | 31.54 | 1834.728 | 19 | 1.2 | 918.3724 | 2 | 53.9 | 1245 | 1263 |  | SPIDER |
| Y.GAGAGSGAASGAGAGAGAGAGTGSSGFGPY.V | 31.52 | 2254.984 | 30 | 0.3 | 752.6688 | 3 | 49.4 | 652 | 681 |  | PEAKS DB |
| G.AGAGAGAGAGTGSSGFGPY.V | 31.45 | 1511.664 | 19 | -0.2 | 756.8392 | 2 | 48.2 | 663 | 681 |  | PEAKS DB |
| G.A(+27.99)ASGAGAGAGAGAGTGSSGFGPY.V | 30.94 | 1825.787 | 23 | 0.9 | 913.9014 | 2 | 56.8 | 659 | 681 | Formylation | PEAKS PTM |
| A.GAGAGAGAGTGSSGFGPY.V | 30.78 | 1440.627 | 18 | 0.4 | 721.321 | 2 | 48 | 664 | 681 |  | PEAKS DB |
| A.SGAGAGAGAGAGTGSSGFGPY.V | 30.77 | 1655.718 | 21 | 0 | 828.866 | 2 | 48.4 | 661 | 681 |  | PEAKS DB |
| S.GAASGAGAGAGAGAGTGSSGFGPY.V | 29.77 | 1854.813 | 24 | 0.1 | 928.414 | 2 | 49.1 | 658 | 681 |  | PEAKS DB |
| G.SGAGAGSGAGAGSGAGAGSGAGAGY.G | 29.55 | 1781.756 | 25 | 0.1 | 891.8855 | 2 | 36.2 | 513 | 537 |  | SPIDER |
| A.GAGAGTGSSGFGPY.V | 29.12 | 1184.51 | 14 | 0.1 | 593.2622 | 2 | 47.2 | 668 | 681 |  | PEAKS DB |
| F.RALPC(+57.02)VNC(+57.02) | 28.73 | 988.4583 | 8 | 0.3 | 495.2365 | 2 | 36.2 | 5256 | 5263 | Carbamidomethylation | PEAKS DB |
| A.N(sub G)TGAGSGAGAGSGAGAGSGAGAGS.G | 28.24 | 1705.725 | 24 | 0 | 853.8698 | 2 | 29.7 | 2129 | 2152 |  | SPIDER |
| D.GTVAQSYVAADAGAY.S | 27.34 | 1442.668 | 15 | -0.4 | 722.3408 | 2 | 50.6 | 109 | 123 |  | PEAKS DB |
| G.AGAGTGSSGFGPY.V | 27.21 | 1127.488 | 13 | 0.4 | 564.7516 | 2 | 46.9 | 669 | 681 |  | PEAKS DB |
| G.SSGFGPYVAN(+.98)GGY.S | 26.81 | 1275.541 | 13 | 0.3 | 638.7778 | 2 | 54.3 | 675 | 687 | Deamidation (NQ) | PEAKS DB |
| A.GAGAGAGTGSSGFGPY.V | 26.8 | 1312.568 | 16 | 0.2 | 657.2916 | 2 | 47.7 | 666 | 681 |  | PEAKS DB |
| G.SGAGAGYGAGVGAGYGAGY.G | 26.76 | 1561.68 | 19 | 0.1 | 781.8472 | 2 | 49.5 | 1109 | 1127 |  | SPIDER |
| G.AGSGAGSGAGAGSGAGAGY.G | 26.72 | 1381.586 | 19 | -0.5 | 691.7998 | 2 | 34 | 2086 | 2104 |  | SPIDER |
| G.SGAGAGSGAGAGSGAGAGYGAGVGAGY.G | 26.61 | 2013.878 | 27 | 0.6 | 1007.947 | 2 | 46.6 | 1097 | 1123 |  | PEAKS DB |
| G.AGYGAGAGVGYGAGAGAGY.G | 26.41 | 1545.685 | 19 | 0.2 | 773.8499 | 2 | 49.3 | 3174 | 3192 |  | PEAKS DB |
| A.SGAGAGAGAGAGTGSSGFGPYVAN.G | 26.39 | 1939.866 | 24 | 2.3 | 970.9425 | 2 | 49.8 | 661 | 684 |  | PEAKS DB |
| A.GAGYGAGAGSGAASGAGAGAGAGAGTGSSGFGPY.V | 26.36 | 2603.127 | 34 | 0.5 | 868.7167 | 3 | 51.6 | 648 | 681 |  | PEAKS DB |
| G.SGAASGAGAGAGAGAGTGSSGF.G | 26.32 | 1624.708 | 22 | 0.7 | 813.3617 | 2 | 41 | 657 | 678 |  | PEAKS DB |
| G.SGAGAGSGAGAGYGAGVGAGY.G | 25.95 | 1613.707 | 21 | -1 | 807.8599 | 2 | 46 | 1103 | 1123 |  | PEAKS DB |
| A.GSGAGAGAGSGAGAGSGAGAGSGAGAG.S | 25.92 | 1803.773 | 27 | -0.1 | 902.8937 | 2 | 30.4 | 1070 | 1096 |  | SPIDER |
| A.SGAGAGAGAGTGSSGFGPY.V | 25.68 | 1527.659 | 19 | 0.4 | 764.837 | 2 | 48.2 | 5124 | 5142 |  | PEAKS DB |
| F.N(sub G)TGSGAGAGSGAGAGSGAGAGS.G | 25.42 | 1577.667 | 22 | 0 | 789.8405 | 2 | 29.1 | 702 | 723 |  | SPIDER |
| Q.SYVAADAGAYSQSGPYVSN.S | 25.14 | 1905.838 | 19 | 0.3 | 953.9266 | 2 | 50.1 | 114 | 132 |  | SPIDER |
| A.NINDFDEDYFGSDVTVQS.S | 25.1 | 2063.86 | 18 | -0.3 | 1032.937 | 2 | 61 | 22 | 39 |  | SPIDER |
| G.A(+27.99)GAGAGAGTGSSGFGPY.V | 25.09 | 1411.6 | 17 | -0.4 | 706.8072 | 2 | 57.1 | 665 | 681 | Formylation | PEAKS PTM |
| G.AGAGSGAGAGYGVGYGAGAGAGY.G | 25.08 | 1817.797 | 23 | 0.4 | 909.9061 | 2 | 49.6 | 2878 | 2900 |  | SPIDER |
| G.AGAGAGTGSSGFGPY.V | 24.91 | 1255.547 | 15 | 0 | 628.7807 | 2 | 47.3 | 667 | 681 |  | PEAKS DB |
| G.AGAGSGAGSGAGAGSGAGAGSGAGAGS.G | 24.62 | 1833.784 | 27 | -0.1 | 917.899 | 2 | 30.5 | 1308 | 1334 |  | SPIDER |
| G.AGAGAGAGAGTGSSGFGPYVAN.G | 24.51 | 1795.813 | 22 | -0.4 | 898.9131 | 2 | 49.7 | 663 | 684 |  | PEAKS DB |
| G.AGAGAGAGTGSSGFGPYVAN.G | 24.23 | 1667.754 | 20 | 0.2 | 834.8844 | 2 | 49.5 | 665 | 684 |  | PEAKS DB |
| G.SGAGAGSGAGAGYGAGY.G | 24 | 1329.559 | 17 | 0 | 665.7865 | 2 | 40.2 | 1358 | 1374 |  | PEAKS DB |
| A.S(+42.01)GAGAGAGAGTGSSGFGPY.V | 23.8 | 1569.67 | 19 | -0.6 | 785.8416 | 2 | 56.9 | 5124 | 5142 | Acetylation (N-term) | PEAKS PTM |
| Y.STHQGYTSDF.S | 23.77 | 1141.468 | 10 | 0.6 | 571.7415 | 2 | 37 | 136 | 145 |  | PEAKS DB |
| A.ASN(sub G)AGSGAGAGSGAGAGSGAGAGSG.A | 23.64 | 1762.747 | 25 | 0.3 | 882.3809 | 2 | 29.7 | 418 | 442 |  | SPIDER |
| W.SSESDFGTGSGAGAGSGAGAG.S | 23.39 | 1685.676 | 21 | 0.3 | 843.8457 | 2 | 38.7 | 1249 | 1269 |  | PEAKS DB |
| G.AGAGSGAGAGSGAGAGSGAGAGSGAGAG.S | 23.37 | 1874.81 | 28 | 0.1 | 938.4125 | 2 | 30.8 | 425 | 452 |  | PEAKS DB |
| G.AGSGAGAGSGAGAGSGAGAGSGAGAGSGA(-.98).G | 23.36 | 1960.858 | 29 | 0 | 981.4365 | 2 | 30.1 | 421 | 449 | Amidation | PEAKS PTM |
| S.GAGA(sub V)GSGAGAGSGAGAGSGAGAGSGAGAG.Y | 23.33 | 1931.832 | 29 | 0.4 | 966.9235 | 2 | 31.1 | 1489 | 1517 |  | SPIDER |
| G.A(+58.01)GAGAGAGTGSSGFGPY.V | 23.24 | 1441.611 | 17 | -0.3 | 721.8126 | 2 | 56.9 | 665 | 681 | Carboxymethyl (KW, X@N-term) | PEAKS PTM |
| Y.VAADAGAYSQSGPYVSNSGY.S | 23.15 | 1962.86 | 20 | -0.1 | 982.4369 | 2 | 50.1 | 116 | 135 |  | PEAKS DB |
| G.AGSGAGAGSGAGAGSGAGAGSGAGYGAGVGAGYGV(sub A)R(sub G)Y.G | 23.1 | 2889.303 | 37 | -11 | 964.0973 | 3 | 48.7 | 1262 | 1298 |  | SPIDER |
| A.N(+.98)INDFDEDYFGSDVTVQ.S | 23.05 | 1977.812 | 17 | 0 | 989.9131 | 2 | 64.1 | 22 | 38 | Deamidation (NQ) | SPIDER |
| Y.GAGAGSGAGSGAGAGSGAGAGSG.A | 22.87 | 1547.656 | 23 | -0.3 | 774.8351 | 2 | 28.9 | 1307 | 1329 |  | PEAKS DB |
| D.GTVAQSYVAADAGAYSQSGPYVSN.S | 22.86 | 2362.071 | 24 | 0.9 | 788.3651 | 3 | 53.3 | 109 | 132 |  | SPIDER |
| S.GAGAGSGAGAGSGAGAGSGAGAGSG.A | 22.68 | 1675.715 | 25 | 0.1 | 838.8646 | 2 | 29.6 | 424 | 448 |  | SPIDER |
| F.RALPC(+57.02)VN(+.98)C(+57.02) | 22.64 | 989.4423 | 8 | 0.3 | 495.7285 | 2 | 37.3 | 5256 | 5263 | Carbamidomethylation; Deamidation (NQ) | PEAKS DB |
| G.TGAGSGAGAGSGAGAGSGAGAG.S | 22.63 | 1504.65 | 22 | -0.2 | 753.3322 | 2 | 29.4 | 2130 | 2151 |  | SPIDER |
| Y.EYAWSSESDFG.T | 22.62 | 1276.488 | 11 | 0.2 | 639.2516 | 2 | 54.7 | 1245 | 1255 |  | SPIDER |
| Y.GAGAGSGAASGAGAGAGAGTGSSGFGPY.V | 22.59 | 2126.925 | 28 | 1 | 1064.471 | 2 | 49.3 | 5115 | 5142 |  | PEAKS DB |
| S.SGFGPYVAD(sub H)GGYSGYEY.A | 22.38 | 1787.731 | 17 | 0.1 | 894.8731 | 2 | 58.7 | 1230 | 1246 |  | SPIDER |
| Y.VAN(+.98)GGYSGYEY.A | 22.1 | 1179.472 | 11 | -0.1 | 590.7432 | 2 | 47.2 | 1639 | 1649 | Deamidation (NQ) | PEAKS DB |
| G.AGYGAGAGSGAASGAGAGAGAGAGTGSSGFGPY.V | 22.1 | 2546.106 | 33 | -0.2 | 849.709 | 3 | 51.6 | 649 | 681 |  | PEAKS DB |
| G.A(+27.99)GAGAGAGAGTGSSGFGPY.V | 22.02 | 1539.659 | 19 | 0.4 | 770.837 | 2 | 57.1 | 663 | 681 | Formylation | PEAKS PTM |
| G.SGAGAGSGAGAGSGAGAGSGAGAGY(-.98).G | 21.98 | 1780.772 | 25 | -0.4 | 891.3931 | 2 | 34 | 513 | 537 | Amidation | PEAKS PTM |
| G.SGAGAGYGAGVGAGYGAGAGS(+42.01)GAAF.G | 21.83 | 2001.882 | 25 | 6.2 | 1001.954 | 2 | 48.3 | 2383 | 2407 | Acetylation (TSCYH) | PEAKS PTM |
| G.SGAGAGSGAGAGSGA(sub T)GAGSGAGAGYGA.G | 21.77 | 1909.815 | 27 | 0.7 | 955.9154 | 2 | 37.1 | 459 | 485 |  | SPIDER |
| G.AGAGSGAGYGAGVGAGY.G | 21.77 | 1341.595 | 17 | -0.4 | 671.8044 | 2 | 45.1 | 1278 | 1294 |  | SPIDER |
| G.AGAGYGAGAGVGYGAGAGAGY.G | 21.76 | 1673.743 | 21 | 0.3 | 837.8792 | 2 | 49.4 | 4662 | 4682 |  | PEAKS DB |
| A.SGAGSGAGAGSGAGAGSGAGAGSGAGAGS(-.98).G | 21.7 | 1976.853 | 29 | -0.2 | 989.4337 | 2 | 29.9 | 419 | 447 | Amidation | PEAKS PTM |
| G.AGAGSGAGAGSGAGAGY.G | 21.6 | 1237.532 | 17 | -0.1 | 619.7733 | 2 | 33.2 | 521 | 537 |  | PEAKS DB |
| G.SGAGAGSGAGAGSGAGAGYGR(sub A)GA.G | 21.58 | 1722.767 | 23 | -6.9 | 862.3848 | 2 | 39.7 | 519 | 541 |  | SPIDER |
| Y.VAADAGAYSQSGPYVSN.S | 21.44 | 1655.743 | 17 | 0 | 828.8786 | 2 | 45.3 | 116 | 132 |  | PEAKS DB |
| Y.GAASGAGAGAGAGAGT(sub A)G.Y | 21.31 | 1159.522 | 17 | 0.2 | 580.7682 | 2 | 29 | 172 | 188 |  | SPIDER |
| G.AGSGAGAGAGSGAGAGSGAGAGSGAGA.G | 21.31 | 1817.789 | 27 | 0.1 | 909.9017 | 2 | 31.1 | 1069 | 1095 |  | PEAKS DB |
| F.GPYVAD(sub H)GGYSGY.E | 21.3 | 1204.504 | 12 | 0.4 | 603.2593 | 2 | 48.6 | 1233 | 1244 |  | SPIDER |
| Y.GAGAGAGYGAGAGSGAGSGAGAG.S | 21.2 | 1607.692 | 23 | -0.1 | 804.8534 | 2 | 34.9 | 1299 | 1321 |  | PEAKS DB |
| G.AASGAGAGAGAGAGTGSSGFGPYVAN.G | 21.19 | 2081.94 | 26 | 1.9 | 1041.979 | 2 | 50.1 | 659 | 684 |  | PEAKS DB |
| G.YGAGVGAGYGVGY.G | 21.15 | 1189.54 | 13 | -0.4 | 595.7772 | 2 | 51.2 | 1779 | 1791 |  | PEAKS DB |
| G.SGAGAGYGAGVGAGYGVGYGA.G | 21.14 | 1717.77 | 21 | 0.6 | 859.8926 | 2 | 52 | 1773 | 1793 |  | SPIDER |
| G.SGAGAGYGAGVGAGY.G | 21.12 | 1213.536 | 15 | -0.3 | 607.7753 | 2 | 44.9 | 1109 | 1123 |  | PEAKS DB |
| Y.GAGAGAGYGVGYGAGAGAGY.G | 21.06 | 1602.706 | 20 | -1 | 802.3596 | 2 | 49.6 | 3416 | 3435 |  | PEAKS DB |
| G.AGYGAGAGSGAGSGAGAGSGAGAG.S | 21.02 | 1694.724 | 24 | -0.2 | 848.3693 | 2 | 34.4 | 1304 | 1327 |  | PEAKS DB |
| A.GAGTGSSGFGPY.V | 21.01 | 1056.451 | 12 | 0.2 | 529.233 | 2 | 46.6 | 670 | 681 |  | PEAKS DB |
| G.AGAGAGAGAGTGSSGFGPYVANG.G | 20.98 | 1852.834 | 23 | 0.4 | 927.4246 | 2 | 49.8 | 663 | 685 |  | PEAKS DB |
| R.ALPC(+57.02)VNC(+57.02) | 20.93 | 832.3572 | 7 | -0.1 | 417.1858 | 2 | 41.9 | 5257 | 5263 | Carbamidomethylation | PEAKS DB |
| G.SGAASGAGAGAGAGAGTGS.S | 20.87 | 1333.586 | 19 | -0.4 | 667.7999 | 2 | 29.5 | 657 | 675 |  | PEAKS DB |
| F.GTGQ(sub S)GAGAGSGAGAGSGAG.A | 20.86 | 1346.581 | 19 | -1 | 674.2971 | 2 | 28.2 | 702 | 720 |  | SPIDER |
| G.SSGFGPYVAD(sub H)GGYSGYEY.A | 20.84 | 1874.763 | 18 | -0.7 | 938.3883 | 2 | 58.3 | 1229 | 1246 |  | SPIDER |
| G.TGSSGFGPYVAN(+.98)GGY.S | 20.84 | 1433.61 | 15 | -0.5 | 717.8118 | 2 | 54 | 673 | 687 | Deamidation (NQ) | PEAKS DB |
| G.YGAGAGAGYGAGAGAGY(-.98).G | 20.83 | 1388.611 | 17 | 0 | 695.3127 | 2 | 43.9 | 537 | 553 | Amidation | PEAKS PTM |
| G.AGTGSSGFGPY.V | 20.76 | 999.4297 | 11 | 0.2 | 500.7222 | 2 | 46.2 | 671 | 681 |  | PEAKS DB |
| G.AGSGAGAGAGSGAGAGSGAGAGSGAGA(-.98).G | 20.75 | 1816.805 | 27 | -1.1 | 909.4087 | 2 | 30 | 1069 | 1095 | Amidation | PEAKS PTM |
| S.GAGAGYGAGVGAGY.G | 20.64 | 1126.504 | 14 | 0.2 | 564.2595 | 2 | 44.9 | 1110 | 1123 |  | PEAKS DB |
| G.SGAGAGSGAGAGSGAGAGSGAGAGS(-.98).G | 20.56 | 1704.741 | 25 | -0.2 | 853.3777 | 2 | 28.7 | 423 | 447 | Amidation | PEAKS PTM |
| Y.GIGVGAGYGAGAGVGY.G | 20.51 | 1324.641 | 16 | -0.1 | 663.3278 | 2 | 53 | 3913 | 3928 |  | PEAKS DB |
| Y.GAGAGSGAGSGAGAGSGAGAGSGAGAGSGAG.A | 20.51 | 2075.885 | 31 | 1.8 | 1038.952 | 2 | 31.4 | 1307 | 1337 |  | PEAKS DB |
| G.SGAGAGAGSGAGAGSGAGAGSGAGAGY(-.98).G | 20.44 | 1908.831 | 27 | -0.7 | 955.4221 | 2 | 34.4 | 4475 | 4501 | Amidation | PEAKS PTM |
| W.SSESDFGTGSGAGAGSGAGA(-.98).G | 20.44 | 1627.671 | 20 | -0.2 | 814.8426 | 2 | 37.4 | 1249 | 1268 | Amidation | PEAKS PTM |
| F.R(+43.01)ALPC(+57.02)VNC(+57.02) | 20.38 | 1031.464 | 8 | -0.3 | 516.7392 | 2 | 44.6 | 5256 | 5263 | Carbamylation; Carbamidomethylation | PEAKS PTM |
| Y.GAGAGSGAGSGAGAGSGAGAGSGAG(-.98).A | 20.38 | 1674.731 | 25 | -0.5 | 838.3722 | 2 | 29.1 | 1307 | 1331 | Amidation | PEAKS PTM |
| Y.GAGVGAGYGAGYGAGAGAGY(-.98).G | 20.36 | 1601.722 | 20 | 0.2 | 801.8685 | 2 | 47.6 | 632 | 651 | Amidation | PEAKS PTM |
| Y.EYAWSSESDF.G | 20.35 | 1219.467 | 10 | -0.3 | 610.7405 | 2 | 55.6 | 1245 | 1254 |  | PEAKS DB |
| G.SGAGAGSGAGAGSGAGSGAGAGSG.A | 20.3 | 1634.688 | 24 | 0.1 | 818.3514 | 2 | 29.1 | 2555 | 2578 |  | PEAKS DB |
| G.SGAASGAGAGAGAGAGTGSSGFGPYVAN.G | 20.28 | 2225.994 | 28 | 0.3 | 743.0054 | 3 | 50 | 657 | 684 |  | PEAKS DB |
| F.ETR(sub G)SGAASGAGAGAGSGAGAGSGAGY(sub A)GSGAGAGSGY(sub A).G | 20.28 | 2818.214 | 36 | 5.9 | 940.4174 | 3 | 34.9 | 5165 | 5200 |  | SPIDER |
| G.AGSGAGAGSGAGAGSGAGSGAGAGSGQ(sub A)GAGY(+15.99).G | 20.27 | 2254.943 | 31 | 3.5 | 752.6577 | 3 | 31.2 | 2858 | 2888 | Oxidation or Hydroxylation | SPIDER |
| G.YGAGAGAGYGAGAGAGY.G | 20.23 | 1389.595 | 17 | -0.6 | 695.8043 | 2 | 46.2 | 537 | 553 |  | PEAKS DB |
| Y.GAGAGSGAGSGAGAGSGAGAG.S | 20.22 | 1403.603 | 21 | 0.2 | 702.8087 | 2 | 28.7 | 1307 | 1327 |  | PEAKS DB |
| S.GAGAGSGAGYGAGVGAGY.G | 20.15 | 1398.616 | 18 | 0.2 | 700.3156 | 2 | 45.5 | 1277 | 1294 |  | PEAKS DB |
| K.FRALPC(+57.02)VNC(+57.02) | 20.15 | 1135.527 | 9 | 0.1 | 568.7706 | 2 | 45.8 | 5255 | 5263 | Carbamidomethylation | PEAKS DB |
| S.SESDFGTGSGAGAGSGAGAG.S | 20.12 | 1598.644 | 20 | 0.1 | 800.3296 | 2 | 38.5 | 1250 | 1269 |  | PEAKS DB |
| W.SSESDFGTGSGAGA(-.98).G | 20.08 | 1227.5 | 14 | -0.5 | 614.7571 | 2 | 35 | 1249 | 1262 | Amidation | PEAKS PTM |
| W.SSESDFGTGSGAGAGSGAGAGSGA(-.98).G | 20.04 | 1899.783 | 24 | 0.2 | 950.899 | 2 | 38.3 | 1249 | 1272 | Amidation | PEAKS PTM |
| Y.VAHGGYSGY.E | 20.01 | 909.3981 | 9 | -0.2 | 455.7062 | 2 | 30.9 | 1236 | 1244 |  | PEAKS DB |
| S.SDFGTGSGAGAGSGAGAG.S | 19.98 | 1382.57 | 18 | -0.5 | 692.2919 | 2 | 36.8 | 699 | 716 |  | PEAKS DB |
| G.AGAGSGAGAGSGAGAGSGAGAGSGAGY(+15.99)GAG.V | 19.87 | 2110.89 | 30 | 4 | 704.6401 | 3 | 30.8 | 1260 | 1289 | Oxidation or Hydroxylation | SPIDER |
| G.AGYGAGAGVGYGAGAGAGY(-.98).G | 19.86 | 1544.701 | 19 | 0 | 773.3576 | 2 | 47.5 | 3174 | 3192 | Amidation | PEAKS PTM |
| G.SGAGAGAGSGAGAGSGAGAGSGAG.A | 19.84 | 1618.693 | 24 | -1.4 | 810.3527 | 2 | 29.2 | 1071 | 1094 |  | PEAKS DB |
| W.SSESDFGTGSGAGAG.S | 19.63 | 1285.506 | 15 | 0.8 | 643.7607 | 2 | 36.8 | 1249 | 1263 |  | PEAKS DB |
| G.SGAGAGSGAGAGAGSGAGAGSGAGAGS(-.98).G | 19.56 | 1832.8 | 27 | 0.1 | 917.4072 | 2 | 29.4 | 1065 | 1091 | Amidation | PEAKS PTM |
| G.SGAGAGSGAGAGYGAGVGAGYG.A | 19.55 | 1670.728 | 22 | -1.5 | 836.3702 | 2 | 45.2 | 1103 | 1124 |  | PEAKS DB |
| W.SSDFW(sub G)TGSGAGAGSGAGAG.S | 19.51 | 1598.66 | 19 | -9.3 | 800.3297 | 2 | 38.5 | 698 | 716 |  | SPIDER |
| G.AGAGAGAGTGSSGFGPYVAN(+.98)GGY.S | 19.49 | 1945.844 | 23 | 2.2 | 973.9315 | 2 | 54 | 665 | 687 | Deamidation (NQ) | PEAKS DB |
| V.GAGYGAGYGAGAGAGY.G | 19.45 | 1318.558 | 16 | 0.7 | 660.2866 | 2 | 46 | 636 | 651 |  | PEAKS DB |
| G.AGSGAGAGSGAGAGAGSGAGAGY.G | 19.44 | 1637.703 | 23 | -2.8 | 819.8564 | 2 | 35.4 | 3886 | 3908 |  | PEAKS DB |
| G.AGTGSSGFGPYVAN(+.98)GGY.S | 19.41 | 1561.669 | 17 | 0.7 | 781.842 | 2 | 53.9 | 671 | 687 | Deamidation (NQ) | PEAKS DB |
| G.YGAGAGVGYGAGAGAGY.G | 19.38 | 1417.626 | 17 | -0.1 | 709.8203 | 2 | 49.2 | 3176 | 3192 |  | PEAKS DB |
| G.AGSGAGAGAGSGAGAGSGAGAGSGAL(sub G)A.G | 19.37 | 1873.851 | 27 | -14 | 937.92 | 2 | 30.2 | 1069 | 1095 |  | SPIDER |
| G.SGAASGAGAGSGAGAGSGAGAGS(-.98).G | 19.26 | 1590.698 | 23 | -0.2 | 796.3562 | 2 | 28.8 | 497 | 519 | Amidation | PEAKS PTM |
| G.AGYGAGAGAGYGAGAGAGY(-.98).G | 19.21 | 1516.669 | 19 | -0.5 | 759.3416 | 2 | 44.5 | 535 | 553 | Amidation | PEAKS PTM |
| G.AGSGAGAGSGAGAGYGVGYGAGAGAGY(-.98).G | 19.19 | 2088.925 | 27 | 0.3 | 1045.47 | 2 | 48.3 | 2874 | 2900 | Amidation | PEAKS PTM |
| G.AGSGAGAGYGAGVGAGYGAGY.G | 19.18 | 1689.738 | 21 | -0.8 | 845.8757 | 2 | 49.5 | 1107 | 1127 |  | PEAKS DB |
| G.SGAGAGSGAGAGSGAGAGAGSGAGAGSG.A | 19.18 | 1890.805 | 28 | -15 | 946.3959 | 2 | 30.4 | 4463 | 4490 |  | PEAKS DB |
| Y.GAGYGAGAGAGYGAGAGSGAGSGAGAGSGAGAGY.G | 19.17 | 2519.07 | 34 | -1 | 840.6963 | 3 | 46.9 | 2071 | 2104 |  | PEAKS DB |
| Y.GAGYGVGAGAGY.G | 19.05 | 998.4457 | 12 | 0 | 500.2301 | 2 | 44.3 | 394 | 405 |  | PEAKS DB |
| G.YGAGYGAGAGAGY(-.98).G | 19.02 | 1132.494 | 13 | -0.6 | 567.2538 | 2 | 41.4 | 639 | 651 | Amidation | PEAKS PTM |
| Y.GVGYGAGAGAGYGAGAG.S | 19.02 | 1311.584 | 17 | -0.2 | 656.7993 | 2 | 43.2 | 1788 | 1804 |  | PEAKS DB |
| G.AGSGAASGAGAGAGAGAGTGSSGFGPYL(sub V)NGG.Y | 18.97 | 2411.074 | 31 | -0.4 | 804.6982 | 3 | 50.2 | 4163 | 4193 |  | SPIDER |
| G.AGYGAGYGAGAGAGY(-.98).G | 18.96 | 1260.552 | 15 | 0.2 | 631.2835 | 2 | 43.4 | 637 | 651 | Amidation | PEAKS PTM |
| A.NINDFDEDYFGS.D | 18.81 | 1434.558 | 12 | -0.4 | 718.2858 | 2 | 58.2 | 22 | 33 |  | SPIDER |
| V.GYGAGVGAGYGAGY.G | 18.77 | 1218.531 | 14 | -0.7 | 610.2721 | 2 | 48.8 | 630 | 643 |  | PEAKS DB |
| V.GAGYGVGYGAGVGAGY(-.98).G | 18.76 | 1373.636 | 16 | 0.3 | 687.8257 | 2 | 49.9 | 3089 | 3104 | Amidation | PEAKS PTM |
| G.AGSGAGAGSGAGAGSGAGAGSGAGA(-.98).G | 18.75 | 1688.746 | 25 | 0.1 | 845.3805 | 2 | 29.2 | 421 | 445 | Amidation | PEAKS PTM |
| G.AGAGAGAGTGSSGFGPYVANG.G | 18.72 | 1724.775 | 21 | -0.1 | 863.3949 | 2 | 49.5 | 665 | 685 |  | PEAKS DB |
| G.AGAGSGAGAGSGAGSGAGAGSGAGAGW(sub Y)GVG.Y | 18.71 | 2145.942 | 30 | -1.4 | 716.3204 | 3 | 30.9 | 2862 | 2891 |  | SPIDER |
| A.F(+15.99)GAGAGAGAGSGAGAGSGAG.A | 18.66 | 1422.612 | 20 | -0.4 | 712.3131 | 2 | 33.1 | 2407 | 2426 | Oxidation or Hydroxylation | PEAKS DB |
| G.AGYGAGVGAGYGAGAGS(+42.01)GAAF.G | 18.65 | 1729.77 | 21 | 6.1 | 865.8973 | 2 | 47.5 | 2387 | 2407 | Acetylation (TSCYH) | PEAKS PTM |
| G.SGAASGAGAGSGAGAGAGSGAGAGSGA(-.98).G | 18.63 | 1846.815 | 27 | -0.3 | 924.4147 | 2 | 30.2 | 2531 | 2557 | Amidation | PEAKS PTM |
| G.AGAGSGAASGAGAGAGAGTGSSGFGPY.V | 18.62 | 2069.904 | 27 | -0.8 | 690.9747 | 3 | 49.1 | 5116 | 5142 |  | PEAKS DB |
| G.AGAGTGSSGFGPYVAN.G | 18.62 | 1411.637 | 16 | -0.7 | 706.8251 | 2 | 48.9 | 669 | 684 |  | PEAKS DB |
| G.AGYGAGVGAGYGAGAGQ(sub V)GY.G | 18.57 | 1602.706 | 19 | -1.2 | 802.3594 | 2 | 48.5 | 3166 | 3184 |  | SPIDER |
| Q.SGPYVSNSGYSTHQ.G | 18.55 | 1482.638 | 14 | -0.5 | 742.3257 | 2 | 35.2 | 126 | 139 |  | PEAKS DB |
| Y.GAGVGAGYGAGYGAGAGAG(-.98).Y | 18.51 | 1438.659 | 19 | -1 | 720.3361 | 2 | 43 | 632 | 650 | Amidation | PEAKS PTM |
| G.SGAGAGSGAGAGYGAGVGAGYGV(-.98).G | 18.49 | 1768.813 | 23 | 1.7 | 885.4152 | 2 | 48.1 | 1767 | 1789 | Amidation | PEAKS PTM |
| G.AGAGSGAGAGSGAGAGSGAGAGS.G | 18.48 | 1561.672 | 23 | -2.4 | 781.8412 | 2 | 29.3 | 425 | 447 |  | PEAKS DB |
| T.GAGYGAGAGAGY.G | 18.44 | 970.4144 | 12 | 0.1 | 486.2145 | 2 | 39.3 | 330 | 341 |  | PEAKS DB |
| G.SGAGAGSGAGAGYGAGVGAGYGAGY.G | 18.44 | 1961.85 | 25 | -1 | 981.9315 | 2 | 49.9 | 1103 | 1127 |  | PEAKS DB |
| W.SSESDFGTGSGAGAGS(-.98).G | 18.43 | 1371.554 | 16 | -0.1 | 686.7841 | 2 | 34.9 | 1249 | 1264 | Amidation | PEAKS PTM |
| G.AGAGSGAGAGSGAGSGAGAGSGAGAGY.G | 18.37 | 1909.815 | 27 | 0.1 | 955.9148 | 2 | 36.6 | 2862 | 2888 |  | PEAKS DB |
| Y.VAN(+.98)GGYSGYE.Y | 18.37 | 1016.409 | 10 | 0 | 509.2116 | 2 | 40 | 1639 | 1648 | Deamidation (NQ) | PEAKS DB |
| A.GYGAGYGAGAGAGY(-.98).G | 18.35 | 1189.515 | 14 | -0.3 | 595.7646 | 2 | 42.7 | 638 | 651 | Amidation | PEAKS PTM |
| Y.GAGVGAGYGAGAGY.G | 18.27 | 1126.504 | 14 | 0.8 | 564.2599 | 2 | 44 | 4933 | 4946 |  | PEAKS DB |
| A.N(+.98)INDFDEDYFGSDVTVQSS.N | 18.25 | 2151.876 | 19 | 5.3 | 1076.951 | 2 | 61.5 | 22 | 40 | Deamidation (NQ) | SPIDER |
| A.SGAGAGAGAGAGTGSSGFG.P | 18.23 | 1395.601 | 19 | 0.2 | 698.8082 | 2 | 38.2 | 661 | 679 |  | PEAKS DB |
| G.SGAASGAGAGAGSGAGAGSGAGAG.S | 18.19 | 1632.709 | 24 | -0.2 | 817.3615 | 2 | 30.2 | 5168 | 5191 |  | PEAKS DB |
| G.SGAGAGSGAGAGSGAGAGSGAGAGSGAGAGYGAGVGAGY(-.98).G | 18.19 | 2813.235 | 39 | -0.4 | 938.7519 | 3 | 45.5 | 1085 | 1123 | Amidation | PEAKS PTM |
| S.GAGAGAGAGAGTGSSGF(-.98).G | 18.15 | 1250.564 | 17 | -0.2 | 626.2891 | 2 | 36.1 | 662 | 678 | Amidation | PEAKS PTM |
| N.SGYSTHQGY.T | 18.14 | 998.4094 | 9 | 0 | 500.212 | 2 | 29.9 | 133 | 141 |  | PEAKS DB |
| A.SGAGAGAGAGAGTGSSGFGPYVAN(+.98)G.G | 18.14 | 1997.872 | 25 | 0.2 | 999.9432 | 2 | 50.7 | 661 | 685 | Deamidation (NQ) | PEAKS DB |
| G.AGSGAGAGSGAGAGYGAGVGAGY.G | 18.12 | 1741.766 | 23 | 4.3 | 871.8938 | 2 | 46.6 | 1101 | 1123 |  | PEAKS DB |
| F.G(+41.03)TGSGAGAGSGAGAGSGAGAGSGAGAGSGAGAGSGAG.A | 18.11 | 2547.093 | 37 | 8 | 850.0451 | 3 | 33.2 | 702 | 738 | Amidination of lysines or N-terminal amines with methyl acetimidate | PEAKS PTM |
| G.AGAGAGTGSSGFGPYVAN.G | 18.1 | 1539.695 | 18 | -0.3 | 770.8547 | 2 | 49.6 | 667 | 684 |  | PEAKS DB |
| Y.VAHGGYSGYEY.A | 18.1 | 1201.504 | 11 | -0.2 | 601.7591 | 2 | 39.2 | 1236 | 1246 |  | PEAKS DB |
| G.VGAGYGAGAGVGYGAGAGAGY(-.98).G | 18.1 | 1700.791 | 21 | -0.2 | 851.4025 | 2 | 50.3 | 3172 | 3192 | Amidation | PEAKS PTM |
| G.SGAGAGSGAGAGSGAGAGSGAGAGSGAG(-.98).A | 18.09 | 1889.821 | 28 | -0.3 | 945.9176 | 2 | 29.8 | 423 | 450 | Amidation | PEAKS PTM |
| Y.GAGAGAGYGAGAGSGAASGAGAG.S | 18.02 | 1621.708 | 23 | 0.1 | 811.8613 | 2 | 36.2 | 484 | 506 |  | PEAKS DB |
| G.SGAASGAGAGAGAGAGTGSSGFGPYVAN(+.98)GGY.S | 18.01 | 2504.084 | 31 | 0 | 835.702 | 3 | 54 | 657 | 687 | Deamidation (NQ) | PEAKS DB |
| Y.GAGAGSGAGSGAGAGSGAGAGSGAGAGSGAGAGY(+15.99)GAGY(+15.99).G | 18 | 2747.14 | 38 | 4.4 | 916.7247 | 3 | 33.9 | 2233 | 2270 | Oxidation or Hydroxylation | PEAKS DB |
| G.YGAGAGVGYGAGAGAGY(-.98).G | 17.96 | 1416.642 | 17 | 0.2 | 709.3285 | 2 | 47.8 | 3176 | 3192 | Amidation | PEAKS PTM |
| G.SGAASGAGAGSGAGAGSGAGAGSGA(-.98).G | 17.93 | 1718.757 | 25 | -0.4 | 860.3853 | 2 | 29.5 | 497 | 521 | Amidation | PEAKS PTM |
| G.AGAGYGAGAGSGAASGAGAGAGAGAGTGSSGFGPY.V | 17.93 | 2674.164 | 35 | 1.1 | 892.3964 | 3 | 51.6 | 647 | 681 |  | PEAKS DB |
| Y.AWSSESDFGTGSGAGAGS(-.98).G | 17.93 | 1628.67 | 18 | -0.2 | 815.3423 | 2 | 46.9 | 1247 | 1264 | Amidation | PEAKS PTM |
| G.SGAGAGSGAGAGSGAGAGSGTGA(+43.04).G | 17.93 | 1634.724 | 23 | 6.9 | 818.3751 | 2 | 29 | 453 | 475 | Carboxyl modification with ethanolamine | PEAKS PTM |
| G.YGAGVGAGYGAGY.G | 17.92 | 1161.509 | 13 | 0.7 | 581.7622 | 2 | 47.9 | 631 | 643 |  | PEAKS DB |
| G.SGAGAGSGAGVGSGAGAGSGAGAGS(-.98).G | 17.85 | 1732.773 | 25 | 0.8 | 867.3942 | 2 | 31.5 | 1482 | 1506 | Amidation | PEAKS PTM |
| Y.SQSGPYVSNSGY.S | 17.82 | 1244.531 | 12 | -0.1 | 623.2726 | 2 | 42 | 124 | 135 |  | PEAKS DB |
| G.AGAGSGAASGAGAGAGAGAGTGSSGFGPYVAN.G | 17.8 | 2482.111 | 32 | 4.4 | 828.3812 | 3 | 50.3 | 653 | 684 |  | PEAKS DB |
| G.VGAGYGAGYGAGAGAGY.G | 17.8 | 1417.626 | 17 | -1.6 | 709.8193 | 2 | 48.1 | 635 | 651 |  | PEAKS DB |
| F.G(+71.04)TGSGAGAGSGAGAGSGAGAGSGAG.A | 17.78 | 1776.762 | 25 | 0.1 | 889.3884 | 2 | 30.4 | 702 | 726 | Propionamide (K, X@N-term) | PEAKS PTM |
| G.AGYGAGVGAGYGAGAGYGAGW(sub Y)GV.G | 17.76 | 1944.875 | 23 | -1.3 | 973.4437 | 2 | 47.7 | 4930 | 4952 |  | SPIDER |
| G.AGAGSGAGAGSGAGAGAGSGAGAGY(-.98).G | 17.72 | 1764.778 | 25 | 0.3 | 883.3962 | 2 | 34.4 | 3884 | 3908 | Amidation | PEAKS PTM |
| G.AGSGAASGAGAGAGAGAGTGSSGFGPYVAN.G | 17.71 | 2354.052 | 30 | -0.7 | 785.6908 | 3 | 50.1 | 655 | 684 |  | PEAKS DB |
| F.R(+43.01)(+14.02)ALPC(+57.02)VNC(+57.02) | 17.68 | 1045.48 | 8 | -0.5 | 523.7469 | 2 | 36.3 | 5256 | 5263 | Carbamylation; Methylation(KR); Carbamidomethylation | PEAKS PTM |
| A.NINDFDEDYFG.S | 17.64 | 1347.526 | 11 | 0.1 | 674.7701 | 2 | 59 | 22 | 32 |  | SPIDER |
| Y.GAGAGSGAGSGAGAGSGAGAGSGAGAG(-.98).S | 17.64 | 1802.789 | 27 | -0.4 | 902.4015 | 2 | 29.8 | 1307 | 1333 | Amidation | PEAKS PTM |
| A.Y(sub S)GAGAGAGAGSAAGSGAGAGAG.T | 17.63 | 1564.687 | 22 | -1.6 | 783.3493 | 2 | 35.7 | 283 | 304 |  | SPIDER |
| W.SSESDFGTGSGAGAGSGA(-.98).G | 17.62 | 1499.612 | 18 | 0.1 | 750.8135 | 2 | 36.3 | 1249 | 1266 | Amidation | PEAKS PTM |
| G.SGAGAGSGAGAGSGAGAGYGAGV(-.98).G | 17.57 | 1664.75 | 23 | -0.5 | 833.382 | 2 | 38.3 | 723 | 745 | Amidation | PEAKS PTM |
| Y.VAADAGAYSQ.S | 17.55 | 951.4297 | 10 | 0.1 | 476.7222 | 2 | 33.8 | 116 | 125 |  | PEAKS DB |
| G.AGYGAGVGAGYGAGY.G | 17.54 | 1289.568 | 15 | -0.2 | 645.791 | 2 | 49.2 | 1113 | 1127 |  | PEAKS DB |
| G.SGAGAGSGAGAGSGAGAGSGAGAGSGAG.A | 17.48 | 1890.805 | 28 | -0.3 | 946.4095 | 2 | 30.4 | 423 | 450 |  | PEAKS DB |
| G.AGAGAGTGSSGFGPYVANG.G | 17.47 | 1596.717 | 19 | -0.1 | 799.3656 | 2 | 49.2 | 667 | 685 |  | PEAKS DB |
| F.G(+226.08)TGSGAGAGSGAGAGSGAGAGY.G | 17.46 | 1822.754 | 22 | 14.1 | 912.3972 | 2 | 36.9 | 3817 | 3838 | Biotinylation | PEAKS PTM |
| G.AGAGAGY(+15.99)GAGAGSGAASGAGAGAGSGAGAGTGAGAGSGAGAGY(+15.99).G | 17.43 | 3102.326 | 43 | 13.9 | 1035.13 | 3 | 52.2 | 3186 | 3228 | Oxidation or Hydroxylation | PEAKS DB |
| S.SGFGPYVAN(+.98)GGY.S | 17.37 | 1188.509 | 12 | 0 | 595.2616 | 2 | 54.7 | 676 | 687 | Deamidation (NQ) | PEAKS DB |
| G.SGAGAGSGAGAGSGAGAE(sub G)F(sub S).G | 17.31 | 1437.612 | 19 | 7.4 | 719.8185 | 2 | 31.8 | 423 | 441 |  | SPIDER |
| G.AGAGSGAGAGSGAGAGSGAGAG.S | 17.31 | 1474.64 | 22 | -0.3 | 738.3268 | 2 | 29.2 | 425 | 446 |  | PEAKS DB |
| G.AGYGAGAGAGYGAGAGAGY.G | 17.31 | 1517.653 | 19 | -0.3 | 759.8337 | 2 | 46.4 | 535 | 553 |  | PEAKS DB |
| Y.GAGAGAGYGAGAGSGAASGA.G | 17.21 | 1436.628 | 20 | -1 | 719.3205 | 2 | 35.7 | 484 | 503 |  | PEAKS DB |
| Y.GAGYGAGAGAGYGAGAGSGAGS(-.98).G | 17.16 | 1641.713 | 22 | -0.4 | 821.8635 | 2 | 38.8 | 1295 | 1316 | Amidation | PEAKS PTM |
| S.GAGVGYGAGVGAGYGAGY.G | 17.15 | 1502.679 | 18 | -0.1 | 752.3467 | 2 | 51.3 | 626 | 643 |  | PEAKS DB |
| Y.G(+87.03)AGAGAGYGAGAGVGYGAGAGAGY.G | 17.12 | 1945.855 | 24 | -0.2 | 973.9348 | 2 | 49.7 | 4659 | 4682 | Glycidamide adduct | PEAKS PTM |
| G.SGAGAGSGAGAGYGAGVGA.G | 17.11 | 1393.622 | 19 | -0.5 | 697.8181 | 2 | 39.9 | 1103 | 1121 |  | PEAKS DB |
| N.SGYSTHQGYTSDF.S | 17.11 | 1448.585 | 13 | 0.8 | 725.3001 | 2 | 42.6 | 133 | 145 |  | PEAKS DB |
| Y.GAGAGSGAGSGAGAGSGAGAGSGAGAGSGAGAG.S | 17.1 | 2203.944 | 33 | -0.4 | 735.6549 | 3 | 31.9 | 1307 | 1339 |  | PEAKS DB |
| G.SAASSVSSASSR.S | 17.07 | 1095.516 | 12 | -0.6 | 548.7648 | 2 | 27.5 | 5220 | 5231 |  | PEAKS DB |
| S.GAGAGSGAGAGYGAGY.G | 17.07 | 1242.527 | 16 | -0.2 | 622.2704 | 2 | 40.1 | 1359 | 1374 |  | PEAKS DB |
| S.SGFGPYVAHGGY.S | 17.04 | 1210.541 | 12 | -0.2 | 606.2775 | 2 | 47.5 | 1230 | 1241 |  | PEAKS DB |
| G.AGAGSGAGAGYGVGYGAGAGAGY(-.98).G | 17.02 | 1816.813 | 23 | 0.1 | 909.4138 | 2 | 48 | 2878 | 2900 | Amidation | PEAKS PTM |
| S.GAGAGSGAGAGSGAGAGSGAGAG.S | 16.98 | 1531.661 | 23 | 0.1 | 766.8379 | 2 | 29.5 | 424 | 446 |  | PEAKS DB |
| A.GAGSGAGSGAGAGSGAGAGSGAGAGSGAG.A | 16.95 | 1947.827 | 29 | 0.4 | 974.921 | 2 | 30.6 | 1309 | 1337 |  | PEAKS DB |
| A.GAGSGAGAGSGAGAGSGAGAGSGAGAGGSVSY(+15.99).G | 16.94 | 2312.985 | 32 | -13 | 771.9924 | 3 | 32.1 | 5177 | 5208 | Oxidation or Hydroxylation | PEAKS DB |
| Y.GAGVGAGYGVGYGAGAGAGY(-.98).G | 16.94 | 1629.754 | 20 | 1 | 815.8848 | 2 | 49.9 | 1780 | 1799 | Amidation | PEAKS PTM |
| Y.GAGVGAGYGAGAGSGAGSGAGAGSGAGAGS(-18.01).G | 16.92 | 2104.916 | 30 | -3 | 1053.462 | 2 | 30.6 | 3312 | 3341 | Dehydration | PEAKS PTM |
| A.GVGYGAGVGAGYGAG.Y | 16.91 | 1211.557 | 15 | 0.1 | 606.7858 | 2 | 46.7 | 628 | 642 |  | PEAKS DB |
| G.YGAGVGAGYGAGAGY(-.98).G | 16.89 | 1288.584 | 15 | 0 | 645.2991 | 2 | 45.9 | 4932 | 4946 | Amidation | PEAKS PTM |
| G.SGAGAGYGAGAGAGY.G | 16.87 | 1185.505 | 15 | -0.2 | 593.7596 | 2 | 39.7 | 477 | 491 |  | PEAKS DB |
| Y.GAGAGSGAGSGAGAGSGAGAGYGAGAGAGYGAGY.G | 16.86 | 2519.07 | 34 | 11.3 | 840.7067 | 3 | 32.6 | 2083 | 2116 |  | PEAKS DB |
| G.SGAGAGAGSGAGAGSGAGAGSGA(-.98).G | 16.82 | 1560.688 | 23 | 0 | 781.3511 | 2 | 28.6 | 1071 | 1093 | Amidation | PEAKS PTM |
| G.AGSGAGAGSGAGSGAGAGSGAGAGSGAGAGSGAGAGY.G | 16.82 | 2582.098 | 37 | -1.6 | 861.7051 | 3 | 39.1 | 2920 | 2956 |  | PEAKS DB |
| S.G(+163.05)AGAGYGAGAGAGYGAGAGSGAG.S | 16.81 | 1846.769 | 23 | 10.2 | 924.4012 | 2 | 45.7 | 1985 | 2007 | Phenethyl isothiocyanate | PEAKS PTM |
| Y.GAGVGVGYGAGYGAGAGAGY.G | 16.81 | 1630.738 | 20 | 0.9 | 816.3768 | 2 | 51.3 | 742 | 761 |  | PEAKS DB |
| G.YGAGAGSGAASGAGAGAGAGAGTGSSGFGPY.V | 16.8 | 2418.047 | 31 | -1.2 | 807.022 | 3 | 51.1 | 651 | 681 |  | PEAKS DB |
| G.AGVGAGYGVGYGAGAGAGY(-.98).G | 16.8 | 1572.732 | 19 | -0.3 | 787.373 | 2 | 50.2 | 1781 | 1799 | Amidation | PEAKS PTM |
| Y.GAGAGSGAASGAGAGAG.A | 16.77 | 1145.506 | 17 | -0.4 | 573.7601 | 2 | 28.5 | 652 | 668 |  | PEAKS DB |
| G.SGAGAGYGAGVGAGYGVGY(-.98).G | 16.76 | 1588.727 | 19 | -1 | 795.3699 | 2 | 50.6 | 1773 | 1791 | Amidation | PEAKS PTM |
| G.AGAGYGAGAGSGAGSGAGAGSGA(-.98).G | 16.73 | 1636.719 | 23 | 2.7 | 819.3689 | 2 | 32.9 | 1302 | 1324 | Amidation | PEAKS PTM |
| A.GAGYGAGAGAGYGAGA.G | 16.7 | 1226.532 | 16 | -0.2 | 614.2729 | 2 | 40.5 | 480 | 495 |  | PEAKS DB |
| Y.GAGAGSGAASGAGAGAGAGAGTGSSGFGPYVAN.G | 16.68 | 2539.132 | 33 | 0.3 | 847.385 | 3 | 50.3 | 652 | 684 |  | PEAKS DB |
| G.AGSGAGAGSGAGAGSGAGAGYGAGV(-.98).G | 16.68 | 1792.809 | 25 | 0.8 | 897.4124 | 2 | 38.7 | 721 | 745 | Amidation | PEAKS PTM |
| F.G(+41.03)TGSGAGAGSGAGAGSGAGAGSGAGAGSGAGAGSGAGAGY.G | 16.66 | 2838.215 | 40 | 5.3 | 947.0839 | 3 | 39.8 | 702 | 741 | Amidination of lysines or N-terminal amines with methyl acetimidate | PEAKS PTM |
| G.AGAGAGAGTGSSGFGPYVAN(+.98)G.G | 16.6 | 1725.759 | 21 | -0.4 | 863.8866 | 2 | 50.6 | 665 | 685 | Deamidation (NQ) | PEAKS DB |
| F.GTGSGAGAGSGAGAGSGAGAG.S | 16.52 | 1433.613 | 21 | -0.7 | 717.8133 | 2 | 28.7 | 702 | 722 |  | PEAKS DB |
| D.GTVAQSYVAADAGAYSQ.S | 16.5 | 1657.758 | 17 | 0.2 | 829.8866 | 2 | 49.6 | 109 | 125 |  | PEAKS DB |
| G.SGAGVGYGAGVGAGY.G | 16.49 | 1241.568 | 15 | 0 | 621.7911 | 2 | 48.2 | 625 | 639 |  | PEAKS DB |
| G.AGSGAGAGAGSGAGAGSGAGAGSGAGAGYG(+15.99).A | 16.48 | 2110.89 | 30 | 4.1 | 1056.457 | 2 | 30.9 | 4473 | 4502 | Oxidation or Hydroxylation | PEAKS DB |
| G.SGAGAGSGAGVGYGAGV(-.98).G | 16.46 | 1292.611 | 17 | 0 | 647.3127 | 2 | 40.5 | 619 | 635 | Amidation | PEAKS PTM |
| G.SGAGAGSGAGAGSGAGAGAGSGAGAGSGA(-.98).G | 16.45 | 1960.858 | 29 | -1.8 | 981.4346 | 2 | 31 | 4463 | 4491 | Amidation | PEAKS PTM |
| A.GSGAGAGAGSGAGAGY.G | 16.41 | 1166.495 | 16 | 0.3 | 584.255 | 2 | 32.7 | 3893 | 3908 |  | PEAKS DB |
| G.AGSGAGAGSGAGSGAGAGSGAGAGSGAGAGSGAGSGAG.A | 16.41 | 2563.088 | 38 | -0.1 | 855.3698 | 3 | 33 | 2842 | 2879 |  | PEAKS DB |
| N.SGYSTHQGYTSDFSTS.A | 16.39 | 1723.696 | 16 | -0.3 | 862.8551 | 2 | 42.2 | 133 | 148 |  | SPIDER |
| G.AGYGAGAGAGYGAGY.G | 16.34 | 1261.536 | 15 | -0.9 | 631.7748 | 2 | 46.1 | 383 | 397 |  | PEAKS DB |
| Y.G(+163.05)AGAGVGYGAGAGAGYGAGAG.S | 16.32 | 1730.747 | 21 | 9.9 | 866.3893 | 2 | 48.9 | 3177 | 3197 | Phenethyl isothiocyanate | PEAKS PTM |
| G.AGAGSGAGVGSGAGAGSGAGAGSGA(-.98).G | 16.32 | 1716.778 | 25 | -0.7 | 859.3954 | 2 | 32 | 1484 | 1508 | Amidation | PEAKS PTM |
| G.SGAGAGAGSGAGAGY(-.98).G | 16.3 | 1108.49 | 15 | -0.4 | 555.252 | 2 | 29.9 | 3894 | 3908 | Amidation | PEAKS PTM |
| Y.GAGYGAGVGAGYGAGAGY(-.98).G | 16.3 | 1473.664 | 18 | -0.3 | 737.8388 | 2 | 47.1 | 4929 | 4946 | Amidation | PEAKS PTM |
| A.GAGYGAGAGAGYGAGAG.S | 16.3 | 1283.553 | 17 | -0.7 | 642.7833 | 2 | 39.8 | 480 | 496 |  | PEAKS DB |
| Y.GAAV(sub S)GTGAGYGAGAGAGY.G | 16.29 | 1426.648 | 18 | -0.7 | 714.3306 | 2 | 48.8 | 324 | 341 |  | SPIDER |
| G.SGAASGAGAGAGAGAGTGSS(-.98).G | 16.29 | 1419.634 | 20 | 0.1 | 710.8242 | 2 | 28.7 | 657 | 676 | Amidation | PEAKS PTM |
| G.AGYGAGVGAGYGVGY(-.98).G | 16.28 | 1316.615 | 15 | 0 | 659.3147 | 2 | 50.2 | 1777 | 1791 | Amidation | PEAKS PTM |
| G.AGSGAGSGAGAGSGAGAGYGAG.A | 16.26 | 1566.666 | 22 | -0.8 | 784.3395 | 2 | 34.3 | 2086 | 2107 |  | PEAKS DB |
| G.SGAGAGSGAGAGSGAGAGSGAGAGYGAGVGAGY(-.98).G | 16.21 | 2413.064 | 33 | -1.8 | 805.3606 | 3 | 45 | 1091 | 1123 | Amidation | PEAKS PTM |
| A.GAGYGAGAGSGAGSGAGAG.S | 16.2 | 1351.575 | 19 | -1 | 676.7942 | 2 | 32.5 | 1303 | 1321 |  | PEAKS DB |
| Y.GAGAGAGYGAGAGSGAG.S | 16.18 | 1207.522 | 17 | -0.1 | 604.7681 | 2 | 32.6 | 1299 | 1315 |  | PEAKS DB |
| Y.GGASGAGAGAGAGAGAGAGAGY(-2.02).G | 16.13 | 1532.66 | 22 | 2.5 | 767.3394 | 2 | 44.5 | 342 | 363 | 2-amino-3-oxo-butanoic_acid | PEAKS PTM |
| G.SGAGAGSGAGSGAGAGSGAGAGSG(-.98).A | 16.11 | 1633.704 | 24 | -0.5 | 817.8588 | 2 | 28.4 | 2561 | 2584 | Amidation | PEAKS PTM |
| Y.GAGAGSGAGSGAGAGSGAGAGSGAGAGSGAGAGSGAGAGY(-.98).G | 16.11 | 2766.194 | 40 | 2 | 923.0737 | 3 | 37.9 | 3320 | 3359 | Amidation | PEAKS PTM |
| Y.AWSSESDFGTG.S | 16.11 | 1142.452 | 11 | -0.6 | 572.2328 | 2 | 48.4 | 1247 | 1257 |  | PEAKS DB |
| **CHYMOTRYPSIN-TRYPSIN** |  |  |  |  |  |  |  |  |  |  |  |
| **Peptide** | **-10lgP** | **Mass** | **Length** | **ppm** | **m/z** | **z** | **RT** | **Start** | **End** | **PTM** | **Found By** |
| G.AGAGAGAGTGSSGFGPY.V | 42.48 | 1383.606 | 17 | -0.5 | 692.8096 | 2 | 47.8 | 665 | 681 |  | PEAKS DB |
| A.SGAGAGAGAGAGTGSSGFGPY.V | 42.09 | 1655.718 | 21 | 0 | 828.866 | 2 | 48.4 | 661 | 681 |  | PEAKS DB |
| F.RALPC(+57.02)VNC(+57.02) | 41.55 | 988.4583 | 8 | 0.3 | 495.2365 | 2 | 36.2 | 5256 | 5263 | Carbamidomethylation | PEAKS DB |
| G.SGAASGAGAGAGAGAGTGSSGFGPY.V | 41.16 | 1941.845 | 25 | 0 | 971.9299 | 2 | 48.8 | 657 | 681 |  | PEAKS DB |
| D.GTVAQSYVAADAGAY.S | 40.68 | 1442.668 | 15 | -0.4 | 722.3408 | 2 | 50.6 | 109 | 123 |  | PEAKS DB |
| F.RALPC(+57.02)VN(+.98)C(+57.02) | 40.67 | 989.4423 | 8 | 0.3 | 495.7285 | 2 | 37.3 | 5256 | 5263 | Carbamidomethylation; Deamidation (NQ) | PEAKS DB |
| R.ALPC(+57.02)VNC(+57.02) | 39.9 | 832.3572 | 7 | -0.1 | 417.1858 | 2 | 41.9 | 5257 | 5263 | Carbamidomethylation | PEAKS DB |
| G.AGAGAGTGSSGFGPY.V | 38.27 | 1255.547 | 15 | 0 | 628.7807 | 2 | 47.3 | 667 | 681 |  | PEAKS DB |
| Y.VAHGGYSGY.E | 37.67 | 909.3981 | 9 | -0.2 | 455.7062 | 2 | 30.9 | 1236 | 1244 |  | PEAKS DB |
| A.GAGAGAGTGSSGFGPY.V | 37.16 | 1312.568 | 16 | 0.2 | 657.2916 | 2 | 47.7 | 666 | 681 |  | PEAKS DB |
| G.SAASSVSSASSR.S | 36.85 | 1095.516 | 12 | -0.6 | 548.7648 | 2 | 27.5 | 5220 | 5231 |  | PEAKS DB |
| A.GAGTGSSGFGPY.V | 36.85 | 1056.451 | 12 | 0.2 | 529.233 | 2 | 46.6 | 670 | 681 |  | PEAKS DB |
| A.GAGAGAGAGTGSSGFGPY.V | 36.67 | 1440.627 | 18 | 0.4 | 721.321 | 2 | 48 | 664 | 681 |  | PEAKS DB |
| F.R(+43.01)ALPC(+57.02)VNC(+57.02) | 36.46 | 1031.464 | 8 | -0.3 | 516.7392 | 2 | 44.6 | 5256 | 5263 | Carbamylation; Carbamidomethylation | PEAKS PTM |
| A.GAGAGTGSSGFGPY.V | 36.36 | 1184.51 | 14 | 0.1 | 593.2622 | 2 | 47.2 | 668 | 681 |  | PEAKS DB |
| G.AASGAGAGAGAGAGTGSSGFGPY.V | 35.93 | 1797.792 | 23 | 0.5 | 899.9036 | 2 | 48.7 | 659 | 681 |  | PEAKS DB |
| G.AGAGAGAGAGTGSSGFGPY.V | 35.71 | 1511.664 | 19 | -0.2 | 756.8392 | 2 | 48.2 | 663 | 681 |  | PEAKS DB |
| F.R(+57.02)ALPC(+57.02)VNC(+57.02) | 35.36 | 1045.48 | 8 | -0.5 | 523.7469 | 2 | 36.3 | 5256 | 5263 | Carbamidomethylation (DHKE, X@N-term); Carbamidomethylation | PEAKS PTM |
| G.AGAGTGSSGFGPY.V | 35.16 | 1127.488 | 13 | 0.4 | 564.7516 | 2 | 46.9 | 669 | 681 |  | PEAKS DB |
| G.SGAGAGSGAGAGYGAGY.G | 34 | 1329.559 | 17 | 0 | 665.7865 | 2 | 40.2 | 1358 | 1374 |  | PEAKS DB |
| G.SGAGAGSGAGAGYGAGVGAGY.G | 33.9 | 1613.707 | 21 | -1 | 807.8599 | 2 | 46 | 1103 | 1123 |  | PEAKS DB |
| Y.STHQGYTSDF.S | 33.04 | 1141.468 | 10 | 0.6 | 571.7415 | 2 | 37 | 136 | 145 |  | PEAKS DB |
| S.GAGAGYGAGVGAGY.G | 32.7 | 1126.504 | 14 | 0.2 | 564.2595 | 2 | 44.9 | 1110 | 1123 |  | PEAKS DB |
| G.A(+27.99)GAGAGAGTGSSGFGPY.V | 32.41 | 1411.6 | 17 | -0.4 | 706.8072 | 2 | 57.1 | 665 | 681 | Formylation | PEAKS PTM |
| G.AGTGSSGFGPY.V | 32.35 | 999.4297 | 11 | 0.2 | 500.7222 | 2 | 46.2 | 671 | 681 |  | PEAKS DB |
| Y.VAADAGAYSQ.S | 32.23 | 951.4297 | 10 | 0.1 | 476.7222 | 2 | 33.8 | 116 | 125 |  | PEAKS DB |
| N.SGYSTHQGY.T | 31.9 | 998.4094 | 9 | 0 | 500.212 | 2 | 29.9 | 133 | 141 |  | PEAKS DB |
| A.S(+42.01)GAGAGAGAGTGSSGFGPY.V | 31.65 | 1569.67 | 19 | -0.6 | 785.8416 | 2 | 56.9 | 5124 | 5142 | Acetylation (N-term) | PEAKS PTM |
| W.SSESDFGTGSGAGAGSGAGAG.S | 31.53 | 1685.676 | 21 | 0.3 | 843.8457 | 2 | 38.7 | 1249 | 1269 |  | PEAKS DB |
| G.SSGFGPYVAN(+.98)GGY.S | 31.51 | 1275.541 | 13 | 0.3 | 638.7778 | 2 | 54.3 | 675 | 687 | Deamidation (NQ) | SPIDER |
| N.SGYSTHQGYTSDF.S | 31.07 | 1448.585 | 13 | 0.8 | 725.3001 | 2 | 42.6 | 133 | 145 |  | SPIDER |
| Y.G(+163.05)AGAGVGYGAGAGAGY.G | 31.06 | 1417.608 | 16 | 12.5 | 709.8203 | 2 | 49.2 | 3177 | 3192 | Phenethyl isothiocyanate | PEAKS PTM |
| S.SDFGTGSGAGAGSGAGAG.S | 31.02 | 1382.57 | 18 | -0.5 | 692.2919 | 2 | 36.8 | 699 | 716 |  | PEAKS DB |
| Y.GAGAGAGYGAGAGSGAGSGAGAG.S | 30.11 | 1607.692 | 23 | -0.1 | 804.8534 | 2 | 34.9 | 1299 | 1321 |  | PEAKS DB |
| G.AGYGAGAGAGAGAGY.G | 30.1 | 1169.51 | 15 | -0.1 | 585.7623 | 2 | 40.4 | 199 | 213 |  | PEAKS DB |
| G.SGAGAGSGAGAGSGAGAGYGAGVGAGY.G | 29.97 | 2013.878 | 27 | 0.6 | 1007.947 | 2 | 46.6 | 1097 | 1123 |  | PEAKS DB |
| G.AGSGAGYGAGVGAGY.G | 29.97 | 1213.536 | 15 | -0.3 | 607.7753 | 2 | 44.9 | 1280 | 1294 |  | PEAKS DB |
| G.AGVGAGYGAGAGY.G | 29.97 | 1069.483 | 13 | 0.1 | 535.7487 | 2 | 43.8 | 4934 | 4946 |  | PEAKS DB |
| A.SGAGAGAGAGTGSSGFGPY.V | 29.87 | 1527.659 | 19 | 0.4 | 764.837 | 2 | 48.2 | 5124 | 5142 |  | PEAKS DB |
| W.SSESDFGTGSGAGAG.S | 29.63 | 1285.506 | 15 | 0.8 | 643.7607 | 2 | 36.8 | 1249 | 1263 |  | PEAKS DB |
| Y.GAGAGAGYGAGAGSGAG.S | 29.51 | 1207.522 | 17 | -0.1 | 604.7681 | 2 | 32.6 | 1299 | 1315 |  | PEAKS DB |
| G.TGSSGFGPYVAN(+.98)GGYSGY.E | 29.47 | 1740.727 | 18 | -6 | 871.3654 | 2 | 55.9 | 1630 | 1647 | Deamidation (NQ) | SPIDER |
| A.GAGYGAGAGAGAGAGY.G | 29.35 | 1226.532 | 16 | -0.2 | 614.2729 | 2 | 40.5 | 198 | 213 |  | PEAKS DB |
| A.SGAGAGAGAGAGTGSSGFG.P | 28.95 | 1395.601 | 19 | 0.2 | 698.8082 | 2 | 38.2 | 661 | 679 |  | PEAKS DB |
| Y.VAADAGAYSQSGPYVSN.S | 28.78 | 1655.743 | 17 | 0 | 828.8786 | 2 | 45.3 | 116 | 132 |  | SPIDER |
| G.AGAGSGAGAGSGAGAGSGAGAGSGAGAG.S | 28.67 | 1874.81 | 28 | 0.1 | 938.4125 | 2 | 30.8 | 425 | 452 |  | PEAKS DB |
| G.AGAGSGAGAGSGAGAGY.G | 28.54 | 1237.532 | 17 | -0.1 | 619.7733 | 2 | 33.2 | 521 | 537 |  | PEAKS DB |
| S.GAGAGSGAGYGAGVGAGY.G | 28.51 | 1398.616 | 18 | 0.2 | 700.3156 | 2 | 45.5 | 1277 | 1294 |  | PEAKS DB |
| G.AGAGSGAGAGYGAGVGAGY.G | 28.51 | 1469.653 | 19 | -0.4 | 735.8337 | 2 | 46 | 1105 | 1123 |  | PEAKS DB |
| G.AGSGAGAGYGAGVGAGY.G | 28.44 | 1341.595 | 17 | -0.4 | 671.8044 | 2 | 45.1 | 1107 | 1123 |  | PEAKS DB |
| S.SESDFGTGSGAGAGSGAGAG.S | 28.37 | 1598.644 | 20 | 0.1 | 800.3296 | 2 | 38.5 | 1250 | 1269 |  | PEAKS DB |
| G.AGSGAGSGAGAGSGAGAGY.G | 28.3 | 1381.586 | 19 | -0.5 | 691.7998 | 2 | 34 | 2086 | 2104 |  | PEAKS DB |
| Y.SQSGPYVSNSGY.S | 28.3 | 1244.531 | 12 | -0.1 | 623.2726 | 2 | 42 | 124 | 135 |  | PEAKS DB |
| Y.EYAWSSESDFGTGS.G | 28.29 | 1521.59 | 14 | -0.6 | 761.8016 | 2 | 54.3 | 1245 | 1258 |  | SPIDER |
| Y.GAGVGAGYGAGAGY.G | 28.23 | 1126.504 | 14 | 0.8 | 564.2599 | 2 | 44 | 4933 | 4946 |  | PEAKS DB |
| G.SGAASGAGAGAGAGAGTGS.S | 28.12 | 1333.586 | 19 | -0.4 | 667.7999 | 2 | 29.5 | 657 | 675 |  | PEAKS DB |
| A.NINDFDEDY.F | 27.97 | 1143.436 | 9 | -0.1 | 572.725 | 2 | 51.4 | 22 | 30 |  | PEAKS DB |
| T.GAGYGAGAGAGY.G | 27.85 | 970.4144 | 12 | 0.1 | 486.2145 | 2 | 39.3 | 330 | 341 |  | PEAKS DB |
| Y.SQSGPYVSN.S | 27.66 | 937.4141 | 9 | -0.4 | 469.7142 | 2 | 31.4 | 124 | 132 |  | PEAKS DB |
| G.SGAASGAGAGAGAGAGTGSSGF.G | 27.51 | 1624.708 | 22 | 0.7 | 813.3617 | 2 | 41 | 657 | 678 |  | PEAKS DB |
| T.GAGYGAGAGAGY(-.98).G | 27.14 | 969.4304 | 12 | -0.1 | 485.7224 | 2 | 36.2 | 330 | 341 | Amidation | PEAKS PTM |
| G.AGAGVGYGAGAGAGY(-.98).G | 27.1 | 1196.557 | 15 | 0.1 | 599.286 | 2 | 41.9 | 3178 | 3192 | Amidation | PEAKS PTM |
| G.SGAGAGYGAGAGSGAA.S | 27.02 | 1180.511 | 16 | -0.3 | 591.2625 | 2 | 33 | 2583 | 2598 |  | PEAKS DB |
| G.SAASSVSSASSR(-.98).S | 27.02 | 1094.532 | 12 | -0.2 | 548.2729 | 2 | 27.4 | 5220 | 5231 | Amidation | PEAKS PTM |
| G.SSGFGPYVAH.G | 26.94 | 1020.466 | 10 | 0.3 | 511.2407 | 2 | 41.9 | 1229 | 1238 |  | SPIDER |
| Y.GAGAGSGAGSGAGAGSGAGAG.S | 26.81 | 1403.603 | 21 | 0.2 | 702.8087 | 2 | 28.7 | 1307 | 1327 |  | PEAKS DB |
| A.GYGAGAGAGAGAGY.G | 26.8 | 1098.473 | 14 | 0.1 | 550.2438 | 2 | 39.4 | 200 | 213 |  | PEAKS DB |
| F.R(+27.99)ALPC(+57.02)VNC(+57.02) | 26.76 | 1016.453 | 8 | -0.2 | 509.2338 | 2 | 45.3 | 5256 | 5263 | Formylation; Carbamidomethylation | PEAKS PTM |
| G.A(+27.99)GAGAGAGAGTGSSGFGPY.V | 26.71 | 1539.659 | 19 | 0.4 | 770.837 | 2 | 57.1 | 663 | 681 | Formylation | PEAKS PTM |
| G.AGSGAGSGAGAGSGAGAGSGAGAGY.G | 26.67 | 1781.756 | 25 | 0.1 | 891.8855 | 2 | 36.2 | 2565 | 2589 |  | PEAKS DB |
| Y.GAGAGSGAGSGAGAGSGAGAGSGAG.A | 26.63 | 1675.715 | 25 | 0.1 | 838.8646 | 2 | 29.6 | 1307 | 1331 |  | PEAKS DB |
| A.VIEEQITTK.K | 26.61 | 1059.581 | 9 | 0.1 | 530.7979 | 2 | 34.9 | 54 | 62 |  | PEAKS DB |
| S.GAASGAGAGAGAGAGTGSSGFGPY.V | 26.49 | 1854.813 | 24 | 0.1 | 928.414 | 2 | 49.1 | 658 | 681 |  | PEAKS DB |
| G.SGAGAGSGAGAGSGAGAGSGAGSGA.G | 26.33 | 1705.725 | 25 | 0 | 853.8698 | 2 | 29.7 | 2549 | 2573 |  | PEAKS DB |
| S.GAGAGSGAGAGSGAGAGSGAGAG.S | 26.23 | 1531.661 | 23 | 0.1 | 766.8379 | 2 | 29.5 | 424 | 446 |  | PEAKS DB |
| F.RALPC(+57.02)VN(-17.03)C(+57.02) | 26.17 | 971.4317 | 8 | -0.6 | 486.7228 | 2 | 41 | 5256 | 5263 | Carbamidomethylation; Ammonia-loss (N) | PEAKS PTM |
| Y.GAGAGSGAGSGAGAGSGAGAGSG.A | 26.1 | 1547.656 | 23 | -0.3 | 774.8351 | 2 | 28.9 | 1307 | 1329 |  | PEAKS DB |
| G.AGSGAASGAGAGAGAGAGTGSSGFGPY.V | 26 | 2069.904 | 27 | -0.5 | 1035.959 | 2 | 49 | 655 | 681 |  | PEAKS DB |
| S.GAGAGSGAGAGYGAGY.G | 25.97 | 1242.527 | 16 | -0.2 | 622.2704 | 2 | 40.1 | 1359 | 1374 |  | PEAKS DB |
| G.AGVGAGYGVGY(-.98).G | 25.88 | 968.4716 | 11 | -0.3 | 485.2429 | 2 | 45.2 | 1781 | 1791 | Amidation | PEAKS PTM |
| A.GAGAGAGAGTGSSGF(-.98).G | 25.78 | 1122.505 | 15 | -0.3 | 562.2598 | 2 | 34.7 | 664 | 678 | Amidation | PEAKS PTM |
| Y.GIGVGAGYGAGAGVGY.G | 25.71 | 1324.641 | 16 | -0.1 | 663.3278 | 2 | 53 | 3913 | 3928 |  | PEAKS DB |
| G.SGAGAGYGAGAGAGY.G | 25.65 | 1185.505 | 15 | -0.2 | 593.7596 | 2 | 39.7 | 477 | 491 |  | PEAKS DB |
| A.A(+57.02)SGAGAGSGAGAGSGAGAGSGAG.A | 25.54 | 1618.693 | 23 | -1.4 | 810.3527 | 2 | 29.2 | 500 | 522 | Carbamidomethylation (DHKE, X@N-term) | PEAKS PTM |
| R.ALPC(+57.02)VN(-17.03)C(+57.02) | 25.47 | 815.3306 | 7 | 0.5 | 408.6728 | 2 | 42.1 | 5257 | 5263 | Carbamidomethylation; Ammonia-loss (N) | PEAKS PTM |
| S.GAGVGYGAGVGAGY.G | 25.41 | 1154.536 | 14 | -0.2 | 578.275 | 2 | 47.9 | 626 | 639 |  | PEAKS DB |
| G.SGAGAGSGAGAGSGAGAGYGAGVG.V | 25.36 | 1722.756 | 24 | -0.4 | 862.3848 | 2 | 39.7 | 723 | 746 |  | PEAKS DB |
| G.SGAGAGSGAGAGSGAGAGSGAGSGAG.A | 25.33 | 1762.747 | 26 | 0.3 | 882.3809 | 2 | 29.7 | 2549 | 2574 |  | PEAKS DB |
| G.A(+58.01)GAGAGTGSSGFGPY.V | 25.33 | 1313.552 | 15 | 0 | 657.7834 | 2 | 56.9 | 667 | 681 | Carboxymethyl (KW, X@N-term) | PEAKS PTM |
| W.SSESDFGTGSGAGA(-.98).G | 25.3 | 1227.5 | 14 | -0.5 | 614.7571 | 2 | 35 | 1249 | 1262 | Amidation | PEAKS PTM |
| F.G(+57.02)TGSGAGAGSGAGAGSGAGAGS.G | 25.02 | 1577.667 | 22 | 0 | 789.8405 | 2 | 29.1 | 702 | 723 | Carbamidomethylation (DHKE, X@N-term) | PEAKS PTM |
| Y.EYAWSSESDF.G | 24.96 | 1219.467 | 10 | -0.3 | 610.7405 | 2 | 55.6 | 1245 | 1254 |  | SPIDER |
| A.GAGYGAGAGSGAGSGAGAG.S | 24.95 | 1351.575 | 19 | -1 | 676.7942 | 2 | 32.5 | 1303 | 1321 |  | PEAKS DB |
| F.RALPC(+57.02)VN.C | 24.94 | 828.4276 | 7 | 0.1 | 415.2211 | 2 | 33.1 | 5256 | 5262 | Carbamidomethylation | PEAKS DB |
| Y.GAGAGAGYGAGAGSGAA.S | 24.84 | 1221.537 | 17 | -0.2 | 611.7758 | 2 | 34 | 484 | 500 |  | PEAKS DB |
| F.G(+71.04)TGSGAGAGSGAGAGSGAGAG.S | 24.74 | 1504.65 | 21 | -0.2 | 753.3322 | 2 | 29.4 | 702 | 722 | Propionamide (K, X@N-term) | PEAKS PTM |
| Y.GAGAGVGYGAGAGAGY.G | 24.66 | 1254.563 | 16 | -0.2 | 628.2886 | 2 | 44.4 | 3177 | 3192 |  | PEAKS DB |
| G.AGAGYGAGAGSGAASGA.G | 24.59 | 1251.548 | 17 | 0.1 | 626.7813 | 2 | 33.8 | 487 | 503 |  | PEAKS DB |
| W.SSESDFGTGSGAGAGS(-.98).G | 24.48 | 1371.554 | 16 | -0.1 | 686.7841 | 2 | 34.9 | 1249 | 1264 | Amidation | PEAKS PTM |
| W.SSESDFGTGSGAGAGSGAGA(-.98).G | 24.43 | 1627.671 | 20 | -0.2 | 814.8426 | 2 | 37.4 | 1249 | 1268 | Amidation | PEAKS PTM |
| G.SGAGAGYGAGVGAGYGVGY.G | 24.38 | 1589.711 | 19 | 0.3 | 795.863 | 2 | 52.3 | 1773 | 1791 |  | SPIDER |
| Y.GAGYGVGAGAGY.G | 24.34 | 998.4457 | 12 | 0 | 500.2301 | 2 | 44.3 | 394 | 405 |  | PEAKS DB |
| Y.GAGAGAGYGAGAGSGAASGA.G | 24.32 | 1436.628 | 20 | -1 | 719.3205 | 2 | 35.7 | 484 | 503 |  | PEAKS DB |
| G.SGTGSGAGAGSGAGAGSGAGAG.S | 24.25 | 1520.645 | 22 | 0.2 | 761.33 | 2 | 29 | 3365 | 3386 |  | PEAKS DB |
| G.AGSGAGAGSGAGSGAGAGSGAGAGSGAG.A | 24.24 | 1890.805 | 28 | -0.3 | 946.4095 | 2 | 30.4 | 2559 | 2586 |  | PEAKS DB |
| G.AGSGAGSGAGAGSGAGAGYG.A | 24.05 | 1438.607 | 20 | -0.1 | 720.3108 | 2 | 33.2 | 2086 | 2105 |  | PEAKS DB |
| G.SVSYGAGRGYGQGAG.S | 24.04 | 1385.632 | 15 | 0 | 693.8234 | 2 | 34.3 | 5205 | 5219 |  | SPIDER |
| Y.GAGAGAGYGAGAGSG.A | 24.01 | 1079.463 | 15 | -0.1 | 540.7388 | 2 | 31.4 | 484 | 498 |  | PEAKS DB |
| Y.GAGAGAGYGAGAGSGAASGAGAG.S | 24.01 | 1621.708 | 23 | 0.1 | 811.8613 | 2 | 36.2 | 484 | 506 |  | PEAKS DB |
| G.SGAASGAGAGAGSGAGAGSGAGAG.S | 24 | 1632.709 | 24 | -0.2 | 817.3615 | 2 | 30.2 | 5168 | 5191 |  | PEAKS DB |
| Y.GAGVGAGYGAGAGV(-.98).G | 24 | 1061.525 | 14 | -0.1 | 531.7699 | 2 | 40.6 | 3169 | 3182 | Amidation | PEAKS PTM |
| S.SESDFGTGSGAGAGSGA(-.98).G | 23.82 | 1412.58 | 17 | -0.5 | 707.2971 | 2 | 36 | 1250 | 1266 | Amidation | PEAKS PTM |
| G.A(+58.01)GAGAGAGTGSSGFGPY.V | 23.77 | 1441.611 | 17 | -0.3 | 721.8126 | 2 | 56.9 | 665 | 681 | Carboxymethyl (KW, X@N-term) | PEAKS PTM |
| G.SGAGAGSGAGAGSGAGAGSGAGSG.S | 23.69 | 1634.688 | 24 | 0.1 | 818.3514 | 2 | 29.1 | 1737 | 1760 |  | PEAKS DB |
| G.SGAGAGSGAGAGSGAGAGSGTGA(+43.04).G | 23.69 | 1634.724 | 23 | 6.9 | 818.3751 | 2 | 29 | 453 | 475 | Carboxyl modification with ethanolamine | PEAKS PTM |
| G.AGVGAGYGAGAGY(-.98).G | 23.68 | 1068.499 | 13 | -0.3 | 535.2565 | 2 | 41.1 | 4934 | 4946 | Amidation | PEAKS PTM |
| G.SGAGAGSGAGAGSGAGAGYGAG.A | 23.64 | 1566.666 | 22 | -0.8 | 784.3395 | 2 | 34.3 | 519 | 540 |  | PEAKS DB |
| G.SGAGVGYGAGVGAGY.G | 23.53 | 1241.568 | 15 | 0 | 621.7911 | 2 | 48.2 | 625 | 639 |  | PEAKS DB |
| Y.GAGYGVGAGAGY(-.98).G | 23.5 | 997.4617 | 12 | -0.2 | 499.738 | 2 | 41.4 | 394 | 405 | Amidation | PEAKS PTM |
| G.AGAGSGAGYGAGVGAG.Y | 23.49 | 1178.532 | 16 | -0.3 | 590.2729 | 2 | 37.3 | 1278 | 1293 |  | PEAKS DB |
| G.SGAGAGAGSGAGAGSGAGAG.S | 23.48 | 1346.581 | 20 | -1 | 674.2971 | 2 | 28.2 | 1071 | 1090 |  | PEAKS DB |
| A.GAGAGAGYGAGAG.A | 23.45 | 935.4097 | 13 | -0.3 | 468.712 | 2 | 31.2 | 194 | 206 |  | PEAKS DB |
| S.GAASGAGAGAGAGAGTG.S | 23.36 | 1159.522 | 17 | 0.2 | 580.7682 | 2 | 29 | 658 | 674 |  | PEAKS DB |
| G.AGAGSGAGAGSGAGAGSGAGAG.S | 23.33 | 1474.64 | 22 | -0.3 | 738.3268 | 2 | 29.2 | 425 | 446 |  | PEAKS DB |
| D.GTVAQSYVAA.D | 23.28 | 965.4818 | 10 | -0.3 | 483.748 | 2 | 41.7 | 109 | 118 |  | PEAKS DB |
| Y.G(+163.05)AGVGAGYGVGY.G | 23.22 | 1189.523 | 12 | 14.4 | 595.7772 | 2 | 51.2 | 1780 | 1791 | Phenethyl isothiocyanate | PEAKS PTM |
| G.SGAGAGSGAGAGSGAGAGS.G | 23.2 | 1305.554 | 19 | 0.4 | 653.7848 | 2 | 27.9 | 423 | 441 |  | PEAKS DB |
| G.AGAGSGAGAGYGAGVGAG.Y | 23.06 | 1306.59 | 18 | 0.4 | 654.3026 | 2 | 38.5 | 1105 | 1122 |  | PEAKS DB |
| A.GSGAGAGAGSGAGAGY.G | 23.06 | 1166.495 | 16 | 0.3 | 584.255 | 2 | 32.7 | 3893 | 3908 |  | PEAKS DB |
| G.AGAGAGYGAGAGSGAG.S | 23.04 | 1150.5 | 16 | -0.6 | 576.2571 | 2 | 32.1 | 1300 | 1315 |  | PEAKS DB |
| Y.GAGVGAGYGAGAGY(-.98).G | 23 | 1125.52 | 14 | 0 | 563.7674 | 2 | 40.9 | 4933 | 4946 | Amidation | PEAKS PTM |
| G.SGAASGAGAGSGAGAGSGAGAGS(-.98).G | 22.98 | 1590.698 | 23 | -0.2 | 796.3562 | 2 | 28.8 | 497 | 519 | Amidation | PEAKS PTM |
| G.SGAASGAGAGAGAGAGTG.S | 22.97 | 1246.554 | 18 | -0.3 | 624.2839 | 2 | 29.2 | 657 | 674 |  | PEAKS DB |
| Y.GAGAGAGYGAGAGS(-.98).G | 22.97 | 1021.458 | 14 | 0 | 511.7361 | 2 | 29.9 | 484 | 497 | Amidation | PEAKS PTM |
| Y.VAADAGAYSQSGP(-.98).Y | 22.94 | 1191.552 | 13 | -2.6 | 596.7817 | 2 | 36.2 | 116 | 128 | Amidation | PEAKS PTM |
| Y.GAGAGAGYGAGAGSGAG(-.98).S | 22.91 | 1206.538 | 17 | 0 | 604.2761 | 2 | 31.4 | 1299 | 1315 | Amidation | PEAKS PTM |
| F.R(+54.01)ALPC(+57.02)VNC(+57.02) | 22.82 | 1042.469 | 8 | -1.3 | 522.241 | 2 | 39.2 | 5256 | 5263 | Methylglyoxal-derived hydroimidazolone; Carbamidomethylation | PEAKS PTM |
| G.AGAGSGAGAGYGAGVGAG(-.98).Y | 22.77 | 1305.606 | 18 | -0.3 | 653.8101 | 2 | 36.7 | 1105 | 1122 | Amidation | PEAKS PTM |
| G.SGAGAGSGAGAGSGAGAGSGAGAGSGAG.A | 22.72 | 1890.805 | 28 | -15 | 946.3959 | 2 | 30.4 | 423 | 450 |  | PEAKS DB |
| W.SSESDFGTGSGAGAGSGA(-.98).G | 22.72 | 1499.612 | 18 | 0.1 | 750.8135 | 2 | 36.3 | 1249 | 1266 | Amidation | PEAKS PTM |
| G.SGAGAGSGAGAGSGAGVG.Y | 22.72 | 1246.554 | 18 | 0 | 624.2841 | 2 | 30.7 | 613 | 630 |  | PEAKS DB |
| G.SGAASGAGAGSGAGAGSGAGAGSGA(-.98).G | 22.7 | 1718.757 | 25 | -0.4 | 860.3853 | 2 | 29.5 | 497 | 521 | Amidation | PEAKS PTM |
| G.AGSGAGAGSGAGAGAGSGAGAGY.G | 22.7 | 1637.703 | 23 | -2.8 | 819.8564 | 2 | 35.4 | 3886 | 3908 |  | PEAKS DB |
| G.AGSGAGAGAGSGAGAGSGAGAGSGAGA(-.98).G | 22.7 | 1816.805 | 27 | -1.1 | 909.4087 | 2 | 30 | 1069 | 1095 | Amidation | PEAKS PTM |
| G.SGAGAGAGSGAGAGYG.A | 22.69 | 1166.495 | 16 | -0.3 | 584.2546 | 2 | 31.3 | 3894 | 3909 |  | PEAKS DB |
| G.SGAGAGYGAGAGVGY(-.98).G | 22.65 | 1212.552 | 15 | 0.2 | 607.2835 | 2 | 41.9 | 4846 | 4860 | Amidation | PEAKS PTM |
| Y.GAGAGSGAASGAGAGAG.A | 22.64 | 1145.506 | 17 | -0.4 | 573.7601 | 2 | 28.5 | 652 | 668 |  | PEAKS DB |
| G.AGSGAGAGSGAGAGYGAGVGAGY.G | 22.62 | 1741.766 | 23 | -0.9 | 871.8892 | 2 | 46.3 | 1101 | 1123 |  | PEAKS DB |
| N.SGYSTHQGYTS.D | 22.62 | 1186.489 | 11 | 0.4 | 594.252 | 2 | 29.9 | 133 | 143 |  | SPIDER |
| G.SGAGAGSGAGVGYGAGV(-.98).G | 22.62 | 1292.611 | 17 | 0 | 647.3127 | 2 | 40.5 | 619 | 635 | Amidation | PEAKS PTM |
| A.GAGAGAGSGAGAGY.G | 22.61 | 1022.442 | 14 | -0.6 | 512.2278 | 2 | 32.2 | 2334 | 2347 |  | PEAKS DB |
| G.SGAGAGSGAGAGYGAG.A | 22.6 | 1166.495 | 16 | -0.5 | 584.2545 | 2 | 31.8 | 525 | 540 |  | PEAKS DB |
| G.AGAGSGAGAGYGAGVGAGY(-.98).G | 22.58 | 1468.669 | 19 | 0.2 | 735.3422 | 2 | 43.4 | 1105 | 1123 | Amidation | PEAKS PTM |
| G.SGAGAGAGSGAGAGY.G | 22.54 | 1109.474 | 15 | -0.6 | 555.7438 | 2 | 32.5 | 3894 | 3908 |  | PEAKS DB |
| G.SGAGAGSGAGAGYGAGAG.A | 22.51 | 1294.554 | 18 | 0.3 | 648.2843 | 2 | 33.1 | 525 | 542 |  | PEAKS DB |
| G.SGAGAGSGAGAGYGAGAGAGY(-.98).G | 22.49 | 1584.692 | 21 | -1.4 | 793.352 | 2 | 39.7 | 525 | 545 | Amidation | PEAKS PTM |
| S.SESDFGTGSGAGAGSGAGAGS(-.98).G | 22.49 | 1684.692 | 21 | 1.4 | 843.3546 | 2 | 36.8 | 1250 | 1270 | Amidation | PEAKS PTM |
| G.YGAGAGAGAGAGY(-.98).G | 22.49 | 1040.468 | 13 | 0.2 | 521.2411 | 2 | 37 | 201 | 213 | Amidation | PEAKS PTM |
| G.A(+27.99)ASGAGAGAGAGAGTGSSGFGPY.V | 22.47 | 1825.787 | 23 | 0.9 | 913.9014 | 2 | 56.8 | 659 | 681 | Formylation | PEAKS PTM |
| G.AGAGSGAGAGSGAGAGSGAGAGSGAG(-.98).A | 22.45 | 1745.768 | 26 | -0.2 | 873.8909 | 2 | 29.4 | 425 | 450 | Amidation | PEAKS PTM |
| G.AGAGSGAGAGYGAGAGAGY.G | 22.43 | 1441.622 | 19 | -0.4 | 721.8181 | 2 | 41.9 | 527 | 545 |  | PEAKS DB |
| G.SGAASGAGAGSGAGAGSGA.G | 22.43 | 1319.57 | 19 | -0.3 | 660.7921 | 2 | 28.9 | 497 | 515 |  | PEAKS DB |
| G.AGAGSGAGAGSGAGAGSGAGSGA.G | 22.41 | 1561.672 | 23 | -2.4 | 781.8412 | 2 | 29.3 | 2551 | 2573 |  | PEAKS DB |
| G.AGAGAGYGAGAGSGAGSGAGA(-.98).G | 22.41 | 1492.665 | 21 | 0.7 | 747.3405 | 2 | 33 | 1300 | 1320 | Amidation | PEAKS PTM |
| G.AGAGVGYGAGAGAGY.G | 22.39 | 1197.541 | 15 | -0.5 | 599.7776 | 2 | 45.2 | 3178 | 3192 |  | PEAKS DB |
| Y.GAGYGAGAGSGAAS(-.98).G | 22.37 | 1051.468 | 14 | 0 | 526.7414 | 2 | 29.9 | 406 | 419 | Amidation | PEAKS PTM |
| G.SGAGAGSGAGAGYGAGY(-.98).G | 22.22 | 1328.575 | 17 | 0 | 665.2945 | 2 | 37.5 | 1358 | 1374 | Amidation | PEAKS PTM |
| G.SGAGAGSGAGAGAGSGAGAGY.G | 22.21 | 1509.644 | 21 | -0.9 | 755.8287 | 2 | 35 | 3888 | 3908 |  | PEAKS DB |
| G.AASGAGAGSGAGAGSGAGAGS(-.98).G | 22.2 | 1446.645 | 21 | 0 | 724.3296 | 2 | 28.1 | 499 | 519 | Amidation | PEAKS PTM |
| G.SGAGAGAGSGAGAGSGAGAGSGA(-.98).G | 22.1 | 1560.688 | 23 | 0 | 781.3511 | 2 | 28.6 | 1071 | 1093 | Amidation | PEAKS PTM |
| G.SGAGAGSGAGAGYGAGVG(-.98).V | 22.09 | 1321.601 | 18 | -0.1 | 661.8077 | 2 | 36.3 | 729 | 746 | Amidation | PEAKS PTM |
| G.AGSGAGAGSGAGSGAGAGSGAGAGS(-.98).G | 22.08 | 1704.741 | 25 | -0.2 | 853.3777 | 2 | 28.7 | 2559 | 2583 | Amidation | PEAKS PTM |
| F.RALPC(+57.02)VN(-.98).C | 22.06 | 827.4436 | 7 | 0 | 414.7291 | 2 | 31.6 | 5256 | 5262 | Carbamidomethylation; Amidation | PEAKS PTM |
| G.AGAGAGYGAGAGSGAGS.G | 22.05 | 1237.532 | 17 | 0.1 | 619.7734 | 2 | 32.2 | 1300 | 1316 |  | PEAKS DB |
| G.SGAGAGSGAGAGSGAGAGYG(-.98).A | 22.03 | 1437.623 | 20 | -0.4 | 719.8185 | 2 | 31.8 | 519 | 538 | Amidation | PEAKS PTM |
| G.SGAGAGSGAGAGSGAGAGSGAGAGGS(-.98).V | 22.03 | 1761.763 | 26 | -0.5 | 881.8881 | 2 | 29.1 | 5180 | 5205 | Amidation | PEAKS PTM |
| G.AGVGYGAGVGAGY(-.98).G | 22 | 1096.53 | 13 | 0 | 549.2723 | 2 | 45.8 | 627 | 639 | Amidation | PEAKS PTM |
| G.AGAGAGAGAGTGSSGF(-.98).G | 22 | 1193.543 | 16 | -0.4 | 597.7783 | 2 | 35.5 | 663 | 678 | Amidation | PEAKS PTM |
| G.SGAGAGYGAGAGSGAASGA(-.98).G | 21.99 | 1394.617 | 19 | 0.2 | 698.3161 | 2 | 32.8 | 2583 | 2601 | Amidation | PEAKS PTM |
| R.ALPC(+57.02)VNC(+57.02)(+21.98) | 21.97 | 854.3391 | 7 | 0.1 | 428.1769 | 2 | 42.1 | 5257 | 5263 | Carbamidomethylation; Sodium adduct | PEAKS PTM |
| S.GAGAGSGAGAGAGSGAGAG.S | 21.92 | 1259.549 | 19 | -0.1 | 630.7817 | 2 | 28.6 | 1066 | 1084 |  | PEAKS DB |
| Y.G(+163.05)AGVGAGYGAGAGY.G | 21.9 | 1289.55 | 14 | 13.6 | 645.791 | 2 | 49.2 | 4933 | 4946 | Phenethyl isothiocyanate | PEAKS PTM |
| G.AGSGAGAGSGAGAGSGAGAG(+21.98).S | 21.89 | 1368.563 | 20 | -0.8 | 685.2882 | 2 | 28.3 | 421 | 440 | Sodium adduct | PEAKS PTM |
| A.GYGAGAGSGAGSGAGAG.S | 21.88 | 1223.517 | 17 | -0.3 | 612.7654 | 2 | 31.4 | 1305 | 1321 |  | PEAKS DB |
| G.SGAGAGYGAGAGSGAAS.G | 21.88 | 1267.543 | 17 | -0.9 | 634.7781 | 2 | 32.3 | 2583 | 2599 |  | PEAKS DB |
| R.ALPC(+57.02)VN(+15.99)C(+57.02) | 21.86 | 848.3521 | 7 | 0.6 | 425.1836 | 2 | 41.4 | 5257 | 5263 | Carbamidomethylation; Oxidation or Hydroxylation | PEAKS PTM |
| G.AGYGAGV(sub A)GAGYGAGAGAGY.G | 21.81 | 1545.685 | 19 | 0.2 | 773.8499 | 2 | 49.3 | 535 | 553 |  | SPIDER |
| Y.VAN(+.98)GGYSGYEYA.W | 21.79 | 1250.509 | 12 | -0.3 | 626.2617 | 2 | 47.3 | 1639 | 1650 | Deamidation (NQ) | SPIDER |
| S.GAGAGAGAGAGTGSSGF(-.98).G | 21.78 | 1250.564 | 17 | -0.2 | 626.2891 | 2 | 36.1 | 662 | 678 | Amidation | PEAKS PTM |
| A.A(+57.02)SGAGAGSGAGAGSGAGAGSG.A | 21.75 | 1490.635 | 21 | -0.4 | 746.3242 | 2 | 28.6 | 500 | 520 | Carbamidomethylation (DHKE, X@N-term) | PEAKS PTM |
| G.AGAGAGAGTGSSGFGP(-.98).Y | 21.74 | 1219.558 | 16 | 0.2 | 610.7864 | 2 | 38 | 665 | 680 | Amidation | PEAKS PTM |
| G.SGAGAGSGAGAGYGAGVG.V | 21.73 | 1322.585 | 18 | -0.1 | 662.2997 | 2 | 38 | 729 | 746 |  | PEAKS DB |
| G.SGAGAGYGAGVGA.G | 21.73 | 993.4515 | 13 | 0.2 | 497.7331 | 2 | 37.6 | 1109 | 1121 |  | PEAKS DB |
| G.SGAGAGYGAGAGAGY(-.98).G | 21.72 | 1184.521 | 15 | 0 | 593.2678 | 2 | 36.8 | 477 | 491 | Amidation | PEAKS PTM |
| A.GSGAGAGSGAGAGSGAGAGGS.V | 21.72 | 1419.597 | 21 | 0.4 | 710.8063 | 2 | 28.2 | 5185 | 5205 |  | PEAKS DB |
| S.GAGAGSGAGAGYGAG(-.98).A | 21.7 | 1078.479 | 15 | 0.1 | 540.2469 | 2 | 30.3 | 526 | 540 | Amidation | PEAKS PTM |
| G.SGAGAGAGSGAGAGSGAGAGSG(-.98).A | 21.65 | 1489.651 | 22 | -0.3 | 745.8323 | 2 | 27.9 | 1071 | 1092 | Amidation | PEAKS PTM |
| W.SSESDFGTGSGA(-.98).G | 21.6 | 1099.442 | 12 | -0.1 | 550.7281 | 2 | 33.5 | 1249 | 1260 | Amidation | PEAKS PTM |
| A.G(+163.05)AGAGAGYGAGAGAGY.G | 21.59 | 1389.577 | 16 | 12.1 | 695.8043 | 2 | 46.2 | 378 | 393 | Phenethyl isothiocyanate | PEAKS PTM |
| G.AGSGAGAGSGAGAGYGAGV(-.98).G | 21.56 | 1392.638 | 19 | 0.1 | 697.3264 | 2 | 36.8 | 727 | 745 | Amidation | PEAKS PTM |
| S.AAVGAGAGAGAAAGS(-.98).G | 21.53 | 1056.531 | 15 | -0.1 | 529.2728 | 2 | 30.9 | 149 | 163 | Amidation | PEAKS PTM |
| Y.GAGAGSGAGSGAGAGSGAGAGSGAGAG(+21.98).S | 21.53 | 1825.755 | 27 | 0 | 913.8848 | 2 | 30.4 | 1307 | 1333 | Sodium adduct | PEAKS PTM |
| A.SGAGAGAGAGSGAGAGSGAGAGSGA(-.98).G | 21.51 | 1688.746 | 25 | 0.1 | 845.3805 | 2 | 29.2 | 5034 | 5058 | Amidation | PEAKS PTM |
| G.SSGFGPYVAD(sub H)GGYSGYEY.A | 21.42 | 1874.763 | 18 | -0.7 | 938.3883 | 2 | 58.3 | 1229 | 1246 |  | SPIDER |
| G.AGAGYGAGAGSGAGSGAGAG.S | 21.32 | 1422.612 | 20 | -0.4 | 712.3131 | 2 | 33.1 | 1302 | 1321 |  | PEAKS DB |
| Y.GAGAGSGAGSGAGAGSGAGAGSGAG(-.98).A | 21.27 | 1674.731 | 25 | -0.5 | 838.3722 | 2 | 29.1 | 1307 | 1331 | Amidation | PEAKS PTM |
| G.AGAGAGAGTGSSGFGPY(+21.98).V | 21.25 | 1405.587 | 17 | 0.1 | 703.801 | 2 | 48 | 665 | 681 | Sodium adduct | PEAKS PTM |
| Y.VAD(sub H)GGYSGYEY.A | 21.21 | 1179.472 | 11 | -0.1 | 590.7432 | 2 | 47.2 | 1236 | 1246 |  | SPIDER |
| G.AGVGYGAGAGAGY(-.98).G | 21.21 | 1068.499 | 13 | 0.2 | 535.2568 | 2 | 42.1 | 3180 | 3192 | Amidation | PEAKS PTM |
| G.SGAGAGSGAGVGSGAGAGSGA(-.98).G | 21.17 | 1460.66 | 21 | 0 | 731.3375 | 2 | 30.6 | 825 | 845 | Amidation | PEAKS PTM |
| Y.GAGAGSGAASGAGAGSGAGAG.S | 21.07 | 1417.618 | 21 | -0.7 | 709.8159 | 2 | 29.3 | 492 | 512 |  | PEAKS DB |
| G.SGAGAGSGAGAGYGAGVGA.G | 20.93 | 1393.622 | 19 | -0.5 | 697.8181 | 2 | 39.9 | 1103 | 1121 |  | PEAKS DB |
| G.AGAGSGAGAGAGSGAGAG.S | 20.92 | 1202.528 | 18 | -0.4 | 602.2708 | 2 | 28.1 | 1067 | 1084 |  | PEAKS DB |
| A.GVGAGYGAGY(-.98).G | 20.87 | 869.4031 | 10 | 0.3 | 435.709 | 2 | 39 | 634 | 643 | Amidation | PEAKS PTM |
| G.SGAGSGAGAGYGAGAGAGY.G | 20.86 | 1457.617 | 19 | -1.4 | 729.8148 | 2 | 40.9 | 4998 | 5016 |  | PEAKS DB |
| V.GYGAGVGAGY(-.98).G | 20.84 | 869.4031 | 10 | 0 | 435.7088 | 2 | 40.1 | 630 | 639 | Amidation | PEAKS PTM |
| G.AGSGAGAGSGAGAGSGAGAGSGAGVGY(+44.99).G | 20.84 | 1982.831 | 27 | 3.9 | 992.4268 | 2 | 30.1 | 605 | 631 | Oxidation to nitro | PEAKS PTM |
| G.VGAGAGYGAGY(-.98).G | 20.81 | 940.4402 | 11 | 0 | 471.2274 | 2 | 40.2 | 399 | 409 | Amidation | PEAKS PTM |
| G.AGSGAGAGSGAGSGAGAGSGAGAGSGA.G | 20.8 | 1833.784 | 27 | -0.1 | 917.899 | 2 | 30.5 | 2559 | 2585 |  | PEAKS DB |
| G.SSGFGPYVAN(+.98)GG.Y | 20.8 | 1112.477 | 12 | 0 | 557.246 | 2 | 49.9 | 675 | 686 | Deamidation (NQ) | SPIDER |

Table S5. List of unique peptides detected from *A. pernyi* digested with trypsin, FA-trypsin, chymotrypsin, and chymotrypsin-trypsin at False Discovery Rate ≤1%.

| **TRYPSIN** |  |  |  |  |  |  |  |  |  |  |  |
| --- | --- | --- | --- | --- | --- | --- | --- | --- | --- | --- | --- |
| **Peptide** | **-10lgP** | **Mass** | **Length** | **ppm** | **m/z** | **z** | **RT** | **Start** | **End** | **PTM** | **Found By** |
| R.AAGSAAAAAAAAAAAASGAGR.S | 59.31 | 1584.7968 | 21 | 0.7 | 793.4062 | 2 | 35.2 | 2211 | 2231 |  | PEAKS DB |
| R.QASHGAGGAAGAAAGAAAGSSAE(sub R)R.G | 58.66 | 1952.9048 | 24 | -0.6 | 651.9752 | 3 | 20.8 | 112 | 135 |  | SPIDER |
| A.AAGSGAGGIGGGFGR.G | 55.86 | 1190.5792 | 15 | -0.3 | 596.2967 | 2 | 26.4 | 2420 | 2434 |  | PEAKS DB |
| A.AAAAGSGAGGIGGGFGR.G | 50.27 | 1332.6534 | 17 | 0.2 | 667.3341 | 2 | 27.3 | 2418 | 2434 |  | PEAKS DB |
| Y.GSGSAAAAAGSGAGGSGGG.Y | 49.71 | 1305.5544 | 19 | 0.3 | 653.7847 | 2 | 19.1 | 2498 | 2516 |  | PEAKS DB |
| A.AAAGSGAGGIGGGFGR.G | 45.87 | 1261.6163 | 16 | -0.2 | 631.8153 | 2 | 26.9 | 2419 | 2434 |  | PEAKS DB |
| R.AAGSAAAAAAAAAAAAASGAGGAGGG.Y | 43.67 | 1798.8557 | 26 | 0.2 | 600.626 | 3 | 43.5 | 1590 | 1615 |  | PEAKS DB |
| R.AAGSAAAAAAAAAAAAASGAGGAGGG(+15.99).Y | 41.56 | 1814.8506 | 26 | 0.9 | 908.4333 | 2 | 42.7 | 1590 | 1615 | Oxidation or Hydroxylation | PEAKS DB |
| Y.GSGSSSAAAGSGAGGVGGG.Y | 41.03 | 1349.5807 | 19 | -0.3 | 675.7974 | 2 | 20.6 | 150 | 168 |  | PEAKS DB |
| Y.GS(+79.97)Y(+15.99)SAAAAAAAAAAAAGSGAGGR.G | 36.2 | 1902.822 | 23 | 4.9 | 952.423 | 2 | 49.1 | 1538 | 1560 | Phosphorylation (STY); Oxidation or Hydroxylation | PEAKS PTM |
| A.GSGAGGIGGGFGR.G | 35.89 | 1048.505 | 13 | -0.3 | 525.2596 | 2 | 25.9 | 2422 | 2434 |  | PEAKS DB |
| A.AP(sub G)SGAGGIGGGFGR.G | 35.39 | 1159.5734 | 14 | 0 | 580.7939 | 2 | 28.9 | 2421 | 2434 |  | SPIDER |
| A.G(+43.01)SGAGGIGGGFGR.G | 35.37 | 1091.5107 | 13 | -0.1 | 546.7626 | 2 | 32.9 | 2422 | 2434 | Carbamylation | PEAKS PTM |
| A.AAAAASSGAGGR.G | 32.91 | 945.4628 | 12 | -0.2 | 473.7386 | 2 | 0.68 | 341 | 352 |  | PEAKS DB |
| R.QS(sub A)SHGAGGAAGG(sub A)AAGAAAGSSAR.R | 32.63 | 1825.8414 | 23 | -5.8 | 609.6176 | 3 | 24.5 | 112 | 134 |  | SPIDER |
| G.SP(sub G)AGGIGGGFGR.G | 32.53 | 1031.5148 | 12 | 0.2 | 516.7648 | 2 | 31.1 | 2423 | 2434 |  | SPIDER |
| **FA-TRYPSIN** |  |  |  |  |  |  |  |  |  |  |  |
| **Peptide** | **-10lgP** | **Mass** | **Length** | **ppm** | **m/z** | **z** | **RT** | **Start** | **End** | **PTM** | **Found By** |
| R.AAGSAAAAAAAAAAAASGAGR.S | 52.93 | 1584.7968 | 21 | 1.5 | 793.4069 | 2 | 47.5 | 2211 | 2231 |  | PEAKS DB |
| D.D(+15.99)GY(+15.99)GSDSAAAAAAAAAAAAGSGAGGR.G | 46.96 | 2125.926 | 26 | -12 | 1063.958 | 2 | 54.8 | 1656 | 1681 | Oxidation or Hydroxylation | PEAKS DB |
| Y.GSDSAAAAAAAAAAAAASGAR.G | 46.25 | 1658.7971 | 21 | -1 | 830.405 | 2 | 51.3 | 638 | 658 |  | PEAKS DB |
| R.AAGSAAAAAAAAAAAAASGAGGAGGG.Y | 44.98 | 1798.8557 | 26 | 0.1 | 900.4352 | 2 | 56.1 | 1590 | 1615 |  | PEAKS DB |
| G.S(+27.99)GAGGIGGGFGR.G | 43.93 | 1019.4784 | 12 | -0.4 | 510.7462 | 2 | 48 | 2423 | 2434 | Formylation | PEAKS PTM |
| D.S(+27.99)AAAAAAAAAAAASSGAGGRGD.G | 41.33 | 1729.7979 | 22 | 0.1 | 865.9063 | 2 | 57.4 | 333 | 354 | Formylation | PEAKS PTM |
| R.GDGGY(+44.99)GSGSSAAAAAAAAAASAAR.R | 40.42 | 1982.8677 | 24 | 1.1 | 992.4422 | 2 | 53.8 | 539 | 562 | Oxidation to nitro | PEAKS PTM |
| D.S(+27.99)AAAAAAAAAAAAAAGSGAGGIGGGFGRGD.G | 40.11 | 2330.0999 | 30 | 0.1 | 1166.0574 | 2 | 68 | 2407 | 2436 | Formylation | PEAKS PTM |
| D.SAAAAAAAAAAAAASGAR.G | 38.41 | 1399.7167 | 18 | -0.3 | 700.8654 | 2 | 46.8 | 641 | 658 |  | PEAKS DB |
| R.SAGSAAAAAAAAAAAAASGAGGSGG.S | 37.47 | 1773.8241 | 25 | 1.4 | 887.9205 | 2 | 56.6 | 2464 | 2488 |  | PEAKS DB |
| A.GSGAGGIGGGFGR.G | 37.16 | 1048.505 | 13 | -0.3 | 525.2596 | 2 | 38.6 | 2422 | 2434 |  | PEAKS DB |
| D.D(+15.99)GY(+15.99)GSDSAAAAAAAAAAAAGSGAGGRG.G | 37.14 | 2182.9475 | 27 | -12 | 1092.4685 | 2 | 54.4 | 1656 | 1682 | Oxidation or Hydroxylation | PEAKS DB |
| D.GGY(+44.99)GSDSAAAAAAAAAAAAASGAR.G | 36.84 | 1980.8884 | 24 | -0.3 | 991.4512 | 2 | 56.7 | 635 | 658 | Oxidation to nitro | PEAKS PTM |
| D.S(+27.99)AAAAAAAAAAAAASGAR.G | 34.84 | 1427.7117 | 18 | -0.8 | 714.8625 | 2 | 59.9 | 641 | 658 | Formylation | PEAKS PTM |
| R.SAGSAAAAAAAAAAAAASGAGG.S | 33.16 | 1572.7491 | 22 | 0.1 | 787.3819 | 2 | 57.3 | 2464 | 2485 |  | PEAKS DB |
| D.SAAAAAAAAAAAAAAGSGAGGIGGGFGRGD.G | 32.02 | 2302.105 | 30 | 2.7 | 768.3777 | 3 | 56.2 | 2407 | 2436 |  | PEAKS DB |
| Y.GSGGSSAAAAAAAAAAAAR.R | 31.91 | 1458.7174 | 19 | 0.7 | 730.3665 | 2 | 42.8 | 358 | 376 |  | PEAKS DB |
| W.GDDGYGD(sub S)DSAAAAAAAAAAT(sub A)AGSGAGGR.G | 31.47 | 2323.99 | 28 | 4.5 | 1163.0074 | 2 | 56.1 | 1654 | 1681 |  | SPIDER |
| R.GDGGYGSGGSSAAAAAAAAAAAAR.R | 31.4 | 1907.8721 | 24 | 5.4 | 954.9484 | 2 | 52 | 353 | 376 |  | PEAKS DB |
| D.GGYGS(+75.98)GGSSAAAAAAAAAAAAR.R | 31.4 | 1811.8042 | 22 | -14 | 906.897 | 2 | 59.1 | 355 | 376 | EDT | PEAKS PTM |
| G.S(+27.99)SAAAAAAAAAASAAR.R | 30.98 | 1315.6479 | 16 | 0.4 | 658.8315 | 2 | 53.4 | 547 | 562 | Formylation | PEAKS PTM |
| G.Y(+183.98)GSYSAAAAAAAAAAAAGSGAGGR.G | 30.13 | 2153.9072 | 24 | -5.1 | 1077.9554 | 2 | 63.1 | 1537 | 1560 | 3-Sulfobenzoic succinimidyl ester | PEAKS PTM |
| G.GY(+15.99)GSD(+15.99)SAAAAAAAAAAAAAGSGAGGRGD.G | 30.07 | 2253.9846 | 28 | -13 | 1127.9851 | 2 | 62.2 | 2156 | 2183 | Oxidation or Hydroxylation | PEAKS DB |
| D.GGY(+44.99)GSDSAAAAAAAAAAAASSGAGGR.G | 29.31 | 2110.9263 | 26 | 0.3 | 1056.4707 | 2 | 54.5 | 327 | 352 | Oxidation to nitro | PEAKS PTM |
| W.GD(+15.99)DGYGSDSAAAAAAAAAAAAGSGAG.G | 29.26 | 2068.8569 | 26 | 11.6 | 1035.4478 | 2 | 54.9 | 1654 | 1679 | Oxidation or Hydroxylation | PEAKS DB |
| G.S(+27.99)GSSAAAAAAAAAASAAR.R | 27.9 | 1459.7014 | 18 | 1.2 | 730.8589 | 2 | 52.6 | 545 | 562 | Formylation | PEAKS PTM |
| **CHYMOTRYPSIN** |  |  |  |  |  |  |  |  |  |  |  |
| **Peptide** | **-10lgP** | **Mass** | **Length** | **ppm** | **m/z** | **z** | **RT** | **Start** | **End** | **PTM** | **Found By** |
| Y.GSDSAAAAAAAAAAAAAGAGAGGAGGSY.G | 49.23 | 2092.9409 | 28 | 0.3 | 698.6545 | 3 | 50.8 | 481 | 508 |  | PEAKS DB |
| Y.GSDSAAAAAAAAAAAAAAGSGAGGIGGGF.G | 47.38 | 2176.0144 | 29 | -0.4 | 726.3452 | 3 | 57.4 | 2404 | 2432 |  | PEAKS DB |
| Y.GSDSAAAAAAAAAAAAASGAGGSGGY(+15.99).G | 47.1 | 1996.8722 | 26 | -0.3 | 999.4431 | 2 | 48.7 | 452 | 477 | Oxidation or Hydroxylation | PEAKS DB |
| Y.GSDSAAAAAAAAAAAAAGSGAGGSGGY.G | 44.9 | 2037.8987 | 27 | 0.1 | 680.3069 | 3 | 48.5 | 197 | 223 |  | PEAKS DB |
| Y.GSDSAAAAAAAAAAAAAASGAGGSGGY.G | 44.76 | 2051.9143 | 27 | -0.2 | 684.9786 | 3 | 51.7 | 1160 | 1186 |  | PEAKS DB |
| Y.SSDSAAAAAAAAAAAAAGSGAGGAGGGY.G | 44.17 | 2108.9358 | 28 | -1.2 | 703.985 | 3 | 49 | 1624 | 1651 |  | PEAKS DB |
| H.DSAAGSAAAAAAAAAAAAASGAGGSGGGY.G | 44.12 | 2179.9729 | 29 | -0.5 | 727.6646 | 3 | 54.7 | 567 | 595 |  | PEAKS DB |
| Y.GSDSAAAAAAAAAAAAASGARGSGGY.G | 43.97 | 2079.9568 | 26 | 0 | 694.3262 | 3 | 46 | 638 | 663 |  | PEAKS DB |
| Y.GSDSAAAAAAAAAAAAASGAGGSGGY.G | 42.26 | 1980.8772 | 26 | -0.2 | 661.2996 | 3 | 50.2 | 452 | 477 |  | PEAKS DB |
| Y.GSDSSAAAAAAAAAASGAGGSGGGY.G | 41.64 | 1911.8193 | 25 | 0.4 | 638.2806 | 3 | 42.2 | 2372 | 2396 |  | PEAKS DB |
| R.AAGSAAAAAAAAAAAASGAGRSGGSY.G | 40.6 | 2035.967 | 26 | 0.4 | 679.6632 | 3 | 43 | 2211 | 2236 |  | PEAKS DB |
| D.SAAAAAAAAAAAAASGAGGSGGY.G | 40.32 | 1721.7968 | 23 | -0.6 | 574.9392 | 3 | 46.9 | 455 | 477 |  | PEAKS DB |
| Y.GSDSAAAAAAAAAAAAAAAGSGAGGVGGGY.G | 40.07 | 2249.0308 | 30 | -0.4 | 750.6839 | 3 | 54.6 | 1813 | 1842 |  | PEAKS DB |
| Y.GWGDDGYGSDSAAAAAAAAAAAAGSGAGGRGGGY.G | 40.01 | 2843.2131 | 34 | -0.3 | 948.7447 | 3 | 51.6 | 1652 | 1685 |  | PEAKS DB |
| Y.GAYGSDSSAAAAAAAAAASGAGGSGGGY.G | 39.45 | 2202.9414 | 28 | -1 | 1102.4769 | 2 | 47 | 2369 | 2396 |  | PEAKS DB |
| Y.GSYGSDSAAAAAAAAAAAGSGAGGVGGGY.G | 39.17 | 2271.999 | 29 | -0.3 | 758.3401 | 3 | 48.5 | 2307 | 2335 |  | PEAKS DB |
| H.DRAAGSAAAAAAAAAAAAASGAGGAGGGY.G | 39.11 | 2233.0471 | 29 | -0.9 | 1117.5298 | 2 | 50.8 | 1588 | 1616 |  | PEAKS DB |
| A.AAAAAGSGAGGIGGGF.G | 38.97 | 1190.568 | 16 | 0.2 | 596.2914 | 2 | 39.4 | 2417 | 2432 |  | PEAKS DB |
| D.SAAGSAAAAAAAAAAAAASGAGGSGGGY.G | 38.95 | 2064.946 | 28 | -0.3 | 689.3224 | 3 | 51.7 | 568 | 595 |  | PEAKS DB |
| Y.GGYGSDSAAAAAAAAAAAAAAAGSGAGGVGGGY.G | 38.5 | 2526.137 | 33 | -1 | 843.0521 | 3 | 56.9 | 1810 | 1842 |  | PEAKS DB |
| H.DRAAGSAAAAAAAAAAAAASGAGGSGGY.G | 37.97 | 2192.0205 | 28 | 0.4 | 1097.0179 | 2 | 50.3 | 2056 | 2083 |  | PEAKS DB |
| Y.GSDSAAAAAAAAAAAAASGAR.G | 37.83 | 1658.7971 | 21 | 0.6 | 553.94 | 3 | 43 | 638 | 658 |  | PEAKS DB |
| Y.GSD(+15.99)SAAAAAAAAAAAAAGSGAGGRGDGGY.G | 37.58 | 2295.011 | 29 | -1.1 | 766.0101 | 3 | 42.2 | 2158 | 2186 | Oxidation or Hydroxylation | PEAKS DB |
| D.SAAAAAAAAAAAAAGSSAGGAGGGY.G | 36.81 | 1849.8553 | 25 | 0.4 | 617.626 | 3 | 45.7 | 233 | 257 |  | PEAKS DB |
| Y.GSDSAAAAAAAAAAAAAGSGAGGAGGY.G | 36.57 | 2021.9038 | 27 | -0.3 | 674.975 | 3 | 50.4 | 2090 | 2116 |  | PEAKS DB |
| D.SAAAAAAAAAAAAAGAGAGGAGGSY.G | 36.39 | 1833.8605 | 25 | 1.3 | 612.2949 | 3 | 47.4 | 484 | 508 |  | PEAKS DB |
| Y.GSDSAAAAAAAAAAAAGSGAGGRGGGY.G | 35.95 | 2092.9521 | 27 | -0.7 | 698.6575 | 3 | 41.6 | 1659 | 1685 |  | PEAKS DB |
| H.DRAAGSAAAAAAAAAAAASGAGGSGGSY.G | 35.44 | 2208.0154 | 28 | 1 | 1105.0161 | 2 | 46.8 | 1743 | 1770 |  | PEAKS DB |
| D.SAAAAAAAAAAAAAGSGAGGAGGY.G | 35.18 | 1762.8234 | 24 | 0.2 | 588.6152 | 3 | 46.1 | 2093 | 2116 |  | PEAKS DB |
| D.SAAAAAAAAAAAAAASGAGGSGGY.G | 35.1 | 1792.8339 | 24 | -0.4 | 598.6183 | 3 | 48.7 | 1163 | 1186 |  | PEAKS DB |
| Y.GSDSAAAAAAAAAAAAGSGAGGRRGY.G | 34.8 | 2135.0103 | 26 | -5 | 1068.5071 | 2 | 49.3 | 2343 | 2368 |  | PEAKS DB |
| Y.GSDSAAAAAAAAAAAAAGSGAGGRGDGGY.G | 34.63 | 2279.0161 | 29 | 0.1 | 760.6794 | 3 | 44.6 | 2158 | 2186 |  | PEAKS DB |
| A.AAAAAASGARGSGGY.G | 34.41 | 1236.5847 | 15 | 0.5 | 619.2999 | 2 | 27.4 | 649 | 663 |  | PEAKS DB |
| D.SAAAAAAAAAAAAAAAGSGAGGVGGGY.G | 34.33 | 1989.9503 | 27 | 0.2 | 664.3242 | 3 | 51.8 | 1816 | 1842 |  | PEAKS DB |
| Y.GGYGSDSAAAAAAAAAAAAAGAGAGGAGGSY.G | 32.9 | 2370.0471 | 31 | -1.7 | 791.0217 | 3 | 52.9 | 478 | 508 |  | PEAKS DB |
| Y.GSGSSSAAAGSGAGGVGGGY.G | 32.79 | 1512.644 | 20 | -0.4 | 757.329 | 2 | 0.03 | 150 | 169 |  | PEAKS DB |
| A.AAAAAAASGAGRSGGSY.G | 32.12 | 1394.6538 | 17 | -0.2 | 698.334 | 2 | 28.1 | 2220 | 2236 |  | PEAKS DB |
| Y.SSYGSGSSSAAAGSGAGGVGGGY.G | 32.12 | 1849.7714 | 23 | 0.5 | 925.8934 | 2 | 33 | 147 | 169 |  | PEAKS DB |
| Y.GSDSAAAAAAAAAAAAAGAGAGGAGG(+15.99).S | 31.82 | 1858.8405 | 26 | 0.4 | 930.4279 | 2 | 46.1 | 481 | 506 | Oxidation or Hydroxylation | PEAKS DB |
| A.AAAGSGAGGIGGGF.G | 31.81 | 1048.4938 | 14 | -0.3 | 525.254 | 2 | 37.8 | 2419 | 2432 |  | PEAKS DB |
| Y.GSDSAAAAAAAAAAAAAGSGAGGAGGYGGY.G | 31.79 | 2299.01 | 30 | -0.7 | 767.3434 | 3 | 51.1 | 2090 | 2119 |  | PEAKS DB |
| Y.GSGSSSAAAGSGAGGVGGGY(+21.98).G | 31.78 | 1534.626 | 20 | 0.4 | 768.3206 | 2 | 31.6 | 150 | 169 | Sodium adduct | PEAKS PTM |
| Y.GSGSAAAAAGSGAGGSGGGYGWGDGGY.G | 31.64 | 2160.8733 | 27 | 0.4 | 1081.4443 | 2 | 41.5 | 2498 | 2524 |  | PEAKS DB |
| Y.GSDSAAAAAAAAAAAASSGAGGRGDGGY.G | 31.6 | 2237.9897 | 28 | 0 | 747.0038 | 3 | 43.2 | 330 | 357 |  | PEAKS DB |
| D.SAAAAAAAAAAAAASGARGSGGY.G | 31.56 | 1820.8765 | 23 | -0.3 | 607.9659 | 3 | 43.1 | 641 | 663 |  | PEAKS DB |
| D.SAAAAAAAAAAAAAGSGAGGSGGY.G | 31.48 | 1778.8182 | 24 | -0.2 | 593.9466 | 3 | 45 | 200 | 223 |  | PEAKS DB |
| D.SSAAAAAAAAAASGAGGSGGGY.G | 31.47 | 1652.739 | 22 | -0.6 | 827.3763 | 2 | 39.2 | 2375 | 2396 |  | PEAKS DB |
| Y.GSGSAAAAAGSGAGGSGGGY.G | 31.33 | 1468.6178 | 20 | 0.9 | 735.3168 | 2 | 0.03 | 2498 | 2517 |  | PEAKS DB |
| F.YETHNSY.S | 31.26 | 912.3613 | 7 | 0 | 457.1879 | 2 | 26.8 | 140 | 146 |  | PEAKS DB |
| A.AAAAGSGAGGIGGGF.G | 30.73 | 1119.5309 | 15 | -0.6 | 560.7724 | 2 | 38.3 | 2418 | 2432 |  | PEAKS DB |
| Y.GSDSAAAAAAAAAAAAAGSGAGGSGGY(+21.98).G | 30.38 | 2059.8806 | 27 | -0.6 | 687.6337 | 3 | 48.6 | 197 | 223 | Sodium adduct | PEAKS PTM |
| S.GGYGGYGSDSAAAAAAAAAAAAAGSGAGGVGGGY.G | 30.19 | 2661.1689 | 34 | 0.8 | 888.0643 | 3 | 54.8 | 661 | 694 |  | PEAKS DB |
| Y.GSDSAAAAAAAAAAAAASGAGGSGGY(+21.98).G | 30.03 | 2002.8591 | 26 | -1.2 | 1002.4357 | 2 | 50.3 | 452 | 477 | Sodium adduct | PEAKS PTM |
| Y.GAGSAAAAAAAAAAGAGASRQVGIY.G | 30.01 | 2060.0398 | 25 | 0.1 | 687.6873 | 3 | 45.6 | 2552 | 2576 |  | PEAKS DB |
| Y.GSDS(-18.01)AAAAAAAAAAAAASGAGGSGGY.G | 29.86 | 1962.8667 | 26 | 0.7 | 655.2966 | 3 | 49.7 | 452 | 477 | Dehydration | PEAKS PTM |
| Y.SSDSAAAAAAAAAAAAAGSGAGGAGGGY(+21.98).G | 29.53 | 2130.9177 | 28 | -0.5 | 1066.4656 | 2 | 48.9 | 1624 | 1651 | Sodium adduct | PEAKS PTM |
| Y.GSDSAAAAAAAAAAAAAGSGAGGSGGYGGY.G | 29.53 | 2315.0049 | 30 | 0.1 | 772.6757 | 3 | 50.5 | 197 | 226 |  | PEAKS DB |
| G.SAAAAAAAAAAAAASGAGGAGGGY.G | 29.12 | 1762.8234 | 24 | 0.5 | 588.6154 | 3 | 47.6 | 1593 | 1616 |  | PEAKS DB |
| D.SAAAAAAAAAAAAAAGSGAGGIGGGF.G | 29.1 | 1916.934 | 26 | -0.5 | 959.4738 | 2 | 55 | 2407 | 2432 |  | PEAKS DB |
| D.GYGSDSAAAAAAAAAAAAGSGAGGRGGGY.G | 29.06 | 2313.0369 | 29 | -0.4 | 772.0193 | 3 | 44.9 | 1657 | 1685 |  | PEAKS DB |
| Y.GWDYESY.G | 28.59 | 918.3395 | 7 | 0.2 | 460.1771 | 2 | 42.2 | 2491 | 2497 |  | PEAKS DB |
| Y.GWGDGGYGSDSAAAAAAAAAAAAASGARGSGGY.G | 28.35 | 2772.2124 | 33 | -0.1 | 925.0779 | 3 | 54.5 | 631 | 663 |  | PEAKS DB |
| A.AAGAGASRQVGIY.G | 28.25 | 1219.6309 | 13 | -0.2 | 610.8226 | 2 | 32.1 | 2564 | 2576 |  | PEAKS DB |
| D.SAAAAAAAAAAAAAGSGAGGRGDGGY.G | 28.04 | 2019.9358 | 26 | 0.2 | 674.3193 | 3 | 41.5 | 2161 | 2186 |  | PEAKS DB |
| W.GDGGYGSDSAAAAAAAAAAAASSGAGGRGDGGY.G | 28 | 2687.1443 | 33 | 0.4 | 896.7224 | 3 | 47.4 | 325 | 357 |  | PEAKS DB |
| Y.GGYGSDSAAAAAAAAAAAAAGSGAGGSGGY.G | 27.83 | 2315.0049 | 30 | -0.3 | 772.6754 | 3 | 51.5 | 194 | 223 |  | PEAKS DB |
| Y.GSDSAAAAAAAAAAAAAGAGAGGAGGSY(+21.98).G | 27.75 | 2114.9229 | 28 | 0.3 | 1058.469 | 2 | 50.7 | 481 | 508 | Sodium adduct | PEAKS PTM |
| Y.GWGDGGYSSDSAAAAAAAAAAAAAGSGAGGAGGGY.G | 27.68 | 2801.1912 | 35 | -1.2 | 934.7366 | 3 | 57 | 1617 | 1651 |  | PEAKS DB |
| A.AAAAAAGSGAGGIGGGF.G | 27.32 | 1261.6051 | 17 | 0.4 | 631.8101 | 2 | 39.8 | 2416 | 2432 |  | PEAKS DB |
| Y.GSDSSAAAAAAAAAASGAGGSGGGY(+21.98).G | 27.29 | 1933.8014 | 25 | -0.1 | 967.9078 | 2 | 42 | 2372 | 2396 | Sodium adduct | PEAKS PTM |
| Y.GWGDGGYGSDSAAAAAAAAAAAAASGAGGSGGY.G | 27.19 | 2673.1326 | 33 | 0.6 | 892.052 | 3 | 59.2 | 1218 | 1250 |  | PEAKS DB |
| A.AAAAAAAAASGARGSGGY.G | 27.13 | 1449.696 | 18 | 0.2 | 725.8554 | 2 | 30.4 | 646 | 663 |  | PEAKS DB |
| A.AAAAAAAASGARGSGGY.G | 27.02 | 1378.6589 | 17 | 0 | 690.3367 | 2 | 29.2 | 647 | 663 |  | PEAKS DB |
| R.AGHDSAAGSAAAAAAAAAAAAASGAGGSGGGY.G | 26.59 | 2445.0903 | 32 | 0.1 | 816.0375 | 3 | 50.3 | 564 | 595 |  | PEAKS DB |
| Y.GSDSAAAAAAAAAAAAAGSGAGGAGGY(+21.98).G | 26.35 | 2043.8857 | 27 | 0.6 | 1022.9508 | 2 | 49.3 | 2090 | 2116 | Sodium adduct | PEAKS PTM |
| Y.GSDSAAAAAAAAAAAAAASGAGGAGGY(+15.99).G | 26.3 | 2051.9143 | 27 | -1 | 1026.9634 | 2 | 52.8 | 1473 | 1499 | Oxidation or Hydroxylation | PEAKS DB |
| Y.GSDSAAAAAAAAAAAAAGSGAGGAGGY(+15.99).G | 26.28 | 2037.8987 | 27 | 0.3 | 1019.9569 | 2 | 49 | 2090 | 2116 | Oxidation or Hydroxylation | PEAKS DB |
| Y.GSDSAAAAAAAAAAAAASGAGGSGGYGGY.G | 26.11 | 2257.9834 | 29 | 0.6 | 753.6689 | 3 | 52.3 | 452 | 480 |  | PEAKS DB |
| Y.GSDSAAAAAAAAAAAAGSGAGGRGGG.Y | 26.1 | 1929.8888 | 26 | -0.2 | 644.3034 | 3 | 38.5 | 1659 | 1684 |  | PEAKS DB |
| Y.GSDSAAAAAAAAAAAASSGAGGRGD(-18.01)GGY.G | 25.8 | 2219.979 | 28 | -2.4 | 1110.9941 | 2 | 43.6 | 330 | 357 | Dehydration | PEAKS PTM |
| D.SAAAAAAAAAAAAAGAGAGGAGGSY(+15.99).G | 25.7 | 1849.8553 | 25 | 1 | 925.9359 | 2 | 45.8 | 484 | 508 | Oxidation or Hydroxylation | PEAKS DB |
| W.DYESYGSGSAAAAAGSGAGGSGGGY.G | 25.67 | 2125.8459 | 25 | 0.5 | 1063.9308 | 2 | 38.8 | 2493 | 2517 |  | PEAKS DB |
| W.GDGGYGSDSAAAAAAAAAAAAAAGSGAGGIGGGF.G | 25.61 | 2625.1689 | 34 | -0.1 | 1313.5917 | 2 | 61.9 | 2399 | 2432 |  | PEAKS DB |
| A.AAAAAAAGSGAGGRGGGY.G | 25.59 | 1391.6542 | 18 | -0.2 | 696.8342 | 2 | 28.8 | 1668 | 1685 |  | PEAKS DB |
| A.AAAAAAASGARGSGGY.G | 25.29 | 1307.6218 | 16 | -0.1 | 654.8181 | 2 | 28.3 | 648 | 663 |  | PEAKS DB |
| A.AAAAAASGAGRSGGSY.G | 24.96 | 1323.6167 | 16 | 0.5 | 662.816 | 2 | 27.4 | 2221 | 2236 |  | PEAKS DB |
| Y.GSDSAAAAAAAAAAAAASGAGGSGGY(+15.99)GGY.G | 24.87 | 2273.9785 | 29 | -0.1 | 759.0001 | 3 | 50.6 | 452 | 480 | Oxidation or Hydroxylation | PEAKS DB |
| Y.GWGDSGYGSDSAAAAAAAAAAAAASGAGGSGGY.G | 24.77 | 2703.1433 | 33 | 0.2 | 1352.5792 | 2 | 58.2 | 445 | 477 |  | PEAKS DB |
| Y.GSDS(-18.01)SAAAAAAAAAASGAGGSGGGY.G | 24.72 | 1893.8088 | 25 | 0.2 | 947.9119 | 2 | 41.6 | 2372 | 2396 | Dehydration | PEAKS PTM |
| A.AAAAAAGSGAGGRGGGY.G | 24.65 | 1320.6171 | 17 | 0.4 | 661.316 | 2 | 28.3 | 1669 | 1685 |  | PEAKS DB |
| Y.GSD(+15.99)SSAAAAAAAAAASGAGGSGGGY.G | 24.37 | 1927.8143 | 25 | -1.8 | 964.9127 | 2 | 43.2 | 2372 | 2396 | Oxidation or Hydroxylation | PEAKS DB |
| Y.G(+27.99)SGSAAAAAGSGAGGSGGGY.G | 24.14 | 1496.6127 | 20 | 1.3 | 749.3146 | 2 | 35.4 | 2498 | 2517 | Formylation | PEAKS PTM |
| H.GRSAGSAAAAAAAAAAAAASGAGGSGGSY.G | 24.09 | 2237.042 | 29 | 0.9 | 746.6886 | 3 | 48.4 | 2462 | 2490 |  | PEAKS DB |
| S.AAAAAAAAAAAAASGAGGAGGGY.G | 23.96 | 1675.7913 | 23 | 1.7 | 838.9044 | 2 | 42.6 | 1594 | 1616 |  | PEAKS DB |
| A.AAAAASGAGRSGGSY.G | 23.9 | 1252.5796 | 15 | 0 | 627.2971 | 2 | 26.9 | 2222 | 2236 |  | PEAKS DB |
| Y.GGYGGYGSDSAAAAAAAAAAAAAGSSAGGAGGGY.G | 23.86 | 2663.1482 | 34 | 0 | 888.7233 | 3 | 53.3 | 224 | 257 |  | PEAKS DB |
| Y.GSDSAAAAAAAAAAAAAASGAGGSGGY(+21.98).G | 23.56 | 2073.8962 | 27 | -0.1 | 1037.9553 | 2 | 51.8 | 1160 | 1186 | Sodium adduct | PEAKS PTM |
| Y.GGYGGYGSDSAAAAAAAAAAAAAAAGSGAGGVGGGY.G | 23.4 | 2803.2432 | 36 | -2 | 935.4198 | 3 | 58.3 | 1807 | 1842 |  | PEAKS DB |
| S.GYGSDSAAAAAAAAAAAAASGAGGSGGY.G | 23.3 | 2200.9619 | 28 | -0.5 | 1101.4877 | 2 | 52.7 | 450 | 477 |  | PEAKS DB |
| Y.SSYGSGSSSAAAGSGAGGVGGGYGSD.S | 23.27 | 2108.8518 | 26 | 0 | 1055.4332 | 2 | 33.8 | 147 | 172 |  | PEAKS DB |
| R.AAGSAAAAAAAAAAAAASGAGGSGGY.G | 23.25 | 1920.8925 | 26 | -2.9 | 641.3029 | 3 | 49.9 | 2058 | 2083 |  | PEAKS DB |
| A.AAAAAAAAAGAGAGGAGGSY.G | 23.21 | 1462.6799 | 20 | 0.1 | 732.3473 | 2 | 34.1 | 489 | 508 |  | PEAKS DB |
| Y.GSDSAAAAAAAAAAAAAGSGAGGR.G | 22.99 | 1829.8616 | 24 | 0 | 915.938 | 2 | 41.8 | 2158 | 2181 |  | PEAKS DB |
| Y.GAYGSDS(-18.01)SAAAAAAAAAASGAGGSGGGY.G | 22.95 | 2184.9307 | 28 | -0.5 | 1093.472 | 2 | 44.7 | 2369 | 2396 | Dehydration | PEAKS PTM |
| Y.G(+27.99)SGSSSAAAGSGAGGVGGGY.G | 22.94 | 1540.6389 | 20 | -1 | 771.326 | 2 | 36.5 | 150 | 169 | Formylation | PEAKS PTM |
| Y.SS(-18.01)DSAAAAAAAAAAAAAGSGAGGAGGGY.G | 22.8 | 2090.9253 | 28 | 0.2 | 697.9825 | 3 | 48.4 | 1624 | 1651 | Dehydration | PEAKS PTM |
| Y.GGYGSDSAAAAAAAAAAAAAGSGAGGAGGY.G | 22.46 | 2299.01 | 30 | -0.2 | 1150.5121 | 2 | 52.2 | 2087 | 2116 |  | PEAKS DB |
| Y.GS(-18.01)DSSAAAAAAAAAASGAGGSGGGY.G | 22.24 | 1893.8088 | 25 | 0.8 | 947.9124 | 2 | 41.9 | 2372 | 2396 | Dehydration | PEAKS PTM |
| G.Y(+15.99)GSD(+15.99)SAAAAAAAAAAAAASGARGSGGY.G | 21.69 | 2275.01 | 27 | -2.2 | 1138.5098 | 2 | 52.1 | 637 | 663 | Oxidation or Hydroxylation | PEAKS DB |
| D.SAAAAAAAAAAAASSGAGGRGDGGY.G | 21.66 | 1978.9092 | 25 | -0.3 | 660.6435 | 3 | 40.3 | 333 | 357 |  | PEAKS DB |
| Y.GSGSAAAAAGSGAGGSGGGY(+21.98).G | 21.61 | 1490.5997 | 20 | -0.3 | 746.3069 | 2 | 29.2 | 2498 | 2517 | Sodium adduct | PEAKS PTM |
| A.AAAAAAAAGSSAGGAGGGY.G | 21.44 | 1407.6378 | 19 | -0.7 | 704.8257 | 2 | 31.2 | 239 | 257 |  | PEAKS DB |
| Y.GWGDGGYGSDSAAAAAAAAAAAAASGAR.G | 21.44 | 2351.0525 | 28 | 1.6 | 1176.5354 | 2 | 52.1 | 631 | 658 |  | PEAKS DB |
| D.DGYGSDSAAAAAAAAAAAAGSGAGGRGGGY.G | 21.16 | 2428.064 | 30 | -0.3 | 1215.0389 | 2 | 47.1 | 1656 | 1685 |  | PEAKS DB |
| D.RAAGSAAAAAAAAAAAAASGAGGSGGY(+15.99).G | 21.09 | 2092.9885 | 27 | -0.1 | 1047.5015 | 2 | 44.7 | 2057 | 2083 | Oxidation or Hydroxylation | PEAKS DB |
| W.GDGGYGSDSAAAAAAAAAAAAASGAGGSGGY(+15.99).G | 20.98 | 2446.0269 | 31 | -0.1 | 1224.0206 | 2 | 52.1 | 1220 | 1250 | Oxidation or Hydroxylation | PEAKS DB |
| D.SAAAAAAAAAAAAGSGAGGRGGGY.G | 20.92 | 1833.8717 | 24 | 2.1 | 612.2991 | 3 | 38.7 | 1662 | 1685 |  | PEAKS DB |
| R.GGGFYETHNSY.S | 20.89 | 1230.4941 | 11 | -0.3 | 616.2542 | 2 | 33.6 | 136 | 146 |  | PEAKS DB |
| Y.GGYGSDSAAAAAAAAAAAAAASGAGGAGGYGGY.G | 20.82 | 2590.1318 | 33 | -5.1 | 864.3801 | 3 | 41.3 | 1470 | 1502 |  | PEAKS DB |
| Y.G(+27.99)SDSAAAAAAAAAAAAAGSGAGGSGGY.G | 20.8 | 2065.8936 | 27 | 0.8 | 1033.9548 | 2 | 49.9 | 197 | 223 | Formylation | PEAKS PTM |
| W.GDGGYGSDSAAAAAAAAAAAAAGSGAGGRGDGGY.G | 20.64 | 2728.1709 | 34 | -8.6 | 910.3897 | 3 | 47 | 2153 | 2186 |  | PEAKS DB |
| Y.GSDSAAAAAAAAAAAAAAGSGAGGIGGGFGRGDGGY.G | 20.6 | 2838.2915 | 36 | 1.6 | 947.106 | 3 | 53.8 | 2404 | 2439 |  | PEAKS DB |
| Y.GSDSAAAAAAAAAAAGSGAGGVGGGYGW.G | 20.41 | 2207.9832 | 28 | -2.3 | 737 | 3 | 44.9 | 2310 | 2337 |  | PEAKS DB |
| A.AAAAAAAGSGAGGIGGGF.G | 20.4 | 1332.6422 | 18 | 2.7 | 667.3302 | 2 | 40 | 2415 | 2432 |  | PEAKS DB |
| Y.GSDSAAAAAAAAAAAAAAGSGAGGI.G | 20.39 | 1857.8816 | 25 | -13 | 929.9358 | 2 | 45.2 | 2404 | 2428 |  | PEAKS DB |
| D.RAAGSAAAAAAAAAAAASGAGGSGGSY.G | 20.34 | 2092.9885 | 27 | 0.9 | 698.6708 | 3 | 44.6 | 1744 | 1770 |  | PEAKS DB |
| Y.GSDSAAAAAAAAAAAAAGAGAGGAGG.S | 20.1 | 1842.8456 | 26 | -1.5 | 922.4287 | 2 | 48.2 | 481 | 506 |  | PEAKS DB |
| **CHYMOTRYPSIN-TRYPSIN** |  |  |  |  |  |  |  |  |  |  |  |
| **Peptide** | **-10lgP** | **Mass** | **Length** | **ppm** | **m/z** | **z** | **RT** | **Start** | **End** | **PTM** | **Found By** |
| W.GDGGYGSDSAAAAAAAAAAAAASGAR.G | 46.01 | 2107.9519 | 26 | 6 | 703.6621 | 3 | 67.5 | 633 | 658 |  | PEAKS DB |
| R.RGGGFYETHNSY.S | 45.74 | 1386.5952 | 12 | 2.5 | 463.2068 | 3 | 46.2 | 135 | 146 |  | PEAKS DB |
| Y.GSGSSAAAAAAAAAAAGSR.R | 44.92 | 1474.7124 | 19 | 6 | 738.3679 | 2 | 50.1 | 2525 | 2543 |  | PEAKS DB |
| Y.GSGSSAAAAAAAAAAAGSRR.S | 44.77 | 1630.8135 | 20 | 4.2 | 544.6141 | 3 | 49.4 | 2525 | 2544 |  | PEAKS DB |
| Y.GSDSAAAAAAAAAAAAASGAR.G | 42.17 | 1658.7971 | 21 | 5.2 | 553.9425 | 3 | 63.4 | 638 | 658 |  | PEAKS DB |
| W.GDDGYGSDSAAAAAAAAAAAAGSGAGGR.G | 41.9 | 2265.9846 | 28 | 5.4 | 756.3395 | 3 | 62.1 | 1654 | 1681 |  | PEAKS DB |
| W.GDGGYGSGSSAAAAAAAAAAAGSRR.S | 40.41 | 2079.968 | 25 | 2.7 | 694.3318 | 3 | 55 | 2520 | 2544 |  | PEAKS DB |
| R.AYGAGSAAAAAAAAAAGAGASRQVGIY.G | 40 | 2294.1401 | 27 | 3.9 | 765.7236 | 3 | 70.3 | 2550 | 2576 |  | PEAKS DB |
| Y.GSGGSSAAAAAAAAAAAAR.R | 37.66 | 1458.7174 | 19 | 4.8 | 730.3694 | 2 | 53.4 | 358 | 376 |  | PEAKS DB |
| R.AAGSAAAAAAAAAAAAASGAGGSGGY.G | 33.11 | 1920.8925 | 26 | 6.1 | 641.3087 | 3 | 67.8 | 2058 | 2083 |  | PEAKS DB |
| R.RGGGFYETHN(+.98)SYSSY.G | 31.31 | 1724.7067 | 15 | 4.3 | 575.912 | 3 | 51.3 | 135 | 149 | Deamidation (NQ) | PEAKS DB |
| R.GDGGYGSGGSSAAAAAAAAAAAARR.A | 28.83 | 2063.9731 | 25 | 4.9 | 689.0017 | 3 | 57.1 | 353 | 377 |  | PEAKS DB |

Table S6. List of unique peptides detected from *A. mylitta* digested with trypsin, FA-trypsin, chymotrypsin, and chymotrypsin-trypsin at False Discovery Rate ≤1%.

| **TRYPSIN** |  |  |  |  |  |  |  |  |  |  |  |
| --- | --- | --- | --- | --- | --- | --- | --- | --- | --- | --- | --- |
| **Peptide** | **-10lgP** | **Mass** | **Length** | **ppm** | **m/z** | **z** | **RT** | **Start** | **End** | **PTM** | **Found By** |
| R.Q(-17.03)ASHGAGGAAGAAAGAAASSSVR.G | 79.95 | 1864.878 | 23 | -0.6 | 933.4455 | 2 | 26 | 111 | 133 | Pyro-glu from Q | PEAKS PTM |
| H.GAGGAAGAAAGAAASSSVR.G | 59.32 | 1458.717 | 19 | -1.2 | 730.3651 | 2 | 24.2 | 115 | 133 |  | PEAKS DB |
| R.Q(+.98)ASHGAGGAAGAAAGAAASSSVR.G | 57.86 | 1882.888 | 23 | -1.1 | 628.6359 | 3 | 23 | 111 | 133 | Deamidation (NQ) | PEAKS DB |
| R.Q(+43.01)ASHGAGGAAGAAAGAAASSSVR.G | 56.94 | 1924.91 | 23 | -0.8 | 642.6434 | 3 | 26 | 111 | 133 | Carbamylation | PEAKS PTM |
| R.QASHGAGGAAGAAAGAAASSSVR.G | 56.11 | 1881.904 | 23 | -0.5 | 628.3083 | 3 | 22.8 | 111 | 133 |  | PEAKS DB |
| R.GDGGY(+44.99)GSGSSAAAAAAAAAAASAAR.R | 52.61 | 2053.905 | 25 | 0.5 | 1027.96 | 2 | 43.2 | 476 | 500 | Oxidation to nitro | PEAKS PTM |
| H.D(+45.99)EYVDSHGQLVER.F | 49.66 | 1591.694 | 13 | -9.9 | 796.8462 | 2 | 33.8 | 24 | 36 | Beta-methylthiolation (ND) | PEAKS PTM |
| Q.ASHGAGGAAGAAAGAAASSSVR.G | 47.57 | 1753.846 | 22 | -1.3 | 585.6216 | 3 | 21.8 | 112 | 133 |  | PEAKS DB |
| R.QASHGAGGAAGAAAGAAASS.S | 45.91 | 1539.703 | 20 | -2.5 | 770.8566 | 2 | 20.6 | 111 | 130 |  | PEAKS DB |
| R.QAS(-18.01)HGAGGAAGAAAGAAASSSVR.G | 44.7 | 1863.894 | 23 | 0.1 | 932.9541 | 2 | 26.2 | 111 | 133 | Dehydration | PEAKS PTM |
| Y.Y(sub S)SYGSDSAAAAAAAAASGAGGR.G | 42.31 | 1901.85 | 22 | -2.1 | 951.9305 | 2 | 51.9 | 146 | 167 |  | SPIDER |
| Y.VDS(-18.01)HGQLVER.F | 41.83 | 1120.563 | 10 | -1.1 | 561.2879 | 2 | 23.5 | 27 | 36 | Dehydration | PEAKS PTM |
| G.A(+58.01)GGAAGAAAGAAASSSVR.G | 41.59 | 1459.701 | 18 | -0.1 | 730.8579 | 2 | 30.4 | 116 | 133 | Carboxymethyl (KW, X@N-term) | PEAKS PTM |
| **FA-TRYPSIN** |  |  |  |  |  |  |  |  |  |  |  |
| **Peptide** | **-10lgP** | **Mass** | **Length** | **ppm** | **m/z** | **z** | **RT** | **Start** | **End** | **PTM** | **Found By** |
| Y.GSDSGAAAAAAAAAAAAASGAGGR.G | 49.16 | 1829.862 | 24 | 0.2 | 915.9382 | 2 | 52.6 | 240 | 263 |  | PEAKS DB |
| S.G(+43.01)AAAAAAAAAAAAASGAGGR.G | 37.44 | 1526.755 | 20 | -0.6 | 764.3843 | 2 | 59 | 244 | 263 | Carbamylation | PEAKS PTM |
| D.SGAAAAAAAAAAAAASGAGGR.G | 37.08 | 1570.781 | 21 | 0.2 | 786.398 | 2 | 47 | 243 | 263 |  | PEAKS DB |
| Y.GSGSSAAAAAAAAAAASAAR.R | 37.05 | 1559.765 | 20 | 1.4 | 780.8909 | 2 | 44.9 | 481 | 500 |  | PEAKS DB |
| Y.GSDSGAAAAAAAAAAAAASGAGGRGD.G | 36.92 | 2001.91 | 26 | -1.5 | 1001.961 | 2 | 53.2 | 240 | 265 |  | PEAKS DB |
| Y.GSDSGAAAAAAAAAAAAASGAGGRG.D | 35.85 | 1886.883 | 25 | 1 | 944.4497 | 2 | 49.6 | 240 | 264 |  | PEAKS DB |
| R.Q(-17.03)ASHGAGGAAGAAAGAAASSSVR.G | 35.81 | 1864.878 | 23 | -0.4 | 622.6329 | 3 | 37 | 111 | 133 | Pyro-glu from Q | PEAKS PTM |
| G.SDSGAAAAAAAAAAAAASGAGGR.G | 35.17 | 1772.84 | 23 | 0.8 | 887.428 | 2 | 50.5 | 241 | 263 |  | PEAKS DB |
| D.SGAAAAAAAAAAAAASGAGGRGD.G | 33.37 | 1742.83 | 23 | 1.5 | 872.4233 | 2 | 47.5 | 243 | 265 |  | PEAKS DB |
| Y.VDSHGQLVER.F | 32.47 | 1138.573 | 10 | -1 | 380.5312 | 3 | 32.2 | 27 | 36 |  | PEAKS DB |
| S.GSSAAAAAAAAAAASAAR.R | 32.37 | 1415.712 | 18 | 0.1 | 708.8632 | 2 | 44 | 483 | 500 |  | PEAKS DB |
| D.EY(+15.99)E(sub V)DSHGQLVER.F | 31.85 | 1476.648 | 12 | 0.1 | 739.3314 | 2 | 47.3 | 25 | 36 | Oxidation or Hydroxylation | SPIDER |
| G.SGSSAAAAAAAAAAASAAR.R | 31.51 | 1502.744 | 19 | 1.4 | 752.3802 | 2 | 44.7 | 482 | 500 |  | PEAKS DB |
| S.SYGS(+162.05)DSAAAAAAAAASGAGGR.G | 28.7 | 1900.84 | 21 | 13.2 | 951.4397 | 2 | 58.4 | 147 | 167 | Hexose (NSY) | PEAKS PTM |
| D.SAAAAAAAAASGAGGR.G | 27.37 | 1229.611 | 16 | -0.5 | 615.8126 | 2 | 36.1 | 152 | 167 |  | PEAKS DB |
| S.A(+175.04)AAAAAAAAAASAAR.R | 27.25 | 1359.668 | 15 | 5.3 | 680.845 | 2 | 55.4 | 486 | 500 | Naphthalene-2,3-dicarboxaldehyde | PEAKS PTM |
| Y.GSDPGAAAAAAAAAAAAAS.G | 25.46 | 1441.68 | 19 | 8 | 721.8529 | 2 | 67.7 | 452 | 470 |  | PEAKS DB |
| D.EYVD(+45.99)SHGQLVER.F | 24.46 | 1476.667 | 12 | -14 | 739.3304 | 2 | 46.2 | 25 | 36 | Beta-methylthiolation (ND) | PEAKS PTM |
| **CHYMOTRYPSIN** |  |  |  |  |  |  |  |  |  |  |  |
| **Peptide** | **-10lgP** | **Mass** | **Length** | **ppm** | **m/z** | **z** | **RT** | **Start** | **End** | **PTM** | **Found By** |
| Y.VDSHGQLVERF.T | 44.27 | 1285.642 | 11 | 0.3 | 643.8282 | 2 | 36.2 | 27 | 37 |  | PEAKS DB |
| Y.GSDSAAAAAAAAAAAAAAGSGAGGRGDGGY.G | 43.29 | 2350.053 | 30 | -0.9 | 784.3577 | 3 | 46.5 | 203 | 232 |  | PEAKS DB |
| D.SGAAAAAAAAAAAAASGAGGRGDGGY.G | 42.94 | 2019.936 | 26 | -0.2 | 674.3191 | 3 | 43.7 | 243 | 268 |  | PEAKS DB |
| Y.VD(-18.01)SHGQLVERF.T | 42.53 | 1267.631 | 11 | 0.1 | 634.8228 | 2 | 38.5 | 27 | 37 | Dehydration | PEAKS PTM |
| Y.GSDSGAAAAAAAAAAAAASGAGGRGDGGY.G | 40.95 | 2279.016 | 29 | 0.1 | 760.6794 | 3 | 46.8 | 240 | 268 |  | PEAKS DB |
| Y.VDSHGQ(+.98)LVERF.T | 40.18 | 1286.626 | 11 | 0.4 | 429.8826 | 3 | 37.3 | 27 | 37 | Deamidation (NQ) | PEAKS DB |
| Y.GRGDGGYGSDSAAAAAAAAAAAAGSGAGGRGDGGY.G | 39.56 | 2870.256 | 35 | -0.1 | 957.7593 | 3 | 45.2 | 374 | 408 |  | PEAKS DB |
| A.AAAAAGSGAGGRGDGGYGWGDGGY.G | 38.42 | 2056.862 | 24 | -0.4 | 1029.438 | 2 | 38 | 216 | 239 |  | PEAKS DB |
| H.GAGGAAGAAAGAAASSSVRGGGGF.Y | 38.42 | 1833.872 | 24 | -0.1 | 612.2978 | 3 | 36.6 | 115 | 138 |  | PEAKS DB |
| Y.GSDSGAAAAAAAAAAAAASGAGGRGDG.G | 38.17 | 2058.931 | 27 | -0.3 | 1030.473 | 2 | 43.8 | 240 | 266 |  | PEAKS DB |
| Y.GSDSGAAAAAAAAAAAAASGAGGR.G | 37.71 | 1829.862 | 24 | -0.4 | 610.9609 | 3 | 43.5 | 240 | 263 |  | PEAKS DB |
| Y.D(sub S)SYGSDSAAAAAAAAASGAGGRGH.G | 36.89 | 2047.894 | 24 | -1 | 1024.953 | 2 | 39.4 | 146 | 169 |  | SPIDER |
| A.AAAASASGAGGSGGSY.E | 36.84 | 1240.532 | 16 | 0.6 | 621.2736 | 2 | 29.4 | 323 | 338 |  | PEAKS DB |
| Y.GWGDGGYGSDSGAAAAAAAAAAAAASGAGGRGDGGY.G | 36.46 | 2971.272 | 36 | 0.5 | 991.4316 | 3 | 54.9 | 233 | 268 |  | PEAKS DB |
| Y.GSDSGAAAAAAAAAAAAASGAGGRGD.G | 35.99 | 2001.91 | 26 | 0.2 | 668.3107 | 3 | 43.8 | 240 | 265 |  | PEAKS DB |
| Y.GSDSGAAAAAAAAAAAAASGA.G | 35.54 | 1559.718 | 21 | -1 | 780.8652 | 2 | 48.4 | 240 | 260 |  | PEAKS DB |
| Y.GSDSGAAAAAAAAAAAAASGAGGRGD(-18.01)GGY.G | 34.63 | 2261.006 | 29 | 0 | 754.6758 | 3 | 46.3 | 240 | 268 | Dehydration | PEAKS PTM |
| D.EYVDSHGQLVERF.T | 34.5 | 1577.747 | 13 | -0.7 | 789.8804 | 2 | 38.3 | 25 | 37 |  | PEAKS DB |
| Y.GSDSAAAAAAAAAAAAAAGSGAGGRGD.G | 34.35 | 2072.947 | 27 | -0.4 | 691.9893 | 3 | 44.5 | 203 | 229 |  | PEAKS DB |
| G.SGAGGRGDGGYGW.G | 34.29 | 1195.501 | 13 | 0 | 598.7576 | 2 | 34 | 222 | 234 |  | PEAKS DB |
| D.SAAAAAAAAAAAAAAGSGAGGRGDGGY.G | 34.19 | 2090.973 | 27 | 0.4 | 697.9985 | 3 | 43.8 | 206 | 232 |  | PEAKS DB |
| D.GGYGSDSGAAAAAAAAAAAAASGAGGRGDGGY.G | 34.17 | 2556.122 | 32 | 0.9 | 1279.07 | 2 | 48.5 | 237 | 268 |  | PEAKS DB |
| A.AAAAAASGAGV(sub G)RGDGGY.G | 34.14 | 1420.669 | 17 | -0.4 | 711.3417 | 2 | 30.6 | 252 | 268 |  | SPIDER |
| Y.GRGDGGYGSDSAAAAAAAAAAAAGS(-18.01)GAGGRGDGGY.G | 34.05 | 2852.246 | 35 | 2.2 | 951.7579 | 3 | 43.9 | 374 | 408 | Dehydration | PEAKS PTM |
| W.GDGGYGSDSGAAAAAAAAAAAAASGAGGRGDGGY.G | 33.57 | 2728.171 | 34 | 0 | 910.3976 | 3 | 50.6 | 235 | 268 |  | PEAKS DB |
| Y.GSDSGAAAAAAAAAAAAASGAGGRG.D | 33.55 | 1886.883 | 25 | 1.9 | 944.4505 | 2 | 43.4 | 240 | 264 |  | PEAKS DB |
| R.GGGGFYETHDSY.S | 33.25 | 1288.5 | 12 | -0.1 | 645.257 | 2 | 33.9 | 134 | 145 |  | PEAKS DB |
| D.SHGQLVERF.T | 33.21 | 1071.546 | 9 | -0.4 | 536.7802 | 2 | 35.9 | 29 | 37 |  | PEAKS DB |
| Y.VD(+15.99)SHGQLVERF.T | 33.11 | 1301.636 | 11 | -1.3 | 651.8246 | 2 | 38.3 | 27 | 37 | Oxidation or Hydroxylation | PEAKS DB |
| Y.GSDSGAAAAAAAAAAAAAS.G | 33.08 | 1431.659 | 19 | 0.2 | 716.8369 | 2 | 44.3 | 240 | 258 |  | PEAKS DB |
| Y.GRGDGGYGSDS(-18.01)AAAAAAAAAAAAGSGAGGRGDGGY.G | 32.64 | 2852.246 | 35 | -0.2 | 951.7556 | 3 | 42.8 | 374 | 408 | Dehydration | PEAKS PTM |
| Y.GSGSSAAAAAAAAAAASAA.R | 32.63 | 1403.664 | 19 | -0.2 | 702.8392 | 2 | 42 | 481 | 499 |  | PEAKS DB |
| Y.GSDSAAAAAAAAAAAAAAGSGAGGR.G | 32.45 | 1900.899 | 25 | 0 | 634.6402 | 3 | 44.2 | 203 | 227 |  | PEAKS DB |
| G.GRGDGGYGWGDGGY.G | 32.06 | 1372.543 | 14 | -0.1 | 687.2788 | 2 | 38.8 | 226 | 239 |  | PEAKS DB |
| Y.VDSD(sub H)GQLVERF.T | 31.97 | 1263.61 | 11 | -0.1 | 632.812 | 2 | 40.9 | 27 | 37 |  | SPIDER |
| Y.GRGDGGYGSDSAAAAAAAAAAAAGSGAGGRGD(-18.01)GGY.G | 31.88 | 2852.246 | 35 | 1.8 | 951.7576 | 3 | 44.9 | 374 | 408 | Dehydration | PEAKS PTM |
| Y.SSYGSDSAAAAAAAAASGAGGRGHGGY.G | 31.29 | 2297.006 | 27 | -0.4 | 766.6755 | 3 | 37.6 | 146 | 172 |  | PEAKS DB |
| V.DSHGQLVERF.T | 31.07 | 1186.573 | 10 | -0.2 | 594.2936 | 2 | 36.2 | 28 | 37 |  | PEAKS DB |
| A.SHGAGGAAGAAAGAAASSSVRGGGGFY.E | 30.7 | 2221.026 | 27 | -1.5 | 741.3481 | 3 | 38 | 113 | 139 |  | PEAKS DB |
| A.AASASGAGGSGGSYEW.D | 30.6 | 1413.58 | 16 | -0.4 | 707.7968 | 2 | 38 | 325 | 340 |  | PEAKS DB |
| Y.GRGDGGYGSDSAAAAAAAAAAAAGSGAGGRGD.G | 30.57 | 2593.15 | 32 | 1 | 865.3915 | 3 | 41.7 | 374 | 405 |  | PEAKS DB |
| H.GAGGAAGAAAGAAASSSVRGGGGFY.E | 30.34 | 1996.935 | 25 | -0.2 | 999.4746 | 2 | 38.4 | 115 | 139 |  | PEAKS DB |
| D.E(+27.99)YVDSHGQLVERF.T | 30.3 | 1605.742 | 13 | 0.7 | 803.879 | 2 | 44.6 | 25 | 37 | Formylation | PEAKS PTM |
| A.SSSVRGGGGFY.E | 29.94 | 1072.494 | 11 | 0.2 | 537.2543 | 2 | 31.7 | 129 | 139 |  | PEAKS DB |
| H.DEYVDSHGQLVERF.T | 29.82 | 1692.774 | 14 | -0.4 | 565.2651 | 3 | 39.5 | 24 | 37 |  | PEAKS DB |
| L.AAAAAAASASGAGGSGGSY.E | 29.7 | 1453.643 | 19 | 1.3 | 727.8299 | 2 | 31.9 | 320 | 338 |  | PEAKS DB |
| Y.GSGSSAAAAAAAAAAASAAR.R | 29.56 | 1559.765 | 20 | -0.1 | 520.929 | 3 | 38.5 | 481 | 500 |  | PEAKS DB |
| A.AAGSGAGGRGDGGYGWGDGGY.G | 29.26 | 1843.751 | 21 | -0.3 | 922.8825 | 2 | 38.2 | 219 | 239 |  | PEAKS DB |
| A.AAGSGAGGRGDGGYGW.G | 29.16 | 1394.596 | 16 | -2.5 | 698.3037 | 2 | 34.1 | 219 | 234 |  | PEAKS DB |
| Y.G(+27.99)SDSAAAAAAAAASGAGGRGH.G | 28.97 | 1710.767 | 21 | -0.4 | 856.3904 | 2 | 35.9 | 149 | 169 | Formylation | PEAKS PTM |
| G.SGAGGRGDGGYGWGDGGY.G | 28.89 | 1644.655 | 18 | -0.2 | 823.3347 | 2 | 38.6 | 222 | 239 |  | PEAKS DB |
| Y.GSD(+21.98)SAAAAAAAAASGAGGRGHGGY.G | 28.74 | 1981.86 | 24 | 1.7 | 991.939 | 2 | 40.1 | 149 | 172 | Sodium adduct | PEAKS PTM |
| A.AAAAASASGAGGSGGSY.E | 28.6 | 1311.569 | 17 | 0.8 | 656.7924 | 2 | 29.8 | 322 | 338 |  | PEAKS DB |
| Y.GSDSGAAAAAAAAAAAAASGAGGRGD(-.98).G | 28.39 | 2000.926 | 26 | -1 | 1001.469 | 2 | 44.2 | 240 | 265 | Amidation | PEAKS PTM |
| Y.GSDSAAAAAAAAAAAAAGSGAGGVGGGYGRGDGGY.G | 28.26 | 2769.234 | 35 | 0.3 | 924.0854 | 3 | 48.4 | 346 | 380 |  | PEAKS DB |
| Y.GR(+54.01)GDGGYGSDSAAAAAAAAAAAAGSGAGGRGDGGY.G | 28.2 | 2924.267 | 35 | 0.7 | 975.7635 | 3 | 45.6 | 374 | 408 | Methylglyoxal-derived hydroimidazolone | PEAKS PTM |
| Y.G(+27.99)SDSGAAAAAAAAAAAAASGAGGRGDGGY.G | 27.95 | 2307.011 | 29 | 0.1 | 1154.513 | 2 | 51.8 | 240 | 268 | Formylation | PEAKS PTM |
| S.SSVRGGGGFY.E | 27.91 | 985.4617 | 10 | 0 | 493.7381 | 2 | 31.8 | 130 | 139 |  | PEAKS DB |
| Y.GSDSAAAAAAAAAAAAAAGSGAGGRGD(-18.01)GGY.G | 27.71 | 2332.043 | 30 | -0.2 | 778.3547 | 3 | 46.6 | 203 | 232 | Dehydration | PEAKS PTM |
| Y.GRGDGGYGSDSAAAAAAAAAAAAGSGAGGRGDGGYGW.G | 27.69 | 3113.357 | 37 | -0.2 | 1038.793 | 3 | 50.3 | 374 | 410 |  | PEAKS DB |
| Y.VDSHGH(sub Q)LVERF.T | 27.56 | 1294.642 | 11 | 0.3 | 432.5547 | 3 | 37.5 | 27 | 37 |  | SPIDER |
| Y.G(+71.04)RGDGGYGSDSAAAAAAAAAAAAGSGAGGRGDGGY.G | 27.49 | 2941.294 | 35 | 0.2 | 981.4386 | 3 | 45.9 | 374 | 408 | Propionamide (K, X@N-term) | PEAKS PTM |
| Y.VD(+15.99)SH(+15.99)GQLVERF.T | 27.46 | 1317.631 | 11 | -1.2 | 659.8221 | 2 | 38.1 | 27 | 37 | Oxidation or Hydroxylation; Oxidation (HW) | PEAKS PTM |
| H.GAGGAAGAAAGAAASSSVR.G | 27.45 | 1458.717 | 19 | 0.3 | 730.3662 | 2 | 30.8 | 115 | 133 |  | PEAKS DB |
| Y.GWGDGGYGSDSGAAA.A | 27.43 | 1326.511 | 15 | -0.5 | 664.2626 | 2 | 37.2 | 233 | 247 |  | PEAKS DB |
| Y.GSGSSAAAAAAAAAAAS.A | 27.39 | 1261.59 | 17 | -0.4 | 631.8019 | 2 | 36.9 | 481 | 497 |  | PEAKS DB |
| W.GDGGYGT(sub S)DSGAAAAAAAAAAAAASGAGGRGDGGY.G | 27.38 | 2742.187 | 34 | 3.6 | 915.0728 | 3 | 42.5 | 235 | 268 |  | SPIDER |
| Y.GS(-18.01)DSAAAAAAAAASGAGGRGHGGY.G | 27.08 | 1941.868 | 24 | 0.3 | 648.2967 | 3 | 34.6 | 149 | 172 | Dehydration | PEAKS PTM |
| A.AAAAAGSGAGGRGDGGYGW.G | 26.89 | 1607.708 | 19 | -1 | 804.8603 | 2 | 34.8 | 216 | 234 |  | PEAKS DB |
| Y.GSDSGAAAAAAAAAAAAASGAGGR(+54.01)GDGGY.G | 26.49 | 2333.027 | 29 | 0.3 | 778.6831 | 3 | 47.3 | 240 | 268 | Methylglyoxal-derived hydroimidazolone | PEAKS PTM |
| A.AAAAAASASGAGGSGGSY.E | 26.2 | 1382.606 | 18 | 2.7 | 692.3123 | 2 | 30.3 | 321 | 338 |  | PEAKS DB |
| A.AAAAGSGAGGRGDGGYGW.G | 25.98 | 1536.671 | 18 | 0.3 | 769.3428 | 2 | 34.7 | 217 | 234 |  | PEAKS DB |
| Y.GSD(-18.01)SAAAAAAAAASGAGGRGHGGY.G | 25.95 | 1941.868 | 24 | 1.8 | 648.2977 | 3 | 34.5 | 149 | 172 | Dehydration | PEAKS PTM |
| D.E(-18.01)YVDSHGQLVERF.T | 25.95 | 1559.737 | 13 | 0.2 | 780.8759 | 2 | 44.5 | 25 | 37 | Pyro-glu from E | PEAKS PTM |
| Y.GRGDGGYGSDSAAAAAAAAAAAAGSGAGGRGDGGY(+15.99).G | 25.63 | 2886.251 | 35 | 1.5 | 963.0925 | 3 | 41.6 | 374 | 408 | Oxidation or Hydroxylation | PEAKS DB |
| Y.GSDSAAAAAAAAASGAGGR(+54.01)GHGGY.G | 25.45 | 2013.889 | 24 | 0.5 | 1007.952 | 2 | 35.8 | 149 | 172 | Methylglyoxal-derived hydroimidazolone | PEAKS PTM |
| Q.ASHGAGGAAGAAAGAAASSSVRGGGGFY.E | 25.41 | 2292.063 | 28 | 0.1 | 765.0284 | 3 | 37.7 | 112 | 139 |  | PEAKS DB |
| S.SVRGGGGFY.E | 25.41 | 898.4297 | 9 | -0.9 | 450.2217 | 2 | 32 | 131 | 139 |  | PEAKS DB |
| Y.GRGDGGYGSDSAAAAAAAAAAAAG.S | 25.4 | 1935.867 | 24 | 0.6 | 968.9413 | 2 | 42.7 | 269 | 292 |  | PEAKS DB |
| Y.GSDSAAAAAAAAASGAGGRGHGGYGSD.S | 25.32 | 2218.959 | 27 | -1.3 | 740.6592 | 3 | 34.2 | 149 | 175 |  | PEAKS DB |
| Y.GSDSAAAAAAAAAAAAGSGAGGRGDGGYGW.G | 25.15 | 2451.08 | 30 | -0.4 | 1226.547 | 2 | 48.6 | 381 | 410 |  | PEAKS DB |
| Y.GWGD(+21.98)GGYGSDSGAAAAAAAAAAAAASGAGGRGDGGY.G | 25.1 | 2993.254 | 36 | -0.7 | 998.7578 | 3 | 54.9 | 233 | 268 | Sodium adduct | PEAKS PTM |
| Y.VDS(-18.01)HGQLVERF.T | 24.88 | 1267.631 | 11 | -0.1 | 423.5509 | 3 | 37.5 | 27 | 37 | Dehydration | PEAKS PTM |
| A.SASGAGGSGGSYEW.D | 24.78 | 1271.505 | 14 | -0.3 | 636.7598 | 2 | 37.5 | 327 | 340 |  | PEAKS DB |
| Y.GWGDGGY(+15.99)GSDSGAAAAAAAAAAAAASGAGGRGDGGY.G | 24.67 | 2987.267 | 36 | 1 | 996.7639 | 3 | 51.9 | 233 | 268 | Oxidation or Hydroxylation | PEAKS DB |
| Y.GRGDGGYGSDSAAAAAAAAAAA.A | 24.65 | 1807.809 | 22 | -0.8 | 904.9108 | 2 | 39.7 | 269 | 290 |  | PEAKS DB |
| Y.GRGDGGYGSDSAAAAAAAAAAAAGS(-.98).G | 24.63 | 2021.915 | 25 | -2.2 | 1011.963 | 2 | 43.3 | 269 | 293 | Amidation | PEAKS PTM |
| Y.GRGDGGYGSDSAAAAAAAA.A | 24.61 | 1594.697 | 19 | 0.1 | 798.356 | 2 | 33.3 | 269 | 287 |  | PEAKS DB |
| A.AGSGAGGRGDGGYGW.G | 24.43 | 1323.559 | 15 | -0.2 | 662.7867 | 2 | 34.1 | 220 | 234 |  | PEAKS DB |
| Y.G(+27.99)RGDGGYGSDSAAAAAAAAAAAAGSGAGGRGDGGY.G | 24.3 | 2898.251 | 35 | 0 | 967.091 | 3 | 46.7 | 374 | 408 | Formylation | PEAKS PTM |
| A.AAGAAASSSVRGGGGFY.E | 24.26 | 1484.701 | 17 | 0.2 | 743.3578 | 2 | 33.9 | 123 | 139 |  | PEAKS DB |
| Q.ASHGAGGAAGAAAGAAASSSVRGGGGF.Y | 24.23 | 2129 | 27 | -0.6 | 710.6734 | 3 | 36.1 | 112 | 138 |  | PEAKS DB |
| Y.GSDSAAAAAAAAASGAGGR.G | 24.12 | 1488.692 | 19 | 1 | 745.3538 | 2 | 33.4 | 149 | 167 |  | PEAKS DB |
| Y.GWGDGGYGSDSGAAAA.A | 24.12 | 1397.548 | 16 | -0.5 | 699.7811 | 2 | 39.1 | 233 | 248 |  | PEAKS DB |
| Y.GRGD(-18.01)GGYGSDSAAAAAAAAAAAAGSGAGGRGDGGY.G | 24.08 | 2852.246 | 35 | 0.6 | 951.7564 | 3 | 45.8 | 374 | 408 | Dehydration | PEAKS PTM |
| Y.GSDSAAAAAAAAASGAGGRGH.G | 23.98 | 1682.772 | 21 | 0 | 561.9313 | 3 | 32 | 149 | 169 |  | PEAKS DB |
| Y.EWDYGSY.G | 23.77 | 918.3395 | 7 | -0.4 | 460.1769 | 2 | 43.1 | 339 | 345 |  | PEAKS DB |
| Y.GWGDGGYGSDSGAAAAAAAAAAAAASGAGGRGDGGY(-18.01).G | 23.58 | 2953.261 | 36 | 0.4 | 985.428 | 3 | 54.4 | 233 | 268 | Dehydration | PEAKS PTM |
| H.HDEYVDD(sub S)HGQLVERF.T | 23.48 | 1857.828 | 15 | -1.5 | 620.2824 | 3 | 39.3 | 23 | 37 |  | SPIDER |
| A.SHGAGGAAGAAAGAAASS.S | 23.13 | 1340.607 | 18 | 0.3 | 671.3109 | 2 | 30.5 | 113 | 130 |  | PEAKS DB |
| Y.G(+27.99)SDSAAAAAAAAASGAGGRGHGGY.G | 22.85 | 1987.873 | 24 | -0.4 | 994.9434 | 2 | 38.1 | 149 | 172 | Formylation | PEAKS PTM |
| Y.GRGDGGYGSDSAAAAAAAAAA.A | 22.59 | 1736.771 | 21 | 0 | 869.3929 | 2 | 36.1 | 269 | 289 |  | PEAKS DB |
| Y.VDSHGQLVER(+54.01)F.T | 22.41 | 1339.652 | 11 | 0.3 | 447.5581 | 3 | 37.8 | 27 | 37 | Methylglyoxal-derived hydroimidazolone | PEAKS PTM |
| Y.GSDSGAAAAAAAAAAAAASGAGGRGD(+37.95)GGY.G | 22.31 | 2316.963 | 29 | -1.1 | 580.2474 | 4 | 46.4 | 240 | 268 | Replacement of 2 protons by calcium | PEAKS PTM |
| A.AAAAAASASGAGGSGGSY(+21.98).E | 22.22 | 1404.588 | 18 | 0.2 | 703.3015 | 2 | 30.4 | 321 | 338 | Sodium adduct | PEAKS PTM |
| G.GGFYETHDSY.S | 22.21 | 1174.457 | 10 | 0.1 | 588.2357 | 2 | 33.8 | 136 | 145 |  | PEAKS DB |
| **CHYMOTRYPSIN-TRYPSIN** |  |  |  |  |  |  |  |  |  |  |  |
| **Peptide** | **-10lgP** | **Mass** | **Length** | **ppm** | **m/z** | **z** | **RT** | **Start** | **End** | **PTM** | **Found By** |
| K.NIHHDEYVDSHGQLVER.F | 88.2 | 2046.951 | 17 | 1.7 | 683.323 | 3 | 44.6 | 20 | 36 |  | PEAKS DB |
| K.N(+.98)IHHDEYVDSHGQLVER.F | 85.01 | 2047.935 | 17 | 3.5 | 512.991 | 4 | 50.4 | 20 | 36 | Deamidation (NQ) | PEAKS DB |
| A.KNIHHDEYVDSHGQLVER.F | 83.41 | 2175.046 | 18 | 0.7 | 726.0205 | 3 | 44.5 | 19 | 36 |  | SPIDER |
| R.QASHGAGGAAGAAAGAAASSSVR.G | 82.44 | 1881.904 | 23 | 3.9 | 628.309 | 3 | 46 | 111 | 133 |  | PEAKS DB |
| H.HDEYVDSHGQLVER.F | 80.36 | 1682.765 | 14 | -1.6 | 561.926 | 3 | 47.7 | 23 | 36 |  | PEAKS DB |
| N.IHHDEYVDSHGQLVER.F | 78.84 | 1932.908 | 16 | -1.2 | 645.3069 | 3 | 44.5 | 21 | 36 |  | PEAKS DB |
| K.N(+43.01)IHHDEYVDSHGQLVER.F | 77.93 | 2089.957 | 17 | 1.1 | 523.4952 | 4 | 51.9 | 20 | 36 | Carbamylation | PEAKS PTM |
| K.N(+57.02)IHHDEYVDSHGQLVER.F | 75.05 | 2103.972 | 17 | -2.1 | 526.9974 | 4 | 48.5 | 20 | 36 | Carbamidomethylation (DHKE, X@N-term) | PEAKS PTM |
| K.N(+27.99)IHHDEYVDSHGQLVER.F | 74.73 | 2074.946 | 17 | 0.5 | 519.7421 | 4 | 52.7 | 20 | 36 | Formylation (Protein N-term) | PEAKS PTM |
| A.KN(+.98)IHHDEYVDSHGQLVER.F | 70.81 | 2176.03 | 18 | 2.2 | 545.014 | 4 | 49 | 19 | 36 | Deamidation (NQ) | SPIDER |
| N.I(+43.01)HHDEYVDSHGQLVER.F | 70.44 | 1975.914 | 16 | 0.2 | 494.9841 | 4 | 54.1 | 21 | 36 | Carbamylation | PEAKS PTM |
| R.GDGGYGSGSSAAAAAAAAAAASAAR.R | 70.07 | 2008.92 | 25 | 1.9 | 670.6462 | 3 | 69.1 | 476 | 500 |  | PEAKS DB |
| D.E(-18.01)YVDSHGQLVER.F | 69.82 | 1412.669 | 12 | 2.4 | 707.3408 | 2 | 55.9 | 25 | 36 | Pyro-glu from E | PEAKS PTM |
| K.N(+228.11)IHHDEYVDSHGQLVER.F | 69.46 | 2275.062 | 17 | -1.1 | 569.7701 | 4 | 50.5 | 20 | 36 | 2,4-diacetamido-2,4,6-trideoxyglucopyranose | PEAKS PTM |
| N.I(+225.09)HHDEYVDSHGQLVER.F | 69.15 | 2158.002 | 16 | 8 | 720.3444 | 3 | 49.3 | 21 | 36 | Iminobiotinylation | PEAKS PTM |
| R.Q(-17.03)ASHGAGGAAGAAAGAAASSSVR.G | 68.94 | 1864.878 | 23 | -1.1 | 622.6303 | 3 | 49.8 | 111 | 133 | Pyro-glu from Q | PEAKS PTM |
| K.N(+43.01)(+.98)IHHDEYVDSHGQLVER.F | 68.48 | 2090.941 | 17 | 0.8 | 523.7411 | 4 | 53.1 | 20 | 36 | Carbamylation; Deamidation (NQ) | PEAKS PTM |
| R.GGGGFYETHDSY.S | 68.35 | 1288.5 | 12 | 3.8 | 645.2573 | 2 | 54.9 | 134 | 145 |  | PEAKS DB |
| Y.VDSHGQLVER.F | 67.84 | 1138.573 | 10 | -1.6 | 570.2909 | 2 | 44.5 | 27 | 36 |  | PEAKS DB |
| T.AP(sub K)NIHHDEYVDSHGQLVER.F | 67.4 | 2215.041 | 19 | 2.5 | 554.7669 | 4 | 50.4 | 18 | 36 |  | SPIDER |
| K.N(+226.08)IHHDEYVDSHGQLVER.F | 66.45 | 2273.028 | 17 | 1.7 | 569.2634 | 4 | 52.3 | 20 | 36 | Biotinylation | PEAKS PTM |
| K.N(+.98)IHHD(-18.01)EYVDSHGQLVER.F | 65.66 | 2029.924 | 17 | -0.8 | 508.4862 | 4 | 52 | 20 | 36 | Deamidation (NQ); Dehydration | PEAKS PTM |
| K.N(-17.03)IHHDEYVDSHGQLVER.F | 65.61 | 2029.924 | 17 | -1.4 | 508.4859 | 4 | 55.4 | 20 | 36 | Ammonia-loss (N) | PEAKS PTM |
| K.NIHHDEYVD(-18.01)SHGQLVER.F | 65.04 | 2028.94 | 17 | 2.2 | 508.2417 | 4 | 48.8 | 20 | 36 | Dehydration | PEAKS PTM |
| K.NIHHDEYVDSHGQLVERF.T | 64.24 | 2194.019 | 18 | 1.1 | 549.5107 | 4 | 65.1 | 20 | 37 |  | SPIDER |
| V.DSHGQLVER.F | 62.77 | 1039.505 | 9 | -2 | 520.7568 | 2 | 50.7 | 28 | 36 |  | PEAKS DB |
| E.YVDSHGQLVER.F | 62.7 | 1301.636 | 11 | 3.1 | 434.8859 | 3 | 43.3 | 26 | 36 |  | PEAKS DB |
| R.GDGGYGWGDGGY.G | 62.67 | 1159.421 | 12 | 1.4 | 580.7164 | 2 | 62 | 228 | 239 |  | PEAKS DB |
| N.I(+27.99)HHDEYVDSHGQLVER.F | 62.45 | 1960.903 | 16 | -1 | 491.2308 | 4 | 54.1 | 21 | 36 | Formylation (Protein N-term) | PEAKS PTM |
| R.GGGGFYETHDSYSSY.G | 62.38 | 1625.627 | 15 | 0.9 | 813.8187 | 2 | 60.5 | 134 | 148 |  | SPIDER |
| K.N(+225.09)IHHDEYVDSHGQLVER.F | 62.26 | 2272.044 | 17 | -7.9 | 569.012 | 4 | 52.3 | 20 | 36 | Iminobiotinylation | PEAKS PTM |
| T.AP(sub K)N(+.98)IHHDEYVDSHGQLVER.F | 61.95 | 2216.025 | 19 | 1.7 | 555.0125 | 4 | 52.6 | 18 | 36 | Deamidation (NQ) | SPIDER |
| Y.VDSHGQLVERF.T | 61.92 | 1285.642 | 11 | -0.3 | 429.5528 | 3 | 60.5 | 27 | 37 |  | PEAKS DB |
| K.N(+57.02)(+.98)IHHDEYVDSHGQLVER.F | 61.13 | 2104.956 | 17 | 1.7 | 527.2454 | 4 | 50.1 | 20 | 36 | Carbamidomethylation (DHKE, X@N-term); Deamidation (NQ) | PEAKS PTM |
| K.N(+42.01)IH(+57.02)H(+57.02)DEYVDSHGQLVER.F | 59.52 | 2203.004 | 17 | 5.6 | 551.7595 | 4 | 51.2 | 20 | 36 | Acetylation (Protein N-term); Carbamidomethylation (DHKE, X@N-term) | PEAKS PTM |
| K.N(+42.01)(+203.08)IHHDEYVDSHGQLVER.F | 59.51 | 2292.041 | 17 | -9.6 | 574.01 | 4 | 52 | 20 | 36 | Acetylation (Protein N-term); HexNAcylation (N) | PEAKS PTM |
| K.N(+149.03)IHHDEYVDSHGQLVER.F | 59.47 | 2195.981 | 17 | -8.4 | 549.996 | 4 | 58.6 | 20 | 36 | Benzyl isothiocyanate | PEAKS PTM |
| H.DEYVDSHGQLVER.F | 59.25 | 1545.706 | 13 | 1.9 | 516.2418 | 3 | 49.9 | 24 | 36 |  | PEAKS DB |
| K.NA(sub I)HHDEYVDSHGQLVER.F | 59.1 | 2004.904 | 17 | -4.5 | 669.3033 | 3 | 56 | 20 | 36 |  | SPIDER |
| D.EYVDSHGQLVER.F | 59.04 | 1430.679 | 12 | -0.9 | 477.8982 | 3 | 48.1 | 25 | 36 |  | PEAKS DB |
| T.AKN(+.98)IHHDEYVDSHGQLVER.F | 58.18 | 2247.067 | 19 | -1.1 | 562.7715 | 4 | 46.7 | 18 | 36 | Deamidation (NQ) | SPIDER |
| D.SHGQLVERF.T | 57.98 | 1071.546 | 9 | 0 | 536.7785 | 2 | 56 | 29 | 37 |  | PEAKS DB |
| K.N(+57.02)IH(+57.02)H(+57.02)DEYVDSHGQLVER.F | 57.01 | 2218.015 | 17 | -5 | 555.5064 | 4 | 55.3 | 20 | 36 | Carbamidomethylation (DHKE, X@N-term) | PEAKS PTM |
| A.P(sub K)NIHHDEYVDSHGQLVER.F | 55 | 2144.003 | 18 | -2.1 | 537.0052 | 4 | 53.5 | 19 | 36 |  | SPIDER |
| K.NIHHD(-18.01)EYVDSHGQLVER.F | 54.53 | 2028.94 | 17 | -1.8 | 508.2397 | 4 | 52.5 | 20 | 36 | Dehydration | PEAKS PTM |
| K.N(+27.99)(+.98)IHHDEYVDSHGQLVER.F | 54.22 | 2075.93 | 17 | -1.5 | 519.9871 | 4 | 55.8 | 20 | 36 | Formylation; Deamidation (NQ) | PEAKS PTM |
| I.HHDEYVDSHGQLVER.F | 53.94 | 1819.824 | 15 | -0.9 | 455.9612 | 4 | 45 | 22 | 36 |  | PEAKS DB |
| K.N(-15.01)IHHDEYVDSHGQLVER.F | 53.26 | 2031.94 | 17 | -0.8 | 508.9901 | 4 | 53.8 | 20 | 36 | ISD (z+2)-series | PEAKS PTM |
| A.KNR(sub I)HHDEYVDSHGQLVER.F | 53.23 | 2218.063 | 18 | -3.4 | 555.5192 | 4 | 47.9 | 19 | 36 |  | SPIDER |
| K.NIHHDEY(+95.94)VDSHGQLVER.F | 52.41 | 2142.894 | 17 | 0 | 536.729 | 4 | 54.5 | 20 | 36 | Thiophosphorylation | PEAKS PTM |
| A.KNV(sub I)Y(sub H)HDEYVDSHGQLVER.F | 51.51 | 2187.034 | 18 | 5.5 | 730.0203 | 3 | 47.5 | 19 | 36 |  | SPIDER |
| D.SHGQLVER.F | 51.4 | 924.4777 | 8 | 0.3 | 463.2447 | 2 | 32.6 | 29 | 36 |  | PEAKS DB |
| T.AH(sub K)N(+.98)IHHDEYVDSHGQLVER.F | 51.37 | 2256.031 | 19 | 0.4 | 565.0132 | 4 | 55.3 | 18 | 36 | Deamidation (NQ) | SPIDER |
| N.V(sub I)Y(sub H)HDEYVDSHGQLVER.F | 51.31 | 1944.897 | 16 | 3.5 | 487.2314 | 4 | 50.5 | 21 | 36 |  | SPIDER |
| A.H(sub K)NIHHDEYVDSHGQLVER.F | 51.2 | 2184.01 | 18 | -6.4 | 547.0043 | 4 | 57.9 | 19 | 36 |  | SPIDER |
| T.AKNP(sub I)HHDEYVDSHGQLVER.F | 51.15 | 2230.052 | 19 | -1.1 | 558.5176 | 4 | 49.1 | 18 | 36 |  | SPIDER |
| A.KH(sub N)M(sub I)HHDEYVDSHGQLVER.F | 50.08 | 2216.018 | 18 | 4.7 | 555.0125 | 4 | 52.6 | 19 | 36 |  | SPIDER |
| K.NIHHDE(+258.09)YVDSHGQLVER.F | 49.85 | 2305.036 | 17 | -0.3 | 577.2641 | 4 | 52.2 | 20 | 36 | Diglutamyl | PEAKS PTM |
| T.P(sub A)N(sub K)NIHHDEYVDSHGQLVER.F | 49.68 | 2258.046 | 19 | 1.6 | 565.5178 | 4 | 56 | 18 | 36 |  | SPIDER |
| T.AKN(+.98)V(sub I)HHDEYVDSHGQLVER.F | 49.62 | 2233.051 | 19 | -0.6 | 447.6157 | 5 | 46.4 | 18 | 36 | Deamidation (NQ) | SPIDER |
| K.N(+42.01)IHHDEY(+31.99)VDSHGQLVER.F | 49.25 | 2120.951 | 17 | 2.1 | 531.2444 | 4 | 51.5 | 20 | 36 | Acetylation (Protein N-term); Dihydroxy | PEAKS PTM |
| N.I(+362.14)HHDEYVDSHGQLVER.F | 48.94 | 2295.044 | 16 | -7.9 | 574.7619 | 4 | 53.3 | 21 | 36 | Nucleophilic addtion to cytopiloyne | PEAKS PTM |
| T.T(sub A)P(sub K)NIHHDEYVDSHGQLVER.F | 48.88 | 2245.051 | 19 | -0.1 | 562.2681 | 4 | 51.1 | 18 | 36 |  | SPIDER |
| Y.GSDSAAAAAAAAASGAGGR.G | 48.26 | 1488.692 | 19 | -1 | 745.3498 | 2 | 53.2 | 149 | 167 |  | PEAKS DB |
| I.HHD(+356.19)EYVDSHGQLVER.F | 48.22 | 2176.012 | 15 | 7.4 | 436.2114 | 5 | 49 | 22 | 36 | Biotin polyethyleneoxide amine | PEAKS PTM |
| K.NIH(+142.11)HDEYVDSHGQLVER.F | 48.06 | 2189.061 | 17 | 5.1 | 548.2735 | 4 | 45 | 20 | 36 | Diphthamide | PEAKS PTM |
| N.IHHDEYVD(+37.95)SHGQLVER.F | 47.87 | 1970.855 | 16 | 0.4 | 493.7195 | 4 | 46.9 | 21 | 36 | Replacement of 2 protons by calcium | PEAKS PTM |
| K.N(+.98)IHHD(+43.99)EYVDSHGQLVER.F | 47.73 | 2091.925 | 17 | 2.9 | 523.9882 | 4 | 56.8 | 20 | 36 | Deamidation (NQ); Carboxylation (DKW) | PEAKS PTM |
| K.N(+42.01)IHHD(+43.99)EYVDSHGQLVER.F | 47.46 | 2132.951 | 17 | 1 | 534.2438 | 4 | 54.1 | 20 | 36 | Acetylation (Protein N-term); Carboxylation (DKW) | PEAKS PTM |
| A.KNIHHDEYVDS(-18.01)HGQL.V | 47.37 | 1772.823 | 15 | -0.5 | 591.946 | 3 | 44.5 | 19 | 33 | Dehydration | PEAKS PTM |
| A.KH(sub N)E(sub I)HHDEYVDSHGQLVER.F | 44.44 | 2214.02 | 18 | -5.2 | 554.5076 | 4 | 55.6 | 19 | 36 |  | SPIDER |
| R.GDGGYGWGD(+57.02)GGY.G | 44.4 | 1216.442 | 12 | 1.7 | 609.2273 | 2 | 59.9 | 228 | 239 | Carbamidomethylation (DHKE, X@N-term) | PEAKS PTM |
| R.GGGGFYETHDS(-18.01)Y.S | 43.77 | 1270.489 | 12 | 1.6 | 636.2506 | 2 | 58.8 | 134 | 145 | Dehydration | PEAKS PTM |
| K.N(+42.01)IHHDEYVDSHGQ(+.98)LVER.F | 43.51 | 2089.945 | 17 | 6.4 | 523.4952 | 4 | 51.9 | 20 | 36 | Acetylation (Protein N-term); Deamidation (NQ) | PEAKS PTM |
| K.NIHHD(+43.99)EYVDSHGQLVER.F | 43.31 | 2090.941 | 17 | 0.8 | 523.7411 | 4 | 53.1 | 20 | 36 | Carboxylation (DKW) | PEAKS PTM |
| T.AKNT(sub I)HHDEYVDSHGQLVER.F | 43.27 | 2234.046 | 19 | -3.7 | 559.5149 | 4 | 50.7 | 18 | 36 |  | SPIDER |
| R.GDGGYGSGSSAAAAAAAAAAASAARR.A | 43.11 | 2165.021 | 26 | 2.3 | 722.6801 | 3 | 68.4 | 476 | 501 |  | PEAKS DB |
| R.GDGGYGWGD(+21.98)GGY.G | 43.1 | 1181.403 | 12 | 0 | 591.7065 | 2 | 62 | 228 | 239 | Sodium adduct | PEAKS PTM |
| R.GDGGYGR.G | 42.75 | 680.2878 | 7 | -2.3 | 341.1492 | 2 | 22.6 | 264 | 270 |  | PEAKS DB |
| R.GGGGFYETHDSYS.S | 42.69 | 1375.532 | 13 | 2.2 | 688.7722 | 2 | 52.2 | 134 | 146 |  | SPIDER |
| K.NIHHDEYVDS(+79.96)HGQLVER.F | 41.8 | 2126.908 | 17 | 5 | 532.735 | 4 | 49.3 | 20 | 36 | Sulfation | PEAKS PTM |
| R.GDGGYGSGSSAAAAAAAAAAASAAR(+14.02).R | 41.69 | 2022.935 | 25 | 7.2 | 675.3217 | 3 | 61.9 | 476 | 500 | Methylation(KR) | PEAKS PTM |
| D.E(+43.01)YVDSHGQLVER.F | 41 | 1473.685 | 12 | -2.3 | 737.8455 | 2 | 54.4 | 25 | 36 | Carbamylation | PEAKS PTM |
| K.N(+42.01)IHHDEYVDSH(+15.99)GQLVER.F | 38.59 | 2104.956 | 17 | 1.7 | 527.2454 | 4 | 50.1 | 20 | 36 | Acetylation (Protein N-term); Oxidation (HW) | PEAKS PTM |
| A.KH(sub N)T(sub I)HHDEYVDSHGQLVER.F | 38.49 | 2186.025 | 18 | -5 | 547.509 | 4 | 60.2 | 19 | 36 |  | SPIDER |
| K.NIHHDEYVDF(sub S)HGQLVER.F | 37.61 | 2106.987 | 17 | 4.3 | 527.7545 | 4 | 55.9 | 20 | 36 |  | SPIDER |
| T.T(sub A)H(sub K)NIHHDEYVDSHGQLVER.F | 37.33 | 2285.057 | 19 | -2.8 | 572.2681 | 4 | 50.3 | 18 | 36 |  | SPIDER |
| I.H(+226.08)HDEYVDSHGQLVER.F | 37.25 | 2045.901 | 15 | 5.8 | 512.4838 | 4 | 55.5 | 22 | 36 | Biotinylation | PEAKS PTM |
| K.NIHHDEY(-18.01)VDSHGQLVER.F | 37.05 | 2028.94 | 17 | 2.2 | 508.2417 | 4 | 48.8 | 20 | 36 | Dehydration | PEAKS PTM |
| T.S(sub A)KN(+.98)IHHDEYVDSHGQLVER.F | 36.08 | 2263.062 | 19 | -2.4 | 566.7694 | 4 | 46.6 | 18 | 36 | Deamidation (NQ) | SPIDER |
| A.KN(+.98)H(sub I)HHDEYVDSHGQLVER.F | 36.05 | 2200.005 | 18 | 10.7 | 551.0125 | 4 | 61.6 | 19 | 36 | Deamidation (NQ) | SPIDER |
| K.N(+.98)IHHDEY(+162.05)VDSHGQLVER.F | 35.98 | 2209.988 | 17 | -4.6 | 553.4998 | 4 | 60.9 | 20 | 36 | Deamidation (NQ); Hexose (NSY) | PEAKS PTM |
| K.N(+.98)IHHDEY(+79.97)VDSHGQ(+.98)LVER.F | 35.98 | 2128.885 | 17 | -0.7 | 533.2263 | 4 | 53.2 | 20 | 36 | Deamidation (NQ); Phosphorylation (STY) | PEAKS PTM |
| Y.GSGSSAAAAAAAAAAASAARR.A | 35.77 | 1715.866 | 21 | 1.4 | 572.9615 | 3 | 62.7 | 481 | 501 |  | PEAKS DB |
| D.EYVDSHGQLVERF.T | 35.48 | 1577.747 | 13 | 0.3 | 526.9214 | 3 | 65.4 | 25 | 37 |  | SPIDER |
| R.GGGGFYETH.D | 34.41 | 923.3773 | 9 | 2.5 | 462.6955 | 2 | 43.3 | 134 | 142 |  | PEAKS DB |
| W.GDGGYGSDPGAAAAAAAAAAAAASGAR.G | 33.98 | 2174.994 | 27 | 0.7 | 726.0032 | 3 | 58.9 | 447 | 473 |  | PEAKS DB |
| R.GGGGFYETHD(-18.01)SY.S | 33.96 | 1270.489 | 12 | 0.7 | 636.2501 | 2 | 54.9 | 134 | 145 | Dehydration | PEAKS PTM |
| A.Y(sub K)NM(sub I)HHDEYVDSHGQLVER.F | 33.17 | 2227.971 | 18 | -8.2 | 557.9934 | 4 | 45.8 | 19 | 36 |  | SPIDER |
| K.NIHHDEYVDS(+121.04)HGQLVER.F | 33.07 | 2167.986 | 17 | -9.8 | 542.9966 | 4 | 59.4 | 20 | 36 | Phosphorylation to pyridyl thiol | PEAKS PTM |
| A.KN(+.98)V(sub I)Y(sub H)HDEYVDSHGQLVER.F | 31.9 | 2188.019 | 18 | 9.3 | 548.0151 | 4 | 48.9 | 19 | 36 | Deamidation (NQ) | SPIDER |
| A.H(sub K)N(+.98)V(sub I)HHDEYVDSHGQLVER.F | 31.31 | 2170.978 | 18 | 6.8 | 543.7536 | 4 | 55.4 | 19 | 36 | Deamidation (NQ) | SPIDER |
| R.GDGGYGWGD(+57.02)GGY(-.98).G | 30.83 | 1215.458 | 12 | 1 | 608.7349 | 2 | 57.8 | 228 | 239 | Carbamidomethylation (DHKE, X@N-term); Amidation | PEAKS PTM |
| Y.GSDSAAAAAAAAAAAAAGSGAGGVGGGYGR.G | 30.81 | 2320.079 | 30 | -1.5 | 774.3632 | 3 | 74 | 346 | 375 |  | PEAKS DB |
| R.GHGGYGSDSAAAAAAAAAAAAAAASGAG.G | 30.33 | 2172.978 | 28 | 4.5 | 725.3342 | 3 | 69.8 | 168 | 195 |  | PEAKS DB |

Table S7. List of unique peptides detected from *A. yamamai* digested with trypsin, FA-trypsin, chymotrypsin, and chymotrypsin-trypsin at False Discovery Rate ≤1%

| **TRYPSIN** |  |  |  |  |  |  |  |  |  |  |  |
| --- | --- | --- | --- | --- | --- | --- | --- | --- | --- | --- | --- |
| **Peptide** | **-10lgP** | **Mass** | **Length** | **ppm** | **m/z** | **z** | **RT** | **Start** | **End** | **PTM** | **Found By** |
| R.AAGSAAAAAAAAAAAAASGAGGSGR.G | 61.22 | 1856.909 | 25 | 0.9 | 929.4625 | 2 | 49.4 | 908 | 932 |  | PEAKS DB |
| R.AAGSAAAAAAAAAAAAASGAGR.S | 59.83 | 1655.834 | 22 | -0.5 | 828.9238 | 2 | 49.8 | 2351 | 2372 |  | PEAKS DB |
| R.SSGSAAAAAAAAAAAAASGAGGSGGG.Y | 43.63 | 1846.841 | 26 | -1.1 | 924.4265 | 2 | 53.4 | 2540 | 2565 |  | PEAKS DB |
| R.GD(+15.99)GGY(+15.99)GSGSSAAAAAAAAAAAAASGAGG.S | 42.59 | 2126.91 | 28 | -12 | 1064.45 | 2 | 62.4 | 2010 | 2037 | Oxidation or Hydroxylation | PEAKS DB |
| G.D(+15.99)SGYGSGSSAAAAAAAAAAAR.R | 38.51 | 1768.798 | 21 | -14 | 885.3939 | 2 | 50.2 | 2513 | 2533 | Oxidation or Hydroxylation | PEAKS DB |
| R.AAGSAAAAAAAAAAAAASGAGGSGR(-43.05).G | 37.98 | 1813.855 | 25 | 5.4 | 907.9398 | 2 | 52.9 | 908 | 932 | Arginine oxidation to glutamic semialdehyde | PEAKS PTM |
| S.E(sub A)AAAAAAAAAAAGSGAGGR.G | 37.81 | 1470.717 | 19 | 0.8 | 736.3666 | 2 | 53.4 | 182 | 200 |  | SPIDER |
| R.AAGSAAAAAAAAAAAAASGAGGSGGS(+117.00)Y.G | 37.12 | 2124.922 | 27 | -5.4 | 1063.463 | 2 | 60.8 | 1061 | 1087 | Phospho-propargylamine | PEAKS PTM |
| R.AAGSAAAAAAAAAAAAASGAGGS(+117.00)GGSY.G | 35.94 | 2124.922 | 27 | -7.5 | 1063.46 | 2 | 60.8 | 1061 | 1087 | Phospho-propargylamine | PEAKS PTM |
| G.DSGY(+15.99)GSGSSAAAAAAAAAAAR.R | 33.87 | 1768.798 | 21 | -12 | 590.5994 | 3 | 50.1 | 2513 | 2533 | Oxidation or Hydroxylation | PEAKS DB |
| R.GDSGY(+29.97)GSGSSAAAAAAAAAAAR.R | 32.96 | 1839.798 | 22 | 8.1 | 920.9138 | 2 | 52.5 | 2512 | 2533 | Quinone | PEAKS PTM |
| G.D(+88.00)SGYGSGSSAAAAAAAAAAAR.R | 32.92 | 1840.801 | 21 | -5.4 | 921.4028 | 2 | 61.4 | 2513 | 2533 | 3-sulfanylpropanoyl | PEAKS PTM |
| G.SSAAAAAAAAAAAAASGAGG.S | 32.65 | 1444.691 | 20 | 8.3 | 723.3586 | 2 | 50.3 | 2018 | 2037 |  | PEAKS DB |
| G.YGSGS(-20.03)SAAAAAAAAAAAR.R | 32.36 | 1473.696 | 18 | -9.8 | 737.8481 | 2 | 58 | 2516 | 2533 | Formation of five membered aromatic heterocycle | PEAKS PTM |
| G.SSAAAAAAAAAAAE(sub A)SR.R | 32.11 | 1345.659 | 16 | -0.1 | 673.8365 | 2 | 50.4 | 2745 | 2760 |  | SPIDER |
| **FA-TRYPSIN** |  |  |  |  |  |  |  |  |  |  |  |
| **Peptide** | **-10lgP** | **Mass** | **Length** | **ppm** | **m/z** | **z** | **RT** | **Start** | **End** | **PTM** | **Found By** |
| R.AAGSAAAAAAAAAAAAASGAGGSGR.G | 51.02 | 1856.909 | 25 | 0.7 | 929.4623 | 2 | 49.4 | 908 | 932 |  | PEAKS DB |
| R.AAGSAAAAAAAAAAAAASGAGR.S | 49.91 | 1655.834 | 22 | 0.1 | 828.9243 | 2 | 49.8 | 2351 | 2372 |  | PEAKS DB |
| R.AAGSAAAAAAAAAAAAASGAGGSGGSY(+117.00).G | 38.96 | 2124.922 | 27 | -7.8 | 1063.46 | 2 | 60.8 | 1061 | 1087 | Phospho-propargylamine | PEAKS PTM |
| Y.GSDSAAAAAAAAAAAAAGSGAGR.A | 37.72 | 1772.84 | 23 | 1.2 | 887.4284 | 2 | 49.4 | 486 | 508 |  | PEAKS DB |
| R.A(+43.01)AGSAAAAAAAAAAAAASGAGGSGR.G | 37.02 | 1899.915 | 25 | -0.2 | 950.9644 | 2 | 60.9 | 908 | 932 | Carbamylation | PEAKS PTM |
| R.A(+43.01)AGSAAAAAAAAAAAAASGAGR.S | 36.89 | 1698.84 | 22 | -0.7 | 850.4265 | 2 | 61.5 | 2351 | 2372 | Carbamylation | PEAKS PTM |
| D.IY(+44.99)EEDVVINR.V | 36.56 | 1293.62 | 10 | 0.3 | 647.8175 | 2 | 47.9 | 76 | 85 | Oxidation to nitro | PEAKS PTM |
| R.GD(+15.99)GGY(+15.99)GSGSSAAAAAAAAAAAAASGAGG.S | 36.34 | 2126.91 | 28 | -13 | 1064.449 | 2 | 62.4 | 2010 | 2037 | Oxidation or Hydroxylation | PEAKS DB |
| A.AAAAAAAAAGSGAGR.A | 34.36 | 1142.579 | 15 | 0.1 | 572.2969 | 2 | 32.9 | 494 | 508 |  | PEAKS DB |
| R.AAGSAAAAAAAAAAAAASGAGGSGR(-43.05).G | 33.61 | 1813.855 | 25 | 6.4 | 907.9408 | 2 | 53.1 | 908 | 932 | Arginine oxidation to glutamic semialdehyde | PEAKS PTM |
| A.AAAAAAAGSGAGGAGGD.Y | 31.61 | 1201.532 | 17 | 0.3 | 601.7736 | 2 | 33.3 | 649 | 665 |  | PEAKS DB |
| R.AAGSAAAAAAAAAAAAASGAGR(+15.99)SG(+15.99).G | 31.52 | 1831.877 | 24 | 0.9 | 916.9467 | 2 | 54.5 | 2351 | 2374 | Oxidation or Hydroxylation | PEAKS DB |
| Y.GSGSSAAAAAAAAAAAASR.R | 30.32 | 1488.728 | 19 | 0.3 | 745.3715 | 2 | 43.8 | 2742 | 2760 |  | PEAKS DB |
| D.SAAGAAAAAAAAAASGAGGR.G | 29.79 | 1499.744 | 20 | 0.6 | 750.8798 | 2 | 44.3 | 1990 | 2009 |  | PEAKS DB |
| G.D(+15.99)SGYGSGSSAAAAAAAAAAAR.R | 29.7 | 1768.798 | 21 | -13 | 885.3946 | 2 | 50.1 | 2513 | 2533 | Oxidation or Hydroxylation | PEAKS DB |
| A.AAAAAAAAGSGAGGAGGD.Y | 29.68 | 1272.569 | 18 | 0.6 | 637.2923 | 2 | 33.9 | 648 | 665 |  | PEAKS DB |
| G.DSGY(+15.99)GSGSSAAAAAAAAAAAR.R | 28.8 | 1768.798 | 21 | -14 | 590.5983 | 3 | 50.2 | 2513 | 2533 | Oxidation or Hydroxylation | PEAKS DB |
| Y.E(+43.01)EDVVINR.V | 28.68 | 1015.493 | 8 | -0.1 | 508.7539 | 2 | 45.4 | 78 | 85 | Carbamylation | PEAKS PTM |
| R.AAGS(+59.02)AAAAAAAAAAAAASGAGGSGR.G | 28.22 | 1915.928 | 25 | -8.2 | 958.9636 | 2 | 62.3 | 908 | 932 | Aminoethylcysteine | PEAKS PTM |
| Y.GSDSAAAAAAAAAAAAAGSGAGGAGGD.Y | 28.19 | 1973.867 | 27 | 2.3 | 987.9433 | 2 | 55.6 | 639 | 665 |  | PEAKS DB |
| R.AAGSAAAAAAAAAAAAASGAGGSGGS(+14.02)YG.W | 27.85 | 2078.962 | 28 | -13 | 693.9857 | 3 | 58.2 | 1061 | 1088 | Methylation(others) | PEAKS PTM |
| G.D(+88.00)SGYGSGSSAAAAAAAAAAAR.R | 27.72 | 1840.801 | 21 | -3.3 | 921.4047 | 2 | 61.3 | 2513 | 2533 | 3-sulfanylpropanoyl | PEAKS PTM |
| S.G(+42.01)SSAAAAAAAAAAAR.R | 27.37 | 1228.616 | 15 | -0.1 | 615.3152 | 2 | 51.6 | 2519 | 2533 | Acetylation (N-term) | PEAKS PTM |
| Y.EEDVVINR.V | 27.33 | 972.4876 | 8 | 0 | 487.2511 | 2 | 36.6 | 78 | 85 |  | PEAKS DB |
| D.SAAAAAAAAAAAAAGSGAGR.A | 26.88 | 1513.76 | 20 | 0.5 | 757.8875 | 2 | 44.9 | 489 | 508 |  | PEAKS DB |
| R.AAGSAAAAAAAAAAAAASGAGGSGGS(+59.02)Y.G | 26.87 | 2066.944 | 27 | 10.3 | 1034.49 | 2 | 59.9 | 1061 | 1087 | Aminoethylcysteine | PEAKS PTM |
| E.EDVVINR.V | 26.78 | 843.445 | 7 | -0.2 | 422.7297 | 2 | 35.2 | 79 | 85 |  | PEAKS DB |
| R.A(+43.01)AGS(+79.97)AAAAAAAAAAAAASGAGR.S | 26.39 | 1778.806 | 22 | 14.7 | 890.4233 | 2 | 49.8 | 2351 | 2372 | Carbamylation; Phosphorylation (STY) | PEAKS PTM |
| R.GDSGYGSGS(+86.00)SAAAAAAAAAAAR.R | 25.91 | 1895.825 | 22 | 7.1 | 948.9262 | 2 | 50.6 | 2512 | 2533 | Malonylation | PEAKS PTM |
| Y.S(+42.01)SDSAAAAAAAAAAAAAGSGAGGV.G | 25.76 | 1844.85 | 24 | -12 | 923.4211 | 2 | 65.5 | 2385 | 2408 | Acetylation (N-term) | PEAKS PTM |
| G.SES(-18.01)AAAAAAAAAAGSGAGGV.G | 25.67 | 1498.701 | 20 | 8.8 | 750.3644 | 2 | 66.7 | 397 | 416 | Dehydration | PEAKS PTM |
| S.DSAAGAAAAAAAAAASGAGGRG.D | 25.44 | 1671.792 | 22 | 0.7 | 836.9041 | 2 | 48.5 | 1989 | 2010 |  | PEAKS DB |
| R.A(+42.01)AGSAAAAAAAAAAAAASGAGR.S | 25.27 | 1697.845 | 22 | -6.3 | 849.9242 | 2 | 50.3 | 2351 | 2372 | Acetylation (N-term) | PEAKS PTM |
| S.ES(-2.02)AAAAAAAAAAGSGAGGVG.G | 25.25 | 1484.685 | 20 | 8.9 | 743.3566 | 2 | 62.3 | 398 | 417 | 2-amino-3-oxo-butanoic_acid | PEAKS PTM |
| R.AAGS(+59.02)AAAAAAAAAAAAASGAGR.S | 25.21 | 1714.853 | 22 | -9.9 | 858.4254 | 2 | 61.8 | 2351 | 2372 | Aminoethylcysteine | PEAKS PTM |
| G.S(+43.01)GSSAAAAAAAAAAAR.R | 25.16 | 1316.643 | 16 | 0 | 659.3289 | 2 | 50.2 | 2518 | 2533 | Carbamylation | PEAKS PTM |
| Y.GSGSS(+86.00)AAAAAAAAAAAR.R | 24.69 | 1416.659 | 17 | -0.3 | 709.3367 | 2 | 59 | 2517 | 2533 | Malonylation | PEAKS PTM |
| G.S(-15.01)SAAAAAAAAAAAR.R | 24.59 | 1114.573 | 14 | -0.2 | 558.2936 | 2 | 53 | 2520 | 2533 | ISD (z+2)-series | PEAKS PTM |
| Y.GSDSAAGAAAAAAAAAASGAGGR.G | 24.17 | 1758.825 | 23 | -0.5 | 880.4191 | 2 | 47.6 | 1987 | 2009 |  | PEAKS DB |
| G.YGSGS(-20.03)SAAAAAAAAAAAR.R | 24.08 | 1473.696 | 18 | -9.9 | 737.848 | 2 | 58.3 | 2516 | 2533 | Formation of five membered aromatic heterocycle | PEAKS PTM |
| **CHYMOTRYPSIN** |  |  |  |  |  |  |  |  |  |  |  |
| **Peptide** | **-10lgP** | **Mass** | **Length** | **ppm** | **m/z** | **z** | **RT** | **Start** | **End** | **PTM** | **Found By** |
| A.GSAAAAAAAAAAGAGASRPVGIY.G | 45.48 | 1900.976 | 23 | 0.1 | 634.6658 | 3 | 44.2 | 2771 | 2793 |  | PEAKS DB |
| Y.GSESAAAAAAAAAAGSGAGGVGGGY.G | 45.24 | 1907.861 | 25 | -0.4 | 954.9373 | 2 | 45.6 | 396 | 420 |  | PEAKS DB |
| D.SAAAAAAAAAAAAASGAGGRGD.G | 45.21 | 1685.808 | 22 | 0.3 | 562.9434 | 3 | 41.3 | 2152 | 2173 |  | PEAKS DB |
| Y.GSDSAAAAAAAAAAASGAGGSGGGY.G | 44.83 | 1895.825 | 25 | 0.8 | 948.9203 | 2 | 45.8 | 521 | 545 |  | PEAKS DB |
| D.SAAAAAAAAAAAAAGSGAGGAGGDY.G | 44.24 | 1877.85 | 25 | -0.6 | 626.957 | 3 | 46.9 | 642 | 666 |  | PEAKS DB |
| R.AAGSAAAAAAAAAAAAASGAGRSGGGY.G | 41.23 | 2076.994 | 27 | -0.6 | 693.3381 | 3 | 46.3 | 2351 | 2377 |  | PEAKS DB |
| D.GGYGSDSAAAAAAAAAAAAASGAGGRGD.G | 39.54 | 2221.995 | 28 | 1.4 | 1112.006 | 2 | 47.5 | 2146 | 2173 |  | PEAKS DB |
| R.AAGSAAAAAAAAAAAAASGAGGSGR.G | 38.37 | 1856.909 | 25 | -0.6 | 619.9765 | 3 | 43.5 | 908 | 932 |  | PEAKS DB |
| D.GGYGSDSAAAAAAAAAAAAAGSGAGRAGGD.Y | 38.04 | 2350.053 | 30 | -0.4 | 784.358 | 3 | 46.8 | 483 | 512 |  | PEAKS DB |
| D.SAAGAAAAAAAAAASGAGGRGD.G | 37.52 | 1671.792 | 22 | -0.3 | 836.9032 | 2 | 39.5 | 1990 | 2011 |  | PEAKS DB |
| Y.GSDSAAAAAAAAAAAASSGAGGSGGGY.G | 37.03 | 2053.894 | 27 | -0.2 | 1027.954 | 2 | 48.4 | 2574 | 2600 |  | PEAKS DB |
| Y.GGYGSDSAAAAAAAAAAAGSGAG.G | 35.47 | 1751.771 | 23 | -0.3 | 876.8925 | 2 | 46.6 | 2481 | 2503 |  | PEAKS DB |
| D.SAAAAAAAAAAAAAGSGAGRAGGD.Y | 35.36 | 1813.867 | 24 | 0 | 605.6295 | 3 | 40.5 | 489 | 512 |  | PEAKS DB |
| D.SAAAAAAAAAAAGSGAGGVGGGYGRGD.S | 33.55 | 2090.973 | 27 | -0.3 | 697.998 | 3 | 39.3 | 2487 | 2513 |  | PEAKS DB |
| D.GGYGSDSAAAAAAAAAAAAAGSGAGGAGGD.Y | 33.27 | 2250.974 | 30 | -0.3 | 1126.494 | 2 | 50.8 | 636 | 665 |  | PEAKS DB |
| D.SAAAAAAAAAAAAAGSGAGGAGGD.Y | 32.88 | 1714.787 | 24 | -0.3 | 572.6028 | 3 | 43.8 | 642 | 665 |  | PEAKS DB |
| Y.GGYGSDSAAAAAAAAAASAGSGAGG.V | 32.45 | 1895.825 | 25 | -0.1 | 948.9194 | 2 | 46.1 | 2444 | 2468 |  | PEAKS DB |
| W.GDGGYGSDSAAAAAAAAAAAAASGAGGAGGY.G | 32.08 | 2414.037 | 31 | 1.4 | 805.6874 | 3 | 55.7 | 259 | 289 |  | PEAKS DB |
| Y.GSDSAAAAAAAAAAAAASGAGGAG.G | 30.86 | 1744.798 | 24 | -0.2 | 582.6063 | 3 | 48.1 | 264 | 287 |  | PEAKS DB |
| D.SAAAAAAAAAAAAASGAGGAGGYGGYGSD.S | 30.76 | 2241.989 | 29 | -0.2 | 748.3366 | 3 | 50 | 267 | 295 |  | PEAKS DB |
| D.GGYGSDSAAAAAAAAAAASGAGGSGGGY.G | 29.18 | 2172.931 | 28 | -0.6 | 1087.472 | 2 | 48.9 | 518 | 545 |  | PEAKS DB |
| Y.GSESAAAAAAAAAAGSGAGGVG.G | 28.4 | 1630.755 | 22 | 0.6 | 816.3851 | 2 | 42.3 | 396 | 417 |  | PEAKS DB |
| W.GDGGYGSDSAAAAAAAAAAAAAGSGAGRA.G | 28.18 | 2293.032 | 29 | -0.5 | 765.3508 | 3 | 47.7 | 481 | 509 |  | PEAKS DB |
| Y.GSDSAAAAAAAAAAAAASGAGGRGD.G | 27.04 | 1944.888 | 25 | -1 | 973.4505 | 2 | 41.6 | 2149 | 2173 |  | PEAKS DB |
| Y.GGYGSDSAAAAAAAAAASAGSGAGG(-.98).V | 26.73 | 1894.841 | 25 | -0.3 | 948.4272 | 2 | 45.2 | 2444 | 2468 | Amidation | PEAKS PTM |
| Y.GGYGSDSAAAAAAAAAAAGSGAGGVGGGY(-.98).G | 26.72 | 2241.005 | 29 | 1.2 | 1121.511 | 2 | 53.3 | 2481 | 2509 | Amidation | PEAKS PTM |
| Y.GGYGSDSAAAAAAAAAAAGSGA(-.98).G | 26.34 | 1693.766 | 22 | -0.4 | 847.8897 | 2 | 45.9 | 2481 | 2502 | Amidation | PEAKS PTM |
| D.VVINRVPGASSSAAAASSASAGS(-.98).G | 26.25 | 2015.04 | 23 | 0.4 | 1008.528 | 2 | 34.5 | 81 | 103 | Amidation | PEAKS PTM |
| Y.GSDSAAAAAAAAAAAAASGAGGAGGY(-.98).G | 26.24 | 1963.898 | 26 | -0.4 | 982.956 | 2 | 51.2 | 264 | 289 | Amidation | PEAKS PTM |
| Y.GSDSAAAAAAAAAAAAASGAGGAGGY.G | 26.21 | 1964.882 | 26 | -0.4 | 655.9678 | 3 | 52.3 | 264 | 289 |  | PEAKS DB |
| A.GHDRAAGSAAAAAAAAAAAAASGAGGSGGSY.G | 26.16 | 2473.133 | 31 | -0.7 | 825.3843 | 3 | 44.3 | 1057 | 1087 |  | PEAKS DB |
| Y.GSDSAAAAAAAAAAAAASGAGGAGG.Y | 26.16 | 1801.819 | 25 | 0.5 | 901.9172 | 2 | 47.6 | 264 | 288 |  | PEAKS DB |
| W.GDGGYGSDSAAAAAAAAAAAAAGSGAGGAGGDY.G | 26.05 | 2586.085 | 33 | -1.5 | 863.0345 | 3 | 54 | 634 | 666 |  | PEAKS DB |
| Y.GSDSAAAAAAAAAAAAAGSGAGGAGGDY.G | 25.68 | 2136.931 | 28 | 0.3 | 713.3177 | 3 | 50.5 | 639 | 666 |  | PEAKS DB |
| Y.GGYGSDSAAAAAAAAAAAGSGAGGVGGGY.G | 24.99 | 2241.989 | 29 | 0.3 | 748.337 | 3 | 54.5 | 2481 | 2509 |  | PEAKS DB |
| Y.GGYGSDSAAAAAAAAAAAAASSGAGGAGGGYGW(-.98).G | 24.9 | 2628.159 | 33 | -1.6 | 877.0588 | 3 | 44.5 | 1764 | 1796 | Amidation | PEAKS PTM |
| D.GGYGSDSAAAAAAAAAAASGAGGSGGGY(-.98).G | 24.7 | 2171.947 | 28 | 0 | 1086.981 | 2 | 47.5 | 518 | 545 | Amidation | PEAKS PTM |
| G.SGFYETHDSY.S | 24.48 | 1204.467 | 10 | -1 | 603.2403 | 2 | 34.5 | 137 | 146 |  | PEAKS DB |
| A.AAAAAAGAGASRPVGIY.G | 24.46 | 1472.774 | 17 | -0.9 | 737.3934 | 2 | 35.3 | 2777 | 2793 |  | PEAKS DB |
| D.GGYGSDSAAAAAAAAAAAAASGAGGAGGY(-.98).G | 23.78 | 2241.005 | 29 | 0.9 | 748.0095 | 3 | 53.2 | 261 | 289 | Amidation | PEAKS PTM |
| Y.GGGDGGYGSGSSAAAAAAAAAAAAR.R | 23.74 | 1964.894 | 25 | 0.9 | 655.9724 | 3 | 42.8 | 421 | 445 |  | PEAKS DB |
| D.VVINRVPGASSSAAAASSASAG.S | 22.79 | 1928.992 | 22 | 2.3 | 965.5052 | 2 | 35.4 | 81 | 102 |  | PEAKS DB |
| Y.GGYGSDSAAAAAAAAAAAAASSGAGGAGGGYGW.G | 22.69 | 2629.143 | 33 | 5.1 | 877.3927 | 3 | 46 | 1764 | 1796 |  | PEAKS DB |
| Y.S(+163.05)SYGSESAAAAAAAAAAGSGAGGVGGGYGGGD.G | 22.08 | 2694.125 | 32 | 5.4 | 899.0539 | 3 | 49 | 393 | 424 | Phenethyl isothiocyanate | PEAKS PTM |
| A.AAAAAGAGASRPVGIY.G | 21.78 | 1401.737 | 16 | 0.8 | 701.876 | 2 | 34.6 | 2778 | 2793 |  | PEAKS DB |
| R.AAGSAAAAAAAAAAAAASGAGR.S | 21.5 | 1655.834 | 22 | -0.4 | 552.9517 | 3 | 44 | 2351 | 2372 |  | PEAKS DB |
| D.VVINRVPGASSSAAAASSA.S | 21.32 | 1713.901 | 19 | 1.2 | 857.9587 | 2 | 35 | 81 | 99 |  | PEAKS DB |
| D.GGYGSDSAAAAAAAAAAAAAGSGAGRAGGDY(-.98).G | 21.31 | 2512.133 | 31 | 2.7 | 838.3871 | 3 | 47.5 | 483 | 513 | Amidation | PEAKS PTM |
| D.RAYGAGSAAAAAAAAAAGAGASRPVGIYGTD.D | 20.69 | 2692.332 | 31 | -1 | 898.4503 | 3 | 44.7 | 2766 | 2796 |  | PEAKS DB |
| D.GGYGSDSAAAAAAAAAAASGAGGAGG.G | 20.65 | 1936.851 | 26 | -1 | 969.4318 | 2 | 46.8 | 671 | 696 |  | PEAKS DB |
| D.SAAAAAAAAAAAAASGAGGR(+14.02)GD.G | 20.47 | 1699.824 | 22 | 0.2 | 567.6153 | 3 | 41.9 | 2152 | 2173 | Methylation(KR) | PEAKS PTM |
| W.GDGGYGSDSAAAAAAAAAAAASGAGG(-.98).A | 20.06 | 1993.872 | 26 | 0.5 | 997.944 | 2 | 48.6 | 787 | 812 | Amidation | PEAKS PTM |
| W.GDGGYGSDSAAAAAAAAAAAAASGAGGRGD.G | 19.98 | 2394.043 | 30 | 3.5 | 799.0245 | 3 | 48.6 | 2144 | 2173 |  | PEAKS DB |
| Y.GGYGSDSAAAAAAAAAASAGS(+79.97)GAGGVGGG.Y | 19.94 | 2245.924 | 29 | 0.6 | 749.6489 | 3 | 44.3 | 2444 | 2472 | Phosphorylation (STY) | PEAKS PTM |
| **CHYMOTRYPSIN-TRYPSIN** |  |  |  |  |  |  |  |  |  |  |  |
| **Peptide** | **-10lgP** | **Mass** | **Length** | **ppm** | **m/z** | **z** | **RT** | **Start** | **End** | **PTM** | **Found By** |
| R.GDSGYGSGSSAAAAAAAAAAAR.R | 73.57 | 1809.824 | 22 | 1.7 | 905.9182 | 2 | 60.7 | 2512 | 2533 |  | PEAKS DB |
| R.GDSGYGSGSSAAAAAAAAAAARR.A | 57.3 | 1965.925 | 23 | 3 | 656.3157 | 3 | 59.7 | 2512 | 2534 |  | PEAKS DB |
| R.AAGSAAAAAAAAAAAAASGAGR.S | 57.14 | 1655.834 | 22 | 3.5 | 552.9522 | 3 | 72.7 | 2351 | 2372 |  | PEAKS DB |
| R.AAGSAAAAAAAAAAAAASGAGGSGR.G | 56.28 | 1856.909 | 25 | 4 | 619.9775 | 3 | 71.3 | 908 | 932 |  | PEAKS DB |
| Y.GSGSSAAAAAAAAAAAR.R | 54.68 | 1330.659 | 17 | -1.1 | 666.334 | 2 | 53.3 | 2517 | 2533 |  | PEAKS DB |
| L.HHHDEYVDNHGQLVER.F | 51.96 | 1983.894 | 16 | -1.4 | 496.9785 | 4 | 42.9 | 22 | 37 |  | PEAKS DB |
| R.GD(+21.98)SGYGSGSSAAAAAAAAAAAR.R | 51.01 | 1831.806 | 22 | -1.1 | 611.6068 | 3 | 60.7 | 2512 | 2533 | Sodium adduct | PEAKS PTM |
| A.NNLHHHDEYVDNHGQLVER.F | 50.73 | 2325.064 | 19 | 2 | 776.0277 | 3 | 44.3 | 19 | 37 |  | SPIDER |
| R.STEGHPLLSIC(+57.02)C(+57.02)RPC(+57.02)SHR.H | 50.73 | 2165.988 | 18 | 1.5 | 542.5035 | 4 | 57.6 | 2828 | 2845 | Carbamidomethylation | PEAKS DB |
| D.PYGHEDIYEEDVVINR.V | 50.22 | 1946.901 | 16 | 0.5 | 649.9727 | 3 | 66.3 | 70 | 85 |  | PEAKS DB |
| A.NNP(sub L)HHHDEYVDNHGQLVER.F | 47.69 | 2309.032 | 19 | -0.7 | 578.2632 | 4 | 49.9 | 19 | 37 |  | SPIDER |
| R.AGHDRAAGSAAAAAAAAAAAAASGAGR.S | 47.36 | 2192.079 | 27 | -1.3 | 549.0248 | 4 | 69.5 | 2346 | 2372 |  | PEAKS DB |
| A.N(+.98)NLHHHDEYVDNHGQLVER.F | 47.29 | 2326.048 | 19 | 0.7 | 466.2158 | 5 | 46.5 | 19 | 37 | Deamidation (NQ) | SPIDER |
| A.NN(+.98)LHHHDEYVDNHGQLVER.F | 47.01 | 2326.048 | 19 | 4.9 | 582.5203 | 4 | 46.6 | 19 | 37 | Deamidation (NQ) | SPIDER |
| A.NNLHHHDEYVDN(+.98)HGQLVER.F | 46.85 | 2326.048 | 19 | 0.4 | 776.3546 | 3 | 46 | 19 | 37 | Deamidation (NQ) | SPIDER |
| R.AAGSAAAAAAAAAAAAASGAGGS(-18.01)GR.G | 46.16 | 1838.898 | 25 | -3 | 613.9697 | 3 | 71.3 | 908 | 932 | Dehydration | PEAKS PTM |
| D.SGYGSGSSAAAAAAAAAAAR.R | 46.14 | 1637.776 | 20 | 0.6 | 819.8932 | 2 | 60.1 | 2514 | 2533 |  | PEAKS DB |
| R.AYGAGSAAAAAAAAAAGAGASRPVGIY.G | 45.27 | 2263.135 | 27 | -1.1 | 755.3824 | 3 | 80.1 | 2767 | 2793 |  | PEAKS DB |
| R.S(+79.97)TEGHPLLSICC(+57.02)RPC(+57.02)SHR.H | 44.86 | 2188.933 | 18 | -3.7 | 548.2369 | 4 | 62.4 | 2828 | 2845 | Phosphorylation (STY); Carbamidomethylation | PEAKS PTM |
| R.GGSGFYETHDSY.S | 44.43 | 1318.51 | 12 | 2.7 | 660.2622 | 2 | 54.1 | 135 | 146 |  | PEAKS DB |
| N.NLHHHDEYVDN(+.98)HGQLVER.F | 44.19 | 2212.005 | 18 | 4.9 | 554.0095 | 4 | 45.2 | 20 | 37 | Deamidation (NQ) | SPIDER |
| R.GGSGFYETHDSYSSY.G | 43.99 | 1655.638 | 15 | 1.8 | 828.8251 | 2 | 60.1 | 135 | 149 |  | SPIDER |
| R.AGHDHAAGSSGGGYSWDYSSY.G | 43.82 | 2130.83 | 21 | -0.7 | 711.2815 | 3 | 66.1 | 375 | 395 |  | SPIDER |
| D.IYEEDVVINR.V | 43.55 | 1248.635 | 10 | 1.4 | 625.3239 | 2 | 59.4 | 76 | 85 |  | PEAKS DB |
| Y.GRGDSGYGSGSSAAAAAAAAAAAR.R | 42.6 | 2022.947 | 24 | -1.3 | 675.32 | 3 | 59 | 2510 | 2533 |  | PEAKS DB |
| R.S(+79.97)TEGHPLLSIC(+57.02)CRPC(+57.02)SHR.H | 42.41 | 2188.933 | 18 | -3.7 | 548.2369 | 4 | 62.4 | 2828 | 2845 | Phosphorylation (STY); Carbamidomethylation | PEAKS PTM |
| R.GD(-18.01)SGYGSGSSAAAAAAAAAAARR.A | 42.29 | 1947.915 | 23 | 0.9 | 650.3109 | 3 | 59.6 | 2512 | 2534 | Dehydration | PEAKS PTM |
| T.ANNA(sub L)HHHDEYVDNHGQLVER.F | 41.89 | 2354.054 | 20 | -3.1 | 589.5172 | 4 | 50.4 | 18 | 37 |  | SPIDER |
| A.NNR(sub L)HHHDEYVDNHGQLVER.F | 41.1 | 2368.081 | 19 | -4.1 | 593.0233 | 4 | 47.5 | 19 | 37 |  | SPIDER |
| S.GSSAAAAAAAAAAARR.A | 40.23 | 1342.707 | 16 | -0.7 | 672.3582 | 2 | 59.6 | 2519 | 2534 |  | PEAKS DB |
| D.SGYGSGSSAAAAAAAAAAARR.A | 39.76 | 1793.877 | 21 | 1.4 | 598.9653 | 3 | 59 | 2514 | 2534 |  | PEAKS DB |
| N.N(+.98)LHHHDEYVDNHGQLVER.F | 39.74 | 2212.005 | 18 | -0.1 | 554.0068 | 4 | 48 | 20 | 37 | Deamidation (NQ) | SPIDER |
| R.AGHDHAAGSSGGGYSW.D | 39.39 | 1515.613 | 16 | -1.2 | 506.2094 | 3 | 51.7 | 375 | 390 |  | PEAKS DB |
| A.N(+.98)N(+.98)LHHHDEYVDNHGQLVER.F | 38.89 | 2327.032 | 19 | -3.1 | 466.4108 | 5 | 49.4 | 19 | 37 | Deamidation (NQ) | SPIDER |
| N.LHHHDEYVDNHGQLVER.F | 38.36 | 2096.978 | 17 | 0.8 | 420.4019 | 5 | 43.9 | 21 | 37 |  | SPIDER |
| A.N(+.98)H(sub N)LHHHDEYVDNHGQLVER.F | 38.26 | 2349.064 | 19 | -1.2 | 470.818 | 5 | 49.8 | 19 | 37 | Deamidation (NQ) | SPIDER |
| R.GDSGYGSGSSAAAAAAAAAAAR(+54.01)R.A | 37.74 | 2019.936 | 23 | 1.8 | 674.3185 | 3 | 61.6 | 2512 | 2534 | Methylglyoxal-derived hydroimidazolone | PEAKS PTM |
| A.N(+.98)NLHHHDEYVDN(+.98)HGQLVER.F | 37.61 | 2327.032 | 19 | 3.9 | 776.6852 | 3 | 48 | 19 | 37 | Deamidation (NQ) | SPIDER |
| A.NN(+.98)LHHHDEYVDNHGQ(+.98)LVER.F | 37.61 | 2327.032 | 19 | 3.6 | 466.4139 | 5 | 48 | 19 | 37 | Deamidation (NQ) | SPIDER |
| R.QAGHERAAGSAAAAAAAAAAAAASGAGGSGR.G | 37.6 | 2535.229 | 31 | 2.8 | 634.8143 | 4 | 68.3 | 902 | 932 |  | PEAKS DB |
| Y.ATANNC(sub L)HHHDEYVDNHGQLVER.F | 37.58 | 2558.111 | 22 | -4 | 512.6259 | 5 | 47.1 | 16 | 37 |  | SPIDER |
| A.NN(+.98)R(sub L)HHHDEYVDNHGQLVER.F | 37.51 | 2369.065 | 19 | -2.2 | 593.2704 | 4 | 48.9 | 19 | 37 | Deamidation (NQ) | SPIDER |
| N.N(+.98)LHHHDEYVDN(+.98)HGQLVER.F | 37.28 | 2212.989 | 18 | 1 | 554.2534 | 4 | 49.5 | 20 | 37 | Deamidation (NQ) | SPIDER |
| N.LHHHDEYVDN(+.98)HGQLVER.F | 37.03 | 2097.962 | 17 | 3.8 | 525.4982 | 4 | 45.9 | 21 | 37 | Deamidation (NQ) | SPIDER |
| A.H(sub N)N(+.98)LHHHDEYVDNHGQLVER.F | 36.96 | 2349.064 | 19 | 0.7 | 588.2719 | 4 | 49.8 | 19 | 37 | Deamidation (NQ) | SPIDER |
| A.H(sub N)NM(sub L)HHHDEYVDNHGQLVER.F | 36.76 | 2366.036 | 19 | 3.9 | 592.5168 | 4 | 51.4 | 19 | 37 |  | SPIDER |
| R.GD(+21.98)S(+79.97)GYGSGSSAAAAAAAAAAAR.R | 36.72 | 1911.772 | 22 | -2.3 | 638.2614 | 3 | 65.5 | 2512 | 2533 | Sodium adduct; Phosphorylation (STY) | PEAKS PTM |
| R.STEGHPLLSIC(+57.02)CRPC(+57.02)SHR.H | 36.39 | 2108.967 | 18 | 0.7 | 528.2477 | 4 | 62.4 | 2828 | 2845 | Carbamidomethylation | PEAKS PTM |
| A.N(+.98)NLHHHDEYVDNHGQLVERF.T | 36.22 | 2473.116 | 20 | 0.6 | 619.2849 | 4 | 61 | 19 | 38 | Deamidation (NQ) | SPIDER |
| R.GD(+37.95)SGYGSGSSAAAAAAAAAAAR.R | 35.96 | 1847.771 | 22 | -1.1 | 462.9482 | 4 | 60.8 | 2512 | 2533 | Replacement of 2 protons by calcium | PEAKS PTM |
| R.GDGGYGSGSSAAAAAAAAAAAAARQAGHER.A | 35.82 | 2600.208 | 30 | -2.3 | 651.0558 | 4 | 73.4 | 878 | 907 |  | PEAKS DB |
| D.SAAAAAAAAAAAAAGSGAGR.A | 35.81 | 1513.76 | 20 | -0.7 | 505.592 | 3 | 63.8 | 489 | 508 |  | PEAKS DB |
| R.AAGSAAAAAAAAAAAAAS(-18.01)GAGR.S | 35.76 | 1637.823 | 22 | 2.3 | 546.948 | 3 | 72.5 | 2351 | 2372 | Dehydration | PEAKS PTM |
| A.NN(+.98)LHHHDEYVDNHGQLVERF.T | 35.6 | 2473.116 | 20 | -1.8 | 619.2833 | 4 | 62.9 | 19 | 38 | Deamidation (NQ) | SPIDER |
| T.AN(+.98)NLHHHDEYVDNHGQLVER.F | 35.6 | 2397.085 | 20 | -3 | 600.2749 | 4 | 48.2 | 18 | 37 | Deamidation (NQ) | SPIDER |
| N.NA(sub L)HHHDEYVDNHGQLVER.F | 35.42 | 2168.974 | 18 | -2.4 | 543.2478 | 4 | 53.3 | 20 | 37 |  | SPIDER |
| A.NNA(sub L)HHHDEYVDNHGQLVER.F | 35.41 | 2283.017 | 19 | -3.2 | 762.0082 | 3 | 51.6 | 19 | 37 |  | SPIDER |
| A.TANNM(sub L)HHHDEYVDNHGQLVER.F | 35.2 | 2515.105 | 21 | -4.5 | 629.7788 | 4 | 49.7 | 17 | 37 |  | SPIDER |
| A.NNV(sub L)Y(sub H)HHDEYVDNHGQLVER.F | 35.2 | 2337.052 | 19 | 4 | 780.0255 | 3 | 46.3 | 19 | 37 |  | SPIDER |
| N.NLHHHDEYVDNHGQ(-18.01).L | 35.02 | 1695.714 | 14 | -1.3 | 566.2429 | 3 | 44.4 | 20 | 33 | Dehydration | PEAKS PTM |
| A.NNW(sub L)HHHDEYVDNHGQLVER.F | 34.82 | 2398.059 | 19 | -8.6 | 600.5151 | 4 | 52.3 | 19 | 37 |  | SPIDER |
| A.N(+.98)NLHHHDEYVDN(+.98)HGQLVERF.T | 34.57 | 2474.1 | 20 | 1.7 | 619.5315 | 4 | 62.5 | 19 | 38 | Deamidation (NQ) | SPIDER |
| N.NH(sub L)HHHDEYVDNHGQLVER.F | 34.52 | 2234.995 | 18 | 9.4 | 559.7598 | 4 | 47.3 | 20 | 37 |  | SPIDER |
| N.NW(sub L)HHHDEYVDNHGQLVER.F | 34.51 | 2284.016 | 18 | -7.2 | 572.0055 | 4 | 53.1 | 20 | 37 |  | SPIDER |
| A.Q(sub N)NLY(sub H)HHDEYVDNHGQLVER.F | 33.93 | 2365.084 | 19 | -8.7 | 592.2713 | 4 | 49.2 | 19 | 37 |  | SPIDER |
| A.N(+.98)N(+.98)LHHHDEYVDN(+.98)HGQLVER.F | 33.6 | 2328.016 | 19 | -2.1 | 466.6081 | 5 | 50.3 | 19 | 37 | Deamidation (NQ) | SPIDER |
| L.HHHDEY(+79.97)VDN(+203.08)HGQLVER.F | 33.37 | 2266.939 | 16 | 8.9 | 567.7455 | 4 | 56.5 | 22 | 37 | Phosphorylation (STY); HexNAcylation (N) | PEAKS PTM |
| Y.GHEDIYEEDVVINR.V | 33.08 | 1686.785 | 14 | -1 | 563.2667 | 3 | 63.3 | 72 | 85 |  | PEAKS DB |
| A.NNC(sub L)HHHDEYVDNHGQLVER.F | 32.74 | 2314.989 | 19 | 0.6 | 579.7531 | 4 | 56.3 | 19 | 37 |  | SPIDER |
| R.GGSGFYETHDSY(-.98).S | 32.67 | 1317.526 | 12 | 1.9 | 659.7697 | 2 | 51.1 | 135 | 146 | Amidation | PEAKS PTM |
| D.SAAGAAAAAAAAAASGAGGR.G | 32.56 | 1499.744 | 20 | 0.5 | 750.8774 | 2 | 63.9 | 1990 | 2009 |  | PEAKS DB |
| L.HHHDEYVDN(+228.11)HGQLVER.F | 32.46 | 2212.005 | 16 | -0.4 | 443.4067 | 5 | 46.1 | 22 | 37 | 2,4-diacetamido-2,4,6-trideoxyglucopyranose | PEAKS PTM |
| R.GGSGFYETHDSYS.S | 32.42 | 1405.542 | 13 | -2.6 | 703.7745 | 2 | 51.4 | 135 | 147 |  | SPIDER |
| R.AAGSAAAAAAAAAAAAASGAGGSGR(+21.98).G | 31.84 | 1878.891 | 25 | 0.2 | 627.3025 | 3 | 71.3 | 908 | 932 | Sodium adduct | PEAKS PTM |
| R.GGS(-18.01)GFYETHDSY.S | 31.65 | 1300.5 | 12 | -1.9 | 651.254 | 2 | 54.1 | 135 | 146 | Dehydration | PEAKS PTM |
| A.NN(+.98)W(sub L)HHHDEYVDNHGQLVER.F | 31.39 | 2399.043 | 19 | -2.9 | 600.7645 | 4 | 51.7 | 19 | 37 | Deamidation (NQ) | SPIDER |
| Y.GSGS(-2.02)SAAAAAAAAAAAR.R | 31.24 | 1328.643 | 17 | 3.1 | 665.329 | 2 | 53.5 | 2517 | 2533 | 2-amino-3-oxo-butanoic_acid | PEAKS PTM |
| A.N(+.98)NW(sub L)HHHDEYVDNHGQLVER.F | 31.23 | 2399.043 | 19 | -2.9 | 600.7645 | 4 | 51.7 | 19 | 37 | Deamidation (NQ) | SPIDER |
| L.H(+226.08)(+57.02)HHDEYVDNHGQLVER.F | 31.06 | 2266.993 | 16 | 3.2 | 567.7556 | 4 | 56 | 22 | 37 | Biotinylation; Carbamidomethylation (DHKE, X@N-term) | PEAKS PTM |
| A.NNM(sub L)HHHH(sub D)EYVDNHGQLVER.F | 30.59 | 2365.052 | 19 | 1.8 | 474.0172 | 5 | 50.2 | 19 | 37 |  | SPIDER |
| R.AGGDYGWGDGGY.G | 30.57 | 1173.436 | 12 | -1.6 | 587.7228 | 2 | 63.1 | 509 | 520 |  | PEAKS DB |
| A.H(sub N)NC(sub L)HHHDEYVDNHGQLVER.F | 30.54 | 2338.005 | 19 | 8.1 | 585.5115 | 4 | 53 | 19 | 37 |  | SPIDER |
| A.N(+.98)NLY(sub H)HHDEYVDNHGQLVER.F | 30.28 | 2352.052 | 19 | -3.9 | 589.0163 | 4 | 48.4 | 19 | 37 | Deamidation (NQ) | SPIDER |
| R.GGSGFYETHDS.Y | 30.25 | 1155.447 | 11 | -0.3 | 578.7289 | 2 | 43.5 | 135 | 145 |  | PEAKS DB |
| T.D(sub A)NNLHHHDEYVDNHGQLVER.F | 30.19 | 2440.09 | 20 | -1.8 | 611.027 | 4 | 52.7 | 18 | 37 |  | SPIDER |
| D.GGYGSDSAAAAAAAAAAAAAGSGAGR.A | 30.15 | 2049.946 | 26 | 1 | 684.3214 | 3 | 73.6 | 483 | 508 |  | PEAKS DB |
| A.N(+.98)NH(sub L)HHHDEYVDNHGQLVER.F | 30.13 | 2350.023 | 19 | -5.6 | 588.5079 | 4 | 46.4 | 19 | 37 | Deamidation (NQ) | SPIDER |
| N.V(sub L)Y(sub H)HHDEYVDNHGQLVER.F | 29.99 | 2108.966 | 17 | 5.1 | 528.25 | 4 | 47.4 | 21 | 37 |  | SPIDER |
| R.STEGHPLLSIC(+57.02)C(+57.02)F(sub R)PC(+57.02)SHR.H | 29.63 | 2156.955 | 18 | -0.7 | 719.9898 | 3 | 60 | 2828 | 2845 | Carbamidomethylation | SPIDER |
| R.STEGHPLLSIC(+57.02)C(+15.99)E(sub R)PCSHR.H | 29.46 | 2040.882 | 18 | 6 | 511.2292 | 4 | 68.1 | 2828 | 2845 | Carbamidomethylation; Oxidation or Hydroxylation (C) | SPIDER |
| S.GYGSGSS(+150.04)AAAAAAAAAAAR.R | 29.43 | 1700.785 | 19 | -3.8 | 567.9319 | 3 | 64.9 | 2515 | 2533 | S-guanylation-2 | PEAKS PTM |
| N.LHHHDEYVDNHGQ(-18.01).L | 29.19 | 1581.671 | 13 | 0.1 | 528.2294 | 3 | 43.9 | 21 | 33 | Dehydration | PEAKS PTM |
| R.GGSGFYETHDS(-18.01)Y.S | 29.13 | 1300.5 | 12 | 0.8 | 651.2557 | 2 | 58.3 | 135 | 146 | Dehydration | PEAKS PTM |
| A.N(+.98)N(+.98)LF(sub H)HHDEYVDNHGQLVER.F | 29.12 | 2337.041 | 19 | 10.5 | 585.272 | 4 | 46.3 | 19 | 37 | Deamidation (NQ) | SPIDER |
| R.AAGSAAAAAAAAAAAAASGAGR(+21.98).S | 28.73 | 1677.816 | 22 | 0.8 | 560.278 | 3 | 72.7 | 2351 | 2372 | Sodium adduct | PEAKS PTM |
| W.GDGGYGSDSAAGAAAAAAAAAASGAGGRG.D | 28.61 | 2265.001 | 29 | 1.4 | 756.0063 | 3 | 69.9 | 1982 | 2010 |  | PEAKS DB |
| R.GGSGFYETHD.S | 28.56 | 1068.415 | 10 | -0.2 | 535.213 | 2 | 44.4 | 135 | 144 |  | PEAKS DB |
| L.H(+226.08)HHDEYVDN(+.98)HGQLVER.F | 28.52 | 2210.955 | 16 | 6.8 | 553.7482 | 4 | 54.2 | 22 | 37 | Biotinylation; Deamidation (NQ) | PEAKS PTM |
| G.GYGSDSAAAAAAAAAAAAGSGAGGR.G | 28.44 | 1978.909 | 25 | 1.1 | 660.6425 | 3 | 69 | 176 | 200 |  | SPIDER |
| T.AN(+.98)NA(sub L)HHHDEYVDNHGQLVER.F | 28.31 | 2355.038 | 20 | -0.6 | 589.7646 | 4 | 52 | 18 | 37 | Deamidation (NQ) | SPIDER |
| A.G(sub N)NLHHHDEYVDNHGQLVER.F | 28.23 | 2268.042 | 19 | 0 | 568.0161 | 4 | 44.2 | 19 | 37 |  | SPIDER |
| A.N(+.98)NLHHHDEYVDP(sub N)HGQLVER.F | 27.46 | 2309.057 | 19 | -8.5 | 578.265 | 4 | 47 | 19 | 37 | Deamidation (NQ) | SPIDER |
| R.GGSGFYETH.D | 27.14 | 953.3879 | 9 | 0.3 | 477.7 | 2 | 43 | 135 | 143 |  | PEAKS DB |
| R.STEGHPLLQ(sub S)ICCRPCQ(sub S)HR.H | 26.79 | 2076.977 | 18 | 9.4 | 520.2548 | 4 | 57.1 | 2828 | 2845 |  | SPIDER |
| A.NNA(sub L)HHHDEYVDN(+.98)HGQLVER.F | 26.39 | 2284.001 | 19 | 1.1 | 572.0063 | 4 | 52.4 | 19 | 37 | Deamidation (NQ) | SPIDER |
| R.AGGDYGWGDGGY(-.98).G | 26.11 | 1172.452 | 12 | -1 | 587.2311 | 2 | 61.3 | 509 | 520 | Amidation | PEAKS PTM |
| A.A(sub N)NLHHHDEYVDNHGQLVER.F | 25.91 | 2282.058 | 19 | 1.9 | 571.5211 | 4 | 44.9 | 19 | 37 |  | SPIDER |
| A.NNLHHHDEYVD(-18.01)NHGQL.V | 25.6 | 1922.841 | 16 | -0.6 | 641.952 | 3 | 44.2 | 19 | 34 | Dehydration | PEAKS PTM |
| N.NV(sub L)Y(sub H)HHDEYVDNHGQLVER.F | 25.6 | 2223.009 | 18 | 5.4 | 556.761 | 4 | 46.3 | 20 | 37 |  | SPIDER |
| R.AGHDHAAGSSGGGYSWD.Y | 25.21 | 1630.64 | 17 | -1.9 | 544.5512 | 3 | 49 | 375 | 391 |  | PEAKS DB |
| A.N(+.98)T(sub N)F(sub L)HHHDEYVDNHGQLVER.F | 25.09 | 2347.037 | 19 | 6.3 | 587.7684 | 4 | 54.4 | 19 | 37 | Deamidation (NQ) | SPIDER |
| A.NNH(sub L)HHHDEYVDN(+.98)HGQLVER.F | 25 | 2350.023 | 19 | 9.6 | 471.0149 | 5 | 51.9 | 19 | 37 | Deamidation (NQ) | SPIDER |
| N.NM(sub L)F(sub H)HHDEYVDNHGQLVER.F | 24.7 | 2238.987 | 18 | 8.3 | 560.757 | 4 | 53.3 | 20 | 37 |  | SPIDER |
| A.N(+.98)NLHHHDEYVD.N | 24.65 | 1392.57 | 11 | -0.6 | 465.1955 | 3 | 38.8 | 19 | 29 | Deamidation (NQ) | PEAKS DB |
| N.Q(sub N)Q(sub L)HHHDEYVDNHGQLVER.F | 24.48 | 2240.011 | 18 | -4.1 | 561.006 | 4 | 53.2 | 20 | 37 |  | SPIDER |
| A.NNM(sub L)Q(sub H)HHDEYVDNHGQLVER.F | 24.15 | 2334.02 | 19 | -0.8 | 584.51 | 4 | 56 | 19 | 37 |  | SPIDER |
| A.N(+.98)NLF(sub H)HHDEYVDN(+.98)HGQLVER.F | 23.69 | 2337.041 | 19 | -4.8 | 585.263 | 4 | 52.2 | 19 | 37 | Deamidation (NQ) | SPIDER |

Table S8. List of unique peptides detected from *A. assamensis* digested with trypsin, FA-trypsin, chymotrypsin, and chymotrypsin-trypsin at False Discovery Rate ≤1%

| **TRYPSIN** |  |  |  |  |  |  |  |  |  |  |  |
| --- | --- | --- | --- | --- | --- | --- | --- | --- | --- | --- | --- |
| **Peptide** | **-10lgP** | **Mass** | **Length** | **ppm** | **m/z** | **z** | **RT** | **Start** | **End** | **PTM** | **Found By** |
| R.HHDEYVDSN(+.98)GQLFER.F | 66.51 | 1845.792 | 15 | -2.7 | 616.2695 | 3 | 44.3 | 23 | 37 | Deamidation (NQ) | PEAKS DB |
| R.NAETRPNLSGNER.L | 64.48 | 1456.702 | 13 | -2.9 | 729.3561 | 2 | 34.3 | 47 | 59 |  | PEAKS DB |
| K.RVPGASSSAAAASSASAGGR.G | 64.23 | 1716.85 | 20 | -2.4 | 573.2893 | 3 | 34.6 | 85 | 104 |  | PEAKS DB |
| R.HHDEYVDSNGQLFER.F | 63.47 | 1844.808 | 15 | -2.8 | 615.9415 | 3 | 42.4 | 23 | 37 |  | PEAKS DB |
| Y.VDSNGQLFER.F | 62.7 | 1163.557 | 10 | -2.5 | 582.7844 | 2 | 44.3 | 28 | 37 |  | PEAKS DB |
| D.SNGQLFER.F | 59.9 | 949.4617 | 8 | -2.1 | 475.7372 | 2 | 40.6 | 30 | 37 |  | PEAKS DB |
| R.NAETRPNLSGNE.R | 58.72 | 1300.601 | 12 | -2.4 | 651.3061 | 2 | 36.4 | 47 | 58 |  | PEAKS DB |
| R.NAETRPN(+.98)LSGNER.L | 57.07 | 1457.686 | 13 | -2.1 | 729.8486 | 2 | 37.3 | 47 | 59 | Deamidation (NQ) | PEAKS DB |
| Y.VDSN(+.98)GQLFER.F | 56.93 | 1164.541 | 10 | -2.2 | 583.2766 | 2 | 46.8 | 28 | 37 | Deamidation (NQ) | PEAKS DB |
| R.N(+.98)AETRPNLSGNER.L | 56.58 | 1457.686 | 13 | -1.7 | 729.8489 | 2 | 35.3 | 47 | 59 | Deamidation (NQ) | PEAKS DB |
| D.SN(+.98)GQLFER.F | 56.36 | 950.4457 | 8 | -2.9 | 476.2288 | 2 | 42.8 | 30 | 37 | Deamidation (NQ) | PEAKS DB |
| R.N(+.98)AETRPN(+.98)LSGNER.L | 55.91 | 1458.67 | 13 | -2.6 | 487.2293 | 3 | 36.3 | 47 | 59 | Deamidation (NQ) | PEAKS DB |
| R.NAETP(sub R)PNLSGNER.L | 55.57 | 1397.653 | 13 | -2.4 | 699.8323 | 2 | 41.3 | 47 | 59 |  | SPIDER |
| R.NAETRPNLSGN(+.98)ER.L | 55.47 | 1457.686 | 13 | -2.9 | 729.848 | 2 | 34.6 | 47 | 59 | Deamidation (NQ) | PEAKS DB |
| E.TRPNLSGNER.L | 53.83 | 1142.579 | 10 | -2.4 | 572.2955 | 2 | 33.7 | 50 | 59 |  | PEAKS DB |
| R.LVETIVLEEDPYGHEN(+203.08).I | 50.31 | 2058.963 | 16 | -14 | 1030.475 | 2 | 66.8 | 60 | 75 | HexNAcylation (N) | PEAKS PTM |
| R.AAGSAAAAAAAAAAAAAGSGSGGY(+15.99)GGGY(+15.99)G(+15.99).W | 49.16 | 2231.968 | 29 | -7.9 | 744.9907 | 3 | 58.6 | 329 | 357 | Oxidation or Hydroxylation | PEAKS DB |
| R.NAETRPNLSGN.E | 48.72 | 1171.558 | 11 | -2.1 | 586.7851 | 2 | 35.6 | 47 | 57 |  | PEAKS DB |
| R.AAGSAAAAAAAAAAAAAGSGAGGHGG.G | 48.64 | 1864.878 | 26 | -2.7 | 622.6315 | 3 | 52.7 | 205 | 230 |  | PEAKS DB |
| R.N(+27.99)AETRPNLSGNER.L | 48.41 | 1484.697 | 13 | -2.3 | 743.3539 | 2 | 38 | 47 | 59 | Formylation | PEAKS PTM |
| R.NAETRPNLSG.N | 47.64 | 1057.515 | 10 | -2.1 | 529.7638 | 2 | 35.6 | 47 | 56 |  | PEAKS DB |
| D.SN(+.98)GQ(+.98)LFER.F | 47.61 | 951.4297 | 8 | -2.5 | 476.7209 | 2 | 46.2 | 30 | 37 | Deamidation (NQ) | PEAKS DB |
| V.DSN(+.98)GQLFER.F | 47.07 | 1065.473 | 9 | -2.1 | 533.7425 | 2 | 47.3 | 29 | 37 | Deamidation (NQ) | PEAKS DB |
| R.NAETRPNLSGNERLVE.T | 47.06 | 1797.897 | 16 | -2.9 | 600.3045 | 3 | 42.3 | 47 | 62 |  | PEAKS DB |
| R.NAETRPNLSGN(+.98).E | 46.04 | 1172.542 | 11 | -2.2 | 587.277 | 2 | 36.5 | 47 | 57 | Deamidation (NQ) | PEAKS DB |
| K.RVPGASSSAAAASSASAGGRGGF.Y | 46.03 | 1977.962 | 23 | -2.4 | 660.3262 | 3 | 39.3 | 85 | 107 |  | PEAKS DB |
| Y.V(+43.01)DSNGQLFER.F | 46.01 | 1206.563 | 10 | -1.5 | 604.2878 | 2 | 56.7 | 28 | 37 | Carbamylation | PEAKS PTM |
| N.AETRPNLSGNER.L | 45.99 | 1342.659 | 12 | -1.5 | 448.5596 | 3 | 34.2 | 48 | 59 |  | PEAKS DB |
| R.AAGSAAAAAAAAAAAAGGSGAGGHGGG.Y | 44.45 | 1907.883 | 27 | -1.5 | 636.9674 | 3 | 48.8 | 771 | 797 |  | PEAKS DB |
| R.HHD(+15.99)E(+43.99)YVDSNGQLFER.F | 43.19 | 1904.793 | 15 | 3.6 | 635.9404 | 3 | 44.3 | 23 | 37 | Oxidation or Hydroxylation; Carboxylation (E) | PEAKS PTM |
| N.AETRPN(+.98)LSGNER.L | 42.32 | 1343.643 | 12 | -2.8 | 448.887 | 3 | 34.5 | 48 | 59 | Deamidation (NQ) | PEAKS DB |
| D.SN(+.98)GQLFERF.T | 42.11 | 1097.514 | 9 | -2.1 | 549.7632 | 2 | 57.6 | 30 | 38 | Deamidation (NQ) | PEAKS DB |
| D.SNGQLFERF.T | 41.8 | 1096.53 | 9 | -2.1 | 549.2712 | 2 | 52.3 | 30 | 38 |  | PEAKS DB |
| R.NAETRPNL.S | 41.77 | 913.4617 | 8 | -2.1 | 457.7372 | 2 | 36.4 | 47 | 54 |  | PEAKS DB |
| R.AAGSAAAAAAAAAAAAAGSGAGGHGGG.Y | 41.55 | 1921.899 | 27 | -2.2 | 641.6389 | 3 | 52.5 | 205 | 231 |  | PEAKS DB |
| Y.VDSN(-17.03)GQLFER.F | 40.93 | 1146.531 | 10 | -2.8 | 574.2709 | 2 | 46.5 | 28 | 37 | Ammonia-loss (N) | PEAKS PTM |
| R.NAEG(sub T)RPNLSGNER.L | 40.73 | 1412.676 | 13 | -9.9 | 707.3381 | 2 | 40.3 | 47 | 59 |  | SPIDER |
| R.AAGSAAAAAAAAAAAAGSGAGGSGYG.Y | 40.68 | 1906.877 | 26 | 9.3 | 636.6388 | 3 | 47.3 | 2561 | 2586 |  | PEAKS DB |
| R.LVETIVLEEDPY(+44.99)GHENI.Y | 39.97 | 2013.953 | 17 | -2.4 | 1007.981 | 2 | 65.8 | 60 | 76 | Oxidation to nitro | PEAKS PTM |
| E.RN(+.98)AETRPNLSGNER.L | 39.44 | 1613.787 | 14 | -3.2 | 538.9346 | 3 | 34.8 | 46 | 59 | Deamidation (NQ) | PEAKS DB |
| R.N(+.98)AETRPNLSGNERLVETIVLEED.P | 39.39 | 2598.277 | 23 | -3.2 | 867.097 | 3 | 62.3 | 47 | 69 | Deamidation (NQ) | SPIDER |
| D.SN(+.98)GQLFERFTTR.K | 39.09 | 1455.711 | 12 | -2 | 728.8611 | 2 | 55.7 | 30 | 41 | Deamidation (NQ) | PEAKS DB |
| R.PNLSGNERLVETIVLEED.P | 39.01 | 2026.022 | 18 | -1.5 | 1014.017 | 2 | 63.9 | 52 | 69 |  | PEAKS DB |
| N.GQLFERFTTR.K | 38.06 | 1253.652 | 10 | -2.8 | 627.8313 | 2 | 46.6 | 32 | 41 |  | PEAKS DB |
| R.NAE(+21.98)TR(+14.02)PNLSGNER.L | 38.03 | 1492.7 | 13 | -0.9 | 747.3563 | 2 | 44.6 | 47 | 59 | Sodium adduct; Methylation(KR) | PEAKS PTM |
| Y.R(sub V)DSNGQLFER.F | 37.42 | 1220.59 | 10 | -11 | 611.2952 | 2 | 46.5 | 28 | 37 |  | SPIDER |
| R.NAETRPNLS.G | 37.12 | 1000.494 | 9 | -2.7 | 501.2528 | 2 | 35.2 | 47 | 55 |  | PEAKS DB |
| R.LVETIVLEEDPYT(sub G)HENI.Y | 36.2 | 2012.994 | 17 | -13 | 1007.491 | 2 | 63.9 | 60 | 76 |  | SPIDER |
| R.HHDEY(+44.99)VDSNGQLFER.F | 34.93 | 1889.793 | 15 | -2.2 | 630.9368 | 3 | 45 | 23 | 37 | Oxidation to nitro | PEAKS PTM |
| R.NAETRPNLSGNERLVET.I | 34.78 | 1898.945 | 17 | -2.2 | 633.9874 | 3 | 42.2 | 47 | 63 |  | PEAKS DB |
| E.TRPN(+.98)LSGNER.L | 34.78 | 1143.563 | 10 | -1.8 | 572.7878 | 2 | 36 | 50 | 59 | Deamidation (NQ) | PEAKS DB |
| R.HHDEYVDSN(+15.99)GQLFER.F | 34.58 | 1860.803 | 15 | -1.7 | 621.2737 | 3 | 41.8 | 23 | 37 | Oxidation or Hydroxylation | PEAKS DB |
| D.SNGQLFERFT.T | 34.42 | 1197.578 | 10 | -0.9 | 599.7956 | 2 | 53.1 | 30 | 39 |  | PEAKS DB |
| N.GQLFER.F | 34.18 | 748.3868 | 6 | -3.1 | 375.1995 | 2 | 40.3 | 32 | 37 |  | PEAKS DB |
| R.NAETRPNLSGN(-18.01).E | 33.86 | 1153.548 | 11 | -2.1 | 577.7798 | 2 | 35 | 47 | 57 | Dehydration | PEAKS PTM |
| A.ET(-18.01)RPNLSGNER.L | 33.36 | 1253.611 | 11 | -1.7 | 627.8118 | 2 | 37 | 49 | 59 | Dehydration | PEAKS PTM |
| N.GQLFERFT.T | 33.33 | 996.5029 | 8 | -2.8 | 499.2573 | 2 | 52.4 | 32 | 39 |  | PEAKS DB |
| A.ETRPN(+.98)LSGNER.L | 32.71 | 1272.606 | 11 | -2.6 | 637.3085 | 2 | 37.7 | 49 | 59 | Deamidation (NQ) | SPIDER |
| R.NAETRPN(-17.03)LSGNER.L | 31.1 | 1439.675 | 13 | -2.7 | 480.8977 | 3 | 34.9 | 47 | 59 | Ammonia-loss (N) | PEAKS PTM |
| D.SNGQLFERFTTR.K | 31.1 | 1454.727 | 12 | -2.5 | 728.3688 | 2 | 47.5 | 30 | 41 |  | PEAKS DB |
| R.NAETRPNLSGN(+.98)ERLVETIVLEED.P | 29.32 | 2598.277 | 23 | -2.2 | 867.0978 | 3 | 62.6 | 47 | 69 | Deamidation (NQ) | PEAKS DB |
| R.NAETRPNLSGNERLVETIVLEED.P | 29.18 | 2597.293 | 23 | -3.7 | 866.7685 | 3 | 58.9 | 47 | 69 |  | PEAKS DB |
| D.SN(-17.03)GQLFER.F | 28.94 | 932.4352 | 8 | -2.1 | 467.2239 | 2 | 42.2 | 30 | 37 | Ammonia-loss (N) | PEAKS PTM |
| Y.R(sub V)DSN(+.98)GQLFER.F | 28.56 | 1221.574 | 10 | -12 | 611.787 | 2 | 47.5 | 28 | 37 | Deamidation (NQ) | SPIDER |
| R.NAETRPN(+.98)LSGNERLVETIVLEED.P | 28.53 | 2598.277 | 23 | -3 | 867.0972 | 3 | 61.8 | 47 | 69 | Deamidation (NQ) | PEAKS DB |
| G.Q(-17.03)LFERFTTR.K | 27.96 | 1179.604 | 9 | -3.2 | 590.8072 | 2 | 57.5 | 33 | 41 | Pyro-glu from Q | PEAKS PTM |
| V.DSNGQLFER.F | 27.9 | 1064.489 | 9 | -2 | 533.2505 | 2 | 47.9 | 29 | 37 |  | PEAKS DB |
| Y.E(sub V)DE(sub S)NGQLFER.F | 27.71 | 1235.542 | 10 | -2.2 | 618.7768 | 2 | 66.2 | 28 | 37 |  | SPIDER |
| D.RAAGSAAAAAAAAAAAAAGSGAGGHGGG.Y | 27.6 | 2078 | 28 | -2 | 693.6725 | 3 | 47.3 | 204 | 231 |  | PEAKS DB |
| N.GQ(+.98)LFER.F | 27.3 | 749.3708 | 6 | -2.2 | 750.3764 | 1 | 53.5 | 32 | 37 | Deamidation (NQ) | PEAKS DB |
| N.AETRPNLSGN(+.98).E | 27.05 | 1058.499 | 10 | -3.5 | 530.2551 | 2 | 36.3 | 48 | 57 | Deamidation (NQ) | PEAKS DB |
| F.E(-18.01)RNAETRPNLSGNER.L | 27.05 | 1723.835 | 15 | -2.2 | 575.6177 | 3 | 35 | 45 | 59 | Pyro-glu from E | PEAKS PTM |
| H.HD(+212.01)EYVDSNGQLFER.F | 26.38 | 1919.757 | 14 | 3 | 640.9283 | 3 | 44.5 | 24 | 37 | phosphate-ribosylation | PEAKS PTM |
| D.VVIKRVPGASSSAAAASSASAGGR.G | 26.08 | 2156.166 | 24 | -2.3 | 540.0475 | 4 | 36.6 | 81 | 104 |  | PEAKS DB |
| H.FERNAETRPNLSGNER.L | 25.66 | 1888.914 | 16 | -2.4 | 473.2346 | 4 | 34.9 | 44 | 59 |  | PEAKS DB |
| A.ETR(+54.01)PNLSGNER.L | 25.13 | 1325.632 | 11 | -1.6 | 663.8224 | 2 | 39 | 49 | 59 | Methylglyoxal-derived hydroimidazolone | PEAKS PTM |
| Y.V(+42.01)D(+15.99)SNGQLFER.F | 24.53 | 1221.563 | 10 | -2.5 | 611.787 | 2 | 48.1 | 28 | 37 | Acetylation (N-term); Oxidation or Hydroxylation | PEAKS PTM |
| R.NAETRPNLSGNE(+14.02).R | 24.38 | 1314.616 | 12 | -1.2 | 658.3146 | 2 | 38.2 | 47 | 58 | Methylation(others) | PEAKS PTM |
| N.GQLFERF.T | 23.69 | 895.4551 | 7 | -3.3 | 448.7334 | 2 | 52.1 | 32 | 38 |  | PEAKS DB |
| R.NAETRPNLSGNERLT(sub V)ETIVLEED.P | 23.04 | 2599.273 | 23 | -0.9 | 867.4307 | 3 | 65.2 | 47 | 69 |  | SPIDER |
| R.NAETRPNLSGNERL.V | 22.72 | 1569.786 | 14 | -2.9 | 524.2677 | 3 | 38.8 | 47 | 60 |  | PEAKS DB |
| N.GQ(+.98)LFERFTTR.K | 22.56 | 1254.636 | 10 | -2.3 | 628.3237 | 2 | 57.7 | 32 | 41 | Deamidation (NQ) | PEAKS DB |
| Y.M(sub V)DE(sub S)NGQLFER.F | 22.54 | 1237.54 | 10 | 12.2 | 619.7847 | 2 | 55.2 | 28 | 37 |  | SPIDER |
| R.GGFY(+44.99)ESHDSFVVDSS.Y | 22.54 | 1676.659 | 15 | -2.3 | 839.3348 | 2 | 58.3 | 105 | 119 | Oxidation to nitro | PEAKS PTM |
| **FA-TRYPSIN** |  |  |  |  |  |  |  |  |  |  |  |
| **Peptide** | **-10lgP** | **Mass** | **Length** | **ppm** | **m/z** | **z** | **RT** | **Start** | **End** | **PTM** | **Found By** |
| R.VPGASSSAAAASSASAGGR.G | 64.73 | 1560.749 | 19 | -1.7 | 781.3805 | 2 | 38.6 | 86 | 104 |  | PEAKS DB |
| E.GGYGSGSSGAAAAAAAAAAAAAR.R | 55.82 | 1806.861 | 23 | -1.4 | 904.4365 | 2 | 52.9 | 903 | 925 |  | PEAKS DB |
| R.HHDEYVDSNGQLFER.F | 55.44 | 1844.808 | 15 | -1.8 | 615.9421 | 3 | 43.4 | 23 | 37 |  | PEAKS DB |
| R.LVETIVLEEDPYGHEN.I | 53.42 | 1855.884 | 16 | -1.3 | 928.948 | 2 | 59.4 | 60 | 75 |  | PEAKS DB |
| H.DEYVDSNGQLFER.F | 53 | 1570.69 | 13 | -1.6 | 786.351 | 2 | 53.3 | 25 | 37 |  | PEAKS DB |
| D.PYGHENIYEEDVVIK.R | 52.49 | 1803.868 | 15 | -2.2 | 902.9392 | 2 | 47.9 | 70 | 84 |  | PEAKS DB |
| D.GGYGSGSSGAAAAAAAAAAAAAAR.R | 52.15 | 1877.898 | 24 | -1.6 | 939.9548 | 2 | 55.3 | 299 | 322 |  | PEAKS DB |
| H.DEYVDSN(+.98)GQLFER.F | 51.87 | 1571.674 | 13 | -1.5 | 786.8431 | 2 | 55.1 | 25 | 37 | Deamidation (NQ) | PEAKS DB |
| R.HHDEYVDSN(+.98)GQLFER.F | 51.44 | 1845.792 | 15 | -3.1 | 616.2693 | 3 | 46.2 | 23 | 37 | Deamidation (NQ) | PEAKS DB |
| R.H(+27.99)HDEYVDSNGQLFER.F | 51.41 | 1872.803 | 15 | -1.8 | 625.2737 | 3 | 46.7 | 23 | 37 | Formylation | PEAKS PTM |
| D.GGYGSGSSGAAAAAAAAAAAAR.R | 50.5 | 1735.824 | 22 | -0.3 | 868.9189 | 2 | 50.1 | 177 | 198 |  | PEAKS DB |
| D.E(+43.01)YVDSNGQLFER.F | 50.04 | 1498.669 | 12 | -1.4 | 750.3406 | 2 | 59.7 | 26 | 37 | Carbamylation | PEAKS PTM |
| D.EYVDSNGQLFER.F | 49.99 | 1455.663 | 12 | -1.1 | 728.838 | 2 | 49.9 | 26 | 37 |  | PEAKS DB |
| R.LVETIVLEEDPYGHENI.Y | 49.12 | 1968.968 | 17 | -2.3 | 985.489 | 2 | 62.1 | 60 | 76 |  | PEAKS DB |
| R.H(+43.01)HDEYVDSNGQLFER.F | 48.93 | 1887.814 | 15 | -1.7 | 630.2774 | 3 | 46.1 | 23 | 37 | Carbamylation | PEAKS PTM |
| D.EYVDSN(+.98)GQLFER.F | 48.25 | 1456.647 | 12 | -1.9 | 729.3294 | 2 | 51.6 | 26 | 37 | Deamidation (NQ) | PEAKS DB |
| R.N(+27.99)AETRPNLSGNER.L | 48.2 | 1484.697 | 13 | -1.6 | 743.3545 | 2 | 39.3 | 47 | 59 | Formylation | PEAKS PTM |
| R.HHDEY(+15.99)VDSN(+.98)GQLFER.F | 48.1 | 1861.787 | 15 | -3.6 | 621.6006 | 3 | 43.5 | 23 | 37 | Oxidation or Hydroxylation; Deamidation (NQ) | PEAKS DB |
| R.HHDEYVDSN(-17.03)GQLFER.F | 47.89 | 1827.781 | 15 | -1.7 | 610.2667 | 3 | 44.5 | 23 | 37 | Ammonia-loss (N) | PEAKS PTM |
| E.YVDSNGQLFER.F | 47.84 | 1326.62 | 11 | -1.4 | 664.3165 | 2 | 47.3 | 27 | 37 |  | PEAKS DB |
| E.YVDSN(+.98)GQLFER.F | 46.67 | 1327.604 | 11 | -1.4 | 664.8085 | 2 | 50 | 27 | 37 | Deamidation (NQ) | PEAKS DB |
| R.H(+27.99)HDEYVDSN(+.98)GQLFER.F | 46.66 | 1873.787 | 15 | -1.6 | 625.6018 | 3 | 48 | 23 | 37 | Formylation; Deamidation (NQ) | PEAKS PTM |
| Y.VDSNGQLFER.F | 46.62 | 1163.557 | 10 | -1.7 | 582.7849 | 2 | 44.8 | 28 | 37 |  | PEAKS DB |
| R.HHDEY(+15.99)VDSNGQLFER.F | 46.49 | 1860.803 | 15 | -2.1 | 621.2735 | 3 | 41.7 | 23 | 37 | Oxidation or Hydroxylation | PEAKS DB |
| R.HHDEYVDS(-18.01)N(+.98)GQLFER.F | 46.25 | 1827.781 | 15 | -1.9 | 610.2665 | 3 | 45.7 | 23 | 37 | Dehydration; Deamidation (NQ) | PEAKS PTM |
| D.E(-18.01)YVDSNGQLFER.F | 45.66 | 1437.653 | 12 | -1.6 | 719.8323 | 2 | 60.6 | 26 | 37 | Pyro-glu from E | PEAKS PTM |
| H.DEYVDSN(-17.03)GQLFER.F | 45.53 | 1553.663 | 13 | -2.3 | 777.8372 | 2 | 55.8 | 25 | 37 | Ammonia-loss (N) | PEAKS PTM |
| R.LVETIVLEEDPYGHENI(-.98).Y | 45.29 | 1967.984 | 17 | -1.7 | 984.9976 | 2 | 60.2 | 60 | 76 | Amidation | PEAKS PTM |
| R.NAETRPNLSGN(-17.03)ER.L | 45.29 | 1439.675 | 13 | -1 | 720.8442 | 2 | 38 | 47 | 59 | Ammonia-loss (N) | PEAKS PTM |
| R.LVETIVLEEDPYGHEN(-18.01).I | 45.28 | 1837.873 | 16 | -2.4 | 919.9418 | 2 | 57.8 | 60 | 75 | Dehydration | PEAKS PTM |
| R.LVETIVLEEDPYGHEN(-.98).I | 45.22 | 1854.9 | 16 | -1 | 928.4563 | 2 | 57.2 | 60 | 75 | Amidation | PEAKS PTM |
| D.SN(+.98)GQLFER.F | 44.98 | 950.4457 | 8 | -2 | 476.2292 | 2 | 44.4 | 30 | 37 | Deamidation (NQ) | PEAKS DB |
| R.NAETRPNLSGNE.R | 44.21 | 1300.601 | 12 | -1.9 | 651.3064 | 2 | 38.7 | 47 | 58 |  | PEAKS DB |
| W.GDGGYGSDSAAAAAAAAAAAAAGSGGR.G | 43.92 | 2150.958 | 27 | -1.3 | 1076.485 | 2 | 58.6 | 1120 | 1146 |  | PEAKS DB |
| D.EYVDSN(-17.03)GQLFER.F | 43.43 | 1438.637 | 12 | -1.3 | 720.3246 | 2 | 52.2 | 26 | 37 | Ammonia-loss (N) | PEAKS PTM |
| R.N(+.98)AETRPNLSGNER.L | 43.35 | 1457.686 | 13 | -1.8 | 729.8489 | 2 | 38.2 | 47 | 59 | Deamidation (NQ) | PEAKS DB |
| D.SNGQLFER.F | 43.27 | 949.4617 | 8 | -1.8 | 475.7373 | 2 | 41.5 | 30 | 37 |  | PEAKS DB |
| H.HDEYVDSNGQLFER.F | 42.9 | 1707.749 | 14 | -1.6 | 570.256 | 3 | 46.4 | 24 | 37 |  | PEAKS DB |
| R.LVETIVLEEDPYGHENIYEEDVVIK.R | 41.92 | 2944.459 | 25 | -1.3 | 982.4924 | 3 | 62.5 | 60 | 84 |  | PEAKS DB |
| D.S(+43.01)NGQLFER.F | 41.85 | 992.4675 | 8 | -1.6 | 497.2403 | 2 | 50.8 | 30 | 37 | Carbamylation | PEAKS PTM |
| R.AAGSAAAAAAAAAAAAAGSGAGGHGGGYG.W | 41.83 | 2141.984 | 29 | -2 | 715.0004 | 3 | 54.9 | 205 | 233 |  | PEAKS DB |
| V.DSNGQLFER.F | 41.63 | 1064.489 | 9 | -2.1 | 533.2505 | 2 | 45.3 | 29 | 37 |  | PEAKS DB |
| R.H(+43.01)HDEYVDSN(+.98)GQLFER.F | 41.53 | 1888.798 | 15 | -2.3 | 630.605 | 3 | 47.9 | 23 | 37 | Carbamylation; Deamidation (NQ) | PEAKS PTM |
| R.HHD(+15.99)EY(+15.99)VDSNGQLFER.F | 41.28 | 1876.798 | 15 | -1.7 | 626.6054 | 3 | 46 | 23 | 37 | Oxidation or Hydroxylation | PEAKS DB |
| R.NAETRPNLSGN(-18.01).E | 41.19 | 1153.548 | 11 | -1.9 | 577.78 | 2 | 38.1 | 47 | 57 | Dehydration | PEAKS PTM |
| R.HHDEY(+79.96)VDSNGQLFER.F | 41.17 | 1924.765 | 15 | -1.6 | 642.5944 | 3 | 45.7 | 23 | 37 | Sulfation | PEAKS PTM |
| D.EYVD(-18.01)SNGQLFER.F | 41.1 | 1437.653 | 12 | -1.4 | 719.8325 | 2 | 49.9 | 26 | 37 | Dehydration | PEAKS PTM |
| Y.VDSN(+.98)GQLFER.F | 40.97 | 1164.541 | 10 | -2.3 | 583.2765 | 2 | 46.6 | 28 | 37 | Deamidation (NQ) | PEAKS DB |
| R.NAE(+21.98)TRPNLSGNER.L | 40.96 | 1478.684 | 13 | 0.1 | 740.3492 | 2 | 43.3 | 47 | 59 | Sodium adduct | PEAKS PTM |
| D.PYGHENIYEEDVVIKR.V | 40.83 | 1959.969 | 16 | -0.8 | 654.3298 | 3 | 44.2 | 70 | 85 |  | PEAKS DB |
| R.HHD(+15.99)EYVDSNGQLFER.F | 40.71 | 1860.803 | 15 | -1.4 | 621.2739 | 3 | 46.4 | 23 | 37 | Oxidation or Hydroxylation | PEAKS DB |
| D.SAAAAAAAAAAAAAGSGGR.G | 40.42 | 1442.723 | 19 | -1.3 | 722.3676 | 2 | 48.9 | 1128 | 1146 |  | PEAKS DB |
| R.AAGSAAAAAAAAAAAAGGSGAGGHGGGYG(-.98).W | 39.62 | 2126.984 | 29 | -1.5 | 710.0009 | 3 | 49.8 | 771 | 799 | Amidation | PEAKS PTM |
| R.NAETRPNLSGNE(-.98).R | 39.25 | 1299.617 | 12 | -1.7 | 650.8145 | 2 | 38.2 | 47 | 58 | Amidation | PEAKS PTM |
| A.E(-18.01)TRPNLSGNER.L | 39.16 | 1253.611 | 11 | -1.6 | 627.8119 | 2 | 38.9 | 49 | 59 | Pyro-glu from E | PEAKS PTM |
| D.SN(-17.03)GQLFER.F | 39.06 | 932.4352 | 8 | -1.2 | 467.2243 | 2 | 51.1 | 30 | 37 | Ammonia-loss (N) | PEAKS PTM |
| N.A(+164.06)ETRPNLSGNER.L | 38.85 | 1506.719 | 12 | -2.7 | 754.3648 | 2 | 40.3 | 48 | 59 | O-Diisopropylphosphorylation | PEAKS PTM |
| R.HHDEY(+79.96)VDSN(+.98)GQLFER.F | 38.74 | 1925.749 | 15 | -1.1 | 642.9227 | 3 | 47.1 | 23 | 37 | Sulfation; Deamidation (NQ) | PEAKS PTM |
| K.RVPGASSSAAAASSASAGGR.G | 38.55 | 1716.85 | 20 | -1.3 | 573.2899 | 3 | 37.9 | 85 | 104 |  | PEAKS DB |
| R.AAGSAAAAAAAAAAAAAGSGAGGHGGGYG(-.98).W | 38.4 | 2141 | 29 | -1.5 | 714.6728 | 3 | 53.4 | 205 | 233 | Amidation | PEAKS PTM |
| D.SN(+.98)GQ(+.98)LFER.F | 38.32 | 951.4297 | 8 | -2.1 | 476.7212 | 2 | 46.6 | 30 | 37 | Deamidation (NQ) | PEAKS DB |
| R.NAETRPNLSGNER.L | 38.29 | 1456.702 | 13 | -0.7 | 486.5742 | 3 | 37.6 | 47 | 59 |  | PEAKS DB |
| R.NAETRPNLSGNE(+14.02)R.L | 38.17 | 1470.718 | 13 | -1.9 | 736.3646 | 2 | 38.1 | 47 | 59 | Methylation(others) | PEAKS PTM |
| R.NAETRPNLSGN(-.98).E | 37.94 | 1170.574 | 11 | -1.6 | 586.2934 | 2 | 37.8 | 47 | 57 | Amidation | PEAKS PTM |
| R.NAETRPNLSG.N | 37.86 | 1057.515 | 10 | -1.8 | 529.764 | 2 | 38.4 | 47 | 56 |  | PEAKS DB |
| V.DSN(+.98)GQLFER.F | 37.64 | 1065.473 | 9 | -2.4 | 533.7423 | 2 | 47.7 | 29 | 37 | Deamidation (NQ) | PEAKS DB |
| R.NAETRPN(+.98)LSGNER.L | 37.59 | 1457.686 | 13 | -1.8 | 729.8489 | 2 | 39.4 | 47 | 59 | Deamidation (NQ) | PEAKS DB |
| R.HHDEY(+15.99)VDSNGQ(+.98)LFER.F | 37.41 | 1861.787 | 15 | -2.7 | 621.6011 | 3 | 48.2 | 23 | 37 | Oxidation or Hydroxylation; Deamidation (NQ) | PEAKS DB |
| D.SAAAAAAAAAAAAAGGSGGR.G | 37.4 | 1499.744 | 20 | -1.2 | 750.8784 | 2 | 48.7 | 845 | 864 |  | PEAKS DB |
| Y.VDSN(-17.03)GQLFER.F | 36.71 | 1146.531 | 10 | -1.7 | 574.2715 | 2 | 46.8 | 28 | 37 | Ammonia-loss (N) | PEAKS PTM |
| R.NAETRPNLSGN.E | 36.47 | 1171.558 | 11 | -1.4 | 586.7855 | 2 | 38.3 | 47 | 57 |  | PEAKS DB |
| R.NAETRPNLSGN(+.98).E | 36.17 | 1172.542 | 11 | -1.4 | 587.2775 | 2 | 38.8 | 47 | 57 | Deamidation (NQ) | PEAKS DB |
| R.N(+.98)AETRPN(+.98)LSGNER.L | 35.98 | 1458.67 | 13 | -2.4 | 487.2294 | 3 | 38.6 | 47 | 59 | Deamidation (NQ) | PEAKS DB |
| R.NAE(+21.98)TR(+14.02)PNLSGNER.L | 35.62 | 1492.7 | 13 | 1.2 | 747.3579 | 2 | 44.9 | 47 | 59 | Sodium adduct; Methylation(KR) | PEAKS PTM |
| R.NAETRPNLS.G | 35.51 | 1000.494 | 9 | -2 | 501.2531 | 2 | 38.2 | 47 | 55 |  | PEAKS DB |
| R.GGFYESHDSFVVD.S | 35.3 | 1457.61 | 13 | -1.6 | 729.811 | 2 | 55.1 | 105 | 117 |  | PEAKS DB |
| D.SAAAAAAAAAAAAGSGGR.G | 35.23 | 1371.685 | 18 | -1.4 | 686.8491 | 2 | 45.6 | 2207 | 2224 |  | PEAKS DB |
| H.D(+43.01)EYVDSNGQLFER.F | 35.05 | 1613.696 | 13 | -1.7 | 807.8538 | 2 | 60.2 | 25 | 37 | Carbamylation | PEAKS PTM |
| R.AAGSAAAAAAAAAAAAAGSGAGGHGGG.Y | 34.61 | 1921.899 | 27 | -2.8 | 641.6385 | 3 | 52.7 | 205 | 231 |  | PEAKS DB |
| R.NAE(+14.02)TRPNLSGNER.L | 34.28 | 1470.718 | 13 | -2.6 | 491.2452 | 3 | 38.1 | 47 | 59 | Methylation(others) | PEAKS PTM |
| N.GQ(+.98)LFER.F | 33.38 | 749.3708 | 6 | -2.6 | 375.6917 | 2 | 42.7 | 32 | 37 | Deamidation (NQ) | PEAKS DB |
| N.A(+43.01)ETRPNLSGNER.L | 33.33 | 1385.665 | 12 | -1.4 | 693.8386 | 2 | 39.4 | 48 | 59 | Carbamylation | PEAKS PTM |
| N.AETRPNLSGNER.L | 33.24 | 1342.659 | 12 | -1.6 | 672.3357 | 2 | 37.5 | 48 | 59 |  | PEAKS DB |
| R.NAETRPNLS(-.98).G | 33.19 | 999.5098 | 9 | -1.9 | 500.7612 | 2 | 37.8 | 47 | 55 | Amidation | PEAKS PTM |
| R.GGFYESHD.S | 33.13 | 910.3457 | 8 | -2.4 | 456.179 | 2 | 40 | 105 | 112 |  | PEAKS DB |
| R.HHDEYVDS(-18.01)NGQLFER.F | 33.02 | 1826.797 | 15 | -1.8 | 457.7058 | 4 | 42.6 | 23 | 37 | Dehydration | PEAKS PTM |
| R.HHDEYVDSN(+15.99)GQ(+.98)LFER.F | 32.8 | 1861.787 | 15 | -3.2 | 621.6008 | 3 | 43.8 | 23 | 37 | Oxidation or Hydroxylation; Deamidation (NQ) | PEAKS DB |
| R.NAETRPNL.S | 32.75 | 913.4617 | 8 | -2 | 457.7372 | 2 | 38.7 | 47 | 54 |  | PEAKS DB |
| D.S(+27.99)NGQLFER.F | 32.19 | 977.4566 | 8 | -2 | 489.7346 | 2 | 51.2 | 30 | 37 | Formylation | PEAKS PTM |
| D.GGYGSGSSGAAAAAAAAAAAAAAAR.R | 32.15 | 1948.935 | 25 | -2 | 650.651 | 3 | 57.7 | 1539 | 1563 |  | PEAKS DB |
| R.GGFYESHDSFVVDS.S | 31.85 | 1544.642 | 14 | -2.4 | 773.3264 | 2 | 54.3 | 105 | 118 |  | PEAKS DB |
| D.SN(-17.03)GQLFERFTTR.K | 31.85 | 1437.7 | 12 | -1.3 | 719.8564 | 2 | 57 | 30 | 41 | Ammonia-loss (N) | PEAKS PTM |
| G.GYGSGSSGAAAAAAAAAAAAR.R | 31.72 | 1678.802 | 21 | -1.8 | 560.607 | 3 | 50.6 | 178 | 198 |  | PEAKS DB |
| R.NAETRPNLSGN(+15.99)ER.L | 31.55 | 1472.697 | 13 | -1.3 | 491.9055 | 3 | 37.1 | 47 | 59 | Oxidation or Hydroxylation | PEAKS DB |
| R.AAGSAAAAAAAAAAAAAGSGAGGHGGG(-.98).Y | 31.37 | 1920.915 | 27 | -0.1 | 641.3122 | 3 | 51.1 | 205 | 231 | Amidation | PEAKS PTM |
| R.N(+.98)AETRPNLSGNER(+14.02).L | 31.03 | 1471.702 | 13 | -1.9 | 491.5735 | 3 | 39 | 47 | 59 | Deamidation (NQ); Methylation(KR) | PEAKS PTM |
| R.NAETRPNLSGNER(+28.03).L | 30.99 | 1484.733 | 13 | -1.4 | 495.9176 | 3 | 38.8 | 47 | 59 | Dimethylation(KR) | PEAKS PTM |
| R.LVETIVLEEDPYGHENIY(-.98).E | 30.73 | 2131.047 | 18 | -2.5 | 1066.528 | 2 | 62.3 | 60 | 77 | Amidation | PEAKS PTM |
| R.GGFYESHDSFVVDSSYGS.S | 30.65 | 1938.791 | 18 | -1.5 | 970.4012 | 2 | 57 | 105 | 122 |  | PEAKS DB |
| D.PYGHENIYEED.V | 30.24 | 1364.552 | 11 | -2.3 | 683.2817 | 2 | 42.9 | 70 | 80 |  | PEAKS DB |
| D.EYVDSN(+.98)GQ(+.98)LFER.F | 29.53 | 1457.631 | 12 | -1.5 | 729.8217 | 2 | 53.5 | 26 | 37 | Deamidation (NQ) | PEAKS DB |
| N.GQLFER.F | 29.33 | 748.3868 | 6 | -2.2 | 375.1999 | 2 | 41.3 | 32 | 37 |  | PEAKS DB |
| R.HHDEYVDSN(+.98)GQ(+.98)LFER.F | 29.29 | 1846.776 | 15 | 7.3 | 616.6037 | 3 | 52.6 | 23 | 37 | Deamidation (NQ) | PEAKS DB |
| R.NAETRPNL(-.98).S | 29.02 | 912.4777 | 8 | -1.2 | 457.2456 | 2 | 37.9 | 47 | 54 | Amidation | PEAKS PTM |
| R.HHDEYVDSN(-17.03)GQ.L | 29.01 | 1282.485 | 11 | -1.7 | 642.2487 | 2 | 44.6 | 23 | 33 | Ammonia-loss (N) | PEAKS PTM |
| R.AAGSAAAAAAAAAAAAAGSGAGGHG.G | 28.85 | 1807.856 | 25 | -2 | 603.6248 | 3 | 53.4 | 205 | 229 |  | PEAKS DB |
| Y.GS(+27.05)DSAAAAAAAAAAAAGSGGR.G | 28.84 | 1657.813 | 21 | -1 | 829.913 | 2 | 48 | 2204 | 2224 | Ethyl amino | PEAKS PTM |
| N.G(+43.01)QLFER.F | 28.67 | 791.3926 | 6 | -1.6 | 396.7029 | 2 | 52.6 | 32 | 37 | Carbamylation | PEAKS PTM |
| R.N(+.98)AETRPNL.S | 28.34 | 914.4457 | 8 | -2.3 | 458.2291 | 2 | 40.9 | 47 | 54 | Deamidation (NQ) | PEAKS DB |
| R.AAGSAAAAAAAAAAAAGGSGAGGHGGG.Y | 27.99 | 1907.883 | 27 | -1.3 | 636.9675 | 3 | 48.8 | 771 | 797 |  | PEAKS DB |
| D.SNGQ(+.98)LFERFTTR.K | 27.64 | 1455.711 | 12 | -2.4 | 728.8608 | 2 | 52 | 30 | 41 | Deamidation (NQ) | PEAKS DB |
| R.GGGYGLGD.G | 27.58 | 694.2922 | 8 | -1.3 | 695.2985 | 1 | 47 | 140 | 147 |  | PEAKS DB |
| R.NAETRPNLSGN(+15.00).E | 26.86 | 1186.558 | 11 | -1.8 | 594.2852 | 2 | 39.5 | 47 | 57 | Deamidation followed by a methylation | PEAKS PTM |
| R.HHDEYVDSN(+.98).G | 26.81 | 1115.416 | 9 | -1.8 | 558.7141 | 2 | 37.5 | 23 | 31 | Deamidation (NQ) | PEAKS DB |
| D.SAAAAAAAAAAAAAGSGGRGSGGGYG(-.98).W | 26.74 | 1976.941 | 26 | -1.9 | 659.9864 | 3 | 49.5 | 1128 | 1153 | Amidation | PEAKS PTM |
| R.SAGGHPLLSI(-.98).C | 26.38 | 949.5345 | 10 | -1.9 | 475.7736 | 2 | 47 | 2781 | 2790 | Amidation | PEAKS PTM |
| A.ETRPNLSGNER.L | 26.3 | 1271.622 | 11 | -2 | 636.8169 | 2 | 37.5 | 49 | 59 |  | PEAKS DB |
| R.GGFYESHD(+14.02).S | 26.27 | 924.3613 | 8 | -1.9 | 463.1871 | 2 | 40.3 | 105 | 112 | Methylation(others) | PEAKS PTM |
| R.VPGASSSAAAASS(-18.01)ASAGGR.G | 26.25 | 1542.739 | 19 | -2.2 | 772.3749 | 2 | 44.3 | 86 | 104 | Dehydration | PEAKS PTM |
| G.Q(-17.03)LFERFTTR.K | 25.46 | 1179.604 | 9 | -2 | 590.8079 | 2 | 58 | 33 | 41 | Pyro-glu from Q | PEAKS PTM |
| N.GQLFERFTTR.K | 25.25 | 1253.652 | 10 | -1.7 | 627.832 | 2 | 46.7 | 32 | 41 |  | PEAKS DB |
| R.GGFYESHDSFVVD(+14.02).S | 25.23 | 1471.626 | 13 | -0.8 | 736.8194 | 2 | 57.2 | 105 | 117 | Methylation(others) | PEAKS PTM |
| D.PYGHENIYEED(+14.02).V | 25.2 | 1378.568 | 11 | -2.1 | 690.2897 | 2 | 44.3 | 70 | 80 | Methylation(others) | PEAKS PTM |
| R.N(+.98)AETRPNLSG.N | 25.15 | 1058.499 | 10 | -2.3 | 530.2557 | 2 | 39.9 | 47 | 56 | Deamidation (NQ) | PEAKS DB |
| R.HHDEYVDSNG.Q | 25.01 | 1171.453 | 10 | -1.4 | 586.733 | 2 | 37.2 | 23 | 32 |  | PEAKS DB |
| R.NAETRPNL(+14.02).S | 24.94 | 927.4774 | 8 | -2.3 | 464.7449 | 2 | 40.7 | 47 | 54 | Methylation(C-term) | PEAKS PTM |
| D.R(+.98)AAGSAAAAAAAAAAAAAGSGAGGHGGG.Y | 23.9 | 2078.984 | 28 | -1.8 | 694.0007 | 3 | 55.4 | 204 | 231 | Deamidation (R) | PEAKS PTM |
| R.GGGYGLGD(+14.02).G | 23.72 | 708.3079 | 8 | -1.7 | 709.3139 | 1 | 47.3 | 140 | 147 | Methylation(others) | PEAKS PTM |
| G.QLFER.F | 23.52 | 691.3653 | 5 | -2.3 | 346.6891 | 2 | 40.9 | 33 | 37 |  | PEAKS DB |
| R.NAETRPNLSGN(+.98)ERLVETIVLEED.P | 23.51 | 2598.277 | 23 | -1.4 | 867.0985 | 3 | 63.8 | 47 | 69 | Deamidation (NQ) | PEAKS DB |
| R.N(+43.01)AETRPNLSGNER.L | 23.43 | 1499.708 | 13 | -1.9 | 750.8597 | 2 | 39.2 | 47 | 59 | Carbamylation | PEAKS PTM |
| N.GQLFE(+14.02)R.F | 23.42 | 762.4024 | 6 | -2.4 | 382.2076 | 2 | 43.2 | 32 | 37 | Methylation(others) | PEAKS PTM |
| R.NAETR(-42.02)PNLSGNER.L | 23.22 | 1414.68 | 13 | -1.9 | 472.5664 | 3 | 37.4 | 47 | 59 | Ornithine from Arginine | PEAKS PTM |
| N.G(+27.99)QLFER.F | 23.11 | 776.3817 | 6 | -1.4 | 777.3879 | 1 | 54.1 | 32 | 37 | Formylation | PEAKS PTM |
| A.ETRPN(+.98)LSGNER.L | 22.94 | 1272.606 | 11 | -1.5 | 637.3093 | 2 | 37.9 | 49 | 59 | Deamidation (NQ) | PEAKS DB |
| R.AAGSAAAAAAAAAAAAAG(-.98).S | 22.71 | 1283.658 | 18 | -1.8 | 642.8351 | 2 | 58 | 205 | 222 | Amidation | PEAKS PTM |
| R.NAETRPN(+.98)LSG.N | 22.53 | 1058.499 | 10 | -1.9 | 530.2559 | 2 | 38.9 | 47 | 56 | Deamidation (NQ) | PEAKS DB |
| R.SAGGHPLLSIC(+57.02)(-.98).C | 22.21 | 1109.565 | 11 | -3.3 | 555.788 | 2 | 47.4 | 2781 | 2791 | Carbamidomethylation; Amidation | PEAKS PTM |
| D.SN(+.98)GQLFERFTTR.K | 22.16 | 1455.711 | 12 | -1.8 | 486.2433 | 3 | 52 | 30 | 41 | Deamidation (NQ) | PEAKS DB |
| R.NAE(+43.99)TR(+14.02)PNLSGNER.L | 22.15 | 1514.707 | 13 | -2.5 | 505.9084 | 3 | 38.3 | 47 | 59 | Carboxylation (E); Methylation(KR) | PEAKS PTM |
| D.GGYGSGSSGAAAAAAAAAAAAAARR.A | 21.02 | 2033.999 | 25 | -2 | 679.0056 | 3 | 51.2 | 299 | 323 |  | PEAKS DB |
| R.SAGGHPLLSI.C | 20.96 | 950.5185 | 10 | -2 | 476.2656 | 2 | 50.8 | 2781 | 2790 |  | PEAKS DB |
| **CHYMOTRYPSIN** |  |  |  |  |  |  |  |  |  |  |  |
| **Peptide** | **-10lgP** | **Mass** | **Length** | **ppm** | **m/z** | **z** | **RT** | **Start** | **End** | **PTM** | **Found By** |
| W.GDGGYGSDSAAAAAAAAAAAAAGSGGRGSGGGY.G | 85.84 | 2629.139 | 33 | 3.9 | 1315.582 | 2 | 77.9 | 1120 | 1152 |  | PEAKS DB |
| Y.GHENIYEEDVVIKRVPGASSSAAAASSASAGGRGGF.Y | 84.54 | 3503.703 | 36 | 3 | 876.9356 | 4 | 72.9 | 72 | 107 |  | PEAKS DB |
| F.VLDGGYDSEGSAAAAAAAAAAAASSGARSAGGHPL.L | 81.38 | 3027.428 | 35 | 3.6 | 1010.154 | 3 | 83.2 | 2753 | 2787 |  | PEAKS DB |
| Y.GHENIYEEDVVIKRVPGASSSAAAASSASAGGRGGFY.E | 80.85 | 3666.766 | 37 | 3.6 | 917.7021 | 4 | 74.8 | 72 | 108 |  | PEAKS DB |
| Y.GSGSSGAAAAAAAAAAAAAARRAGHDRAAGSAAAAAAAAAAAAAGSGAGGHGGGY.G | 80.47 | 4360.09 | 55 | 2.9 | 873.0278 | 5 | 82.2 | 621 | 675 |  | PEAKS DB |
| Y.GHENIYEEDVVIKRVPGASS.S | 76.07 | 2198.097 | 20 | 2.3 | 733.7078 | 3 | 68.5 | 72 | 91 |  | PEAKS DB |
| Y.GWGDGGYGSDSAAAAAAAAAAAAAGSGGRGSGGGY.G | 76.02 | 2872.24 | 35 | 3.8 | 1437.132 | 2 | 83.7 | 1118 | 1152 |  | PEAKS DB |
| Y.GWGDGGYGSDSAAAAAAAAAAAAAGGSGAGGHGGGY.G | 75.26 | 2894.224 | 36 | 5.8 | 1448.128 | 2 | 84 | 799 | 834 |  | PEAKS DB |
| L.DGGYDSEGSAAAAAAAAAAAASSGARSAGGHPL.L | 73.93 | 2815.276 | 33 | 3.8 | 939.436 | 3 | 81.9 | 2755 | 2787 |  | PEAKS DB |
| Y.GHENIYEEDVVIKRVPGASSSA.A | 69.24 | 2356.166 | 22 | 3.6 | 786.3987 | 3 | 69 | 72 | 93 |  | PEAKS DB |
| Y.GWGDGGYGSDSAAAAAAAAAAAAAGGSGGRGSGDGY.G | 69.14 | 2987.267 | 36 | 5.4 | 1494.649 | 2 | 83.4 | 835 | 870 |  | PEAKS DB |
| Y.GWGDEGYGSGSAAAAAAAAAAAAAGSGAGGSGGGY.G | 68.92 | 2801.191 | 35 | 3.1 | 934.7406 | 3 | 84.9 | 392 | 426 |  | PEAKS DB |
| W.GDGGYGSDSAAAAAAAAAAAAAGGSGAGGHGGGY.G | 64.68 | 2651.123 | 34 | 3.3 | 884.7178 | 3 | 78.2 | 801 | 834 |  | PEAKS DB |
| Y.GRGDGGYGSGSSAAAAAAAAAAAARRAGY.D | 63.8 | 2511.196 | 29 | 3.5 | 628.8085 | 4 | 77.4 | 2689 | 2717 |  | PEAKS DB |
| F.VVDSSYGSSSSSSAAAAAGSGAGGRGGGY.G | 63.06 | 2479.085 | 29 | 4.7 | 1240.555 | 2 | 53.9 | 115 | 143 |  | PEAKS DB |
| Y.GWGDGGYGSDSAAAAAAAAAAAAAGSGAGGSGGGY.G | 61.86 | 2787.176 | 35 | 3.7 | 930.0692 | 3 | 85.1 | 517 | 551 |  | PEAKS DB |
| R.RAGHDRAAGSAAAAAAAAAAAAGGSGAGGY.D | 61.19 | 2455.17 | 30 | 2.4 | 819.3992 | 3 | 68.5 | 1242 | 1271 |  | PEAKS DB |
| Y.GWGDGGYGSGSAAAAAAAAGSGAGGSGGGY.G | 61.11 | 2373.985 | 30 | 3.5 | 1188.004 | 2 | 70.1 | 1667 | 1696 |  | PEAKS DB |
| Y.GHENIYEEDVVIKRVPGASSSAAAAS.S | 60.61 | 2656.309 | 26 | 2.9 | 886.4462 | 3 | 68.8 | 72 | 97 |  | PEAKS DB |
| Y.GWGDDGSGSGSAAAAAAAAAAAAGSGAGGSGGGY.G | 60.06 | 2640.107 | 34 | 3.4 | 881.046 | 3 | 78.2 | 233 | 266 |  | PEAKS DB |
| H.DRAAGSAAAAAAAAAAAAAGSGAGGHGGGY.G | 58.64 | 2356.09 | 30 | 3 | 786.3731 | 3 | 74.5 | 203 | 232 |  | PEAKS DB |
| Y.GSDSAAAAAAAAAAAAGSGGRGSGDGY.G | 58.12 | 2166.953 | 27 | 3.3 | 723.3272 | 3 | 69.7 | 2204 | 2230 |  | PEAKS DB |
| Y.GWGDEGYGSGSAAAAAAAAAAAAGSGAGGAGDGGY.G | 57.78 | 2829.186 | 35 | 4.3 | 944.0733 | 3 | 82.6 | 267 | 301 |  | PEAKS DB |
| Y.GWGDEGYGSGSAAAAAAAAAAAAAAGSGAGGSGGGY.G | 57.42 | 2872.228 | 36 | 3.8 | 958.4203 | 3 | 87.2 | 1823 | 1858 |  | PEAKS DB |
| H.DRAAGSAAAAAAAAAAAAGGSGAGGHGGGY.G | 57.41 | 2342.075 | 30 | 3.2 | 781.7014 | 3 | 69.6 | 769 | 798 |  | PEAKS DB |
| Y.GSGSSGAAAAAAAAAAAAARRAGHDRAAGSAAAAAAAAAAAAGGSGAGGY.D | 57.2 | 3966.914 | 50 | 2.9 | 992.7386 | 4 | 81.2 | 1222 | 1271 |  | PEAKS DB |
| Y.EEDVVIKRVPGASSSAAAASSASAGGRGGFY.E | 56.94 | 2953.453 | 31 | 3.5 | 985.4951 | 3 | 69 | 78 | 108 |  | PEAKS DB |
| R.AGHDRAAGSAAAAAAAAAAAAAGSGAGGHGGGY.G | 54.78 | 2621.208 | 33 | 4.3 | 656.312 | 4 | 72.3 | 200 | 232 |  | PEAKS DB |
| Y.GSDSAAAAAAAAAAAAAGSGAGGYGDGY.G | 54.49 | 2228.957 | 28 | 3.9 | 1115.49 | 2 | 79.2 | 1443 | 1470 |  | PEAKS DB |
| Y.ESHDSFVVDSSYGSSSSSSAAAAAGSGAGGRGGGY.G | 54.12 | 3181.346 | 35 | 3 | 1061.459 | 3 | 64.6 | 109 | 143 |  | PEAKS DB |
| A.GHDRAAGSAAAAAAAAAAAAAGSGAGGHGGGY.G | 53.44 | 2550.171 | 32 | 3.2 | 638.552 | 4 | 72.2 | 201 | 232 |  | PEAKS DB |
| Y.GSDSAAAAAAAAAAAAAGGSGAGGHGGGY.G | 53.26 | 2201.969 | 29 | 3.1 | 734.9991 | 3 | 72.4 | 806 | 834 |  | PEAKS DB |
| F.ERNAETRPN(+.98)LSGNERLVETIVL.E | 53.15 | 2510.309 | 22 | 3.7 | 837.78 | 3 | 90.3 | 45 | 66 | Deamidation (NQ) | PEAKS DB |
| R.RAGHDRAAGSAAAAAAAAAAAAAGSGAGGHGGGY.G | 53.11 | 2777.309 | 34 | 3.1 | 695.3367 | 4 | 70.9 | 199 | 232 |  | PEAKS DB |
| Y.EEDVVIKRVPGASSSAAAASSASAGGRGGF.Y | 52.39 | 2790.39 | 30 | 3.3 | 698.607 | 4 | 66.2 | 78 | 107 |  | PEAKS DB |
| Y.GWGDDGYGSGSAAAAAAAAAAAAAGSGAGGVGGGY.G | 51.9 | 2799.212 | 35 | 3.8 | 934.0814 | 3 | 87.9 | 2494 | 2528 |  | PEAKS DB |
| Y.GWGDGGYGSDSAAAAAAAAAAAAAGSGAGGY.G | 51.77 | 2529.079 | 31 | 3.1 | 1265.551 | 2 | 87.6 | 1276 | 1306 |  | PEAKS DB |
| Y.GSGSAAAAAAAAAAAAGSGAGGAGDGGY.G | 51.69 | 2064.91 | 28 | 3.8 | 689.3131 | 3 | 70.3 | 274 | 301 |  | PEAKS DB |
| F.ERN(+.98)AETRPNLSGNERLVETIVL.E | 51.68 | 2510.309 | 22 | 4.4 | 837.7806 | 3 | 87.7 | 45 | 66 | Deamidation (NQ) | PEAKS DB |
| Y.GWGDGGYGSDSAAAAAAAAAAAAGSGAGGY.G | 51.59 | 2458.042 | 30 | 2.2 | 1230.031 | 2 | 84.6 | 960 | 989 |  | PEAKS DB |
| Y.GHENIYEEDVVIKRVPG.A | 51.36 | 1952.996 | 17 | 2.2 | 652.0072 | 3 | 68.6 | 72 | 88 |  | PEAKS DB |
| Y.GWGDGGYGSDSAAAAAAAAAAAAGSGAGGVGGGY.G | 50.97 | 2728.175 | 34 | 4.8 | 910.4032 | 3 | 85.3 | 2426 | 2459 |  | PEAKS DB |
| Y.GSGSSAAAAAAAAAAAARRAGY.D | 50.17 | 1848.919 | 22 | 3.5 | 925.47 | 2 | 75.3 | 2696 | 2717 |  | PEAKS DB |
| Y.GSDSAAAAAAAAAAAAAGGSGGRGSGDGY.G | 49.81 | 2295.011 | 29 | 3.3 | 766.0135 | 3 | 73.3 | 842 | 870 |  | PEAKS DB |
| Y.GSGSAAAAAAAAAAAAAGSGAGGAGDGGY.G | 49.3 | 2135.947 | 29 | 3.7 | 1068.985 | 2 | 75 | 717 | 745 |  | PEAKS DB |
| Y.GHENIYEEDVVIKRVPGAS.S | 48.64 | 2111.065 | 19 | 2.7 | 704.6974 | 3 | 68.9 | 72 | 90 |  | PEAKS DB |
| F.ERNAETRPNLSGN(+.98)ERLVETIVL.E | 48.55 | 2510.309 | 22 | 4 | 837.7803 | 3 | 87.9 | 45 | 66 | Deamidation (NQ) | PEAKS DB |
| R.AGHDRAAGSAAAAAAAAAAAAAGSGAGGY.G | 48.48 | 2313.085 | 29 | 3.8 | 772.0384 | 3 | 75.4 | 484 | 512 |  | PEAKS DB |
| Y.GHENIYEEDVVIKRVPGASSSAA.A | 48.39 | 2427.203 | 23 | 3.3 | 810.0776 | 3 | 69.1 | 72 | 94 |  | PEAKS DB |
| Y.GHENIYEEDVVIKRVPGA.S | 47.76 | 2024.033 | 18 | 2.9 | 675.6868 | 3 | 70.7 | 72 | 89 |  | PEAKS DB |
| Y.GHENIYEEDVVIKRVPGASSS.A | 47.57 | 2285.129 | 21 | 2.5 | 762.7188 | 3 | 68.5 | 72 | 92 |  | PEAKS DB |
| R.RAGHDRAAGSAAAAAAAAAAAAAGSGSGGY.G | 47.46 | 2485.181 | 30 | 2.5 | 622.304 | 4 | 72.1 | 323 | 352 |  | PEAKS DB |
| F.ERNAETRPNLSGNERLVETIVL.E | 47.41 | 2509.325 | 22 | 3.9 | 1255.675 | 2 | 85.8 | 45 | 66 |  | PEAKS DB |
| Y.GSGSSAAAAAAAAAAAGSGAGGSDGGY.G | 47.4 | 2039.878 | 27 | 3.7 | 1020.95 | 2 | 65.6 | 2467 | 2493 |  | PEAKS DB |
| W.GDGGYGSGSAAAAAAAAGSGAGGSGGGY.G | 47.14 | 2130.884 | 28 | 4.1 | 1066.454 | 2 | 61.1 | 1669 | 1696 |  | PEAKS DB |
| W.GDGGYGSDSAAAAAAAAAAAAGSGAGGY.G | 46.92 | 2214.941 | 28 | 3.5 | 1108.482 | 2 | 78.3 | 962 | 989 |  | PEAKS DB |
| H.DRAAGSAAAAAAAAAAAAAGSGAGGY.G | 46.82 | 2047.967 | 26 | 3.5 | 1024.994 | 2 | 77.6 | 487 | 512 |  | PEAKS DB |
| Y.DSEGSAAAAAAAAAAAASSGARSAGGHPL.L | 46.77 | 2423.142 | 29 | 3.2 | 808.724 | 3 | 79.1 | 2759 | 2787 |  | PEAKS DB |
| Y.GSGSSGAAAAAAAAAAAAAAR.R | 46.74 | 1600.792 | 21 | 2.8 | 801.4053 | 2 | 71 | 302 | 322 |  | PEAKS DB |
| H.DRAAGSAAAAAAAAAAAAGGSGAGGY.D | 46.57 | 2033.951 | 26 | 4.1 | 1017.987 | 2 | 72.3 | 1246 | 1271 |  | PEAKS DB |
| W.DYEGYGSDSAAAAAAAAAAAAGSGGRGSGDGY.G | 46.49 | 2794.17 | 32 | 3.4 | 932.4005 | 3 | 78 | 2590 | 2621 |  | PEAKS DB |
| Y.GSDSAAAAAAAAAAAAGSGGRGSGDGYGW.G | 46.46 | 2410.053 | 29 | 4.8 | 1206.04 | 2 | 79.6 | 2204 | 2232 |  | PEAKS DB |
| Y.GSGSAAAAAAAAAAAAAAGSGAGGSGGGY.G | 46.18 | 2107.952 | 29 | 3.9 | 1054.987 | 2 | 76.9 | 1830 | 1858 |  | PEAKS DB |
| H.DRAAGSAAAAAAAAAAAAAGSGSGGY.G | 45.98 | 2063.962 | 26 | 4.1 | 1032.992 | 2 | 75.9 | 327 | 352 |  | PEAKS DB |
| Y.GWGDGGYGSD(+21.98)SAAAAAAAAAAAAAGGSGGRGSGDGY.G | 45.86 | 3009.249 | 36 | 3.3 | 1004.093 | 3 | 83.4 | 835 | 870 | Sodium adduct | PEAKS PTM |
| Y.GSGSAAAAAAAAGSGAGGSGGGY.G | 45.77 | 1681.729 | 23 | 3.9 | 841.8751 | 2 | 53.6 | 1674 | 1696 |  | PEAKS DB |
| W.GDGGYGSDSAAAAAAAAAAAAAGSGAGGY.G | 45.42 | 2285.979 | 29 | 2.8 | 763.0023 | 3 | 81.6 | 1278 | 1306 |  | PEAKS DB |
| Y.GSGSSGAAAAAAAAAAAAAR.R | 45.28 | 1529.755 | 20 | 1.8 | 765.8859 | 2 | 67.4 | 906 | 925 |  | PEAKS DB |
| Y.GSDSAAAAAAAAAAAAGSGAGGVGGGY.G | 44.6 | 2035.919 | 27 | 3.5 | 1018.971 | 2 | 75.5 | 2433 | 2459 |  | PEAKS DB |
| Y.GSGSSAAAAAAAAAAAAGSGAGGAGDGGY.G | 44.39 | 2151.942 | 29 | 3.6 | 1076.982 | 2 | 72 | 151 | 179 |  | PEAKS DB |
| Y.GSDSAAAAAAAAAAAAAGSGAGGSGGGY.G | 44.22 | 2094.92 | 28 | 3.4 | 1048.471 | 2 | 75.3 | 524 | 551 |  | PEAKS DB |
| W.GDGGYGSDSAAAAAAAAAAAAAGSGAGGSGGGY.G | 43.74 | 2544.075 | 33 | 5.7 | 1273.052 | 2 | 79.3 | 519 | 551 |  | PEAKS DB |
| Y.GSDSAAAAAAAAAAAAGSGAGGY.G | 43.53 | 1765.787 | 23 | 3.2 | 883.9034 | 2 | 72.9 | 967 | 989 |  | PEAKS DB |
| Y.GHENIYEEDVVIK.R | 43.44 | 1543.752 | 13 | 2.5 | 772.8851 | 2 | 66.5 | 72 | 84 |  | PEAKS DB |
| Y.GSDSAAAAAAAAAAAAAGSGGRGSGGGY.G | 43.3 | 2179.984 | 28 | 3.2 | 1091.003 | 2 | 72 | 1125 | 1152 |  | PEAKS DB |
| K.RVPGASSSAAAASSASAGGRGGFY.E | 43.13 | 2141.025 | 24 | 2.7 | 714.6842 | 3 | 59.8 | 85 | 108 |  | PEAKS DB |
| S.GSGSAAAAAAAAAAAAGSGAGGAGGGY.G | 42.87 | 1949.883 | 27 | 3.6 | 650.9705 | 3 | 71.2 | 1478 | 1504 |  | PEAKS DB |
| F.ERNAETRPNLSGNERL.V | 42.78 | 1854.93 | 16 | 3.3 | 464.7412 | 4 | 54.6 | 45 | 60 |  | PEAKS DB |
| Y.GSSSSSSAAAAAGSGAGGRGGGY.G | 42.21 | 1828.794 | 23 | 3.8 | 915.4075 | 2 | 43.7 | 121 | 143 |  | PEAKS DB |
| R.AGHDRAAGSAAAAAAAAAAAAGGSGAGGY.D | 41.96 | 2299.069 | 29 | 2.9 | 767.3658 | 3 | 70.2 | 1243 | 1271 |  | PEAKS DB |
| Y.GHENIYEEDVVIKRVPGASSSAAAASSA.S | 41.28 | 2814.378 | 28 | 3.7 | 939.1369 | 3 | 69.7 | 72 | 99 |  | PEAKS DB |
| Y.GSDSAAAAAAAAAAAAAGSGAGGY.G | 41.12 | 1836.824 | 24 | 3.2 | 919.4221 | 2 | 77.7 | 1283 | 1306 |  | PEAKS DB |
| Y.GSDDGFVLDGGYDSEGSAAAAAAAAAAAASSGARSAGGHPL.L | 40.48 | 3605.625 | 41 | 3.5 | 1202.887 | 3 | 90 | 2747 | 2787 |  | SPIDER |
| Y.GSGSSGAAAAAAAAAAAAR.R | 40.01 | 1458.717 | 19 | 2.7 | 730.368 | 2 | 63 | 180 | 198 |  | PEAKS DB |
| Y.GHENIYEEDVVIKRVPGASSSAAAA.S | 39.96 | 2569.277 | 25 | 3.9 | 857.4363 | 3 | 69.5 | 72 | 96 |  | PEAKS DB |
| Y.GSGSSGAAAAAAAAAAAAAARRAGH.D | 39.43 | 2022.01 | 25 | 2.9 | 506.5113 | 4 | 69.4 | 302 | 326 |  | PEAKS DB |
| Y.ESHDSFVVDSSY.G | 39.34 | 1370.563 | 12 | 3.3 | 686.2908 | 2 | 63.3 | 109 | 120 |  | PEAKS DB |
| Y.DRAHGAGSAAAAAAAAAAGA(sub P)GATRPVGVY.G | 39.3 | 2508.258 | 29 | 3 | 837.0958 | 3 | 73 | 2718 | 2746 |  | SPIDER |
| Y.GWGDGGYGSDSAAAAAAAAAAAAGSGAGGY(+21.98).G | 39.07 | 2480.024 | 30 | 3 | 1241.023 | 2 | 84.8 | 960 | 989 | Sodium adduct | PEAKS PTM |
| Y.GHENIYEEDVVIKRVPGASS(-.98).S | 39.04 | 2197.113 | 20 | 2.2 | 733.3798 | 3 | 67.8 | 72 | 91 | Amidation | PEAKS PTM |
| Y.GW(+3.99)GDGGYGSDSAAAAAAAAAAAAAGGSGAGGHGGGY.G | 38.59 | 2898.219 | 36 | 3.1 | 967.0832 | 3 | 82.3 | 799 | 834 | Tryptophan oxidation to kynurenin | PEAKS PTM |
| Y.GWGDGGYGSD(+21.98)SAAAAAAAAAAAAAGGSGAGGHGGGY.G | 38.18 | 2916.206 | 36 | 2.7 | 973.0785 | 3 | 83.9 | 799 | 834 | Sodium adduct | PEAKS PTM |
| S.AAAAAAAAAAAAAGSGAGGHGGGY.G | 38.07 | 1798.835 | 24 | 3.2 | 600.6207 | 3 | 63.7 | 209 | 232 |  | PEAKS DB |
| Y.GSGSSGAAAAAAAAAAAAAAARRAGH.D | 37.77 | 2093.047 | 26 | 1.8 | 524.2701 | 4 | 73.7 | 1542 | 1567 |  | PEAKS DB |
| Y.VDSNGQLFERF.T | 37.73 | 1310.626 | 11 | 3.5 | 656.3223 | 2 | 79.6 | 28 | 38 |  | PEAKS DB |
| F.YESHDSFVVDSSY.G | 37.61 | 1533.626 | 13 | 1.7 | 767.8216 | 2 | 67 | 108 | 120 |  | PEAKS DB |
| Y.GSGSAAAAAAAAAAAGSGAGGSGDGY.G | 37.23 | 1952.846 | 26 | 3.6 | 651.9583 | 3 | 65.8 | 2629 | 2654 |  | PEAKS DB |
| F.ERNAETRPNLSGNERLVE.T | 37.16 | 2083.041 | 18 | 3.1 | 521.769 | 4 | 57.8 | 45 | 62 |  | PEAKS DB |
| A.AAAAAAAAAAGSGAGGHGGGY.G | 37.1 | 1585.723 | 21 | 4.1 | 793.8722 | 2 | 51.1 | 212 | 232 |  | PEAKS DB |
| D.RAAGSAAAAAAAAAAAAAGSGAGGHGGGY.G | 36.98 | 2241.064 | 29 | 3.9 | 748.0313 | 3 | 72.5 | 204 | 232 |  | PEAKS DB |
| A.AAAAAAAGSGAGGHGGGY.G | 36.79 | 1372.612 | 18 | 2.8 | 687.3152 | 2 | 41.5 | 215 | 232 |  | PEAKS DB |
| F.VLDGGYDSEGSAAAAAAAAAAAASSGARSAGGHPLL.S | 36.74 | 3140.512 | 36 | 3.9 | 1047.849 | 3 | 89.7 | 2753 | 2788 |  | SPIDER |
| A.AAARRAGHDRAAGSAAAAAAAAAAAAGGSGAGGY.D | 36.51 | 2824.382 | 34 | 1.2 | 707.1037 | 4 | 67.4 | 1238 | 1271 |  | PEAKS DB |
| Y.G(+43.01)SSSSSSAAAAAGSGAGGRGGGY.G | 36.42 | 1871.799 | 23 | 3.4 | 936.9101 | 2 | 47.6 | 121 | 143 | Carbamylation | PEAKS PTM |
| A.AAAAAAAAAAAGSGAGGHGGGY.G | 36.29 | 1656.76 | 22 | 3.9 | 829.3907 | 2 | 55.4 | 211 | 232 |  | PEAKS DB |
| Y.GW(+31.99)GDGGYGSDSAAAAAAAAAAAAAGSGGRGSGGGY.G | 36.23 | 2904.23 | 35 | 3.6 | 969.0872 | 3 | 81.4 | 1118 | 1152 | Dihydroxy | PEAKS PTM |
| Y.GS(+114.04)SSSSSAAAAAGSGAGGRGGGY.G | 35.95 | 1942.836 | 23 | 4.3 | 972.4297 | 2 | 47.3 | 121 | 143 | Ubiquitin | PEAKS PTM |
| A.AAAAAAAAGSGAGGHGGGY.G | 35.9 | 1443.649 | 19 | 2.6 | 722.8337 | 2 | 43.9 | 214 | 232 |  | PEAKS DB |
| Y.GSSSSSSAAAAAGSGAGGRGGGY(+125.90).G | 35.69 | 1954.69 | 23 | 4 | 978.3563 | 2 | 53.4 | 121 | 143 | Iodination | PEAKS PTM |
| A.AAAAAAGSGAGGHGGGY.G | 35.52 | 1301.575 | 17 | 3.8 | 651.7972 | 2 | 39.4 | 216 | 232 |  | PEAKS DB |
| A.KNIRHHDEYVDSNGQLF.E | 35.41 | 2070.987 | 17 | 2.7 | 518.7554 | 4 | 64.1 | 19 | 35 |  | PEAKS DB |
| S.SSAAAAAGSGAGGRGGGY.G | 35.39 | 1423.644 | 18 | 3.4 | 712.8317 | 2 | 41.8 | 126 | 143 |  | PEAKS DB |
| F.ERNAETRPNLSGNERLVET.I | 35.39 | 2184.088 | 19 | 2 | 729.0382 | 3 | 58.7 | 45 | 63 |  | PEAKS DB |
| F.ERNAETRPNL.S | 35.31 | 1198.606 | 10 | 2.3 | 400.5434 | 3 | 46 | 45 | 54 |  | PEAKS DB |
| A.SSASAGGRGGFY.E | 35.31 | 1115.5 | 12 | 3.6 | 558.759 | 2 | 51.2 | 97 | 108 |  | PEAKS DB |
| Y.GHENIYEEDVVI.K | 35.23 | 1415.657 | 12 | 3.3 | 708.838 | 2 | 76.7 | 72 | 83 |  | PEAKS DB |
| S.SAAAAAGSGAGGRGGGY.G | 35.08 | 1336.612 | 17 | 1.5 | 669.3143 | 2 | 40.4 | 127 | 143 |  | PEAKS DB |
| A.AAAAAAAAAGSGAGGHGGGY.G | 35.08 | 1514.686 | 20 | 3.6 | 758.3531 | 2 | 47 | 213 | 232 |  | PEAKS DB |
| K.NIRHHDEYVDSN(+.98)GQLF.E | 35.06 | 1943.876 | 16 | 3 | 648.968 | 3 | 69.5 | 20 | 35 | Deamidation (NQ) | PEAKS DB |
| Y.G(+43.01)R(+14.02)GDGGYGSGSSAAAAAAAAAAAARRAGY.D | 34.98 | 2568.218 | 29 | 2.3 | 643.0632 | 4 | 76.9 | 2689 | 2717 | Carbamylation; Methylation(KR) | PEAKS PTM |
| Y.GSDSAAAAAAAAAAAAGSGAGGY(+21.98).G | 34.54 | 1787.769 | 23 | 3.5 | 894.8947 | 2 | 74.1 | 967 | 989 | Sodium adduct | PEAKS PTM |
| A.AAAAAAAGSGAGGAGDGGY.G | 34.41 | 1421.617 | 19 | 2.5 | 711.8176 | 2 | 45.9 | 161 | 179 |  | PEAKS DB |
| L.EEDPYGHENIY.E | 34.36 | 1364.552 | 11 | 2.7 | 683.2851 | 2 | 59.8 | 67 | 77 |  | PEAKS DB |
| Y.GWGDGGYGSD(+37.95)SAAAAAAAAAAAAAGSGGRGSGGGY.G | 34.31 | 2910.187 | 35 | 3.3 | 971.0727 | 3 | 83.7 | 1118 | 1152 | Replacement of 2 protons by calcium | PEAKS PTM |
| N.IRHHDEYVDSN(+.98)GQLF.E | 34.15 | 1829.833 | 15 | 2.9 | 610.9535 | 3 | 69.1 | 21 | 35 | Deamidation (NQ) | PEAKS DB |
| Y.GSGSAAAAAAAAAAAAGSGAGGAGD(+21.98)GGY.G | 34.06 | 2086.892 | 28 | 4.6 | 1044.458 | 2 | 71.2 | 274 | 301 | Sodium adduct | PEAKS PTM |
| Y.GSGSAAAAAAAAGSGAGGSGGGY(+21.98).G | 33.96 | 1703.711 | 23 | 3.9 | 852.8661 | 2 | 53.7 | 1674 | 1696 | Sodium adduct | PEAKS PTM |
| Y.GSDDGFVLDGGY.D | 33.79 | 1200.493 | 12 | 2.4 | 601.2554 | 2 | 73.7 | 2747 | 2758 |  | PEAKS DB |
| Y.GSGSSGAAAAAAAAAAAARR.A | 33.69 | 1614.819 | 20 | 2.6 | 539.2816 | 3 | 62.3 | 180 | 199 |  | PEAKS DB |
| G.SAAAAAAAAAAAAAGSGAGGHGGGY.G | 33.61 | 1885.867 | 25 | 3 | 629.6313 | 3 | 70.9 | 208 | 232 |  | PEAKS DB |
| G.S(+114.04)DSAAAAAAAAAAAAGSGGRGSGDGY.G | 33.61 | 2223.974 | 26 | 3.3 | 1112.998 | 2 | 69.7 | 2205 | 2230 | Ubiquitin | PEAKS PTM |
| Y.GSGSSGAAAAAAAAAAAAAAARR.A | 33.45 | 1827.93 | 23 | 3.8 | 610.3196 | 3 | 73.5 | 1542 | 1564 |  | PEAKS DB |
| Y.GS(-15.99)GSAAAAAAAAAAAAGSGAGGAGEGGY.G | 33.16 | 2062.93 | 28 | 4 | 1032.477 | 2 | 73.3 | 878 | 905 | Deoxy | PEAKS PTM |
| A.AAAAAAAGGSGAGGHGGGY.G | 33.09 | 1429.633 | 19 | 3.7 | 715.8267 | 2 | 41.2 | 780 | 798 |  | PEAKS DB |
| G.S(+114.04)DSAAAAAAAAAAAAAGSGGRGSGGGY.G | 33.08 | 2237.006 | 27 | 3.3 | 1119.514 | 2 | 73.5 | 1126 | 1152 | Ubiquitin | PEAKS PTM |
| S.SSSSSAAAAAGSGAGGRGGGY.G | 32.98 | 1684.74 | 21 | -3.4 | 843.3744 | 2 | 44.3 | 123 | 143 |  | PEAKS DB |
| Y.GSDSAAAAAAAAAAAAAGSGAGGSGGGY(+21.98).G | 32.97 | 2116.902 | 28 | 4 | 706.6442 | 3 | 75.4 | 524 | 551 | Sodium adduct | PEAKS PTM |
| A.AAAAAAAGSGAGGSGDGY.G | 32.93 | 1380.591 | 18 | 3.7 | 691.3051 | 2 | 43.9 | 1011 | 1028 |  | PEAKS DB |
| A.AAAAAAAAGGSGAGGHGGGY.G | 32.35 | 1500.671 | 20 | 3.8 | 751.3454 | 2 | 43.3 | 779 | 798 |  | PEAKS DB |
| A.AAAAAAAAAAGGSGAGGHGGGY.G | 32.23 | 1642.745 | 22 | 4.1 | 822.3831 | 2 | 50.1 | 777 | 798 |  | PEAKS DB |
| A.AAAAAAAAAGGSGAGGHGGGY.G | 32.22 | 1571.708 | 21 | 3.4 | 786.8638 | 2 | 46.2 | 778 | 798 |  | PEAKS DB |
| Y.GWGDGGYGSDSAAAAAAAAAAAAAGGSGAGGHGGGYG.W | 31.88 | 2951.245 | 37 | 2.7 | 984.7584 | 3 | 83.6 | 799 | 835 |  | SPIDER |
| A.AAAAAGSGAGGHGGGY.G | 31.82 | 1230.538 | 16 | 3.8 | 616.2785 | 2 | 37.6 | 217 | 232 |  | PEAKS DB |
| F.ERNAETRPNLSGNERLVE(+14.02).T | 31.75 | 2097.056 | 18 | 2.9 | 525.2728 | 4 | 62.7 | 45 | 62 | Methylation(others) | PEAKS PTM |
| A.AAAAAAAAAGSGAGGY.G | 31.73 | 1206.563 | 16 | 3.6 | 604.2909 | 2 | 50 | 497 | 512 |  | PEAKS DB |
| Y.GSGSSGAAAAAAAAAAAARRAGH.D | 31.72 | 1879.936 | 23 | 2.3 | 470.9924 | 4 | 64.6 | 180 | 202 |  | PEAKS DB |
| Y.GWGDGGYGSDSAAAAAAAAAAAAAGSGAGGSGGGY(+21.98).G | 31.56 | 2809.158 | 35 | 3.7 | 937.3966 | 3 | 85.2 | 517 | 551 | Sodium adduct | PEAKS PTM |
| A.AAAAAAGGSGAGGHGGGY.G | 31.46 | 1358.596 | 18 | 3.5 | 680.3078 | 2 | 39.1 | 781 | 798 |  | PEAKS DB |
| Y.GSDSAAAAAAAAAAAAAGSGAGGY(+21.98).G | 31.43 | 1858.806 | 24 | 3.1 | 930.413 | 2 | 77.9 | 1283 | 1306 | Sodium adduct | PEAKS PTM |
| S.AAAASSASAGGRGGFY.E | 31.21 | 1399.648 | 16 | 3.2 | 700.8335 | 2 | 54.2 | 93 | 108 |  | PEAKS DB |
| G.S(+114.04)SSSSSAAAAAGSGAGGRGGGY.G | 31.19 | 1885.815 | 22 | 0.8 | 943.9155 | 2 | 44.7 | 122 | 143 | Ubiquitin | PEAKS PTM |
| A.AAAAAAAAAAGSGAGGY.G | 31.07 | 1277.6 | 17 | 4 | 639.8098 | 2 | 54 | 496 | 512 |  | PEAKS DB |
| F.ERN(+.98)AETRPNLSGNERLVE.T | 31.05 | 2084.025 | 18 | 1.6 | 695.6833 | 3 | 59 | 45 | 62 | Deamidation (NQ) | PEAKS DB |
| Y.GW(+3.99)GDGGYGSDSAAAAAAAAAAAAAGSGGRGSGGGY.G | 31.01 | 2876.234 | 35 | 3.4 | 959.7553 | 3 | 82.1 | 1118 | 1152 | Tryptophan oxidation to kynurenin | PEAKS PTM |
| A.KNIRHHDEYVDSN(+.98)GQLF.E | 30.84 | 2071.971 | 17 | 1.7 | 691.6655 | 3 | 67.1 | 19 | 35 | Deamidation (NQ) | PEAKS DB |
| Y.GWGDGGYGSDSQ(sub A)AAAAAAAAAAAAGSGGRGSGGGY.G | 30.64 | 2929.261 | 35 | 4 | 977.4315 | 3 | 83.4 | 1118 | 1152 |  | SPIDER |
| S.SASAGGRGGFY.E | 30.62 | 1028.468 | 11 | 3.2 | 515.2427 | 2 | 50.9 | 98 | 108 |  | PEAKS DB |
| F.ERN(+.98)AETRPNLSGNERLVETIVLEEDPY.G | 30.61 | 3143.537 | 27 | 3.4 | 1048.857 | 3 | 96.3 | 45 | 71 | Deamidation (NQ) | SPIDER |
| F.R(sub V)VDSSYGSSSSSSAAAAAGSGAGGRGGGY.G | 30.46 | 2536.117 | 29 | -0.9 | 846.379 | 3 | 54.2 | 115 | 143 |  | SPIDER |
| Y.DRN(sub A)HGAGSAAAAAAAAAAGA(sub P)GATRPVGVY.G | 30.37 | 2551.264 | 29 | 2.3 | 851.4305 | 3 | 74.8 | 2718 | 2746 |  | SPIDER |
| A.AAAAGGSGAGGHGGGY.G | 30.19 | 1216.522 | 16 | 2.5 | 609.2698 | 2 | 36 | 783 | 798 |  | PEAKS DB |
| N.AETRPNLSGNERLVETIVL.E | 30.17 | 2110.138 | 19 | 2.4 | 1056.079 | 2 | 88.5 | 48 | 66 |  | PEAKS DB |
| F.ERNAETRPNLSGNER.L | 30.03 | 1741.846 | 15 | 2.4 | 581.6238 | 3 | 41.6 | 45 | 59 |  | PEAKS DB |
| Y.GSGSSGAAAAAAAAAAAAAARR.A | 29.98 | 1756.893 | 22 | 3 | 586.64 | 3 | 72.1 | 302 | 323 |  | PEAKS DB |
| N.AETRPNLSGNERL.V | 29.9 | 1455.743 | 13 | 2.3 | 486.256 | 3 | 56.4 | 48 | 60 |  | PEAKS DB |
| Y.N(sub G)SDSAAAAAAAAAAAAAGSGGRGSGGGY.G | 29.85 | 2237.006 | 28 | 2.5 | 746.6777 | 3 | 73.6 | 1125 | 1152 |  | SPIDER |
| F.ERNAETRPNLSGNERLVETIVLEED.P | 29.78 | 2882.437 | 25 | 3.1 | 961.8226 | 3 | 85.6 | 45 | 69 |  | SPIDER |
| A.AAAAAAAAAGSGGRGSGDGY.G | 29.75 | 1607.729 | 20 | 2.8 | 804.8739 | 2 | 45.9 | 2211 | 2230 |  | PEAKS DB |
| Y.GSGSAAAAAAAAAAAGSGAGGSGD(+21.98)GY.G | 29.55 | 1974.828 | 26 | 3.9 | 988.425 | 2 | 65.8 | 2629 | 2654 | Sodium adduct | PEAKS PTM |
| Y.GSGSAAAAAAAAAAAAAAGSGAGGSGGGY(+21.98).G | 29.55 | 2129.934 | 29 | 4.1 | 1065.979 | 2 | 77 | 1830 | 1858 | Sodium adduct | PEAKS PTM |
| Y.N(sub G)HENIYEEDVVIKRVPGASSSAA.A | 29.31 | 2484.224 | 23 | 2.7 | 829.0843 | 3 | 69.2 | 72 | 94 |  | SPIDER |
| G.S(+114.04)DSAAAAAAAAAAAAGSGAGGY.G | 29.29 | 1822.808 | 22 | 3.3 | 912.4143 | 2 | 74.1 | 968 | 989 | Ubiquitin | PEAKS PTM |
| Y.GSSSSSSAAAAAGSGAGGRG.G | 29.13 | 1551.687 | 20 | 3.8 | 776.8539 | 2 | 36.9 | 121 | 140 |  | PEAKS DB |
| G.SAAAAAAAAAAAAGGSGAGGHGGGY.G | 29.11 | 1871.851 | 25 | 3 | 624.9595 | 3 | 65 | 774 | 798 |  | PEAKS DB |
| A.AASSASAGGRGGFY.E | 28.98 | 1257.574 | 14 | -0.4 | 629.7939 | 2 | 52.2 | 95 | 108 |  | PEAKS DB |
| Y.GR(+14.02)GDGGYGSGSSAAAAAAAAAAAGSGAGGSDGGY.G | 28.75 | 2716.171 | 34 | 5.7 | 1359.101 | 2 | 70 | 2460 | 2493 | Methylation(KR) | PEAKS PTM |
| F.E(-18.01)RNAETRPNLSGNERL.V | 28.69 | 1836.919 | 16 | 2.5 | 613.3152 | 3 | 58.1 | 45 | 60 | Pyro-glu from E | PEAKS PTM |
| Y.GSDSAAAAAAAAAAAAGSGAGGVGGGY(+21.98).G | 28.52 | 2057.901 | 27 | 4.6 | 1029.963 | 2 | 75.6 | 2433 | 2459 | Sodium adduct | PEAKS PTM |
| F.YESHDSF.V | 28.4 | 883.3348 | 7 | 2.3 | 442.6757 | 2 | 46.3 | 108 | 114 |  | PEAKS DB |
| H.D(+15.99)RAAGSAAAAAAAAAAAAAGSGGRGSGGGY.G | 28.32 | 2407.122 | 30 | 2.8 | 803.3837 | 3 | 68.1 | 2043 | 2072 | Oxidation or Hydroxylation | PEAKS PTM |
| A.AAAGGSGAGGHGGGY.G | 28.17 | 1145.485 | 15 | 3.6 | 573.7518 | 2 | 34.8 | 784 | 798 |  | PEAKS DB |
| A.AAAAGSGAGGHGGGY.G | 28.17 | 1159.501 | 15 | 2.7 | 580.7592 | 2 | 36.1 | 218 | 232 |  | PEAKS DB |
| A.AAAAAAAAGGSGAGGY.D | 28.14 | 1192.547 | 16 | 2.5 | 597.2824 | 2 | 46.5 | 1256 | 1271 |  | PEAKS DB |
| Y.GHENIY.E | 28.12 | 731.3239 | 6 | 3.1 | 366.6703 | 2 | 43.8 | 72 | 77 |  | PEAKS DB |
| S.SSSSAAAAAGSGAGGRGGGY.G | 28.1 | 1597.708 | 20 | 3.7 | 799.8643 | 2 | 43.6 | 124 | 143 |  | PEAKS DB |
| N.AETRPNLSGNERLVET.I | 28.05 | 1784.902 | 16 | 3 | 595.9763 | 3 | 60.4 | 48 | 63 |  | PEAKS DB |
| F.ERNAETRPNLSGN.E | 28.03 | 1456.702 | 13 | 3.2 | 729.3605 | 2 | 41.9 | 45 | 57 |  | PEAKS DB |
| F.ERNAETRPNLSGNERLVETI.V | 27.86 | 2297.172 | 20 | 2.7 | 575.3019 | 4 | 70.8 | 45 | 64 |  | PEAKS DB |
| Y.VDSN(+.98)GQLF.E | 27.85 | 879.3974 | 8 | 2.3 | 440.707 | 2 | 66.6 | 28 | 35 | Deamidation (NQ) | PEAKS DB |
| A.AAAAAAAGSGGRGSGDGY.G | 27.81 | 1465.655 | 18 | 3.1 | 733.8368 | 2 | 40.8 | 2213 | 2230 |  | PEAKS DB |
| Y.DRAHGAGSAAAAAAAAAAGPGATRPVG(+258.09).V | 27.8 | 2530.227 | 27 | 7.2 | 633.5686 | 4 | 73 | 2718 | 2744 | Diglutamyl | PEAKS PTM |
| A.AAAAAAAAGSGGRGSGDGY.G | 27.79 | 1536.692 | 19 | 3.9 | 769.3561 | 2 | 43 | 2212 | 2230 |  | PEAKS DB |
| A.AAAAAAAAGSGAGGAGDGGY.G | 27.67 | 1492.654 | 20 | 3.9 | 747.3373 | 2 | 47.9 | 160 | 179 |  | PEAKS DB |
| G.WGDGGYGS(+114.04)DSAAAAAAAAAAAAAGSGGRGSGGGY.G | 27.51 | 2929.261 | 34 | 3.8 | 977.4313 | 3 | 83.5 | 1119 | 1152 | Ubiquitin | PEAKS PTM |
| F.ERNAETRPNLSGNERLVET(-15.99).I | 27.45 | 2168.093 | 19 | 2.5 | 543.0319 | 4 | 56.5 | 45 | 63 | Deoxy | PEAKS PTM |
| Y.GW(+3.99)GDGGYGSDSAAAAAAAAAAAAGSGAGGY.G | 27.42 | 2462.037 | 30 | 4.4 | 1232.031 | 2 | 82.9 | 960 | 989 | Tryptophan oxidation to kynurenin | PEAKS PTM |
| A.AAAAAAGSGAGGY.G | 27.33 | 993.4515 | 13 | 3.5 | 497.7348 | 2 | 42.2 | 500 | 512 |  | PEAKS DB |
| G.S(+114.04)GSAAAAAAAAGSGAGGSGGGY.G | 27.28 | 1738.751 | 22 | 3.8 | 870.3859 | 2 | 53.7 | 1675 | 1696 | Ubiquitin | PEAKS PTM |
| F.E(+43.01)R(+14.02)NAETRPNLSGNERLVETIVL.E | 27.14 | 2566.346 | 22 | 1.3 | 856.4571 | 3 | 86.4 | 45 | 66 | Carbamylation; Methylation(KR) | PEAKS PTM |
| A.AAAAAAGSGAGGAGDGGY.G | 27.13 | 1350.58 | 18 | 3.6 | 676.2997 | 2 | 42.4 | 162 | 179 |  | PEAKS DB |
| F.ERNAETRPNLS.G | 27.09 | 1285.638 | 11 | 3.2 | 429.5545 | 3 | 41.6 | 45 | 55 |  | PEAKS DB |
| A.AAAAAAAGSGGRGSGGGY.G | 27.06 | 1407.649 | 18 | 1.9 | 704.8331 | 2 | 40.1 | 1135 | 1152 |  | PEAKS DB |
| A.SAGGRGGFY.E | 26.96 | 870.3984 | 9 | 2.8 | 436.2077 | 2 | 49.7 | 100 | 108 |  | PEAKS DB |
| G.SAAAAAAAAAAAAGGSGAGGY.D | 26.89 | 1563.728 | 21 | 2.8 | 782.8733 | 2 | 67.8 | 1251 | 1271 |  | PEAKS DB |
| A.AAAGSGAGGHGGGY.G | 26.82 | 1088.464 | 14 | 3.1 | 545.2407 | 2 | 34.8 | 219 | 232 |  | PEAKS DB |
| N.AETRPNLSGNERLVETIVLEEDPY.G | 26.81 | 2743.367 | 24 | 3.3 | 915.4658 | 3 | 93.9 | 48 | 71 |  | SPIDER |
| A.AAGSGAGGHGGGY.G | 26.74 | 1017.426 | 13 | 3.3 | 509.7221 | 2 | 33.5 | 220 | 232 |  | PEAKS DB |
| Y.GWGDEGYGSGSAAAAAAA.A | 26.73 | 1567.654 | 18 | 3.6 | 784.837 | 2 | 63.5 | 267 | 284 |  | PEAKS DB |
| F.ERNAETRPNLSGNERLVE(+37.95)TIVL.E | 26.63 | 2547.272 | 22 | 1.5 | 510.4624 | 5 | 86.1 | 45 | 66 | Replacement of 2 protons by calcium | PEAKS PTM |
| F.ERNAETRPN.L | 26.56 | 1085.521 | 9 | 4.5 | 362.8494 | 3 | 28.5 | 45 | 53 |  | PEAKS DB |
| A.AAAAAAAAGSGGRGSGGGY.G | 26.44 | 1478.686 | 19 | 4.3 | 740.3535 | 2 | 42.3 | 1134 | 1152 |  | PEAKS DB |
| Y.N(sub G)HENIYEEDVVIKRVPGAS.S | 26.26 | 2168.086 | 19 | 2 | 723.7041 | 3 | 69.1 | 72 | 90 |  | SPIDER |
| A.AAAAAGSGGRGSGDGY.G | 26.18 | 1323.58 | 16 | 2.7 | 662.7992 | 2 | 36.9 | 2215 | 2230 |  | PEAKS DB |
| G.S(+114.04)GSSAAAAAAAAAAAGSGAGGSDGGY.G | 26.1 | 2096.899 | 26 | 4 | 1049.461 | 2 | 66.5 | 2468 | 2493 | Ubiquitin | PEAKS PTM |
| Y.GSDSAAAAAAAAAAAAAGGSGAGGHGGGYG.W | 25.94 | 2258.99 | 30 | 2 | 754.0054 | 3 | 73.7 | 806 | 835 |  | PEAKS DB |
| F.ERNAETRPNLSGNERLVETIVL(+37.95).E | 25.83 | 2547.272 | 22 | 1.4 | 510.4623 | 5 | 86 | 45 | 66 | Replacement of 2 protons by calcium | PEAKS PTM |
| A.AAAAAGSGAGGAGDGGY.G | 25.77 | 1279.543 | 17 | 3.2 | 640.7808 | 2 | 40.8 | 163 | 179 |  | PEAKS DB |
| Y.ESHDSF.V | 25.76 | 720.2715 | 6 | 3.1 | 361.1441 | 2 | 39 | 109 | 114 |  | PEAKS DB |
| A.AAAAAAAGSGAGGY.G | 25.72 | 1064.489 | 14 | -2.2 | 533.2504 | 2 | 44.4 | 499 | 512 |  | PEAKS DB |
| A.AAAAGSGGRGSGGGY.G | 25.69 | 1194.538 | 15 | 3.1 | 598.278 | 2 | 34.7 | 1138 | 1152 |  | PEAKS DB |
| A.AAAAAGSGGRGSGGGY.G | 25.67 | 1265.575 | 16 | 2.8 | 633.7964 | 2 | 36.1 | 1137 | 1152 |  | PEAKS DB |
| A.AAGGSGAGGHGGGY.G | 25.26 | 1074.448 | 14 | 3.4 | 538.233 | 2 | 33.7 | 785 | 798 |  | PEAKS DB |
| Y.DRAHGN(sub A)GSAAAAAAAAAAGA(sub P)GATRPVGVY.G | 25.14 | 2551.264 | 29 | 3.1 | 851.4312 | 3 | 74.9 | 2718 | 2746 |  | SPIDER |
| Y.N(sub G)HENIYEEDVVIKRVPGASSS.A | 25.12 | 2342.15 | 21 | 2.7 | 781.7261 | 3 | 68.5 | 72 | 92 |  | SPIDER |
| A.AAAAAAAASSGARSAGGHPL.L | 25.01 | 1663.839 | 20 | 2.5 | 555.6216 | 3 | 51.7 | 2768 | 2787 |  | PEAKS DB |
| A.AAGSGGRGSGDGY.G | 25 | 1110.469 | 13 | 3.6 | 556.2438 | 2 | 33.3 | 2218 | 2230 |  | PEAKS DB |
| A.AAAAAAAAAAGSGGRGSGGGY.G | 24.98 | 1620.76 | 21 | 2.8 | 541.2623 | 3 | 49.5 | 1132 | 1152 |  | PEAKS DB |
| Y.N(sub G)SGSSGAAAAAAAAAAAARRAGH.D | 24.96 | 1936.958 | 23 | 2.8 | 646.6616 | 3 | 64.7 | 180 | 202 |  | SPIDER |
| A.KNIRHHDEY(+79.96)VDSNGQLF.E | 24.82 | 2150.944 | 17 | 2.7 | 717.9905 | 3 | 65.7 | 19 | 35 | Sulfation | PEAKS PTM |
| A.AAAAGGSGGRGSGDGY.G | 24.67 | 1309.565 | 16 | 2.5 | 655.7913 | 2 | 35.7 | 855 | 870 |  | PEAKS DB |
| Y.GSDSAAAAAAAAAAAAGSGAGGY(+125.90).G | 24.59 | 1891.683 | 23 | 3.7 | 946.8524 | 2 | 81.5 | 967 | 989 | Iodination | PEAKS PTM |
| C.FHGHSY.E | 24.54 | 746.3136 | 6 | 3 | 374.1652 | 2 | 36.1 | 2796 | 2801 |  | PEAKS DB |
| A.A(+87.03)AAAAAAGSGGRGSGGGY.G | 24.49 | 1494.681 | 18 | 4 | 748.3508 | 2 | 43 | 1135 | 1152 | Glycidamide adduct | PEAKS PTM |
| Y.GWGDEGYGSGSAAA.A | 24.43 | 1283.505 | 14 | 3.1 | 642.762 | 2 | 58.3 | 267 | 280 |  | PEAKS DB |
| Y.GSDSAAAAAAAAAAAAGSGAGGY(+37.95).G | 24.32 | 1803.734 | 23 | 3.7 | 602.254 | 3 | 74.2 | 967 | 989 | Replacement of 2 protons by calcium | PEAKS PTM |
| A.AAAAAAGGSGAGGY.D | 24.21 | 1050.473 | 14 | 2.4 | 526.2451 | 2 | 42 | 1258 | 1271 |  | PEAKS DB |
| G.S(+114.04)GSSGAAAAAAAAAAAAAR.R | 24.06 | 1586.776 | 19 | 2.2 | 794.397 | 2 | 67.5 | 907 | 925 | Ubiquitin | PEAKS PTM |
| Y.GSGSSGAAAAAAAAAAA.A | 24.05 | 1231.579 | 17 | 2.9 | 616.7986 | 2 | 57.1 | 180 | 196 |  | PEAKS DB |
| A.AASSASAGGRGGF.Y | 24.04 | 1094.511 | 13 | 2.1 | 548.2637 | 2 | 44 | 95 | 107 |  | PEAKS DB |
| A.AAAAAAAAAGGSGGRGSGDGY.G | 24.04 | 1664.75 | 21 | 3.8 | 833.3856 | 2 | 45.6 | 850 | 870 |  | PEAKS DB |
| Y.VDSN(-17.03)GQLF.E | 24.04 | 861.3868 | 8 | 1.7 | 431.7014 | 2 | 67 | 28 | 35 | Ammonia-loss (N) | PEAKS PTM |
| Y.GW(+3.99)GDEGYGSGSAAAAAAAAAAAAAGSGAGGSGGGY.G | 24.03 | 2805.186 | 35 | 4.5 | 936.0735 | 3 | 83.3 | 392 | 426 | Tryptophan oxidation to kynurenin | PEAKS PTM |
| A.AAGSGGRGSGGGY.G | 24.02 | 1052.464 | 13 | 3.3 | 527.2408 | 2 | 32.1 | 1140 | 1152 |  | PEAKS DB |
| A.AAAAAAAGGSGGRGSGDGY.G | 23.95 | 1522.676 | 19 | 3.6 | 762.348 | 2 | 40.6 | 852 | 870 |  | PEAKS DB |
| F.ERN(+.98)AETRPNLSGNERLVET.I | 23.89 | 2185.072 | 19 | 2.5 | 547.2767 | 4 | 59.9 | 45 | 63 | Deamidation (NQ) | PEAKS DB |
| Y.GSGSSGAAAAAAAAAAAAAARRAGH(+15.99).D | 23.88 | 2038.005 | 25 | 2.9 | 510.51 | 4 | 66.3 | 302 | 326 | Oxidation (HW) | PEAKS PTM |
| Y.DRQ(sub A)HGAGSAAAAAAAAAAGA(sub P)GATRPVGVY.G | 23.68 | 2565.28 | 29 | 2.8 | 642.329 | 4 | 72.7 | 2718 | 2746 |  | SPIDER |
| A.AAAAAAGSGGRGSGDGY.G | 23.67 | 1394.617 | 17 | 2.8 | 698.3179 | 2 | 38.5 | 2214 | 2230 |  | PEAKS DB |
| G.S(+114.04)GSSAAAAAAAAAAAARRAGY.D | 23.57 | 1905.94 | 21 | 3.1 | 636.3227 | 3 | 75.3 | 2697 | 2717 | Ubiquitin | PEAKS PTM |
| Y.GHENIYEED.V | 23.44 | 1104.436 | 9 | 2.5 | 553.2266 | 2 | 45.9 | 72 | 80 |  | PEAKS DB |
| A.AAAAAAAAGGSGGRGSGDGY.G | 23.43 | 1593.713 | 20 | 4 | 797.8671 | 2 | 42.9 | 851 | 870 |  | PEAKS DB |
| G.WGDGGYGS(+136.03)DSAAAAAAAAAAAAAGSGGRGSGGGY.G | 23.28 | 2951.247 | 34 | 1.7 | 984.758 | 3 | 83.5 | 1119 | 1152 | O-Diethylphosphorylation | PEAKS PTM |
| H.DRQ(sub A)AGSAAAAAAAAAAAAAGSGAGGHGGGY.G | 23.24 | 2413.112 | 30 | 3.8 | 805.3809 | 3 | 75.7 | 203 | 232 |  | SPIDER |
| Y.N(sub G)SGSSAAAAAAAAAAAARRAGY.D | 22.94 | 1905.94 | 22 | 3.8 | 953.9811 | 2 | 75.4 | 2696 | 2717 |  | SPIDER |
| Y.GSGSSGAAAAAAAAAAAA.R | 22.9 | 1302.616 | 18 | 2.8 | 652.3173 | 2 | 63.6 | 180 | 197 |  | PEAKS DB |
| Y.GW(+3.99)GDGGYGSDSAAAAAAAAAAAAAGSGAGGSGGGY.G | 22.82 | 2791.17 | 35 | 3.7 | 931.4009 | 3 | 83.5 | 517 | 551 | Tryptophan oxidation to kynurenin | PEAKS PTM |
| Y.GSGSSGAAAAAAAAAAA(+14.02).A | 22.5 | 1245.595 | 17 | 4.2 | 623.8073 | 2 | 54 | 180 | 196 | Methylation(C-term) | PEAKS PTM |
| Y.GHENIY(+125.90).E | 22.49 | 857.2205 | 6 | 3.1 | 429.6188 | 2 | 60.9 | 72 | 77 | Iodination | PEAKS PTM |
| Y.GSDSAAAAAAAAAAAAGSGGRGSGD(+37.95)GY.G | 22.48 | 2204.899 | 27 | 3.3 | 552.2339 | 4 | 69.8 | 2204 | 2230 | Replacement of 2 protons by calcium | PEAKS PTM |
| T.AKNV(sub I)RHHDEYVDSNGQLF.E | 22.41 | 2128.009 | 18 | 2 | 710.3448 | 3 | 64.6 | 18 | 35 |  | SPIDER |
| Y.GSSSSSSAAAAAGSGAGGRGGG.Y | 22.38 | 1665.73 | 22 | 2.9 | 833.8748 | 2 | 36.6 | 121 | 142 |  | PEAKS DB |
| G.S(+114.04)GSSGAAAAAAAAAAAAAAR.R | 22.35 | 1657.813 | 20 | 3.2 | 829.9164 | 2 | 71.1 | 303 | 322 | Ubiquitin | PEAKS PTM |
| Y.GSDSAAAAAAAAAAAAAGSGAGGSGGGY(+37.95).G | 22.32 | 2132.867 | 28 | 3.4 | 711.9655 | 3 | 75.3 | 524 | 551 | Replacement of 2 protons by calcium | PEAKS PTM |
| Y.GF(sub S)SSSSSAAAAAGSGAGGRGGGY.G | 22.22 | 1888.83 | 23 | 1.7 | 945.4239 | 2 | 47.7 | 121 | 143 |  | SPIDER |
| T.AKNV(sub I)RHHDEYVDSN(+.98)GQLF.E | 22.17 | 2128.993 | 18 | 3 | 533.257 | 4 | 65.3 | 18 | 35 | Deamidation (NQ) | SPIDER |
| Y.GWGDEGYGSGSAAAAAA.A | 22.16 | 1496.617 | 17 | 1.5 | 749.3167 | 2 | 61.8 | 267 | 283 |  | PEAKS DB |
| A.AAAAAAGSGGRGSGGGY.G | 22.1 | 1336.612 | 17 | 3.7 | 669.3157 | 2 | 37.8 | 1136 | 1152 |  | PEAKS DB |
| Y.GSGSSGAAAAAAAAAAAA(+14.02).R | 22.05 | 1316.632 | 18 | 2.9 | 659.3252 | 2 | 59.4 | 180 | 197 | Methylation(C-term) | PEAKS PTM |
| A.AAAAAAAAAGSGGRGSGGGY.G | 21.85 | 1549.723 | 20 | 1.8 | 517.5826 | 3 | 45.4 | 1133 | 1152 |  | PEAKS DB |
| F.ERNAETRPNLSG.N | 21.82 | 1342.659 | 12 | 3.2 | 448.5617 | 3 | 42.9 | 45 | 56 |  | PEAKS DB |
| Y.GSGSAAAAAAAAGSGAGGSGGGY(+37.95).G | 21.67 | 1719.676 | 23 | 3.4 | 574.2346 | 3 | 53.7 | 1674 | 1696 | Replacement of 2 protons by calcium | PEAKS PTM |
| Y.GSS(-18.01)SSSSAAAAAGSGAGGRGGGY.G | 21.57 | 1810.783 | 23 | 4.6 | 906.4029 | 2 | 46.7 | 121 | 143 | Dehydration | PEAKS PTM |
| G.S(+114.04)GSSGAAAAAAAAAAAAR.R | 21.55 | 1515.739 | 18 | 2.9 | 758.8789 | 2 | 63 | 181 | 198 | Ubiquitin | PEAKS PTM |
| Y.GSGSSGAAAAAAAAA.A | 21.49 | 1089.505 | 15 | 3.7 | 545.7618 | 2 | 47.2 | 180 | 194 |  | PEAKS DB |
| F.ERNAETRPNLSGN(-18.01).E | 21.48 | 1438.691 | 13 | 4.6 | 480.5732 | 3 | 42.6 | 45 | 57 | Dehydration | PEAKS PTM |
| A.AAAAAAGGSGGRGSGDGY.G | 21.25 | 1451.639 | 18 | 2.5 | 726.8286 | 2 | 38.6 | 853 | 870 |  | PEAKS DB |
| G.S(+114.04)GSAAAAAAAAAAAGSGAGGSGDGY.G | 21.01 | 2009.867 | 25 | 5.5 | 1005.947 | 2 | 65.9 | 2630 | 2654 | Ubiquitin | PEAKS PTM |
| P.G(+71.04)ATRPVGVY.G | 21 | 989.5294 | 9 | 2.6 | 495.7733 | 2 | 52 | 2738 | 2746 | Propionamide (K, X@N-term) | PEAKS PTM |
| Y.GSGSSGAAAAAAAA.A | 20.97 | 1018.468 | 14 | 3.3 | 510.2429 | 2 | 41.7 | 180 | 193 |  | PEAKS DB |
| A.AAAAAGSGAGGSDGGY.G | 20.81 | 1238.516 | 16 | 2.7 | 620.2672 | 2 | 40.7 | 2478 | 2493 |  | PEAKS DB |
| F.ERN(+.98)AETRPNLSGNER.L | 20.74 | 1742.83 | 15 | 3.8 | 436.7163 | 4 | 42.7 | 45 | 59 | Deamidation (NQ) | PEAKS DB |
| Y.N(sub G)HENIYEEDVVI.K | 20.7 | 1472.678 | 12 | 3.6 | 737.3491 | 2 | 76.8 | 72 | 83 |  | SPIDER |
| A.AAAAAAAARRAGY.D | 20.65 | 1189.632 | 13 | 1.6 | 397.5518 | 3 | 45.4 | 2705 | 2717 |  | PEAKS DB |
| Y.GHENIYEEDV(-.98).V | 20.53 | 1202.52 | 10 | 3 | 602.2693 | 2 | 55.2 | 72 | 81 | Amidation | PEAKS PTM |
| A.Q(sub A)AAGA(sub P)GATRPVGVY.G | 20.28 | 1316.684 | 14 | 3.4 | 659.3513 | 2 | 55.1 | 2733 | 2746 |  | SPIDER |
| A.AAAGGSGGRGSGDGY.G | 20.25 | 1238.528 | 15 | 4.8 | 620.274 | 2 | 34.5 | 856 | 870 |  | PEAKS DB |
| F.ERNAETRPNLSGNE(+14.02).R | 20.14 | 1599.76 | 14 | 0.5 | 534.2609 | 3 | 48.9 | 45 | 58 | Methylation(others) | PEAKS PTM |
| Y.GSDSAAAAAAAAAAAAAGSGAGGSGGGY(+123.01).G | 20.13 | 2217.929 | 28 | 3.8 | 555.4916 | 4 | 73.6 | 524 | 551 | glycosylphosphatidylinositol | PEAKS PTM |
| Y.DRAHGAGSAAAAAAA.A | 20.11 | 1266.606 | 15 | 4.4 | 423.2113 | 3 | 38.1 | 2718 | 2732 |  | PEAKS DB |
| G.SGS(+114.04)AAAAAAAAAAAGSGAGGSGDGY.G | 19.97 | 2009.867 | 25 | 4.1 | 1005.945 | 2 | 65.8 | 2630 | 2654 | Ubiquitin | PEAKS PTM |
| G.S(+114.04)GSSGAAAAAAAAAAAAAARRAGH.D | 19.9 | 2079.032 | 24 | 3.5 | 694.0203 | 3 | 69.4 | 303 | 326 | Ubiquitin | PEAKS PTM |
| A.ASSASAGGRGGFY.E | 19.89 | 1186.537 | 13 | 5.4 | 594.2788 | 2 | 51.9 | 96 | 108 |  | PEAKS DB |
| A.AAGGSGGRGSGDGY.G | 19.81 | 1167.491 | 14 | 4.2 | 584.7549 | 2 | 33.5 | 857 | 870 |  | PEAKS DB |
| Y.GSSSSSSAAAAAGS(-18.01)GAGGRGGGY.G | 19.76 | 1810.783 | 23 | 3.6 | 906.402 | 2 | 46.3 | 121 | 143 | Dehydration | PEAKS PTM |
| F.ERH(sub N)S(sub A)ETRPNLSGNERL.V | 19.65 | 1893.94 | 16 | 2.8 | 632.3225 | 3 | 57.8 | 45 | 60 |  | SPIDER |
| F.E(-18.01)RNAETRPN.L | 19.64 | 1067.511 | 9 | 3.6 | 534.7646 | 2 | 32.1 | 45 | 53 | Pyro-glu from E | PEAKS PTM |
| Y.GSDSAAAAAAAAAAAAAGSGGRGSGGGY(+37.95).G | 19.53 | 2217.931 | 28 | 3.4 | 555.4919 | 4 | 73.7 | 1125 | 1152 | Replacement of 2 protons by calcium | PEAKS PTM |
| Y.G(+27.99)SSSSSSAAAAAGSGAGGRGGGY.G | 19.51 | 1856.789 | 23 | 3.1 | 929.4044 | 2 | 46.4 | 121 | 143 | Formylation | PEAKS PTM |
| Y.N(sub G)HENIY.E | 19.49 | 788.3453 | 6 | 2.6 | 395.181 | 2 | 43.8 | 72 | 77 |  | SPIDER |
| F.E(+43.01)RNAETRPN.L | 19.49 | 1128.527 | 9 | 3.4 | 565.2728 | 2 | 32.9 | 45 | 53 | Carbamylation | PEAKS PTM |
| Y.DRAHGAGSAAAAAAAA.A | 19.28 | 1337.644 | 16 | 3.8 | 446.8901 | 3 | 43.2 | 2718 | 2733 |  | PEAKS DB |
| F.ERH(sub N)S(sub A)ETRPNLSGNERLVE.T | 19.21 | 2122.052 | 18 | 2.7 | 708.3597 | 3 | 60.6 | 45 | 62 |  | SPIDER |
| Y.GSSSSSSAAAAAGSGAGGRG(-.98).G | 19.2 | 1550.703 | 20 | 3.8 | 776.3619 | 2 | 35.7 | 121 | 140 | Amidation | PEAKS PTM |
| Y.GSSSSSSAAAAAGSGAGGR(-.98).G | 19.07 | 1493.682 | 19 | 3.4 | 747.8507 | 2 | 35.7 | 121 | 139 | Amidation | PEAKS PTM |
| F.ERNAETRPNLSGNERLVET(-18.01)IVL.E | 18.97 | 2491.314 | 22 | 2.3 | 623.8373 | 4 | 85.9 | 45 | 66 | Dehydration | PEAKS PTM |
| F.ERNAETR(+39.99)PNLSGNERLVETIVL.E | 18.95 | 2549.32 | 22 | 1 | 850.7814 | 3 | 93.2 | 45 | 66 | Glyoxal-derived hydroimiadazolone | PEAKS PTM |
| D.GGSGSGSAAAAAAAAAAAA.G | 18.81 | 1359.638 | 19 | 2.9 | 680.8282 | 2 | 63.5 | 1475 | 1493 |  | PEAKS DB |
| D.GGSGSGSAAAAAAAAAA.A | 18.78 | 1217.564 | 17 | 3.7 | 609.7913 | 2 | 51.3 | 1475 | 1491 |  | PEAKS DB |
| P.GATRPVGVY.G | 18.7 | 918.4923 | 9 | 1.7 | 460.2542 | 2 | 51.8 | 2738 | 2746 |  | PEAKS DB |
| G.SSSSSS(+136.03)AAAAAGSGAGGRGGGY.G | 18.61 | 1907.801 | 22 | -1.7 | 636.9399 | 3 | 44.7 | 122 | 143 | O-Diethylphosphorylation | PEAKS PTM |
| G.SGAGGHGGGY.G | 18.52 | 818.3307 | 10 | 4.1 | 410.1743 | 2 | 31.2 | 223 | 232 |  | PEAKS DB |
| A.AAAGSGAGGAGDGGY.G | 18.5 | 1137.469 | 15 | 3.1 | 569.7433 | 2 | 40.5 | 165 | 179 |  | PEAKS DB |
| A.AAAASSGARSAGGHPL.L | 18.43 | 1379.691 | 16 | 2.9 | 460.9055 | 3 | 42.5 | 2772 | 2787 |  | PEAKS DB |
| A.AAAAAGGSGAGGY.D | 18.42 | 979.4359 | 13 | 1.8 | 490.7261 | 2 | 40.2 | 1259 | 1271 |  | PEAKS DB |
| Y.GSSSSSSAAAAAGSGAGGRGGGY(+21.98).G | 18.23 | 1850.776 | 23 | 3.2 | 617.9344 | 3 | 45.9 | 121 | 143 | Sodium adduct | PEAKS PTM |
| G.S(+114.04)GSSGAAAAAAAAAAAAARRAGH.D | 18.17 | 2007.995 | 23 | 3 | 670.3408 | 3 | 68 | 907 | 929 | Ubiquitin | PEAKS PTM |
| F.ERNAETRPNLSGNERLVET(-18.01)IV.L | 18.11 | 2378.23 | 21 | 3.5 | 595.5669 | 4 | 85.7 | 45 | 65 | Dehydration | PEAKS PTM |
| P.G(sub N)LSGNERLVETIVL.E | 18.03 | 1498.835 | 14 | 1.9 | 750.4265 | 2 | 89.1 | 53 | 66 |  | SPIDER |
| A.AAAAAAAAGSGAGGY(+21.98).G | 17.95 | 1157.508 | 15 | 2.8 | 579.7628 | 2 | 47 | 498 | 512 | Sodium adduct | PEAKS PTM |
| F.ERH(sub N)S(sub A)ETRPNLSGNERLVETI.V | 17.93 | 2336.183 | 20 | 3 | 779.7374 | 3 | 73.4 | 45 | 64 |  | SPIDER |
| F.ERNAETRPNLS(-.98).G | 17.79 | 1284.653 | 11 | 3.3 | 643.3361 | 2 | 41.1 | 45 | 55 | Amidation | PEAKS PTM |
| Y.DRAHGAGSAAAAAA.A | 17.71 | 1195.569 | 14 | 2.6 | 399.5314 | 3 | 36.1 | 2718 | 2731 |  | PEAKS DB |
| F.ERH(sub N)S(sub A)ETRPNL.S | 17.63 | 1237.616 | 10 | 2.6 | 619.8171 | 2 | 50.2 | 45 | 54 |  | SPIDER |
| A.AAAAAARRAGY.D | 17.56 | 1047.557 | 11 | 4.9 | 350.1948 | 3 | 37.2 | 2707 | 2717 |  | PEAKS DB |
| Y.GW(+3.99)GDGGYGSGSAAAAAAAAGSGAGGSGGGY.G | 17.46 | 2377.98 | 30 | 5.1 | 1190.003 | 2 | 67.9 | 1667 | 1696 | Tryptophan oxidation to kynurenin | PEAKS PTM |
| A.AAAAAAGSGAGGAGD(+21.98)GGY.G | 17.39 | 1372.562 | 18 | 1.8 | 687.2894 | 2 | 44.1 | 162 | 179 | Sodium adduct | PEAKS PTM |
| G.SSSSS(+136.03)SAAAAAGSGAGGRGGGY.G | 17.38 | 1907.801 | 22 | -1.1 | 636.9402 | 3 | 44.8 | 122 | 143 | O-Diethylphosphorylation | PEAKS PTM |
| A.KNIRHHDEYVDS(+79.97)N(+.98)GQLF.E | 17.36 | 2151.938 | 17 | -3.2 | 538.9899 | 4 | 67.2 | 19 | 35 | Phosphorylation (STY); Deamidation (NQ) | PEAKS PTM |
| D.GGSGSGSAAAAAAAAAAA.A | 17.35 | 1288.601 | 18 | 2.2 | 645.309 | 2 | 57.2 | 1475 | 1492 |  | PEAKS DB |
| Y.GSDSAAAAAAAAAAAAGSGAGGY(+17.03).G | 17.25 | 1782.813 | 23 | 3.9 | 892.4174 | 2 | 74.1 | 967 | 989 | Replacement of proton with ammonium ion | PEAKS PTM |
| Y.GWGDGGYGSDSAAAAAAAAAAAAGSGAGGY(+17.03).G | 17.22 | 2475.069 | 30 | 3.1 | 1238.545 | 2 | 84.7 | 960 | 989 | Replacement of proton with ammonium ion | PEAKS PTM |
| Y.GWGDEGYGSG.S | 17.21 | 983.3621 | 10 | 2.9 | 492.6897 | 2 | 55.4 | 267 | 276 |  | PEAKS DB |
| F.ERNAETRPNLSGNE(-.98).R | 17.11 | 1584.76 | 14 | 3.1 | 529.2624 | 3 | 42.7 | 45 | 58 | Amidation | PEAKS PTM |
| D.GGSGSGSAAAAAAAAA.A | 17.04 | 1146.527 | 16 | 3.6 | 574.2726 | 2 | 47.2 | 1475 | 1490 |  | PEAKS DB |
| Y.GSDSAAAAAAAAAAAAAGS(-18.01)GAGGSGGGY.G | 16.93 | 2076.91 | 28 | -0.9 | 1039.461 | 2 | 76.9 | 524 | 551 | Dehydration | PEAKS PTM |
| L.LSM(sub I)CCR(sub K)PCFHGHSY.E | 16.85 | 1639.652 | 14 | 5.4 | 547.5608 | 3 | 67.3 | 2788 | 2801 |  | SPIDER |
| Y.GSSSSSSAAAAAGSGAGGRGG(-.98).G | 16.83 | 1607.725 | 21 | 4.8 | 804.8735 | 2 | 35.7 | 121 | 141 | Amidation | PEAKS PTM |
| Y.W(sub E)SHDSFVVDSSY.G | 16.51 | 1427.599 | 12 | -8.7 | 714.8007 | 2 | 64.3 | 109 | 120 |  | SPIDER |
| **CHYMOTRYPSIN-TRYPSIN** |  |  |  |  |  |  |  |  |  |  |  |
| **Peptide** | **-10lgP** | **Mass** | **Length** | **ppm** | **m/z** | **z** | **RT** | **Start** | **End** | **PTM** | **Found By** |
| R.AGHDRAAGSAAAAAAAAAAAAAGSGAGGHGGGY.G | 88.81 | 2621.208 | 33 | 4.4 | 874.747 | 3 | 72.2 | 200 | 232 |  | PEAKS DB |
| Y.GWGDGGYGSDSAAAAAAAAAAAAAGGSGAGGHGGGY.G | 87.64 | 2894.224 | 36 | 3.4 | 965.7518 | 3 | 84.1 | 799 | 834 |  | PEAKS DB |
| Y.DSEGSAAAAAAAAAAAASSGARSAGGHPL.L | 83.65 | 2423.142 | 29 | 3.5 | 808.7242 | 3 | 79.2 | 2759 | 2787 |  | PEAKS DB |
| R.AGHDRAAGSAAAAAAAAAAAAAGSGAGGYGGGY.G | 77.4 | 2647.212 | 33 | 4.3 | 883.4151 | 3 | 77 | 484 | 516 |  | PEAKS DB |
| R.RAGHDRAAGSAAAAAAAAAAAAGGSGAGGY.D | 71.42 | 2455.17 | 30 | 3.6 | 614.8019 | 4 | 68.7 | 1242 | 1271 |  | PEAKS DB |
| Y.DSEGSAAAAAAAAAAAASSGAR.S | 70.17 | 1803.835 | 22 | 4 | 902.9282 | 2 | 71.5 | 2759 | 2780 |  | PEAKS DB |
| Y.GSGSSGAAAAAAAAAAAAAR.R | 69.06 | 1529.755 | 20 | 3.3 | 765.8871 | 2 | 67.3 | 906 | 925 |  | PEAKS DB |
| R.AAGSAAAAAAAAAAAAAGSGAGGHGGGY.G | 68.52 | 2084.962 | 28 | 3.9 | 1043.493 | 2 | 75 | 205 | 232 |  | PEAKS DB |
| Y.GSGSSGAAAAAAAAAAAAAAR.R | 68.36 | 1600.792 | 21 | 3.1 | 801.4056 | 2 | 70.8 | 302 | 322 |  | PEAKS DB |
| R.RAGHDRAAGSAAAAAAAAAAAAAGSGAGGHGGGY.G | 67.9 | 2777.309 | 34 | 3.5 | 695.3369 | 4 | 70.8 | 199 | 232 |  | PEAKS DB |
| L.DGGYDSEGSAAAAAAAAAAAASSGARSAGGHPL.L | 67.27 | 2815.276 | 33 | 2.5 | 939.4348 | 3 | 82.1 | 2755 | 2787 |  | PEAKS DB |
| Y.GSDSAAAAAAAAAAAAGSGGRGSGDGY.G | 66.75 | 2166.953 | 27 | 3.7 | 1084.488 | 2 | 69.6 | 2204 | 2230 |  | PEAKS DB |
| Y.GSDSAAAAAAAAAAAAGSGGR.G | 66.34 | 1630.766 | 21 | 3.2 | 544.5977 | 3 | 66.1 | 2204 | 2224 |  | PEAKS DB |
| Y.GHENIYEEDVVIK.R | 66.24 | 1543.752 | 13 | 3.7 | 772.886 | 2 | 66.3 | 72 | 84 |  | PEAKS DB |
| Y.GWGDDGYGSGSAAAAAAAAAAAAAGSGAGGAGDGGY.G | 65.68 | 2886.208 | 36 | 2.3 | 963.0787 | 3 | 85.8 | 710 | 745 |  | PEAKS DB |
| Y.GWGDEGYGSGSAAAAAAAAAAAAGSGAGGAGDGGY.G | 65.44 | 2829.186 | 35 | 3.1 | 944.0722 | 3 | 82.2 | 267 | 301 |  | PEAKS DB |
| F.VLDGGYDSEGSAAAAAAAAAAAASSGAR.S | 64.94 | 2408.12 | 28 | 3.6 | 1205.072 | 2 | 79.1 | 2753 | 2780 |  | PEAKS DB |
| Y.GSGSSGAAAAAAAAAAAAAAAR.R | 64.76 | 1671.829 | 22 | 3.6 | 836.9247 | 2 | 75.4 | 1542 | 1563 |  | PEAKS DB |
| R.RAGHDRAAGSAAAAAAAAAAAAAGSGAGGY.G | 64.56 | 2469.186 | 30 | 3.6 | 618.3059 | 4 | 73.9 | 483 | 512 |  | PEAKS DB |
| R.AAGSAAAAAAAAAAAAAGSGAGGH.G | 62.67 | 1750.835 | 24 | 3.7 | 876.4278 | 2 | 71.2 | 205 | 228 |  | PEAKS DB |
| Y.GWGDGGYGSDSAAAAAAAAAAAAAGSGGR.G | 62.59 | 2394.058 | 29 | 3.6 | 1198.041 | 2 | 82.7 | 1118 | 1146 |  | PEAKS DB |
| S.GSGSAAAAAAAAAAAAGSGAGGAGGGY.G | 62.44 | 1949.883 | 27 | 3.7 | 975.9522 | 2 | 71.1 | 1478 | 1504 |  | PEAKS DB |
| K.RVPGASSSAAAASSASAGGRGGF.Y | 62.4 | 1977.962 | 23 | 4.5 | 660.3307 | 3 | 54.4 | 85 | 107 |  | PEAKS DB |
| H.DRAAGSAAAAAAAAAAAAGGSGAGGY.D | 62.12 | 2033.951 | 26 | 3.5 | 1017.987 | 2 | 72.2 | 1246 | 1271 |  | PEAKS DB |
| Y.G(+43.01)HENIYEEDVVIK.R | 62.11 | 1586.758 | 13 | 3.4 | 794.3887 | 2 | 70.7 | 72 | 84 | Carbamylation | PEAKS PTM |
| R.AAGSAAAAAAAAAAAAAGSGSGGY.G | 61.57 | 1792.834 | 24 | 3.8 | 897.4276 | 2 | 76.5 | 329 | 352 |  | PEAKS DB |
| Y.GWGDEGYGSGSAAAAAAAAAAAAAGSGAGGSGGGY.G | 61.35 | 2801.191 | 35 | 3.7 | 934.7411 | 3 | 85.2 | 392 | 426 |  | PEAKS DB |
| L.EEDPYGHENIYEEDVVIK.R | 61.24 | 2176.98 | 18 | 3.6 | 726.6699 | 3 | 73 | 67 | 84 |  | PEAKS DB |
| Y.GHENIYEEDVVIKR.V | 60.66 | 1699.853 | 14 | 2.9 | 567.6265 | 3 | 61.9 | 72 | 85 |  | PEAKS DB |
| W.GDGGYGSDSAAAAAAAAAAAAGSGAGGY.G | 60.51 | 2214.941 | 28 | 3.6 | 1108.482 | 2 | 78.5 | 962 | 989 |  | PEAKS DB |
| H.DRAAGSAAAAAAAAAAAAAGSGAGGY.G | 60.29 | 2047.967 | 26 | 4 | 1024.995 | 2 | 77.7 | 487 | 512 |  | PEAKS DB |
| R.AAGSAAAAAAAAAAAAAGSGAGGHGGGYG.W | 60.26 | 2141.984 | 29 | 4.4 | 1072.004 | 2 | 74.8 | 205 | 233 |  | PEAKS DB |
| F.VLDGGYDSEGSAAAAAAAAAAAASSGARSAGGHPL.L | 60.2 | 3027.428 | 35 | 3.8 | 1010.154 | 3 | 84.5 | 2753 | 2787 |  | SPIDER |
| Y.GSGSAAAAAAAAAAAAAGSGAGGSGGGY.G | 60.14 | 2036.915 | 28 | 3.8 | 679.9814 | 3 | 74.2 | 399 | 426 |  | PEAKS DB |
| R.AGHDRAAGSAAAAAAAAAAAAAGSGAGGY.G | 60.02 | 2313.085 | 29 | 3.1 | 772.0378 | 3 | 75.4 | 484 | 512 |  | PEAKS DB |
| R.AAGSAAAAAAAAAAAAGGSGAGGHGGGYG.W | 59.94 | 2127.968 | 29 | 3.6 | 1064.995 | 2 | 69.3 | 771 | 799 |  | PEAKS DB |
| Y.GSDSAAAAAAAAAAAAAGSGGR.G | 59.77 | 1701.803 | 22 | 3.4 | 568.2769 | 3 | 70.7 | 1125 | 1146 |  | PEAKS DB |
| Y.G(+43.01)HENIYEEDVVIKR.V | 59.72 | 1742.859 | 14 | 3.9 | 581.9625 | 3 | 65.9 | 72 | 85 | Carbamylation | PEAKS PTM |
| Y.GSDSAAAAAAAAAAAAAGSGGRGSGGGY.G | 59.68 | 2179.984 | 28 | 3.7 | 1091.003 | 2 | 73.6 | 1125 | 1152 |  | PEAKS DB |
| R.AAGSAAAAAAAAAAAAGGSGAGGHGGGY.G | 59.56 | 2070.947 | 28 | 3.7 | 1036.484 | 2 | 69.7 | 771 | 798 |  | PEAKS DB |
| R.VPGASSSAAAASSASAGGRGGFY.E | 59.48 | 1984.924 | 23 | 3.1 | 993.4723 | 2 | 61.6 | 86 | 108 |  | PEAKS DB |
| Y.GWGDGGYGSGSAAAAAAAAGSGAGGSGGGY.G | 59.43 | 2373.985 | 30 | 3.6 | 792.3383 | 3 | 70.1 | 1667 | 1696 |  | PEAKS DB |
| Y.GSGSAAAAAAAAAAAAGSGAGGAGDGGY.G | 59.33 | 2064.91 | 28 | 4.2 | 1033.466 | 2 | 70.1 | 274 | 301 |  | PEAKS DB |
| F.VLDGGYDSE(+21.98)GSAAAAAAAAAAAASSGAR.S | 59.11 | 2430.102 | 28 | 3.6 | 811.0443 | 3 | 79.7 | 2753 | 2780 | Sodium adduct | PEAKS PTM |
| W.GDGGYGSDSAAAAAAAAAAAAAGSGGRGSGGGY.G | 58.23 | 2629.139 | 33 | 4.4 | 877.3907 | 3 | 78 | 1120 | 1152 |  | PEAKS DB |
| R.AAGSAAAAAAAAAAAAGGSGAGGYDGGY.G | 57.75 | 2154.957 | 28 | 4.6 | 719.3294 | 3 | 73.6 | 1248 | 1275 |  | PEAKS DB |
| H.DRAAGSAAAAAAAAAAAAGGSGAGGHGGGY.G | 57.66 | 2342.075 | 30 | 3.2 | 781.7014 | 3 | 69.6 | 769 | 798 |  | PEAKS DB |
| H.DRAAGSAAAAAAAAAAAAAGSGAGGHGGGY.G | 57.32 | 2356.09 | 30 | 3.7 | 786.3737 | 3 | 74.6 | 203 | 232 |  | PEAKS DB |
| Y.DRAHGAGSAAAAAAAAAAGA(sub P)GATRPVGVY.G | 57.28 | 2508.258 | 29 | 3.5 | 837.0963 | 3 | 72.9 | 2718 | 2746 |  | SPIDER |
| Y.GSGSSGAAAAAAAAAAAAR.R | 57.11 | 1458.717 | 19 | 3.8 | 730.3688 | 2 | 62.8 | 180 | 198 |  | PEAKS DB |
| Y.GWGDGGYGSDSAAAAAAAAAAAAGSGAGGY.G | 56.92 | 2458.042 | 30 | 3 | 1230.032 | 2 | 84.9 | 960 | 989 |  | PEAKS DB |
| R.AAGSAAAAAAAAAAAAAGSGAGGHGGGYGW.G | 56.88 | 2328.063 | 30 | 2.3 | 777.0301 | 3 | 84.6 | 205 | 234 |  | PEAKS DB |
| Y.GWGDGGYGSDSAAAAAAAAAAAAAGGSGGR.G | 56.54 | 2451.08 | 30 | 3.9 | 1226.552 | 2 | 82.3 | 835 | 864 |  | PEAKS DB |
| Y.GSGSSAAAAAAAAAAAGSGAGGSDGGY.G | 56.39 | 2039.878 | 27 | 4.4 | 1020.951 | 2 | 65.4 | 2467 | 2493 |  | PEAKS DB |
| R.AGHDRAAGSAAAAAAAAAAAAAGSGSGGY.G | 56.25 | 2329.079 | 29 | 3.7 | 777.3699 | 3 | 73.8 | 324 | 352 |  | PEAKS DB |
| Y.ESHDSFVVDSSY.G | 56.21 | 1370.563 | 12 | 4.3 | 686.2915 | 2 | 62.8 | 109 | 120 |  | PEAKS DB |
| H.DRAAGSAAAAAAAAAAAAAGSGSGGY.G | 56.05 | 2063.962 | 26 | 4.2 | 1032.993 | 2 | 76.1 | 327 | 352 |  | PEAKS DB |
| Y.GSGSSGAAAAAAAAAAAAAARRAGH.D | 55.84 | 2022.01 | 25 | 2.9 | 506.5113 | 4 | 69.3 | 302 | 326 |  | PEAKS DB |
| Y.GSGSAAAAAAAAAAAAAAGSGAGGSGGGY.G | 55.55 | 2107.952 | 29 | 4.4 | 1054.988 | 2 | 77.1 | 1830 | 1858 |  | PEAKS DB |
| Y.GWGDGGYGSDSAAAAAAAAAAAAGSGAGGVGGGY.G | 55.28 | 2728.175 | 34 | 1.7 | 910.4004 | 3 | 85.7 | 2426 | 2459 |  | PEAKS DB |
| Y.GSDSAAAAAAAAAAAAAGSGAGGSGDGY.G | 55.14 | 2152.926 | 28 | 3.9 | 1077.474 | 2 | 75.6 | 1001 | 1028 |  | PEAKS DB |
| Y.GSGSSGAAAAAAAAAAAAARRAGHDR.A | 55.09 | 2222.101 | 26 | 2.8 | 445.4287 | 5 | 66.6 | 906 | 931 |  | PEAKS DB |
| R.AAGSAAAAAAAAAAAAAGSGAGGY.G | 55.04 | 1776.839 | 24 | 3.4 | 889.4298 | 2 | 78.3 | 489 | 512 |  | PEAKS DB |
| W.DYEGYGSDSAAAAAAAAAAAAGSGGR.G | 55.04 | 2257.983 | 26 | 4.4 | 753.6717 | 3 | 76.2 | 2590 | 2615 |  | PEAKS DB |
| Y.GWGDGGYGSDSAAAAAAAAAAAAAGSGAGGSGGGY.G | 54.82 | 2787.176 | 35 | 2.3 | 930.0679 | 3 | 85.4 | 517 | 551 |  | PEAKS DB |
| Y.GWGDDGSGSGSAAAAAAAAAAAAGSGAGGSGGGY.G | 54.61 | 2640.107 | 34 | 4.4 | 881.0469 | 3 | 78.2 | 233 | 266 |  | PEAKS DB |
| R.AHGAGSAAAAAAAAAAGA(sub P)GATRPVGVY.G | 54.44 | 2237.13 | 27 | 3.3 | 746.7198 | 3 | 73.1 | 2720 | 2746 |  | SPIDER |
| Y.GWGDGGSGSGSAAAAAAAAAAAAGSGAGGAGGGY.G | 54.21 | 2566.107 | 34 | 3.3 | 856.379 | 3 | 78.6 | 1471 | 1504 |  | PEAKS DB |
| Y.GWGDGGYGSDSAAAAAAAAAAAAAGSGAGGY.G | 53.95 | 2529.079 | 31 | 2.2 | 844.0355 | 3 | 87.8 | 1276 | 1306 |  | PEAKS DB |
| Y.GSDSAAAAAAAAAAAAGSGAGGVGGGY.G | 53.93 | 2035.919 | 27 | 4.2 | 1018.971 | 2 | 75.7 | 2433 | 2459 |  | PEAKS DB |
| Y.GSGSSGAAAAAAAAAAAAARRAGH.D | 53.69 | 1950.973 | 24 | 3.6 | 488.7523 | 4 | 68 | 906 | 929 |  | PEAKS DB |
| R.AAGSAAAAAAAAAAAAGGSGAGGYGGGY.G | 53.64 | 2096.951 | 28 | 2.7 | 699.9929 | 3 | 74.5 | 1570 | 1597 |  | PEAKS DB |
| Y.GSGSSGAAAAAAAAAAAAAAARR.A | 53.58 | 1827.93 | 23 | 3.1 | 610.3192 | 3 | 73.2 | 1542 | 1564 |  | PEAKS DB |
| Y.GWGDEGYGSGSAAAAAAAAAAAAAAGSGAGGSGGGY.G | 53.5 | 2872.228 | 36 | 3.7 | 958.4203 | 3 | 87.6 | 1823 | 1858 |  | PEAKS DB |
| R.HHDEYVDSN(+.98)GQLF.E | 53.41 | 1560.648 | 13 | 2.9 | 521.2248 | 3 | 70.7 | 23 | 35 | Deamidation (NQ) | PEAKS DB |
| R.AAGSAAAAAAAAAAAAGGSGAGGY.D | 53.3 | 1762.823 | 24 | 3.2 | 882.4218 | 2 | 72.5 | 1248 | 1271 |  | PEAKS DB |
| Y.GSDSAAAAAAAAAAAAAGSGAGGY.G | 53.23 | 1836.824 | 24 | 4.1 | 919.4229 | 2 | 77.8 | 1283 | 1306 |  | PEAKS DB |
| D.PYGHENIYEEDVVIK.R | 53.1 | 1803.868 | 15 | 2.5 | 602.2981 | 3 | 69.6 | 70 | 84 |  | PEAKS DB |
| Y.GSGSSGAAAAAAAAAAAAAAARRAGHDR.A | 52.86 | 2364.175 | 28 | 3.1 | 592.0529 | 4 | 71.9 | 1542 | 1569 |  | PEAKS DB |
| Y.GSGSAAAAAAAAGSGAGGSGGGY.G | 52.78 | 1681.729 | 23 | 5 | 841.876 | 2 | 51.7 | 1674 | 1696 |  | PEAKS DB |
| Y.GSGSAAAAAAAAAAAAAGSGAGGAGDGGY.G | 52.42 | 2135.947 | 29 | 3.8 | 712.9922 | 3 | 74.2 | 717 | 745 |  | PEAKS DB |
| Y.GHENIYEEDVVIKRVPGASSSAA.A | 52.35 | 2427.203 | 23 | 4.1 | 810.0782 | 3 | 69.1 | 72 | 94 |  | SPIDER |
| Y.G(+43.01)SGSSGAAAAAAAAAAAAAAR.R | 52 | 1643.798 | 21 | 3.3 | 822.9087 | 2 | 74.5 | 302 | 322 | Carbamylation | PEAKS PTM |
| Y.GHENIYEEDVVIKRVPGAS.S | 51.73 | 2111.065 | 19 | 3.7 | 704.6981 | 3 | 69 | 72 | 90 |  | SPIDER |
| Y.GHENIYEEDVVIKRVPGASSS.A | 51.67 | 2285.129 | 21 | 3.2 | 762.7193 | 3 | 68.5 | 72 | 92 |  | SPIDER |
| Y.GSGSSGAAAAAAAAAAAAAARRAGHDR.A | 51.65 | 2293.138 | 27 | 3.5 | 574.2938 | 4 | 67.6 | 302 | 328 |  | PEAKS DB |
| Y.GSGSSAAAAAAAAAAAARRAGY.D | 51.57 | 1848.919 | 22 | 3.6 | 925.47 | 2 | 75.4 | 2696 | 2717 |  | PEAKS DB |
| Y.GSDSAAAAAAAAAAAAAGSGAGGSGGGY.G | 51.46 | 2094.92 | 28 | 2.7 | 1048.47 | 2 | 75.2 | 524 | 551 |  | PEAKS DB |
| Y.GSDSAAAAAAAAAAAAAGGSGGR.G | 51.26 | 1758.825 | 23 | 3.7 | 880.4227 | 2 | 70 | 842 | 864 |  | PEAKS DB |
| Y.GHENIYEEDVVIKRVPGASSSAAAASSASAGGR.G | 51.13 | 3242.592 | 33 | 3.8 | 811.6583 | 4 | 67.6 | 72 | 104 |  | SPIDER |
| Y.GSDDGFVLDGGYDSEGSAAAAAAAAAAAASSGAR.S | 50.88 | 2986.318 | 34 | 3.1 | 996.4496 | 3 | 87.9 | 2747 | 2780 |  | SPIDER |
| K.RVPGASSSAAAASSASAGGR.G | 50.27 | 1716.85 | 20 | 2.4 | 573.2921 | 3 | 43.1 | 85 | 104 |  | PEAKS DB |
| R.AAGSAAAAAAAAAAAAGGSGAGGH.G | 50.01 | 1736.819 | 24 | 3.8 | 579.9492 | 3 | 65.1 | 771 | 794 |  | PEAKS DB |
| R.NAETRPNLSGNER.L | 49.76 | 1456.702 | 13 | 2.9 | 486.5759 | 3 | 42.7 | 47 | 59 |  | PEAKS DB |
| Y.GSDSAAAAAAAAAAAAAGSGAGGYGDGY.G | 49.63 | 2228.957 | 28 | 4.3 | 1115.491 | 2 | 79.4 | 1443 | 1470 |  | PEAKS DB |
| R.VPGASSSAAAASSASAGGRGGFYESHDSF.V | 49.51 | 2687.185 | 29 | 3.9 | 896.739 | 3 | 67.1 | 86 | 114 |  | SPIDER |
| S.AAAAAAAAAAAAAGSGAGGHGGGY.G | 49.51 | 1798.835 | 24 | 3.5 | 600.6209 | 3 | 63.6 | 209 | 232 |  | PEAKS DB |
| Y.G(+43.01)SGSSGAAAAAAAAAAAAAR.R | 49.44 | 1572.76 | 20 | 3.6 | 787.3903 | 2 | 71.1 | 906 | 925 | Carbamylation | PEAKS PTM |
| Y.GWGDDGYGSGSAAAAAAAAAAAAGSGAGGAGGGY.G | 49.21 | 2700.144 | 34 | 3.2 | 901.058 | 3 | 83 | 2655 | 2688 |  | PEAKS DB |
| Y.GWGDGGYGSD(+21.98)SAAAAAAAAAAAAAGSGGR.G | 49.09 | 2416.04 | 29 | 2.7 | 806.3562 | 3 | 82.9 | 1118 | 1146 | Sodium adduct | PEAKS PTM |
| W.GDGGYGSDSAAAAAAAAAAAAAGSGGR.G | 48.81 | 2150.958 | 27 | 3.5 | 717.9956 | 3 | 75.9 | 1120 | 1146 |  | PEAKS DB |
| Y.GHENIYEEDVVI.K | 48.58 | 1415.657 | 12 | 3.2 | 708.838 | 2 | 76.8 | 72 | 83 |  | PEAKS DB |
| Y.GWGDDGYGSGSAAAAAAAAAAAAAGSGAGGVGGGY.G | 48.58 | 2799.212 | 35 | 4.5 | 934.0822 | 3 | 88 | 2494 | 2528 |  | PEAKS DB |
| K.NIRHHDEYVDSN(+.98)GQLF.E | 48.52 | 1943.876 | 16 | 2.8 | 648.9678 | 3 | 69.2 | 20 | 35 | Deamidation (NQ) | SPIDER |
| R.VPGASSSAAAASSASAGGRGGF.Y | 48.34 | 1821.861 | 22 | 4.1 | 911.9413 | 2 | 56.8 | 86 | 107 |  | PEAKS DB |
| Y.GSDSAAAAAAAAAAAAAGGSGAGGHGGGY.G | 48.22 | 2201.969 | 29 | 2.8 | 734.9988 | 3 | 73.5 | 806 | 834 |  | PEAKS DB |
| Y.GHENIYEEDVVIKRVPGASSSAAAAS.S | 48.08 | 2656.309 | 26 | 4 | 886.4471 | 3 | 68.8 | 72 | 97 |  | SPIDER |
| Y.G(+43.01)SDSAAAAAAAAAAAAAGSGGR.G | 48.08 | 1744.809 | 22 | 4.3 | 873.4153 | 2 | 75.1 | 1125 | 1146 | Carbamylation | PEAKS PTM |
| F.ERNAETRPNLSGNER.L | 47.59 | 1741.846 | 15 | 3.1 | 581.6243 | 3 | 19.2 | 45 | 59 |  | PEAKS DB |
| F.YESHDSFVVDSSY.G | 47.48 | 1533.626 | 13 | 4 | 767.8233 | 2 | 66.9 | 108 | 120 |  | PEAKS DB |
| W.DYEGYGSDSAAAAAAAAAAAAGSGGRGSGDGY.G | 47.3 | 2794.17 | 32 | 4.2 | 932.4012 | 3 | 78.1 | 2590 | 2621 |  | SPIDER |
| R.AAGSAAAAAAAAAAAAGGSGAGGY(+21.98).D | 47.17 | 1784.805 | 24 | 3.1 | 595.9442 | 3 | 72.5 | 1248 | 1271 | Sodium adduct | PEAKS PTM |
| Y.GSGS(-18.01)SGAAAAAAAAAAAARR.A | 47.1 | 1596.808 | 20 | 3.6 | 533.2785 | 3 | 62 | 180 | 199 | Dehydration | PEAKS PTM |
| Y.GSGSSGAAAAAAAAAAAAAAR(+15.99).R | 47.08 | 1616.787 | 21 | 3.9 | 809.4037 | 2 | 69.4 | 302 | 322 | Oxidation or Hydroxylation | PEAKS PTM |
| R.A(+43.01)AGSAAAAAAAAAAAAAGSGAGGHGGGY.G | 46.92 | 2127.968 | 28 | 2.8 | 1064.994 | 2 | 84.5 | 205 | 232 | Carbamylation | PEAKS PTM |
| Y.GSGSSGAAAAAAAAAAAARR.A | 46.84 | 1614.819 | 20 | 3.5 | 808.4194 | 2 | 61.9 | 180 | 199 |  | PEAKS DB |
| Y.GSDSAAAAAAAAAAAAAGGSGGRGSGDGY.G | 46.76 | 2295.011 | 29 | 4.1 | 766.0141 | 3 | 73.1 | 842 | 870 |  | PEAKS DB |
| R.NAETRPNLSGNERLVETIVLEEDPY.G | 46.56 | 2857.409 | 25 | 2.6 | 953.4796 | 3 | 94.1 | 47 | 71 |  | SPIDER |
| Y.GSGSSGAAAAAAAAAAAARRAGH.D | 46.21 | 1879.936 | 23 | 3.5 | 470.993 | 4 | 64.4 | 180 | 202 |  | PEAKS DB |
| Y.GS(-15.99)GSAAAAAAAAAAAAGSGAGGAGEGGY.G | 46.14 | 2062.93 | 28 | 4.1 | 1032.477 | 2 | 73.3 | 878 | 905 | Deoxy | PEAKS PTM |
| Y.GHENIYEEDVVIKRVPGASSSAAAASSASAGGRGGFY.E | 46.09 | 3666.766 | 37 | 4.5 | 917.7029 | 4 | 74.9 | 72 | 108 |  | SPIDER |
| E.TRPNLSGNERLVETIVLEEDPY.G | 46.07 | 2543.287 | 22 | 3.2 | 848.7722 | 3 | 93.3 | 50 | 71 |  | SPIDER |
| R.LVETIVLEEDPYGHENIY.E | 45.99 | 2132.031 | 18 | 3.3 | 1067.026 | 2 | 88.2 | 60 | 77 |  | SPIDER |
| Y.GSDSAAAAAAAAAAAAAGSGAGGY(+21.98).G | 45.99 | 1858.806 | 24 | 4.5 | 930.4142 | 2 | 78 | 1283 | 1306 | Sodium adduct | PEAKS PTM |
| F.ERNAETRPNLSGNERL.V | 45.91 | 1854.93 | 16 | 3.5 | 464.7413 | 4 | 53.7 | 45 | 60 |  | SPIDER |
| Y.GSGSSGAAAAAAAAAAAAAARR.A | 45.85 | 1756.893 | 22 | 3.7 | 586.6404 | 3 | 72.2 | 302 | 323 |  | PEAKS DB |
| R.N(+.98)AETRPNLSGNERLVETIVLEEDPY.G | 45.81 | 2858.393 | 25 | 3.5 | 953.8084 | 3 | 97.6 | 47 | 71 | Deamidation (NQ) | SPIDER |
| G.SAAAAAAAAAAAAAGSGAGGHGGGY.G | 44.95 | 1885.867 | 25 | 3.3 | 629.6315 | 3 | 70.8 | 208 | 232 |  | PEAKS DB |
| Y.GHENIYE(+37.95)EDVVIK.R | 44.92 | 1581.699 | 13 | 2.5 | 528.2415 | 3 | 66.5 | 72 | 84 | Replacement of 2 protons by calcium | PEAKS PTM |
| R.LVETIVLEEDPYGHENIYEEDVVIK.R | 44.73 | 2944.459 | 25 | 4.3 | 982.4979 | 3 | 91.5 | 60 | 84 |  | SPIDER |
| R.AAGSAAAAAAAAAAAAAGSGAGGY(+21.98).G | 44.71 | 1798.821 | 24 | 2.5 | 600.6157 | 3 | 78.4 | 489 | 512 | Sodium adduct | PEAKS PTM |
| R.AAGSAAAAAAAAAAAAAGSGSGGY(+21.98).G | 44.67 | 1814.816 | 24 | 2.8 | 605.9476 | 3 | 76.5 | 329 | 352 | Sodium adduct | PEAKS PTM |
| Y.G(+43.01)SGSSGAAAAAAAAAAAAARR.A | 44.64 | 1728.862 | 21 | 3.6 | 865.4411 | 2 | 70.3 | 906 | 926 | Carbamylation | PEAKS PTM |
| G.S(+114.04)GSSGAAAAAAAAAAAAAAR.R | 44.56 | 1657.813 | 20 | 3.8 | 829.917 | 2 | 70.9 | 303 | 322 | Ubiquitin | PEAKS PTM |
| R.AGHDRAAGSAAAAAAAAAAAAGGSGAGGY.D | 44.49 | 2299.069 | 29 | 3.4 | 767.3661 | 3 | 70.3 | 1243 | 1271 |  | PEAKS DB |
| R.LVETIVLEEDPYGHENIYEEDVVIKR.V | 44.41 | 3100.56 | 26 | 1.1 | 1034.529 | 3 | 86.6 | 60 | 85 |  | SPIDER |
| N.AETRPNLSGNERLVETIVLEEDPY.G | 44.22 | 2743.367 | 24 | 3.5 | 915.466 | 3 | 94.1 | 48 | 71 |  | SPIDER |
| K.RVPGASSSAAAASSASAGGRGGFYESHDSF.V | 44.19 | 2843.286 | 30 | 3.9 | 711.8315 | 4 | 65 | 85 | 114 |  | SPIDER |
| E.N(+380.15)IYEEDVVIK.R | 44.13 | 1600.776 | 10 | 2.1 | 801.397 | 2 | 66.4 | 75 | 84 | Nucleophilic addition to cytopiloyne+H2O | PEAKS PTM |
| Y.DRQ(sub A)HGAGSAAAAAAAAAAGA(sub P)GATRPVGVY.G | 44.13 | 2565.28 | 29 | 4.2 | 856.1041 | 3 | 73.5 | 2718 | 2746 |  | SPIDER |
| Y.GHENIY.E | 44.1 | 731.3239 | 6 | 4.1 | 366.6707 | 2 | 44 | 72 | 77 |  | PEAKS DB |
| Y.GSSSSSSAAAAAGSGAGGRGGGY.G | 43.7 | 1828.794 | 23 | 6.5 | 915.41 | 2 | 44.6 | 121 | 143 |  | PEAKS DB |
| G.S(+114.04)GSSGAAAAAAAAAAAAAR.R | 43.61 | 1586.776 | 19 | 3.8 | 794.3983 | 2 | 67.4 | 907 | 925 | Ubiquitin | PEAKS PTM |
| Y.G(+43.01)SGSSGAAAAAAAAAAAAR.R | 43.48 | 1501.723 | 19 | 3.8 | 751.8718 | 2 | 66.8 | 180 | 198 | Carbamylation | PEAKS PTM |
| R.AAGSAAAAAAAAAAAAAGSGAGGHGGGY(+21.98).G | 43.46 | 2106.944 | 28 | 3.1 | 703.3242 | 3 | 75.1 | 205 | 232 | Sodium adduct | PEAKS PTM |
| Y.GSGSSGAAAAAAAAAAAARRAGHDR.A | 43.44 | 2151.064 | 25 | 3.8 | 538.7753 | 4 | 64.1 | 180 | 204 |  | PEAKS DB |
| Y.GSDSAAAAAAAAAAAAAGSGAGGSGGGY(+21.98).G | 43.42 | 2116.902 | 28 | 3.5 | 1059.462 | 2 | 75.3 | 524 | 551 | Sodium adduct | PEAKS PTM |
| F.VVDSSYGSSSSSSAAAAAGSGAGGRGGGY.G | 43.35 | 2479.085 | 29 | 4.9 | 827.3729 | 3 | 52.3 | 115 | 143 |  | PEAKS DB |
| W.GDGGYGSGSAAAAAAAAGSGAGGSGGGY.G | 43.17 | 2130.884 | 28 | 3.9 | 1066.453 | 2 | 60.7 | 1669 | 1696 |  | PEAKS DB |
| Y.GLGDGGYGSGSSAAAAAAAAAAAAGSGAGGAGDGGY.G | 42.89 | 2771.202 | 36 | 4.1 | 924.745 | 3 | 79.3 | 144 | 179 |  | PEAKS DB |
| Y.GHENIYEED(+21.98)VVIK.R | 42.69 | 1565.734 | 13 | 3.2 | 783.8766 | 2 | 66.8 | 72 | 84 | Sodium adduct | PEAKS PTM |
| R.AAGSAAAAAAAAAAAAGGSGAGGHGGGY(+21.98).G | 42.48 | 2092.929 | 28 | 2.7 | 698.652 | 3 | 69.6 | 771 | 798 | Sodium adduct | PEAKS PTM |
| F.VVDSSYGSSSSSSAAAAAGSGAGGR.G | 42.37 | 2144.957 | 25 | 4.7 | 1073.491 | 2 | 48.7 | 115 | 139 |  | PEAKS DB |
| W.GDGGYGSDSAAAAAAAAAAAAAGSGAGGY.G | 42.36 | 2285.979 | 29 | 4.5 | 1144.002 | 2 | 82 | 1278 | 1306 |  | PEAKS DB |
| D.SAAAAAAAAAAAAAGSGGR.G | 42.33 | 1442.723 | 19 | 3.8 | 722.3713 | 2 | 65.8 | 1128 | 1146 |  | PEAKS DB |
| R.VPGASSSAAAASSASAGGR.G | 42.32 | 1560.749 | 19 | 3 | 781.3842 | 2 | 44.6 | 86 | 104 |  | PEAKS DB |
| Y.ESHDSFVVDSSYGSSSSSSAAAAAGSGAGGR.G | 42.28 | 2847.218 | 31 | 4.2 | 950.0839 | 3 | 60.6 | 109 | 139 |  | PEAKS DB |
| Y.GSDDGFVLDGGY.D | 42.25 | 1200.493 | 12 | 3.6 | 601.2562 | 2 | 76.2 | 2747 | 2758 |  | PEAKS DB |
| R.LVETIVLEEDPYN(sub G)HENIYEEDVVIK.R | 42.12 | 3001.481 | 25 | 3.7 | 1001.505 | 3 | 89.9 | 60 | 84 |  | SPIDER |
| G.S(+114.04)DSAAAAAAAAAAAAGSGAGGY.G | 41.99 | 1822.808 | 22 | 2.9 | 912.414 | 2 | 74 | 968 | 989 | Ubiquitin | PEAKS PTM |
| A.KNIRHHDEYVDSN(+.98)GQLF.E | 41.98 | 2071.971 | 17 | 4 | 519.0021 | 4 | 65.4 | 19 | 35 | Deamidation (NQ) | SPIDER |
| R.AAGSAAAAAAAAAAAAAGS(-18.01)GAGGY.G | 41.74 | 1758.829 | 24 | 3.3 | 587.2854 | 3 | 78.5 | 489 | 512 | Dehydration | PEAKS PTM |
| Y.GSGSSGAAAAAAAAAAAAARR.A | 40.85 | 1685.856 | 21 | 2.8 | 562.9608 | 3 | 66.5 | 906 | 926 |  | PEAKS DB |
| G.S(+114.04)DSAAAAAAAAAAAAGSGGR.G | 40.81 | 1687.787 | 20 | 4 | 844.9043 | 2 | 66.2 | 2205 | 2224 | Ubiquitin | PEAKS PTM |
| F.ERNAETRPNLSGN(+.98)ERLVETIVL.E | 40.47 | 2510.309 | 22 | 2.3 | 837.7788 | 3 | 90.6 | 45 | 66 | Deamidation (NQ) | SPIDER |
| Y.GSGSSGAAAAAAAAAAAAR(+380.15)RAG.H | 40.3 | 2123.024 | 22 | 2 | 531.7644 | 4 | 68.5 | 180 | 201 | Nucleophilic addition to cytopiloyne+H2O | PEAKS PTM |
| Y.DSE(+21.98)GSAAAAAAAAAAAASSGAR.S | 40.18 | 1825.817 | 22 | 3.2 | 609.6147 | 3 | 71.6 | 2759 | 2780 | Sodium adduct | PEAKS PTM |
| H.D(+15.99)RAAGSAAAAAAAAAAAAAGSGGRGSGGGY.G | 40.1 | 2407.122 | 30 | 4.1 | 803.3846 | 3 | 68.2 | 2043 | 2072 | Oxidation or Hydroxylation | PEAKS PTM |
| H.DRQ(sub A)AGSAAAAAAAAAAAAAGSGAGGHGGGY.G | 39.93 | 2413.112 | 30 | 3.9 | 805.381 | 3 | 75.7 | 203 | 232 |  | SPIDER |
| R.GDGGYGSGSSAAAAAAAAAAAARRAGY.D | 39.9 | 2298.074 | 27 | 3 | 767.0342 | 3 | 79 | 2691 | 2717 |  | SPIDER |
| Y.GSGSSGAAAAAAAAAAAAAARRAGH(+15.99)DR.A | 39.88 | 2309.133 | 27 | 3.2 | 462.8354 | 5 | 64.1 | 302 | 328 | Oxidation (HW) | PEAKS PTM |
| R.RAGHDRAAGSAAAAAAAAAAAAAGSGSGGY.G | 39.75 | 2485.181 | 30 | 3.4 | 622.3046 | 4 | 72.1 | 323 | 352 |  | PEAKS DB |
| F.ERN(+.98)AETRPNLSGNERLVETIVL.E | 39.65 | 2510.309 | 22 | 3.5 | 837.7798 | 3 | 88 | 45 | 66 | Deamidation (NQ) | SPIDER |
| R.Q(sub A)HGAGSAAAAAAAAAAGA(sub P)GATRPVGVY.G | 39.24 | 2294.152 | 27 | 4 | 765.7275 | 3 | 73.4 | 2720 | 2746 |  | SPIDER |
| Y.GSDSAAAAAAAAAAAAGSGAGGVGGGY(+21.98).G | 39.19 | 2057.901 | 27 | 3.7 | 1029.962 | 2 | 75.6 | 2433 | 2459 | Sodium adduct | PEAKS PTM |
| R.S(sub A)AGSAAAAAAAAAAAAGSS(sub G)AGGSGY.G | 39.05 | 1895.861 | 25 | 4 | 948.9415 | 2 | 71.6 | 2561 | 2585 |  | SPIDER |
| Y.GSGSSGAAAAAAAAAAAAAAR(+15.99)R.A | 38.85 | 1772.888 | 22 | 2.8 | 591.9715 | 3 | 68.2 | 302 | 323 | Oxidation or Hydroxylation | PEAKS PTM |
| A.KNIRHHDEYVDSNGQLF.E | 38.74 | 2070.987 | 17 | 3.2 | 691.3385 | 3 | 64 | 19 | 35 |  | SPIDER |
| G.S(+114.04)GSSGAAAAAAAAAAAAR.R | 38.74 | 1515.739 | 18 | 3 | 758.879 | 2 | 62.6 | 181 | 198 | Ubiquitin | PEAKS PTM |
| W.GDGGYGSDSAAAAAAAAAAAAAGSGAGGSGGGY.G | 38.73 | 2544.075 | 33 | 4.3 | 849.0358 | 3 | 79.4 | 519 | 551 |  | PEAKS DB |
| R.AAGSAS(sub A)AAAAAAAAAAGSS(sub G)AGGSGY.G | 38.6 | 1895.861 | 25 | 3.9 | 948.9414 | 2 | 71.9 | 2561 | 2585 |  | SPIDER |
| Y.GHENIYEEDVV.I | 38.59 | 1302.573 | 11 | 4 | 652.2963 | 2 | 65.5 | 72 | 82 |  | PEAKS DB |
| F.ERNAETRPNLSGNERLVETIVLEEDPY.G | 38.36 | 3142.553 | 27 | 3.3 | 1048.528 | 3 | 90.5 | 45 | 71 |  | SPIDER |
| E.N(+380.15)IYEEDVVIKR.V | 38.32 | 1756.877 | 11 | 1.3 | 586.6337 | 3 | 61.5 | 75 | 85 | Nucleophilic addition to cytopiloyne+H2O | PEAKS PTM |
| R.S(sub A)AGSAAAAAAAAAAAAGSGT(sub A)GGSGY.G | 38.07 | 1895.861 | 25 | 3 | 632.9628 | 3 | 71.9 | 2561 | 2585 |  | SPIDER |
| R.AAGSAAAAAAAAAAAAGGSGAGGY(+37.95).D | 37.77 | 1800.77 | 24 | 2.4 | 601.2654 | 3 | 72.6 | 1248 | 1271 | Replacement of 2 protons by calcium | PEAKS PTM |
| Y.VDSN(+.98)GQLF.E | 37.77 | 879.3974 | 8 | 2.9 | 440.7072 | 2 | 66.3 | 28 | 35 | Deamidation (NQ) | PEAKS DB |
| R.NAETRPNLSGNERL.V | 37.43 | 1569.786 | 14 | 3.9 | 524.2713 | 3 | 55.8 | 47 | 60 |  | PEAKS DB |
| Y.GSGSAAAAAAAAAAAGSGAGGSGD(+21.98)GY.G | 37.43 | 1974.828 | 26 | 4.4 | 988.4256 | 2 | 65.7 | 2629 | 2654 | Sodium adduct | PEAKS PTM |
| R.AAGSAAAAAAAAAAAAAGSGAGGHGGGY(+37.95).G | 37.3 | 2122.909 | 28 | 1.3 | 708.6446 | 3 | 75.2 | 205 | 232 | Replacement of 2 protons by calcium | PEAKS PTM |
| F.ERNAETRPNLSGNERLVE.T | 37.09 | 2083.041 | 18 | 3.6 | 695.3566 | 3 | 58 | 45 | 62 |  | SPIDER |
| R.AAGSAAAAAS(sub A)AAAAAAAGSGGR.G | 37.03 | 1657.813 | 22 | 4.5 | 829.9175 | 2 | 65 | 2045 | 2066 |  | SPIDER |
| Y.GSD(+21.98)SAAAAAAAAAAAAAGSGGR.G | 36.93 | 1723.785 | 22 | 2.6 | 575.6038 | 3 | 70.8 | 1125 | 1146 | Sodium adduct | PEAKS PTM |
| Y.GSGSAAAAAAAAAAAGSGAGGSGDGY(+21.98).G | 36.56 | 1974.828 | 26 | 4.7 | 988.4259 | 2 | 65.7 | 2629 | 2654 | Sodium adduct | PEAKS PTM |
| Y.GSD(+21.98)SAAAAAAAAAAAAGSGGR.G | 36.36 | 1652.748 | 21 | 3.1 | 551.9249 | 3 | 66.2 | 2204 | 2224 | Sodium adduct | PEAKS PTM |
| G.S(+43.01)GSSGAAAAAAAAAAAAAAAR.R | 36.32 | 1657.813 | 21 | 2.8 | 553.6132 | 3 | 71.1 | 1543 | 1563 | Carbamylation | PEAKS PTM |
| G.S(+114.04)GSSGAAAAAAAAAAAARR.A | 36.11 | 1671.84 | 19 | 3.2 | 558.2891 | 3 | 61.9 | 181 | 199 | Ubiquitin | PEAKS PTM |
| Y.GSGSSGAAAAAAAAAAAAQ(sub R)R.A | 36.1 | 1586.776 | 20 | 4.1 | 794.3986 | 2 | 69.9 | 180 | 199 |  | SPIDER |
| R.NAETRPNLSGNERLVETIVL.E | 35.99 | 2224.181 | 20 | 2.8 | 742.4031 | 3 | 88.6 | 47 | 66 |  | SPIDER |
| Y.G(+43.01)SGSSGAAAAAAAAAAAAARRAGHDR.A | 35.74 | 2265.107 | 26 | 4.2 | 567.2864 | 4 | 70 | 906 | 931 | Carbamylation | PEAKS PTM |
| Y.GSSSSSSAAAAAGSGAGGR.G | 35.43 | 1494.666 | 19 | 5.6 | 748.3444 | 2 | 36 | 121 | 139 |  | PEAKS DB |
| Y.G(+43.01)SGSSGAAAAAAAAAAAAAARR.A | 35.25 | 1799.899 | 22 | 3.6 | 900.9598 | 2 | 73.2 | 302 | 323 | Carbamylation | PEAKS PTM |
| H.DRAAGSAAAAAAAAAAAAAGSGAGGY(+21.98).G | 35.05 | 2069.949 | 26 | 3.5 | 690.9927 | 3 | 77.7 | 487 | 512 | Sodium adduct | PEAKS PTM |
| Y.GWGDGGYGSDSAAAAAAAAAAAAAGSGAGGS(+27.05)GDGY.G | 34.79 | 2872.228 | 35 | 7.6 | 958.424 | 3 | 83.8 | 994 | 1028 | Ethyl amino | PEAKS PTM |
| Y.GSGSSGAAAAAAAAAAAARR(+380.15)AG.H | 34.61 | 2123.024 | 22 | 1.9 | 708.6833 | 3 | 68.4 | 180 | 201 | Nucleophilic addition to cytopiloyne+H2O | PEAKS PTM |
| Y.GSGSAAAAAAAAGSGAGGSGGGY(+21.98).G | 34.59 | 1703.711 | 23 | 3.8 | 852.866 | 2 | 51.7 | 1674 | 1696 | Sodium adduct | PEAKS PTM |
| G.SGSAAAAAAAAAAAAAAGSGR(sub A)GGSGGGY.G | 34.55 | 2135.994 | 28 | 3.6 | 713.008 | 3 | 67.9 | 1831 | 1858 |  | SPIDER |
| G.S(+114.04)GSSGAAAAAAAAAAAAAARRAGHDR.A | 34.43 | 2350.16 | 26 | 3.7 | 588.5494 | 4 | 67.7 | 303 | 328 | Ubiquitin | PEAKS PTM |
| G.S(+114.04)GSSGAAAAAAAAAAAAAARR.A | 34.22 | 1813.914 | 21 | 2.3 | 605.6467 | 3 | 69.5 | 303 | 323 | Ubiquitin | PEAKS PTM |
| Y.GHENIYEEDVVIK(+43.01)R(+14.02).V | 34.16 | 1756.874 | 14 | 2.9 | 586.6338 | 3 | 61.7 | 72 | 85 | Carbamylation; Methylation(KR) | PEAKS PTM |
| R.GSGDGYGWGDGGY.G | 34.16 | 1246.453 | 13 | 3.3 | 624.2357 | 2 | 66.8 | 865 | 877 |  | PEAKS DB |
| R.AAGSAAAAAAAAAAAAAGSGSGGY(+37.95).G | 33.92 | 1830.781 | 24 | 2.7 | 611.2692 | 3 | 76.6 | 329 | 352 | Replacement of 2 protons by calcium | PEAKS PTM |
| Y.GSGSSGAAAAAAAAAAAA.R | 33.41 | 1302.616 | 18 | 3.4 | 652.3177 | 2 | 63.1 | 180 | 197 |  | PEAKS DB |
| G.S(+43.01)DSAAAAAAAAAAAAAGSGGR.G | 33.29 | 1687.787 | 21 | 4 | 844.9043 | 2 | 66.4 | 1126 | 1146 | Carbamylation | PEAKS PTM |
| Y.GSDSAAAAAAAAAAAAAGSGAGGY(+37.95).G | 33.2 | 1874.771 | 24 | 3.8 | 625.9332 | 3 | 78 | 1283 | 1306 | Replacement of 2 protons by calcium | PEAKS PTM |
| Y.GSD(+37.95)SAAAAAAAAAAAAAGSGGR.G | 33.13 | 1739.75 | 22 | 3.1 | 580.9257 | 3 | 70.8 | 1125 | 1146 | Replacement of 2 protons by calcium | PEAKS PTM |
| Y.V(+43.01)DSNGQLF.E | 33.03 | 921.4192 | 8 | 4.1 | 461.7188 | 2 | 76.9 | 28 | 35 | Carbamylation | PEAKS PTM |
| Y.GSD(+21.98)SAAAAAAAAAAAAAGGSGGR.G | 32.54 | 1780.806 | 23 | 3.4 | 594.6115 | 3 | 70.3 | 842 | 864 | Sodium adduct | PEAKS PTM |
| H.DRAT(sub A)GSAAAAAAAAAAAAGSGT(sub A)GGSGY.G | 32.2 | 2181.005 | 27 | 4 | 1091.514 | 2 | 70.5 | 2559 | 2585 |  | SPIDER |
| A.A(+87.03)GSAAAAAAAAAAAAAGSGGRGSGGGY.G | 31.88 | 2135.994 | 27 | 3.6 | 713.0079 | 3 | 68.1 | 2046 | 2072 | Glycidamide adduct | PEAKS PTM |
| Y.N(sub G)HENIYEEDVVI.K | 31.77 | 1472.678 | 12 | 3.7 | 737.3492 | 2 | 76.9 | 72 | 83 |  | SPIDER |
| G.S(+114.04)GSSAAAAAAAAAAAARRAGY.D | 31.73 | 1905.94 | 21 | 3.8 | 636.3232 | 3 | 75.5 | 2697 | 2717 | Ubiquitin | PEAKS PTM |
| R.VPGASSSAAAASSASAGGRGGFY(+21.98).E | 31.68 | 2006.906 | 23 | 3.1 | 669.9779 | 3 | 61.6 | 86 | 108 | Sodium adduct | PEAKS PTM |
| E.N(+380.15)IYEEDVVI.K | 31.23 | 1472.681 | 9 | 1.6 | 737.349 | 2 | 76.7 | 75 | 83 | Nucleophilic addition to cytopiloyne+H2O | PEAKS PTM |
| R.T(sub A)GHDRS(sub A)AGSAAAAAAAAAAAAGSGT(sub A)GGSGY.G | 30.8 | 2462.117 | 30 | 4.3 | 821.7164 | 3 | 68.7 | 2556 | 2585 |  | SPIDER |
| Y.VDS(-18.01)NGQLF.E | 30.6 | 860.4028 | 8 | 3.3 | 431.2101 | 2 | 66.8 | 28 | 35 | Dehydration | PEAKS PTM |
| Y.V(+58.01)DSNGQLF.E | 29.7 | 936.4188 | 8 | 3.3 | 469.2182 | 2 | 66.3 | 28 | 35 | Carboxymethyl (KW, X@N-term) | PEAKS PTM |
| A.AAAAAAAAAAGSGAGGHGGGY.G | 29.54 | 1585.723 | 21 | 3 | 793.8713 | 2 | 47.9 | 212 | 232 |  | PEAKS DB |
| R.AGH(+15.99)DRAAGSAAAAAAAAAAAAAGSGGRGSGGGY.G | 29.37 | 2672.24 | 33 | 2.8 | 669.0691 | 4 | 66.1 | 2040 | 2072 | Oxidation (HW) | PEAKS PTM |
| Y.G(+43.01)SGSSGAAAAAAAAAAAARRAGHDR.A | 29.26 | 2194.07 | 25 | 3 | 549.5264 | 4 | 67 | 180 | 204 | Carbamylation | PEAKS PTM |
| Y.GWGDEGYGSGSAAAAAAA.A | 29.09 | 1567.654 | 18 | 4.4 | 784.8376 | 2 | 62.9 | 267 | 284 |  | PEAKS DB |
| R.AAGSAAAAAS(sub A)AAAAAAAGSGAGGHGGGYG.W | 28.86 | 2157.979 | 29 | 2.7 | 720.3354 | 3 | 68.2 | 205 | 233 |  | SPIDER |
| G.S(+114.04)GSSGAAAAAAAAAAAAARRAGHDR.A | 28.62 | 2279.123 | 25 | 2.5 | 570.7894 | 4 | 66.7 | 907 | 931 | Ubiquitin | PEAKS PTM |
| Y.GWGDEGYGSGSAAAAAA.A | 28.49 | 1496.617 | 17 | 3 | 749.3179 | 2 | 61.1 | 267 | 283 |  | PEAKS DB |
| R.AAGSAAAAAAAAAAAAAGSGGR(+15.99)GSGGGY.G | 28.41 | 2135.994 | 28 | 3.9 | 1069.009 | 2 | 68 | 2045 | 2072 | Oxidation or Hydroxylation | PEAKS PTM |
| G.SSGAAAAAAAAAAAAARR.A | 28.36 | 1484.781 | 18 | 3.3 | 495.9359 | 3 | 65.2 | 909 | 926 |  | PEAKS DB |
| Y.GSGSSGAAAAAAAAAAAAR(+37.95).R | 28.33 | 1496.664 | 19 | 3.2 | 499.897 | 3 | 62.6 | 180 | 198 | Replacement of 2 protons by calcium | PEAKS PTM |
| G.AGEGGYGSGSSGAAAAAAAAAAAAARR.A | 28.27 | 2220.063 | 27 | 8.5 | 741.0345 | 3 | 65.3 | 900 | 926 |  | PEAKS DB |
| Y.VDSNGQLFER.F | 27.86 | 1163.557 | 10 | 2.1 | 582.787 | 2 | 56.9 | 28 | 37 |  | PEAKS DB |
| R.AAGSAAAAAAAAAAAAAGSGGR(+15.99).G | 27.77 | 1657.813 | 22 | 3.8 | 829.917 | 2 | 64.8 | 2045 | 2066 | Oxidation or Hydroxylation | PEAKS PTM |
| A.AAAAAAAAAAAGSGAGGHGGGY.G | 27.65 | 1656.76 | 22 | 7.9 | 829.394 | 2 | 54.3 | 211 | 232 |  | PEAKS DB |
| Y.VDSN(+.98)GQLF(+21.98).E | 27.53 | 901.3793 | 8 | 3.4 | 451.6985 | 2 | 66.2 | 28 | 35 | Deamidation (NQ); Sodium adduct | PEAKS PTM |
| F.ERH(sub N)S(sub A)ETRPNLSGNERLVETIVL.E | 27.37 | 2548.336 | 22 | 3 | 850.455 | 3 | 88.6 | 45 | 66 |  | SPIDER |
| A.AAAAAAAAAAGSGAGGY.G | 27.18 | 1277.6 | 17 | 3.2 | 639.8093 | 2 | 51.9 | 496 | 512 |  | PEAKS DB |
| R.AAGSAAAAAAAAAAAAGGSGAGGY(+17.03).D | 26.57 | 1779.85 | 24 | 3.4 | 890.9352 | 2 | 72.6 | 1248 | 1271 | Replacement of proton with ammonium ion | PEAKS PTM |
| R.AGH(+15.99)DRAAGSAAAAAAAAAAAAAGSGGR.G | 26.34 | 2194.059 | 27 | 4.3 | 549.5243 | 4 | 62.8 | 2040 | 2066 | Oxidation (HW) | PEAKS PTM |
| Y.VDSN(-17.03)GQLF.E | 26.3 | 861.3868 | 8 | 3.7 | 431.7023 | 2 | 67 | 28 | 35 | Ammonia-loss (N) | PEAKS PTM |
| S.SGAAAAAAAAAAAAAARR.A | 25.72 | 1468.786 | 18 | 3 | 490.604 | 3 | 67 | 306 | 323 |  | PEAKS DB |
| Y.GSGSAAAAAAAAGSGAGGSGGGY(+37.95).G | 25.2 | 1719.676 | 23 | 3.6 | 574.2347 | 3 | 51.7 | 1674 | 1696 | Replacement of 2 protons by calcium | PEAKS PTM |
| Y.GSDSAAAAAAAAAAAAAGSGAGGY(+17.03).G | 24.73 | 1853.85 | 24 | 3.7 | 927.9359 | 2 | 77.9 | 1283 | 1306 | Replacement of proton with ammonium ion | PEAKS PTM |
| Y.GSGSSGAAAAAAAAAAAARRAGHDRAAG.S | 24.68 | 2350.16 | 28 | 4.9 | 588.55 | 4 | 67.5 | 180 | 207 |  | SPIDER |
| Y.GSGSSGAAAAAAAAAAA.A | 24.5 | 1231.579 | 17 | 3.9 | 616.7993 | 2 | 55.8 | 180 | 196 |  | PEAKS DB |
| Y.GSGSSGAAAAAAAAAAAAAAR(+37.95).R | 24.49 | 1638.739 | 21 | 3.1 | 547.2552 | 3 | 71 | 302 | 322 | Replacement of 2 protons by calcium | PEAKS PTM |
| F.ERH(sub N)S(sub A)ETRPNLSGNERL.V | 24.02 | 1893.94 | 16 | 3.6 | 632.323 | 3 | 57.2 | 45 | 60 |  | SPIDER |
| G.SGSAAAAAAAAAAAAAGSGAGGAGD(+37.03)G.G | 23.93 | 1895.872 | 26 | -2.3 | 632.9632 | 3 | 71.8 | 718 | 743 | Propargylamine | PEAKS PTM |
| G.SGS(+114.04)SGAAAAAAAAAAAAAARRAGHDR.A | 23.48 | 2350.16 | 26 | 3.8 | 471.041 | 5 | 67.6 | 303 | 328 | Ubiquitin | PEAKS PTM |
| R.NAETRPNLSGNERW(sub L)VETIV.L | 23.28 | 2184.092 | 19 | 0.9 | 547.0308 | 4 | 57.4 | 47 | 65 |  | SPIDER |
| Y.GSGSSGAAAAAAAAAA.A | 21.64 | 1160.542 | 16 | 3.9 | 581.2806 | 2 | 50.9 | 180 | 195 |  | PEAKS DB |

Table S9. List of unique peptides detected from *S. ricini* digested with trypsin, FA-trypsin, chymotrypsin, and chymotrypsin-trypsin at False Discovery Rate ≤1%

| TRYPSIN |  |  |  |  |  |  |  |  |  |  |  |
| --- | --- | --- | --- | --- | --- | --- | --- | --- | --- | --- | --- |
| Peptide | **-10lgP** | **Mass** | **Length** | **ppm** | **m/z** | **z** | **RT** | **Start** | **End** | **PTM** | **Found By** |
| K.QVPQGGAASSAASSASAGSGSGAPTIIVER.G | 82.53 | 2670.321 | 30 | 0.7 | 891.1149 | 3 | 33.3 | 101 | 130 |  | PEAKS DB |
| R.VPGGGTLVEE(sub K)IVIER.A | 79.03 | 1566.862 | 15 | 0.5 | 784.4385 | 2 | 44.9 | 70 | 84 |  | SPIDER |
| K.QVPQGGAASSAASSASAGSG.S | 76.47 | 1646.75 | 20 | -1.9 | 824.3804 | 2 | 23.8 | 101 | 120 |  | PEAKS DB |
| A.SSAASSASAGSGSGAPTIIVER.G | 75.6 | 1961.965 | 22 | 1.4 | 981.9913 | 2 | 30.4 | 109 | 130 |  | PEAKS DB |
| S.SAASSASAGSGSGAPTIIVER.G | 75.56 | 1874.933 | 21 | 0.2 | 938.4742 | 2 | 30.4 | 110 | 130 |  | PEAKS DB |
| K.Q(-17.03)VPQGGAASSAASSASAGSGSGAPTIIVER.G | 74.12 | 2653.294 | 30 | -0.7 | 885.438 | 3 | 40.4 | 101 | 130 | Pyro-glu from Q | PEAKS PTM |
| A.ASSASAGSGSGAPTIIVER.G | 72.74 | 1716.864 | 19 | 0.1 | 859.4395 | 2 | 29.6 | 112 | 130 |  | PEAKS DB |
| K.QVPQGGAASSAASSASAGSGSGA.P | 71.41 | 1861.84 | 23 | 1.2 | 931.9285 | 2 | 24.7 | 101 | 123 |  | PEAKS DB |
| K.QVPQGGAASSAASSASAG(-.98).S | 70.39 | 1501.712 | 18 | 1 | 751.8641 | 2 | 23 | 101 | 118 | Amidation | PEAKS PTM |
| K.QVPQGGAASSAASSASAGSGSGAP(-.98).T | 69.46 | 1957.909 | 24 | 0.5 | 979.9623 | 2 | 24.9 | 101 | 124 | Amidation | PEAKS PTM |
| R.SLDDDIN(sub H)SLER.G | 68.6 | 1275.594 | 11 | 0.6 | 638.8047 | 2 | 36.7 | 20 | 30 |  | SPIDER |
| K.QVPQGGAASSAASSASAGSG(-.98).S | 67.18 | 1645.766 | 20 | 0.9 | 823.8907 | 2 | 23.1 | 101 | 120 | Amidation | PEAKS PTM |
| K.QVPQGGAASSAASSASAGSGSGAPT(-.98).I | 66.86 | 2058.957 | 25 | 3.7 | 1030.489 | 2 | 25.5 | 101 | 125 | Amidation | PEAKS PTM |
| A.SAGSGSGAPTIIVER.G | 65.62 | 1400.726 | 15 | -0.2 | 701.3701 | 2 | 29.1 | 116 | 130 |  | PEAKS DB |
| S.Q(sub A)SAGSGSGAPTIIVER.G | 64.99 | 1528.785 | 16 | -0.7 | 765.399 | 2 | 29.6 | 115 | 130 |  | SPIDER |
| R.VD(sub P)GGGTLVEK.I | 63.76 | 973.508 | 10 | -0.3 | 487.7611 | 2 | 22.5 | 70 | 79 |  | SPIDER |
| K.QVPQGGAASSAASSASAG.S | 63.74 | 1502.696 | 18 | -0.3 | 752.3551 | 2 | 23.7 | 101 | 118 |  | PEAKS DB |
| R.VPGGGTLVEK.I | 63.73 | 955.5338 | 10 | 0 | 478.7742 | 2 | 23.3 | 70 | 79 |  | PEAKS DB |
| S.AP(sub G)SGSGAPTIIVER.G | 62.51 | 1353.725 | 14 | -0.1 | 677.8698 | 2 | 31.7 | 117 | 130 |  | SPIDER |
| K.QVPQGGAASSAASSA.S | 62.19 | 1287.606 | 15 | 0.6 | 644.8104 | 2 | 22.7 | 101 | 115 |  | PEAKS DB |
| R.D(sub S)LDDDIHSLER.G | 61.6 | 1326.605 | 11 | 0.2 | 664.3099 | 2 | 39.8 | 20 | 30 |  | SPIDER |
| K.S(sub K)FERDSAPSR.V | 60.79 | 1150.537 | 10 | -8.9 | 384.5161 | 3 | 18.9 | 60 | 69 |  | SPIDER |
| K.QVPQGGAASSAASS.A | 60.66 | 1216.568 | 14 | 0.5 | 609.2917 | 2 | 21 | 101 | 114 |  | PEAKS DB |
| R.SLDDDID(sub H)SLER.G | 60.5 | 1276.578 | 11 | 0.1 | 639.2964 | 2 | 37.8 | 20 | 30 |  | SPIDER |
| A.SSASAGSGSGAPTIIVER.G | 60.15 | 1645.827 | 18 | 0.2 | 823.921 | 2 | 29.5 | 113 | 130 |  | PEAKS DB |
| K.KFD(sub E)RDSAPSR.V | 59.15 | 1177.584 | 10 | -9.3 | 393.5316 | 3 | 18.8 | 60 | 69 |  | SPIDER |
| R.S(+43.01)LDDDIHSLER.G | 58.4 | 1341.616 | 11 | -0.1 | 671.8152 | 2 | 38.4 | 20 | 30 | Carbamylation | PEAKS PTM |
| K.QVPQGGAASSAASSASAGSGSGAP.T | 57.12 | 1958.893 | 24 | 0.4 | 980.4541 | 2 | 26.6 | 101 | 124 |  | PEAKS DB |
| K.QVPQGGAASSAASSASAGSGSGAPT(-2.02)IIVER.G | 57.11 | 2668.305 | 30 | 2 | 890.4442 | 3 | 35.4 | 101 | 130 | 2-amino-3-oxo-butanoic_acid | PEAKS PTM |
| R.KKFD(sub E)RDSAPSR.V | 56.7 | 1305.679 | 11 | -8.7 | 436.2298 | 3 | 17.6 | 59 | 69 |  | SPIDER |
| K.QVPQGGAASSAAS(-.98).S | 56.66 | 1128.552 | 13 | -0.1 | 565.2834 | 2 | 20.3 | 101 | 113 | Amidation | PEAKS PTM |
| K.QVPQGGAASSAASS(-.98).A | 56.01 | 1215.584 | 14 | 0.2 | 608.7996 | 2 | 20.3 | 101 | 114 | Amidation | PEAKS PTM |
| R.VPGGGTLVEK(+27.99)IVIER.A | 56 | 1593.909 | 15 | 0.1 | 797.9618 | 2 | 44.9 | 70 | 84 | Formylation | PEAKS PTM |
| R.S(+27.99)LDDDIHSLER.G | 55.78 | 1326.605 | 11 | 1.1 | 664.3105 | 2 | 38.6 | 20 | 30 | Formylation (Protein N-term) | PEAKS PTM |
| R.N(sub S)W(sub L)DDDIHSLER.G | 55.73 | 1398.616 | 11 | 7 | 700.3203 | 2 | 39.7 | 20 | 30 |  | SPIDER |
| K.KFS(sub E)RDSAPSR.V | 55.22 | 1149.589 | 10 | -9.7 | 384.1999 | 3 | 16.1 | 60 | 69 |  | SPIDER |
| S.SASAGSGSGAPTIIVER.G | 55.18 | 1558.795 | 17 | 1.1 | 780.4056 | 2 | 29.4 | 114 | 130 |  | PEAKS DB |
| R.V(+27.99)HETGDGFLLR.G | 54.34 | 1270.631 | 11 | 0.5 | 636.3229 | 2 | 38.9 | 2822 | 2832 | Formylation | PEAKS PTM |
| S.ASAGSGSGAPTIIVER.G | 53.6 | 1471.763 | 16 | 0.3 | 736.889 | 2 | 29.3 | 115 | 130 |  | PEAKS DB |
| V.PQGGAASSAASSASAGSGSGAPTIIVER.G | 53.45 | 2443.194 | 28 | -0.3 | 815.405 | 3 | 31.8 | 103 | 130 |  | PEAKS DB |
| S.LDDDIHSLER.G | 52.6 | 1211.578 | 10 | 0.3 | 606.7966 | 2 | 27.6 | 21 | 30 |  | PEAKS DB |
| R.VHETGDGFLLR.G | 52.44 | 1242.636 | 11 | 0.6 | 415.2194 | 3 | 30.5 | 2822 | 2832 |  | PEAKS DB |
| V.P(+43.01)GGGTLVEK.I | 52.08 | 899.4712 | 9 | -0.1 | 450.7428 | 2 | 27.8 | 71 | 79 | Carbamylation | PEAKS PTM |
| R.VPGGGTLVEK(+14.96)IVIER.A | 51.99 | 1580.877 | 15 | 0.9 | 791.4467 | 2 | 45.3 | 70 | 84 | Alpha-amino adipic acid | PEAKS PTM |
| R.SHGAGSAAGAAAAAAAAAAAAGGAGR.G | 51.8 | 1963.957 | 26 | -0.1 | 655.6596 | 3 | 33.6 | 139 | 164 |  | PEAKS DB |
| R.D(sub S)R(sub L)DDDIHSLER.G | 51.76 | 1369.622 | 11 | -2.6 | 685.8166 | 2 | 39.1 | 20 | 30 |  | SPIDER |
| S.AASSASAGSGSGAPTIIVER.G | 51.7 | 1787.901 | 20 | 0.3 | 894.9581 | 2 | 29.8 | 111 | 130 |  | PEAKS DB |
| K.QVPQGGAASSAASSAS(-.98).A | 51.25 | 1373.653 | 16 | 0.9 | 687.8346 | 2 | 22 | 101 | 116 | Amidation | PEAKS PTM |
| K.QVPQGGAASSAASSAS.A | 51.15 | 1374.638 | 16 | 0.3 | 688.3262 | 2 | 22.5 | 101 | 116 |  | PEAKS DB |
| R.SLDDDIHSL.E | 50.39 | 1013.467 | 9 | 0.2 | 507.7406 | 2 | 36.2 | 20 | 28 |  | PEAKS DB |
| K.QVPQGGAASSAAS.S | 50.28 | 1129.536 | 13 | -0.1 | 565.7754 | 2 | 20.7 | 101 | 113 |  | PEAKS DB |
| V.PGGGTLVEE(sub K)IVIER.A | 50.09 | 1467.793 | 14 | -0.1 | 734.9038 | 2 | 43.5 | 71 | 84 |  | SPIDER |
| R.DSAPSR(-42.02)VPGGGTLVEK.I | 50.08 | 1526.794 | 16 | 0.9 | 764.4049 | 2 | 25.2 | 64 | 79 | Ornithine from Arginine | PEAKS PTM |
| R.V(+43.01)HETGDGFLLR.G | 49.9 | 1285.642 | 11 | 0.3 | 643.8282 | 2 | 38.6 | 2822 | 2832 | Carbamylation | PEAKS PTM |
| A.RSV(sub L)DDDIHSLER.G | 49.69 | 1440.696 | 12 | -8.1 | 721.3493 | 2 | 33.7 | 19 | 30 |  | SPIDER |
| A.AAAAAAAGGAGGGAGR.G | 49.09 | 1155.575 | 16 | -0.1 | 578.7944 | 2 | 18.1 | 2746 | 2761 |  | PEAKS DB |
| K.QVPQGGAASSAASSASAGSGSGAPTIIVER(-.98).G | 48.97 | 2669.337 | 30 | -12 | 890.7755 | 3 | 33.2 | 101 | 130 | Amidation | PEAKS PTM |
| K.KFERDG(sub S)APSR.V | 48.25 | 1161.589 | 10 | 1.3 | 388.2041 | 3 | 17.8 | 60 | 69 |  | SPIDER |
| A.RD(sub S)LDDDIHSLER.G | 48.14 | 1482.706 | 12 | -0.3 | 495.2426 | 3 | 34.2 | 19 | 30 |  | SPIDER |
| K.Q(+.98)VPQGGAASSAA.S | 48.04 | 1043.488 | 12 | -0.3 | 522.7513 | 2 | 21.9 | 101 | 112 | Deamidation (NQ) | PEAKS DB |
| S.R(sub L)DDDIHSLER.G | 47.34 | 1254.595 | 10 | -8.9 | 628.2993 | 2 | 37.8 | 21 | 30 |  | SPIDER |
| L.D(+27.99)DDIHSLER.G | 47.03 | 1126.489 | 9 | -0.7 | 564.2514 | 2 | 28.5 | 22 | 30 | Formylation | PEAKS PTM |
| K.FERDSAPSR.V | 46.93 | 1063.505 | 9 | 0.2 | 355.5089 | 3 | 17.9 | 61 | 69 |  | PEAKS DB |
| R.SLDDDIHSLER.G | 46.57 | 1298.61 | 11 | 0.7 | 650.3129 | 2 | 33.5 | 20 | 30 |  | PEAKS DB |
| D.DDIHSLER.G | 46.53 | 983.4672 | 8 | 15.2 | 492.7483 | 2 | 28 | 23 | 30 |  | SPIDER |
| K.M(sub F)EP(sub R)DSAPSR.V | 46.53 | 988.4283 | 9 | -7.4 | 495.2178 | 2 | 18.9 | 61 | 69 |  | SPIDER |
| K.QVPQGGAASSAASSASAGSGS.G | 46.21 | 1733.782 | 21 | -0.2 | 867.8979 | 2 | 24 | 101 | 121 |  | PEAKS DB |
| A.AAAAAAGGAGSGY(+44.99)GGGAR.R | 46.18 | 1436.639 | 18 | 0.7 | 719.3273 | 2 | 23.2 | 554 | 571 | Oxidation to nitro | PEAKS PTM |
| S.E(sub A)SD(sub A)GSGSGAPTIIVER.G | 45.62 | 1573.758 | 16 | 0.7 | 787.887 | 2 | 41.9 | 115 | 130 |  | SPIDER |
| A.N(sub A)AAAAAGGSR.S | 45.59 | 844.4151 | 10 | 0.4 | 423.215 | 2 | 21.2 | 1022 | 1031 |  | SPIDER |
| R.S(+42.01)LDDDIHSLER.G | 45.54 | 1340.621 | 11 | 1.3 | 671.3185 | 2 | 39.2 | 20 | 30 | Acetylation (Protein N-term) | PEAKS PTM |
| V.PGGGTLVEK.I | 45.29 | 856.4654 | 9 | -0.5 | 429.2397 | 2 | 19.6 | 71 | 79 |  | PEAKS DB |
| R.KKFERDT(sub S)APSR.V | 45.18 | 1333.71 | 11 | 0.1 | 334.4349 | 4 | 17 | 59 | 69 |  | SPIDER |
| K.QVPQGGAASSA.A | 45.04 | 971.4672 | 11 | 0.4 | 486.741 | 2 | 20.2 | 101 | 111 |  | PEAKS DB |
| A.N(sub A)AAAAGGAGR.G | 44.81 | 814.4045 | 10 | -0.3 | 408.2094 | 2 | 20.9 | 155 | 164 |  | SPIDER |
| T.ARSLDDDA(sub I)HSLER.G | 44.71 | 1483.702 | 13 | -7.3 | 495.5709 | 3 | 35.6 | 18 | 30 |  | SPIDER |
| R.SLDDDIHSL(-.98).E | 44.33 | 1012.483 | 9 | -1.5 | 507.2478 | 2 | 34.6 | 20 | 28 | Amidation | PEAKS PTM |
| A.E(sub R)T(sub S)LDDDIHSLER.G | 44.17 | 1441.669 | 12 | 0.8 | 721.8421 | 2 | 39.5 | 19 | 30 |  | SPIDER |
| K.QVPQGGAASSAA.S | 44.09 | 1042.504 | 12 | 0 | 522.2594 | 2 | 20.9 | 101 | 112 |  | PEAKS DB |
| L.DDDIHSLER.G | 43.98 | 1098.494 | 9 | 0.3 | 550.2545 | 2 | 24.2 | 22 | 30 |  | PEAKS DB |
| R.DSAPSRVPGGGTA(sub L)VER(sub K)IVIER.A | 43.96 | 2165.155 | 21 | 7.2 | 722.7309 | 3 | 43.5 | 64 | 84 |  | SPIDER |
| K.QVPQ(+.98)GGAASSAASSA.S | 43.82 | 1288.59 | 15 | -2.1 | 645.3007 | 2 | 23.8 | 101 | 115 | Deamidation (NQ) | PEAKS DB |
| P.G(+43.01)GGTLVEK.I | 43.47 | 802.4185 | 8 | -0.3 | 402.2164 | 2 | 24.1 | 72 | 79 | Carbamylation | PEAKS PTM |
| G.SGSGAPTIIVER.G | 43.47 | 1185.635 | 12 | 0.8 | 593.8254 | 2 | 28.8 | 119 | 130 |  | PEAKS DB |
| R.VPGGGTLVEK(+43.01)IVIER.A | 43.08 | 1608.92 | 15 | -0.3 | 537.3137 | 3 | 44.5 | 70 | 84 | Carbamylation | PEAKS PTM |
| K.QVPQGGAASSAASSASAGSGS(-.98).G | 43.07 | 1732.798 | 21 | 0.1 | 867.4062 | 2 | 23.2 | 101 | 121 | Amidation | PEAKS PTM |
| K.QVPQ(+.98)GGAASSAASSASAGSGSGAPTIIVER.G | 42.19 | 2671.305 | 30 | 0.3 | 891.4425 | 3 | 34.5 | 101 | 130 | Deamidation (NQ) | PEAKS DB |
| S.AGSGSGAPTIIVER.G | 42.12 | 1313.694 | 14 | -0.2 | 657.8541 | 2 | 29 | 117 | 130 |  | PEAKS DB |
| R.VPGGGTLVEK(+15.99).I | 42.1 | 971.5287 | 10 | -0.2 | 486.7715 | 2 | 23.2 | 70 | 79 | Oxidation or Hydroxylation | PEAKS DB |
| A.R(+.98)SLDDDIHSLER.G | 41.49 | 1455.695 | 12 | 0 | 728.8549 | 2 | 33.4 | 19 | 30 | Deamidation (R) | PEAKS PTM |
| R.VHETGDGF(+15.99)LLR.G | 41.25 | 1258.631 | 11 | 0.4 | 420.551 | 3 | 26.7 | 2822 | 2832 | Oxidation or Hydroxylation | PEAKS DB |
| K.QVPQGGAASSAASSASAGS(-.98).G | 40.58 | 1588.744 | 19 | -0.5 | 795.379 | 2 | 23 | 101 | 119 | Amidation | PEAKS PTM |
| R.LGTSK(+27.99)IAVH | 40.21 | 952.5342 | 9 | 0.4 | 477.2745 | 2 | 27.1 | 2872 | 2880 | Formylation | PEAKS PTM |
| A.A(+43.01)AAAAGGAGGGAGR.G | 39.77 | 1056.506 | 14 | -0.1 | 529.2602 | 2 | 22 | 2748 | 2761 | Carbamylation | PEAKS PTM |
| R.APTGHETI.Y | 39.69 | 824.4028 | 8 | 0.3 | 413.2088 | 2 | 19.5 | 85 | 92 |  | PEAKS DB |
| S.A(+175.04)SAGSGSGAPTIIVER.G | 39.57 | 1646.805 | 16 | 3.2 | 824.4125 | 2 | 36.2 | 115 | 130 | Naphthalene-2,3-dicarboxaldehyde | PEAKS PTM |
| R.S(+114.04)LDDDIHSLER.G | 39.51 | 1412.653 | 11 | -7.7 | 707.3284 | 2 | 41.4 | 20 | 30 | Ubiquitin | PEAKS PTM |
| A.A(+43.01)AAAAAGGAGGGAGR.G | 38.93 | 1127.543 | 15 | -0.5 | 564.7786 | 2 | 23.9 | 2747 | 2761 | Carbamylation | PEAKS PTM |
| A.GSGSGAPTIIVER.G | 38.7 | 1242.657 | 13 | -0.4 | 622.3354 | 2 | 28.7 | 118 | 130 |  | PEAKS DB |
| V.R(+.98)VHETGDGFLLR.G | 38.36 | 1399.721 | 12 | -0.3 | 467.5807 | 3 | 31.6 | 2821 | 2832 | Deamidation (R) | PEAKS PTM |
| G.SGAPTIIVER.G | 38.13 | 1041.582 | 10 | 0.4 | 521.7984 | 2 | 28.7 | 121 | 130 |  | PEAKS DB |
| R.VHETGD(+15.99)GFLLR.G | 38.07 | 1258.631 | 11 | 0.3 | 420.5509 | 3 | 29.5 | 2822 | 2832 | Oxidation or Hydroxylation | PEAKS DB |
| K.KFER(+.98)DSAPSR.V | 37.89 | 1192.584 | 10 | 0 | 398.5352 | 3 | 18.8 | 60 | 69 | Deamidation (R) | PEAKS PTM |
| R.SLDDDIH.S | 37.88 | 813.3505 | 7 | 0.1 | 407.6826 | 2 | 24.2 | 20 | 26 |  | PEAKS DB |
| S.DSAAAAAAAAAAAAAGGAGGG.A | 37.8 | 1499.696 | 21 | 0.2 | 750.8557 | 2 | 50 | 2738 | 2758 |  | PEAKS DB |
| A.AAAAGGAGSGY(+44.99)GGGAR.R | 37.75 | 1294.565 | 16 | 1.6 | 648.2908 | 2 | 21.7 | 556 | 571 | Oxidation to nitro | PEAKS PTM |
| H.ET(-18.01)GDGFLLR.G | 37.6 | 988.4977 | 9 | 0.5 | 495.2564 | 2 | 44 | 2824 | 2832 | Dehydration | PEAKS PTM |
| R.DSAPSR(+15.99)VPGGGTLVEK.I | 37.43 | 1584.811 | 16 | 0.7 | 529.2779 | 3 | 25.5 | 64 | 79 | Oxidation or Hydroxylation | PEAKS DB |
| R.VP(+15.99)GGGTLVEK.I | 37.17 | 971.5287 | 10 | -0.3 | 486.7715 | 2 | 21.2 | 70 | 79 | Oxidation or Hydroxylation | PEAKS DB |
| R.APTGHETIY(+44.99)EEDVVIK.Q | 37.02 | 1844.879 | 16 | -1 | 615.9664 | 3 | 34.6 | 85 | 100 | Oxidation to nitro | PEAKS PTM |
| K.QVPQGGAASSAASSASAGSGSGAP(+15.99)TIIVER.G | 36.73 | 2686.316 | 30 | -0.2 | 896.4456 | 3 | 31.8 | 101 | 130 | Oxidation or Hydroxylation | PEAKS DB |
| K.KFERDSAPSR.V | 36.57 | 1191.6 | 10 | 0.1 | 596.8071 | 2 | 17.4 | 60 | 69 |  | PEAKS DB |
| D.GGAAAAAAAAAAAAAGGAGSG.Y | 36.14 | 1441.691 | 21 | 0.7 | 721.8532 | 2 | 38 | 413 | 433 |  | PEAKS DB |
| P.Q(-17.03)GGAASSAASSASAGSGSGAPTIIVER.G | 36.11 | 2329.115 | 27 | -1.5 | 777.3776 | 3 | 37.8 | 104 | 130 | Pyro-glu from Q | PEAKS PTM |
| S.L(+88.00)DDDIHSLER.G | 35.38 | 1299.576 | 10 | 13.8 | 650.8044 | 2 | 37 | 21 | 30 | 3-sulfanylpropanoyl | PEAKS PTM |
| S.D(+43.99)GGAAAAAAAAAAAAAGGAGGG.Y | 35.2 | 1570.697 | 22 | -0.2 | 786.3557 | 2 | 61.1 | 379 | 400 | Carboxylation (DKW) | PEAKS PTM |
| S.L(+27.99)DDDIHSLER.G | 34.77 | 1239.573 | 10 | 1.6 | 620.7948 | 2 | 37.4 | 21 | 30 | Formylation (Protein N-term) | PEAKS PTM |
| S.A(+88.00)GSGSGAPTIIVER.G | 34.43 | 1401.692 | 14 | 12.5 | 701.8621 | 2 | 36.4 | 117 | 130 | 3-sulfanylpropanoyl | PEAKS PTM |
| K.QVPQ(+.98)GGAASSAASSASAGSGSGA.P | 34.32 | 1862.824 | 23 | 1.8 | 932.4211 | 2 | 25.7 | 101 | 123 | Deamidation (NQ) | PEAKS DB |
| V.PQGGAASSAASSA.S | 33.96 | 1060.479 | 13 | 0.8 | 531.2469 | 2 | 19.3 | 103 | 115 |  | PEAKS DB |
| S.A(+88.00)SAGSGSGAPTIIVER.G | 33.94 | 1559.761 | 16 | 12 | 780.8973 | 2 | 36.8 | 115 | 130 | 3-sulfanylpropanoyl | PEAKS PTM |
| G.P(+15.99)GSAAAAAAAAAAAAGGAGG(+15.99).G | 33.84 | 1442.675 | 20 | 0.6 | 722.3452 | 2 | 52 | 1069 | 1088 | Oxidation or Hydroxylation | PEAKS DB |
| A.RSLD(+43.99)DDIHSLER.G | 33.4 | 1498.701 | 12 | -0.5 | 500.5741 | 3 | 35.2 | 19 | 30 | Carboxylation (DKW) | PEAKS PTM |
| A.S(+43.01)AGSGSGAPTIIVER.G | 33.23 | 1443.732 | 15 | 1.1 | 722.8739 | 2 | 36.3 | 116 | 130 | Carbamylation | PEAKS PTM |
| K.QVPQGGAAS(-.98).S | 32.87 | 812.4141 | 9 | 0.2 | 407.2144 | 2 | 17.9 | 101 | 109 | Amidation | PEAKS PTM |
| K.Q(+.98)VPQGGAASSAASSASAGSGSGAP(+15.99)TIIVER.G | 32.4 | 2687.3 | 30 | 14.3 | 896.7867 | 3 | 33.4 | 101 | 130 | Deamidation (NQ); Oxidation or Hydroxylation | PEAKS DB |
| K.QVPQGGAASSAASSASAGSGSGAPT.I | 32.34 | 2059.941 | 25 | -0.7 | 687.6537 | 3 | 26.2 | 101 | 125 |  | PEAKS DB |
| F.ER(+.98)DSAPSR.V | 31.92 | 917.4202 | 8 | 0.2 | 459.7175 | 2 | 17.7 | 62 | 69 | Deamidation (R) | PEAKS PTM |
| K.QVPQGGAAS.S | 31.79 | 813.3981 | 9 | 0 | 407.7063 | 2 | 18.9 | 101 | 109 |  | PEAKS DB |
| R.VPGGGTLVEK(+14.02).I | 31.31 | 969.5494 | 10 | -0.8 | 485.7816 | 2 | 24 | 70 | 79 | Methylation(KR) | PEAKS PTM |
| K.I(+43.01)VIER.A | 31.09 | 671.3966 | 5 | -0.7 | 336.7054 | 2 | 34.7 | 80 | 84 | Carbamylation | PEAKS PTM |
| R.DSAPSRVPGGGTLVEK.I | 30.86 | 1568.816 | 16 | -0.1 | 523.9458 | 3 | 25.6 | 64 | 79 |  | PEAKS DB |
| R.A(+42.01)PTGHETIY(-18.01)EEDVVIK.Q | 30.74 | 1823.894 | 16 | -9 | 608.9665 | 3 | 29.6 | 85 | 100 | Acetylation (N-term); Dehydration | PEAKS PTM |
| K.QVPQGGAASSA(-.98).A | 30.71 | 970.4832 | 11 | -0.3 | 486.2487 | 2 | 19.2 | 101 | 111 | Amidation | PEAKS PTM |
| R.SLDDDIHS.L | 30.6 | 900.3824 | 8 | 0.3 | 451.1986 | 2 | 25.3 | 20 | 27 |  | PEAKS DB |
| R.VPGGGTLVE(+43.99)KIVIER.A | 30.56 | 1609.904 | 15 | 0.5 | 805.9596 | 2 | 43.5 | 70 | 84 | Carboxylation (E) | PEAKS PTM |
| A.S(+27.99)AGSGSGAPTIIVER.G | 30.55 | 1428.721 | 15 | 0.2 | 715.3678 | 2 | 36.6 | 116 | 130 | Formylation | PEAKS PTM |
| A.AAAGGAGSGY(+44.99)GGGAR.R | 30.51 | 1223.528 | 15 | -0.4 | 612.7709 | 2 | 21.2 | 557 | 571 | Oxidation to nitro | PEAKS PTM |
| FA-TRYPSIN |  |  |  |  |  |  |  |  |  |  |  |
| Peptide | **-10lgP** | **Mass** | **Length** | **ppm** | **m/z** | **z** | **RT** | **Start** | **End** | **PTM** | **Found By** |
| K.QVPQGGAASSAASSASAGSGSGAPTIIVER.G | 79.33 | 2670.321 | 30 | -1.1 | 891.1132 | 3 | 54.3 | 101 | 130 |  | PEAKS DB |
| A.SSAASSASAGSGSGAPTIIVER.G | 79.21 | 1961.965 | 22 | -1.8 | 981.9882 | 2 | 51.8 | 109 | 130 |  | PEAKS DB |
| A.S(+27.99)SAASSASAGSGSGAPTIIVER.G | 75.93 | 1989.96 | 22 | -1.9 | 995.9856 | 2 | 59.9 | 109 | 130 | Formylation | PEAKS PTM |
| K.Q(+.98)VPQGGAASSAASSASAGSGSGAPTIIVER.G | 75.43 | 2671.305 | 30 | -2.6 | 1336.656 | 2 | 54.9 | 101 | 130 | Deamidation (NQ) | PEAKS DB |
| S.SAASSASAGSGSGAPTIIVER.G | 75.35 | 1874.933 | 21 | -1.3 | 938.4727 | 2 | 51.8 | 110 | 130 |  | PEAKS DB |
| S.AASSASAGSGSGAPTIIVER.G | 69.74 | 1787.901 | 20 | -1.9 | 894.9562 | 2 | 51.5 | 111 | 130 |  | PEAKS DB |
| K.Q(-17.03)VPQGGAASSAASSASAGSGSGAPTIIVER.G | 69.45 | 2653.294 | 30 | -2.8 | 885.4362 | 3 | 63.1 | 101 | 130 | Pyro-glu from Q | PEAKS PTM |
| D.SAAAAAAAAAAAAAGGAGGGAGR.G | 67.48 | 1668.829 | 23 | -2.5 | 835.4197 | 2 | 55.6 | 2739 | 2761 |  | PEAKS DB |
| R.VHETGDGFLLR.G | 67.04 | 1242.636 | 11 | -2.3 | 622.3237 | 2 | 51.7 | 2822 | 2832 |  | PEAKS DB |
| R.S(+27.99)HGAGSAAGAAAAAAAAAAAAGGAGR.G | 65.41 | 1991.952 | 26 | -2.2 | 996.9811 | 2 | 60.7 | 139 | 164 | Formylation | PEAKS PTM |
| A.ASSASAGSGSGAPTIIVER.G | 64.74 | 1716.864 | 19 | -2.4 | 859.4373 | 2 | 51.4 | 112 | 130 |  | PEAKS DB |
| A.S(+27.99)SASAGSGSGAPTIIVER.G | 63.51 | 1673.822 | 18 | -2.2 | 837.9164 | 2 | 59.7 | 113 | 130 | Formylation | PEAKS PTM |
| R.VPGGGT(-18.01)LVEK.I | 63.11 | 937.5233 | 10 | -1.9 | 469.768 | 2 | 47 | 70 | 79 | Dehydration | PEAKS PTM |
| K.QVPQ(+.98)GGAASSAASSASAGSGSGAPTIIVER.G | 62.71 | 2671.305 | 30 | -1.6 | 891.4409 | 3 | 54.8 | 101 | 130 | Deamidation (NQ) | PEAKS DB |
| K.Q(-17.03)VPQ(+.98)GGAASSAASSASAGSGSGAPTIIVER.G | 61.44 | 2654.278 | 30 | -2.4 | 885.7646 | 3 | 63.9 | 101 | 130 | Pyro-glu from Q; Deamidation (NQ) | PEAKS PTM |
| R.VPGGGTLVEK(+27.99)IVIER.A | 60.65 | 1593.909 | 15 | -1.5 | 797.9605 | 2 | 66.8 | 70 | 84 | Formylation | PEAKS PTM |
| A.S(+27.99)AGSGSGAPTIIVER.G | 60.62 | 1428.721 | 15 | -3 | 715.3655 | 2 | 60.2 | 116 | 130 | Formylation | PEAKS PTM |
| R.APTGHETIY(+44.99)EEDVVIK.Q | 60.53 | 1844.879 | 16 | -1.9 | 923.4451 | 2 | 55.9 | 85 | 100 | Oxidation to nitro | PEAKS PTM |
| R.V(+43.01)HETGDGFLLR.G | 59.97 | 1285.642 | 11 | -1.9 | 643.8268 | 2 | 61.5 | 2822 | 2832 | Carbamylation | PEAKS PTM |
| G.SDSAAAAAAAAAAAAAGGAGGGAGR.G | 59.37 | 1870.888 | 25 | -1.8 | 936.4496 | 2 | 59.6 | 2737 | 2761 |  | PEAKS DB |
| A.SSASAGSGSGAPTIIVER.G | 58.82 | 1645.827 | 18 | -1.9 | 823.9193 | 2 | 51.3 | 113 | 130 |  | PEAKS DB |
| S.SASAGSGSGAPTIIVER.G | 58.59 | 1558.795 | 17 | -2.1 | 780.4031 | 2 | 51.3 | 114 | 130 |  | PEAKS DB |
| S.S(+27.99)ASAGSGSGAPTIIVER.G | 56.79 | 1586.79 | 17 | -2.4 | 794.4003 | 2 | 60.2 | 114 | 130 | Formylation | PEAKS PTM |
| R.VPGGGTLVEK.I | 53.96 | 955.5338 | 10 | -1.4 | 478.7735 | 2 | 46.5 | 70 | 79 |  | PEAKS DB |
| R.SLDDDIHSLER.G | 52.03 | 1298.61 | 11 | -4.3 | 650.3096 | 2 | 2.94 | 20 | 30 |  | PEAKS DB |
| A.SAGSGSGAPTIIVER.G | 51.38 | 1400.726 | 15 | -1.3 | 701.3693 | 2 | 51.1 | 116 | 130 |  | PEAKS DB |
| R.VPGGGTLVEK(+43.01)IVIER.A | 51.32 | 1608.92 | 15 | -2.3 | 805.4653 | 2 | 66.4 | 70 | 84 | Carbamylation | PEAKS PTM |
| K.Q(+.98)VPQGGAASSAASSA.S | 50.08 | 1288.59 | 15 | -2.4 | 645.3005 | 2 | 45.9 | 101 | 115 | Deamidation (NQ) | PEAKS DB |
| G.SAAGAAAAAAAAAAAAGGAGR.G | 48.86 | 1554.786 | 21 | -1.7 | 778.399 | 2 | 57.9 | 144 | 164 |  | PEAKS DB |
| D.S(+27.99)APSRVPGGGTLVEK.I | 48.57 | 1481.784 | 15 | -1.5 | 741.8981 | 2 | 49.5 | 65 | 79 | Formylation | PEAKS PTM |
| K.Q(-17.03)VPQGGAASSAASSASAGSG.S | 48.48 | 1629.723 | 20 | -1.4 | 815.8676 | 2 | 56.6 | 101 | 120 | Pyro-glu from Q | PEAKS PTM |
| S.AGSGSGAPTIIVER.G | 48.15 | 1313.694 | 14 | -1.8 | 657.853 | 2 | 50.9 | 117 | 130 |  | PEAKS DB |
| S.ASAGSGSGAPTIIVER.G | 47.8 | 1471.763 | 16 | -2.3 | 736.8871 | 2 | 51.3 | 115 | 130 |  | PEAKS DB |
| G.S(+27.99)GAPTIIVER.G | 47.13 | 1069.577 | 10 | -2.6 | 535.7943 | 2 | 61.1 | 121 | 130 | Formylation | PEAKS PTM |
| R.SAAAAAAAAAASSGAR.G | 47.07 | 1273.638 | 16 | -3.4 | 637.8239 | 2 | 45.1 | 2802 | 2817 |  | PEAKS DB |
| D.GGAAAAAAAAAAAAAGGSR.S | 47.02 | 1412.712 | 19 | -1.1 | 707.3624 | 2 | 54.8 | 1013 | 1031 |  | PEAKS DB |
| G.SAAAAAAAAAAAAGGAGGG.Y | 46.09 | 1313.632 | 19 | -1.8 | 657.8223 | 2 | 56.9 | 522 | 540 |  | PEAKS DB |
| D.S(+27.99)AAAAAAAAAAAAAGGAGGGAGR.G | 45.89 | 1696.824 | 23 | -1.7 | 849.4178 | 2 | 65.9 | 2739 | 2761 | Formylation | PEAKS PTM |
| S.DGGAAAAAAAAAAAAAGGAG.R | 45.72 | 1412.664 | 20 | -1.2 | 707.3386 | 2 | 69.3 | 294 | 313 |  | PEAKS DB |
| R.S(+27.99)AAAAAAAAAASSGAR.G | 45.68 | 1301.632 | 16 | -1.8 | 651.8223 | 2 | 59.4 | 2802 | 2817 | Formylation | PEAKS PTM |
| H.G(+27.99)AGSAAGAAAAAAAAAAAAGGAGR.G | 45.37 | 1767.861 | 24 | -1.9 | 884.9362 | 2 | 70.2 | 141 | 164 | Formylation | PEAKS PTM |
| R.S(+43.01)AAAAAAAAAASSGAR.G | 45.34 | 1316.643 | 16 | -1.9 | 659.3276 | 2 | 58.7 | 2802 | 2817 | Carbamylation | PEAKS PTM |
| R.SHGAGSAAGAAAAAAAAAAAAGGA.G | 45.11 | 1750.835 | 24 | -2.2 | 876.4226 | 2 | 59.1 | 139 | 162 |  | PEAKS DB |
| K.QVPQGGAASSAASSASAG.S | 44.96 | 1502.696 | 18 | -2.1 | 752.3537 | 2 | 45.8 | 101 | 118 |  | PEAKS DB |
| G.GTLVEK(+27.99)IVIER.A | 44.66 | 1283.745 | 11 | -2.3 | 642.8782 | 2 | 63.6 | 74 | 84 | Formylation | PEAKS PTM |
| S.A(+43.01)AAAAAAAAAAAAGGAGGGAGR.G | 44.61 | 1624.803 | 22 | -1.7 | 813.4073 | 2 | 69.2 | 2740 | 2761 | Carbamylation | PEAKS PTM |
| S.SAAAAAAAAAAAAAGGAGGG.Y | 44.58 | 1384.669 | 20 | -1.7 | 693.3408 | 2 | 60.9 | 1189 | 1208 |  | PEAKS DB |
| G.GTLVEK(+43.01)IVIER.A | 44.58 | 1298.756 | 11 | -1.6 | 650.3841 | 2 | 63.2 | 74 | 84 | Carbamylation | PEAKS PTM |
| S.S(+43.01)ASAGSGSGAPTIIVER.G | 44.55 | 1601.801 | 17 | 0 | 801.9077 | 2 | 59.9 | 114 | 130 | Carbamylation | PEAKS PTM |
| R.VPGGGTLVE(+14.02)K.I | 44.27 | 969.5494 | 10 | -1.9 | 485.7811 | 2 | 47.9 | 70 | 79 | Methylation(others) | PEAKS PTM |
| G.S(+27.99)DSAAAAAAAAAAAAAGGAGGGAGR.G | 43.86 | 1898.883 | 25 | -2.8 | 950.4461 | 2 | 69.2 | 2737 | 2761 | Formylation | PEAKS PTM |
| A.AAAAAAAAAGGAGGGAGR.G | 43.74 | 1297.649 | 18 | -1.7 | 649.8305 | 2 | 44.5 | 2744 | 2761 |  | PEAKS DB |
| R.APTGHETIY(+44.99)EE.D | 43.66 | 1290.536 | 11 | -2.6 | 646.2738 | 2 | 51.8 | 85 | 95 | Oxidation to nitro | PEAKS PTM |
| R.LGTSK(+27.99)IAVH | 43.65 | 952.5342 | 9 | -1.8 | 477.2735 | 2 | 49 | 2872 | 2880 | Formylation | PEAKS PTM |
| G.DGGAAAAAAAAAAAAAGGAGSGY(+44.99)GGGAR.R | 43.27 | 2162.969 | 28 | -2.8 | 721.9948 | 3 | 70.1 | 544 | 571 | Oxidation to nitro | PEAKS PTM |
| D.GGAAAAAAAAAAAAAGGAGR.G | 43.06 | 1453.739 | 20 | -1.8 | 727.8752 | 2 | 56.1 | 295 | 314 |  | PEAKS DB |
| S.GSGAPTIIVER.G | 43.03 | 1098.603 | 11 | -2 | 550.3078 | 2 | 50.8 | 120 | 130 |  | PEAKS DB |
| R.GHGTAGSAAAAAAAAAAAAASSEGG.S | 42.97 | 1925.883 | 25 | -2.6 | 963.9461 | 2 | 58.3 | 173 | 197 |  | PEAKS DB |
| S.R(+27.99)VPGGGTLVEK.I | 42.76 | 1139.63 | 11 | -2 | 570.8211 | 2 | 49.1 | 69 | 79 | Formylation | PEAKS PTM |
| A.S(+43.01)AGSGSGAPTIIVER.G | 42.7 | 1443.732 | 15 | -1.4 | 722.8721 | 2 | 59.9 | 116 | 130 | Carbamylation | PEAKS PTM |
| S.G(+43.01)SGAPTIIVER.G | 42.44 | 1141.609 | 11 | -2.4 | 571.8105 | 2 | 60.4 | 120 | 130 | Carbamylation | PEAKS PTM |
| S.G(+27.99)SGAPTIIVER.G | 42.34 | 1126.598 | 11 | -1.6 | 564.3054 | 2 | 60.8 | 120 | 130 | Formylation | PEAKS PTM |
| G.D(+27.99)GGAAAAAAAAAAAAAGGSR.S | 42.1 | 1555.734 | 20 | -1.5 | 778.873 | 2 | 72 | 1012 | 1031 | Formylation | PEAKS PTM |
| G.SAAAAAAAAAAAAGGAGG.G | 42.07 | 1256.611 | 18 | -2.2 | 629.3113 | 2 | 57 | 522 | 539 |  | PEAKS DB |
| R.VPGGGTLVEKIVIER(+.98).A | 41.98 | 1566.898 | 15 | -2 | 784.4548 | 2 | 67 | 70 | 84 | Deamidation (R) | PEAKS PTM |
| D.G(+42.01)GAAAAAAAAAAAAAGGAGR.G | 41.83 | 1495.749 | 20 | -2 | 748.8803 | 2 | 69.9 | 295 | 314 | Acetylation (N-term) | PEAKS PTM |
| R.VPGGGTLVEK(+42.01)IVIER.A | 41.66 | 1607.925 | 15 | -2.1 | 804.9678 | 2 | 67.2 | 70 | 84 | Acetylation (K) | PEAKS PTM |
| G.A(+27.99)GSAAGAAAAAAAAAAAAGGAGR.G | 41.31 | 1710.84 | 23 | -2.4 | 856.4251 | 2 | 70.5 | 142 | 164 | Formylation | PEAKS PTM |
| E.T(+27.99)GDGFLLR.G | 41.19 | 905.4606 | 8 | -2 | 453.7367 | 2 | 68.9 | 2825 | 2832 | Formylation | PEAKS PTM |
| D.G(+27.99)GAAAAAAAAAAAAAGGAGR.G | 41.1 | 1481.733 | 20 | -1.4 | 741.8729 | 2 | 68.8 | 295 | 314 | Formylation | PEAKS PTM |
| A.G(+27.99)AAAAAAAAAAAAGGAGR.G | 40.74 | 1353.675 | 18 | -1.6 | 677.8436 | 2 | 66 | 147 | 164 | Formylation | PEAKS PTM |
| D.GGAAAAAAAAAAAAAGGAGD.G | 40.54 | 1412.664 | 20 | -1.8 | 707.3381 | 2 | 62.4 | 497 | 516 |  | PEAKS DB |
| P.S(+27.99)RVPGGGTLVEK.I | 40.3 | 1226.662 | 12 | -2.5 | 614.3367 | 2 | 48.3 | 68 | 79 | Formylation | PEAKS PTM |
| P.SR(+.98)VPGGGTLVEK.I | 40.28 | 1199.651 | 12 | -2.2 | 600.8315 | 2 | 48.5 | 68 | 79 | Deamidation (R) | PEAKS PTM |
| K.Q(-17.03)VPQGGAASSAA.S | 40.14 | 1025.478 | 12 | -2.3 | 513.745 | 2 | 57.1 | 101 | 112 | Pyro-glu from Q | PEAKS PTM |
| A.G(+27.99)SAAGAAAAAAAAAAAAGGAGR.G | 40 | 1639.803 | 22 | -1.6 | 820.9073 | 2 | 69.4 | 143 | 164 | Formylation | PEAKS PTM |
| S.DGGAAAAAAAAAAAAAGGA.G | 39.86 | 1355.643 | 19 | -2.2 | 678.8272 | 2 | 75.4 | 294 | 312 |  | PEAKS DB |
| G.S(+27.99)GSGAPTIIVER.G | 39.79 | 1213.63 | 12 | -1.5 | 607.8215 | 2 | 60.3 | 119 | 130 | Formylation | PEAKS PTM |
| R.VPGGGTLVEK(+43.01).I | 39.73 | 998.5396 | 10 | -1.8 | 500.2762 | 2 | 55 | 70 | 79 | Carbamylation | PEAKS PTM |
| G.G(+27.99)DGGAAAAAAAAAAAAAGGSR.S | 39.55 | 1612.755 | 21 | -1.8 | 807.3834 | 2 | 70.9 | 1011 | 1031 | Formylation | PEAKS PTM |
| R.DSAPSRVPGGGTLVEK.I | 39.54 | 1568.816 | 16 | -2.2 | 785.4135 | 2 | 48 | 64 | 79 |  | PEAKS DB |
| A.G(+27.99)SGSGAPTIIVER.G | 39.52 | 1270.652 | 13 | -1.7 | 636.3321 | 2 | 60.1 | 118 | 130 | Formylation | PEAKS PTM |
| D.N(+.98)AAAAAAAAAAAAGGAGGD(+15.99)Y(+15.99)GR.G | 39.42 | 1807.809 | 22 | -15 | 904.8982 | 2 | 73.9 | 2775 | 2796 | Deamidation (NQ); Oxidation or Hydroxylation | PEAKS DB |
| R.V(+27.99)PGGGTLVEK.I | 39.35 | 983.5287 | 10 | -2.7 | 492.7703 | 2 | 58.4 | 70 | 79 | Formylation | PEAKS PTM |
| G.SGSGAPTIIVER.G | 39.29 | 1185.635 | 12 | -2 | 593.8237 | 2 | 50.8 | 119 | 130 |  | PEAKS DB |
| D.G(+27.99)GAAAAAAAAAAAAAGGSR.S | 39.28 | 1440.707 | 19 | -2.5 | 721.3589 | 2 | 61.2 | 1013 | 1031 | Formylation | PEAKS PTM |
| G.TLVEK(+27.99)IVIER.A | 39.27 | 1226.723 | 10 | -1.7 | 614.3679 | 2 | 62.2 | 75 | 84 | Formylation | PEAKS PTM |
| A.AAAAAAAGGAGSGY(+44.99)GGGAR.R | 39.26 | 1507.676 | 19 | -3 | 754.8431 | 2 | 46.2 | 553 | 571 | Oxidation to nitro | PEAKS PTM |
| G.S(+43.01)GAPTIIVER.G | 39.18 | 1084.588 | 10 | -1.7 | 543.3002 | 2 | 60.6 | 121 | 130 | Carbamylation | PEAKS PTM |
| G.SAAAAAAAAAAAAGGAG.G | 39.04 | 1199.589 | 17 | -2.5 | 600.8005 | 2 | 57 | 522 | 538 |  | PEAKS DB |
| R.LGTSK(+43.01)IAVH | 38.93 | 967.545 | 9 | -1.9 | 484.7789 | 2 | 48.6 | 2872 | 2880 | Carbamylation | PEAKS PTM |
| E.TGDGFLLR.G | 38.91 | 877.4658 | 8 | -2.3 | 439.7391 | 2 | 57.5 | 2825 | 2832 |  | PEAKS DB |
| D.GGAAAAAAAAAAAAAGGAGSGYGGGSW(+43.99).H | 38.72 | 2092.92 | 27 | 1.2 | 1047.468 | 2 | 62.8 | 413 | 439 | Carboxylation (DKW) | PEAKS PTM |
| G.S(+27.99)AAGAAAAAAAAAAAAGGAGR.G | 38.62 | 1582.781 | 21 | -1.7 | 792.3965 | 2 | 69.8 | 144 | 164 | Formylation | PEAKS PTM |
| A.AAAAGGAGSGY(+44.99)GGGAR.R | 38.28 | 1294.565 | 16 | -1.8 | 648.2886 | 2 | 45.2 | 556 | 571 | Oxidation to nitro | PEAKS PTM |
| A.AAAAAAGGAGSGY(+44.99)GGGAR.R | 38.12 | 1436.639 | 18 | -2.1 | 719.3253 | 2 | 45.7 | 554 | 571 | Oxidation to nitro | PEAKS PTM |
| E.TIY(+44.99)EEDVVIK.Q | 38.11 | 1252.619 | 10 | -1.9 | 627.3154 | 2 | 60 | 91 | 100 | Oxidation to nitro | PEAKS PTM |
| R.SHGAGSAAGAAAAAAAAAAAAGGAG.R | 37.95 | 1807.856 | 25 | -2.4 | 904.9331 | 2 | 58.5 | 139 | 163 |  | PEAKS DB |
| A.AAAAAAAAAGGAGSGY(+44.99)GGGAR.R | 37.8 | 1649.751 | 21 | -2.2 | 825.8807 | 2 | 48.1 | 551 | 571 | Oxidation to nitro | PEAKS PTM |
| K.Q(+.98)VPQ(+.98)GGAASSAASSA.S | 37.69 | 1289.574 | 15 | -2 | 645.7927 | 2 | 46.9 | 101 | 115 | Deamidation (NQ) | PEAKS DB |
| G.TLVEK(+43.01)IVIER.A | 37.68 | 1241.734 | 10 | -1.8 | 621.8733 | 2 | 62.1 | 75 | 84 | Carbamylation | PEAKS PTM |
| S.AEAAAAAAAAAAAAGTAAG.G | 37.61 | 1427.7 | 19 | 5.5 | 714.8615 | 2 | 71 | 234 | 252 |  | PEAKS DB |
| K.Q(+.98)VPQGGAASSAA.S | 37.41 | 1043.488 | 12 | -2 | 522.7504 | 2 | 45.5 | 101 | 112 | Deamidation (NQ) | PEAKS DB |
| A.AAAAAAGGAGSGYGGGM(sub S)W.H | 37.31 | 1481.636 | 18 | -9.1 | 741.8184 | 2 | 46.2 | 422 | 439 |  | SPIDER |
| Y.GGDGGAAAAAAAAAAAAAGGAG.S | 37.3 | 1526.707 | 22 | -1.8 | 764.3596 | 2 | 69.8 | 410 | 431 |  | PEAKS DB |
| A.AAAAAGGAGSGY(+44.99)GGGAR.R | 36.99 | 1365.602 | 17 | -2.3 | 683.8068 | 2 | 45.4 | 555 | 571 | Oxidation to nitro | PEAKS PTM |
| A.AAAGGAGSGY(+44.99)GGGAR.R | 36.9 | 1223.528 | 15 | -1.8 | 612.7701 | 2 | 45 | 557 | 571 | Oxidation to nitro | PEAKS PTM |
| R.GHGTAGSAAAAAAAAAAAAASSE.G | 36.9 | 1811.84 | 23 | -2.4 | 906.9249 | 2 | 58.7 | 173 | 195 |  | PEAKS DB |
| K.Q(-17.03)VPQGGAASSAASSA.S | 36.9 | 1270.579 | 15 | -2.2 | 636.2953 | 2 | 57.3 | 101 | 115 | Pyro-glu from Q | PEAKS PTM |
| V.PGGGTLVEK.I | 36.89 | 856.4654 | 9 | -2.4 | 429.239 | 2 | 57.4 | 71 | 79 |  | PEAKS DB |
| S.A(+42.01)P(+15.99)SRVPGGGTLVEK.I | 36.84 | 1424.762 | 14 | -2.1 | 713.387 | 2 | 50.1 | 66 | 79 | Acetylation (N-term); Oxidation or Hydroxylation | PEAKS PTM |
| Y.GP(+15.99)GSAAAAAAAAAAAAGGAG(+15.99).G | 36.65 | 1442.675 | 20 | -0.4 | 722.3444 | 2 | 80.6 | 1068 | 1087 | Oxidation or Hydroxylation | PEAKS DB |
| G.G(+27.99)AAAAAAAAAAAAAGGSR.S | 36.63 | 1383.685 | 18 | -1.5 | 692.8489 | 2 | 68.4 | 1014 | 1031 | Formylation | PEAKS PTM |
| S.D(sub G)SGAPTIIVER.G | 36.55 | 1156.609 | 11 | -1.9 | 579.3105 | 2 | 60.6 | 120 | 130 |  | SPIDER |
| A.AAAAAAAAAAGGAGGGAGR.G | 36.3 | 1368.686 | 19 | -2.4 | 685.3485 | 2 | 45.1 | 2743 | 2761 |  | PEAKS DB |
| D.GGAAAAAAAAAAAAAGGAGSGY(+44.99)GGGAR.R | 36.28 | 2047.942 | 27 | -2.2 | 1024.976 | 2 | 61.7 | 545 | 571 | Oxidation to nitro | PEAKS PTM |
| R.GSGAW(sub G)SGR.S | 35.74 | 776.3565 | 8 | -1.8 | 389.1848 | 2 | 44.5 | 131 | 138 |  | SPIDER |
| R.DSAPSRVPGGG.T | 35.72 | 998.4781 | 11 | -2.3 | 500.2451 | 2 | 45.4 | 64 | 74 |  | PEAKS DB |
| S.SAAAAAAAAAAAAAGGAGG.G | 35.6 | 1327.648 | 19 | -1.6 | 664.8302 | 2 | 61.1 | 1189 | 1207 |  | PEAKS DB |
| S.G(+27.99)APTIIVER.G | 35.55 | 982.5447 | 9 | -1.9 | 492.2787 | 2 | 62.1 | 122 | 130 | Formylation | PEAKS PTM |
| G.SGE(sub A)PTIIVER.G | 35.24 | 1099.587 | 10 | -2.4 | 550.7996 | 2 | 61.2 | 121 | 130 |  | SPIDER |
| G.G(+43.01)AAAAAAAAAAAAAGGSR.S | 35.16 | 1398.696 | 18 | -1.2 | 700.3546 | 2 | 67.9 | 1014 | 1031 | Carbamylation | PEAKS PTM |
| G.A(+27.99)PTIIVER.G | 35.13 | 925.5233 | 8 | -1.8 | 463.7681 | 2 | 63.5 | 123 | 130 | Formylation | PEAKS PTM |
| G.DGGAAAAAAAAAAAAAGGAGSGY(+117.00)G.G | 35.11 | 1893.801 | 24 | -14 | 947.8945 | 2 | 86.2 | 412 | 435 | Phospho-propargylamine | PEAKS PTM |
| D.SAPSR(+.98)VPGGGTLVEK.I | 35.06 | 1454.773 | 15 | -2.5 | 728.3918 | 2 | 49.5 | 65 | 79 | Deamidation (R) | PEAKS PTM |
| G.GAAAAAAAAAAAAAGGAGGG.Y | 35.02 | 1354.659 | 20 | -2.9 | 678.3347 | 2 | 60.1 | 381 | 400 |  | PEAKS DB |
| D.D(+27.99)IHSLER.G | 35 | 896.4352 | 7 | -2.3 | 449.2238 | 2 | 53.5 | 24 | 30 | Formylation | PEAKS PTM |
| G.S(+27.99)AAAAAAAAAAAAASSEGG.S | 34.95 | 1473.669 | 19 | -1.5 | 737.8409 | 2 | 83.7 | 179 | 197 | Formylation | PEAKS PTM |
| D.SAEAAAAAAAAAAAAGTAAGG.S | 34.78 | 1571.754 | 21 | -2.4 | 786.8823 | 2 | 66 | 233 | 253 |  | PEAKS DB |
| R.S(+43.01)HGAGSAAGAAAAAAAAAAAAGGAGR.G | 34.62 | 2006.963 | 26 | -2.5 | 669.9933 | 3 | 60.7 | 139 | 164 | Carbamylation | PEAKS PTM |
| R.SHGAGSAAGAAAAAAAAAAAAGG.A | 34.61 | 1679.798 | 23 | -2.5 | 840.9039 | 2 | 57.4 | 139 | 161 |  | PEAKS DB |
| G.GDGGAAAAAAAAAAAAAGGAGSGY(+44.99)GGGAR.R | 34.58 | 2219.99 | 29 | -2.1 | 741.0024 | 3 | 68 | 543 | 571 | Oxidation to nitro | PEAKS PTM |
| D.S(+27.99)AEAAAAAAAAAAAAGTAAGGSGGG.Y | 34.55 | 1857.845 | 25 | -1.5 | 929.9285 | 2 | 82.4 | 233 | 257 | Formylation | PEAKS PTM |
| R.VPGGGTLVE(+21.98)K.I | 34.46 | 977.5157 | 10 | -5.3 | 489.7625 | 2 | 46 | 70 | 79 | Sodium adduct | PEAKS PTM |
| A.PTIIVER.G | 34.37 | 826.4912 | 7 | -2.1 | 414.252 | 2 | 48 | 124 | 130 |  | PEAKS DB |
| S.D(sub G)APTIIVER.G | 34.37 | 1012.555 | 9 | -2.5 | 507.2837 | 2 | 61.7 | 122 | 130 |  | SPIDER |
| R.APTGHETIY(+44.99)E.E | 34.37 | 1161.494 | 10 | -1.6 | 581.7532 | 2 | 50.9 | 85 | 94 | Oxidation to nitro | PEAKS PTM |
| R.V(+43.01)PGGGTLVEK.I | 34.24 | 998.5396 | 10 | -1.6 | 500.2763 | 2 | 57.3 | 70 | 79 | Carbamylation | PEAKS PTM |
| G.S(+27.99)DGGAAAAAAAAAAAAAGGAGR.G | 34.06 | 1683.792 | 22 | -2.6 | 842.9013 | 2 | 72 | 293 | 314 | Formylation | PEAKS PTM |
| G.T(+27.99)LVEKIVIER.A | 34.04 | 1226.723 | 10 | -2.3 | 614.3676 | 2 | 65.3 | 75 | 84 | Formylation | PEAKS PTM |
| S.GE(sub A)PTIIVER.G | 33.96 | 1012.555 | 9 | -1.8 | 507.284 | 2 | 65.4 | 122 | 130 |  | SPIDER |
| S.DSAAAAAAAAAAAAAGGAG.G | 33.95 | 1385.653 | 19 | -1.9 | 693.8327 | 2 | 80.9 | 2738 | 2756 |  | PEAKS DB |
| A.M(sub G)SGSGAPTIIVER.G | 33.8 | 1316.676 | 13 | 12.1 | 659.3531 | 2 | 51.1 | 118 | 130 |  | SPIDER |
| D.SGAAAAAAAAAAAAAGGAGD.G | 33.74 | 1442.675 | 20 | -1.2 | 722.3439 | 2 | 62.2 | 1046 | 1065 |  | PEAKS DB |
| K.Q(-17.03)VPQGGAASSAASSASAG.S | 33.67 | 1485.67 | 18 | -1.9 | 743.8406 | 2 | 57.1 | 101 | 118 | Pyro-glu from Q | PEAKS PTM |
| D.NAAAAAAAAAAAAGGAGGD.Y | 33.64 | 1398.649 | 19 | -2 | 700.3302 | 2 | 58.5 | 2775 | 2793 |  | PEAKS DB |
| G.GAAAAAAAAAAAAAGGAGSGYGGGSW(+43.99).H | 33.61 | 2035.898 | 26 | 1.2 | 1018.958 | 2 | 61.8 | 414 | 439 | Carboxylation (DKW) | PEAKS PTM |
| K.IVIER(+.98)APTGHE.T | 33.57 | 1221.635 | 11 | -2 | 611.8237 | 2 | 49.1 | 80 | 90 | Deamidation (R) | PEAKS PTM |
| G.GE(sub A)AAAAAAAAAAAAGGSR.S | 33.32 | 1413.696 | 18 | -1.1 | 707.8544 | 2 | 67.9 | 1014 | 1031 |  | SPIDER |
| A.GSGSGAPTIIVER.G | 33.07 | 1242.657 | 13 | -2.5 | 622.3341 | 2 | 50.8 | 118 | 130 |  | PEAKS DB |
| S.DSAAAAAAAAAAAAAGGAGGG.A | 33.01 | 1499.696 | 21 | -3.4 | 750.853 | 2 | 78 | 2738 | 2758 |  | PEAKS DB |
| S.D(+15.99)GGAAAAAAAAAAAAAGGA.G | 33 | 1371.638 | 19 | -1.3 | 686.8253 | 2 | 65.8 | 294 | 312 | Oxidation or Hydroxylation | PEAKS DB |
| A.EAAAAAAAAAAAAGTAAG.G | 32.81 | 1356.663 | 18 | 6.4 | 679.3433 | 2 | 67.2 | 235 | 252 |  | PEAKS DB |
| D.GGAAAAAAAAAAAAAGGAGGG.Y | 32.8 | 1411.68 | 21 | -2.9 | 706.8454 | 2 | 61.6 | 380 | 400 |  | PEAKS DB |
| G.SGAPTIIVER.G | 32.75 | 1041.582 | 10 | -1.4 | 521.7974 | 2 | 50.9 | 121 | 130 |  | PEAKS DB |
| A.AAAAAAAAGGAGSGY(+44.99)GGGAR.R | 32.74 | 1578.713 | 20 | -1.2 | 790.363 | 2 | 47 | 552 | 571 | Oxidation to nitro | PEAKS PTM |
| G.T(+27.99)AGSAAAAAAAAAAAAASSEGG.S | 32.63 | 1702.776 | 22 | -3 | 852.3926 | 2 | 81.7 | 176 | 197 | Formylation | PEAKS PTM |
| S.G(+127.06)SGAPTIIVER.G | 32.61 | 1225.667 | 11 | -2.3 | 613.8392 | 2 | 55.7 | 120 | 130 | N-Succinimidyl-2-morpholine acetate | PEAKS PTM |
| D.GGAAAAAAAAAAAAAGGAGSGY(+44.99)GGSAR.R | 32.55 | 2077.952 | 27 | -3.3 | 693.6558 | 3 | 61.8 | 669 | 695 | Oxidation to nitro | PEAKS PTM |
| D.GGAAAAAAAAAAAAAGGAGSGY(+117.00)G.G | 32.54 | 1778.774 | 23 | -11 | 890.3846 | 2 | 70 | 413 | 435 | Phospho-propargylamine | PEAKS PTM |
| D.SSAAAAAAAAAAAGSAGG.S | 32.54 | 1302.616 | 18 | -2.2 | 652.314 | 2 | 55.1 | 210 | 227 |  | PEAKS DB |
| G.SAAAAAAAAAAAAGGAGGGY(+44.99)GG.D | 32.53 | 1635.724 | 22 | -2.6 | 818.867 | 2 | 64.8 | 522 | 543 | Oxidation to nitro | PEAKS PTM |
| R.APTGHETIY(+44.99)EED.V | 32.52 | 1405.563 | 12 | -2.1 | 703.7875 | 2 | 51.9 | 85 | 96 | Oxidation to nitro | PEAKS PTM |
| G.S(+27.99)AAAAAAAAAAAAGGSGGG.Y | 32.42 | 1357.622 | 19 | -1.4 | 679.8174 | 2 | 74.7 | 320 | 338 | Formylation | PEAKS PTM |
| G.GDGGAAAAAAAAAAAAAGGAG.S | 32.32 | 1469.686 | 21 | -1.8 | 735.8488 | 2 | 70.4 | 411 | 431 |  | PEAKS DB |
| D.GGAAAAAAAAAAAAASGAG.S | 32.26 | 1327.648 | 19 | -1.9 | 664.83 | 2 | 62.1 | 262 | 280 |  | PEAKS DB |
| D.N(+.98)AAAAAAAAAAAAGGAGG.D | 32.24 | 1284.606 | 18 | -3.3 | 643.308 | 2 | 76.4 | 2775 | 2792 | Deamidation (NQ) | PEAKS DB |
| D.D(+27.99)DIHSLER.G | 32.2 | 1011.462 | 8 | -1.7 | 506.7375 | 2 | 54.7 | 23 | 30 | Formylation | PEAKS PTM |
| S.DSAAAAAAAAAAAAAGGAG(+21.98).G | 32.18 | 1407.635 | 19 | -1.8 | 704.8237 | 2 | 81 | 2738 | 2756 | Sodium adduct | PEAKS PTM |
| R.VHETGDGFLL.R | 32.18 | 1086.535 | 10 | -1.8 | 544.2736 | 2 | 62.4 | 2822 | 2831 |  | PEAKS DB |
| D.S(+27.99)GAAAAAAAAAAAAAGGAE.G | 32.17 | 1427.664 | 19 | -3.1 | 714.8371 | 2 | 80.2 | 2103 | 2121 | Formylation | PEAKS PTM |
| Y.GP(+15.99)GSAAAAAAAAAAAAGGAGG(+15.99).G | 32.08 | 1499.696 | 21 | -2.6 | 750.8535 | 2 | 80.2 | 1068 | 1088 | Oxidation or Hydroxylation | PEAKS DB |
| G.A(+43.01)AAAAAAAAAAAAGGAGG.G | 32.07 | 1283.622 | 18 | -2.2 | 642.8167 | 2 | 57.7 | 382 | 399 | Carbamylation | PEAKS PTM |
| R.VPGGGTLVEW(sub K)IVIER.A | 32.06 | 1623.898 | 15 | 11.2 | 542.3128 | 3 | 63.2 | 70 | 84 |  | SPIDER |
| D.I(+27.99)HSLER.G | 32.03 | 781.4082 | 6 | -1.8 | 391.7107 | 2 | 46.1 | 25 | 30 | Formylation | PEAKS PTM |
| S.D(+27.99)GGAAAAAAAAAAAAAGGAG.R | 31.89 | 1440.659 | 20 | -1.8 | 721.3356 | 2 | 82.6 | 294 | 313 | Formylation | PEAKS PTM |
| S.GG(sub A)PTIIVER.G | 31.86 | 940.5342 | 9 | -2.4 | 471.2732 | 2 | 62.8 | 122 | 130 |  | SPIDER |
| S.D(sub G)SGD(sub A)PTIIVER.G | 31.72 | 1200.599 | 11 | -1.4 | 601.3057 | 2 | 67.5 | 120 | 130 |  | SPIDER |
| A.AAAAAAAAAGGAGSGYGGGSW(+43.99).H | 31.71 | 1694.728 | 21 | 1.8 | 848.373 | 2 | 48.9 | 419 | 439 | Carboxylation (DKW) | PEAKS PTM |
| G.SDSGAAAAAAAAAAAAAGGAG.D | 31.59 | 1529.707 | 21 | -2.1 | 765.8591 | 2 | 66.3 | 1044 | 1064 |  | PEAKS DB |
| S.G(+127.06)APTIIVER.G | 31.51 | 1081.613 | 9 | -1.7 | 541.8129 | 2 | 56.6 | 122 | 130 | N-Succinimidyl-2-morpholine acetate | PEAKS PTM |
| S.M(sub G)SGAPTIIVER.G | 31.48 | 1172.622 | 11 | 14.1 | 587.3267 | 2 | 51.1 | 120 | 130 |  | SPIDER |
| S.GAPTIIVER.G | 31.47 | 954.5498 | 9 | -3.2 | 478.2807 | 2 | 50.5 | 122 | 130 |  | PEAKS DB |
| D.S(+42.01)SAAAAAAAAAAGGAG.G | 31.39 | 1186.558 | 16 | -1.9 | 594.285 | 2 | 74.3 | 937 | 952 | Acetylation (N-term) | PEAKS PTM |
| R.APTGHETI.Y | 31.38 | 824.4028 | 8 | -2.1 | 413.2078 | 2 | 44.6 | 85 | 92 |  | PEAKS DB |
| S.DNAAAAAAAAAAAAGGAG(+15.99).G | 31.36 | 1357.622 | 18 | -1.9 | 679.8171 | 2 | 73.3 | 2774 | 2791 | Oxidation or Hydroxylation | PEAKS DB |
| S.DNAAAAAAAAAAAAGG(+21.98).A | 31.3 | 1235.551 | 16 | -2.2 | 618.7812 | 2 | 74.4 | 2774 | 2789 | Sodium adduct | PEAKS PTM |
| S.E(sub A)S(sub A)AAAAAAAAAAAGGAGGGAGR.G | 31.29 | 1655.798 | 22 | -2 | 828.9044 | 2 | 67.2 | 2740 | 2761 |  | SPIDER |
| D.GGAAAAAAAAAAAAAGGAGGGY(+44.99)GG.G | 31.15 | 1733.772 | 24 | -1.8 | 867.8915 | 2 | 68.5 | 380 | 403 | Oxidation to nitro | PEAKS PTM |
| A.P(+27.99)TIIVER.G | 31.01 | 854.4861 | 7 | -2.4 | 428.2493 | 2 | 63.6 | 124 | 130 | Formylation | PEAKS PTM |
| G.E(sub A)PTIIVER.G | 30.94 | 955.5338 | 8 | -2.9 | 478.7728 | 2 | 62.9 | 123 | 130 |  | SPIDER |
| A.AE(sub G)AAAAAAAAAAAAGGAGR.G | 30.87 | 1468.738 | 19 | -2.1 | 735.3748 | 2 | 72 | 146 | 164 |  | SPIDER |
| D.I(+43.01)HSLER.G | 30.73 | 796.4191 | 6 | -2 | 399.216 | 2 | 47.6 | 25 | 30 | Carbamylation | PEAKS PTM |
| G.G(+58.01)AAAAAAAAAAAAAGGAGR.G | 30.69 | 1454.723 | 19 | -1.5 | 728.3674 | 2 | 68.9 | 296 | 314 | Carboxymethyl (KW, X@N-term) | PEAKS PTM |
| S.AEAAAAAAAAAAAAG.T | 30.68 | 1127.557 | 15 | -1.4 | 564.785 | 2 | 83 | 234 | 248 |  | PEAKS DB |
| E.TW(sub I)YEEDVVIK.Q | 30.66 | 1280.629 | 10 | -13 | 641.3134 | 2 | 74 | 91 | 100 |  | SPIDER |
| D.S(+27.99)GAAAAAAAAAAAAAGGAEGG.Y | 30.48 | 1541.707 | 21 | -2.7 | 771.8586 | 2 | 79.6 | 2103 | 2123 | Formylation | PEAKS PTM |
| S.DSAAAAAAAAAAAAAGGAGG.G | 30.12 | 1442.675 | 20 | -2.6 | 722.3428 | 2 | 78.4 | 2738 | 2757 |  | PEAKS DB |
| G.GAAAAAAAAAAAAAGGAGSG.Y | 30.1 | 1384.669 | 20 | -2 | 693.3406 | 2 | 59.8 | 414 | 433 |  | PEAKS DB |
| A.AAAAAAAGGAGSGYGGGSW(+43.99).H | 30.02 | 1552.654 | 19 | 3.2 | 777.3369 | 2 | 46.8 | 421 | 439 | Carboxylation (DKW) | PEAKS PTM |
| D.N(+.98)AAAAAAAAAAAAGGA.G | 30.02 | 1170.563 | 16 | -1.8 | 586.2877 | 2 | 78.5 | 2775 | 2790 | Deamidation (NQ) | PEAKS DB |
| D.GGAAAAAAAAAAAAAGGSGGGY(+44.99)G.G | 29.86 | 1692.745 | 23 | -2.1 | 847.3781 | 2 | 67.4 | 472 | 494 | Oxidation to nitro | PEAKS PTM |
| CHYMOTRYPSIN |  |  |  |  |  |  |  |  |  |  |  |
| Peptide | **-10lgP** | **Mass** | **Length** | **ppm** | **m/z** | **z** | **RT** | **Start** | **End** | **PTM** | **Found By** |
| F.ERDSAPSRVPGGGTLVEKIVIERAPTGHETIY.E | 78.89 | 3433.795 | 32 | 3.3 | 859.4589 | 4 | 81.1 | 62 | 93 |  | PEAKS DB |
| F.ERDSAPSRVPGGGTLVEKIVIERAPTGH.E | 77.22 | 2927.558 | 28 | 3.8 | 732.8995 | 4 | 75.3 | 62 | 89 |  | PEAKS DB |
| F.ERDSAPSRVPGGGTLVEKIVIERAPTGHET.I | 73.04 | 3157.648 | 30 | 3.6 | 632.5391 | 5 | 75 | 62 | 91 |  | PEAKS DB |
| Y.GGGYGGDGGAAAAAAAAAAAAAGGSRSGY.G | 72.93 | 2283.026 | 29 | 3.7 | 1142.525 | 2 | 78.1 | 1006 | 1034 |  | PEAKS DB |
| S.RVPGGGTLVEKIVIERAPTGHETIY.E | 72.39 | 2691.471 | 25 | 3.3 | 898.1672 | 3 | 81.1 | 69 | 93 |  | PEAKS DB |
| Y.GRGHGTAGSAAAAAAAAAAAAASSEGGSAGGY.W | 72.18 | 2574.181 | 32 | 4.4 | 859.0713 | 3 | 74.8 | 171 | 202 |  | PEAKS DB |
| Y.GGGYGSDNAAAAAAAAAAAAGGAGGDYGRGY.G | 71.8 | 2588.127 | 31 | 4.6 | 1295.077 | 2 | 78.2 | 2768 | 2798 |  | PEAKS DB |
| Y.GRGHGTAGSAAAAAAAAAAAAASSEGGSAGGYW.Q | 71.47 | 2760.26 | 33 | 3.8 | 921.0975 | 3 | 84.2 | 171 | 203 |  | PEAKS DB |
| Y.GHGYGSDGGAAAAAAAAAAAAAGGSGGGY.G | 69.98 | 2263.984 | 29 | 3.1 | 1133.003 | 2 | 76.6 | 575 | 603 |  | PEAKS DB |
| Y.GHGYGSDSAAAAAAAAAAAAAGGAGGGAGRGAIGGY.G | 69.45 | 2860.324 | 36 | 4.4 | 954.4526 | 3 | 80.5 | 2732 | 2767 |  | PEAKS DB |
| Y.GSDSDAAAAAAAAASAAAGGAGSGY.G | 69.35 | 1967.846 | 25 | 4.3 | 984.9343 | 2 | 75.1 | 2668 | 2692 |  | PEAKS DB |
| Y.GHGYGSDGGAAAAAAAAAAAAAGGAGGGY.G | 69.3 | 2247.989 | 29 | 3.7 | 1125.006 | 2 | 78.5 | 373 | 401 |  | PEAKS DB |
| F.ERDSAPSRVPGGGTLVEKIVIERAPT.G | 68.94 | 2733.477 | 26 | 4 | 547.7049 | 5 | 78.2 | 62 | 87 |  | PEAKS DB |
| Y.GGGYGHGYGSDGGAAAAAAAAAAAAAGGAGRGY.G | 68.26 | 2681.197 | 33 | 3.5 | 894.7426 | 3 | 79.3 | 284 | 316 |  | PEAKS DB |
| Y.GGDGGAAAAAAAAAAAAAGGSRSGY.G | 68.03 | 1948.899 | 25 | 3.8 | 975.4603 | 2 | 74.7 | 1010 | 1034 |  | PEAKS DB |
| Y.GSDSGAAAAAAAAAAAASGGAGSGY.G | 67.44 | 1909.84 | 25 | 4.6 | 955.9317 | 2 | 74.1 | 2385 | 2409 |  | PEAKS DB |
| Y.GSDNAAAAAAAAAAAAGGAGGDYGRGY.G | 66.27 | 2254 | 27 | 4.6 | 1128.012 | 2 | 75.1 | 2772 | 2798 |  | PEAKS DB |
| Y.GSDGGAAAAAAAAAAAAAGGAGRGY.G | 66 | 1932.904 | 25 | 2.8 | 967.4619 | 2 | 77.6 | 292 | 316 |  | PEAKS DB |
| Y.GRDGGAAAAAAAAAAAAAGGAGSGY.G | 65.47 | 1932.904 | 25 | 4 | 967.463 | 2 | 74.7 | 2466 | 2490 |  | PEAKS DB |
| Y.GSDSAEAAAAAAAAAAAAGTAAGGSGGGY.G | 63.74 | 2251.994 | 29 | 5 | 1127.01 | 2 | 80.6 | 230 | 258 |  | PEAKS DB |
| Y.GHGYGSDGGAAAAAAAAAAAAAGGAGSGY.G | 63.18 | 2278 | 29 | 3.4 | 760.3431 | 3 | 78.4 | 1802 | 1830 |  | PEAKS DB |
| Y.GSDSAAAAAAAAAAAAAGGAGGGAGRGAIGGY.G | 63.13 | 2446.158 | 32 | 3.8 | 816.3965 | 3 | 80 | 2736 | 2767 |  | PEAKS DB |
| Y.GSNSGAAAAAAAAAAAGGAGSGYGGGARGGY.G | 63.01 | 2426.096 | 31 | 4.1 | 809.7092 | 3 | 68.7 | 2701 | 2731 |  | PEAKS DB |
| L.VEKIVIERAPTGHETIY.E | 62.42 | 1954.052 | 17 | 3.4 | 978.0368 | 2 | 66.8 | 77 | 93 |  | PEAKS DB |
| G.SGRSHGAGSAAGAAAAAAAAAAAAGGAGRGGGGGY.G | 61.97 | 2712.283 | 35 | 3.9 | 905.105 | 3 | 72.2 | 136 | 170 |  | PEAKS DB |
| W.HGYGSDSDAAAAAAAAASAAAGGAGSGY.G | 61.47 | 2324.989 | 28 | 4.3 | 1163.507 | 2 | 75.5 | 2665 | 2692 |  | PEAKS DB |
| Y.GSDSGAAAAAAAAAAAAAGGAGSGYGGGSRGGY.G | 61.1 | 2585.149 | 33 | 3.6 | 862.7267 | 3 | 76.5 | 2569 | 2601 |  | PEAKS DB |
| Y.GYGSDSSAAAAAAAAAAGGAGGGY.G | 60.99 | 1900.819 | 24 | 4.3 | 951.4207 | 2 | 70 | 932 | 955 |  | PEAKS DB |
| Y.GSDSGAAAAAAAAAAAAAGGAGSGY.G | 60.95 | 1893.845 | 25 | 3.7 | 947.9333 | 2 | 77.2 | 2569 | 2593 |  | PEAKS DB |
| Y.GGDGGAAAAAAAAAAAAAGGAGSGYGGGARRGY.G | 60.51 | 2608.213 | 33 | 4.5 | 870.4154 | 3 | 75.5 | 542 | 574 |  | PEAKS DB |
| H.GAGSAAGAAAAAAAAAAAAGGAGRGGGGGY.G | 59.94 | 2188.037 | 30 | 4 | 730.3558 | 3 | 77.1 | 141 | 170 |  | PEAKS DB |
| Y.GGGYGSDNAAAAAAAAAAAAGGAGGDY.G | 59.44 | 2154.92 | 27 | 3.8 | 1078.471 | 2 | 77.4 | 2768 | 2794 |  | PEAKS DB |
| Y.EEDVVIKQVPQGGAASSAAS.S | 57.58 | 1941.964 | 20 | 3.1 | 971.9924 | 2 | 58.5 | 94 | 113 |  | PEAKS DB |
| Y.EEDVVIKQVPQGGAASSAA.S | 57.43 | 1854.932 | 19 | 4.3 | 928.4774 | 2 | 59.8 | 94 | 112 |  | PEAKS DB |
| Y.GSDSGAAAAAAAAAAGAAGGAGGGY.G | 57.02 | 1849.819 | 25 | 3.9 | 617.616 | 3 | 70.7 | 2418 | 2442 |  | PEAKS DB |
| Y.GGDGGAAAAAAAAAAAAAGGAGGGY.G | 56.72 | 1803.814 | 25 | 4.6 | 902.9182 | 2 | 77.7 | 728 | 752 |  | PEAKS DB |
| Y.GGGYGGDGGAAAAAAAAAAAAAGGSGSGY.G | 56.33 | 2183.947 | 29 | 4.2 | 1092.985 | 2 | 78.9 | 2631 | 2659 |  | PEAKS DB |
| Y.GSNSGAAAAAAAAAAAGGAGSGY.G | 56.1 | 1750.787 | 23 | 4.4 | 876.4047 | 2 | 67.7 | 2701 | 2723 |  | PEAKS DB |
| A.AAGGAGGGAGRGAIGGY.G | 55.84 | 1318.638 | 17 | 3.2 | 660.3282 | 2 | 51.6 | 2751 | 2767 |  | PEAKS DB |
| F.ERDSAPSRVPGGGTLVEK.I | 55.74 | 1853.96 | 18 | 3.3 | 464.4987 | 4 | 52.9 | 62 | 79 |  | PEAKS DB |
| Y.EEDVVIKQVPQGGAAS.S | 55.52 | 1625.826 | 16 | 4.3 | 813.9238 | 2 | 58.1 | 94 | 109 |  | PEAKS DB |
| Y.GSDGGAAAAAAAAAAAAAGGSGGGY.G | 55.49 | 1849.819 | 25 | 4.7 | 925.9211 | 2 | 75.2 | 579 | 603 |  | PEAKS DB |
| Y.GHGYGSDGGAAAAAAAAAAAAAGGAGGGYGGGY.G | 55.29 | 2582.117 | 33 | 3.6 | 861.716 | 3 | 80.1 | 373 | 405 |  | PEAKS DB |
| Y.GSDSSAAAAAAAAAAAAAGGAGGGY.G | 55.2 | 1893.845 | 25 | 3.9 | 947.9335 | 2 | 78.1 | 1185 | 1209 |  | PEAKS DB |
| Y.GSDGGAAAAAAAAAAAAAGGAGGGYGGGY.G | 55.2 | 2167.952 | 29 | 4.7 | 1084.988 | 2 | 79.3 | 377 | 405 |  | PEAKS DB |
| Y.GGDGGAAAAAAAAAAAAAGGAGSGY.G | 55.19 | 1833.824 | 25 | 4.1 | 917.9231 | 2 | 77.2 | 410 | 434 |  | PEAKS DB |
| Y.GARSAAAAAAAAAASSGARGAVRVHETGDGF.L | 55.04 | 2783.381 | 31 | 3.8 | 696.8552 | 4 | 69.4 | 2799 | 2829 |  | PEAKS DB |
| Y.GSDGGAAAAAAAAAAAAAGGAGSGY.G | 54.51 | 1863.835 | 25 | 4.1 | 932.9284 | 2 | 77.3 | 1806 | 1830 |  | PEAKS DB |
| Y.GSDNAAAAAAAAAAAAGGAGGDY.G | 54.38 | 1820.793 | 23 | 4.4 | 911.4075 | 2 | 73.7 | 2772 | 2794 |  | PEAKS DB |
| Y.GGDGGAAAAAAAAAAAAASGAGSGY.G | 53.87 | 1863.835 | 25 | 3.7 | 932.928 | 2 | 79.6 | 259 | 283 |  | PEAKS DB |
| A.RSLDDDIHSLERGY.R | 53.32 | 1674.796 | 14 | 3.8 | 559.2748 | 3 | 69 | 19 | 32 |  | PEAKS DB |
| Y.GSDGGAAAAAAAAAAAAAGSAESSY.G | 53.12 | 1995.877 | 25 | 4 | 998.9498 | 2 | 80.8 | 901 | 925 |  | PEAKS DB |
| Y.GGDGGAAAAAAAAAAAAGGAGSGY.G | 52.76 | 1762.787 | 24 | 4.3 | 882.4045 | 2 | 73.3 | 1153 | 1176 |  | PEAKS DB |
| Y.RETSKTGYDEYDVDKSGRLY.E | 52.69 | 2381.114 | 20 | 4.1 | 596.2881 | 4 | 60.1 | 33 | 52 |  | SPIDER |
| Y.RETSKTGYDEY.D | 52.53 | 1347.594 | 11 | 3.5 | 674.8068 | 2 | 42.5 | 33 | 43 |  | PEAKS DB |
| S.RVPGGGTLVEKIVIERAPTGHET.I | 52.43 | 2415.323 | 23 | 3.5 | 604.8402 | 4 | 74.2 | 69 | 91 |  | PEAKS DB |
| A.AAAAGGAGGGAGRGAIGGY.G | 52.39 | 1460.712 | 19 | 3.6 | 731.3658 | 2 | 53 | 2749 | 2767 |  | PEAKS DB |
| Y.GSDSGAAAAAAAAAAAAAAGGSGGGY.G | 52.09 | 1950.867 | 26 | 3.8 | 976.4443 | 2 | 78.1 | 443 | 468 |  | PEAKS DB |
| S.RVPGGGTLVEKIVIERAPTGH.E | 52.05 | 2185.233 | 21 | 3.1 | 547.3173 | 4 | 74.6 | 69 | 89 |  | PEAKS DB |
| Y.EEDVVIKQVPQG.G | 51.86 | 1339.698 | 12 | 3.7 | 670.8589 | 2 | 59.5 | 94 | 105 |  | PEAKS DB |
| A.GSGRSHGAGSAAGAAAAAAAAAAAAGGAGRGGGGGY.G | 51.77 | 2769.304 | 36 | 3.7 | 924.112 | 3 | 72.4 | 135 | 170 |  | PEAKS DB |
| G.HGYGS(+114.04)DSAAAAAAAAAAAAAGGAGGGAGRGAIGGY.G | 51.41 | 2917.345 | 35 | 4.2 | 973.4597 | 3 | 80.5 | 2733 | 2767 | Ubiquitin | PEAKS PTM |
| F.ERDSAPSRVPGGGT(-18.01)L.V | 51.28 | 1479.743 | 15 | 3.6 | 494.2567 | 3 | 55.2 | 62 | 76 | Dehydration | PEAKS PTM |
| L.GTSKIAVH | 51.11 | 811.4552 | 8 | 4.8 | 406.7368 | 2 | 35.6 | 2873 | 2880 |  | PEAKS DB |
| Y.GGDGGAAAAAAAAAAAAAGGAGSGY(+21.98).G | 50.76 | 1855.806 | 25 | 3 | 619.6111 | 3 | 77.6 | 410 | 434 | Sodium adduct | PEAKS PTM |
| A.AGGAGGGAGRGAIGGY.G | 50.62 | 1247.601 | 16 | 3.5 | 624.8098 | 2 | 51.3 | 2752 | 2767 |  | PEAKS DB |
| Y.GGGARGGYGHGYGSDGGAAAAAAAAAAAAAGGAGSGY.G | 50.51 | 2953.309 | 37 | 3.2 | 985.4466 | 3 | 77.9 | 1794 | 1830 |  | PEAKS DB |
| D.SAPSRVPGGGTLVEKIVIERAPTGHETIY.E | 49.9 | 3033.625 | 29 | 4.2 | 759.4166 | 4 | 82.2 | 65 | 93 |  | PEAKS DB |
| T.GHETIYEEDVVIKQVPQ.G | 49.86 | 1982.995 | 17 | 3.7 | 662.008 | 3 | 71.6 | 88 | 104 |  | PEAKS DB |
| Y.GAGSAAAAAAAAAAAAGGSGGGY.G | 49.68 | 1677.771 | 23 | 4 | 839.8959 | 2 | 71.9 | 317 | 339 |  | PEAKS DB |
| Y.GGDGGAAAAAAAAAAAAAAGGAGSGY.G | 49.32 | 1904.861 | 26 | 3.9 | 953.4416 | 2 | 80.2 | 801 | 826 |  | PEAKS DB |
| A.AAAAAAGGAGGDYGRGY.G | 49.24 | 1454.654 | 17 | 2.3 | 728.3359 | 2 | 49.6 | 2782 | 2798 |  | PEAKS DB |
| Y.R(+43.01)ETSKTGYDEY.D | 49.12 | 1390.6 | 11 | 3.2 | 696.3096 | 2 | 47.9 | 33 | 43 | Carbamylation | PEAKS PTM |
| R.GAVRVHETGDGF.L | 48.77 | 1243.595 | 12 | 2.8 | 622.8063 | 2 | 52.7 | 2818 | 2829 |  | PEAKS DB |
| Y.GAGSAAAAAAAAAAAAGGAGGGY.G | 48.43 | 1661.776 | 23 | 4.5 | 831.8988 | 2 | 74 | 519 | 541 |  | PEAKS DB |
| A.AAAAAAGGAGGGAGRGAIGGY.G | 48.29 | 1602.786 | 21 | 4.2 | 802.4037 | 2 | 54.5 | 2747 | 2767 |  | PEAKS DB |
| S.RVPGGGTLVEKIVIERAPT.G | 48.09 | 1991.153 | 19 | 3.6 | 498.7972 | 4 | 77.6 | 69 | 87 |  | PEAKS DB |
| Y.EEDVVIKQVPQGGAASSAASSASAGSGSGAPT(+21.98).I | 47.63 | 2894.351 | 32 | 4.8 | 965.7954 | 3 | 60.9 | 94 | 125 | Sodium adduct | PEAKS PTM |
| Y.DEYDVDKSGRLY.E | 47.58 | 1458.663 | 12 | 3.8 | 730.3414 | 2 | 59.7 | 41 | 52 |  | PEAKS DB |
| Y.GGDGGAAAAAAAAAAAAAGSGGGY.G | 47.54 | 1762.787 | 24 | 2.5 | 882.403 | 2 | 76.4 | 2148 | 2171 |  | PEAKS DB |
| Y.GSDSGAAAAAAAAAAAAAGGAGGGY.G | 47.49 | 1863.835 | 25 | 3.8 | 932.9281 | 2 | 76.5 | 1982 | 2006 |  | PEAKS DB |
| T.IYEEDVVIKQVPQ.G | 47.43 | 1558.824 | 13 | 4.1 | 780.4226 | 2 | 69.7 | 92 | 104 |  | PEAKS DB |
| Y.GSDNAAAAAAAAAAAAGGAGGD(+21.98)Y.G | 47.15 | 1842.774 | 23 | 4.8 | 922.3989 | 2 | 74 | 2772 | 2794 | Sodium adduct | PEAKS PTM |
| Y.GSDGGAAAAAAAAAAAAAGGAGGGY.G | 46.91 | 1833.824 | 25 | 4 | 917.923 | 2 | 77.3 | 377 | 401 |  | PEAKS DB |
| K.IVIERAPTGHETIY.E | 46.83 | 1597.846 | 14 | 4.9 | 799.9343 | 2 | 65.6 | 80 | 93 |  | PEAKS DB |
| Y.DVDKSGRLY.E | 46.75 | 1051.53 | 9 | 2.8 | 526.7736 | 2 | 51.8 | 44 | 52 |  | PEAKS DB |
| G.GTLVEKIVIERAPTGHETIY.E | 46.72 | 2225.206 | 20 | 3.2 | 742.7449 | 3 | 79.2 | 74 | 93 |  | PEAKS DB |
| A.AAAGGAGGDYGRGY.G | 46.58 | 1241.543 | 14 | 4.3 | 621.7812 | 2 | 46.3 | 2785 | 2798 |  | PEAKS DB |
| L.DDDIHS(+79.97)LERGY.R | 46.32 | 1398.545 | 11 | 2.9 | 700.282 | 2 | 61.6 | 22 | 32 | Phosphorylation (STY) | PEAKS PTM |
| Y.GGGYGGDGGAAAAAAAAAAAAAGGAGSGY.G | 46.32 | 2167.952 | 29 | 3.9 | 723.6606 | 3 | 80.9 | 406 | 434 |  | PEAKS DB |
| G.S(+114.04)DSAAAAAAAAAAAAAGGAGGGAGRGAIGGY.G | 46.26 | 2503.18 | 31 | 3.7 | 835.4037 | 3 | 80.3 | 2737 | 2767 | Ubiquitin | PEAKS PTM |
| Y.GSDGGAAAAAAAAAAAAAGGSGGGY(+21.98).G | 46.16 | 1871.801 | 25 | 4.4 | 936.9119 | 2 | 75.6 | 579 | 603 | Sodium adduct | PEAKS PTM |
| F.E(-18.01)RDSAPSRVPGGGTL.V | 46.1 | 1479.743 | 15 | 3 | 740.8809 | 2 | 58.9 | 62 | 76 | Pyro-glu from E | PEAKS PTM |
| E.KIVIERAPTGHETIY.E | 46 | 1725.941 | 15 | 3.9 | 863.9813 | 2 | 62.9 | 79 | 93 |  | PEAKS DB |
| Y.GGGARGGYGHGYGSDGGAAAAAAAAAAAAAGGSGGGY.G | 45.97 | 2939.293 | 37 | 1.6 | 980.7732 | 3 | 76.3 | 629 | 665 |  | PEAKS DB |
| Y.GGDGGAAAAAAAAAAAAAGGSGSGY.G | 45.95 | 1849.819 | 25 | 4.1 | 617.6161 | 3 | 75.5 | 2635 | 2659 |  | PEAKS DB |
| Y.GSNSGAAAAAAAAAAAGGAGSGY(+21.98).G | 45.89 | 1772.769 | 23 | 4 | 887.3953 | 2 | 67.9 | 2701 | 2723 | Sodium adduct | PEAKS PTM |
| A.RSLDDDIHS(+79.97)LERGY.R | 45.87 | 1754.763 | 14 | 3.9 | 878.3919 | 2 | 78.2 | 19 | 32 | Phosphorylation (STY) | PEAKS PTM |
| A.AAAAAGGAGGGAGRGAIGGY.G | 45.77 | 1531.749 | 20 | 3.6 | 766.8846 | 2 | 53.6 | 2748 | 2767 |  | PEAKS DB |
| Y.GGDGGAAAAAAAAAAAAGGAGSGY(+21.98).G | 45.48 | 1784.769 | 24 | 4.2 | 893.3955 | 2 | 73.4 | 1153 | 1176 | Sodium adduct | PEAKS PTM |
| A.AAAAGGAGGDYGRGY.G | 45.16 | 1312.58 | 15 | 3.7 | 657.2995 | 2 | 47.4 | 2784 | 2798 |  | PEAKS DB |
| Y.GSDSGAAAAAAAAAAGAAGGAGGGY(+21.98).G | 45.01 | 1871.801 | 25 | 4 | 936.9114 | 2 | 70.8 | 2418 | 2442 | Sodium adduct | PEAKS PTM |
| Y.EEDVVIKQVPQGGAASSA.A | 44.98 | 1783.895 | 18 | 5 | 892.9593 | 2 | 59.2 | 94 | 111 |  | PEAKS DB |
| Y.GGDGGAAAAAAAAAAAAASGAGSGY(+21.98).G | 44.97 | 1885.817 | 25 | 3.9 | 943.9193 | 2 | 79.5 | 259 | 283 | Sodium adduct | PEAKS PTM |
| A.AAAGGAGGGAGRGAIGGY.G | 44.88 | 1389.675 | 18 | -0.8 | 695.8441 | 2 | 52.2 | 2750 | 2767 |  | PEAKS DB |
| Y.G(+43.01)R(+14.02)GHGTAGSAAAAAAAAAAAAASSEGGSAGGY.W | 44.8 | 2631.202 | 32 | 3.8 | 878.078 | 3 | 75 | 171 | 202 | Carbamylation; Methylation(KR) | PEAKS PTM |
| A.AAAAAAAAGGSRSGY.G | 44.58 | 1250.6 | 15 | 3.2 | 626.3094 | 2 | 42.6 | 1020 | 1034 |  | PEAKS DB |
| Y.EEDVVIKQVPQ.G | 44.14 | 1282.677 | 11 | 3 | 642.3477 | 2 | 60.3 | 94 | 104 |  | PEAKS DB |
| A.AAAAAAAGGAGGDYGRGY.G | 44.08 | 1525.691 | 18 | 4.5 | 763.8561 | 2 | 51.2 | 2781 | 2798 |  | PEAKS DB |
| A.AAAAAAAAAGGSRSGY.G | 43.96 | 1321.638 | 16 | 2.8 | 661.8278 | 2 | 46 | 1019 | 1034 |  | PEAKS DB |
| A.AAAAAAAAGGAGGDYGRGY.G | 43.93 | 1596.728 | 19 | 4.1 | 799.3746 | 2 | 53 | 2780 | 2798 |  | PEAKS DB |
| F.ERDSAPSRVPGGGTL.V | 43.71 | 1497.754 | 15 | 3.4 | 500.2601 | 3 | 54.3 | 62 | 76 |  | PEAKS DB |
| Y.GARSAAAAAAAAAASSGARGAVRVH.E | 43.45 | 2177.153 | 25 | 3.3 | 436.4392 | 5 | 61.7 | 2799 | 2823 |  | PEAKS DB |
| Y.EEDVVIKQVPQGGAASSAASSASAGSGSGAPT.I | 43.33 | 2872.369 | 32 | 5.2 | 958.4684 | 3 | 61 | 94 | 125 |  | PEAKS DB |
| R.SLDDDIHS(+79.97)LERGY.R | 42.84 | 1598.661 | 13 | 0.7 | 800.3386 | 2 | 76.2 | 20 | 32 | Phosphorylation (STY) | PEAKS PTM |
| A.AAAAAAAAGGAGRGGGGGY.G | 42.68 | 1432.681 | 19 | 2.5 | 717.3494 | 2 | 44.7 | 152 | 170 |  | PEAKS DB |
| Y.GSDSDAAAAAAAAASAAAGGAGSGY(+21.98).G | 42.52 | 1989.828 | 25 | 4 | 995.925 | 2 | 75.2 | 2668 | 2692 | Sodium adduct | PEAKS PTM |
| Y.GAGSAAAAAAAAAAAAGGAGGGY(+21.98).G | 42.46 | 1683.758 | 23 | 4 | 842.8894 | 2 | 74.2 | 519 | 541 | Sodium adduct | PEAKS PTM |
| Y.GSDGGAAAAAAAAAAAAAGGAGGGY(+21.98).G | 42.44 | 1855.806 | 25 | 3 | 619.6111 | 3 | 77.8 | 377 | 401 | Sodium adduct | PEAKS PTM |
| A.AAAAAAGGAGRGY.G | 42.42 | 1062.521 | 13 | 3.9 | 532.2697 | 2 | 40.5 | 304 | 316 |  | PEAKS DB |
| A.RSLDDDIHSL.E | 42.4 | 1169.568 | 10 | 3.7 | 390.8646 | 3 | 60.8 | 19 | 28 |  | PEAKS DB |
| Y.GSDSSAAAAAAAAAAAGSAGGSY(+21.98).G | 42.03 | 1833.774 | 23 | 3.9 | 917.8979 | 2 | 69.2 | 207 | 229 | Sodium adduct | PEAKS PTM |
| D.GGAAAAAAAAAAAAAGGAGSGYGGGARRGY.G | 41.96 | 2379.143 | 30 | 4.7 | 794.0585 | 3 | 73 | 545 | 574 |  | PEAKS DB |
| Y.GGD(-18.01)GGAAAAAAAAAAAAAGGAGSGY.G | 41.64 | 1815.814 | 25 | 4.4 | 908.918 | 2 | 79.1 | 410 | 434 | Dehydration | PEAKS PTM |
| I.VIERAPTGHETIY.E | 41.56 | 1484.762 | 13 | 3.4 | 495.9297 | 3 | 57 | 81 | 93 |  | PEAKS DB |
| E.RAPTGHETIY.E | 41.42 | 1143.567 | 10 | 3.3 | 572.7928 | 2 | 47.3 | 84 | 93 |  | PEAKS DB |
| A.AAAAAAAAGGAGGGY.G | 41.23 | 1105.515 | 15 | 3.7 | 553.7669 | 2 | 46.6 | 387 | 401 |  | PEAKS DB |
| F.ERDSAPSRVPGGGTLVEK(+43.01)IVIER(+14.02)APTGHETIY.E | 41.14 | 3490.817 | 32 | 2.6 | 699.1724 | 5 | 80.9 | 62 | 93 | Carbamylation; Methylation(KR) | PEAKS PTM |
| F.ERD(-18.01)SAPSRVPGGGTLVEKIVIERAPT.G | 41.08 | 2715.467 | 26 | 3 | 679.876 | 4 | 80.5 | 62 | 87 | Dehydration | PEAKS PTM |
| A.AAAAAAAAAAGGAGSGY.G | 41.06 | 1277.6 | 17 | 4.3 | 639.81 | 2 | 53.9 | 418 | 434 |  | PEAKS DB |
| E.YDVDKSGRLY.E | 40.93 | 1214.593 | 10 | 4.1 | 608.3063 | 2 | 56.7 | 43 | 52 |  | PEAKS DB |
| A.AAAAAAAAAGGAGGGY.G | 40.89 | 1176.552 | 16 | 2.8 | 589.285 | 2 | 49.7 | 386 | 401 |  | PEAKS DB |
| G.AGGGAGRGAIGGY.G | 40.8 | 1062.521 | 13 | 2.6 | 532.269 | 2 | 49.9 | 2755 | 2767 |  | PEAKS DB |
| A.AAAAAAAGGSRSGY.G | 40.79 | 1179.563 | 14 | 3 | 590.7906 | 2 | 40.1 | 1021 | 1034 |  | PEAKS DB |
| A.AAAAAAAGGAGRGY.G | 40.6 | 1133.558 | 14 | 3.6 | 567.7882 | 2 | 43.2 | 303 | 316 |  | PEAKS DB |
| D.GGAAAAAAAAAAAAAGGAGSGY.G | 40.6 | 1604.754 | 22 | 4.4 | 803.3879 | 2 | 74.8 | 413 | 434 |  | PEAKS DB |
| Y.RETSKTGY.D | 39.87 | 940.4614 | 8 | 4.7 | 471.2402 | 2 | 30.2 | 33 | 40 |  | PEAKS DB |
| A.AAAAAAGGAGRGGGGGY.G | 39.86 | 1290.606 | 17 | 3.2 | 646.3126 | 2 | 39.9 | 154 | 170 |  | PEAKS DB |
| L.VEKIVIERAPT.G | 39.81 | 1253.734 | 11 | 3.3 | 418.9201 | 3 | 58.1 | 77 | 87 |  | PEAKS DB |
| Y.G(+43.01)R(+14.02)GHGTAGSAAAAAAAAAAAAASSEGGSAGGYW.Q | 39.67 | 2817.282 | 33 | 3.7 | 940.1046 | 3 | 84.2 | 171 | 203 | Carbamylation; Methylation(KR) | PEAKS PTM |
| Y.RETSKTGYD(-18.01)EY.D | 39.5 | 1329.584 | 11 | 3.2 | 444.2032 | 3 | 43.5 | 33 | 43 | Dehydration | PEAKS PTM |
| S.RVPGGGTLVEKIVIE.R | 39.36 | 1565.914 | 15 | 3.4 | 522.9804 | 3 | 80 | 69 | 83 |  | PEAKS DB |
| Y.DVDKS(+79.97)GRLY.E | 39.28 | 1131.496 | 9 | 2.1 | 566.7565 | 2 | 49 | 44 | 52 | Phosphorylation (STY) | PEAKS PTM |
| F.E(+43.01)RDSAPSRVPGGGTL.V | 38.92 | 1540.759 | 15 | 4.3 | 771.3902 | 2 | 59.2 | 62 | 76 | Carbamylation | PEAKS PTM |
| A.AAAAAAAGGAGGGAGRGAIGGY.G | 38.84 | 1673.823 | 22 | 2 | 837.9205 | 2 | 55.7 | 2746 | 2767 |  | PEAKS DB |
| Y.R(+43.01)(+14.02)ETSKTGYDEY.D | 38.77 | 1404.616 | 11 | 3.6 | 703.3177 | 2 | 42.7 | 33 | 43 | Carbamylation; Methylation(KR) | PEAKS PTM |
| A.AAAAAAAGGAGRGGGGGY.G | 38.65 | 1361.644 | 18 | 4.1 | 681.8318 | 2 | 42.2 | 153 | 170 |  | PEAKS DB |
| A.AAAAAGGAGGDYGRGY.G | 38.62 | 1383.617 | 16 | 0.2 | 692.8157 | 2 | 48.3 | 2783 | 2798 |  | PEAKS DB |
| Y.DVDKSGRLYERL.T | 38.29 | 1449.758 | 12 | 3.7 | 484.2616 | 3 | 60.3 | 44 | 55 |  | PEAKS DB |
| Y.GGDGGAAAAAAAAAAAAAGGAG.S | 38.26 | 1526.707 | 22 | 4.6 | 764.3644 | 2 | 73.1 | 410 | 431 |  | PEAKS DB |
| A.AAAAAAGGSRSGY.G | 38.22 | 1108.526 | 13 | 4.1 | 555.2726 | 2 | 37.8 | 1022 | 1034 |  | PEAKS DB |
| A.AAAAAGGAGRGGGGGY.G | 38.2 | 1219.569 | 16 | 4.2 | 610.7945 | 2 | 38 | 155 | 170 |  | PEAKS DB |
| Y.GARS(+114.04)AAAAAAAAAASSGARGAVRVHETGDGF.L | 38.12 | 2897.424 | 31 | 3.8 | 725.366 | 4 | 73.4 | 2799 | 2829 | Ubiquitin | PEAKS PTM |
| A.AAAAAAAAGGAGSGY.G | 38.07 | 1135.526 | 15 | 3.6 | 568.7722 | 2 | 46.8 | 420 | 434 |  | PEAKS DB |
| Y.GGD(+21.98)GGAAAAAAAAAAAAAGGSRSGY.G | 37.9 | 1970.881 | 25 | 3.1 | 657.9695 | 3 | 74.8 | 1010 | 1034 | Sodium adduct | PEAKS PTM |
| D.SAPSRVPGGGTL.V | 37.83 | 1097.583 | 12 | 2.8 | 549.8002 | 2 | 55.9 | 65 | 76 |  | PEAKS DB |
| Y.GRGHGTAGSAAAAAAAAAAAAASSEGGSAGGYW(+3.99).Q | 37.78 | 2764.255 | 33 | 4.1 | 922.4294 | 3 | 82 | 171 | 203 | Tryptophan oxidation to kynurenin | PEAKS PTM |
| R.SLDDDIHSLERGY.R | 37.62 | 1518.695 | 13 | 2.4 | 507.2402 | 3 | 72.2 | 20 | 32 |  | PEAKS DB |
| A.RSLDDDIHS(+79.97)L.E | 37.59 | 1249.534 | 10 | 3.8 | 625.7766 | 2 | 68 | 19 | 28 | Phosphorylation (STY) | PEAKS PTM |
| L.VEKIVIERAPTGH.E | 37.58 | 1447.815 | 13 | 3.9 | 362.9624 | 4 | 54.2 | 77 | 89 |  | PEAKS DB |
| A.AAAAAAAAAGGAGSGY.G | 37.48 | 1206.563 | 16 | 3.1 | 604.2906 | 2 | 49.7 | 419 | 434 |  | PEAKS DB |
| Y.RETSKTGYDEY(-18.01).D | 37.47 | 1329.584 | 11 | 3.3 | 665.8013 | 2 | 49.4 | 33 | 43 | Dehydration | PEAKS PTM |
| L.VEKIVIERAPTGHETIYEEDVVI.K | 37.34 | 2638.385 | 23 | 3.4 | 660.6058 | 4 | 79.7 | 77 | 99 |  | PEAKS DB |
| F.ERDSAPSRVPGGGTLVE.K | 37.33 | 1725.865 | 17 | 4.5 | 863.9434 | 2 | 58.2 | 62 | 78 |  | PEAKS DB |
| L.VEKIVIERAPTG.H | 37.29 | 1310.756 | 12 | 3.7 | 437.9275 | 3 | 58 | 77 | 88 |  | PEAKS DB |
| F.ERDSAPSRVPGGG.T | 37.28 | 1283.622 | 13 | 4.6 | 428.8832 | 3 | 38.9 | 62 | 74 |  | PEAKS DB |
| A.AAAAAGGAGRGY.G | 37.26 | 991.4835 | 12 | 4.8 | 496.7514 | 2 | 38.4 | 305 | 316 |  | PEAKS DB |
| Y.EEDVVIKQVPQGGAASSAASSA.S | 37.26 | 2100.033 | 22 | 4.2 | 1051.028 | 2 | 60.5 | 94 | 115 |  | PEAKS DB |
| L.VEKIVIERAPTGHET.I | 37.19 | 1677.905 | 15 | 2.7 | 560.3104 | 3 | 54.6 | 77 | 91 |  | PEAKS DB |
| Y.RETSKTGYDEYDVDK.S | 37.03 | 1804.812 | 15 | 2.7 | 602.6127 | 3 | 45.3 | 33 | 47 |  | PEAKS DB |
| F.ERD(-18.01)SAPSRVPGGGTL.V | 37.02 | 1479.743 | 15 | 3 | 740.8809 | 2 | 58.8 | 62 | 76 | Dehydration | PEAKS PTM |
| Y.GGDGGAAAAAAAAAAAAAGGSGSGY(+21.98).G | 36.9 | 1871.801 | 25 | 4.6 | 936.912 | 2 | 75.5 | 2635 | 2659 | Sodium adduct | PEAKS PTM |
| Y.RETSKTGYDEY(+125.90).D | 36.84 | 1473.491 | 11 | 2.8 | 492.1723 | 3 | 56 | 33 | 43 | Iodination | PEAKS PTM |
| C.RLGTSKIAVH | 36.62 | 1080.64 | 10 | 2.9 | 361.2218 | 3 | 45.4 | 2871 | 2880 |  | PEAKS DB |
| A.GGGAGRGAIGGY.G | 36.58 | 991.4835 | 12 | 2.5 | 496.7503 | 2 | 49.4 | 2756 | 2767 |  | PEAKS DB |
| G.GAAAAAAAAAAAAAGGAGSGY.G | 36.4 | 1547.733 | 21 | 4 | 774.8768 | 2 | 73 | 414 | 434 |  | PEAKS DB |
| T.SKTGYDEY.D | 36.38 | 961.4028 | 8 | 1.6 | 481.7094 | 2 | 44.5 | 36 | 43 |  | PEAKS DB |
| S.RVPGGGTLVEKIVIERQ(sub A)PTGHETIY.E | 36.2 | 2748.492 | 25 | 3.5 | 688.1328 | 4 | 80.9 | 69 | 93 |  | SPIDER |
| A.AAAAAGGSRSGY.G | 36.05 | 1037.489 | 12 | 4 | 519.7538 | 2 | 35.8 | 1023 | 1034 |  | PEAKS DB |
| A.AAGGAGRGGGGGY.G | 35.84 | 1006.458 | 13 | 5.3 | 504.239 | 2 | 33.7 | 158 | 170 |  | PEAKS DB |
| L.VEK(+43.01)IVIERAPT.G | 35.67 | 1296.74 | 11 | 4.6 | 649.3803 | 2 | 65.7 | 77 | 87 | Carbamylation | PEAKS PTM |
| Y.D(+43.01)VDKSGRLY.E | 35.67 | 1094.536 | 9 | 3.5 | 548.277 | 2 | 53.8 | 44 | 52 | Carbamylation | PEAKS PTM |
| R.VHETGDGF.L | 35.07 | 860.3665 | 8 | 2.7 | 431.1917 | 2 | 45.7 | 2822 | 2829 |  | PEAKS DB |
| Y.D(+27.99)VDKSGRLY.E | 34.98 | 1079.525 | 9 | 1.2 | 540.7703 | 2 | 55.6 | 44 | 52 | Formylation | PEAKS PTM |
| L.VEK(+43.01)IVIERAPTGHETIY.E | 34.85 | 1997.058 | 17 | 3.5 | 666.6956 | 3 | 73.2 | 77 | 93 | Carbamylation | PEAKS PTM |
| Y.EEDVVIKQVPQ(-.98).G | 34.84 | 1281.693 | 11 | 3.9 | 641.8562 | 2 | 57.7 | 94 | 104 | Amidation | PEAKS PTM |
| Y.DVD(-18.01)KSGRLY.E | 34.75 | 1033.519 | 9 | 3.6 | 517.7687 | 2 | 53.3 | 44 | 52 | Dehydration | PEAKS PTM |
| Y.GGGSWHSY.G | 34.65 | 849.3406 | 8 | 3.3 | 425.679 | 2 | 50.9 | 435 | 442 |  | PEAKS DB |
| Y.GSDGGAAAAAAAAAAAAAGGSGGGY(+37.95).G | 34.65 | 1887.766 | 25 | 3.3 | 630.2647 | 3 | 75.7 | 579 | 603 | Replacement of 2 protons by calcium | PEAKS PTM |
| Y.R(+42.01)ETSKTGYDEY.D | 34.6 | 1389.605 | 11 | 3.5 | 695.8121 | 2 | 51.8 | 33 | 43 | Acetylation (N-term) | PEAKS PTM |
| G.S(+114.04)DSGAAAAAAAAAAGAAGGAGGGY.G | 34.52 | 1906.841 | 24 | 3.6 | 954.431 | 2 | 70.8 | 2419 | 2442 | Ubiquitin | PEAKS PTM |
| A.AAAAGGAGRGY.G | 34.48 | 920.4464 | 11 | 4.2 | 461.2324 | 2 | 36.6 | 306 | 316 |  | PEAKS DB |
| A.AAAAAAAAAAAGGAGSGY.G | 34.12 | 1348.637 | 18 | 4.1 | 675.3286 | 2 | 58.3 | 417 | 434 |  | PEAKS DB |
| Y.RETSK(-1.03)TGYDEY.D | 34.1 | 1346.563 | 11 | 3.2 | 674.2908 | 2 | 50.4 | 33 | 43 | Lysine oxidation to aminoadipic semialdehyde | PEAKS PTM |
| A.RSLDDDIG(ins)HSLERGY.R | 33.87 | 1731.818 | 15 | 3.7 | 578.282 | 3 | 75.5 | 19 | 32 |  | SPIDER |
| Y.GGDGGAAAAAAAAAAAAASGAGSGY(+37.95).G | 33.66 | 1901.782 | 25 | 3 | 634.9364 | 3 | 79.6 | 259 | 283 | Replacement of 2 protons by calcium | PEAKS PTM |
| A.AAAAAAGGAGSGY.G | 33.51 | 993.4515 | 13 | 4.4 | 497.7352 | 2 | 42 | 422 | 434 |  | PEAKS DB |
| F.ERDSAPSRVPGG(-.98).G | 33.51 | 1225.616 | 12 | 4.4 | 409.5479 | 3 | 38.3 | 62 | 73 | Amidation | PEAKS PTM |
| Y.GSDSGAAAAAAAAAAGAAGGAGGGY(+37.95).G | 33.28 | 1887.766 | 25 | 3.6 | 630.2649 | 3 | 70.8 | 2418 | 2442 | Replacement of 2 protons by calcium | PEAKS PTM |
| S.RVPGGGTLVEKI.V | 33.26 | 1224.719 | 12 | 3 | 613.3686 | 2 | 66.8 | 69 | 80 |  | PEAKS DB |
| Y.R(+27.99)ETSKTGYDEY.D | 33.08 | 1375.589 | 11 | 3.4 | 688.8042 | 2 | 48 | 33 | 43 | Formylation | PEAKS PTM |
| Y.G(+43.01)AR(+14.02)SAAAAAAAAAASSGARGAVRVHETGDGF.L | 33.05 | 2840.403 | 31 | 3.3 | 711.1103 | 4 | 69.9 | 2799 | 2829 | Carbamylation; Methylation(KR) | PEAKS PTM |
| A.AAAAGGSRSGY.G | 32.67 | 966.4518 | 11 | 3.6 | 484.235 | 2 | 34.1 | 1024 | 1034 |  | PEAKS DB |
| Y.RETSKTGYDE(+21.98)Y.D | 32.67 | 1369.576 | 11 | 3.5 | 685.7977 | 2 | 43.4 | 33 | 43 | Sodium adduct | PEAKS PTM |
| D.DDIHSLERGY.R | 32.6 | 1203.552 | 10 | 3.4 | 402.1927 | 3 | 60.3 | 23 | 32 |  | PEAKS DB |
| Y.GSDSSAAAAAAAAAAGGAGGGY(+21.98).G | 32.58 | 1702.716 | 22 | 5.9 | 852.3702 | 2 | 66.2 | 934 | 955 | Sodium adduct | PEAKS PTM |
| Y.RETSKTN(sub G)YDEYDVDKSGRLY.E | 32.53 | 2438.135 | 20 | 4.6 | 813.7227 | 3 | 60.3 | 33 | 52 |  | SPIDER |
| T.SKTGYDEYDVDKSGRLY.E | 32.4 | 1994.922 | 17 | 3.5 | 499.7395 | 4 | 61.8 | 36 | 52 |  | SPIDER |
| G.GAGRGAIGGY.G | 32.38 | 877.4406 | 10 | 1.7 | 439.7283 | 2 | 48.7 | 2758 | 2767 |  | PEAKS DB |
| Y.GGGARGGY.G | 32.34 | 693.3194 | 8 | 5.2 | 347.6688 | 2 | 30.6 | 365 | 372 |  | PEAKS DB |
| F.ERDSAPSRVPGGGTLVEKIVIE.R | 32.33 | 2308.239 | 22 | 3.8 | 770.423 | 3 | 79.8 | 62 | 83 |  | PEAKS DB |
| A.RSLDDDIH.S | 32.28 | 969.4515 | 8 | 3.6 | 324.159 | 3 | 45.4 | 19 | 26 |  | PEAKS DB |
| Y.R(+43.01)ETSKTGY.D | 32.05 | 983.4672 | 8 | 3.6 | 492.7427 | 2 | 36.2 | 33 | 40 | Carbamylation | PEAKS PTM |
| A.AAAAAAAGGSGGGY.G | 32.05 | 1050.473 | 14 | 4.3 | 526.246 | 2 | 41.7 | 326 | 339 |  | PEAKS DB |
| A.AAAAAAAGGAGSGY.G | 31.99 | 1064.489 | 14 | 0.1 | 533.2516 | 2 | 44.3 | 421 | 434 |  | PEAKS DB |
| Y.GGDGGAAAAAAAAAAAAAGGAGSGY(+17.03).G | 31.97 | 1850.851 | 25 | 3.8 | 926.4361 | 2 | 77.7 | 410 | 434 | Replacement of proton with ammonium ion | PEAKS PTM |
| A.AAAAAAGGAGGGY.G | 31.88 | 963.441 | 13 | 3.7 | 482.7296 | 2 | 41.9 | 389 | 401 |  | PEAKS DB |
| Y.GHGYGSDGGAAAAAA.A | 31.68 | 1231.522 | 15 | 3.5 | 616.7703 | 2 | 41.6 | 288 | 302 |  | PEAKS DB |
| Y.GGGARRGY.G | 31.66 | 792.399 | 8 | 3.5 | 397.2082 | 2 | 29 | 567 | 574 |  | PEAKS DB |
| Y.GSDGGAQ(sub A)AAAAAAAAAAAGGAGGGY.G | 31.63 | 1890.846 | 25 | 3 | 946.4329 | 2 | 77.7 | 377 | 401 |  | SPIDER |
| A.AAAAAAAAAAGGAGGGAGRGAIGGY.G | 31.6 | 1886.935 | 25 | 1.2 | 944.4756 | 2 | 61.5 | 2743 | 2767 |  | PEAKS DB |
| A.AAAAAAAAAGGSGGGY.G | 31.52 | 1192.547 | 16 | 3.8 | 597.2831 | 2 | 47 | 324 | 339 |  | PEAKS DB |
| G.T(+114.04)SKIAVH | 31.51 | 868.4767 | 7 | 4.8 | 435.2477 | 2 | 36.1 | 2874 | 2880 | Ubiquitin | PEAKS PTM |
| Y.DVDK(+14.02)SGRLY.E | 31.46 | 1065.545 | 9 | 1.9 | 533.781 | 2 | 48.9 | 44 | 52 | Methylation(KR) | PEAKS PTM |
| Y.RETSK(+72.02)TGYDEY.D | 31.3 | 1419.615 | 11 | 3.5 | 474.2141 | 3 | 46.1 | 33 | 43 | Carboxyethyl | PEAKS PTM |
| I.ERAPTGHETIY.E | 31.28 | 1272.61 | 11 | 1.5 | 425.2112 | 3 | 49.5 | 83 | 93 |  | PEAKS DB |
| Y.RETS(+100.02)KTGYDEY.D | 31.11 | 1447.61 | 11 | 3.7 | 724.8151 | 2 | 52.5 | 33 | 43 | Methylmalonylation on Serine | PEAKS PTM |
| Y.D(-18.01)VDKSGRLY.E | 30.95 | 1033.519 | 9 | 2 | 345.5143 | 3 | 48.1 | 44 | 52 | Dehydration | PEAKS PTM |
| A.AAAAAAAAAGGAGRGGGGGY.G | 30.92 | 1503.718 | 20 | 3.1 | 752.8685 | 2 | 47.6 | 151 | 170 |  | PEAKS DB |
| A.RSLDDDIHS(-15.99)LERGY.R | 30.87 | 1658.801 | 14 | 3.3 | 553.9428 | 3 | 70.3 | 19 | 32 | Deoxy | PEAKS PTM |
| Y.GSDNAAAAAAAAAAAAGGAGGD(+37.95)Y.G | 30.84 | 1858.739 | 23 | 3.6 | 620.5893 | 3 | 73.9 | 2772 | 2794 | Replacement of 2 protons by calcium | PEAKS PTM |
| Y.DVDKSGRLY(+125.90).E | 30.77 | 1177.426 | 9 | 3.5 | 393.4841 | 3 | 62.6 | 44 | 52 | Iodination | PEAKS PTM |
| F.LLRGDY.G | 30.76 | 735.3915 | 6 | 4.2 | 368.7046 | 2 | 50.5 | 2830 | 2835 |  | PEAKS DB |
| G.S(+114.04)NSGAAAAAAAAAAAGGAGSGY.G | 30.72 | 1807.809 | 22 | 4.2 | 904.9153 | 2 | 67.9 | 2702 | 2723 | Ubiquitin | PEAKS PTM |
| Y.GAGSAAAAAAAAAAAAGGAGGGY(+17.03).G | 30.64 | 1678.802 | 23 | 4.1 | 840.4119 | 2 | 74.2 | 519 | 541 | Replacement of proton with ammonium ion | PEAKS PTM |
| Y.DVDK(+27.99)SGRLY.E | 30.52 | 1079.525 | 9 | 3.1 | 540.7713 | 2 | 51.3 | 44 | 52 | Formylation | PEAKS PTM |
| Y.GHGYGSDGGAAAAA.A | 30.44 | 1160.485 | 14 | 4.5 | 581.2522 | 2 | 39.1 | 288 | 301 |  | PEAKS DB |
| A.AAAAAAAGGAGGGY.G | 30.32 | 1034.478 | 14 | -1.1 | 518.2457 | 2 | 44.2 | 388 | 401 |  | PEAKS DB |
| Y.GGDGGAAAAAAAAAAAAAGGAGSGY(+37.95).G | 30.29 | 1871.771 | 25 | 2.9 | 624.9327 | 3 | 77.5 | 410 | 434 | Replacement of 2 protons by calcium | PEAKS PTM |
| G.S(+114.04)DSSAAAAAAAAAAAGSAGGSY.G | 30.25 | 1868.814 | 22 | 3.6 | 935.4175 | 2 | 69.2 | 208 | 229 | Ubiquitin | PEAKS PTM |
| F.ERDSAPSRVPG(-.98).G | 30.18 | 1168.595 | 11 | 4.3 | 390.5406 | 3 | 38 | 62 | 72 | Amidation | PEAKS PTM |
| A.AAAAASAAAGGAGSGY.G | 30 | 1222.558 | 16 | 3.6 | 612.2883 | 2 | 46.9 | 2677 | 2692 |  | PEAKS DB |
| T.GYDEYDVDKSGRLY.E | 29.97 | 1678.747 | 14 | 3.3 | 840.3838 | 2 | 64.1 | 39 | 52 |  | SPIDER |
| R.VHETGDGFL.L | 29.86 | 973.4505 | 9 | 3.4 | 487.7342 | 2 | 64.8 | 2822 | 2830 |  | PEAKS DB |
| A.AAAASAAAGGAGSGY.G | 29.85 | 1151.521 | 15 | 3.2 | 576.7694 | 2 | 45 | 2678 | 2692 |  | PEAKS DB |
| A.R(+43.01)SLDDDIHSL.E | 29.84 | 1212.574 | 10 | 3.6 | 607.2962 | 2 | 66 | 19 | 28 | Carbamylation | PEAKS PTM |
| F.ERDSAPSRVPGGGTLV.E | 29.82 | 1596.822 | 16 | 3.5 | 533.2831 | 3 | 60.6 | 62 | 77 |  | PEAKS DB |
| G.HGYGS(+114.04)DGGAAAAAAAAAAAAAGGSGGGY.G | 29.71 | 2321.006 | 28 | 2.9 | 1161.513 | 2 | 76.6 | 576 | 603 | Ubiquitin | PEAKS PTM |
| T.G(sub A)RSLDDDIHSLERGY.R | 29.63 | 1731.818 | 15 | 3.9 | 578.282 | 3 | 69.6 | 18 | 32 |  | SPIDER |
| Y.GGSARRGY.G | 29.49 | 822.4096 | 8 | 3.5 | 412.2135 | 2 | 29.1 | 691 | 698 |  | PEAKS DB |
| Y.GHGYGSDGGAAAA.A | 29.34 | 1089.448 | 13 | 4 | 545.7332 | 2 | 36.5 | 288 | 300 |  | PEAKS DB |
| Y.RETSKTGY(+125.90)DEY.D | 29.31 | 1473.491 | 11 | 4.6 | 737.7561 | 2 | 54.2 | 33 | 43 | Iodination | PEAKS PTM |
| F.ERDSAPS(-18.01)RVPGG.G | 29.06 | 1208.59 | 12 | 3.7 | 403.872 | 3 | 39.8 | 62 | 73 | Dehydration | PEAKS PTM |
| Y.GSDGGAAAAAAAAAAA.A | 29.01 | 1172.542 | 16 | 4 | 587.2807 | 2 | 57.6 | 292 | 307 |  | PEAKS DB |
| Y.RETSK(+58.01)TGYDEY.D | 29.01 | 1405.6 | 11 | 3.8 | 469.5423 | 3 | 45.4 | 33 | 43 | Carboxymethyl (KW, X@N-term) | PEAKS PTM |
| L.VEKIVIERQ(sub A)PTGHETIY.E | 28.88 | 2011.074 | 17 | 2.5 | 671.3669 | 3 | 66.5 | 77 | 93 |  | SPIDER |
| Y.GSDSSAAAAAAAAAAAGSAGGSY(+37.95).G | 28.78 | 1849.739 | 23 | 3.5 | 617.5891 | 3 | 69.2 | 207 | 229 | Replacement of 2 protons by calcium | PEAKS PTM |
| R.VHETGDGFLL.R | 28.76 | 1086.535 | 10 | 3.3 | 544.2764 | 2 | 79.6 | 2822 | 2831 |  | PEAKS DB |
| L.TTRKKF.E | 28.66 | 779.4653 | 6 | 4.4 | 390.7416 | 2 | 27.7 | 56 | 61 |  | PEAKS DB |
| G.TSKIAVH | 28.64 | 754.4337 | 7 | 4.7 | 378.2259 | 2 | 34.9 | 2874 | 2880 |  | PEAKS DB |
| Y.GHGYGSDGGAAAAAAAAAA.A | 28.54 | 1515.67 | 19 | 1.8 | 758.8438 | 2 | 57.3 | 288 | 306 |  | PEAKS DB |
| C.R(+72.02)LGTSKIAVH | 28.29 | 1152.662 | 10 | 4.1 | 385.2293 | 3 | 55.1 | 2871 | 2880 | Dihydroxy methylglyoxal adduct | PEAKS PTM |
| T.GHETIYEEDVVIKQVPQGGAASSAASSASAGSGSGAPT.I | 28.28 | 3572.687 | 38 | 4.2 | 1191.908 | 3 | 70.1 | 88 | 125 |  | PEAKS DB |
| Y.EEDVVIKQVPQGG(-.98).A | 28.19 | 1395.736 | 13 | 3.4 | 698.8775 | 2 | 56.5 | 94 | 106 | Amidation | PEAKS PTM |
| Y.RETSK(+27.99)TGY.D | 28.14 | 968.4563 | 8 | 4.6 | 485.2376 | 2 | 39.1 | 33 | 40 | Formylation | PEAKS PTM |
| S.R(+43.01)VPGGGTLVEKIVIERAPTGHETIY.E | 28.1 | 2734.477 | 25 | 3.3 | 684.6287 | 4 | 86.1 | 69 | 93 | Carbamylation | PEAKS PTM |
| Y.DEYD(+37.95)VDKSGRLY.E | 28.08 | 1496.61 | 12 | 4.4 | 499.8793 | 3 | 61.1 | 41 | 52 | Replacement of 2 protons by calcium | PEAKS PTM |
| S.RVPGGGTL.V | 27.93 | 755.429 | 8 | 2.9 | 378.7228 | 2 | 49.2 | 69 | 76 |  | PEAKS DB |
| A.R(+58.01)SLDDDIHSLERGY.R | 27.64 | 1732.802 | 14 | 4 | 578.6101 | 3 | 71.2 | 19 | 32 | Carboxymethyl (KW, X@N-term) | PEAKS PTM |
| Y.RETS(+114.04)KTGYDEY.D | 27.56 | 1461.637 | 11 | 3.8 | 731.8287 | 2 | 43.7 | 33 | 43 | Ubiquitin | PEAKS PTM |
| Y.DVD(+21.98)KSGRLY.E | 27.52 | 1073.512 | 9 | 1 | 537.7637 | 2 | 48.7 | 44 | 52 | Sodium adduct | PEAKS PTM |
| T.LVEKIVIERAPT.G | 27.43 | 1366.818 | 12 | 3.9 | 456.6152 | 3 | 65.3 | 76 | 87 |  | PEAKS DB |
| A.R(+27.99)SLDDDIH.S | 27.4 | 997.4465 | 8 | 3.3 | 499.7321 | 2 | 51.7 | 19 | 26 | Formylation (Protein N-term) | PEAKS PTM |
| Y.GHGYGSDGGAAAAAAAA.A | 27.36 | 1373.596 | 17 | 1 | 687.8059 | 2 | 48.5 | 288 | 304 |  | PEAKS DB |
| Y.GSDSGAAAAAAAAAAAASGGAGSGY(+17.03).G | 26.82 | 1926.867 | 25 | 4.7 | 964.4451 | 2 | 74.2 | 2385 | 2409 | Replacement of proton with ammonium ion | PEAKS PTM |
| D.GGAAAAAAAAAAAAAGGSGGGY.G | 26.45 | 1590.739 | 22 | 4.5 | 796.3801 | 2 | 72.7 | 472 | 493 |  | PEAKS DB |
| Y.GGDGGAAAAAAAAAA.A | 26.35 | 1071.494 | 15 | 3.1 | 536.7561 | 2 | 53.4 | 259 | 273 |  | PEAKS DB |
| Y.GGDGGAAAAAAAAAAAAAGGSGSGY(+37.95).G | 26.3 | 1887.766 | 25 | 3.9 | 630.2651 | 3 | 75.4 | 2635 | 2659 | Replacement of 2 protons by calcium | PEAKS PTM |
| T.GHETIY.E | 26.26 | 718.3286 | 6 | 1.7 | 360.1722 | 2 | 45.6 | 88 | 93 |  | PEAKS DB |
| Y.GGDGGAAAAAAAAA.A | 26.19 | 1000.457 | 14 | 4.6 | 501.2383 | 2 | 47.8 | 259 | 272 |  | PEAKS DB |
| Y.R(+43.01)(+14.02)ETSKTGY.D | 26.09 | 997.4828 | 8 | 6 | 499.7517 | 2 | 30.6 | 33 | 40 | Carbamylation; Methylation(KR) | PEAKS PTM |
| A.AAAAAAGGAGSGYGGSARRG.Y | 26.07 | 1634.787 | 20 | 3.8 | 818.404 | 2 | 59.4 | 678 | 697 |  | PEAKS DB |
| T.S(+43.01)KIAVH | 26.04 | 696.3918 | 6 | 3.6 | 349.2044 | 2 | 41.8 | 2875 | 2880 | Carbamylation | PEAKS PTM |
| Y.GSNSGAAAAAAAAAAAGGAGSGY(+37.95).G | 26.02 | 1788.734 | 23 | 3.7 | 597.2541 | 3 | 68 | 2701 | 2723 | Replacement of 2 protons by calcium | PEAKS PTM |
| Y.RETSK(+42.01)TGY.D | 25.95 | 982.4719 | 8 | 4.8 | 492.2456 | 2 | 40.7 | 33 | 40 | Acetylation (K) | PEAKS PTM |
| H.SLERGY.R | 25.94 | 723.3551 | 6 | 4 | 362.6863 | 2 | 42.7 | 27 | 32 |  | PEAKS DB |
| Y.GSDGGAAAAAAAAAAAAAGGAGGGYG.G | 25.92 | 1890.846 | 26 | 2.8 | 946.4327 | 2 | 77.6 | 377 | 402 |  | PEAKS DB |
| Y.G(+43.01)GGARRGY.G | 25.9 | 835.4048 | 8 | 4.3 | 418.7115 | 2 | 33.4 | 567 | 574 | Carbamylation | PEAKS PTM |
| L.VEKIVIE.R | 25.84 | 828.4956 | 7 | 4 | 415.2567 | 2 | 60.1 | 77 | 83 |  | PEAKS DB |
| A.AAAAAAAAAAGGAGSGY(+21.98).G | 25.79 | 1299.582 | 17 | 3.6 | 650.8006 | 2 | 53.8 | 418 | 434 | Sodium adduct | PEAKS PTM |
| L.VEK(+43.01)IVIER(+14.02)APTGHETIY.E | 25.7 | 2011.074 | 17 | 3.4 | 671.3674 | 3 | 66.7 | 77 | 93 | Carbamylation; Methylation(KR) | PEAKS PTM |
| Y.EEDVVIKQVPQGGAASSAASSASAGSGSGAPTIIY(sub V)ERGSGAGS.G | 25.58 | 3962.909 | 43 | 8 | 991.7425 | 4 | 80.8 | 94 | 136 |  | SPIDER |
| F.ERDSAPSRVPGGGTLVEK(+27.99).I | 25.26 | 1881.954 | 18 | 2.7 | 628.3271 | 3 | 60.5 | 62 | 79 | Formylation | PEAKS PTM |
| Y.GSDGGAAAAAAAAAAAAAGGAG(-.98).R | 25.01 | 1555.734 | 22 | 3.4 | 778.8768 | 2 | 72.6 | 292 | 313 | Amidation | PEAKS PTM |
| Y.GGGSW(+31.99)HSY.G | 24.95 | 881.3304 | 8 | 3.3 | 441.6739 | 2 | 47.4 | 435 | 442 | Dihydroxy | PEAKS PTM |
| T.SKTGYDEY(+125.90).D | 24.94 | 1087.3 | 8 | 3.8 | 544.6591 | 2 | 58.3 | 36 | 43 | Iodination | PEAKS PTM |
| Y.GSDGGAAAAAAAAAAAAAGGSGGGY(+17.03).G | 24.72 | 1866.846 | 25 | 4.6 | 934.4343 | 2 | 75.3 | 579 | 603 | Replacement of proton with ammonium ion | PEAKS PTM |
| Y.GGDGGAAAAAAAAAAAAAGGSGSGY(+17.03).G | 24.65 | 1866.846 | 25 | 4.4 | 934.4342 | 2 | 75.4 | 2635 | 2659 | Replacement of proton with ammonium ion | PEAKS PTM |
| Y.GGDGGAAAAAAAA.A | 24.6 | 929.4202 | 13 | 4.6 | 465.7195 | 2 | 41.3 | 259 | 271 |  | PEAKS DB |
| T.ARSV(sub L)DDDIHSL.E | 24.48 | 1226.589 | 11 | 3.7 | 614.3041 | 2 | 61 | 18 | 28 |  | SPIDER |
| Y.DVDKSGRLY(-18.01).E | 24.45 | 1033.519 | 9 | 3.4 | 517.7686 | 2 | 62.2 | 44 | 52 | Dehydration | PEAKS PTM |
| S.RVPGGGTLVEK.I | 24.34 | 1111.635 | 11 | 3.7 | 556.8268 | 2 | 47.8 | 69 | 79 |  | PEAKS DB |
| A.AAAAAAAAGGAGSGY(+21.98).G | 24.32 | 1157.508 | 15 | 3.7 | 579.7632 | 2 | 46.7 | 420 | 434 | Sodium adduct | PEAKS PTM |
| Y.H(sub R)ETSKTGYDEY.D | 24.31 | 1328.552 | 11 | 3.9 | 665.2859 | 2 | 52.8 | 33 | 43 |  | SPIDER |
| A.AAAAAGGAGSGYGGSARRG.Y | 24.01 | 1563.75 | 19 | 3.6 | 782.8851 | 2 | 53.1 | 679 | 697 |  | PEAKS DB |
| R.ET(-18.01)SKTGYDEY.D | 23.83 | 1173.483 | 10 | 3.1 | 587.7504 | 2 | 50.5 | 34 | 43 | Dehydration | PEAKS PTM |
| C.R(+43.01)LGTSKIAVH | 23.81 | 1123.646 | 10 | 3.1 | 562.8321 | 2 | 53.6 | 2871 | 2880 | Carbamylation | PEAKS PTM |
| A.AAAAAAAAAGGAGSGY(+21.98).G | 23.66 | 1228.545 | 16 | 2.5 | 615.2812 | 2 | 49.7 | 419 | 434 | Sodium adduct | PEAKS PTM |
| Y.DVDKSGR(+72.02)LY.E | 23.63 | 1123.551 | 9 | 2.7 | 562.7842 | 2 | 51.7 | 44 | 52 | Dihydroxy methylglyoxal adduct | PEAKS PTM |
| Y.GARSAAAAAAAAAA.S | 23.61 | 1099.573 | 14 | 2.9 | 550.7955 | 2 | 49.5 | 2799 | 2812 |  | PEAKS DB |
| H.SLERGY(+125.90).R | 23.57 | 849.2518 | 6 | 3.7 | 425.6347 | 2 | 56.5 | 27 | 32 | Iodination | PEAKS PTM |
| Y.RETSKTGYD(+37.95)EY.D | 23.48 | 1385.541 | 11 | 3.5 | 462.8559 | 3 | 43.3 | 33 | 43 | Replacement of 2 protons by calcium | PEAKS PTM |
| G.G(+43.01)DGGAAAAAAAAAAAAAAGGAGSGY.G | 23.46 | 1890.846 | 25 | 2.8 | 946.4327 | 2 | 77.5 | 802 | 826 | Carbamylation | PEAKS PTM |
| F.ERDSAPS(-18.01)RVPG.G | 23.43 | 1151.568 | 11 | 1.1 | 384.8638 | 3 | 39.3 | 62 | 72 | Dehydration | PEAKS PTM |
| A.AAAAAAAAGGSGGGY.G | 23.42 | 1121.51 | 15 | -0.8 | 561.7619 | 2 | 44.2 | 325 | 339 |  | PEAKS DB |
| Y.GGGARGGY(+125.90).G | 23.42 | 819.2161 | 8 | 3.9 | 410.6169 | 2 | 43.3 | 365 | 372 | Iodination | PEAKS PTM |
| K.IVIERAPT.G | 23.04 | 897.5283 | 8 | 3.4 | 449.7729 | 2 | 53.7 | 80 | 87 |  | PEAKS DB |
| A.R(+43.01)SLDDDIH.S | 22.99 | 1012.457 | 8 | 3.2 | 507.2375 | 2 | 46.6 | 19 | 26 | Carbamylation | PEAKS PTM |
| Y.ERLTTRKKF.E | 22.9 | 1177.693 | 9 | 4.7 | 393.5735 | 3 | 39 | 53 | 61 |  | PEAKS DB |
| Y.DVD(+37.95)KSGRLY.E | 22.83 | 1089.477 | 9 | 0.6 | 364.1664 | 3 | 48.3 | 44 | 52 | Replacement of 2 protons by calcium | PEAKS PTM |
| A.RSLDDDIHS(-18.01)L.E | 22.78 | 1151.557 | 10 | 3.5 | 576.7878 | 2 | 67.2 | 19 | 28 | Dehydration | PEAKS PTM |
| Y.GGDGGAAAAAAAAAAAAGGAGSGY(+17.03).G | 22.73 | 1779.814 | 24 | 4.8 | 890.9183 | 2 | 73.4 | 1153 | 1176 | Replacement of proton with ammonium ion | PEAKS PTM |
| A.RSLDD(-18.01)DIH.S | 22.63 | 951.441 | 8 | 3.4 | 318.1553 | 3 | 45.4 | 19 | 26 | Dehydration | PEAKS PTM |
| L.ERGYRETSKTGYDEY.D | 22.5 | 1852.823 | 15 | 3.4 | 618.6169 | 3 | 46.6 | 29 | 43 |  | PEAKS DB |
| A.AAAAAAAAGGAGD(+21.98)GY.G | 22.46 | 1185.503 | 15 | 3.5 | 593.7606 | 2 | 47.6 | 504 | 518 | Sodium adduct | PEAKS PTM |
| Y.GSDSGAAAAAAAAAAGAAGGAGGGY(+17.03).G | 22.3 | 1866.846 | 25 | 4.3 | 934.4341 | 2 | 70.8 | 2418 | 2442 | Replacement of proton with ammonium ion | PEAKS PTM |
| Y.RQ(sub E)TM(sub S)KTGYDEYDVDKSGRLY.E | 22.11 | 2424.138 | 20 | -2.6 | 607.0402 | 4 | 63.4 | 33 | 52 |  | SPIDER |
| A.R(+.98)SLDDDIH.S | 22.08 | 970.4355 | 8 | 1 | 486.2256 | 2 | 51.9 | 19 | 26 | Deamidation (R) | PEAKS PTM |
| Y.DVDK(+43.01)SGR(+14.02)LY.E | 21.95 | 1108.551 | 9 | 3 | 555.2845 | 2 | 48 | 44 | 52 | Carbamylation; Methylation(KR) | PEAKS PTM |
| Y.GSDSGAAAAAAAAAAAAAGGAGSGY(+17.03).G | 21.94 | 1910.872 | 25 | 4.1 | 956.4471 | 2 | 77.3 | 2569 | 2593 | Replacement of proton with ammonium ion | PEAKS PTM |
| A.AAAGGSRSGY.G | 21.91 | 895.4147 | 10 | 4.2 | 448.7165 | 2 | 32.8 | 1025 | 1034 |  | PEAKS DB |
| Y.GHGYGSDGGAAAAAAAAAAAAAG(-.98).G | 21.85 | 1784.819 | 23 | 3.7 | 893.4201 | 2 | 73.1 | 288 | 310 | Amidation | PEAKS PTM |
| A.AAAAAAAAAAASSEGGSAGGYW.Q | 21.84 | 1837.823 | 22 | 2.8 | 919.9214 | 2 | 73.9 | 182 | 203 |  | PEAKS DB |
| G.SGRSHGAGSAAGAAAAA.A | 21.58 | 1368.649 | 17 | 5.4 | 457.2262 | 3 | 35.8 | 136 | 152 |  | PEAKS DB |
| Y.GSDGGAAAAAAAAAAAAAGGAGSGY(+17.03).G | 21.54 | 1880.861 | 25 | 3 | 941.4407 | 2 | 77.8 | 1806 | 1830 | Replacement of proton with ammonium ion | PEAKS PTM |
| Y.GSDGGAAAAAAAAAA.A | 21.51 | 1101.505 | 15 | 1.1 | 551.7604 | 2 | 52.3 | 292 | 306 |  | PEAKS DB |
| Y.GGGYGSDNAAAAAAAA.A | 21.39 | 1293.559 | 16 | 3.3 | 647.7886 | 2 | 51.5 | 2768 | 2783 |  | PEAKS DB |
| A.AASSGARGAVRVHETGDGF.L | 21.25 | 1843.893 | 19 | 1.7 | 615.6392 | 3 | 52.5 | 2811 | 2829 |  | PEAKS DB |
| Q.GGAASSAASSASAGSGSGAPT.I | 21.17 | 1607.702 | 21 | 4.5 | 804.8621 | 2 | 41.2 | 105 | 125 |  | PEAKS DB |
| H.S(+43.01)LERGY.R | 21.14 | 766.361 | 6 | 3.6 | 384.1891 | 2 | 53.4 | 27 | 32 | Carbamylation | PEAKS PTM |
| L.TTRKK(+43.01)F.E | 21.06 | 822.4712 | 6 | 4.4 | 412.2447 | 2 | 38.2 | 56 | 61 | Carbamylation | PEAKS PTM |
| Y.DVDKSGR(+15.99)LY.E | 20.98 | 1067.525 | 9 | 3.3 | 534.7714 | 2 | 39.2 | 44 | 52 | Oxidation or Hydroxylation | PEAKS PTM |
| Y.EEDVVIKQC(sub V)PQ.G | 20.85 | 1286.618 | 11 | 3.8 | 644.3185 | 2 | 57.8 | 94 | 104 |  | SPIDER |
| F.ERDSAPSRVP(-.98).G | 20.75 | 1111.573 | 10 | 2.9 | 556.7956 | 2 | 36.4 | 62 | 71 | Amidation | PEAKS PTM |
| D.VDKSGRLY.E | 20.73 | 936.5029 | 8 | -0.5 | 469.2585 | 2 | 48.4 | 45 | 52 |  | PEAKS DB |
| A.AAAAAAGGSGGGY.G | 20.73 | 979.4359 | 13 | 1.6 | 490.726 | 2 | 39.4 | 327 | 339 |  | PEAKS DB |
| A.R(+54.01)SLDDDIH.S | 20.65 | 1023.462 | 8 | 2.5 | 512.7396 | 2 | 49.7 | 19 | 26 | Methylglyoxal-derived hydroimidazolone | PEAKS PTM |
| Y.REG(sub T)SKTGYDEY.D | 20.59 | 1303.568 | 11 | 3 | 652.7932 | 2 | 43 | 33 | 43 |  | SPIDER |
| A.AAAAGGAGSGY.G | 20.54 | 851.3773 | 11 | 4.1 | 426.6977 | 2 | 38.3 | 424 | 434 |  | PEAKS DB |
| A.AAAAAAGGAGGGY(+21.98).G | 20.53 | 985.4229 | 13 | 3.7 | 493.7206 | 2 | 41.9 | 389 | 401 | Sodium adduct | PEAKS PTM |
| Y.DVDKSGRLY(+37.95).E | 20.52 | 1089.477 | 9 | 2.7 | 545.7471 | 2 | 48 | 44 | 52 | Replacement of 2 protons by calcium | PEAKS PTM |
| A.AAAAAGGAGSGY(+21.98).G | 20.3 | 944.3964 | 12 | 3.7 | 473.2072 | 2 | 40.2 | 423 | 434 | Sodium adduct | PEAKS PTM |
| Y.GGGSW(+3.99)HSY.G | 20.2 | 853.3354 | 8 | 3.7 | 427.6766 | 2 | 46.9 | 435 | 442 | Tryptophan oxidation to kynurenin | PEAKS PTM |
| A.RSLD(-18.01)DDIH.S | 20.14 | 951.441 | 8 | 1.9 | 318.1549 | 3 | 45.6 | 19 | 26 | Dehydration | PEAKS PTM |
| R.SLDDDIH.S | 20.05 | 813.3505 | 7 | 2.8 | 407.6837 | 2 | 47.9 | 20 | 26 |  | PEAKS DB |
| F.ERDSAPSRVPGG.G | 20.03 | 1226.6 | 12 | 6.8 | 614.3116 | 2 | 38.9 | 62 | 73 |  | PEAKS DB |
| A.RSLDDD(+37.95)IH.S | 20.02 | 1007.399 | 8 | 3.5 | 336.808 | 3 | 45.4 | 19 | 26 | Replacement of 2 protons by calcium | PEAKS PTM |
| W.QGYGSNSGAAA.A | 19.93 | 981.4152 | 11 | 2 | 491.7158 | 2 | 42.2 | 2698 | 2708 |  | PEAKS DB |
| V.T(+28.03)ARSLDDDIH.S | 19.8 | 1169.568 | 10 | 2.7 | 390.8642 | 3 | 58.8 | 17 | 26 | Ethylation | PEAKS PTM |
| G.TSK(+383.23)IAVH | 19.79 | 1137.662 | 7 | 4.2 | 380.2295 | 3 | 46.5 | 2874 | 2880 | Ubiquitination | PEAKS PTM |
| Y.GGGARRGYG.H | 19.71 | 849.4205 | 9 | 3.7 | 425.7191 | 2 | 29.1 | 567 | 575 |  | PEAKS DB |
| E.G(sub T)SKTGYDEY.D | 19.68 | 1018.424 | 9 | 2.6 | 510.2208 | 2 | 44.7 | 35 | 43 |  | SPIDER |
| C.R(+42.01)LGT(+79.97)SKIAVH | 19.58 | 1202.617 | 10 | 3.5 | 401.8811 | 3 | 57.9 | 2871 | 2880 | Acetylation (N-term); Phosphorylation (STY) | PEAKS PTM |
| I.KQVPQGGAASSAASSASAGSGSGAPT.I | 19.44 | 2188.036 | 26 | 3.3 | 730.3549 | 3 | 45.1 | 100 | 125 |  | PEAKS DB |
| Y.GGGYGSDNAAAAAAA.A | 19.18 | 1222.521 | 15 | 3.1 | 612.2698 | 2 | 46.6 | 2768 | 2782 |  | PEAKS DB |
| A.AAAAAAAGGAGSGY(+21.98).G | 19.12 | 1086.471 | 14 | 0.7 | 544.2429 | 2 | 44.4 | 421 | 434 | Sodium adduct | PEAKS PTM |
| G.GGARGGY.G | 19.01 | 636.298 | 7 | 5.1 | 319.1579 | 2 | 30.8 | 366 | 372 |  | PEAKS DB |
| CHYMOTRYPSIN-TRYPSIN |  |  |  |  |  |  |  |  |  |  |  |
| Peptide | **-10lgP** | **Mass** | **Length** | **ppm** | **m/z** | **z** | **RT** | **Start** | **End** | **PTM** | **Found By** |
| R.APTGHETIYEEDVVIKQVPQGGAASSAASSASAGSGSGAPTIIVER.G | 200 | 4452.205 | 46 | 5 | 1114.064 | 4 | 77.6 | 85 | 130 |  | PEAKS DB |
| Y.GHGYGSDSAAAAAAAAAAAAAGGAGGGAGRGAIGGY.G | 114.01 | 2860.324 | 36 | 2.5 | 954.4508 | 3 | 80.9 | 2732 | 2767 |  | PEAKS DB |
| Y.EEDVVIKQVPQGGAASSAASSASAGSGSGAPTIIVER.G | 103 | 3482.749 | 37 | 4.4 | 1161.929 | 3 | 72.1 | 94 | 130 |  | PEAKS DB |
| Y.GSDSGAAAAAAAAAAAAAGGAGSGYGGGSR.G | 90.75 | 2308.043 | 30 | 4.2 | 1155.033 | 2 | 73.9 | 2569 | 2598 |  | PEAKS DB |
| K.IVIERAPTGHETIYEEDVVIK.Q | 89.31 | 2410.274 | 21 | 3.9 | 804.4352 | 3 | 73.4 | 80 | 100 |  | PEAKS DB |
| K.QVPQGGAASSAASSASAGSGSGAPTIIVER.G | 87.07 | 2670.321 | 30 | 4.2 | 1336.173 | 2 | 65.7 | 101 | 130 |  | PEAKS DB |
| K.I(+43.01)VIER(+14.02)APTGHETIYEEDVVIK.Q | 86.27 | 2467.296 | 21 | 3.8 | 823.4424 | 3 | 73.5 | 80 | 100 | Carbamylation; Methylation(KR) | PEAKS PTM |
| Y.GSDSAAAAAAAAAAAAAGGAGGGAGR.G | 85.94 | 1927.91 | 26 | 4 | 964.9659 | 2 | 72.4 | 2736 | 2761 |  | PEAKS DB |
| Y.GSDSAAAAAAAAAAAAAGGAGGGAGRGAIGGY.G | 85.6 | 2446.158 | 32 | 1.6 | 816.3947 | 3 | 80.6 | 2736 | 2767 |  | PEAKS DB |
| Y.GSDGGAAAAAAAAAAAAAGGAGR.G | 84.89 | 1712.819 | 23 | 4.4 | 857.4205 | 2 | 72.8 | 292 | 314 |  | PEAKS DB |
| R.SHGAGSAAGAAAAAAAAAAAAGGAGRGGGGGYGR.G | 84.41 | 2625.25 | 34 | 3.5 | 657.3221 | 4 | 71.4 | 139 | 172 |  | PEAKS DB |
| Y.GGGYGGDGGAAAAAAAAAAAAAGGSR.S | 83.46 | 1975.91 | 26 | 4.4 | 988.9664 | 2 | 74.9 | 1006 | 1031 |  | PEAKS DB |
| Y.GGGYGSDNAAAAAAAAAAAAGGAGGDYGR.G | 83.1 | 2368.043 | 29 | 4.4 | 1185.034 | 2 | 75.1 | 2768 | 2796 |  | PEAKS DB |
| Y.GHGYGSDSAAAAAAAAAAAAAGGAGGGAGR.G | 82.76 | 2342.075 | 30 | 3.7 | 781.7017 | 3 | 73.7 | 2732 | 2761 |  | PEAKS DB |
| Y.GRGHGTAGSAAAAAAAAAAAAASSEGGSAGGY.W | 82.41 | 2574.181 | 32 | 4.1 | 859.071 | 3 | 75.1 | 171 | 202 |  | PEAKS DB |
| R.APTGHETIYEEDVVIK.Q | 81.09 | 1799.894 | 16 | 4.3 | 900.9582 | 2 | 69.1 | 85 | 100 |  | PEAKS DB |
| Y.GGDGGAAAAAAAAAAAAAGGSR.S | 79.29 | 1641.782 | 22 | 4.1 | 821.9016 | 2 | 70.4 | 1010 | 1031 |  | PEAKS DB |
| Y.GRDGGAAAAAAAAAAAAAGGAGSGY.G | 79.1 | 1932.904 | 25 | 4.3 | 967.4633 | 2 | 74.9 | 2466 | 2490 |  | PEAKS DB |
| Y.GSDSGAAAAAAAAAAAASGGAGSGY.G | 78.52 | 1909.84 | 25 | 4.8 | 955.9319 | 2 | 74.3 | 2385 | 2409 |  | PEAKS DB |
| Y.GSDGGAAAAAAAAAAAAAGGAGRGY.G | 78.44 | 1932.904 | 25 | 3.5 | 967.4625 | 2 | 78.1 | 292 | 316 |  | PEAKS DB |
| Y.GSDNAAAAAAAAAAAAGGAGGDYGR.G | 78.13 | 2033.915 | 25 | 4.8 | 1017.97 | 2 | 71.2 | 2772 | 2796 |  | PEAKS DB |
| R.GGYGHGYGSDSAAAAAAAAAAAAAGGAGGGAGR.G | 78.11 | 2619.181 | 33 | 4.6 | 874.0716 | 3 | 75.4 | 2729 | 2761 |  | PEAKS DB |
| K.IVIERAPTGHETIY.E | 77.58 | 1597.846 | 14 | 2.7 | 799.9326 | 2 | 65.5 | 80 | 93 |  | PEAKS DB |
| G.DGGAAAAAAAAAAAAAGGAGSGYGGGAR.R | 76.97 | 2117.984 | 28 | 4.3 | 1060.004 | 2 | 74.2 | 544 | 571 |  | PEAKS DB |
| S.SAASSASAGSGSGAPTIIVER.G | 76.41 | 1874.933 | 21 | 5.4 | 938.479 | 2 | 62.9 | 110 | 130 |  | PEAKS DB |
| Y.GHGYGSDGGAAAAAAAAAAAAAGGSGGGY.G | 76.3 | 2263.984 | 29 | 4.6 | 1133.005 | 2 | 76.8 | 575 | 603 |  | PEAKS DB |
| Y.GSDSDAAAAAAAAASAAAGGAGSGY.G | 76.28 | 1967.846 | 25 | 4.3 | 984.9343 | 2 | 75.3 | 2668 | 2692 |  | PEAKS DB |
| Y.GGDGGAAAAAAAAAAAAAGGAGSGYGGGAR.R | 76.07 | 2232.027 | 30 | 3.8 | 1117.025 | 2 | 74.7 | 542 | 571 |  | PEAKS DB |
| W.HGYGSDSDAAAAAAAAASAAAGGAGSGY.G | 75.59 | 2324.989 | 28 | 5.1 | 1163.508 | 2 | 75.8 | 2665 | 2692 |  | PEAKS DB |
| Y.GSNSGAAAAAAAAAAAGGAGSGYGGGAR.G | 75.38 | 2148.99 | 28 | 4.1 | 717.34 | 3 | 64.8 | 2701 | 2728 |  | PEAKS DB |
| Y.GHGYGSDGGAAAAAAAAAAAAAGGAGR.G | 74.81 | 2126.984 | 27 | 3.4 | 710.0044 | 3 | 73.8 | 288 | 314 |  | PEAKS DB |
| R.APTGHETIYEEDVVIKQVPQ.G | 74.61 | 2252.132 | 20 | 3.8 | 751.7209 | 3 | 72.9 | 85 | 104 |  | PEAKS DB |
| Y.GHGYGSDGGAAAAAAAAAAAAAGGAGSGY.G | 73.95 | 2278 | 29 | 3.8 | 760.3434 | 3 | 78.7 | 1802 | 1830 |  | PEAKS DB |
| A.GSGRSHGAGSAAGAAAAAAAAAAAAGGAGRGGGGGY.G | 73.23 | 2769.304 | 36 | 4 | 693.3361 | 4 | 72.7 | 135 | 170 |  | PEAKS DB |
| Y.GRGHGTAGSAAAAAAAAAAAAASSEGGSAGGYW.Q | 71.15 | 2760.26 | 33 | 4 | 921.0976 | 3 | 84.6 | 171 | 203 |  | PEAKS DB |
| R.SAAAAAAAAAASSGARGAVR.V | 71.12 | 1656.866 | 20 | 4.1 | 553.298 | 3 | 58.6 | 2802 | 2821 |  | PEAKS DB |
| Y.GHGYGSDGGAAAAAAAAAAAAAGGAGGGY.G | 71.04 | 2247.989 | 29 | 3.8 | 750.3398 | 3 | 78.9 | 373 | 401 |  | PEAKS DB |
| R.SHGAGSAAGAAAAAAAAAAAAGGAGR.G | 70.33 | 1963.957 | 26 | 3.3 | 655.6618 | 3 | 71.9 | 139 | 164 |  | PEAKS DB |
| E.RAPTGHETIYEEDVVIK.Q | 70.16 | 1955.995 | 17 | 4.2 | 653.0084 | 3 | 65.4 | 84 | 100 |  | PEAKS DB |
| Y.GSDSGAAAAAAAAAAAAAGGAGSGY.G | 69.98 | 1893.845 | 25 | 4 | 947.9337 | 2 | 77.5 | 2569 | 2593 |  | PEAKS DB |
| R.GAVRVHETGDGF.L | 69.8 | 1243.595 | 12 | 4.1 | 622.8071 | 2 | 52.8 | 2818 | 2829 |  | PEAKS DB |
| K.Q(-17.03)VPQGGAASSAASSASAGSGSGAPTIIVER.G | 69.39 | 2653.294 | 30 | 4.8 | 885.4429 | 3 | 70 | 101 | 130 | Pyro-glu from Q | PEAKS PTM |
| G.SGRSHGAGSAAGAAAAAAAAAAAAGGAGR.G | 69.23 | 2264.112 | 29 | 4.1 | 755.7143 | 3 | 69.4 | 136 | 164 |  | PEAKS DB |
| Y.GYGSDSSAAAAAAAAAAGGAGGGY.G | 68.82 | 1900.819 | 24 | 4.3 | 951.4207 | 2 | 70.1 | 932 | 955 |  | PEAKS DB |
| R.DSAPSRVPGGGTLVEK.I | 68.8 | 1568.816 | 16 | 3.2 | 523.9476 | 3 | 54.9 | 64 | 79 |  | PEAKS DB |
| Y.GSNSGAAAAAAAAAAAGGAGSGY.G | 68.75 | 1750.787 | 23 | 4.4 | 876.4047 | 2 | 67.8 | 2701 | 2723 |  | PEAKS DB |
| W.HSYGSDSGAAAAAAAAAAAAAGGAGSGYGGGSR.G | 68.19 | 2695.197 | 33 | 4.4 | 899.4102 | 3 | 74.3 | 2566 | 2598 |  | PEAKS DB |
| Y.GGGYGHGYGSDGGAAAAAAAAAAAAAGGAGR.G | 67.63 | 2461.112 | 31 | 4.7 | 821.3817 | 3 | 75.5 | 284 | 314 |  | PEAKS DB |
| Q.GGAASSAASSASAGSGSGAPTIIVER.G | 67.6 | 2218.083 | 26 | 5.3 | 740.3721 | 3 | 63.7 | 105 | 130 |  | PEAKS DB |
| R.APTGHETIYEED(-18.01)VVIK.Q | 67.35 | 1781.884 | 16 | 3.5 | 594.9705 | 3 | 75.2 | 85 | 100 | Dehydration | PEAKS PTM |
| Y.GSDGGAAAAAAAAAAAAAGGSGGGY.G | 67.28 | 1849.819 | 25 | 4.2 | 925.9207 | 2 | 75.9 | 579 | 603 |  | PEAKS DB |
| I.KQVPQGGAASSAASSASAGSGSGAPTIIVER.G | 66.67 | 2798.416 | 31 | 4.5 | 933.8167 | 3 | 63.1 | 100 | 130 |  | PEAKS DB |
| R.APTN(sub G)HETIYEEDVVIK.Q | 66.53 | 1856.916 | 16 | 4.5 | 929.4692 | 2 | 67.8 | 85 | 100 |  | SPIDER |
| H.GAGSAAGAAAAAAAAAAAAGGAGRGGGGGY.G | 66.23 | 2188.037 | 30 | 4.2 | 730.356 | 3 | 77.4 | 141 | 170 |  | PEAKS DB |
| K.IVIERAPTGHETIYEEDVVI.K | 65.72 | 2282.179 | 20 | 3.1 | 761.7361 | 3 | 82.4 | 80 | 99 |  | PEAKS DB |
| R.SAAAAAAAAAASSGAR.G | 65.55 | 1273.638 | 16 | 2.4 | 637.8275 | 2 | 52.5 | 2802 | 2817 |  | PEAKS DB |
| R.SLDDDIHSLER.G | 64.47 | 1298.61 | 11 | 4.4 | 650.3152 | 2 | 63.3 | 20 | 30 |  | PEAKS DB |
| R.VPGGGTLVEK(+43.01)IVIER.A | 64.02 | 1608.92 | 15 | 3.9 | 537.316 | 3 | 88.6 | 70 | 84 | Carbamylation | PEAKS PTM |
| K.TGYDEYDVDK.S | 63.75 | 1203.493 | 10 | 3 | 602.7557 | 2 | 51.5 | 38 | 47 |  | PEAKS DB |
| K.TGYDEYDVDKSGRLY.E | 63.3 | 1779.795 | 15 | 4.7 | 890.9091 | 2 | 64.8 | 38 | 52 |  | SPIDER |
| R.AP(+31.99)TGHETIYEEDVVIK.Q | 63.24 | 1831.884 | 16 | 4.6 | 916.9534 | 2 | 72.8 | 85 | 100 | Dihydroxy | PEAKS PTM |
| R.SAAAAAAAAAASSGARGAVRVHETGDGF.L | 63.02 | 2499.221 | 28 | 4.9 | 834.0851 | 3 | 69.9 | 2802 | 2829 |  | PEAKS DB |
| F.ERDSAPSRVPGGGTLVEKIVIER.A | 62.93 | 2464.34 | 23 | 4.1 | 493.8772 | 5 | 76.8 | 62 | 84 |  | SPIDER |
| R.DSAPSRVPGGGTLVEKIVIER.A | 62.43 | 2179.196 | 21 | 3.2 | 727.4083 | 3 | 79.4 | 64 | 84 |  | SPIDER |
| R.VPGGGTLVEK(+14.02)IVIER.A | 62.33 | 1579.93 | 15 | 1.6 | 527.6514 | 3 | 80.8 | 70 | 84 | Methylation(KR) | PEAKS PTM |
| Y.GSDNAAAAAAAAAAAAGGAGGDY.G | 62.29 | 1820.793 | 23 | 4.1 | 911.4073 | 2 | 74 | 2772 | 2794 |  | PEAKS DB |
| R.APTGHETIYEEDVVIKQVPQGGAAS.S | 62.25 | 2595.282 | 25 | 4.3 | 866.1049 | 3 | 71.1 | 85 | 109 |  | PEAKS DB |
| R.APTGHET(-18.01)IYEEDVVIK.Q | 62.18 | 1781.884 | 16 | -0.6 | 594.9681 | 3 | 68.2 | 85 | 100 | Dehydration | PEAKS PTM |
| Y.GARSAAAAAAAAAASSGAR.G | 62.02 | 1557.797 | 19 | 2.8 | 520.2744 | 3 | 55.8 | 2799 | 2817 |  | PEAKS DB |
| Y.RETSKTGYDEYDVDKSGRLY.E | 61.85 | 2381.114 | 20 | 2.1 | 596.2869 | 4 | 59.8 | 33 | 52 |  | SPIDER |
| S.SASAGSGSGAPTIIVER.G | 61.78 | 1558.795 | 17 | 3.2 | 780.4073 | 2 | 62 | 114 | 130 |  | PEAKS DB |
| Y.GSDSSAAAAAAAAAAGGAGGGY.G | 61.61 | 1680.734 | 22 | 4.2 | 841.3777 | 2 | 66.2 | 934 | 955 |  | PEAKS DB |
| Y.GGDGGAAAAAAAAAAAAGGAGSGY.G | 61.5 | 1762.787 | 24 | 3.7 | 882.4041 | 2 | 73.7 | 1153 | 1176 |  | PEAKS DB |
| Y.GHGYGSDGGAAAAAAAAAAAAAGGAGRGY.G | 61.28 | 2347.069 | 29 | 4.4 | 783.367 | 3 | 78.3 | 288 | 316 |  | PEAKS DB |
| F.ERDSAPSRVPGGGTLVEK.I | 61.06 | 1853.96 | 18 | 3.2 | 464.4986 | 4 | 54.4 | 62 | 79 |  | SPIDER |
| Y.GGDGGAAAAAAAAAAAAAGGAGGGY.G | 60.92 | 1803.814 | 25 | 4.3 | 902.9179 | 2 | 78.2 | 728 | 752 |  | PEAKS DB |
| R.SLDDDIHS(+79.97)LER.G | 60.68 | 1378.577 | 11 | 3.5 | 690.298 | 2 | 65.2 | 20 | 30 | Phosphorylation (STY) | PEAKS PTM |
| R.GAVRVHETGDGFL.L | 60.42 | 1356.679 | 13 | 3.3 | 453.235 | 3 | 67.1 | 2818 | 2830 |  | PEAKS DB |
| R.DSAPSRVPGGGTL.V | 60.37 | 1212.61 | 13 | 3.4 | 607.3143 | 2 | 57.7 | 64 | 76 |  | PEAKS DB |
| Y.GARSAAAAAAAAAASSGARGAVR.V | 59.61 | 1941.025 | 23 | 1.1 | 648.0164 | 3 | 59.9 | 2799 | 2821 |  | PEAKS DB |
| R.APTGHETIYEEDVV.I | 59.43 | 1558.715 | 14 | 4.5 | 780.3683 | 2 | 67.4 | 85 | 98 |  | PEAKS DB |
| A.SSASAGSGSGAPTIIVER.G | 59.27 | 1645.827 | 18 | 2.6 | 823.9229 | 2 | 62.1 | 113 | 130 |  | PEAKS DB |
| Y.GGDGGAAAAAAAAAAAAAGGAGSGY.G | 59.17 | 1833.824 | 25 | 3.8 | 917.9229 | 2 | 77.7 | 410 | 434 |  | PEAKS DB |
| R.APTGHETIYEEDVVIKQVPQGGAASSAAS.S | 58.77 | 2911.42 | 29 | 2.7 | 971.4832 | 3 | 70.9 | 85 | 113 |  | PEAKS DB |
| Y.GAGSAAAAAAAAAAAAGGAGGGY.G | 58.42 | 1661.776 | 23 | 4.3 | 831.8987 | 2 | 74.4 | 519 | 541 |  | PEAKS DB |
| A.RSLDDDIHSLERGY.R | 58.19 | 1674.796 | 14 | 3.6 | 559.2747 | 3 | 69.1 | 19 | 32 |  | SPIDER |
| W.QGYGSNSGAAAAAAAAAAAGGAGSGYGGGAR.G | 57.36 | 2497.133 | 31 | 3.7 | 833.3881 | 3 | 68.9 | 2698 | 2728 |  | PEAKS DB |
| H.GAGSAAGAAAAAAAAAAAAGGAGR.G | 57.34 | 1739.866 | 24 | 4.2 | 580.9651 | 3 | 74.8 | 141 | 164 |  | PEAKS DB |
| S.AASSASAGSGSGAPTIIVER.G | 57.25 | 1787.901 | 20 | 5.1 | 894.9625 | 2 | 62.4 | 111 | 130 |  | PEAKS DB |
| R.VPGGGTLVEKIVIER.A | 57.21 | 1565.914 | 15 | 0.3 | 783.9645 | 2 | 80.4 | 70 | 84 |  | PEAKS DB |
| R.VHETGDGFLLRGDY.G | 57.19 | 1577.747 | 14 | 4.2 | 526.9252 | 3 | 75.5 | 2822 | 2835 |  | SPIDER |
| R.A(+43.01)PTGHETIYEEDVVIK.Q | 56.74 | 1842.9 | 16 | 4.4 | 922.4613 | 2 | 73.1 | 85 | 100 | Carbamylation | PEAKS PTM |
| R.APTGHET(+114.04)IYEEDVVIK.Q | 56.65 | 1913.937 | 16 | 3.9 | 638.9888 | 3 | 67.6 | 85 | 100 | Ubiquitin | PEAKS PTM |
| R.APTGHETIYEEDVVIKQVPQG.G | 56.23 | 2309.154 | 21 | 3.9 | 770.7282 | 3 | 72.7 | 85 | 105 |  | PEAKS DB |
| R.SHGQ(sub A)GSAAGAAAAAAAAAAAAGGAGR.G | 56.22 | 2020.979 | 26 | 3.1 | 674.6689 | 3 | 71.9 | 139 | 164 |  | SPIDER |
| R.GAVRVHETGDGFLL.R | 56.17 | 1469.763 | 14 | 3.2 | 735.891 | 2 | 79 | 2818 | 2831 |  | SPIDER |
| R.GAVRVHETGDGFLLR.G | 55.89 | 1625.864 | 15 | 3.8 | 542.9639 | 3 | 69.1 | 2818 | 2832 |  | SPIDER |
| Y.GSDNAAAAAAAAAAAAGGAGGDYGRGY.G | 55.76 | 2254 | 27 | 4 | 752.3435 | 3 | 75.5 | 2772 | 2798 |  | PEAKS DB |
| Y.GSDSGAAAAAAAAAAAAAGGAGGGY.G | 55.11 | 1863.835 | 25 | 4 | 932.9283 | 2 | 77.7 | 1982 | 2006 |  | PEAKS DB |
| R.VHETGDGF.L | 55.07 | 860.3665 | 8 | 3.1 | 431.1918 | 2 | 45.2 | 2822 | 2829 |  | PEAKS DB |
| K.IVIERAPTN(sub G)HETIY.E | 55.05 | 1654.868 | 14 | 4.3 | 828.4448 | 2 | 65.3 | 80 | 93 |  | SPIDER |
| R.VPGGGTLVEK(+58.01)IVIER.A | 55.04 | 1623.92 | 15 | 3.1 | 542.3155 | 3 | 83.2 | 70 | 84 | Carboxymethyl (KW, X@N-term) | PEAKS PTM |
| A.SAGSGSGAPTIIVER.G | 55 | 1400.726 | 15 | 2.9 | 701.3723 | 2 | 61.7 | 116 | 130 |  | PEAKS DB |
| T.GHETIYEEDVVIKQVPQGGAASSAASSASAGSGSGAPTIIVER.G | 54.97 | 4183.067 | 43 | 3.7 | 1046.778 | 4 | 77.3 | 88 | 130 |  | PEAKS DB |
| A.AAGGAGGGAGRGAIGGY.G | 54.86 | 1318.638 | 17 | 3.1 | 660.3282 | 2 | 52 | 2751 | 2767 |  | PEAKS DB |
| Y.RETSKTGYDEYDVDKSGR.L | 54.67 | 2104.966 | 18 | 3 | 527.2504 | 4 | 44.7 | 33 | 50 |  | SPIDER |
| R.VPGGGTLVEKIVIERAPTGHETIY.E | 54.58 | 2535.37 | 24 | 3.5 | 846.1335 | 3 | 85 | 70 | 93 |  | SPIDER |
| R.APTGHETIY.E | 54.23 | 987.4661 | 9 | 1.1 | 494.7409 | 2 | 50 | 85 | 93 |  | PEAKS DB |
| Y.GSDGGAAAAAAAAAAAAAGGAGGGY.G | 54.14 | 1833.824 | 25 | 4.4 | 917.9233 | 2 | 77.6 | 377 | 401 |  | PEAKS DB |
| G.DGGAAAAAAAAAAAAAGGAGSGY.G | 53.96 | 1719.781 | 23 | 3.7 | 860.9011 | 2 | 77.3 | 412 | 434 |  | PEAKS DB |
| K.TGYDEYDVDKSGRLYER.L | 53.83 | 2064.939 | 17 | 3.6 | 517.2438 | 4 | 60.8 | 38 | 54 |  | SPIDER |
| Y.GGDGGAAAAAAAAAAAAAGGAGSGY(+21.98).G | 53.71 | 1855.806 | 25 | 3.5 | 928.9136 | 2 | 77.9 | 410 | 434 | Sodium adduct | PEAKS PTM |
| R.VHETGDGFLLR.G | 53.42 | 1242.636 | 11 | 3.6 | 415.2206 | 3 | 67.4 | 2822 | 2832 |  | SPIDER |
| R.GYRETSKTGYDEYDVDK.S | 53.24 | 2024.896 | 17 | 3.7 | 675.9752 | 3 | 50.8 | 31 | 47 |  | SPIDER |
| R.DSAPSRVPGGGTLVEKIR(sub V)IER.A | 53.15 | 2236.229 | 21 | -1.5 | 746.4158 | 3 | 79.5 | 64 | 84 |  | SPIDER |
| K.IVIERAPTGHET.I | 53.08 | 1321.699 | 12 | 2.8 | 661.8586 | 2 | 50.9 | 80 | 91 |  | PEAKS DB |
| S.RVPGGGTLVEKIVIER.A | 52.97 | 1722.015 | 16 | 3.9 | 431.5128 | 4 | 76 | 69 | 84 |  | SPIDER |
| A.RSLDDDIHSL.E | 52.85 | 1169.568 | 10 | 3.8 | 390.8646 | 3 | 61.1 | 19 | 28 |  | PEAKS DB |
| Y.GSDGGAAAAAAAAAAAAAGGAGSGY.G | 52.68 | 1863.835 | 25 | 3.5 | 932.9279 | 2 | 77.8 | 1806 | 1830 |  | PEAKS DB |
| R.APT(+114.04)GHETIYEEDVVIK.Q | 52.56 | 1913.937 | 16 | 3.6 | 638.9886 | 3 | 67.8 | 85 | 100 | Ubiquitin | PEAKS PTM |
| R.Q(sub A)PTGHETIYEEDVVIK.Q | 52.23 | 1856.916 | 16 | 3.5 | 619.9813 | 3 | 66.1 | 85 | 100 |  | SPIDER |
| R.VPGGGTLVEK(-1.03)IVIER.A | 52.21 | 1564.882 | 15 | 0.5 | 783.4489 | 2 | 90.5 | 70 | 84 | Lysine oxidation to aminoadipic semialdehyde | PEAKS PTM |
| Y.GSDSGAAAAAAAAAAAAAAGGSGGGY.G | 52.2 | 1950.867 | 26 | 4.6 | 976.4451 | 2 | 78.4 | 443 | 468 |  | PEAKS DB |
| R.APTGHETIYE(+21.98)EDVVIK.Q | 52.19 | 1821.876 | 16 | 0.3 | 911.9456 | 2 | 68.2 | 85 | 100 | Sodium adduct | PEAKS PTM |
| A.AAAAGGAGGDYGRGY.G | 52.1 | 1312.58 | 15 | 4.1 | 657.2997 | 2 | 47.5 | 2784 | 2798 |  | PEAKS DB |
| R.DS(-18.01)APSRVPGGGTLVEK.I | 52.08 | 1550.805 | 16 | 4.5 | 517.9446 | 3 | 55 | 64 | 79 | Dehydration | PEAKS PTM |
| V.PGGGTLVEKIVIER.A | 51.95 | 1466.846 | 14 | 1.7 | 489.9566 | 3 | 80.5 | 71 | 84 |  | PEAKS DB |
| R.D(+43.01)SAPSRVPGGGTLVEK.I | 51.8 | 1611.822 | 16 | 4.1 | 806.9213 | 2 | 58.4 | 64 | 79 | Carbamylation | PEAKS PTM |
| R.DSAPSRVPGGGTLVE.K | 51.79 | 1440.721 | 15 | 2.7 | 721.3696 | 2 | 60.4 | 64 | 78 |  | PEAKS DB |
| R.VPGGGTLVEK.I | 51.74 | 955.5338 | 10 | 3.4 | 478.7758 | 2 | 51.7 | 70 | 79 |  | PEAKS DB |
| G.S(+114.04)DSAAAAAAAAAAAAAGGAGGGAGR.G | 51.59 | 1984.931 | 25 | 3.4 | 662.6532 | 3 | 72.4 | 2737 | 2761 | Ubiquitin | PEAKS PTM |
| S.RVPGGGTLVEK.I | 51.16 | 1111.635 | 11 | 4 | 371.5537 | 3 | 47.9 | 69 | 79 |  | PEAKS DB |
| Y.GSNSGAAAAAAAAAAAGGAGSGY(+21.98).G | 51.1 | 1772.769 | 23 | 4.8 | 887.396 | 2 | 67.9 | 2701 | 2723 | Sodium adduct | PEAKS PTM |
| G.HGYGS(+114.04)DSAAAAAAAAAAAAAGGAGGGAGRGAIGGY.G | 51.04 | 2917.345 | 35 | 1.5 | 973.4571 | 3 | 81 | 2733 | 2767 | Ubiquitin | PEAKS PTM |
| R.APTGHETIY(+125.90)EEDVVIK.Q | 50.99 | 1925.791 | 16 | 3.4 | 642.9397 | 3 | 76.6 | 85 | 100 | Iodination | PEAKS PTM |
| Y.GARSAAAAAAAAAASSGARGAVRVHETGDGF.L | 50.83 | 2783.381 | 31 | 4.8 | 696.8559 | 4 | 69.8 | 2799 | 2829 |  | SPIDER |
| Y.DEYDVDKSGRLY.E | 50.38 | 1458.663 | 12 | 4.3 | 730.3417 | 2 | 61.3 | 41 | 52 |  | SPIDER |
| E.RAPTGHETIY.E | 50.28 | 1143.567 | 10 | 4.1 | 382.1979 | 3 | 47.5 | 84 | 93 |  | PEAKS DB |
| A.RSLDDDIHSLER.G | 50.23 | 1454.711 | 12 | 3.9 | 485.9129 | 3 | 64.4 | 19 | 30 |  | SPIDER |
| G.GYGHGYGS(+114.04)DSAAAAAAAAAAAAAGGAGGGAGR.G | 50.17 | 2676.202 | 32 | 4.3 | 893.0786 | 3 | 75.4 | 2730 | 2761 | Ubiquitin | PEAKS PTM |
| R.D(+27.99)SAPSRVPGGGTLVEK.I | 50.1 | 1596.811 | 16 | 3.5 | 799.4154 | 2 | 58.8 | 64 | 79 | Formylation | PEAKS PTM |
| G.SGAPTIIVER.G | 49.78 | 1041.582 | 10 | 3.1 | 521.7998 | 2 | 61.4 | 121 | 130 |  | PEAKS DB |
| F.E(-18.01)RDSAPSRVPGGGTL.V | 49.75 | 1479.743 | 15 | 4.1 | 740.8818 | 2 | 59.1 | 62 | 76 | Pyro-glu from E | PEAKS PTM |
| K.TGYDEYDVDKSGR.L | 49.57 | 1503.648 | 13 | 3.3 | 502.2249 | 3 | 47.7 | 38 | 50 |  | SPIDER |
| R.DSAPSRVPGGGTLVEKR(sub I)VIER.A | 49.56 | 2222.213 | 21 | -1.4 | 741.744 | 3 | 87 | 64 | 84 |  | SPIDER |
| T.GHETIYEEDVVI.K | 49.55 | 1402.662 | 12 | 4 | 702.3409 | 2 | 77.7 | 88 | 99 |  | PEAKS DB |
| S.RVPGGGT(-18.01)LVEK.I | 49.53 | 1093.624 | 11 | 3.4 | 365.55 | 3 | 48.1 | 69 | 79 | Dehydration | PEAKS PTM |
| G.GYGHGYGSDS(+114.04)AAAAAAAAAAAAAGGAGGGAGR.G | 49.44 | 2676.202 | 32 | 3.9 | 893.0782 | 3 | 75.4 | 2730 | 2761 | Ubiquitin | PEAKS PTM |
| D.SAPSRVPGGGTLVEK.I | 49.35 | 1453.789 | 15 | 2.8 | 485.6049 | 3 | 53.4 | 65 | 79 |  | PEAKS DB |
| R.APTGHETIYEEDVVIKQVPQGGAASSAASSASAGSGSGAPT.I | 49.27 | 3841.824 | 41 | 7.5 | 1281.625 | 3 | 71.1 | 85 | 125 |  | PEAKS DB |
| R.VPGGGTLVEKIVIE.R | 49.2 | 1409.813 | 14 | 3.4 | 705.9161 | 2 | 84.4 | 70 | 83 |  | PEAKS DB |
| F.ERDSAPSRVPGGGTL.V | 49.19 | 1497.754 | 15 | 3.1 | 500.26 | 3 | 56.7 | 62 | 76 |  | PEAKS DB |
| S.RVPGGGTLVEKIVIERAPT.G | 49.04 | 1991.153 | 19 | 3.8 | 498.7973 | 4 | 78 | 69 | 87 |  | SPIDER |
| F.ERDSAPSRVPGGGTLVEKIR(sub V)IER.A | 48.94 | 2521.372 | 23 | -0.5 | 505.2815 | 5 | 76.5 | 62 | 84 |  | SPIDER |
| A.PSRVPGGGTLVEK.I | 48.52 | 1295.72 | 13 | 3.5 | 432.9154 | 3 | 55 | 67 | 79 |  | PEAKS DB |
| F.ERDSAPSRVPGGGTLVER(sub K)IVIER.A | 48.43 | 2492.346 | 23 | -0.5 | 624.0934 | 4 | 84.7 | 62 | 84 |  | SPIDER |
| R.VHETGDGFL.L | 48.25 | 973.4505 | 9 | 3.9 | 487.7344 | 2 | 64.9 | 2822 | 2830 |  | PEAKS DB |
| R.APTN(sub G)HETIY.E | 48.02 | 1044.488 | 9 | 3.7 | 523.253 | 2 | 49.7 | 85 | 93 |  | SPIDER |
| A.AAAAAAAAAGGAGGGY.G | 47.94 | 1176.552 | 16 | 3.3 | 589.2853 | 2 | 49.7 | 386 | 401 |  | PEAKS DB |
| Y.RETSKTGYDEYDVDK.S | 47.68 | 1804.812 | 15 | 4.6 | 452.2122 | 4 | 45.3 | 33 | 47 |  | SPIDER |
| E.KIVIERAPTGHETIY.E | 47.56 | 1725.941 | 15 | 3.5 | 576.3231 | 3 | 63.2 | 79 | 93 |  | PEAKS DB |
| K.TGYDEYDVDKSGRLYERLTTR.K | 47.55 | 2536.219 | 21 | 3.5 | 635.0643 | 4 | 70.8 | 38 | 58 |  | SPIDER |
| R.SGYGGGSWHGY.G | 47.42 | 1126.447 | 11 | 3.7 | 564.2327 | 2 | 61 | 1032 | 1042 |  | PEAKS DB |
| A.AAAAAAAAGGAGGGY.G | 47.33 | 1105.515 | 15 | 3.7 | 553.7669 | 2 | 46.8 | 387 | 401 |  | PEAKS DB |
| S.SQ(sub A)SAGSGSGAPTIIVER.G | 47.33 | 1615.817 | 17 | 3.8 | 808.9186 | 2 | 62.3 | 114 | 130 |  | SPIDER |
| S.RVPGGGTLVEKIVIERAPTGH.E | 47.16 | 2185.233 | 21 | 3.9 | 547.3177 | 4 | 74.9 | 69 | 89 |  | SPIDER |
| Y.RETSKTGYDEY.D | 47.14 | 1347.594 | 11 | 4.1 | 674.8072 | 2 | 42.8 | 33 | 43 |  | SPIDER |
| G.HGYGSDS(+114.04)AAAAAAAAAAAAAGGAGGGAGR.G | 46.83 | 2399.096 | 29 | 3.4 | 800.7087 | 3 | 73.7 | 2733 | 2761 | Ubiquitin | PEAKS PTM |
| F.ERDSAPSR.V | 46.71 | 916.4362 | 8 | 4.8 | 306.4875 | 3 | 28 | 62 | 69 |  | PEAKS DB |
| L.VEKIVIERAPTGHETIY.E | 46.65 | 1954.052 | 17 | 3.8 | 489.5222 | 4 | 66.8 | 77 | 93 |  | PEAKS DB |
| G.HGYGS(+114.04)DSAAAAAAAAAAAAAGGAGGGAGR.G | 46.59 | 2399.096 | 29 | 3.4 | 800.7087 | 3 | 73.8 | 2733 | 2761 | Ubiquitin | PEAKS PTM |
| Y.EEDVVIKQVPQGGAASSAASSASAGSGSGAPT.I | 46.39 | 2872.369 | 32 | 6.3 | 958.4695 | 3 | 61.1 | 94 | 125 |  | PEAKS DB |
| R.SAAAAAAAAAASSGARGAVRVH.E | 46.35 | 1892.993 | 22 | 4 | 474.2574 | 4 | 61.3 | 2802 | 2823 |  | PEAKS DB |
| V.PGGGTLVEK.I | 45.95 | 856.4654 | 9 | 3.7 | 429.2415 | 2 | 49.8 | 71 | 79 |  | PEAKS DB |
| S.RVPGGGTLVEKIVIERAPTGHETIY.E | 45.55 | 2691.471 | 25 | 2.8 | 539.3029 | 5 | 81.5 | 69 | 93 |  | SPIDER |
| G.SGSGAPTIIVER.G | 45.47 | 1185.635 | 12 | 3.8 | 593.8271 | 2 | 61.3 | 119 | 130 |  | PEAKS DB |
| K.QVPQGGAASSAASSASAGSGSGAPT.I | 45.22 | 2059.941 | 25 | 3.9 | 687.6569 | 3 | 47.7 | 101 | 125 |  | PEAKS DB |
| A.PSRVPGGGTLVEKIVIER.A | 45.07 | 1906.1 | 18 | 2.2 | 477.5333 | 4 | 77.3 | 67 | 84 |  | SPIDER |
| F.ERDSAPSRVPGGGTLVEKI.V | 45.03 | 1967.044 | 19 | 2.2 | 492.7693 | 4 | 68.4 | 62 | 80 |  | SPIDER |
| Y.DVDKSGRLY.E | 44.94 | 1051.53 | 9 | 2.1 | 526.7733 | 2 | 50.2 | 44 | 52 |  | SPIDER |
| F.ERDSAPSRVPGGGTLVEKR(sub I)VIER.A | 44.73 | 2507.357 | 23 | -0.1 | 627.8464 | 4 | 84.1 | 62 | 84 |  | SPIDER |
| Y.RW(sub E)TSKTGYDEYDVDKSGRLY.E | 44.66 | 2438.15 | 20 | -4.1 | 610.5423 | 4 | 60.4 | 33 | 52 |  | SPIDER |
| R.APTGHETIYE(+37.95)EDVVIK.Q | 44.4 | 1837.841 | 16 | 1 | 460.468 | 4 | 68 | 85 | 100 | Replacement of 2 protons by calcium | PEAKS PTM |
| A.AAGGAGGDYGRGY.G | 44.39 | 1170.505 | 13 | 2.9 | 586.2617 | 2 | 45.6 | 2786 | 2798 |  | PEAKS DB |
| R.VPGGGTLVEK(+43.01)IVIER(+14.02).A | 44.32 | 1622.936 | 15 | 3.4 | 541.9876 | 3 | 79.9 | 70 | 84 | Carbamylation; Methylation(KR) | PEAKS PTM |
| R.R(sub V)HETGDGFLLR.G | 44.07 | 1299.668 | 11 | -5.2 | 434.2278 | 3 | 67.6 | 2822 | 2832 |  | SPIDER |
| K.QVPQGGAASSAASSASAGSGSGAPTIIVE(+21.98)R.G | 43.76 | 2692.303 | 30 | 2.9 | 898.4442 | 3 | 65.6 | 101 | 130 | Sodium adduct | PEAKS PTM |
| F.ERDSAPSRVPGGGT(-18.01)L.V | 43.73 | 1479.743 | 15 | 3.6 | 494.2567 | 3 | 56.3 | 62 | 76 | Dehydration | PEAKS PTM |
| R.DSAPSRVPGGGTR(sub L)VEKIVIER.A | 43.59 | 2222.213 | 21 | -1.8 | 741.7437 | 3 | 87.1 | 64 | 84 |  | SPIDER |
| A.AAAAAAGGAGRGY.G | 43.49 | 1062.521 | 13 | 4 | 532.2697 | 2 | 40.6 | 304 | 316 |  | PEAKS DB |
| P.G(sub S)RVPGGGTLVEKIVIER.A | 43.48 | 1779.037 | 17 | 3.2 | 445.7679 | 4 | 76.6 | 68 | 84 |  | SPIDER |
| Y.RETSKTN(sub G)YDEYDVDK.S | 43.25 | 1861.833 | 15 | 2.9 | 621.6201 | 3 | 45.6 | 33 | 47 |  | SPIDER |
| L.VEKIVIER.A | 43.17 | 984.5967 | 8 | 3.9 | 329.2075 | 3 | 53.7 | 77 | 84 |  | PEAKS DB |
| F.ERDSAPSRVPG.G | 43.13 | 1169.579 | 11 | 3.7 | 390.8683 | 3 | 39.5 | 62 | 72 |  | PEAKS DB |
| A.AAAAAAAAGGAGSGY.G | 42.96 | 1135.526 | 15 | 3 | 568.7719 | 2 | 46.8 | 420 | 434 |  | PEAKS DB |
| T.G(sub A)RSLDDDIHSLERGY.R | 42.81 | 1731.818 | 15 | 3.9 | 578.282 | 3 | 69.5 | 18 | 32 |  | SPIDER |
| R.APTGHETIYEEDVVIKQVPQG(-.98).G | 42.72 | 2308.17 | 21 | 4.2 | 770.4005 | 3 | 71.2 | 85 | 105 | Amidation | PEAKS PTM |
| L.VEKIVIERAPT.G | 42.65 | 1253.734 | 11 | 3.7 | 418.9203 | 3 | 58.3 | 77 | 87 |  | PEAKS DB |
| K.SGRLYERLTTR.K | 42.4 | 1350.737 | 11 | 2.4 | 338.6923 | 4 | 55.7 | 48 | 58 |  | SPIDER |
| Y.GGGYGHGY.G | 42.33 | 766.3034 | 8 | 3.9 | 384.1605 | 2 | 40.5 | 284 | 291 |  | PEAKS DB |
| Y.GSDGGAAAAAAAAAAAAAGGAGGGY(+21.98).G | 42.15 | 1855.806 | 25 | 3.9 | 928.9139 | 2 | 78.1 | 377 | 401 | Sodium adduct | PEAKS PTM |
| Y.GGGSWHGY.G | 42.13 | 819.33 | 8 | 3.7 | 410.6738 | 2 | 54 | 1035 | 1042 |  | PEAKS DB |
| F.ERDSAPSRVPGGGTLVE.K | 42.08 | 1725.865 | 17 | 4.5 | 863.9434 | 2 | 58.4 | 62 | 78 |  | SPIDER |
| Y.RETSKTN(sub G)YDEYDVDKSGRLY.E | 42.08 | 2438.135 | 20 | 2.4 | 610.5425 | 4 | 60.5 | 33 | 52 |  | SPIDER |
| I.VIERAPTGHETIY.E | 41.85 | 1484.762 | 13 | 3.7 | 495.9299 | 3 | 57.3 | 81 | 93 |  | PEAKS DB |
| R.LGTSKIAVH | 41.85 | 924.5392 | 9 | 4.3 | 309.1884 | 3 | 47.2 | 2872 | 2880 |  | PEAKS DB |
| Y.GSDGGAAAAAAAAAAAAAGGAGGGY(+37.95).G | 41.37 | 1871.771 | 25 | 3.1 | 624.9329 | 3 | 78.1 | 377 | 401 | Replacement of 2 protons by calcium | PEAKS PTM |
| R.ETSKTGYDEYDVDK.S | 41.27 | 1648.71 | 14 | 3.3 | 550.5792 | 3 | 49.4 | 34 | 47 |  | SPIDER |
| A.AAAAAAAGGAGGGY.G | 41.1 | 1034.478 | 14 | -0.4 | 518.2461 | 2 | 44.3 | 388 | 401 |  | PEAKS DB |
| Y.RETSKTGY.D | 40.89 | 940.4614 | 8 | 4.7 | 471.2402 | 2 | 30.4 | 33 | 40 |  | PEAKS DB |
| A.RSLDDDIHS(+79.97)L.E | 40.87 | 1249.534 | 10 | 3.1 | 625.7762 | 2 | 67.9 | 19 | 28 | Phosphorylation (STY) | PEAKS PTM |
| F.ERDSAPP(sub S)Q(sub R)VPGGGTLVEK.I | 40.77 | 1835.938 | 18 | 8.7 | 459.9957 | 4 | 53.1 | 62 | 79 |  | SPIDER |
[truncated: 96,714 more chars]
